# Global burden and trends of leukemia attributable to high body mass index risk in adults over the past 30 years

**DOI:** 10.3389/fonc.2024.1404135

**Published:** 2024-06-19

**Authors:** Hang Xiao, Xiao Hu, Pengfei Li, Jianchuan Deng

**Affiliations:** Department of Hematology, The Second Affiliated Hospital of Chongqing Medical University, Chongqing, China

**Keywords:** high BMI, leukemia, global burden disease, epidemiological trends, disease prevention

## Abstract

**Background:**

High BMI (Body Mass Index) is a significant factor impacting health, with a clear link to an increased risk of leukemia. Research on this topic is limited. Understanding the epidemiological trends of leukemia attributable to high BMI risk is crucial for disease prevention and patient support.

**Methods:**

We obtained the data from the Global Burden of Disease Study, analyzing the ASR (age-standardized rates), including ASDR (age-standardized death rate) and age-standardized disability-adjusted life years (DALYs) rate, and estimated annual percentage change (EAPC) by gender, age, country, and region from 1990 to 2019.

**Results:**

In 2019, deaths and DALYs have significantly increased to 21.73 thousand and 584.09 thousand. The global age-standardized death and DALYs rates have slightly increased over the past 30 years (EAPCs: 0.34 and 0.29). Among four common leukemia subtypes, only CML (Chronic Myeloid Leukemia) exhibited a significant decrease in ASDR and age-standardized DALYs rate, with EAPC of -1.74 and -1.52. AML (Acute Myeloid Leukemia) showed the most pronounced upward trend in ASDR, with an EAPC of 1.34. These trends vary by gender, age, region, and national economic status. Older people have been at a significantly greater risk. Females globally have borne a higher burden. While males have shown an increasing trend. The regions experiencing the greatest growth in ASR were South Asia. The countries with the largest increases were Equatorial Guinea. However, It is worth noting that there may be variations among specific subtypes of leukemia. Regions with high Socio-demographic Index (SDI) have had the highest ASR, while low-middle SDI regions have shown the greatest increase in these rates. All ASRs values have been positively correlated with SDI, but there has been a turning point in medium to high SDI regions.

**Conclusions:**

Leukemia attributable to high BMI risk is gradually becoming a heavier burden globally. Different subtypes of leukemia have distinct temporal and regional patterns. This study’s findings will provide information for analyzing the worldwide disease burden patterns and serve as a basis for disease prevention, developing suitable strategies for the modifiable risk factor.

## 1 Introduction

Leukemia, a term based on the Greek expressions ‘leukos’ (representing white) and ‘haima’ (representing blood), denotes a group of malignant blood disorders (1). Modern leukemia is considered to be a malignant clonal tumor originating from hematopoietic stem cells. In clinical practice, leukemia is mainly classified based on the differentiation degree of leukemia cells and the duration of the disease’s natural course. The major classifications of leukemia include AML (acute myeloid leukemia), ALL (acute lymphoblastic leukemia), CML (chronic myeloid leukemia), CLL (chronic lymphocytic leukemia), and other types of leukemia (1). The severity and refractory nature of clinical symptoms in leukemia make it a significant threat to human health. BMI is widely utilized as a reflection of obesity, and current research suggests that it is a significant factor in the development of many types of cancer (2). A high BMI is directly linked to a heightened risk of diverse malignant tumors, including leukemia (3). Moreover, the global prevalence of obesity and the associated disease burden are increasing (4). Overweight and obesity have become increasingly serious global issues that impact people’s health. However, there is currently limited research specifically investigating the temporal trends of leukemia-related epidemiological indicators attributable to high BMI risk. As a result, we are unable to provide a comprehensive and accurate assessment of its severity. Therefore, conducting epidemiological research on leukemia attributable to high BMI risk is particularly important. This research can provide assistance in further maintaining people’s health.

Cancer stands as a major cause of global mortality and is recognized as a substantial public health concern. Existing research suggests that the geographic distribution of leukemia burden follows a pattern based on national development, with higher incidence and mortality rates often observed in highly developed regions of the world (5). The incidence of leukemia varies across different regions, and globally, the incidence of leukemia is declining. However, in developed regions, the incidence of leukemia is still increasing (6). Among the risk factors for leukemia, the age-standardized DALYs rate associated with high BMI is increasing (7). A multicenter prospective study has shown that overweight and obesity are associated with reduced responsiveness to induction therapy, decreased overall survival (OS), and disease-free survival (DFS) in young individuals who are newly diagnosed AML (8). In another study involving patients with ALL between the ages of 15 and 50 who were treated on Dana-Farber Cancer Institute (DFCI) consortium regimens, it was found that patients with higher BMI had poorer OS. Increased BMI was also associated with increased drug toxicity (9). Based on this, understanding the temporal trends and specific geographic patterns of leukemia attributable to high BMI risk becomes a crucial issue in order to better assist patients and formulate more precise prevention and treatment approaches.

The Global Burden of Disease (GBD) study, through close collaboration among governments around the world, provides comprehensive and standardized data to assess the impact of diseases, harm, and contributing factors. This project has facilitated a deeper understanding of global health conditions (10). One important benefit of this database is that it allows for a comprehensive and in-depth understanding of the epidemiological dynamics of leukemia attributable to high BMI risk. Within this research, we extracted data from the GBD collection on leukemia and its subtypes attributable to high BMI risk, considering various ages, genders, and regions. By identifying the temporal patterns of leukemia attributable to high BMI risk on a worldwide, local, and country-specific scale from 1990 to 2019, the disease burden associated with it was further evaluated. Our study findings serve as a significant extension to previous research on leukemia, providing valuable insights that can aid different countries in formulating preventive strategies for leukemia attributable to high BMI risk. This, in turn, can contribute to better patient care and support.

## 2 Methods

### 2.1 Data sources

Our research data is derived from the GBD study 2019, which includes a population health dataset assessing the disease prevalence and impact globally across 204 countries and territories (11). The study offers extensive calculations of disease occurrence, mortality, and prevalence in every country and territory. The collection, processing, and generation of this dataset in GBD Study 2019 have been thoroughly documented with comprehensive details (12, 13). The GBD utilizes all available information, including observational data, survey findings, literature, and medical patient information from hospitals. It generates predictions of mortality and incidence rates and provides a 95% confidence interval (95% UI) (6). The complete dataset of epidemiological studies on leukemia attributable to high BMI risk can be obtained online via the Global Health Data Exchange (GHDx) query tool at http://ghdx.healthdata.org/gbd-results-tool.

### 2.2 Data collection

The GBD 2019 study used the International Classification of Diseases (ICD) by the World Health Organization (WHO) to define leukemia as a cancer originating from hematopoietic stem cells. Based on this, we collected the following data (1): Worldwide information on leukemia attributable to high BMI, including the mortality figures, DALYs, and age-standardized rates (ASR) under various age cohorts, genders, socio-demographic indices (SDIs), nations and regions from 1990 to 2019. Specifically, we have obtained relevant information on leukemia and specific subtypes that can be attributable to high BMI risk. The disease types include leukemia, AML, ALL, CML, CLL, and other leukemia. (2) We have also obtained the SDIs for 21 geographical regions and 204 countries and territories during the years 1990 to 2019. The SDI is a combined measure of a country/region’s level of development. It is derived from various data points such as the fertility rate for women under 25, the mean educational attainment of women aged 15 and above, and income per person. The SDI is closely associated with human health outcomes and can be used for relevant analyses (14). According to the classification of the SDI for 204 countries and territories, five different regions have been defined: high, high-middle, middle, low-middle, and low. In order to track data at the regional level, the 204 countries and territories worldwide have been divided into 21 geographical regions according to their similar geographic locations (11, 15).

### 2.3 Definitions

The BMI is determined by weight and height and is used as a standard to measure the degree of obesity or leanness in a person’s body and assess their overall health (4). In adults (20 years and older), a high BMI is characterized by a BMI of 25 kg/m2 or above. The patients in this study were all collected from adults aged 20 years and above, as obtained from GBD2019 data. The Age-Standardized Rate (ASR) is applied to compare disease incidence and mortality rates, or other health indicators between different populations or regions. ASR includes Age-Standardized Incidence Rate (ASIR), Age-Standardized Death Rate (ASDR), and Age-Standardized DALY Rate. It standardizes disease incidence or mortality rates among different populations or regions to eliminate biases caused by differences in age structures. Disability-Adjusted Life Years (DALYs) represents the total years of healthy life lost due to disease, combining Years Lived with Disability (YLD) and Years of Life Lost (YLL) due to untimely mortality (11).

### 2.4 High BMI risk-factor-attributable leukemia burden estimation

In GBD 2019, the attributable risk factors were determined according to the standards of the World Cancer Research Fund, which include high BMI. The GBD comparative risk assessment framework is used to calculate the proportion of cancer-specific burden attributable to each risk factor, including leukemia and its various subtypes. This framework consists of six main steps, which are followed for each risk-outcome pair. The process involves identifying risk-outcome pairs meeting specific evidence criteria outlined by the World Cancer Research Fund. These pairs are then used to assess GBD risk factors. Following this, relative risk (RR) is estimated for each risk-outcome pair, and the exposure distribution of each risk factor is determined based on factors such as age, gender, location, and year. The Theoretical Minimum Risk Exposure Level (TMREL) is then calculated. Subsequently, using the RR, exposure levels, and TMREL, the Population Attributable Fraction (PAF) and attributable burden are estimated for each risk outcome. This information is used to model the PAF, which is then multiplied by the number of cancer deaths to determine the number of deaths attributable to that risk factor. Finally, the combined burden of multiple risk factors is estimated in the sixth step. Detailed methodologies for each step have been previously documented (14).

### 2.5 Statistical analysis

To quantify the trends in ASR, we calculated the Estimated Annual Percentage Change (EAPC) (16, 17). By taking the years as the independent variable (x) and the natural logarithm transformation of the ASR as the dependent variable (y), we fit a regression equation to the natural logarithm of the rate, i.e., y = α + βx, where x represents the year and y represents ln(ASR). Further calculations were performed to obtain the EAPC values using the formula EAPC = 100 * (e^β - 1). The 95% confidence interval (CI) for EAPC is a comprehensive measure of the trend in the Age-Standardized Rate (ASR) during a set period. If the minimum threshold is higher than 0, it indicates an increasing trend, while if the upper limit is below 0, it indicates a decreasing trend (16). Furthermore, we also analyzed the EAPC for leukemia and its subtypes attributable to high BMI risk and examined the correlation between EAPC and ASR, as well as ASR and SDI. Pearson correlation analysis was utilized to calculate the ρ coefficient and the p-value for correlation. The ρ coefficient ranges from -1 to 1 and is used to reflect the linear correlation between two variables. All tests were two-tailed, and a p-value less than 0.01 was considered statistically significant. We plotted the years of life lost (YLL) due to leukemia and its subtypes attributable to high BMI risk worldwide, stratified by country. The analysis included ASR, EAPC, and percent change in cases over the period 1990–2019. The analysis of data and generation of visualizations in this study were carried out using R software (version 4.3.2) and GraphPad Prism (version 9.5.0).

### 2.6 Patient and public involvement

Our study data is based on the GBD study, which includes data on disease burden worldwide (11). The data in the GBD database is based on aggregated statistical results at a population level and does not involve individual patient identification information. Therefore, in our study, we did not directly access or obtain specific individual patient data. Our research aims to provide relevant information and insights for global disease burden and public health policies by analyzing aggregated data from the GBD database, ultimately aiming to better assist patients.

## 3 Results

### 3.1 The global burden of leukemia attributable to high BMI risk

In 2019, the total number of deaths of leukemia was 0.33 million, and the corresponding DALYs totaled 116.6 million (18). Leukemia attributable to high BMI risk was a part of this Figure. **Table 1** presents the global number of deaths, DALYs, and their trends of leukemia attributable to high BMI risk. Globally, over the past 30 years, the number of deaths from leukemia attributable to high BMI risk has increased from 9.35 thousand in 1990 to 21.73 thousand in 2019. Simultaneously, the number of DALYs from leukemia attributable to high BMI risk has increased from 273.9 thousand in 1990 to 584.09 thousand in 2019. Despite the nearly two to three-fold increase since 1990, the global age-standardized death and DALYs rates have shown a relatively stable trend over the past 30 years. The global ASDR due to leukemia attributable to high BMI risk has changed from 0.24 (95% UI: 0.1 to 0.43) for every 100,000 population in 1990 to 0.27 (95% UI: 0.13 to 0.46) for every 100,000 population in 2019, with an EAPC of 0.34 (95% UI: 0.29 to 0.4). Similarly, the global age-standardized DALYs rates of leukemia attributable to high BMI risk has increased from 6.33 (95% UI: 2.65 to 11.55) for every 100,000 population in 1990 to 7.1 (95% UI: 3.49 to 12.09) for every 100,000 population in 2019, with an EAPC of 0.29 (95% UI: 0.24 to 0.34).

**Table 1:** The number and age-standardized rate of death and DALYs from leukemia attributable to high body-mass index risk in 1990 and 2019.

	Deaths (95% UI)				1990–2019	DALYs (95% UI)				1990–2019
	1990		2019			1990		2019		
	death cases No.×10^3^	ASR per 100000	death cases No.×10^3^	ASR per 100000	EAPC No. (95%CI)	DALYs No.×10^3^	ASR per 100000	DALYs No.×10^3^	ASR per 100000	EAPC No. (95%CI)
Global	9.35(3.93 to 16.82)	0.24(0.1 to 0.43)	21.73(10.51 to 37.03)	0.27(0.13 to 0.46)	0.34(0.29 to 0.4)	273.9(113.63 to 505.11)	6.33(2.65 to 11.55)	584.09(287.66 to 992.77)	7.1(3.49 to 12.09)	0.29(0.24 to 0.34)
Gender										
Female	5.75(2.06 to 11.25)	0.27(0.1 to 0.53)	12.5(4.77 to 22.91)	0.29(0.11 to 0.53)	0.11(0.05 to 0.17)	164.46(59.17 to 330.38)	7.31(2.63 to 14.65)	329.52(127.48 to 610.64)	7.73(2.99 to 14.33)	0.05(-0.02 to 0.11)
Male	3.6(1.45 to 6.78)	0.2(0.08 to 0.38)	9.24(4.3 to 15.89)	0.25(0.12 to 0.44)	0.71(0.66 to 0.77)	109.44(42.98 to 209.77)	5.29(2.1 to 10.02)	254.57(119.21 to 439.43)	6.47(3.01 to 11.16)	0.63(0.59 to 0.67)
Type of cause										
Acute myeloid leukemia	2.41(1.06 to 4.25)	0.06(0.03 to 0.11)	7.01(3.44 to 11.72)	0.09(0.04 to 0.15)	1.34(1.25 to 1.43)	71.16(31.15 to 126.41)	1.64(0.72 to 2.9)	186.48(93.01 to 307.99)	2.26(1.13 to 3.74)	1.17(1.11 to 1.23)
Acute lymphoid leukemia	0.79(0.33 to 1.46)	0.02(0.01 to 0.03)	2.01(0.94 to 3.42)	0.02(0.01 to 0.04)	1.08(1.02 to 1.15)	29.53(12.22 to 56.07)	0.63(0.26 to 1.18)	74.35(35.02 to 128.29)	0.9(0.43 to 1.56)	1.34(1.28 to 1.4)
Chronic myeloid leukemia	1.28(0.56 to 2.25)	0.03(0.01 to 0.06)	1.83(0.92 to 3.06)	0.02(0.01 to 0.04)	-1.74(-1.91 to -1.56)	38.32(16.58 to 69.27)	0.88(0.38 to 1.58)	54.5(27.49 to 90.64)	0.66(0.33 to 1.1)	-1.52(-1.7 to -1.34)
Chronic lymphoid leukemia	1.39(0.62 to 2.44)	0.04(0.02 to 0.07)	3.62(1.76 to 6.15)	0.05(0.02 to 0.08)	0.39(0.25 to 0.54)	31.17(13.93 to 54.87)	0.8(0.36 to 1.41)	76.95(37.84 to 129.81)	0.94(0.46 to 1.59)	0.42(0.3 to 0.54)
Other leukemia	3.47(1.37 to 6.59)	0.09(0.04 to 0.17)	7.27(3.48 to 12.66)	0.09(0.04 to 0.16)	-0.03(-0.1 to 0.04)	103.73(39.51 to 204.34)	2.38(0.92 to 4.62)	191.82(91.8 to 335.4)	2.33(1.12 to 4.08)	-0.23(-0.32 to -0.14)
Socio-demographic factor										
High SDI	3.99(1.77 to 6.92)	0.39(0.17 to 0.67)	7.66(3.81 to 12.53)	0.4(0.2 to 0.66)	0.07(0.02 to 0.13)	98.41(44.45 to 171.36)	9.89(4.47 to 17.25)	161.69(82.87 to 261.28)	9.91(5.15 to 15.95)	-0.12(-0.18 to -0.05)
High-middle SDI	3.11(1.36 to 5.48)	0.29(0.13 to 0.51)	6.06(2.92 to 10.19)	0.31(0.15 to 0.52)	0.05(-0.06 to 0.16)	92.71(40.4 to 165.63)	8.2(3.59 to 14.57)	159.2(77.09 to 265.3)	8.3(4.04 to 13.9)	-0.18(-0.27 to -0.08)
Middle SDI	1.6(0.57 to 3.22)	0.14(0.05 to 0.28)	5.28(2.55 to 9.12)	0.21(0.1 to 0.37)	1.48(1.43 to 1.53)	59.27(20.52 to 122.44)	4.41(1.55 to 8.95)	172.27(83 to 296.35)	6.54(3.14 to 11.26)	1.35(1.29 to 1.41)
Low-middle SDI	0.46(0.14 to 1.01)	0.07(0.02 to 0.16)	1.98(0.9 to 3.6)	0.14(0.06 to 0.25)	2.46(2.41 to 2.5)	16.53(4.92 to 37.11)	2.14(0.65 to 4.75)	65.2(30.16 to 118.77)	4.16(1.91 to 7.56)	2.36(2.32 to 2.39)
Low SDI	0.19(0.06 to 0.43)	0.07(0.02 to 0.17)	0.73(0.3 to 1.34)	0.13(0.05 to 0.23)	1.98(1.83 to 2.13)	6.82(1.98 to 15.43)	2.24(0.67 to 5.02)	25.33(10.67 to 47.11)	3.73(1.58 to 6.86)	1.9(1.75 to 2.05)
Region										
Andean Latin America	0.06(0.02 to 0.11)	0.25(0.1 to 0.46)	0.24(0.12 to 0.42)	0.42(0.21 to 0.73)	1.91(1.76 to 2.05)	2.16(0.89 to 3.97)	8.11(3.38 to 14.65)	7.86(3.83 to 13.64)	12.91(6.32 to 22.35)	1.72(1.56 to 1.87)
Australasia	0.1(0.05 to 0.17)	0.43(0.2 to 0.74)	0.24(0.12 to 0.38)	0.48(0.25 to 0.76)	0.2(0.13 to 0.26)	2.46(1.16 to 4.18)	10.68(5.05 to 18.17)	4.93(2.58 to 7.8)	11.12(5.88 to 17.44)	-0.03(-0.11 to 0.05)
Caribbean	0.07(0.03 to 0.12)	0.26(0.11 to 0.44)	0.17(0.08 to 0.28)	0.32(0.16 to 0.55)	0.97(0.89 to 1.05)	2.27(1 to 3.92)	7.75(3.44 to 13.31)	4.85(2.41 to 8.16)	9.51(4.73 to 16.03)	0.84(0.78 to 0.9)
Central Asia	0.14(0.06 to 0.24)	0.27(0.12 to 0.46)	0.24(0.12 to 0.4)	0.31(0.15 to 0.51)	0.59(0.47 to 0.71)	5.01(2.23 to 8.86)	9.06(4.1 to 15.88)	8.68(4.32 to 14.31)	9.72(4.86 to 15.98)	0.14(0.03 to 0.24)
Central Europe	0.62(0.3 to 1.02)	0.43(0.21 to 0.71)	1.08(0.57 to 1.75)	0.51(0.27 to 0.83)	0.64(0.54 to 0.74)	16.84(8.33 to 27.88)	11.58(5.72 to 19.25)	24.67(13.19 to 39.76)	12.85(6.85 to 20.7)	0.36(0.28 to 0.45)
Central Latin America	0.25(0.1 to 0.44)	0.25(0.11 to 0.44)	0.81(0.39 to 1.37)	0.33(0.16 to 0.57)	0.95(0.88 to 1.02)	9.43(4 to 16.8)	8.16(3.48 to 14.37)	27.31(13.45 to 46.46)	10.79(5.33 to 18.35)	0.93(0.87 to 0.99)
Central Sub-Saharan Africa	0.02(0.01 to 0.04)	0.09(0.03 to 0.17)	0.06(0.02 to 0.11)	0.1(0.04 to 0.19)	0.13(-0.21 to 0.48)	0.71(0.26 to 1.46)	2.43(0.89 to 4.81)	2.02(0.8 to 3.92)	2.8(1.11 to 5.4)	0.17(-0.17 to 0.51)
East Asia	0.87(0.17 to 2.19)	0.09(0.02 to 0.22)	2.63(0.95 to 5.22)	0.13(0.05 to 0.26)	1.52(1.42 to 1.63)	33.8(6.46 to 85.93)	2.97(0.58 to 7.48)	86.92(31.7 to 172.69)	4.44(1.59 to 8.81)	1.38(1.27 to 1.49)
Eastern Europe	1.03(0.49 to 1.7)	0.37(0.18 to 0.62)	1.33(0.67 to 2.14)	0.4(0.2 to 0.64)	-0.02(-0.19 to 0.14)	30.59(14.66 to 51.23)	11.19(5.35 to 18.86)	34.89(17.76 to 56.13)	11.15(5.68 to 17.93)	-0.45(-0.64 to -0.25)
Eastern Sub-Saharan Africa	0.06(0.02 to 0.15)	0.07(0.02 to 0.18)	0.24(0.1 to 0.46)	0.13(0.06 to 0.26)	2.26(2.02 to 2.49)	2.26(0.6 to 5.43)	2.24(0.6 to 5.38)	8.47(3.64 to 15.86)	3.81(1.66 to 7.18)	1.94(1.71 to 2.16)
High-income Asia Pacific	0.25(0.07 to 0.54)	0.12(0.04 to 0.27)	0.44(0.15 to 0.9)	0.1(0.04 to 0.21)	-0.75(-0.79 to -0.7)	7.71(2.18 to 16.9)	3.82(1.07 to 8.42)	9.52(3.28 to 19.16)	2.86(0.99 to 5.72)	-1.22(-1.3 to -1.15)
High-income North America	1.9(0.89 to 3.19)	0.54(0.26 to 0.9)	3.83(2.01 to 5.99)	0.61(0.32 to 0.95)	0.3(0.16 to 0.44)	47.15(22.61 to 77.69)	14.18(6.82 to 23.27)	81.11(43.84 to 125.57)	14.44(7.82 to 22.33)	-0.08(-0.23 to 0.06)
North Africa and Middle East	0.77(0.35 to 1.33)	0.41(0.19 to 0.71)	2.25(1.2 to 3.64)	0.5(0.27 to 0.81)	0.62(0.54 to 0.71)	26.5(11.87 to 46.44)	12.2(5.6 to 21)	72.45(38.84 to 118.32)	13.87(7.45 to 22.45)	0.38(0.3 to 0.45)
Oceania	0.01(0 to 0.01)	0.22(0.09 to 0.42)	0.02(0.01 to 0.04)	0.23(0.1 to 0.44)	-0.2(-0.39 to -0.02)	0.31(0.12 to 0.57)	7.16(2.85 to 13.25)	0.72(0.31 to 1.38)	7.18(3.07 to 13.55)	-0.29(-0.5 to -0.08)
South Asia	0.28(0.08 to 0.64)	0.05(0.01 to 0.11)	1.52(0.69 to 2.77)	0.1(0.05 to 0.19)	2.7(2.54 to 2.87)	9.9(2.91 to 22.78)	1.35(0.4 to 3.08)	48.96(22.24 to 90.06)	3.05(1.39 to 5.57)	2.73(2.6 to 2.87)
Southeast Asia	0.27(0.08 to 0.61)	0.09(0.03 to 0.21)	1.2(0.54 to 2.19)	0.19(0.08 to 0.35)	2.67(2.58 to 2.76)	10.1(2.94 to 23.07)	2.93(0.86 to 6.66)	40.18(18.26 to 72.71)	5.82(2.63 to 10.59)	2.45(2.32 to 2.57)
Southern Latin America	0.13(0.05 to 0.25)	0.29(0.12 to 0.54)	0.32(0.15 to 0.54)	0.39(0.18 to 0.65)	0.73(0.47 to 0.99)	3.85(1.55 to 7.1)	8.18(3.28 to 15.05)	8.2(3.85 to 13.71)	10.46(4.92 to 17.53)	0.61(0.37 to 0.85)
Southern Sub-Saharan Africa	0.08(0.04 to 0.13)	0.26(0.12 to 0.45)	0.19(0.1 to 0.3)	0.34(0.18 to 0.55)	1.13(0.9 to 1.35)	2.59(1.23 to 4.39)	7.5(3.56 to 12.75)	5.58(2.93 to 8.92)	8.69(4.58 to 13.89)	0.71(0.49 to 0.93)
Tropical Latin America	0.23(0.09 to 0.41)	0.23(0.09 to 0.42)	0.77(0.39 to 1.25)	0.32(0.16 to 0.52)	1.3(1.18 to 1.42)	7.78(3.22 to 14.14)	6.81(2.83 to 12.31)	21.96(11.21 to 35.43)	8.9(4.53 to 14.39)	1.01(0.91 to 1.11)
Western Europe	2.14(0.94 to 3.77)	0.38(0.17 to 0.66)	3.81(1.8 to 6.44)	0.4(0.19 to 0.68)	0.16(0.1 to 0.22)	49.73(22.28 to 86.52)	9.44(4.2 to 16.48)	73.03(35.36 to 123.51)	9.28(4.54 to 15.62)	-0.19(-0.27 to -0.11)
Western Sub-Saharan Africa	0.08(0.03 to 0.17)	0.09(0.03 to 0.18)	0.34(0.16 to 0.61)	0.16(0.08 to 0.29)	2.33(2.28 to 2.37)	2.73(0.98 to 5.59)	2.53(0.91 to 5.17)	11.77(5.5 to 20.84)	4.7(2.2 to 8.33)	2.22(2.17 to 2.27)

ASR age-standardized rate; DALY disability adjusted life-year;EAPC estimated annual percentage change; UI uncertainty interval; CI confidence interval;SDI social-demographic index.

**Figure 1:**
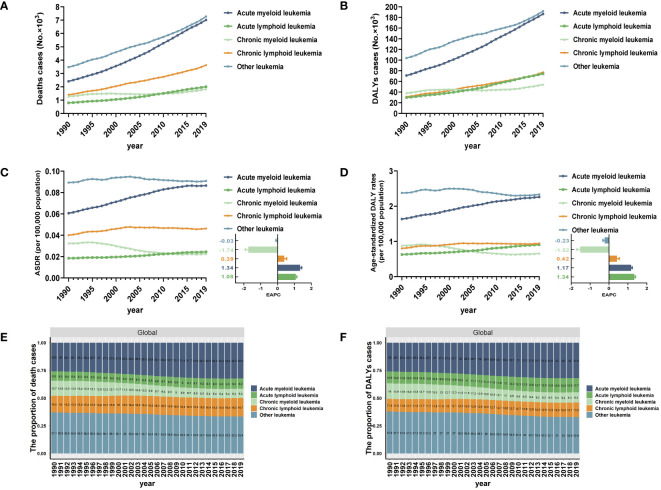
Global trends in death and DALYs for leukemia attributable to high BMI risk from 1990 to 2019. **(A)** The number of death cases for different subtypes of leukemia attributable to high BMI risk from 1990 to 2019. **(B)** The number of DALYs cases for different subtypes of leukemia attributable to high BMI risk from 1990 to 2019. **(C)** ASDR and EAPC in different subtypes of leukemia attributable to high BMI risk from 1990 to 2019. **(D)** Age-standardized DALYs rates and EAPC in different subtypes of leukemia attributable to high BMI risk from 1990 to 2019. **(E)** The proportion of death cases for different subtypes of leukemia attributable to high BMI risk from 1990 to 2019. **(F)** The proportion of DALYs cases for different subtypes of leukemia attributable to high BMI risk from 1990 to 2019.

For different subtypes of leukemia attributable to high BMI risk, in 2019, the number of death cases increased to 7.01 thousand for AML, 2.01 thousand for ALL, 1.83 thousand for CML, 3.62 thousand for CLL, and 7.27 thousand for other leukemia (**Table 1**, **Figure 1A**). The number of death cases has increased for all subtypes compared to previous years. In terms of ASDR based on the 2019 data, the ASDRs of AML, ALL, CML, CLL, and other leukemia attributable to high BMI were 0.09 (95% UI: 0.04 to 0.15), 0.02 (95% UI: 0.01 to 0.04), 0.02 (95% UI: 0.01 to 0.04), 0.05 (95% UI: 0.02 to 0.08), and 0.09 (95% UI: 0.04 to 0.16) for every 100,000 population, respectively. (**Table 1**, **Figure 1C**). Among them, only CML and other leukemia showed a declining trend in the corresponding EAPC (-1.74 and -0.03), while AML showed the most significant upward trend in EAPC at 1.34. The EAPC for ALL and CLL also showed upward trends at 1.08 and 0.39, respectively (**Table 1**, **Figure 1C**).In 2019, the numbers of DALYs of different subtypes of leukemia attributable to high BMI risk increased to 186.48 thousand for AML, 74.35 thousand for ALL, 54.5 thousand for CML, 76.95 thousand for CLL, and 191.82 thousand for other leukemia (**Table 1**, **Figure 1B**). All subtypes showed a significant increase compared to previous years. In terms of age-standardized DALYs rates, according to the 2019 data, the age-standardized DALYs rates of AML, ALL, CML, CLL, and other leukemia attributable to high BMI risk were 2.26 (95% UI: 1.13 to 3.74), 0.9 (95% UI: 0.43 to 1.56), 0.66 (95% UI: 0.33 to 1.1), 0.94 (95% UI: 0.46 to 1.59), and 2.33 (95% UI: 1.12 to 4.08) for every 100,000 population, respectively (**Table 1**, **Figure 1D**). Similar to ASDR, only CML and other leukemia showed a declining trend in the corresponding EAPC (-1.52 and -0.23), while ALL showed the most significant upward trend in EAPC at 1.34. AML and CLL also showed upward trends in EAPC at 1.18 and 0.42, respectively (**Table 1**, **Figure 1D**).

Additionally, we examined the overall changes in the distribution of deaths and DALYs of all specific subtypes of leukemia attributable to the risk. Among all leukemias attributable to the risk of high BMI, AML and other leukemia have consistently formed the highest proportion of death cases. However, between 1990 and 2019, the proportion of AML cases has been increasing (25.8% in 1990 to 32.2% in 2019), while the proportion of other leukemia cases has been decreasing (37.1% in 1990 to 33.4% in 2019). This trend may be attributed to the increasingly accurate diagnostic techniques that have allowed for more precise classification of different types of leukemia (**Figure 1E**). In terms of DALYs, the number and trend of change are similar to the death cases. AML and other leukemia continue to represent the highest proportion. The proportion of AML cases has been consistently increasing (**Figure 1F**).

### 3.2 The global burden of leukemia attributable to high BMI risk, divided by age and gender

**Figure 2:**
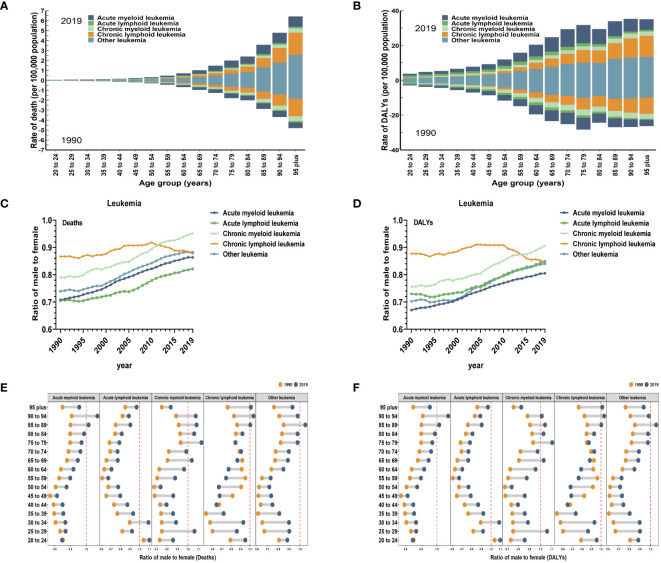
Global death and DALYs for leukemia attributable to high BMI risk by age and sex. **(A)** ASDR in different subtypes of leukemia attributable to high BMI risk by age for both sexes combined in 1990 and 2019. **(B)** Age-standardized DALYs rates in different subtypes of leukemia attributable to high BMI risk by age for both sexes combined in 1990 and 2019. **(C)** The sex ratio of different subtypes of leukemia attributable to high BMI risk death cases from 1990 to 2019. **(D)** The sex ratio of different subtypes of leukemia attributable to high BMI risk DALYs cases from 1990 to 2019. **(E)** The ratio of male to female in different subtypes of leukemia attributable to high BMI risk death cases by age in 1990 and 2019. **(F)** The ratio of male to female in different subtypes of leukemia attributable to high BMI risk DALYs cases by age in 1990 and 2019.

The age composition of the population can offer insights into primary prevention and indicate the health burden experienced by various age groups. The proportion of individuals aged 60 and above is extremely high in the population with leukemia attributable to high BMI risk. The ASDR account for over 90% of all age groups, and the age-standardized DALYs rates account for over 70% of all age groups (**Figures 2A, B**). In 2019, for different subtypes of leukemia, The ASDR of AML attributable to high BMI risk were highest in the 90 to 94 age group, with a rate of 1.04 for every 100,000 population. The corresponding ASDRs of ALL, CML, CLL, and other leukemia attributable to high BMI risk were all highest in the 95 plus age group, with rates of 0.16, 0.43, 2.23, and 2.55 for every 100,000 population, respectively (**Figure 2A**). In 2019, the age-standardized DALYs rates of AML, ALL, CML, CLL, and other leukemia attributable to high BMI risk were highest in the 75 to 79, 65 to 69, 90 to 94, 95 plus, and 95 plus age groups, respectively. The rates were 10.51, 2.10, 2.79, 12.00, and 13.51 for every 100,000 population, respectively (**Figure 2B**).

Globally, when considering gender, the ASR of leukemia attributable to high BMI risk are generally higher in females than males (**Table 1**). In 2019, the global ASDR of leukemia attributable to high BMI risk was 0.29 for every 100,000 females (95% UI: 0.11 to 0.53), which was higher than the rate of 0.25 for every 100,000 males (95% UI: 0.12 to 0.44). Similarly, the age-standardized DALYs rate of leukemia attributable to high BMI risk was 7.73 for every 100,000 females (95% UI: 2.99 to 14.33), higher than the rate of 6.47 for every 100,000 males (95% UI: 3.01 to 11.16) (**Table 1**). From 1990 to 2019, for specific subtypes of leukemia, the number of deaths and DALYs of AML, ALL, CML, CLL, and other leukemias attributable to high BMI risk globally were typically greater among females compared to males. (**Supplementary Figures S1**, **S2**). Globally, the male-to-female ratios of age-standardized death and DALYs rates for various subtypes of leukemia attributable to high BMI risk were all less than 1 (**Figures 2C, D**), indicating a higher proportion of females. However, except for CLL, where the male-to-female ratio remained stable, the male-to-female ratios for AML, ALL, CML, and other leukemia continued to increase (**Figures 2C, D**), suggesting a gradual increase in the proportion of males. This may be related to differences in levels of male and female hormones. It may even include inherent differences in the distribution of fat and muscle proportions between males and females. Further research is needed to confirm these possibilities.

By looking at the age-specific and gender-specific contributions to leukemia attributable to high BMI risk, it is not true that females outnumber males in all age groups. In the 84 to 94 age group for AML, the 30 to 34 age group for ALL, the 25 to 29 and 75 to 94 age groups for CML, the over 85 age group for CLL, and the 85 to 89 age group for other leukemia, the proportion of males gradually exceeds that of females (**Figures 2E, F**).

### 3.3 The burden of leukemia attributable to high BMI risk varies across different regions and countries

**Figure 3:**
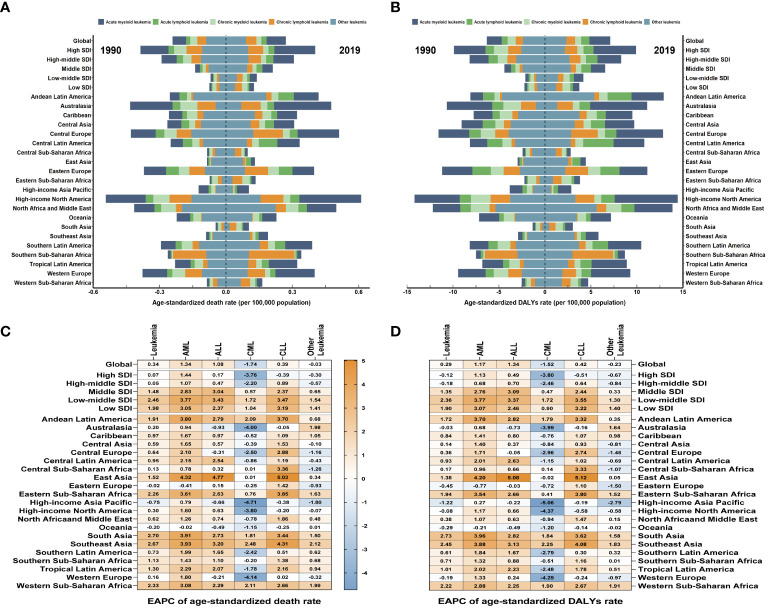
The global trends in leukemia attributable to high BMI risk by regions. **(A)** The ASDR of leukemia attributable to high BMI risk at a regional level in 1990 and 2019. **(B)** The age-standardized DALYs rates of leukemia attributable to high BMI risk at a regional level in 1990 and 2019. **(C)** The EAPC in ASDR of leukemia attributable to high BMI risk from 1990 to 2019, by subtypes and by regions. **(D)** The EAPC in age-standardized DALYs rates of leukemia attributable to high BMI risk from 1990 to 2019, by subtypes and by regions. Blue indicates a downward trend and Red indicates an upward trend.

**Figure 4:**
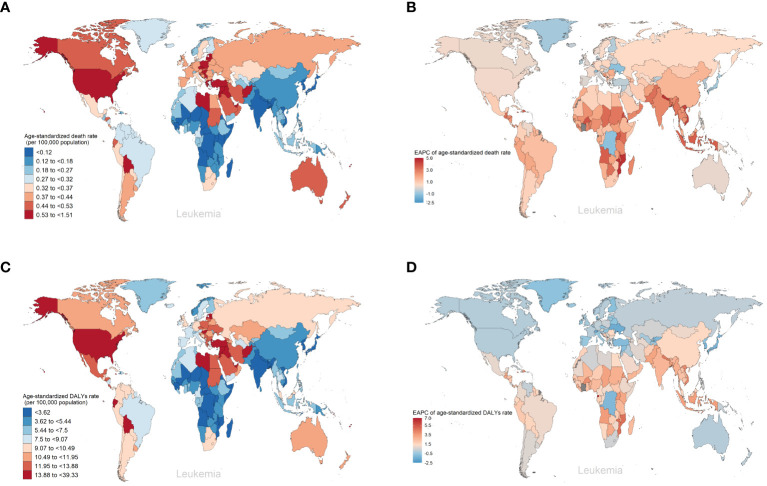
The global trends of leukemia attributable to high BMI risk by countries and territories. **(A)** The ASDR of leukemia attributable to high BMI risk in 2019. **(B)** The EAPC in ASDR of leukemia attributable to high BMI risk from 1990 to 2019. **(C)** The age-standardized DALYs rates of leukemia attributable to high BMI risk in 2019. **(D)** The EAPC in age-standardized DALYs rates of leukemia attributable to high BMI risk from 1990 to 2019.

As for the five SDI regions, the number of deaths from leukemia attributable to high BMI risk has increased. From 1990 to 2019, the ASDRs of leukemia attributable to high BMI risk have increased in all five SDI regions (**Table 1**, **Figure 3A**, **Supplementary Figure S3A**). The High SDI regions had the highest ASDR in 2019 (**Figure 3A**, **Supplementary Figure S3A**), but over the past 30 years, the ASDR in the High SDI regions has continued relatively stable. On the other hand, the Low-Middle SDI regions experienced the largest increase in ASDR, with an EAPC of 2.46 (95% UI: 2.41 to 2.5) (**Table 1**, **Figure 3C**). From a geographical perspective, in 2019, the highest ASDR of leukemia attributed to high BMI risk occurred in High-income North America (ASDR: 0.61 for every 100,000 population), Central Europe (ASDR: 0.51 for every 100,000 population), and North Africa and the Middle East (ASDR: 0.5 for every 100,000 population). The regions with the lowest ASDR were Central Sub-Saharan Africa (ASDR: 0.1 for every 100,000 population), High-income Asia Pacific (ASDR: 0.1 for every 100,000 population), and South Asia (ASDR: 0.1 for every 100,000 population) (**Table 1**, **Figure 3A**). The regions with the highest increase in ASDR were South Asia (EAPC: 2.7), Southeast Asia (EAPC: 2.67), and Western Sub-Saharan Africa (EAPC: 2.33). In contrast, the regions with the largest decrease in ASDR were High-income Asia Pacific (EAPC: -0.75), Oceania (EAPC: -0.2), and Eastern Europe (EAPC: -0.02) (**Table 1**, **Figure 3C**). Among the 204 countries and territories, Equatorial Guinea experienced the most significant increase in the number of deaths from leukemia attributed to high BMI risk (percentage change: 900% to 1200%) (**Supplementary Figure S5A**, **Supplementary Table S1**). In 2019, the ASDR of leukemia attributable to high BMI risk was observed to be highest in the Syrian Arab Republic, with rates of 1.51 for every 100,000 population (**Figure 4A**, **Supplementary Table S1**). On the other hand, Somalia had the lowest ASDR in 2019, with rates of 0.04 for every 100,000 population (**Supplementary Figure S4A**, **Supplementary Table S1**). In addition, the countries with the highest increase in ASDR were Equatorial Guinea (EAPC = 7.26) (**Figure 4B**, **Supplementary Table S1**).

Over the past thirty years, the number of DALYs from leukemia attributable to high BMI risk has increased. The age-standardized DALYs rate in the five SDI regions remains highest in the High SDI region (**Table 1**, **Figure 3B**, **Supplementary Figure S4A**). The High SDI region and High-middle SDI region have shown relatively stable changes, while the age-standardized DALYs rates have increased in the remaining regions (**Supplementary Figure S4A**). Among them, the Low-middle SDI region has experienced the largest upward trend in age-standardized DALYs rates, with an EAPC of 2.36 (95% UI: 2.32 to 2.39) (**Table 1**, **Figure 3D**). From a geographical perspective, in 2019, the highest age-standardized DALYs rates were observed in High-income North America (14.44 for every 100,000 population), North Africa and the Middle East (13.87 for every 100,000 population), and Andean Latin America (12.91 for every 100,000 population). The regions with the lowest age-standardized DALYs rates were Central Sub-Saharan Africa (2.8 for every 100,000 population), High-income Asia Pacific (2.86 for every 100,000 population), and South Asia (3.05 for every 100,000 population) (**Table 1**, **Figure 3B**). The regions with the highest increase in age-standardized DALYs rates were South Asia (EAPC = 2.73), Southeast Asia (EAPC = 2.45), and Western Sub-Saharan Africa (EAPC = 2.22). In contrast, the regions with the greatest reduction were High-income Asia Pacific (EAPC = -1.22), Eastern Europe (EAPC = -0.45), and Oceania (EAPC = -0.29) (**Table 1**, **Figure 3D**). Among the 204 countries and territories, Equatorial Guinea showed the most significant increase in the number of DALYs cases attributable to high BMI (percentage change: 900% to 1200%) (**Supplementary Figure S6A**, **Supplementary Table S1**). In 2019, consistent with ASDR, the Syrian Arab Republic had the highest age-standardized DALYs rates, with 39.33 for every 100,000 population (**Figure 4A**, **Supplementary Table S1**). The lowest age-standardized DALYs rates were observed in Somalia, with 1.26 for every 100,000 population (**Figure 4C**, **Supplementary Table S1**). Furthermore, Equatorial Guinea (EAPC = 6.84) had the highest increases in age-standardized DALYs rates (**Figure 4D**, **Supplementary Table S1**).

### 3.4 The distribution of different subtypes of leukemia attributable to high BMI risk varies across different regions and countries

Among the subtypes of leukemia attributable to high BMI risk, AML, CML, and CLL consistently have the highest ASDR and age-standardized DALYs rates in high SDI regions (**Supplementary Figures S3B-F**, **S4B-F**). ALL and other leukemia have had the highest ASDR and age-standardized DALYs rates in high-middle SDI regions for most of the time (**Supplementary Figures S3**, **S4**). However, for AML, ALL, CML, and CLL, over the past 30 years, the largest increasing trends have occurred in low-middle SDI regions, with EAPCs of 3.77, 3.43,1.72, and 3.47 for ASDR, and 3.77, 3.37,1.72, and 3.55 for age-standardized DALYs rates respectively (**Figures 3C, D**). For other leukemia attributed to high BMI risk, the largest increasing trend in ASDR still occurs in low-middle SDI regions with an EAPC of 1.54, while the largest increasing trend in age-standardized DALYs rates occurs in low SDI regions with an EAPC of 1.40 (**Figures 3C, D**). It is worth noting that CML maintains a low level of ASDR and age-standardized DALYs rates in all five SDI regions, which may be due to the widespread use of tyrosine kinase inhibitors, making CML a disease with relatively good treatment outcomes in all regions (**Supplementary Table S2**, **Supplementary Figures S3B-F**, **S4B-F**). From a geographical perspective, in 2019, AML had the highest ASDR and age-standardized DALYs rates in High-income North America (ASDR: 0.28 for every 100,000 population; age-standardized DALYs rates: 6.84 for every 100,000 population). ALL had the highest ASDR and age-standardized DALYs rates in Central Latin America (ASDR: 0.08 for every 100,000 population; age-standardized DALYs rates: 3.29 for every 100,000 population). CML and other leukemia had the highest ASDR and age-standardized DALYs rates in North Africa and the Middle East (ASDR: 0.06 and 0.22 for every 100,000 population, respectively; age-standardized DALYs rates: 1.65 and 5.63 for every 100,000 population, respectively). CLL had the highest ASDR and age-standardized DALYs rates in Southern Sub-Saharan Africa (ASDR: 0.21 for every 100,000 population; age-standardized DALYs rates: 4.43 for every 100,000 population) (**Supplementary Table S2**, **Figures 3A, B**); The regions with the highest increases in ASDR and age-standardized DALYs rates for AML, ALL, and CLL are East Asia (ASDR: EAPC 4.32, 4.77, 5.03; age-standardized DALYs rates: EAPC 4.2, 5.08, 5.12). For CML and other leukemia, the region with the highest increase in ASDR is Southeast Asia (EAPC 2.48, 2.12), while for CML, the region with the highest increase in age-standardized DALYs rates is Southeast Asia (EAPC 2.25), and for other leukemia, it is Western Sub-Saharan Africa (EAPC 1.91) (**Supplementary Table S2**, **Figures 3C, D**). In contrast, Eastern Europe had the largest decrease in ASDR and age-standardized DALYs rates for AML (ASDR: EAPC = -0.41; age-standardized DALYs rates: EAPC = -0.77). Australasia had the largest decrease in ASDR and age-standardized DALYs rates for ALL (ASDR: EAPC = -0.93; age-standardized DALYs rates: EAPC = -0.73). High-income Asia Pacific had the largest decrease in ASDR for CML, CLL, and other leukemia (EAPC: -4.71, -0.38, -1.8, respectively). High-income Asia Pacific also had the largest decrease in age-standardized DALYs rates for CML and other leukemia (EAPC: -5.06, -2.79), while High-income North America had the largest decrease in age-standardized DALYs rates for CLL (EAPC = -0.58) (**Supplementary Table S2**, **Figures 3C, D**).

**Figure 5:**
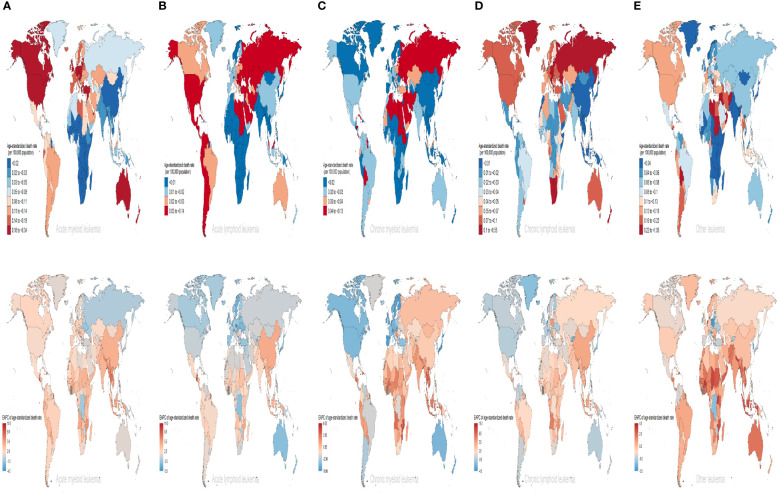
The ASDRs of different subtypes of leukemia attributable to high BMI risk in 2019 and the corresponding EAPC from 1990 to 2019. **(A)** AML **(B)** ALL **(C)** CML **(D)** CLL **(E)** other leukemia. AML acute myeloid leukemia, ALL acute lymphoblastic leukemia, CML chronic myeloid leukemia, CLL chronic lymphocytic leukemia.

Among the 204 countries and territories, Equatorial Guinea had the most significant increase in the number of deaths and DALYs form AML attributable to high BMI risk (deaths: ~2800% increase; DALYs: ~3300% increase). Guatemala had the most significant increase in the number of deaths and DALYs form ALL attributable to high BMI risk (deaths: ~3100% increase; DALYs: ~3200% increase). Equatorial Guinea had the most significant increases in the number of deaths and DALYs from CML and CLL attributable to high BMI risk (deaths: ~1000%, 3200% increase; DALYs: ~1100%, 3000% increase). Djibouti had the most significant increase in the number of deaths from other leukemia attributable to high BMI risk (~880% increase). United Arab Emirates had the most significant increase in the number of DALYs from other leukemia attributable to high BMI risk (~800% increase) (**Supplementary Table S1**, **Supplementary Figures S5B-F**, **S6B-F**).In 2019, the ASDR for AML attributable to high BMI risk was highest in Monaco (0.34 for every 100,000 population), while the age-standardized DALYs rate was highest in Fiji (9.35 for every 100,000 population). For ALL, both the ASDR and age-standardized DALYs rate were highest in San Marino (ASDR: 0.14 for every 100,000 population; age-standardized DALYs rate: 5.16 for every 100,000 population). The ASDR for CML and CLL were both highest in Qatar (0.13 and 0.55 for every 100,000 population respectively), while the age-standardized DALYs rate for CML was highest in the United Arab Emirates and the Syrian Arab Republic (both at 3.3 for every 100,000 population), and the age-standardized DALYs rate for CLL was highest in Qatar (8.54 for every 100,000 population). For other leukemia, both the ASDR and age-standardized DALYs rate were highest in the Syrian Arab Republic (ASDR: 1.05 for every 100,000 population; age-standardized DALYs rate: 25.59 for every 100,000 population) (**Supplementary Table S1**, **Figures 5A-E**, **Supplementary Figures S7A-E**). Furthermore, the largest increases in ASDR and age-standardized DALYs rate for AML, CML, CLL, and other leukemia attributable to high BMI were observed in Equatorial Guinea (ASDR: EAPC 10.27, 6.52, 11.9, 4.12; age-standardized DALYs rate: EAPC 10.03, 6.31, 11.4, 3.95). The largest increases in ASDR and age-standardized DALYs rate for ALL attributable to high BMI were observed in Guatemala (ASDR: EAPC 10.78; age-standardized DALYs rate: EAPC 10.08) (**Supplementary Table S1**, **Figures 5A-E**, **Supplementary Figures S7A-E**).

### 3.5 The correlation between SDI and the burden of leukemia attributable to high BMI risk

**Figure 6:**
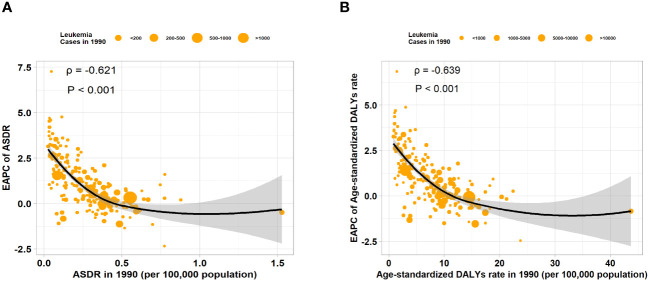
The correlation between EAPC of ASR and ASR of 1990 in 204 countries or territories. **(A)** The correlation between the EAPC of ASDR and ASDR of 1990 in 204 countries or territories. **(B)** The correlation between the EAPC of age-standardized DALYs rate and age-standardized DALYs rate of 1990 in 204 countries or territories.

**Figure 7:**
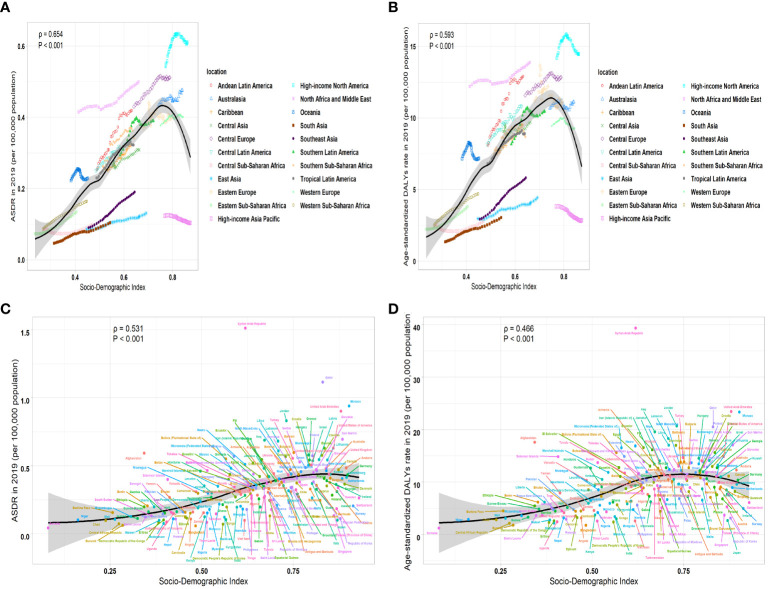
The correlation between ASR of 2019 and SDI from 1990 to 2019 in leukemia attributable to high BMI risk. **(A)** The correlation between ASDR of 2019 and SDI from 1990 to 2019 in leukemia attributable to high BMI risk in 21 regions. **(B)** The correlation between age-standardized DALYs rate and SDI from 1990 to 2019 in leukemia attributable to high BMI risk in 21 regions. **(C)** The correlation between ASDR and SDI from 1990 to 2019 in leukemia attributable to high BMI risk in 204 countries and territories. **(D)** The correlation between age-standardized DALYs rate and SDI from 1990 to 2019 in leukemia attributable to high BMI risk in 204 countries and territories.

We calculated the relationship between the correlation between EAPC of ASR and the ASR of leukemia attributable to high BMI risk in 1990. We found a significant negative correlation between the EAPC of ASR and the ASR (ASDR: correlation coefficient = -0.621, P < 0.001; age-standardized DALYs rate: correlation coefficient = -0.639, P < 0.001) (**Figures 6A, B**). Similar results were observed in various subtypes of leukemia attributable to high BMI risk, where the EAPC showed a significant negative correlation with the corresponding ASDR and age-standardized DALYs rate (**Supplementary Figures S8A-E**). This result suggests that the number of leukemia attributable to high BMI risk in countries and regions with potentially lower ASDR and age-standardized DALYs rates may be underestimated. Then, we examined the relationship between the SDI from 1990 to 2019 and the ASR of 2019 in 21 regions and 204 countries and territories. Among the 21 regions, all ASRs values were significantly positively correlated with the SDI (correlation coefficient ASDR=0.654, age-standardized DALYs rate=0.593, all P-values < 0.001). This suggests that regions with higher SDI are more likely to have a heavier disease burden (**Figures 7A, B**). It is worth noting that, as shown in the Figure, there is a turning point in regions with medium to high SDI, where both the ASDR and age-standardized DALYs rates start to decline significantly. This could be due to increased investments in healthcare and heightened health awareness among the population in regions with higher SDI. At the level of 204 countries and territories, the ASRs values were also positively correlated with SDI (correlation coefficient: ASDR=0.531, age-standardized DALYs rate=0.466, all P-values < 0.001) (**Figures 7C, D**). Similar results were observed in various subtypes of leukemia attributable to high BMI risk. In all 21 regions, the ASRs values were significantly positively correlated with SDI (**Supplementary Figures S9A-E**). Among the subtypes, AML showed the strongest correlation in both ASDR (correlation coefficient: 0.723) and age-standardized DALYs rate (correlation coefficient: 0.706), with all P-values < 0.001 (**Supplementary Figure S9A**); At the level of 204 countries and territories, in various subtypes of leukemia attributable to high BMI risk, the ASRs values still showed a positive correlation with SDI(**Supplementary Figures S10A-E**). Among these subtypes, AML remained the strongest correlate (correlation coefficient: ASDR=0.719, age-standardized DALYs rate=0.639), with all P-values < 0.001 (**Supplementary Figure S10A**). This suggests that AML attributable to high BMI risk has a closer relationship with SDI compared to all other subtypes, emphasizing the need for us to pay more attention to it.

## 4 Discussion

This report’s research findings comprehensively and systematically assess the burden of leukemia attributable to high BMI risk during the years 1990 to 2019, categorized by regions and countries. High BMI, as an established risk factor contributing to leukemia, increases the risk of mortality among leukemia patients (19). This study fills an epidemiological gap by analyzing the temporal patterns of leukemia attributable to high BMI risk over a span of more than 30 years. By tracking these trends, we gain valuable insights into the changing patterns of leukemia attributable to high BMI risk over time. This study enables comparability of leukemia attributable to high BMI risk among various regions, countries, genders, timelines, and subgroups. By establishing a standardized methodology, we can effectively compare the burden of leukemia attributable to high BMI risk and identify variations and patterns across various factors. Since 1990, the statistic of deaths and DALYs from leukemia attributable to high BMI risk has been increasing globally. However, the ASRs have remained relatively stable over the past 30 years, with an EAPC of 0.34 for ASDR and 0.29 for age-standardized DALYs rate. However, what needs our attention is that among the four classifiable subtypes of leukemia, only the ASDR and age-standardized DALYs rate for CML show a declining trend, with an EAPC of -1.74 for ASDR and -1.52 for age-standardized DALYs rate. On the other hand, AML, ALL, and CLL exhibited a rising EAPC trend, suggesting that CML plays a predominantly negative role in the overall ASDR increase for the disease. This may be attributed to the introduction of tyrosine kinase inhibitors (TKIs), which have greatly improved the clinical outcomes and fundamentally changed the treatment patterns and prognosis for CML patients (20, 21). However, the corresponding EAPC for AML shows the most notable rise in ASDR, and the corresponding EAPC for ALL shows the sharpest increase in age-standardized DALYs rates. Although significant progress has been made in providing increasingly targeted and personalized treatment approaches for AML through the integration of cytogenetics, molecular biology, and clinical data (22–24), in this study, AML still remains the most burdensome type among the four classifiable types. It suggests that the acute leukemia attributable to high BMI risk is a serious problem we face.

At the age level, it is widely recognized that aging has an impact on the occurrence of tumors. Aging cells exhibit changes such as genomic genetic instability, impaired mitochondrial function, changes in epigenetic patterns, telomere erosion, stem cell depletion, and intercellular communication (25). Aging leads to dysregulation of hematopoietic stem cells’ hematopoietic function, resulting in a significant increase in the occurrence of age-related leukemia (26, 27). Our research also suggests that leukemia attributable to high BMI risk has significantly higher ASR among the elderly population (**Figures 2A, B**). This indicates that the risks faced by older individuals are much higher than those faced by younger individuals. Another important factor to consider, of course, is the potential for additional comorbidities (such as metabolic syndrome) associated with aging and high BMI, which play a role in morbidity and mortality. From a gender perspective, in studies involving all types of leukemia not related to BMI, the majority of cases were found in males (28). However, in contrast, in this study, the proportion of females affected by leukemia and its subtypes attributable to high BMI risk was higher than males during the years 1990 to 2019. The male-female ratio of ASDR and age-standardized DALYs rates are both less than 1, indicating that females are more likely to be affected by leukemia attributable to high BMI risk compared to males. This could possibly be due to disparities in hormone levels between males and females, as well as differences in the inherent distribution of fat and muscle between males and females (29, 30). Further research is needed to confirm this. However, it is important to note that in various subtypes, the male-to-female ratio of AML, ALL, CML, and other leukemia has been steadily increasing over the years, indicating a trend of increasing proportion of males. Additionally, this ratio varies with age, and it does not mean that females outnumber males in all age groups (**Figures 2C, D**). These trends can provide some supporting evidence for the development of prevention and treatment strategies tailored to different age and gender populations.

The burden and patterns of leukemia linked to high BMI risk vary by region. Throughout the past three decades, the High SDI regions have consistently had the greatest ASR, and these rates have remained stable. However, the Low-Middle SDI regions have experienced the greatest increase in ASR, with an EAPC of 2.46 for ASDR and 2.36 for age-standardized DALYs rates. This is likely primarily due to socio-economic disparities, inadequate healthcare funding, and insufficient health consciousness, which aggravate the burden of disease in lower SDI regions. This can be ameliorated through global cooperation and mutual assistance relationships (31). In terms of regional analysis, High-income North America has the highest ASR. However, South Asia has experienced the largest increase. At the national level, the Syrian Arab Republic has been observed to have the highest ASDR. However, Equatorial Guinea has shown the largest increase in ASDR. This may suggest that developing countries may face an increasing burden of leukemia attributable to high BMI risk diseases. Low-income regions and countries are experiencing an increasing burden of leukemia attributed to the risk of high BMI. Population changes and shifting risk factors are causing developing countries to face a growing disease burden (32). Developing countries lack relatively sufficient medical resources, and the risk awareness of the people is relatively weak. There is a lack of preventive measures and the treatment ratio is low (33, 34). Even the political situation of a country can have an impact on the health burden of its people. Developing countries and low-income regions need more attention in subsequent efforts to eliminate the leukemia linked to a high BMI risk.

Among the different subtypes of leukemia attributable to high BMI risk, the ASRs for AML and ALL show a particularly pronounced upward trend in most countries, while CML exhibits a significant decline in the majority of countries (**Supplementary Table S2**, **Figures 5A-E**, **Figures 6A-E**). There may be several reasons for this observation. Advances in diagnostic techniques may have led to more patients receiving precise diagnoses. The diagnosis of hematological diseases heavily relies on laboratory examinations. For acute leukemia, the internationally recognized diagnostic classification utilizes morphological, immunological, cytogenetic, and molecular biology classifications. Over the past 30 years, advancements in lab technology have had a substantial effect on the diagnosis and treatment of leukemia (35–37). Specific gene mutations and biomarkers used for classifying and predicting leukemia have made significant contributions to the precision of diagnosis (38). Changes in diagnosis and definition have a certain impact on the burden of disease, such as the requirement of having more than 20% leukemic primitive cells within the bone marrow for the diagnosis of AML. However, when specific clonal cytogenetic abnormalities are present, such as t(8;21)(q22;q22), inv(16)(p13q22), t(16;16)(p13;q22), or t(15;17)(q22;q12), even if the percentage of leukemic precursor cells is less than 20%, diagnosing AML should still be made (39, 40). Currently, there has been significant progress in understanding AML, including new insights into the molecular pathogenesis of AML, resulting in updates in disease classification, and this is a field in evolution (41). On the other hand, in terms of treatment, the management of CML has been completely transformed over the past 30 years with the development of tyrosine kinase inhibitors (TKIs), significantly extending the lifespan of CML patients (42). These changes in various factors can potentially have an impact on the alteration of disease burden.

In analyzing the correlation between SDI and the burden of leukemia attributable to high BMI risk, we found a notable positive link between ASR and SDI. This indicates that regions with higher SDI are more likely to have a higher burden of disease. However, it is important to note that there is a turning point in regions with medium to high SDI, where ASRs begin to show a significant decline. This suggests that the significant decline in mortality rates may be due to higher investments in healthcare in regions with higher SDI or stronger awareness and prevention efforts regarding leukemia attributable to high BMI risk. SDI serves as a metric for measuring the level of national socioeconomic development. In economically advanced countries and regions, there is greater investment in healthcare resources (43) and more preventive strategies, contributing to a decrease in disease burden.

The uniqueness of our study lies in the comprehensive analysis of the global epidemiological trends of leukemia attributable to high BMI risk during the previous three decades. However, there are also some limitations. Similar to other Global Burden of Disease (GBD) studies, there are limitations due to differences in the comparability, accuracy, and quality of data from various regions and countries. This is particularly true for data from low-income countries, where data may be missing, which can lead to statistical biases in the results (44, 45). Due to the reliance on costly laboratory tests for the diagnosis of leukemia, economically developed countries often have more accurate diagnostic and reporting systems. In contrast, in regions with underdeveloped clinical hematology services, there is inevitably a risk of underdiagnosis and underreporting, leading to certain discrepancies in the registration process. Despite some constraints, our study represents the primary thorough assessment of the global trends in leukemia attributable to high BMI risk across the worldwide, regional, and national scopes in the last thirty years. It may have the potential to make a positive impact on the advancement of subsequent prevention strategies, research efforts, and resource optimization. This study is beneficial for providing health education, implementing early intervention, and promoting healthy lifestyles for patients. Regular BMI monitoring is conducted to initiate early intervention to mitigate the harm of the disease on people and alleviate societal burden. Through these efforts, our objective is to significantly impact the epidemiological situation, ultimately benefiting people.

## 5 Conclusions

In conclusion, leukemia attributable to high BMI risk remains a significant global public health issue. Although the ASRs have not shown a significant increase worldwide, there is a greater upward trend in developing countries. Among the classifiable subtypes of leukemia attributable to high BMI risk, only the CML subtype shows a declining trend. However, AML, ALL, and CLL all show an increasing trend, with AML being the highest burden among all classifiable types. Older individuals face a significantly higher risk compared to younger people. The burden is generally higher in females, but the rate among males is showing a gradual upward trend. Furthermore, during the previous three decades, regions with high SDI have shown higher ASRs. However, these rates have remained stable in high SDI regions. Conversely, low-middle SDI regions have experienced the largest upward trend, with the disease burden increasing year by year. All age-standardized rates (ASR) values are significantly positively correlated with SDI, but a turning point occurs in regions with medium to high SDI. In these areas, ASRs start to show a significant decline. The disparities in healthcare resources, demographics, social and economic factors, and lifestyle among different regions and countries have contributed to these differences. Our study timely analyzes and assesses global load and time-based patterns of leukemia attributable to high BMI risk, providing valuable information for formulating appropriate policies to prevent the disease and assist people.

## Data availability statement

The original contributions presented in the study are included in the article/**Supplementary Material**. Further inquiries can be directed to the corresponding author/s.

## Author contributions

HX: Data curation, Investigation, Visualization, Writing – original draft. XH: Investigation, Writing – original draft. PL: Writing – original draft. JD: Supervision, Writing – review & editing.

## Supplementary material

Supplementary Figure S1 The number of deaths cases in different subtypes of leukemia attributable to high BMI risk by different sex. A AML B ALL C CML D CLL E other leukemia. AML acute myeloid leukemia, ALL acute lymphoblastic leukemia, CML chronic myeloid leukemia, CLL chronic lymphocytic leukemia.

Supplementary Figure S2 The number of DALYs cases in different subtypes of leukemia attributable to high BMI risk by different sex. A AML B ALL C CML D CLL E other leukemia. AML acute myeloid leukemia, ALL acute lymphoblastic leukemia, CML chronic myeloid leukemia, CLL chronic lymphocytic leukemia.

Supplementary Figure S3 The ASDRs of different subtypes of leukemia attributable to high BMI risk in global and five SDI regions from 1990 to 2019. A all leukemia B AML C ALL D CML E CLL F other leukemia. AML acute myeloid leukemia, ALL acute lymphoblastic leukemia, CML chronic myeloid leukemia, CLL chronic lymphocytic leukemia.

Supplementary Figure S4 The age-standardized DALYs rates of different subtypes of leukemia attributable to high BMI risk in global and five SDI regions from 1990 to 2019. A all leukemia B AML C ALL D CML E CLL F other leukemia. AML acute myeloid leukemia, ALL acute lymphoblastic leukemia, CML chronic myeloid leukemia, CLL chronic lymphocytic leukemia.

Supplementary Figure S5 The percentage change of the number of deaths from different subtypes of leukemia attributable to high BMI risk in 1990 and 2019. A leukemia B AML C ALL D CML E CLL F other leukemia. AML acute myeloid leukemia, ALL acute lymphoblastic leukemia, CML chronic myeloid leukemia, CLL chronic lymphocytic leukemia.

Supplementary Figure S6 The percentage change of the number of DALYs from different subtypes of leukemia attributable to high BMI risk in 1990 and 2019. A leukemia B AML C ALL D CML E CLL F other leukemia. AML acute myeloid leukemia, ALL acute lymphoblastic leukemia, CML chronic myeloid leukemia, CLL chronic lymphocytic leukemia.

Supplementary Figure S7 The age-standardized DALYs rates of different subtypes of leukemia attributable to high BMI risk in 2019 and the corresponding EAPC from 1990 to 2019. A AML B ALL C CML D CLL E other leukemia. AML acute myeloid leukemia, ALL acute lymphoblastic leukemia, CML chronic myeloid leukemia, CLL chronic lymphocytic leukemia.

Supplementary Figure S8 The correlation between EAPC of ASR and ASR of 1990 for different subtypes of leukemia attributable to high BMI risk in 204 countries or territories. A AML B ALL C CML D CLL E other leukemia. AML acute myeloid leukemia, ALL acute lymphoblastic leukemia, CML chronic myeloid leukemia, CLL chronic lymphocytic leukemia.

Supplementary Figure S9 The correlation between ASR of 2019 and SDI from 1990 to 2019 in different subtypes of leukemia attributable to high BMI risk in 21 regions. A AML B ALL C CML D CLL E other leukemia. AML acute myeloid leukemia, ALL acute lymphoblastic leukemia, CML chronic myeloid leukemia, CLL chronic lymphocytic leukemia.

Supplementary Figure S10 The correlation between ASR of 2019 and SDI from 1990 to 2019 in different subtypes of leukemia attributable to high BMI risk in 204 countries and territories. A AML B ALL C CML D CLL E other leukemia. AML acute myeloid leukemia, ALL acute lymphoblastic leukemia, CML chronic myeloid leukemia, CLL chronic lymphocytic leukemia.
