# Supplementary material for: Global burden and trends of leukemia attributable to high body mass index risk in adults over the past 30 years
Source: Front Oncol. 2024 Jun 19;14:1404135. doi: 10.3389/fonc.2024.1404135 (PMC11219942; doi:10.3389/fonc.2024.1404135)
Supplement: Supplementary file 1 [file Table_1.docx]

| **Countries and territories** | **Causes** | **Measure** | **Cases in 1990 No.×10^3^** | **ASR in 1990 per 100000** | **Cases in 2019 No.×10^3^** | **ASR in 2019 per 100000** | **Change in absolute number (%)** | **EAPC from 1990 and 2019** |
| --- | --- | --- | --- | --- | --- | --- | --- | --- |
| Afghanistan | Leukemia | death | 0.029 (0.009 to 0.064) | 0.39 (0.12 to 0.86) | 0.089 (0.034 to 0.189) | 0.59 (0.25 to 1.16) | 2.09 (1.15 to 4.04) | 2.1 (1.52 to 2.68) |
| Afghanistan | Acute myeloid leukemia | death | 0.004 (0.001 to 0.009) | 0.05 (0.02 to 0.12) | 0.015 (0.005 to 0.034) | 0.09 (0.03 to 0.19) | 2.8 (1.47 to 5.27) | 2.64 (1.86 to 3.42) |
| Afghanistan | Acute lymphoid leukemia | death | 0.002 (0.001 to 0.006) | 0.03 (0.01 to 0.08) | 0.008 (0.002 to 0.019) | 0.04 (0.01 to 0.1) | 2.69 (1.51 to 5.33) | 2.27 (1.66 to 2.88) |
| Afghanistan | Chronic myeloid leukemia | death | 0.006 (0.001 to 0.015) | 0.07 (0.02 to 0.2) | 0.015 (0.004 to 0.04) | 0.09 (0.02 to 0.21) | 1.74 (0.73 to 3.57) | 1.08 (0.63 to 1.53) |
| Afghanistan | Chronic lymphoid leukemia | death | 0.001 (0 to 0.002) | 0.01 (0 to 0.02) | 0.003 (0.001 to 0.006) | 0.02 (0.01 to 0.04) | 2.54 (1.13 to 5.21) | 2.98 (2.3 to 3.66) |
| Afghanistan | Other leukemia | death | 0.016 (0.004 to 0.037) | 0.23 (0.06 to 0.51) | 0.048 (0.015 to 0.103) | 0.35 (0.13 to 0.7) | 1.95 (1.01 to 3.89) | 2.21 (1.65 to 2.77) |
| Afghanistan | Leukemia | DALYs | 0.969 (0.274 to 2.277) | 12.48 (3.5 to 29.2) | 3.295 (1.131 to 7.525) | 17.58 (6.87 to 36.96) | 2.4 (1.37 to 4.57) | 1.77 (1.23 to 2.32) |
| Afghanistan | Acute myeloid leukemia | DALYs | 0.132 (0.037 to 0.317) | 1.69 (0.48 to 3.96) | 0.579 (0.179 to 1.319) | 2.88 (0.99 to 6.4) | 3.37 (1.85 to 6.16) | 2.56 (1.79 to 3.33) |
| Afghanistan | Acute lymphoid leukemia | DALYs | 0.079 (0.018 to 0.256) | 0.98 (0.22 to 3.18) | 0.32 (0.091 to 0.84) | 1.47 (0.46 to 3.52) | 3.07 (1.73 to 6.07) | 2.02 (1.43 to 2.62) |
| Afghanistan | Chronic myeloid leukemia | DALYs | 0.203 (0.04 to 0.576) | 2.62 (0.52 to 7.55) | 0.603 (0.136 to 1.662) | 2.91 (0.7 to 7.57) | 1.97 (0.89 to 4.02) | 0.78 (0.36 to 1.2) |
| Afghanistan | Chronic lymphoid leukemia | DALYs | 0.022 (0.006 to 0.055) | 0.29 (0.07 to 0.7) | 0.087 (0.023 to 0.201) | 0.53 (0.15 to 1.2) | 2.89 (1.36 to 5.82) | 2.78 (2.14 to 3.42) |
| Afghanistan | Other leukemia | DALYs | 0.533 (0.109 to 1.273) | 6.9 (1.47 to 16.37) | 1.706 (0.444 to 3.921) | 9.79 (3.09 to 20.61) | 2.2 (1.19 to 4.22) | 1.83 (1.31 to 2.35) |
| Albania | Leukemia | death | 0.005 (0.002 to 0.009) | 0.23 (0.1 to 0.43) | 0.012 (0.006 to 0.023) | 0.3 (0.14 to 0.56) | 1.43 (0.69 to 2.5) | 1.28 (1.1 to 1.47) |
| Albania | Acute myeloid leukemia | death | 0.002 (0.001 to 0.003) | 0.08 (0.03 to 0.15) | 0.005 (0.002 to 0.009) | 0.11 (0.05 to 0.21) | 1.59 (0.72 to 2.91) | 1.74 (1.56 to 1.92) |
| Albania | Acute lymphoid leukemia | death | 0.001 (0 to 0.001) | 0.02 (0.01 to 0.05) | 0.001 (0 to 0.002) | 0.03 (0.01 to 0.06) | 0.92 (0.29 to 1.97) | 1.18 (1.02 to 1.34) |
| Albania | Chronic myeloid leukemia | death | 0 (0 to 0.001) | 0.01 (0 to 0.03) | 0 (0 to 0.001) | 0.01 (0 to 0.02) | 0.5 (-0.06 to 1.46) | -0.23 (-0.51 to 0.05) |
| Albania | Chronic lymphoid leukemia | death | 0 (0 to 0.001) | 0.02 (0.01 to 0.04) | 0.002 (0.001 to 0.004) | 0.06 (0.02 to 0.1) | 5.45 (3.12 to 8.77) | 4.41 (4.01 to 4.82) |
| Albania | Other leukemia | death | 0.002 (0.001 to 0.004) | 0.1 (0.04 to 0.2) | 0.004 (0.002 to 0.008) | 0.09 (0.04 to 0.18) | 0.85 (0.23 to 1.86) | -0.03 (-0.2 to 0.14) |
| Albania | Leukemia | DALYs | 0.162 (0.065 to 0.303) | 6.51 (2.72 to 11.99) | 0.33 (0.154 to 0.608) | 8.83 (4.06 to 16.57) | 1.03 (0.4 to 2.03) | 1.45 (1.25 to 1.64) |
| Albania | Acute myeloid leukemia | DALYs | 0.059 (0.023 to 0.109) | 2.33 (0.93 to 4.35) | 0.131 (0.055 to 0.242) | 3.57 (1.49 to 6.58) | 1.22 (0.48 to 2.46) | 1.95 (1.76 to 2.15) |
| Albania | Acute lymphoid leukemia | DALYs | 0.023 (0.008 to 0.046) | 0.81 (0.3 to 1.62) | 0.04 (0.017 to 0.078) | 1.22 (0.5 to 2.32) | 0.75 (0.14 to 1.76) | 1.73 (1.55 to 1.91) |
| Albania | Chronic myeloid leukemia | DALYs | 0.009 (0.003 to 0.02) | 0.38 (0.13 to 0.82) | 0.012 (0.004 to 0.025) | 0.32 (0.12 to 0.68) | 0.25 (-0.23 to 1.07) | 0.03 (-0.27 to 0.34) |
| Albania | Chronic lymphoid leukemia | DALYs | 0.009 (0.004 to 0.018) | 0.43 (0.17 to 0.81) | 0.054 (0.024 to 0.103) | 1.28 (0.56 to 2.42) | 4.92 (2.76 to 8.15) | 4.58 (4.16 to 4.99) |
| Albania | Other leukemia | DALYs | 0.062 (0.024 to 0.121) | 2.56 (1.02 to 5.04) | 0.093 (0.039 to 0.188) | 2.44 (1.02 to 4.82) | 0.5 (0 to 1.34) | 0.01 (-0.17 to 0.18) |
| Algeria | Leukemia | death | 0.03 (0.013 to 0.054) | 0.24 (0.1 to 0.43) | 0.095 (0.048 to 0.158) | 0.28 (0.14 to 0.47) | 2.15 (1.26 to 3.69) | 0.71 (0.61 to 0.81) |
| Algeria | Acute myeloid leukemia | death | 0.007 (0.003 to 0.014) | 0.05 (0.02 to 0.1) | 0.029 (0.015 to 0.049) | 0.08 (0.04 to 0.13) | 3.05 (1.75 to 5.53) | 1.64 (1.54 to 1.73) |
| Algeria | Acute lymphoid leukemia | death | 0.001 (0 to 0.002) | 0 (0 to 0.01) | 0.003 (0.001 to 0.005) | 0.01 (0 to 0.01) | 2.32 (1.09 to 4.8) | 1.05 (0.98 to 1.12) |
| Algeria | Chronic myeloid leukemia | death | 0.008 (0.004 to 0.015) | 0.06 (0.03 to 0.11) | 0.019 (0.01 to 0.032) | 0.05 (0.03 to 0.09) | 1.27 (0.53 to 2.63) | -0.44 (-0.6 to -0.28) |
| Algeria | Chronic lymphoid leukemia | death | 0.005 (0.002 to 0.01) | 0.05 (0.02 to 0.09) | 0.022 (0.009 to 0.039) | 0.07 (0.03 to 0.13) | 3.08 (1.72 to 5.29) | 1.4 (1.33 to 1.47) |
| Algeria | Other leukemia | death | 0.009 (0.003 to 0.017) | 0.07 (0.03 to 0.13) | 0.023 (0.011 to 0.039) | 0.07 (0.03 to 0.12) | 1.65 (0.77 to 3.69) | 0.13 (0.04 to 0.23) |
| Algeria | Leukemia | DALYs | 1.044 (0.452 to 1.915) | 6.9 (3.08 to 12.43) | 2.96 (1.498 to 4.915) | 7.66 (3.87 to 12.64) | 1.84 (1 to 3.32) | 0.42 (0.32 to 0.53) |
| Algeria | Acute myeloid leukemia | DALYs | 0.261 (0.115 to 0.501) | 1.66 (0.75 to 3.12) | 1.007 (0.505 to 1.688) | 2.49 (1.26 to 4.17) | 2.86 (1.56 to 5.32) | 1.53 (1.45 to 1.62) |
| Algeria | Acute lymphoid leukemia | DALYs | 0.035 (0.013 to 0.074) | 0.19 (0.08 to 0.39) | 0.111 (0.055 to 0.189) | 0.26 (0.13 to 0.45) | 2.14 (0.86 to 4.82) | 1.05 (0.99 to 1.11) |
| Algeria | Chronic myeloid leukemia | DALYs | 0.321 (0.137 to 0.585) | 2.02 (0.88 to 3.62) | 0.667 (0.338 to 1.147) | 1.63 (0.81 to 2.78) | 1.08 (0.34 to 2.47) | -0.72 (-0.88 to -0.55) |
| Algeria | Chronic lymphoid leukemia | DALYs | 0.134 (0.055 to 0.261) | 1.08 (0.44 to 2.06) | 0.505 (0.214 to 0.908) | 1.5 (0.64 to 2.68) | 2.76 (1.46 to 4.89) | 1.09 (1.03 to 1.15) |
| Algeria | Other leukemia | DALYs | 0.292 (0.116 to 0.568) | 1.93 (0.8 to 3.73) | 0.671 (0.323 to 1.187) | 1.78 (0.86 to 3.15) | 1.3 (0.51 to 3.1) | -0.24 (-0.33 to -0.14) |
| American Samoa | Leukemia | death | 0 (0 to 0) | 0.39 (0.2 to 0.63) | 0 (0 to 0) | 0.36 (0.18 to 0.59) | 0.67 (0.28 to 1.17) | -0.62 (-0.77 to -0.48) |
| American Samoa | Acute myeloid leukemia | death | 0 (0 to 0) | 0.11 (0.06 to 0.17) | 0 (0 to 0) | 0.08 (0.05 to 0.14) | 0.34 (-0.05 to 0.98) | -1.26 (-1.45 to -1.07) |
| American Samoa | Acute lymphoid leukemia | death | 0 (0 to 0) | 0.04 (0.02 to 0.06) | 0 (0 to 0) | 0.03 (0.02 to 0.06) | 0.43 (-0.04 to 1.16) | -0.59 (-1.21 to 0.03) |
| American Samoa | Chronic myeloid leukemia | death | 0 (0 to 0) | 0.07 (0.04 to 0.12) | 0 (0 to 0) | 0.04 (0.02 to 0.07) | 0.18 (-0.17 to 0.7) | -2.23 (-2.63 to -1.82) |
| American Samoa | Chronic lymphoid leukemia | death | 0 (0 to 0) | 0.01 (0 to 0.02) | 0 (0 to 0) | 0.01 (0 to 0.02) | 0.94 (0.3 to 1.88) | -0.82 (-1.3 to -0.33) |
| American Samoa | Other leukemia | death | 0 (0 to 0) | 0.17 (0.08 to 0.29) | 0 (0 to 0) | 0.19 (0.09 to 0.32) | 1.24 (0.61 to 2.06) | 0.26 (0.04 to 0.47) |
| American Samoa | Leukemia | DALYs | 0.004 (0.002 to 0.006) | 11.96 (6.53 to 18.61) | 0.006 (0.003 to 0.009) | 11.26 (5.96 to 18.19) | 0.48 (0.1 to 0.99) | -0.54 (-0.7 to -0.39) |
| American Samoa | Acute myeloid leukemia | DALYs | 0.001 (0.001 to 0.002) | 3.76 (2.02 to 5.93) | 0.002 (0.001 to 0.003) | 3.13 (1.66 to 5.16) | 0.22 (-0.15 to 0.81) | -1.1 (-1.29 to -0.92) |
| American Samoa | Acute lymphoid leukemia | DALYs | 0.001 (0 to 0.001) | 1.33 (0.68 to 2.22) | 0.001 (0 to 0.001) | 1.24 (0.61 to 2.26) | 0.3 (-0.16 to 1.05) | -0.46 (-0.92 to 0.01) |
| American Samoa | Chronic myeloid leukemia | DALYs | 0.001 (0 to 0.001) | 2.21 (1.18 to 3.61) | 0.001 (0 to 0.001) | 1.43 (0.71 to 2.37) | 0.09 (-0.25 to 0.61) | -2.14 (-2.55 to -1.73) |
| American Samoa | Chronic lymphoid leukemia | DALYs | 0 (0 to 0) | 0.26 (0.13 to 0.44) | 0 (0 to 0) | 0.27 (0.13 to 0.46) | 0.77 (0.21 to 1.62) | -0.54 (-0.93 to -0.14) |
| American Samoa | Other leukemia | DALYs | 0.001 (0.001 to 0.002) | 4.4 (2.24 to 7.38) | 0.003 (0.001 to 0.004) | 5.19 (2.63 to 8.73) | 1.02 (0.4 to 1.8) | 0.46 (0.26 to 0.67) |
| Andorra | Leukemia | death | 0 (0 to 0) | 0.51 (0.23 to 0.89) | 0.001 (0 to 0.001) | 0.48 (0.22 to 0.83) | 1.51 (0.72 to 2.54) | -0.16 (-0.28 to -0.05) |
| Andorra | Acute myeloid leukemia | death | 0 (0 to 0) | 0.23 (0.1 to 0.41) | 0 (0 to 0.001) | 0.25 (0.11 to 0.44) | 1.67 (0.8 to 2.93) | 0.36 (0.27 to 0.46) |
| Andorra | Acute lymphoid leukemia | death | 0 (0 to 0) | 0.03 (0.01 to 0.06) | 0 (0 to 0) | 0.03 (0.01 to 0.06) | 1.04 (0.34 to 2.07) | -0.13 (-0.23 to -0.02) |
| Andorra | Chronic myeloid leukemia | death | 0 (0 to 0) | 0.06 (0.03 to 0.12) | 0 (0 to 0) | 0.03 (0.01 to 0.05) | 0.27 (-0.19 to 0.96) | -2.77 (-2.95 to -2.59) |
| Andorra | Chronic lymphoid leukemia | death | 0 (0 to 0) | 0.09 (0.04 to 0.17) | 0 (0 to 0) | 0.09 (0.04 to 0.17) | 2.28 (1.1 to 3.94) | 0 (-0.08 to 0.08) |
| Andorra | Other leukemia | death | 0 (0 to 0) | 0.09 (0.04 to 0.18) | 0 (0 to 0) | 0.08 (0.04 to 0.15) | 1.44 (0.61 to 2.69) | -0.51 (-0.71 to -0.31) |
| Andorra | Leukemia | DALYs | 0.007 (0.003 to 0.013) | 12.52 (5.68 to 22.17) | 0.016 (0.007 to 0.027) | 12.31 (5.87 to 21.09) | 1.14 (0.43 to 2.08) | -0.09 (-0.16 to -0.01) |
| Andorra | Acute myeloid leukemia | DALYs | 0.004 (0.002 to 0.006) | 5.88 (2.61 to 10.48) | 0.008 (0.004 to 0.014) | 6.32 (2.97 to 10.91) | 1.3 (0.53 to 2.37) | 0.31 (0.22 to 0.4) |
| Andorra | Acute lymphoid leukemia | DALYs | 0.001 (0 to 0.001) | 1.24 (0.5 to 2.29) | 0.002 (0.001 to 0.003) | 1.53 (0.7 to 2.7) | 1.11 (0.34 to 2.25) | 0.58 (0.51 to 0.65) |
| Andorra | Chronic myeloid leukemia | DALYs | 0.001 (0 to 0.002) | 1.64 (0.67 to 3.16) | 0.001 (0.001 to 0.002) | 0.91 (0.41 to 1.62) | 0.19 (-0.27 to 0.84) | -2.32 (-2.43 to -2.2) |
| Andorra | Chronic lymphoid leukemia | DALYs | 0.001 (0 to 0.002) | 1.74 (0.72 to 3.33) | 0.003 (0.001 to 0.005) | 1.88 (0.82 to 3.35) | 1.81 (0.78 to 3.29) | 0.11 (0.05 to 0.17) |
| Andorra | Other leukemia | DALYs | 0.001 (0 to 0.002) | 2.02 (0.84 to 3.91) | 0.002 (0.001 to 0.004) | 1.68 (0.77 to 3.22) | 0.93 (0.26 to 1.93) | -0.58 (-0.77 to -0.4) |
| Angola | Leukemia | death | 0.002 (0 to 0.005) | 0.04 (0.01 to 0.11) | 0.014 (0.005 to 0.028) | 0.11 (0.04 to 0.22) | 6.21 (3.08 to 21.02) | 3.55 (3.35 to 3.76) |
| Angola | Acute myeloid leukemia | death | 0 (0 to 0.001) | 0 (0 to 0.01) | 0.002 (0.001 to 0.004) | 0.01 (0.01 to 0.03) | 8.61 (4.15 to 29.57) | 4.58 (4.21 to 4.94) |
| Angola | Acute lymphoid leukemia | death | 0 (0 to 0) | 0 (0 to 0) | 0.001 (0 to 0.001) | 0 (0 to 0.01) | 7.07 (3.29 to 25.56) | 3.71 (3.43 to 3.99) |
| Angola | Chronic myeloid leukemia | death | 0 (0 to 0.001) | 0.01 (0 to 0.02) | 0.003 (0.001 to 0.006) | 0.02 (0.01 to 0.04) | 6.28 (2.95 to 23.59) | 3.73 (3.38 to 4.09) |
| Angola | Chronic lymphoid leukemia | death | 0 (0 to 0.001) | 0 (0 to 0.02) | 0.003 (0.001 to 0.007) | 0.03 (0.01 to 0.07) | 17.79 (8.99 to 61.14) | 7.32 (7.05 to 7.59) |
| Angola | Other leukemia | death | 0.001 (0 to 0.003) | 0.02 (0 to 0.07) | 0.005 (0.002 to 0.011) | 0.04 (0.01 to 0.09) | 3.84 (1.62 to 14.28) | 1.75 (1.6 to 1.91) |
| Angola | Leukemia | DALYs | 0.073 (0.012 to 0.2) | 1.28 (0.22 to 3.41) | 0.506 (0.187 to 0.999) | 3.16 (1.18 to 6.28) | 5.95 (2.87 to 20.68) | 3.44 (3.22 to 3.67) |
| Angola | Acute myeloid leukemia | DALYs | 0.008 (0.001 to 0.027) | 0.14 (0.02 to 0.42) | 0.081 (0.029 to 0.166) | 0.46 (0.18 to 0.92) | 8.64 (4.14 to 30.07) | 4.63 (4.24 to 5.01) |
| Angola | Acute lymphoid leukemia | DALYs | 0.004 (0.001 to 0.012) | 0.05 (0.01 to 0.16) | 0.03 (0.01 to 0.065) | 0.15 (0.05 to 0.31) | 7.21 (3.32 to 25.89) | 3.88 (3.57 to 4.19) |
| Angola | Chronic myeloid leukemia | DALYs | 0.015 (0.002 to 0.047) | 0.25 (0.04 to 0.75) | 0.106 (0.035 to 0.233) | 0.62 (0.21 to 1.29) | 6.22 (2.87 to 24.3) | 3.73 (3.37 to 4.1) |
| Angola | Chronic lymphoid leukemia | DALYs | 0.005 (0.001 to 0.015) | 0.11 (0.02 to 0.36) | 0.09 (0.029 to 0.188) | 0.73 (0.24 to 1.56) | 17.75 (8.58 to 62.03) | 7.33 (7.05 to 7.61) |
| Angola | Other leukemia | DALYs | 0.041 (0.007 to 0.115) | 0.73 (0.12 to 2.02) | 0.199 (0.066 to 0.423) | 1.21 (0.41 to 2.54) | 3.82 (1.58 to 14.48) | 1.79 (1.63 to 1.94) |
| Antigua and Barbuda | Leukemia | death | 0 (0 to 0) | 0.2 (0.08 to 0.37) | 0 (0 to 0.001) | 0.31 (0.15 to 0.54) | 1.88 (1.18 to 3.3) | 1.38 (1.28 to 1.49) |
| Antigua and Barbuda | Acute myeloid leukemia | death | 0 (0 to 0) | 0.03 (0.01 to 0.06) | 0 (0 to 0) | 0.07 (0.04 to 0.13) | 3.49 (2.17 to 6.12) | 3.1 (2.98 to 3.22) |
| Antigua and Barbuda | Acute lymphoid leukemia | death | 0 (0 to 0) | 0.01 (0 to 0.02) | 0 (0 to 0) | 0.02 (0.01 to 0.03) | 2.52 (1.5 to 4.73) | 2.33 (2.22 to 2.44) |
| Antigua and Barbuda | Chronic myeloid leukemia | death | 0 (0 to 0) | 0.04 (0.01 to 0.07) | 0 (0 to 0) | 0.04 (0.02 to 0.07) | 1 (0.42 to 2.09) | -0.34 (-0.55 to -0.13) |
| Antigua and Barbuda | Chronic lymphoid leukemia | death | 0 (0 to 0) | 0 (0 to 0.01) | 0 (0 to 0) | 0.01 (0 to 0.01) | 2.67 (1.4 to 5.17) | 2.43 (2.19 to 2.68) |
| Antigua and Barbuda | Other leukemia | death | 0 (0 to 0) | 0.12 (0.05 to 0.23) | 0 (0 to 0) | 0.17 (0.08 to 0.3) | 1.64 (0.98 to 2.97) | 1.13 (1.02 to 1.24) |
| Antigua and Barbuda | Leukemia | DALYs | 0.003 (0.001 to 0.006) | 6.07 (2.48 to 11.16) | 0.009 (0.004 to 0.016) | 8.63 (4.16 to 15.16) | 1.81 (1.11 to 3.19) | 1.13 (1.05 to 1.22) |
| Antigua and Barbuda | Acute myeloid leukemia | DALYs | 0.001 (0 to 0.001) | 1.12 (0.43 to 2.15) | 0.003 (0.001 to 0.004) | 2.41 (1.15 to 4.21) | 3.26 (2.02 to 5.75) | 2.91 (2.8 to 3.03) |
| Antigua and Barbuda | Acute lymphoid leukemia | DALYs | 0 (0 to 0) | 0.32 (0.12 to 0.63) | 0.001 (0 to 0.001) | 0.56 (0.26 to 1) | 2.23 (1.26 to 4.45) | 2.09 (1.97 to 2.2) |
| Antigua and Barbuda | Chronic myeloid leukemia | DALYs | 0.001 (0 to 0.001) | 1.06 (0.42 to 1.96) | 0.001 (0.001 to 0.002) | 1.02 (0.5 to 1.77) | 0.93 (0.37 to 2) | -0.65 (-0.85 to -0.44) |
| Antigua and Barbuda | Chronic lymphoid leukemia | DALYs | 0 (0 to 0) | 0.08 (0.03 to 0.17) | 0 (0 to 0) | 0.15 (0.08 to 0.27) | 2.69 (1.45 to 5.15) | 2.16 (1.97 to 2.35) |
| Antigua and Barbuda | Other leukemia | DALYs | 0.002 (0.001 to 0.003) | 3.48 (1.4 to 6.34) | 0.005 (0.002 to 0.008) | 4.49 (2.13 to 7.87) | 1.54 (0.9 to 2.81) | 0.74 (0.65 to 0.82) |
| Argentina | Leukemia | death | 0.091 (0.035 to 0.168) | 0.29 (0.11 to 0.53) | 0.21 (0.096 to 0.354) | 0.39 (0.18 to 0.66) | 1.31 (0.96 to 2.08) | 0.74 (0.45 to 1.03) |
| Argentina | Acute myeloid leukemia | death | 0.02 (0.008 to 0.037) | 0.06 (0.02 to 0.12) | 0.062 (0.029 to 0.106) | 0.12 (0.05 to 0.2) | 2.07 (1.46 to 3.35) | 1.99 (1.64 to 2.34) |
| Argentina | Acute lymphoid leukemia | death | 0.008 (0.003 to 0.015) | 0.03 (0.01 to 0.05) | 0.022 (0.01 to 0.039) | 0.04 (0.02 to 0.08) | 1.75 (1.17 to 2.96) | 1.76 (1.52 to 1.99) |
| Argentina | Chronic myeloid leukemia | death | 0.015 (0.006 to 0.028) | 0.05 (0.02 to 0.09) | 0.015 (0.007 to 0.026) | 0.03 (0.01 to 0.05) | 0.01 (-0.22 to 0.44) | -2.67 (-3.19 to -2.14) |
| Argentina | Chronic lymphoid leukemia | death | 0.008 (0.003 to 0.016) | 0.03 (0.01 to 0.05) | 0.019 (0.008 to 0.033) | 0.03 (0.02 to 0.06) | 1.26 (0.71 to 2.29) | 0.27 (-0.23 to 0.77) |
| Argentina | Other leukemia | death | 0.039 (0.016 to 0.074) | 0.13 (0.05 to 0.24) | 0.092 (0.042 to 0.155) | 0.17 (0.08 to 0.29) | 1.33 (0.91 to 2.22) | 0.73 (0.5 to 0.96) |
| Argentina | Leukemia | DALYs | 2.532 (0.98 to 4.785) | 7.85 (3.02 to 14.86) | 5.359 (2.507 to 8.984) | 10.46 (4.89 to 17.51) | 1.12 (0.78 to 1.89) | 0.64 (0.38 to 0.91) |
| Argentina | Acute myeloid leukemia | DALYs | 0.61 (0.234 to 1.145) | 1.89 (0.72 to 3.55) | 1.75 (0.818 to 3.011) | 3.45 (1.61 to 5.94) | 1.87 (1.3 to 3.15) | 1.9 (1.55 to 2.24) |
| Argentina | Acute lymphoid leukemia | DALYs | 0.292 (0.105 to 0.566) | 0.91 (0.33 to 1.77) | 0.772 (0.353 to 1.328) | 1.56 (0.72 to 2.69) | 1.65 (1.03 to 2.93) | 1.8 (1.59 to 2.02) |
| Argentina | Chronic myeloid leukemia | DALYs | 0.409 (0.157 to 0.776) | 1.27 (0.48 to 2.41) | 0.358 (0.17 to 0.611) | 0.7 (0.33 to 1.19) | -0.12 (-0.31 to 0.25) | -3.02 (-3.54 to -2.49) |
| Argentina | Chronic lymphoid leukemia | DALYs | 0.172 (0.069 to 0.325) | 0.53 (0.21 to 1) | 0.349 (0.16 to 0.612) | 0.65 (0.3 to 1.14) | 1.03 (0.55 to 1.9) | 0.09 (-0.4 to 0.58) |
| Argentina | Other leukemia | DALYs | 1.049 (0.409 to 1.983) | 3.26 (1.27 to 6.16) | 2.13 (0.999 to 3.597) | 4.1 (1.92 to 6.93) | 1.03 (0.67 to 1.82) | 0.45 (0.23 to 0.66) |
| Armenia | Leukemia | death | 0.009 (0.004 to 0.016) | 0.3 (0.13 to 0.54) | 0.015 (0.008 to 0.025) | 0.38 (0.19 to 0.61) | 0.69 (0.31 to 1.34) | 0.85 (0.7 to 1.01) |
| Armenia | Acute myeloid leukemia | death | 0.001 (0.001 to 0.002) | 0.04 (0.02 to 0.07) | 0.004 (0.002 to 0.007) | 0.09 (0.04 to 0.16) | 2.2 (0.81 to 3.94) | 3.63 (3.17 to 4.09) |
| Armenia | Acute lymphoid leukemia | death | 0.002 (0.001 to 0.004) | 0.06 (0.02 to 0.11) | 0.002 (0.001 to 0.003) | 0.04 (0.02 to 0.07) | -0.14 (-0.41 to 0.81) | -1.48 (-1.99 to -0.97) |
| Armenia | Chronic myeloid leukemia | death | 0.001 (0 to 0.001) | 0.02 (0.01 to 0.04) | 0.001 (0 to 0.001) | 0.02 (0.01 to 0.03) | 0.27 (-0.11 to 0.97) | -0.24 (-0.51 to 0.02) |
| Armenia | Chronic lymphoid leukemia | death | 0.001 (0.001 to 0.003) | 0.05 (0.02 to 0.09) | 0.003 (0.001 to 0.005) | 0.07 (0.03 to 0.12) | 0.99 (0.39 to 2.1) | 1.11 (0.88 to 1.33) |
| Armenia | Other leukemia | death | 0.004 (0.002 to 0.007) | 0.13 (0.06 to 0.25) | 0.006 (0.003 to 0.01) | 0.16 (0.07 to 0.26) | 0.58 (0.2 to 1.26) | 0.6 (0.41 to 0.79) |
| Armenia | Leukemia | DALYs | 0.323 (0.139 to 0.581) | 10.18 (4.41 to 18.21) | 0.457 (0.233 to 0.741) | 11.62 (5.95 to 18.87) | 0.42 (0.07 to 1.04) | 0.47 (0.31 to 0.63) |
| Armenia | Acute myeloid leukemia | DALYs | 0.043 (0.019 to 0.079) | 1.39 (0.6 to 2.5) | 0.122 (0.057 to 0.211) | 3.11 (1.45 to 5.37) | 1.82 (0.58 to 3.53) | 3.35 (2.91 to 3.8) |
| Armenia | Acute lymphoid leukemia | DALYs | 0.074 (0.024 to 0.151) | 2.2 (0.74 to 4.41) | 0.058 (0.029 to 0.097) | 1.53 (0.76 to 2.54) | -0.22 (-0.46 to 0.65) | -1.61 (-2.1 to -1.11) |
| Armenia | Chronic myeloid leukemia | DALYs | 0.022 (0.01 to 0.04) | 0.71 (0.31 to 1.3) | 0.024 (0.012 to 0.04) | 0.6 (0.29 to 1.02) | 0.09 (-0.24 to 0.71) | -0.61 (-0.89 to -0.32) |
| Armenia | Chronic lymphoid leukemia | DALYs | 0.044 (0.018 to 0.08) | 1.46 (0.61 to 2.62) | 0.074 (0.036 to 0.127) | 1.77 (0.86 to 3.02) | 0.68 (0.15 to 1.71) | 0.73 (0.49 to 0.97) |
| Armenia | Other leukemia | DALYs | 0.139 (0.058 to 0.256) | 4.42 (1.85 to 8.15) | 0.179 (0.088 to 0.298) | 4.61 (2.26 to 7.67) | 0.29 (-0.05 to 0.88) | 0.13 (-0.08 to 0.33) |
| Australia | Leukemia | death | 0.084 (0.039 to 0.144) | 0.44 (0.2 to 0.74) | 0.203 (0.103 to 0.325) | 0.48 (0.25 to 0.76) | 1.4 (1.06 to 1.91) | 0.18 (0.09 to 0.26) |
| Australia | Acute myeloid leukemia | death | 0.038 (0.018 to 0.064) | 0.2 (0.09 to 0.33) | 0.108 (0.054 to 0.175) | 0.26 (0.13 to 0.42) | 1.84 (1.35 to 2.49) | 0.93 (0.85 to 1) |
| Australia | Acute lymphoid leukemia | death | 0.007 (0.003 to 0.012) | 0.03 (0.02 to 0.06) | 0.011 (0.006 to 0.018) | 0.03 (0.02 to 0.05) | 0.68 (0.36 to 1.26) | -0.95 (-1.23 to -0.67) |
| Australia | Chronic myeloid leukemia | death | 0.015 (0.007 to 0.026) | 0.08 (0.04 to 0.14) | 0.013 (0.007 to 0.022) | 0.03 (0.02 to 0.05) | -0.13 (-0.3 to 0.13) | -3.93 (-4.29 to -3.57) |
| Australia | Chronic lymphoid leukemia | death | 0.016 (0.007 to 0.028) | 0.08 (0.04 to 0.14) | 0.039 (0.019 to 0.067) | 0.08 (0.04 to 0.14) | 1.45 (0.99 to 2.14) | -0.22 (-0.39 to -0.05) |
| Australia | Other leukemia | death | 0.008 (0.004 to 0.015) | 0.04 (0.02 to 0.08) | 0.031 (0.015 to 0.053) | 0.07 (0.03 to 0.12) | 2.66 (1.76 to 3.7) | 2.22 (1.8 to 2.65) |
| Australia | Leukemia | DALYs | 2.055 (0.966 to 3.487) | 10.71 (5.04 to 18.18) | 4.166 (2.197 to 6.58) | 11.11 (5.91 to 17.49) | 1.03 (0.75 to 1.46) | -0.05 (-0.14 to 0.04) |
| Australia | Acute myeloid leukemia | DALYs | 0.959 (0.445 to 1.613) | 5 (2.32 to 8.42) | 2.297 (1.189 to 3.646) | 6.22 (3.28 to 9.85) | 1.4 (1.02 to 1.94) | 0.65 (0.59 to 0.72) |
| Australia | Acute lymphoid leukemia | DALYs | 0.23 (0.107 to 0.407) | 1.24 (0.58 to 2.19) | 0.367 (0.187 to 0.585) | 1.17 (0.6 to 1.87) | 0.59 (0.27 to 1.16) | -0.75 (-1.03 to -0.46) |
| Australia | Chronic myeloid leukemia | DALYs | 0.368 (0.174 to 0.624) | 1.93 (0.91 to 3.28) | 0.277 (0.143 to 0.452) | 0.75 (0.39 to 1.22) | -0.25 (-0.38 to -0.04) | -3.92 (-4.24 to -3.59) |
| Australia | Chronic lymphoid leukemia | DALYs | 0.319 (0.153 to 0.55) | 1.62 (0.78 to 2.78) | 0.696 (0.35 to 1.163) | 1.64 (0.83 to 2.74) | 1.18 (0.77 to 1.77) | -0.33 (-0.54 to -0.12) |
| Australia | Other leukemia | DALYs | 0.179 (0.083 to 0.305) | 0.92 (0.43 to 1.58) | 0.529 (0.269 to 0.87) | 1.33 (0.68 to 2.17) | 1.96 (1.26 to 2.76) | 1.89 (1.51 to 2.28) |
| Austria | Leukemia | death | 0.039 (0.017 to 0.068) | 0.33 (0.14 to 0.58) | 0.07 (0.032 to 0.12) | 0.38 (0.18 to 0.65) | 0.79 (0.51 to 1.15) | 0.62 (0.44 to 0.81) |
| Austria | Acute myeloid leukemia | death | 0.011 (0.005 to 0.019) | 0.09 (0.04 to 0.16) | 0.032 (0.014 to 0.057) | 0.18 (0.08 to 0.32) | 1.96 (1.01 to 2.8) | 2.7 (2.43 to 2.98) |
| Austria | Acute lymphoid leukemia | death | 0.002 (0.001 to 0.003) | 0.01 (0.01 to 0.03) | 0.003 (0.001 to 0.005) | 0.02 (0.01 to 0.03) | 0.79 (0.39 to 1.35) | 0.77 (0.56 to 0.97) |
| Austria | Chronic myeloid leukemia | death | 0.007 (0.003 to 0.013) | 0.06 (0.03 to 0.11) | 0.005 (0.002 to 0.009) | 0.03 (0.01 to 0.05) | -0.32 (-0.45 to -0.09) | -3.46 (-3.64 to -3.29) |
| Austria | Chronic lymphoid leukemia | death | 0.011 (0.005 to 0.019) | 0.09 (0.04 to 0.15) | 0.019 (0.009 to 0.034) | 0.09 (0.04 to 0.16) | 0.78 (0.44 to 1.39) | 0.33 (-0.17 to 0.83) |
| Austria | Other leukemia | death | 0.009 (0.004 to 0.015) | 0.07 (0.03 to 0.13) | 0.011 (0.005 to 0.02) | 0.06 (0.03 to 0.1) | 0.26 (0.01 to 0.67) | -0.56 (-0.85 to -0.28) |
| Austria | Leukemia | DALYs | 0.885 (0.386 to 1.55) | 8.14 (3.54 to 14.3) | 1.373 (0.649 to 2.329) | 8.69 (4.14 to 14.76) | 0.55 (0.33 to 0.88) | 0.32 (0.17 to 0.48) |
| Austria | Acute myeloid leukemia | DALYs | 0.265 (0.114 to 0.464) | 2.5 (1.08 to 4.4) | 0.656 (0.292 to 1.141) | 4.3 (1.96 to 7.39) | 1.48 (0.78 to 2.15) | 2.17 (1.92 to 2.41) |
| Austria | Acute lymphoid leukemia | DALYs | 0.054 (0.022 to 0.098) | 0.56 (0.23 to 1.03) | 0.1 (0.047 to 0.173) | 0.82 (0.39 to 1.41) | 0.85 (0.39 to 1.56) | 1.18 (0.92 to 1.43) |
| Austria | Chronic myeloid leukemia | DALYs | 0.173 (0.075 to 0.307) | 1.63 (0.71 to 2.89) | 0.112 (0.051 to 0.198) | 0.72 (0.33 to 1.27) | -0.35 (-0.48 to -0.13) | -3.42 (-3.62 to -3.23) |
| Austria | Chronic lymphoid leukemia | DALYs | 0.206 (0.092 to 0.36) | 1.73 (0.76 to 3.06) | 0.315 (0.142 to 0.562) | 1.71 (0.77 to 3.04) | 0.53 (0.23 to 1.16) | -0.02 (-0.5 to 0.46) |
| Austria | Other leukemia | DALYs | 0.188 (0.082 to 0.33) | 1.71 (0.75 to 3) | 0.189 (0.085 to 0.335) | 1.15 (0.52 to 2.04) | 0.01 (-0.19 to 0.34) | -1.11 (-1.41 to -0.81) |
| Azerbaijan | Leukemia | death | 0.014 (0.006 to 0.024) | 0.23 (0.1 to 0.42) | 0.034 (0.016 to 0.058) | 0.33 (0.16 to 0.57) | 1.48 (0.85 to 2.48) | 1.35 (1 to 1.7) |
| Azerbaijan | Acute myeloid leukemia | death | 0.003 (0.001 to 0.005) | 0.05 (0.02 to 0.09) | 0.009 (0.004 to 0.016) | 0.08 (0.04 to 0.15) | 2.32 (1.38 to 3.75) | 2.52 (2.01 to 3.04) |
| Azerbaijan | Acute lymphoid leukemia | death | 0.001 (0.001 to 0.003) | 0.02 (0.01 to 0.04) | 0.003 (0.002 to 0.006) | 0.03 (0.01 to 0.06) | 1.62 (0.9 to 2.87) | 1.41 (1.13 to 1.7) |
| Azerbaijan | Chronic myeloid leukemia | death | 0.001 (0 to 0.002) | 0.01 (0.01 to 0.03) | 0.002 (0.001 to 0.004) | 0.02 (0.01 to 0.04) | 1.18 (0.45 to 2.23) | 1.04 (0.55 to 1.53) |
| Azerbaijan | Chronic lymphoid leukemia | death | 0.001 (0 to 0.001) | 0.01 (0.01 to 0.02) | 0.002 (0.001 to 0.004) | 0.03 (0.01 to 0.05) | 2.66 (1.5 to 4.41) | 2.87 (2.5 to 3.23) |
| Azerbaijan | Other leukemia | death | 0.008 (0.004 to 0.015) | 0.14 (0.06 to 0.26) | 0.017 (0.008 to 0.032) | 0.17 (0.08 to 0.32) | 1.13 (0.54 to 2.13) | 0.77 (0.48 to 1.06) |
| Azerbaijan | Leukemia | DALYs | 0.545 (0.238 to 0.991) | 8.66 (3.81 to 15.52) | 1.236 (0.592 to 2.114) | 10.87 (5.18 to 18.67) | 1.27 (0.67 to 2.2) | 0.74 (0.37 to 1.12) |
| Azerbaijan | Acute myeloid leukemia | DALYs | 0.108 (0.045 to 0.208) | 1.71 (0.72 to 3.24) | 0.335 (0.147 to 0.629) | 2.88 (1.27 to 5.38) | 2.11 (1.21 to 3.52) | 2.21 (1.7 to 2.73) |
| Azerbaijan | Acute lymphoid leukemia | DALYs | 0.059 (0.023 to 0.115) | 0.9 (0.35 to 1.73) | 0.143 (0.061 to 0.264) | 1.23 (0.53 to 2.25) | 1.41 (0.7 to 2.61) | 1.01 (0.7 to 1.32) |
| Azerbaijan | Chronic myeloid leukemia | DALYs | 0.031 (0.012 to 0.061) | 0.51 (0.2 to 0.99) | 0.059 (0.024 to 0.117) | 0.53 (0.22 to 1.04) | 0.92 (0.25 to 1.92) | 0.21 (-0.28 to 0.7) |
| Azerbaijan | Chronic lymphoid leukemia | DALYs | 0.02 (0.008 to 0.036) | 0.36 (0.15 to 0.66) | 0.063 (0.029 to 0.111) | 0.64 (0.3 to 1.1) | 2.21 (1.24 to 3.78) | 2.07 (1.69 to 2.45) |
| Azerbaijan | Other leukemia | DALYs | 0.328 (0.141 to 0.61) | 5.18 (2.21 to 9.58) | 0.636 (0.298 to 1.15) | 5.59 (2.59 to 10.21) | 0.94 (0.38 to 1.92) | 0.06 (-0.26 to 0.38) |
| Bahamas | Leukemia | death | 0.001 (0 to 0.001) | 0.3 (0.14 to 0.5) | 0.001 (0.001 to 0.002) | 0.33 (0.17 to 0.56) | 1.43 (0.94 to 2.18) | 0.48 (0.4 to 0.57) |
| Bahamas | Acute myeloid leukemia | death | 0 (0 to 0) | 0.09 (0.04 to 0.15) | 0 (0 to 0.001) | 0.12 (0.06 to 0.21) | 1.88 (1.22 to 2.91) | 1.31 (1.21 to 1.41) |
| Bahamas | Acute lymphoid leukemia | death | 0 (0 to 0) | 0.02 (0.01 to 0.03) | 0 (0 to 0) | 0.02 (0.01 to 0.03) | 1.36 (0.82 to 2.21) | 0.85 (0.76 to 0.95) |
| Bahamas | Chronic myeloid leukemia | death | 0 (0 to 0) | 0.06 (0.03 to 0.1) | 0 (0 to 0) | 0.05 (0.02 to 0.08) | 0.84 (0.41 to 1.45) | -0.57 (-0.67 to -0.46) |
| Bahamas | Chronic lymphoid leukemia | death | 0 (0 to 0) | 0.05 (0.02 to 0.09) | 0 (0 to 0) | 0.06 (0.03 to 0.11) | 2.07 (1.42 to 3.08) | 1.05 (0.87 to 1.23) |
| Bahamas | Other leukemia | death | 0 (0 to 0) | 0.09 (0.04 to 0.15) | 0 (0 to 0.001) | 0.08 (0.04 to 0.14) | 1.06 (0.62 to 1.67) | -0.32 (-0.43 to -0.22) |
| Bahamas | Leukemia | DALYs | 0.02 (0.01 to 0.034) | 9.8 (4.66 to 16.32) | 0.046 (0.024 to 0.078) | 10.82 (5.57 to 18.36) | 1.25 (0.76 to 2.01) | 0.49 (0.41 to 0.56) |
| Bahamas | Acute myeloid leukemia | DALYs | 0.007 (0.003 to 0.012) | 3.23 (1.51 to 5.56) | 0.019 (0.01 to 0.033) | 4.43 (2.22 to 7.73) | 1.65 (1 to 2.67) | 1.32 (1.23 to 1.42) |
| Bahamas | Acute lymphoid leukemia | DALYs | 0.002 (0.001 to 0.003) | 0.69 (0.33 to 1.17) | 0.004 (0.002 to 0.006) | 0.87 (0.45 to 1.46) | 1.16 (0.62 to 1.97) | 0.9 (0.81 to 0.99) |
| Bahamas | Chronic myeloid leukemia | DALYs | 0.004 (0.002 to 0.006) | 1.87 (0.89 to 3.16) | 0.006 (0.003 to 0.011) | 1.53 (0.78 to 2.54) | 0.7 (0.28 to 1.33) | -0.59 (-0.69 to -0.49) |
| Bahamas | Chronic lymphoid leukemia | DALYs | 0.002 (0.001 to 0.003) | 1.18 (0.53 to 2.05) | 0.006 (0.003 to 0.01) | 1.42 (0.68 to 2.48) | 1.97 (1.31 to 2.98) | 0.98 (0.82 to 1.15) |
| Bahamas | Other leukemia | DALYs | 0.006 (0.003 to 0.01) | 2.82 (1.35 to 4.73) | 0.011 (0.005 to 0.018) | 2.57 (1.31 to 4.27) | 0.89 (0.46 to 1.5) | -0.39 (-0.48 to -0.3) |
| Bahrain | Leukemia | death | 0.001 (0.001 to 0.002) | 0.7 (0.34 to 1.14) | 0.005 (0.003 to 0.008) | 0.6 (0.31 to 0.95) | 2.79 (1.77 to 4.41) | -1.14 (-1.41 to -0.87) |
| Bahrain | Acute myeloid leukemia | death | 0 (0 to 0.001) | 0.17 (0.08 to 0.3) | 0.002 (0.001 to 0.003) | 0.19 (0.1 to 0.31) | 4.07 (2.44 to 6.73) | -0.38 (-0.63 to -0.14) |
| Bahrain | Acute lymphoid leukemia | death | 0 (0 to 0) | 0.02 (0.01 to 0.05) | 0 (0 to 0.001) | 0.02 (0.01 to 0.04) | 2.59 (1.36 to 4.8) | -0.82 (-1.06 to -0.58) |
| Bahrain | Chronic myeloid leukemia | death | 0 (0 to 0.001) | 0.2 (0.09 to 0.36) | 0.001 (0.001 to 0.002) | 0.12 (0.06 to 0.2) | 1.54 (0.59 to 3.29) | -2.71 (-3.02 to -2.4) |
| Bahrain | Chronic lymphoid leukemia | death | 0 (0 to 0) | 0.2 (0.08 to 0.37) | 0.001 (0 to 0.002) | 0.17 (0.07 to 0.3) | 2.57 (1.44 to 4.21) | -1.27 (-1.65 to -0.9) |
| Bahrain | Other leukemia | death | 0 (0 to 0) | 0.09 (0.04 to 0.17) | 0.001 (0 to 0.001) | 0.09 (0.05 to 0.16) | 3.39 (1.74 to 6.81) | 0.11 (-0.33 to 0.56) |
| Bahrain | Leukemia | DALYs | 0.05 (0.025 to 0.08) | 17.42 (8.77 to 28.5) | 0.177 (0.091 to 0.286) | 13.87 (7.18 to 21.92) | 2.53 (1.58 to 4.08) | -1.49 (-1.73 to -1.25) |
| Bahrain | Acute myeloid leukemia | DALYs | 0.016 (0.008 to 0.027) | 4.87 (2.39 to 8.3) | 0.073 (0.036 to 0.127) | 5.15 (2.6 to 8.32) | 3.57 (2.1 to 5.96) | -0.55 (-0.8 to -0.3) |
| Bahrain | Acute lymphoid leukemia | DALYs | 0.004 (0.002 to 0.008) | 0.89 (0.39 to 1.67) | 0.013 (0.007 to 0.025) | 0.86 (0.43 to 1.51) | 2.23 (1.06 to 4.21) | -0.82 (-1.13 to -0.52) |
| Bahrain | Chronic myeloid leukemia | DALYs | 0.017 (0.008 to 0.028) | 5.45 (2.57 to 9.26) | 0.04 (0.02 to 0.068) | 2.87 (1.46 to 4.81) | 1.4 (0.53 to 3.05) | -3.17 (-3.48 to -2.85) |
| Bahrain | Chronic lymphoid leukemia | DALYs | 0.007 (0.003 to 0.012) | 3.96 (1.59 to 7.1) | 0.024 (0.01 to 0.042) | 2.86 (1.22 to 5.05) | 2.57 (1.41 to 4.39) | -1.87 (-2.24 to -1.51) |
| Bahrain | Other leukemia | DALYs | 0.007 (0.003 to 0.012) | 2.25 (1.07 to 4.11) | 0.027 (0.013 to 0.046) | 2.14 (1.04 to 3.58) | 3.03 (1.53 to 6.36) | -0.34 (-0.76 to 0.07) |
| Bangladesh | Leukemia | death | 0.014 (0.002 to 0.041) | 0.03 (0 to 0.07) | 0.079 (0.028 to 0.165) | 0.06 (0.02 to 0.12) | 4.47 (1.96 to 16.29) | 3.14 (2.93 to 3.35) |
| Bangladesh | Acute myeloid leukemia | death | 0.003 (0 to 0.008) | 0 (0 to 0.01) | 0.023 (0.009 to 0.05) | 0.02 (0.01 to 0.04) | 7.43 (3.42 to 24.96) | 4.63 (4.46 to 4.8) |
| Bangladesh | Acute lymphoid leukemia | death | 0.001 (0 to 0.003) | 0 (0 to 0) | 0.006 (0.002 to 0.013) | 0 (0 to 0.01) | 4.34 (1.57 to 18.19) | 3.72 (3.45 to 3.98) |
| Bangladesh | Chronic myeloid leukemia | death | 0.005 (0.001 to 0.015) | 0.01 (0 to 0.03) | 0.02 (0.007 to 0.043) | 0.01 (0 to 0.03) | 2.75 (0.77 to 12.87) | 2.04 (1.8 to 2.29) |
| Bangladesh | Chronic lymphoid leukemia | death | 0.002 (0 to 0.006) | 0 (0 to 0.01) | 0.016 (0.005 to 0.036) | 0.01 (0 to 0.03) | 7.13 (3.55 to 24.54) | 3.99 (3.75 to 4.24) |
| Bangladesh | Other leukemia | death | 0.003 (0 to 0.01) | 0.01 (0 to 0.02) | 0.014 (0.004 to 0.03) | 0.01 (0 to 0.02) | 3.17 (1.13 to 13.55) | 1.98 (1.77 to 2.2) |
| Bangladesh | Leukemia | DALYs | 0.542 (0.09 to 1.515) | 0.82 (0.13 to 2.31) | 2.647 (0.938 to 5.526) | 1.78 (0.63 to 3.7) | 3.88 (1.57 to 15.15) | 3.27 (3.03 to 3.52) |
| Bangladesh | Acute myeloid leukemia | DALYs | 0.108 (0.017 to 0.311) | 0.16 (0.03 to 0.45) | 0.834 (0.301 to 1.768) | 0.55 (0.2 to 1.16) | 6.75 (2.95 to 23.83) | 4.85 (4.64 to 5.07) |
| Bangladesh | Acute lymphoid leukemia | DALYs | 0.055 (0.008 to 0.159) | 0.07 (0.01 to 0.19) | 0.263 (0.094 to 0.562) | 0.16 (0.06 to 0.35) | 3.78 (1.18 to 16.85) | 3.74 (3.44 to 4.04) |
| Bangladesh | Chronic myeloid leukemia | DALYs | 0.213 (0.035 to 0.624) | 0.31 (0.05 to 0.89) | 0.721 (0.245 to 1.576) | 0.48 (0.16 to 1.04) | 2.39 (0.55 to 11.91) | 2.12 (1.83 to 2.4) |
| Bangladesh | Chronic lymphoid leukemia | DALYs | 0.049 (0.007 to 0.147) | 0.1 (0.01 to 0.3) | 0.398 (0.131 to 0.886) | 0.3 (0.1 to 0.65) | 7.09 (3.38 to 25.2) | 4.35 (4.08 to 4.62) |
| Bangladesh | Other leukemia | DALYs | 0.117 (0.017 to 0.355) | 0.18 (0.03 to 0.55) | 0.429 (0.136 to 0.931) | 0.3 (0.09 to 0.64) | 2.66 (0.77 to 13.21) | 2.12 (1.89 to 2.35) |
| Barbados | Leukemia | death | 0.001 (0 to 0.002) | 0.39 (0.17 to 0.67) | 0.002 (0.001 to 0.004) | 0.47 (0.23 to 0.82) | 0.96 (0.6 to 1.52) | 0.64 (0.51 to 0.77) |
| Barbados | Acute myeloid leukemia | death | 0 (0 to 0.001) | 0.12 (0.05 to 0.22) | 0.001 (0 to 0.001) | 0.19 (0.09 to 0.33) | 1.42 (0.89 to 2.18) | 1.51 (1.33 to 1.69) |
| Barbados | Acute lymphoid leukemia | death | 0 (0 to 0) | 0.01 (0.01 to 0.02) | 0 (0 to 0) | 0.02 (0.01 to 0.03) | 0.97 (0.48 to 1.67) | 0.96 (0.79 to 1.12) |
| Barbados | Chronic myeloid leukemia | death | 0 (0 to 0) | 0.04 (0.02 to 0.08) | 0 (0 to 0) | 0.05 (0.02 to 0.08) | 0.76 (0.33 to 1.38) | 0.15 (0.03 to 0.27) |
| Barbados | Chronic lymphoid leukemia | death | 0 (0 to 0) | 0.03 (0.01 to 0.05) | 0 (0 to 0) | 0.04 (0.02 to 0.07) | 1.62 (0.99 to 2.57) | 1.64 (1.56 to 1.73) |
| Barbados | Other leukemia | death | 0.001 (0 to 0.001) | 0.18 (0.08 to 0.3) | 0.001 (0 to 0.001) | 0.18 (0.09 to 0.3) | 0.61 (0.31 to 1.06) | -0.21 (-0.37 to -0.06) |
| Barbados | Leukemia | DALYs | 0.031 (0.014 to 0.053) | 11.68 (5.28 to 19.91) | 0.057 (0.028 to 0.098) | 13.8 (6.79 to 23.6) | 0.84 (0.47 to 1.38) | 0.47 (0.34 to 0.59) |
| Barbados | Acute myeloid leukemia | DALYs | 0.011 (0.005 to 0.019) | 4.32 (1.88 to 7.38) | 0.025 (0.011 to 0.043) | 6.15 (2.86 to 10.63) | 1.17 (0.7 to 1.88) | 1.27 (1.12 to 1.42) |
| Barbados | Acute lymphoid leukemia | DALYs | 0.001 (0.001 to 0.002) | 0.54 (0.24 to 0.92) | 0.002 (0.001 to 0.004) | 0.68 (0.33 to 1.17) | 0.7 (0.28 to 1.33) | 0.69 (0.52 to 0.85) |
| Barbados | Chronic myeloid leukemia | DALYs | 0.003 (0.002 to 0.006) | 1.3 (0.59 to 2.19) | 0.006 (0.003 to 0.01) | 1.33 (0.67 to 2.25) | 0.64 (0.24 to 1.21) | -0.08 (-0.21 to 0.04) |
| Barbados | Chronic lymphoid leukemia | DALYs | 0.002 (0.001 to 0.003) | 0.6 (0.27 to 1.05) | 0.005 (0.002 to 0.008) | 0.94 (0.45 to 1.61) | 1.69 (1.03 to 2.69) | 1.56 (1.48 to 1.64) |
| Barbados | Other leukemia | DALYs | 0.013 (0.006 to 0.023) | 4.92 (2.26 to 8.33) | 0.02 (0.01 to 0.034) | 4.69 (2.36 to 7.92) | 0.51 (0.19 to 0.94) | -0.45 (-0.61 to -0.29) |
| Belarus | Leukemia | death | 0.055 (0.024 to 0.095) | 0.43 (0.19 to 0.74) | 0.067 (0.034 to 0.115) | 0.44 (0.22 to 0.75) | 0.23 (-0.06 to 0.65) | -0.17 (-0.29 to -0.04) |
| Belarus | Acute myeloid leukemia | death | 0.022 (0.01 to 0.039) | 0.17 (0.08 to 0.3) | 0.028 (0.013 to 0.049) | 0.18 (0.09 to 0.32) | 0.24 (-0.09 to 0.76) | 0.03 (-0.09 to 0.14) |
| Belarus | Acute lymphoid leukemia | death | 0.004 (0.002 to 0.008) | 0.03 (0.01 to 0.06) | 0.004 (0.002 to 0.007) | 0.03 (0.01 to 0.05) | -0.02 (-0.33 to 0.47) | -0.83 (-0.96 to -0.71) |
| Belarus | Chronic myeloid leukemia | death | 0.014 (0.006 to 0.026) | 0.11 (0.05 to 0.2) | 0.015 (0.007 to 0.027) | 0.1 (0.05 to 0.18) | 0.1 (-0.21 to 0.58) | -0.66 (-1.02 to -0.3) |
| Belarus | Chronic lymphoid leukemia | death | 0.007 (0.003 to 0.013) | 0.05 (0.02 to 0.1) | 0.012 (0.006 to 0.021) | 0.08 (0.03 to 0.13) | 0.75 (0.17 to 1.93) | 1.19 (1.07 to 1.3) |
| Belarus | Other leukemia | death | 0.007 (0.003 to 0.014) | 0.06 (0.02 to 0.11) | 0.008 (0.004 to 0.014) | 0.05 (0.02 to 0.09) | 0.06 (-0.26 to 0.65) | -0.95 (-1.18 to -0.72) |
| Belarus | Leukemia | DALYs | 1.617 (0.727 to 2.819) | 12.9 (5.77 to 22.53) | 1.728 (0.87 to 2.977) | 11.93 (6.01 to 20.39) | 0.07 (-0.19 to 0.46) | -0.64 (-0.78 to -0.5) |
| Belarus | Acute myeloid leukemia | DALYs | 0.669 (0.294 to 1.166) | 5.31 (2.31 to 9.25) | 0.73 (0.356 to 1.273) | 5.07 (2.45 to 8.91) | 0.09 (-0.2 to 0.57) | -0.4 (-0.53 to -0.28) |
| Belarus | Acute lymphoid leukemia | DALYs | 0.152 (0.064 to 0.29) | 1.28 (0.52 to 2.47) | 0.135 (0.066 to 0.244) | 1.07 (0.52 to 1.95) | -0.11 (-0.41 to 0.43) | -1.11 (-1.3 to -0.91) |
| Belarus | Chronic myeloid leukemia | DALYs | 0.411 (0.179 to 0.751) | 3.31 (1.45 to 6.02) | 0.391 (0.186 to 0.704) | 2.73 (1.31 to 4.87) | -0.05 (-0.32 to 0.38) | -1.17 (-1.56 to -0.78) |
| Belarus | Chronic lymphoid leukemia | DALYs | 0.182 (0.071 to 0.351) | 1.38 (0.54 to 2.67) | 0.294 (0.135 to 0.522) | 1.86 (0.86 to 3.29) | 0.61 (0.07 to 1.7) | 0.93 (0.82 to 1.03) |
| Belarus | Other leukemia | DALYs | 0.204 (0.088 to 0.366) | 1.62 (0.69 to 2.9) | 0.178 (0.085 to 0.319) | 1.19 (0.57 to 2.13) | -0.12 (-0.39 to 0.37) | -1.67 (-1.96 to -1.38) |
| Belgium | Leukemia | death | 0.064 (0.028 to 0.113) | 0.42 (0.18 to 0.74) | 0.099 (0.045 to 0.172) | 0.41 (0.19 to 0.7) | 0.54 (0.32 to 0.88) | -0.17 (-0.43 to 0.1) |
| Belgium | Acute myeloid leukemia | death | 0.015 (0.006 to 0.028) | 0.1 (0.04 to 0.18) | 0.039 (0.018 to 0.069) | 0.17 (0.08 to 0.3) | 1.61 (0.79 to 2.43) | 1.68 (1.47 to 1.89) |
| Belgium | Acute lymphoid leukemia | death | 0.003 (0.001 to 0.006) | 0.02 (0.01 to 0.04) | 0.004 (0.002 to 0.006) | 0.02 (0.01 to 0.03) | 0.17 (-0.06 to 0.75) | -0.98 (-1.24 to -0.72) |
| Belgium | Chronic myeloid leukemia | death | 0.006 (0.003 to 0.012) | 0.04 (0.02 to 0.08) | 0.005 (0.002 to 0.009) | 0.02 (0.01 to 0.04) | -0.19 (-0.37 to 0.07) | -3.9 (-4.5 to -3.29) |
| Belgium | Chronic lymphoid leukemia | death | 0.009 (0.004 to 0.017) | 0.06 (0.03 to 0.11) | 0.017 (0.008 to 0.03) | 0.06 (0.03 to 0.11) | 0.81 (0.44 to 1.4) | -0.3 (-0.59 to -0.01) |
| Belgium | Other leukemia | death | 0.03 (0.013 to 0.054) | 0.2 (0.08 to 0.35) | 0.034 (0.016 to 0.06) | 0.13 (0.06 to 0.24) | 0.12 (-0.08 to 0.43) | -0.83 (-1.43 to -0.22) |
| Belgium | Leukemia | DALYs | 1.367 (0.597 to 2.411) | 9.57 (4.19 to 16.89) | 1.826 (0.862 to 3.118) | 9.06 (4.36 to 15.37) | 0.34 (0.17 to 0.6) | -0.31 (-0.48 to -0.15) |
| Belgium | Acute myeloid leukemia | DALYs | 0.348 (0.15 to 0.63) | 2.5 (1.07 to 4.51) | 0.773 (0.361 to 1.346) | 4.01 (1.9 to 6.86) | 1.22 (0.58 to 1.87) | 1.35 (1.15 to 1.55) |
| Belgium | Acute lymphoid leukemia | DALYs | 0.095 (0.041 to 0.176) | 0.74 (0.32 to 1.37) | 0.119 (0.057 to 0.204) | 0.74 (0.36 to 1.29) | 0.25 (-0.03 to 0.88) | -0.35 (-0.51 to -0.18) |
| Belgium | Chronic myeloid leukemia | DALYs | 0.136 (0.059 to 0.245) | 0.96 (0.42 to 1.74) | 0.101 (0.047 to 0.176) | 0.5 (0.23 to 0.85) | -0.25 (-0.4 to -0.04) | -3.68 (-4.23 to -3.12) |
| Belgium | Chronic lymphoid leukemia | DALYs | 0.167 (0.073 to 0.294) | 1.07 (0.47 to 1.87) | 0.273 (0.124 to 0.477) | 1.16 (0.53 to 2.03) | 0.63 (0.31 to 1.11) | -0.25 (-0.53 to 0.04) |
| Belgium | Other leukemia | DALYs | 0.621 (0.265 to 1.108) | 4.3 (1.85 to 7.71) | 0.56 (0.265 to 0.985) | 2.65 (1.27 to 4.58) | -0.1 (-0.24 to 0.14) | -1.24 (-1.76 to -0.72) |
| Belize | Leukemia | death | 0 (0 to 0) | 0.16 (0.06 to 0.3) | 0.001 (0 to 0.002) | 0.31 (0.16 to 0.51) | 4.9 (3.49 to 7.89) | 2.02 (1.45 to 2.6) |
| Belize | Acute myeloid leukemia | death | 0 (0 to 0) | 0.01 (0 to 0.02) | 0 (0 to 0) | 0.02 (0.01 to 0.04) | 6.85 (4.49 to 11.37) | 2.98 (2.6 to 3.37) |
| Belize | Acute lymphoid leukemia | death | 0 (0 to 0) | 0.01 (0 to 0.02) | 0 (0 to 0) | 0.02 (0.01 to 0.04) | 5.42 (3.5 to 9.92) | 2.52 (2.16 to 2.87) |
| Belize | Chronic myeloid leukemia | death | 0 (0 to 0) | 0.01 (0 to 0.02) | 0 (0 to 0) | 0.02 (0.01 to 0.02) | 4.58 (2.92 to 7.97) | 1.66 (1.06 to 2.27) |
| Belize | Chronic lymphoid leukemia | death | 0 (0 to 0) | 0.01 (0 to 0.02) | 0 (0 to 0) | 0.02 (0.01 to 0.04) | 6.86 (4.04 to 13.24) | 3.62 (3.04 to 4.19) |
| Belize | Other leukemia | death | 0 (0 to 0) | 0.12 (0.05 to 0.23) | 0.001 (0 to 0.001) | 0.22 (0.11 to 0.37) | 4.57 (3.26 to 7.31) | 1.78 (1.17 to 2.41) |
| Belize | Leukemia | DALYs | 0.006 (0.002 to 0.01) | 4.92 (1.9 to 8.88) | 0.036 (0.019 to 0.058) | 10.04 (5.23 to 16.29) | 5.47 (3.87 to 8.89) | 2.13 (1.54 to 2.72) |
| Belize | Acute myeloid leukemia | DALYs | 0 (0 to 0.001) | 0.36 (0.13 to 0.68) | 0.003 (0.002 to 0.006) | 0.92 (0.46 to 1.52) | 7.16 (4.51 to 12.21) | 3.05 (2.65 to 3.46) |
| Belize | Acute lymphoid leukemia | DALYs | 0.001 (0 to 0.001) | 0.43 (0.15 to 0.83) | 0.004 (0.002 to 0.006) | 0.96 (0.49 to 1.6) | 5.6 (3.47 to 10.87) | 2.61 (2.23 to 3) |
| Belize | Chronic myeloid leukemia | DALYs | 0 (0 to 0.001) | 0.26 (0.1 to 0.49) | 0.002 (0.001 to 0.003) | 0.5 (0.26 to 0.82) | 5.18 (3.31 to 8.99) | 1.82 (1.19 to 2.44) |
| Belize | Chronic lymphoid leukemia | DALYs | 0 (0 to 0) | 0.21 (0.07 to 0.41) | 0.002 (0.001 to 0.003) | 0.56 (0.29 to 0.92) | 7.33 (4.36 to 14.08) | 3.6 (3.05 to 4.16) |
| Belize | Other leukemia | DALYs | 0.004 (0.002 to 0.007) | 3.67 (1.4 to 6.63) | 0.025 (0.013 to 0.041) | 7.11 (3.67 to 11.59) | 5.2 (3.69 to 8.21) | 1.9 (1.26 to 2.54) |
| Benin | Leukemia | death | 0.002 (0.001 to 0.004) | 0.1 (0.04 to 0.2) | 0.012 (0.005 to 0.021) | 0.2 (0.09 to 0.38) | 4.38 (2.7 to 8.38) | 2.72 (2.47 to 2.97) |
| Benin | Acute myeloid leukemia | death | 0 (0 to 0.001) | 0.01 (0 to 0.03) | 0.002 (0.001 to 0.004) | 0.03 (0.01 to 0.07) | 5.5 (3.12 to 11.49) | 3.35 (3.12 to 3.59) |
| Benin | Acute lymphoid leukemia | death | 0 (0 to 0) | 0 (0 to 0.01) | 0.001 (0 to 0.002) | 0.01 (0 to 0.02) | 5.18 (2.68 to 11.1) | 2.65 (2.43 to 2.87) |
| Benin | Chronic myeloid leukemia | death | 0 (0 to 0.001) | 0.02 (0.01 to 0.04) | 0.002 (0.001 to 0.004) | 0.03 (0.01 to 0.06) | 3.9 (2.12 to 7.82) | 2.09 (1.87 to 2.31) |
| Benin | Chronic lymphoid leukemia | death | 0 (0 to 0.001) | 0.02 (0.01 to 0.04) | 0.002 (0.001 to 0.004) | 0.04 (0.02 to 0.08) | 3.95 (2.2 to 8.38) | 2.81 (2.57 to 3.05) |
| Benin | Other leukemia | death | 0.001 (0 to 0.002) | 0.04 (0.01 to 0.09) | 0.005 (0.002 to 0.008) | 0.08 (0.04 to 0.16) | 4.27 (2.58 to 8.49) | 2.71 (2.43 to 3) |
| Benin | Leukemia | DALYs | 0.072 (0.027 to 0.146) | 3.02 (1.11 to 5.92) | 0.409 (0.193 to 0.76) | 6.17 (2.9 to 11.42) | 4.65 (2.83 to 8.98) | 2.65 (2.38 to 2.91) |
| Benin | Acute myeloid leukemia | DALYs | 0.012 (0.004 to 0.026) | 0.47 (0.16 to 1) | 0.081 (0.036 to 0.158) | 1.14 (0.49 to 2.19) | 5.64 (3.13 to 11.86) | 3.2 (2.95 to 3.44) |
| Benin | Acute lymphoid leukemia | DALYs | 0.006 (0.002 to 0.013) | 0.19 (0.06 to 0.41) | 0.037 (0.014 to 0.077) | 0.42 (0.16 to 0.83) | 5.57 (2.8 to 12.42) | 2.8 (2.55 to 3.05) |
| Benin | Chronic myeloid leukemia | DALYs | 0.017 (0.006 to 0.036) | 0.66 (0.22 to 1.4) | 0.086 (0.037 to 0.165) | 1.14 (0.5 to 2.17) | 4.1 (2.22 to 8.17) | 2.07 (1.84 to 2.31) |
| Benin | Chronic lymphoid leukemia | DALYs | 0.01 (0.003 to 0.021) | 0.49 (0.15 to 1.03) | 0.048 (0.018 to 0.093) | 0.99 (0.38 to 1.88) | 3.92 (2.08 to 8.18) | 2.62 (2.39 to 2.85) |
| Benin | Other leukemia | DALYs | 0.028 (0.01 to 0.058) | 1.21 (0.43 to 2.52) | 0.157 (0.068 to 0.286) | 2.48 (1.09 to 4.47) | 4.63 (2.76 to 9.21) | 2.69 (2.38 to 2.99) |
| Bermuda | Leukemia | death | 0 (0 to 0.001) | 0.52 (0.25 to 0.84) | 0 (0 to 0.001) | 0.35 (0.18 to 0.57) | 0.33 (0.08 to 0.69) | -1.35 (-1.52 to -1.19) |
| Bermuda | Acute myeloid leukemia | death | 0 (0 to 0) | 0.14 (0.07 to 0.22) | 0 (0 to 0) | 0.13 (0.07 to 0.22) | 0.72 (0.32 to 1.3) | -0.07 (-0.23 to 0.09) |
| Bermuda | Acute lymphoid leukemia | death | 0 (0 to 0) | 0.02 (0.01 to 0.04) | 0 (0 to 0) | 0.02 (0.01 to 0.03) | 0.26 (-0.12 to 0.77) | -1.28 (-1.4 to -1.15) |
| Bermuda | Chronic myeloid leukemia | death | 0 (0 to 0) | 0.05 (0.02 to 0.08) | 0 (0 to 0) | 0.03 (0.01 to 0.04) | 0.06 (-0.22 to 0.52) | -1.66 (-1.94 to -1.38) |
| Bermuda | Chronic lymphoid leukemia | death | 0 (0 to 0) | 0.04 (0.02 to 0.07) | 0 (0 to 0) | 0.04 (0.02 to 0.06) | 0.96 (0.34 to 1.92) | -0.15 (-0.31 to 0) |
| Bermuda | Other leukemia | death | 0 (0 to 0) | 0.28 (0.13 to 0.46) | 0 (0 to 0) | 0.14 (0.07 to 0.24) | 0.09 (-0.13 to 0.42) | -2.41 (-2.58 to -2.24) |
| Bermuda | Leukemia | DALYs | 0.01 (0.005 to 0.015) | 14.45 (7.14 to 23.48) | 0.01 (0.005 to 0.016) | 9.66 (5.01 to 15.63) | 0.06 (-0.15 to 0.36) | -1.43 (-1.62 to -1.24) |
| Bermuda | Acute myeloid leukemia | DALYs | 0.003 (0.001 to 0.005) | 4.31 (2.11 to 7.09) | 0.004 (0.002 to 0.006) | 4.05 (2.07 to 6.61) | 0.37 (0.03 to 0.84) | -0.18 (-0.34 to -0.01) |
| Bermuda | Acute lymphoid leukemia | DALYs | 0 (0 to 0.001) | 0.73 (0.34 to 1.27) | 0.001 (0 to 0.001) | 0.59 (0.29 to 0.98) | 0.09 (-0.25 to 0.56) | -0.93 (-1.05 to -0.8) |
| Bermuda | Chronic myeloid leukemia | DALYs | 0.001 (0 to 0.002) | 1.4 (0.65 to 2.38) | 0.001 (0 to 0.001) | 0.77 (0.37 to 1.27) | -0.14 (-0.37 to 0.19) | -1.84 (-2.15 to -1.53) |
| Bermuda | Chronic lymphoid leukemia | DALYs | 0.001 (0 to 0.001) | 0.95 (0.43 to 1.68) | 0.001 (0 to 0.002) | 0.86 (0.38 to 1.43) | 0.74 (0.18 to 1.64) | -0.2 (-0.36 to -0.04) |
| Bermuda | Other leukemia | DALYs | 0.005 (0.002 to 0.008) | 7.06 (3.4 to 11.64) | 0.004 (0.002 to 0.006) | 3.39 (1.74 to 5.45) | -0.18 (-0.35 to 0.08) | -2.73 (-2.93 to -2.53) |
| Bhutan | Leukemia | death | 0 (0 to 0.001) | 0.07 (0.02 to 0.18) | 0.001 (0 to 0.002) | 0.16 (0.06 to 0.31) | 3.39 (1.67 to 8.9) | 2.72 (2.59 to 2.86) |
| Bhutan | Acute myeloid leukemia | death | 0 (0 to 0) | 0.01 (0 to 0.03) | 0 (0 to 0.001) | 0.04 (0.02 to 0.09) | 5.39 (2.37 to 14.63) | 4.17 (3.93 to 4.42) |
| Bhutan | Acute lymphoid leukemia | death | 0 (0 to 0) | 0 (0 to 0.01) | 0 (0 to 0) | 0.01 (0 to 0.02) | 3.19 (1.13 to 10.62) | 3 (2.8 to 3.2) |
| Bhutan | Chronic myeloid leukemia | death | 0 (0 to 0) | 0.02 (0.01 to 0.06) | 0 (0 to 0) | 0.03 (0.01 to 0.07) | 1.74 (0.48 to 6.04) | 0.99 (0.86 to 1.12) |
| Bhutan | Chronic lymphoid leukemia | death | 0 (0 to 0) | 0.02 (0 to 0.04) | 0 (0 to 0.001) | 0.05 (0.02 to 0.1) | 5.81 (3.17 to 14.28) | 3.85 (3.71 to 3.98) |
| Bhutan | Other leukemia | death | 0 (0 to 0) | 0.01 (0 to 0.04) | 0 (0 to 0) | 0.02 (0.01 to 0.05) | 2.48 (1.04 to 8.59) | 1.92 (1.78 to 2.07) |
| Bhutan | Leukemia | DALYs | 0.008 (0.002 to 0.019) | 2.24 (0.52 to 5.42) | 0.029 (0.011 to 0.058) | 4.43 (1.66 to 8.73) | 2.75 (1.19 to 8.14) | 2.38 (2.24 to 2.53) |
| Bhutan | Acute myeloid leukemia | DALYs | 0.002 (0 to 0.004) | 0.43 (0.09 to 1.08) | 0.009 (0.003 to 0.019) | 1.3 (0.48 to 2.72) | 4.86 (1.95 to 13.83) | 4.1 (3.84 to 4.35) |
| Bhutan | Acute lymphoid leukemia | DALYs | 0.001 (0 to 0.002) | 0.14 (0.03 to 0.38) | 0.002 (0.001 to 0.005) | 0.3 (0.11 to 0.63) | 2.85 (0.85 to 10.36) | 2.89 (2.69 to 3.08) |
| Bhutan | Chronic myeloid leukemia | DALYs | 0.003 (0.001 to 0.008) | 0.85 (0.19 to 2.13) | 0.008 (0.003 to 0.015) | 1.1 (0.38 to 2.26) | 1.4 (0.23 to 5.61) | 0.63 (0.48 to 0.78) |
| Bhutan | Chronic lymphoid leukemia | DALYs | 0.001 (0 to 0.002) | 0.39 (0.08 to 0.99) | 0.006 (0.002 to 0.012) | 1.05 (0.37 to 2.14) | 4.94 (2.56 to 12.72) | 3.65 (3.5 to 3.81) |
| Bhutan | Other leukemia | DALYs | 0.001 (0 to 0.004) | 0.44 (0.08 to 1.19) | 0.004 (0.002 to 0.009) | 0.68 (0.24 to 1.37) | 1.93 (0.61 to 7.75) | 1.56 (1.41 to 1.71) |
| Bolivia (Plurinational State of) | Leukemia | death | 0.011 (0.004 to 0.022) | 0.31 (0.12 to 0.61) | 0.05 (0.024 to 0.09) | 0.55 (0.26 to 0.97) | 3.47 (2.03 to 6.08) | 1.93 (1.86 to 2) |
| Bolivia (Plurinational State of) | Acute myeloid leukemia | death | 0.002 (0.001 to 0.003) | 0.04 (0.02 to 0.09) | 0.011 (0.005 to 0.019) | 0.12 (0.05 to 0.21) | 5.99 (3.38 to 11.02) | 3.54 (3.36 to 3.73) |
| Bolivia (Plurinational State of) | Acute lymphoid leukemia | death | 0.002 (0.001 to 0.003) | 0.04 (0.01 to 0.09) | 0.008 (0.004 to 0.015) | 0.09 (0.04 to 0.16) | 4.47 (2.27 to 8.89) | 2.74 (2.55 to 2.92) |
| Bolivia (Plurinational State of) | Chronic myeloid leukemia | death | 0.001 (0 to 0.002) | 0.03 (0.01 to 0.07) | 0.005 (0.002 to 0.009) | 0.06 (0.02 to 0.1) | 3.18 (1.7 to 6.32) | 1.79 (1.75 to 1.82) |
| Bolivia (Plurinational State of) | Chronic lymphoid leukemia | death | 0 (0 to 0.001) | 0.01 (0 to 0.02) | 0.003 (0.001 to 0.005) | 0.03 (0.01 to 0.06) | 7.43 (4.39 to 13.14) | 4.09 (3.98 to 4.2) |
| Bolivia (Plurinational State of) | Other leukemia | death | 0.007 (0.002 to 0.014) | 0.19 (0.07 to 0.38) | 0.024 (0.01 to 0.044) | 0.26 (0.12 to 0.48) | 2.53 (1.39 to 4.68) | 1.06 (1 to 1.12) |
| Bolivia (Plurinational State of) | Leukemia | DALYs | 0.399 (0.153 to 0.785) | 9.8 (3.84 to 18.84) | 1.56 (0.713 to 2.815) | 15.61 (7.19 to 28.14) | 2.91 (1.54 to 5.42) | 1.53 (1.44 to 1.61) |
| Bolivia (Plurinational State of) | Acute myeloid leukemia | DALYs | 0.055 (0.02 to 0.114) | 1.34 (0.5 to 2.76) | 0.35 (0.16 to 0.635) | 3.45 (1.58 to 6.24) | 5.39 (2.9 to 10.44) | 3.33 (3.13 to 3.52) |
| Bolivia (Plurinational State of) | Acute lymphoid leukemia | DALYs | 0.058 (0.019 to 0.135) | 1.35 (0.46 to 3.06) | 0.286 (0.124 to 0.541) | 2.75 (1.21 to 5.17) | 3.94 (1.85 to 8.39) | 2.46 (2.27 to 2.66) |
| Bolivia (Plurinational State of) | Chronic myeloid leukemia | DALYs | 0.041 (0.015 to 0.085) | 1.03 (0.38 to 2.1) | 0.148 (0.067 to 0.272) | 1.5 (0.68 to 2.77) | 2.58 (1.28 to 5.51) | 1.27 (1.23 to 1.32) |
| Bolivia (Plurinational State of) | Chronic lymphoid leukemia | DALYs | 0.009 (0.003 to 0.018) | 0.25 (0.1 to 0.51) | 0.065 (0.029 to 0.114) | 0.71 (0.32 to 1.24) | 6.25 (3.59 to 11) | 3.57 (3.45 to 3.68) |
| Bolivia (Plurinational State of) | Other leukemia | DALYs | 0.236 (0.084 to 0.495) | 5.83 (2.12 to 11.79) | 0.711 (0.306 to 1.342) | 7.2 (3.11 to 13.46) | 2.01 (0.95 to 3.97) | 0.57 (0.49 to 0.64) |
| Bosnia and Herzegovina | Leukemia | death | 0.009 (0.004 to 0.016) | 0.21 (0.09 to 0.39) | 0.022 (0.011 to 0.038) | 0.38 (0.19 to 0.67) | 1.41 (0.74 to 2.46) | 2.39 (2.1 to 2.69) |
| Bosnia and Herzegovina | Acute myeloid leukemia | death | 0.003 (0.001 to 0.006) | 0.07 (0.03 to 0.13) | 0.008 (0.004 to 0.014) | 0.14 (0.06 to 0.24) | 1.55 (0.49 to 3.08) | 2.86 (2.59 to 3.14) |
| Bosnia and Herzegovina | Acute lymphoid leukemia | death | 0.001 (0 to 0.002) | 0.03 (0.01 to 0.05) | 0.002 (0.001 to 0.004) | 0.04 (0.02 to 0.07) | 0.59 (0.04 to 1.51) | 1.25 (1.1 to 1.4) |
| Bosnia and Herzegovina | Chronic myeloid leukemia | death | 0.001 (0 to 0.002) | 0.03 (0.01 to 0.05) | 0.002 (0.001 to 0.004) | 0.04 (0.02 to 0.07) | 0.96 (0.28 to 2.13) | 1.82 (1.32 to 2.31) |
| Bosnia and Herzegovina | Chronic lymphoid leukemia | death | 0.001 (0.001 to 0.002) | 0.03 (0.01 to 0.06) | 0.007 (0.003 to 0.012) | 0.11 (0.06 to 0.2) | 4.57 (2.78 to 7.34) | 5.24 (4.63 to 5.85) |
| Bosnia and Herzegovina | Other leukemia | death | 0.002 (0.001 to 0.005) | 0.06 (0.02 to 0.13) | 0.003 (0.002 to 0.006) | 0.05 (0.03 to 0.11) | 0.29 (-0.12 to 1.08) | -0.38 (-0.57 to -0.18) |
| Bosnia and Herzegovina | Leukemia | DALYs | 0.281 (0.118 to 0.496) | 6.14 (2.6 to 10.88) | 0.543 (0.272 to 0.943) | 10.07 (4.99 to 17.47) | 0.93 (0.4 to 1.84) | 2.01 (1.78 to 2.25) |
| Bosnia and Herzegovina | Acute myeloid leukemia | DALYs | 0.099 (0.04 to 0.185) | 2.1 (0.84 to 3.97) | 0.206 (0.097 to 0.368) | 3.98 (1.82 to 7.14) | 1.08 (0.3 to 2.3) | 2.56 (2.33 to 2.8) |
| Bosnia and Herzegovina | Acute lymphoid leukemia | DALYs | 0.046 (0.018 to 0.091) | 0.95 (0.37 to 1.89) | 0.062 (0.028 to 0.116) | 1.34 (0.6 to 2.51) | 0.34 (-0.11 to 1.16) | 1.2 (1.08 to 1.32) |
| Bosnia and Herzegovina | Chronic myeloid leukemia | DALYs | 0.033 (0.013 to 0.065) | 0.72 (0.29 to 1.4) | 0.051 (0.024 to 0.092) | 0.94 (0.44 to 1.71) | 0.52 (0 to 1.51) | 1.28 (0.86 to 1.71) |
| Bosnia and Herzegovina | Chronic lymphoid leukemia | DALYs | 0.031 (0.013 to 0.058) | 0.74 (0.31 to 1.38) | 0.15 (0.074 to 0.26) | 2.47 (1.22 to 4.26) | 3.78 (2.21 to 6.21) | 4.94 (4.38 to 5.51) |
| Bosnia and Herzegovina | Other leukemia | DALYs | 0.071 (0.027 to 0.147) | 1.62 (0.62 to 3.32) | 0.074 (0.035 to 0.138) | 1.35 (0.63 to 2.55) | 0.04 (-0.3 to 0.72) | -0.71 (-0.92 to -0.5) |
| Botswana | Leukemia | death | 0.001 (0 to 0.002) | 0.14 (0.05 to 0.29) | 0.005 (0.002 to 0.009) | 0.36 (0.17 to 0.63) | 5.07 (2.46 to 10.7) | 3.16 (2.86 to 3.46) |
| Botswana | Acute myeloid leukemia | death | 0 (0 to 0) | 0.01 (0 to 0.02) | 0 (0 to 0.001) | 0.02 (0.01 to 0.04) | 7.16 (3.3 to 16.3) | 3.57 (3.39 to 3.76) |
| Botswana | Acute lymphoid leukemia | death | 0 (0 to 0) | 0 (0 to 0.01) | 0 (0 to 0) | 0.01 (0 to 0.02) | 6.38 (3.1 to 15.39) | 3.03 (2.7 to 3.36) |
| Botswana | Chronic myeloid leukemia | death | 0 (0 to 0) | 0 (0 to 0.01) | 0 (0 to 0) | 0.01 (0 to 0.02) | 4.45 (2.06 to 10.42) | 1.92 (1.44 to 2.4) |
| Botswana | Chronic lymphoid leukemia | death | 0 (0 to 0.001) | 0.08 (0.03 to 0.16) | 0.003 (0.001 to 0.005) | 0.23 (0.09 to 0.43) | 5.66 (2.72 to 11.68) | 3.83 (3.46 to 4.2) |
| Botswana | Other leukemia | death | 0 (0 to 0.001) | 0.05 (0.02 to 0.1) | 0.001 (0.001 to 0.003) | 0.09 (0.04 to 0.16) | 3.82 (1.81 to 8.75) | 1.87 (1.61 to 2.13) |
| Botswana | Leukemia | DALYs | 0.026 (0.009 to 0.055) | 3.86 (1.37 to 7.86) | 0.166 (0.077 to 0.304) | 9.53 (4.48 to 17.13) | 5.27 (2.47 to 11.26) | 3.01 (2.72 to 3.29) |
| Botswana | Acute myeloid leukemia | DALYs | 0.002 (0.001 to 0.006) | 0.3 (0.1 to 0.66) | 0.022 (0.009 to 0.042) | 0.95 (0.42 to 1.82) | 7.66 (3.36 to 18.42) | 3.79 (3.61 to 3.97) |
| Botswana | Acute lymphoid leukemia | DALYs | 0.001 (0 to 0.003) | 0.15 (0.04 to 0.34) | 0.01 (0.004 to 0.021) | 0.44 (0.18 to 0.84) | 6.73 (3.24 to 16.95) | 3.33 (3.06 to 3.6) |
| Botswana | Chronic myeloid leukemia | DALYs | 0.001 (0 to 0.003) | 0.16 (0.05 to 0.36) | 0.007 (0.003 to 0.013) | 0.35 (0.16 to 0.63) | 4.8 (2.16 to 12.07) | 2.06 (1.6 to 2.53) |
| Botswana | Chronic lymphoid leukemia | DALYs | 0.011 (0.004 to 0.022) | 1.8 (0.64 to 3.63) | 0.072 (0.029 to 0.136) | 4.98 (2.01 to 9.37) | 5.68 (2.58 to 12.04) | 3.59 (3.24 to 3.94) |
| Botswana | Other leukemia | DALYs | 0.011 (0.003 to 0.023) | 1.45 (0.47 to 3.1) | 0.055 (0.024 to 0.098) | 2.82 (1.27 to 4.98) | 4.15 (1.94 to 10.03) | 2 (1.73 to 2.26) |
| Brazil | Leukemia | death | 0.221 (0.091 to 0.401) | 0.23 (0.1 to 0.42) | 0.752 (0.374 to 1.214) | 0.32 (0.16 to 0.52) | 2.4 (1.87 to 3.44) | 1.29 (1.17 to 1.41) |
| Brazil | Acute myeloid leukemia | death | 0.072 (0.029 to 0.131) | 0.07 (0.03 to 0.13) | 0.306 (0.152 to 0.503) | 0.13 (0.06 to 0.21) | 3.25 (2.56 to 4.59) | 2.26 (2.12 to 2.4) |
| Brazil | Acute lymphoid leukemia | death | 0.021 (0.008 to 0.039) | 0.02 (0.01 to 0.03) | 0.075 (0.038 to 0.122) | 0.03 (0.02 to 0.05) | 2.64 (1.84 to 4.06) | 2.02 (1.86 to 2.18) |
| Brazil | Chronic myeloid leukemia | death | 0.038 (0.016 to 0.068) | 0.04 (0.02 to 0.07) | 0.059 (0.03 to 0.096) | 0.03 (0.01 to 0.04) | 0.58 (0.32 to 1.07) | -1.85 (-2.3 to -1.41) |
| Brazil | Chronic lymphoid leukemia | death | 0.016 (0.007 to 0.03) | 0.02 (0.01 to 0.04) | 0.081 (0.04 to 0.133) | 0.04 (0.02 to 0.06) | 3.9 (3.06 to 5.32) | 2.11 (1.94 to 2.28) |
| Brazil | Other leukemia | death | 0.075 (0.03 to 0.135) | 0.08 (0.03 to 0.15) | 0.231 (0.116 to 0.38) | 0.1 (0.05 to 0.17) | 2.09 (1.57 to 3.07) | 0.99 (0.89 to 1.1) |
| Brazil | Leukemia | DALYs | 7.606 (3.15 to 13.816) | 6.82 (2.83 to 12.35) | 21.334 (10.79 to 34.426) | 8.87 (4.48 to 14.35) | 1.81 (1.35 to 2.74) | 0.99 (0.89 to 1.09) |
| Brazil | Acute myeloid leukemia | DALYs | 2.615 (1.052 to 4.792) | 2.27 (0.93 to 4.12) | 9.313 (4.653 to 15.157) | 3.84 (1.91 to 6.26) | 2.56 (1.95 to 3.81) | 1.99 (1.87 to 2.1) |
| Brazil | Acute lymphoid leukemia | DALYs | 0.863 (0.338 to 1.649) | 0.68 (0.27 to 1.28) | 2.892 (1.493 to 4.693) | 1.2 (0.62 to 1.95) | 2.35 (1.55 to 3.94) | 2.19 (2.03 to 2.34) |
| Brazil | Chronic myeloid leukemia | DALYs | 1.308 (0.548 to 2.369) | 1.18 (0.5 to 2.12) | 1.576 (0.817 to 2.566) | 0.65 (0.34 to 1.07) | 0.21 (0 to 0.61) | -2.56 (-3.03 to -2.09) |
| Brazil | Chronic lymphoid leukemia | DALYs | 0.39 (0.169 to 0.7) | 0.44 (0.19 to 0.78) | 1.623 (0.828 to 2.661) | 0.69 (0.35 to 1.13) | 3.16 (2.49 to 4.36) | 1.73 (1.57 to 1.89) |
| Brazil | Other leukemia | DALYs | 2.429 (0.998 to 4.409) | 2.26 (0.92 to 4.08) | 5.93 (3.039 to 9.737) | 2.48 (1.27 to 4.07) | 1.44 (1.02 to 2.27) | 0.55 (0.46 to 0.65) |
| Brunei Darussalam | Leukemia | death | 0 (0 to 0) | 0.15 (0.04 to 0.37) | 0.001 (0 to 0.002) | 0.24 (0.1 to 0.47) | 2.98 (1.69 to 7.14) | 2.21 (2.01 to 2.41) |
| Brunei Darussalam | Acute myeloid leukemia | death | 0 (0 to 0) | 0.03 (0.01 to 0.06) | 0 (0 to 0) | 0.04 (0.02 to 0.08) | 3.22 (1.76 to 8.29) | 2.08 (1.82 to 2.33) |
| Brunei Darussalam | Acute lymphoid leukemia | death | 0 (0 to 0) | 0 (0 to 0.01) | 0 (0 to 0) | 0.01 (0 to 0.02) | 5.78 (1.82 to 19.12) | 4.79 (4.46 to 5.11) |
| Brunei Darussalam | Chronic myeloid leukemia | death | 0 (0 to 0) | 0.05 (0.01 to 0.14) | 0 (0 to 0) | 0.07 (0.03 to 0.13) | 1.89 (0.82 to 5.52) | 1.5 (1.1 to 1.91) |
| Brunei Darussalam | Chronic lymphoid leukemia | death | 0 (0 to 0) | 0.05 (0.01 to 0.13) | 0 (0 to 0.001) | 0.1 (0.03 to 0.2) | 4.66 (2.69 to 10.44) | 2.71 (2.55 to 2.88) |
| Brunei Darussalam | Other leukemia | death | 0 (0 to 0) | 0.02 (0 to 0.04) | 0 (0 to 0) | 0.03 (0.01 to 0.05) | 2.59 (1.31 to 7.11) | 2.06 (1.84 to 2.28) |
| Brunei Darussalam | Leukemia | DALYs | 0.008 (0.002 to 0.021) | 4.73 (1.08 to 11.4) | 0.029 (0.012 to 0.057) | 7.08 (2.82 to 13.61) | 2.5 (1.32 to 6.34) | 1.98 (1.74 to 2.21) |
| Brunei Darussalam | Acute myeloid leukemia | DALYs | 0.002 (0 to 0.005) | 0.94 (0.2 to 2.37) | 0.008 (0.003 to 0.015) | 1.58 (0.6 to 3.16) | 2.84 (1.45 to 7.64) | 2.12 (1.92 to 2.33) |
| Brunei Darussalam | Acute lymphoid leukemia | DALYs | 0 (0 to 0.001) | 0.12 (0.02 to 0.38) | 0.002 (0.001 to 0.004) | 0.38 (0.13 to 0.83) | 5.07 (1.57 to 17.85) | 4.63 (4.3 to 4.96) |
| Brunei Darussalam | Chronic myeloid leukemia | DALYs | 0.004 (0.001 to 0.01) | 1.87 (0.4 to 4.77) | 0.009 (0.004 to 0.018) | 2.09 (0.83 to 3.99) | 1.53 (0.58 to 4.91) | 1.04 (0.63 to 1.46) |
| Brunei Darussalam | Chronic lymphoid leukemia | DALYs | 0.001 (0 to 0.003) | 1.2 (0.27 to 2.9) | 0.007 (0.002 to 0.014) | 2.19 (0.75 to 4.38) | 4.59 (2.7 to 10.48) | 2.71 (2.52 to 2.9) |
| Brunei Darussalam | Other leukemia | DALYs | 0.001 (0 to 0.003) | 0.59 (0.12 to 1.49) | 0.004 (0.001 to 0.007) | 0.83 (0.32 to 1.64) | 2.07 (0.93 to 6.22) | 1.71 (1.49 to 1.93) |
| Bulgaria | Leukemia | death | 0.043 (0.022 to 0.071) | 0.36 (0.18 to 0.58) | 0.058 (0.029 to 0.098) | 0.44 (0.22 to 0.74) | 0.34 (0.06 to 0.7) | 1.13 (0.83 to 1.43) |
| Bulgaria | Acute myeloid leukemia | death | 0.014 (0.007 to 0.023) | 0.11 (0.05 to 0.19) | 0.021 (0.01 to 0.036) | 0.16 (0.08 to 0.28) | 0.49 (0.1 to 1) | 1.82 (1.56 to 2.09) |
| Bulgaria | Acute lymphoid leukemia | death | 0.004 (0.002 to 0.007) | 0.03 (0.02 to 0.06) | 0.004 (0.002 to 0.008) | 0.04 (0.02 to 0.06) | 0.09 (-0.21 to 0.5) | 0.72 (0.47 to 0.98) |
| Bulgaria | Chronic myeloid leukemia | death | 0.007 (0.003 to 0.013) | 0.06 (0.03 to 0.11) | 0.005 (0.002 to 0.008) | 0.03 (0.02 to 0.06) | -0.38 (-0.55 to -0.1) | -2.26 (-2.63 to -1.89) |
| Bulgaria | Chronic lymphoid leukemia | death | 0.005 (0.002 to 0.009) | 0.04 (0.02 to 0.07) | 0.009 (0.004 to 0.016) | 0.06 (0.03 to 0.11) | 0.86 (0.2 to 1.85) | 1.97 (1.73 to 2.21) |
| Bulgaria | Other leukemia | death | 0.013 (0.006 to 0.022) | 0.11 (0.05 to 0.19) | 0.019 (0.009 to 0.033) | 0.14 (0.07 to 0.25) | 0.48 (0.11 to 0.95) | 1.46 (0.87 to 2.06) |
| Bulgaria | Leukemia | DALYs | 1.277 (0.643 to 2.089) | 11 (5.47 to 18.17) | 1.479 (0.752 to 2.509) | 12.74 (6.34 to 21.82) | 0.16 (-0.11 to 0.48) | 0.91 (0.59 to 1.24) |
| Bulgaria | Acute myeloid leukemia | DALYs | 0.407 (0.193 to 0.683) | 3.48 (1.65 to 5.91) | 0.557 (0.271 to 0.974) | 4.98 (2.43 to 8.83) | 0.37 (0.01 to 0.84) | 1.84 (1.55 to 2.14) |
| Bulgaria | Acute lymphoid leukemia | DALYs | 0.131 (0.059 to 0.232) | 1.22 (0.54 to 2.17) | 0.129 (0.065 to 0.229) | 1.31 (0.63 to 2.33) | -0.01 (-0.29 to 0.37) | 0.77 (0.48 to 1.06) |
| Bulgaria | Chronic myeloid leukemia | DALYs | 0.21 (0.097 to 0.369) | 1.77 (0.81 to 3.14) | 0.107 (0.053 to 0.186) | 0.85 (0.42 to 1.49) | -0.49 (-0.64 to -0.25) | -2.7 (-3.09 to -2.3) |
| Bulgaria | Chronic lymphoid leukemia | DALYs | 0.123 (0.057 to 0.21) | 0.95 (0.44 to 1.64) | 0.202 (0.099 to 0.362) | 1.46 (0.72 to 2.59) | 0.65 (0.03 to 1.57) | 1.94 (1.67 to 2.22) |
| Bulgaria | Other leukemia | DALYs | 0.406 (0.2 to 0.685) | 3.58 (1.75 to 6.04) | 0.483 (0.234 to 0.825) | 4.15 (1.98 to 7.25) | 0.19 (-0.11 to 0.57) | 0.9 (0.32 to 1.49) |
| Burkina Faso | Leukemia | death | 0.003 (0.001 to 0.006) | 0.05 (0.01 to 0.13) | 0.016 (0.006 to 0.03) | 0.16 (0.06 to 0.3) | 5.21 (3.05 to 12.94) | 3.85 (3.8 to 3.9) |
| Burkina Faso | Acute myeloid leukemia | death | 0 (0 to 0.001) | 0.01 (0 to 0.02) | 0.003 (0.001 to 0.005) | 0.02 (0.01 to 0.05) | 6.44 (3.46 to 16.76) | 4.5 (4.39 to 4.62) |
| Burkina Faso | Acute lymphoid leukemia | death | 0 (0 to 0) | 0 (0 to 0.01) | 0.001 (0 to 0.002) | 0.01 (0 to 0.01) | 5.99 (3.01 to 15.78) | 3.76 (3.66 to 3.86) |
| Burkina Faso | Chronic myeloid leukemia | death | 0 (0 to 0.001) | 0.01 (0 to 0.02) | 0.003 (0.001 to 0.006) | 0.03 (0.01 to 0.05) | 5.4 (2.86 to 15.05) | 3.74 (3.68 to 3.8) |
| Burkina Faso | Chronic lymphoid leukemia | death | 0.001 (0 to 0.001) | 0.01 (0 to 0.03) | 0.003 (0.001 to 0.006) | 0.04 (0.01 to 0.07) | 4.96 (2.58 to 13.59) | 4.12 (4.06 to 4.18) |
| Burkina Faso | Other leukemia | death | 0.001 (0 to 0.003) | 0.02 (0.01 to 0.06) | 0.006 (0.002 to 0.013) | 0.06 (0.02 to 0.13) | 4.73 (2.65 to 12.13) | 3.56 (3.5 to 3.62) |
| Burkina Faso | Leukemia | DALYs | 0.083 (0.02 to 0.202) | 1.6 (0.38 to 3.84) | 0.544 (0.215 to 1.022) | 4.51 (1.78 to 8.4) | 5.56 (3.23 to 13.91) | 3.81 (3.76 to 3.86) |
| Burkina Faso | Acute myeloid leukemia | DALYs | 0.013 (0.003 to 0.032) | 0.23 (0.05 to 0.59) | 0.096 (0.035 to 0.192) | 0.74 (0.26 to 1.49) | 6.64 (3.66 to 16.73) | 4.34 (4.24 to 4.44) |
| Burkina Faso | Acute lymphoid leukemia | DALYs | 0.006 (0.001 to 0.016) | 0.1 (0.02 to 0.26) | 0.045 (0.014 to 0.097) | 0.28 (0.09 to 0.61) | 6.57 (3.13 to 17.62) | 3.96 (3.86 to 4.05) |
| Burkina Faso | Chronic myeloid leukemia | DALYs | 0.019 (0.004 to 0.049) | 0.35 (0.08 to 0.88) | 0.127 (0.047 to 0.258) | 0.93 (0.35 to 1.89) | 5.63 (2.88 to 15.7) | 3.69 (3.62 to 3.76) |
| Burkina Faso | Chronic lymphoid leukemia | DALYs | 0.012 (0.003 to 0.03) | 0.27 (0.06 to 0.64) | 0.075 (0.026 to 0.145) | 0.82 (0.29 to 1.57) | 5.12 (2.59 to 14.74) | 4.08 (4.02 to 4.14) |
| Burkina Faso | Other leukemia | DALYs | 0.033 (0.007 to 0.084) | 0.66 (0.15 to 1.63) | 0.201 (0.073 to 0.414) | 1.74 (0.63 to 3.59) | 5.1 (2.85 to 12.96) | 3.53 (3.46 to 3.6) |
| Burundi | Leukemia | death | 0.001 (0 to 0.003) | 0.05 (0.01 to 0.11) | 0.003 (0.001 to 0.007) | 0.06 (0.02 to 0.13) | 1.63 (0.68 to 4.05) | 0.84 (0.73 to 0.95) |
| Burundi | Acute myeloid leukemia | death | 0 (0 to 0) | 0 (0 to 0.01) | 0 (0 to 0.001) | 0.01 (0 to 0.01) | 3.17 (1.28 to 7.37) | 2.53 (2.4 to 2.66) |
| Burundi | Acute lymphoid leukemia | death | 0 (0 to 0) | 0 (0 to 0) | 0 (0 to 0) | 0 (0 to 0.01) | 2.98 (1.29 to 7.42) | 2.04 (1.89 to 2.18) |
| Burundi | Chronic myeloid leukemia | death | 0 (0 to 0.001) | 0.01 (0 to 0.03) | 0.001 (0 to 0.002) | 0.01 (0 to 0.03) | 0.93 (0.13 to 3.24) | -0.69 (-1 to -0.38) |
| Burundi | Chronic lymphoid leukemia | death | 0 (0 to 0.001) | 0.01 (0 to 0.03) | 0.001 (0 to 0.002) | 0.02 (0.01 to 0.05) | 2.08 (0.9 to 4.9) | 1.73 (1.65 to 1.8) |
| Burundi | Other leukemia | death | 0 (0 to 0.001) | 0.02 (0 to 0.04) | 0.001 (0 to 0.002) | 0.02 (0.01 to 0.05) | 1.52 (0.55 to 4.04) | 0.59 (0.48 to 0.7) |
| Burundi | Leukemia | DALYs | 0.043 (0.011 to 0.101) | 1.42 (0.37 to 3.31) | 0.114 (0.036 to 0.243) | 1.79 (0.58 to 3.78) | 1.67 (0.67 to 4.12) | 0.61 (0.47 to 0.75) |
| Burundi | Acute myeloid leukemia | DALYs | 0.003 (0.001 to 0.009) | 0.1 (0.03 to 0.26) | 0.014 (0.004 to 0.033) | 0.2 (0.06 to 0.46) | 3.29 (1.32 to 7.6) | 2.47 (2.35 to 2.6) |
| Burundi | Acute lymphoid leukemia | DALYs | 0.002 (0 to 0.006) | 0.06 (0.01 to 0.16) | 0.009 (0.003 to 0.021) | 0.11 (0.03 to 0.26) | 3.13 (1.33 to 7.64) | 2.15 (2.01 to 2.29) |
| Burundi | Chronic myeloid leukemia | DALYs | 0.015 (0.003 to 0.039) | 0.46 (0.11 to 1.22) | 0.029 (0.009 to 0.063) | 0.41 (0.13 to 0.89) | 0.97 (0.13 to 3.35) | -0.82 (-1.13 to -0.51) |
| Burundi | Chronic lymphoid leukemia | DALYs | 0.006 (0.001 to 0.016) | 0.27 (0.06 to 0.69) | 0.021 (0.007 to 0.045) | 0.44 (0.13 to 0.99) | 2.28 (0.97 to 5.44) | 1.65 (1.57 to 1.73) |
| Burundi | Other leukemia | DALYs | 0.016 (0.004 to 0.04) | 0.52 (0.12 to 1.27) | 0.041 (0.012 to 0.093) | 0.63 (0.18 to 1.4) | 1.55 (0.5 to 4.3) | 0.37 (0.24 to 0.51) |
| Cabo Verde | Leukemia | death | 0 (0 to 0) | 0.09 (0.03 to 0.19) | 0.001 (0.001 to 0.002) | 0.26 (0.13 to 0.47) | 4.51 (2.86 to 8.73) | 3.35 (3.14 to 3.56) |
| Cabo Verde | Acute myeloid leukemia | death | 0 (0 to 0) | 0.02 (0 to 0.03) | 0 (0 to 0) | 0.05 (0.02 to 0.1) | 5.74 (3.32 to 12.15) | 4.38 (4.15 to 4.61) |
| Cabo Verde | Acute lymphoid leukemia | death | 0 (0 to 0) | 0.01 (0 to 0.01) | 0 (0 to 0) | 0.01 (0 to 0.03) | 4.72 (2.52 to 10.69) | 3 (2.76 to 3.24) |
| Cabo Verde | Chronic myeloid leukemia | death | 0 (0 to 0) | 0.01 (0 to 0.03) | 0 (0 to 0) | 0.03 (0.01 to 0.06) | 4.19 (2.46 to 8.64) | 2.55 (2.34 to 2.76) |
| Cabo Verde | Chronic lymphoid leukemia | death | 0 (0 to 0) | 0.02 (0 to 0.03) | 0 (0 to 0) | 0.06 (0.03 to 0.11) | 4.97 (2.58 to 10.41) | 4.24 (3.99 to 4.49) |
| Cabo Verde | Other leukemia | death | 0 (0 to 0) | 0.04 (0.01 to 0.1) | 0 (0 to 0.001) | 0.11 (0.05 to 0.21) | 3.95 (2.33 to 8.31) | 2.77 (2.53 to 3.01) |
| Cabo Verde | Leukemia | DALYs | 0.007 (0.002 to 0.014) | 2.98 (1.03 to 6.09) | 0.035 (0.018 to 0.062) | 7.31 (3.59 to 12.84) | 4.23 (2.65 to 8.64) | 2.99 (2.8 to 3.18) |
| Cabo Verde | Acute myeloid leukemia | DALYs | 0.001 (0 to 0.003) | 0.55 (0.17 to 1.19) | 0.008 (0.004 to 0.015) | 1.66 (0.75 to 3.08) | 5.64 (3.26 to 12.41) | 4.04 (3.82 to 4.27) |
| Cabo Verde | Acute lymphoid leukemia | DALYs | 0.001 (0 to 0.001) | 0.23 (0.06 to 0.54) | 0.003 (0.001 to 0.005) | 0.53 (0.19 to 0.98) | 4.17 (2.01 to 10.21) | 2.76 (2.52 to 3.01) |
| Cabo Verde | Chronic myeloid leukemia | DALYs | 0.001 (0 to 0.003) | 0.54 (0.16 to 1.29) | 0.005 (0.002 to 0.01) | 1 (0.43 to 1.97) | 3.45 (1.94 to 7.24) | 2.07 (1.89 to 2.24) |
| Cabo Verde | Chronic lymphoid leukemia | DALYs | 0.001 (0 to 0.002) | 0.37 (0.11 to 0.84) | 0.005 (0.002 to 0.009) | 1.26 (0.57 to 2.28) | 4.81 (2.67 to 10.16) | 3.9 (3.68 to 4.13) |
| Cabo Verde | Other leukemia | DALYs | 0.003 (0.001 to 0.006) | 1.3 (0.42 to 2.87) | 0.014 (0.006 to 0.027) | 2.87 (1.32 to 5.66) | 3.81 (2.17 to 8.39) | 2.53 (2.31 to 2.75) |
| Cambodia | Leukemia | death | 0.004 (0.001 to 0.012) | 0.08 (0.01 to 0.22) | 0.019 (0.007 to 0.038) | 0.15 (0.05 to 0.3) | 3.46 (1.75 to 9.37) | 2.35 (2.13 to 2.57) |
| Cambodia | Acute myeloid leukemia | death | 0 (0 to 0.001) | 0.01 (0 to 0.02) | 0.003 (0.001 to 0.006) | 0.02 (0.01 to 0.05) | 6.28 (3.09 to 17.39) | 4.2 (3.93 to 4.48) |
| Cambodia | Acute lymphoid leukemia | death | 0 (0 to 0.001) | 0 (0 to 0.02) | 0.001 (0 to 0.004) | 0.01 (0 to 0.03) | 5 (2.19 to 15.81) | 3.69 (3.46 to 3.93) |
| Cambodia | Chronic myeloid leukemia | death | 0 (0 to 0.001) | 0.01 (0 to 0.02) | 0.002 (0.001 to 0.004) | 0.01 (0 to 0.03) | 3.83 (1.78 to 11.9) | 2.49 (2.23 to 2.75) |
| Cambodia | Chronic lymphoid leukemia | death | 0 (0 to 0) | 0 (0 to 0.01) | 0.001 (0 to 0.002) | 0.01 (0 to 0.01) | 7.2 (3.59 to 19.59) | 4.41 (4.14 to 4.68) |
| Cambodia | Other leukemia | death | 0.003 (0.001 to 0.009) | 0.06 (0.01 to 0.18) | 0.012 (0.004 to 0.026) | 0.1 (0.03 to 0.2) | 2.87 (1.33 to 8.54) | 1.84 (1.65 to 2.03) |
| Cambodia | Leukemia | DALYs | 0.162 (0.029 to 0.465) | 2.62 (0.49 to 7.29) | 0.636 (0.222 to 1.293) | 4.47 (1.57 to 8.97) | 2.93 (1.4 to 8.37) | 2 (1.79 to 2.21) |
| Cambodia | Acute myeloid leukemia | DALYs | 0.015 (0.002 to 0.046) | 0.24 (0.04 to 0.73) | 0.099 (0.033 to 0.231) | 0.68 (0.22 to 1.56) | 5.72 (2.62 to 16.43) | 4.04 (3.76 to 4.32) |
| Cambodia | Acute lymphoid leukemia | DALYs | 0.01 (0.001 to 0.043) | 0.15 (0.02 to 0.61) | 0.055 (0.018 to 0.138) | 0.36 (0.12 to 0.89) | 4.32 (1.7 to 14.95) | 3.42 (3.19 to 3.64) |
| Cambodia | Chronic myeloid leukemia | DALYs | 0.012 (0.002 to 0.039) | 0.2 (0.03 to 0.62) | 0.051 (0.017 to 0.117) | 0.36 (0.12 to 0.82) | 3.22 (1.36 to 10.32) | 2.08 (1.84 to 2.32) |
| Cambodia | Chronic lymphoid leukemia | DALYs | 0.003 (0 to 0.008) | 0.05 (0.01 to 0.15) | 0.02 (0.007 to 0.044) | 0.15 (0.05 to 0.34) | 6.38 (3.13 to 17.18) | 4.08 (3.83 to 4.34) |
| Cambodia | Other leukemia | DALYs | 0.122 (0.019 to 0.374) | 1.98 (0.33 to 5.85) | 0.411 (0.137 to 0.849) | 2.91 (1 to 5.98) | 2.37 (0.98 to 7.5) | 1.44 (1.26 to 1.62) |
| Cameroon | Leukemia | death | 0.01 (0.004 to 0.019) | 0.21 (0.09 to 0.38) | 0.04 (0.019 to 0.071) | 0.3 (0.14 to 0.52) | 2.9 (1.58 to 4.99) | 1.25 (1.21 to 1.3) |
| Cameroon | Acute myeloid leukemia | death | 0.002 (0.001 to 0.004) | 0.04 (0.02 to 0.08) | 0.01 (0.004 to 0.018) | 0.06 (0.03 to 0.12) | 3.48 (1.76 to 6.34) | 1.64 (1.51 to 1.77) |
| Cameroon | Acute lymphoid leukemia | death | 0 (0 to 0.001) | 0.01 (0 to 0.01) | 0.002 (0.001 to 0.003) | 0.01 (0 to 0.02) | 3.83 (1.82 to 7.49) | 1.39 (1.3 to 1.48) |
| Cameroon | Chronic myeloid leukemia | death | 0.001 (0.001 to 0.003) | 0.02 (0.01 to 0.05) | 0.004 (0.002 to 0.009) | 0.03 (0.01 to 0.06) | 2.22 (1.02 to 4.47) | 0.4 (0.36 to 0.44) |
| Cameroon | Chronic lymphoid leukemia | death | 0.002 (0.001 to 0.004) | 0.05 (0.02 to 0.1) | 0.007 (0.003 to 0.014) | 0.07 (0.03 to 0.13) | 2.61 (1.4 to 4.71) | 1.32 (1.28 to 1.35) |
| Cameroon | Other leukemia | death | 0.004 (0.002 to 0.008) | 0.09 (0.04 to 0.18) | 0.017 (0.008 to 0.03) | 0.13 (0.06 to 0.23) | 2.89 (1.56 to 5.12) | 1.25 (1.14 to 1.36) |
| Cameroon | Leukemia | DALYs | 0.348 (0.142 to 0.626) | 6.17 (2.53 to 11.13) | 1.424 (0.667 to 2.537) | 8.53 (4.04 to 15.07) | 3.09 (1.67 to 5.41) | 1.18 (1.13 to 1.23) |
| Cameroon | Acute myeloid leukemia | DALYs | 0.082 (0.032 to 0.16) | 1.32 (0.52 to 2.6) | 0.38 (0.166 to 0.722) | 2.03 (0.86 to 3.86) | 3.66 (1.81 to 6.88) | 1.55 (1.44 to 1.67) |
| Cameroon | Acute lymphoid leukemia | DALYs | 0.016 (0.006 to 0.036) | 0.22 (0.08 to 0.49) | 0.083 (0.031 to 0.172) | 0.35 (0.14 to 0.72) | 4.17 (1.91 to 8.49) | 1.55 (1.47 to 1.62) |
| Cameroon | Chronic myeloid leukemia | DALYs | 0.056 (0.021 to 0.112) | 0.89 (0.34 to 1.79) | 0.189 (0.074 to 0.397) | 0.96 (0.38 to 2.01) | 2.36 (1.06 to 4.89) | 0.29 (0.23 to 0.34) |
| Cameroon | Chronic lymphoid leukemia | DALYs | 0.052 (0.019 to 0.101) | 1.12 (0.42 to 2.19) | 0.184 (0.078 to 0.336) | 1.54 (0.67 to 2.84) | 2.51 (1.3 to 4.66) | 1.14 (1.11 to 1.17) |
| Cameroon | Other leukemia | DALYs | 0.142 (0.056 to 0.271) | 2.6 (1.04 to 4.96) | 0.587 (0.262 to 1.066) | 3.64 (1.65 to 6.54) | 3.15 (1.67 to 5.67) | 1.24 (1.12 to 1.37) |
| Canada | Leukemia | death | 0.145 (0.067 to 0.244) | 0.45 (0.21 to 0.76) | 0.325 (0.163 to 0.521) | 0.47 (0.24 to 0.75) | 1.25 (0.97 to 1.71) | 0.23 (0.16 to 0.3) |
| Canada | Acute myeloid leukemia | death | 0.04 (0.018 to 0.069) | 0.12 (0.06 to 0.21) | 0.132 (0.063 to 0.217) | 0.2 (0.1 to 0.32) | 2.34 (1.58 to 3.22) | 2.1 (1.87 to 2.32) |
| Canada | Acute lymphoid leukemia | death | 0.009 (0.004 to 0.015) | 0.03 (0.01 to 0.05) | 0.015 (0.008 to 0.026) | 0.03 (0.01 to 0.04) | 0.78 (0.43 to 1.39) | -0.08 (-0.19 to 0.03) |
| Canada | Chronic myeloid leukemia | death | 0.018 (0.008 to 0.03) | 0.06 (0.03 to 0.09) | 0.016 (0.008 to 0.026) | 0.02 (0.01 to 0.04) | -0.11 (-0.28 to 0.13) | -3.78 (-4.19 to -3.36) |
| Canada | Chronic lymphoid leukemia | death | 0.028 (0.013 to 0.048) | 0.09 (0.04 to 0.15) | 0.07 (0.035 to 0.117) | 0.09 (0.05 to 0.16) | 1.53 (1.07 to 2.25) | 0.12 (-0.17 to 0.41) |
| Canada | Other leukemia | death | 0.051 (0.023 to 0.086) | 0.16 (0.07 to 0.27) | 0.091 (0.045 to 0.152) | 0.13 (0.06 to 0.21) | 0.8 (0.49 to 1.24) | -0.71 (-0.78 to -0.65) |
| Canada | Leukemia | DALYs | 3.614 (1.707 to 6.101) | 11.33 (5.36 to 19.18) | 6.802 (3.5 to 10.904) | 11.14 (5.76 to 17.82) | 0.88 (0.64 to 1.26) | -0.07 (-0.13 to -0.01) |
| Canada | Acute myeloid leukemia | DALYs | 1.055 (0.487 to 1.825) | 3.33 (1.53 to 5.76) | 2.893 (1.429 to 4.676) | 4.87 (2.41 to 7.91) | 1.74 (1.21 to 2.43) | 1.63 (1.44 to 1.81) |
| Canada | Acute lymphoid leukemia | DALYs | 0.323 (0.152 to 0.567) | 1.04 (0.49 to 1.83) | 0.553 (0.279 to 0.918) | 1.13 (0.58 to 1.89) | 0.71 (0.35 to 1.32) | 0.29 (0.15 to 0.43) |
| Canada | Chronic myeloid leukemia | DALYs | 0.481 (0.229 to 0.81) | 1.52 (0.72 to 2.56) | 0.376 (0.188 to 0.622) | 0.64 (0.32 to 1.06) | -0.22 (-0.36 to -0.01) | -3.78 (-4.21 to -3.36) |
| Canada | Chronic lymphoid leukemia | DALYs | 0.581 (0.276 to 0.988) | 1.78 (0.85 to 3.03) | 1.318 (0.677 to 2.173) | 1.9 (0.97 to 3.13) | 1.27 (0.86 to 1.92) | -0.07 (-0.36 to 0.22) |
| Canada | Other leukemia | DALYs | 1.174 (0.552 to 1.999) | 3.66 (1.72 to 6.23) | 1.662 (0.837 to 2.693) | 2.61 (1.31 to 4.17) | 0.42 (0.19 to 0.77) | -1.28 (-1.35 to -1.2) |
| Central African Republic | Leukemia | death | 0.001 (0 to 0.002) | 0.06 (0.01 to 0.13) | 0.002 (0.001 to 0.004) | 0.07 (0.02 to 0.16) | 1.45 (0.72 to 3.28) | 1.17 (1 to 1.34) |
| Central African Republic | Acute myeloid leukemia | death | 0 (0 to 0) | 0 (0 to 0.01) | 0 (0 to 0) | 0.01 (0 to 0.01) | 1.62 (0.56 to 4.11) | 1.24 (1.14 to 1.35) |
| Central African Republic | Acute lymphoid leukemia | death | 0 (0 to 0) | 0 (0 to 0) | 0 (0 to 0) | 0 (0 to 0.01) | 1.65 (0.76 to 4.05) | 1.05 (0.97 to 1.13) |
| Central African Republic | Chronic myeloid leukemia | death | 0 (0 to 0) | 0.01 (0 to 0.03) | 0 (0 to 0.001) | 0.01 (0 to 0.03) | 1.28 (0.45 to 3.35) | 0.76 (0.65 to 0.88) |
| Central African Republic | Chronic lymphoid leukemia | death | 0 (0 to 0) | 0.01 (0 to 0.02) | 0 (0 to 0.001) | 0.02 (0.01 to 0.05) | 3.83 (2.04 to 8.62) | 4.24 (3.67 to 4.81) |
| Central African Republic | Other leukemia | death | 0 (0 to 0.001) | 0.03 (0.01 to 0.08) | 0.001 (0 to 0.002) | 0.03 (0.01 to 0.08) | 1.01 (0.43 to 2.31) | 0.11 (-0.03 to 0.25) |
| Central African Republic | Leukemia | DALYs | 0.026 (0.006 to 0.064) | 1.68 (0.43 to 4.01) | 0.066 (0.02 to 0.153) | 2.15 (0.65 to 4.83) | 1.49 (0.73 to 3.36) | 1.11 (0.95 to 1.27) |
| Central African Republic | Acute myeloid leukemia | DALYs | 0.002 (0.001 to 0.006) | 0.14 (0.03 to 0.37) | 0.006 (0.002 to 0.015) | 0.19 (0.06 to 0.45) | 1.73 (0.62 to 4.21) | 1.31 (1.2 to 1.42) |
| Central African Republic | Acute lymphoid leukemia | DALYs | 0.001 (0 to 0.003) | 0.06 (0.01 to 0.17) | 0.003 (0.001 to 0.009) | 0.09 (0.02 to 0.22) | 1.76 (0.81 to 4.28) | 1.19 (1.11 to 1.26) |
| Central African Republic | Chronic myeloid leukemia | DALYs | 0.005 (0.001 to 0.015) | 0.33 (0.07 to 0.87) | 0.013 (0.004 to 0.031) | 0.39 (0.11 to 0.9) | 1.38 (0.49 to 3.47) | 0.87 (0.75 to 0.98) |
| Central African Republic | Chronic lymphoid leukemia | DALYs | 0.002 (0 to 0.006) | 0.18 (0.04 to 0.47) | 0.011 (0.003 to 0.027) | 0.47 (0.13 to 1.1) | 3.92 (2.04 to 8.85) | 4.18 (3.61 to 4.76) |
| Central African Republic | Other leukemia | DALYs | 0.015 (0.003 to 0.038) | 0.96 (0.22 to 2.39) | 0.032 (0.008 to 0.076) | 1.01 (0.28 to 2.38) | 1.11 (0.48 to 2.49) | 0.22 (0.08 to 0.36) |
| Chad | Leukemia | death | 0.001 (0 to 0.003) | 0.04 (0.01 to 0.09) | 0.006 (0.002 to 0.012) | 0.1 (0.04 to 0.19) | 4.19 (2.56 to 9.01) | 3.27 (3.13 to 3.41) |
| Chad | Acute myeloid leukemia | death | 0 (0 to 0) | 0.01 (0 to 0.01) | 0.001 (0 to 0.002) | 0.01 (0 to 0.03) | 4.88 (2.61 to 11.56) | 3.74 (3.66 to 3.81) |
| Chad | Acute lymphoid leukemia | death | 0 (0 to 0) | 0 (0 to 0) | 0 (0 to 0.001) | 0 (0 to 0.01) | 4.73 (2.48 to 11.63) | 3.11 (3.06 to 3.16) |
| Chad | Chronic myeloid leukemia | death | 0 (0 to 0.001) | 0.01 (0 to 0.02) | 0.001 (0 to 0.002) | 0.02 (0.01 to 0.03) | 4.17 (2.28 to 9.56) | 2.94 (2.81 to 3.07) |
| Chad | Chronic lymphoid leukemia | death | 0 (0 to 0.001) | 0.01 (0 to 0.02) | 0.001 (0 to 0.002) | 0.02 (0.01 to 0.04) | 3.57 (1.8 to 9.53) | 3.22 (3.11 to 3.32) |
| Chad | Other leukemia | death | 0.001 (0 to 0.001) | 0.02 (0 to 0.04) | 0.003 (0.001 to 0.005) | 0.04 (0.01 to 0.09) | 4.16 (2.42 to 9.16) | 3.28 (3.08 to 3.49) |
| Chad | Leukemia | DALYs | 0.038 (0.009 to 0.09) | 1.17 (0.29 to 2.75) | 0.21 (0.077 to 0.417) | 2.83 (1.05 to 5.57) | 4.57 (2.77 to 9.92) | 3.25 (3.11 to 3.39) |
| Chad | Acute myeloid leukemia | DALYs | 0.006 (0.001 to 0.016) | 0.17 (0.04 to 0.43) | 0.037 (0.012 to 0.079) | 0.46 (0.15 to 0.98) | 5.2 (2.83 to 12.46) | 3.64 (3.57 to 3.7) |
| Chad | Acute lymphoid leukemia | DALYs | 0.003 (0.001 to 0.008) | 0.07 (0.01 to 0.19) | 0.017 (0.005 to 0.036) | 0.17 (0.05 to 0.37) | 5.08 (2.56 to 12.78) | 3.17 (3.12 to 3.23) |
| Chad | Chronic myeloid leukemia | DALYs | 0.008 (0.002 to 0.021) | 0.25 (0.06 to 0.63) | 0.045 (0.015 to 0.095) | 0.54 (0.18 to 1.11) | 4.37 (2.4 to 10.02) | 2.87 (2.74 to 3.01) |
| Chad | Chronic lymphoid leukemia | DALYs | 0.005 (0.001 to 0.013) | 0.19 (0.04 to 0.46) | 0.025 (0.008 to 0.052) | 0.45 (0.15 to 0.94) | 3.76 (1.86 to 10.08) | 3.17 (3.08 to 3.26) |
| Chad | Other leukemia | DALYs | 0.015 (0.004 to 0.038) | 0.49 (0.12 to 1.2) | 0.087 (0.03 to 0.183) | 1.22 (0.43 to 2.53) | 4.63 (2.66 to 10.04) | 3.32 (3.11 to 3.54) |
| Chile | Leukemia | death | 0.031 (0.013 to 0.054) | 0.3 (0.13 to 0.51) | 0.087 (0.042 to 0.143) | 0.37 (0.18 to 0.61) | 1.82 (1.44 to 2.45) | 0.85 (0.65 to 1.05) |
| Chile | Acute myeloid leukemia | death | 0.008 (0.003 to 0.014) | 0.07 (0.03 to 0.13) | 0.031 (0.015 to 0.051) | 0.13 (0.06 to 0.22) | 2.95 (2.2 to 4.01) | 2.19 (1.91 to 2.47) |
| Chile | Acute lymphoid leukemia | death | 0.004 (0.002 to 0.007) | 0.04 (0.01 to 0.06) | 0.012 (0.006 to 0.021) | 0.06 (0.03 to 0.09) | 2.05 (1.27 to 3.07) | 1.46 (1.12 to 1.8) |
| Chile | Chronic myeloid leukemia | death | 0.006 (0.002 to 0.01) | 0.05 (0.02 to 0.09) | 0.008 (0.004 to 0.014) | 0.03 (0.02 to 0.06) | 0.44 (0.18 to 0.85) | -2.02 (-2.55 to -1.48) |
| Chile | Chronic lymphoid leukemia | death | 0.002 (0.001 to 0.004) | 0.03 (0.01 to 0.05) | 0.009 (0.004 to 0.015) | 0.04 (0.02 to 0.06) | 2.62 (1.89 to 3.85) | 1 (0.61 to 1.39) |
| Chile | Other leukemia | death | 0.011 (0.005 to 0.019) | 0.11 (0.05 to 0.19) | 0.027 (0.013 to 0.045) | 0.11 (0.05 to 0.19) | 1.46 (1.04 to 2.11) | 0.48 (0.35 to 0.61) |
| Chile | Leukemia | DALYs | 0.997 (0.426 to 1.756) | 8.67 (3.75 to 15.12) | 2.312 (1.151 to 3.771) | 10.29 (5.12 to 16.8) | 1.32 (0.99 to 1.89) | 0.62 (0.43 to 0.8) |
| Chile | Acute myeloid leukemia | DALYs | 0.271 (0.115 to 0.491) | 2.31 (0.99 to 4.17) | 0.881 (0.424 to 1.453) | 3.94 (1.9 to 6.51) | 2.25 (1.64 to 3.23) | 1.86 (1.6 to 2.13) |
| Chile | Acute lymphoid leukemia | DALYs | 0.166 (0.067 to 0.3) | 1.32 (0.54 to 2.38) | 0.448 (0.217 to 0.735) | 2.1 (1.01 to 3.46) | 1.69 (1.03 to 2.77) | 1.47 (1.14 to 1.8) |
| Chile | Chronic myeloid leukemia | DALYs | 0.179 (0.079 to 0.309) | 1.59 (0.7 to 2.74) | 0.2 (0.098 to 0.329) | 0.88 (0.43 to 1.44) | 0.12 (-0.09 to 0.47) | -2.5 (-3.02 to -1.97) |
| Chile | Chronic lymphoid leukemia | DALYs | 0.053 (0.024 to 0.093) | 0.53 (0.24 to 0.94) | 0.162 (0.077 to 0.276) | 0.68 (0.32 to 1.15) | 2.08 (1.46 to 3.07) | 0.7 (0.34 to 1.07) |
| Chile | Other leukemia | DALYs | 0.327 (0.142 to 0.567) | 2.92 (1.28 to 5.06) | 0.621 (0.305 to 1.017) | 2.7 (1.33 to 4.43) | 0.9 (0.57 to 1.43) | 0.04 (-0.11 to 0.19) |
| China | Leukemia | death | 0.831 (0.16 to 2.104) | 0.09 (0.02 to 0.22) | 2.535 (0.92 to 5.039) | 0.13 (0.05 to 0.26) | 2.05 (1.11 to 5.41) | 1.54 (1.43 to 1.65) |
| China | Acute myeloid leukemia | death | 0.055 (0.01 to 0.142) | 0.01 (0 to 0.01) | 0.331 (0.12 to 0.648) | 0.02 (0.01 to 0.03) | 4.99 (2.63 to 12.48) | 4.11 (3.98 to 4.25) |
| China | Acute lymphoid leukemia | death | 0.074 (0.013 to 0.206) | 0.01 (0 to 0.02) | 0.43 (0.15 to 0.859) | 0.02 (0.01 to 0.05) | 4.85 (1.93 to 13.29) | 4.83 (4.45 to 5.2) |
| China | Chronic myeloid leukemia | death | 0.024 (0.005 to 0.061) | 0 (0 to 0.01) | 0.053 (0.019 to 0.103) | 0 (0 to 0.01) | 1.25 (0.43 to 3.89) | -0.04 (-0.16 to 0.08) |
| China | Chronic lymphoid leukemia | death | 0.028 (0.005 to 0.073) | 0 (0 to 0.01) | 0.216 (0.077 to 0.42) | 0.01 (0 to 0.02) | 6.7 (3.86 to 15.73) | 5.22 (4.93 to 5.51) |
| China | Other leukemia | death | 0.65 (0.124 to 1.651) | 0.07 (0.01 to 0.17) | 1.505 (0.537 to 3.002) | 0.08 (0.03 to 0.15) | 1.31 (0.56 to 4.04) | 0.38 (0.15 to 0.61) |
| China | Leukemia | DALYs | 32.519 (6.124 to 82.757) | 2.96 (0.57 to 7.46) | 84.303 (30.802 to 167.814) | 4.46 (1.61 to 8.86) | 1.59 (0.75 to 4.62) | 1.41 (1.29 to 1.52) |
| China | Acute myeloid leukemia | DALYs | 2.094 (0.387 to 5.377) | 0.19 (0.04 to 0.49) | 10.713 (3.928 to 20.825) | 0.57 (0.21 to 1.1) | 4.12 (2.03 to 10.82) | 4.09 (3.95 to 4.23) |
| China | Acute lymphoid leukemia | DALYs | 2.986 (0.515 to 8.579) | 0.26 (0.05 to 0.74) | 16.453 (5.695 to 32.771) | 0.89 (0.31 to 1.78) | 4.51 (1.7 to 12.81) | 5.14 (4.74 to 5.54) |
| China | Chronic myeloid leukemia | DALYs | 0.854 (0.162 to 2.249) | 0.08 (0.02 to 0.21) | 1.719 (0.626 to 3.326) | 0.09 (0.03 to 0.17) | 1.01 (0.27 to 3.58) | 0.01 (-0.13 to 0.15) |
| China | Chronic lymphoid leukemia | DALYs | 1.01 (0.185 to 2.654) | 0.1 (0.02 to 0.25) | 6.947 (2.545 to 13.361) | 0.36 (0.13 to 0.69) | 5.88 (3.26 to 14.62) | 5.27 (4.97 to 5.56) |
| China | Other leukemia | DALYs | 25.575 (4.79 to 65.934) | 2.32 (0.44 to 5.97) | 48.47 (17.263 to 96.262) | 2.55 (0.9 to 5.09) | 0.9 (0.25 to 3.27) | 0.09 (-0.17 to 0.35) |
| Colombia | Leukemia | death | 0.046 (0.019 to 0.086) | 0.23 (0.09 to 0.43) | 0.155 (0.071 to 0.275) | 0.29 (0.14 to 0.52) | 2.38 (1.47 to 3.86) | 0.77 (0.68 to 0.86) |
| Colombia | Acute myeloid leukemia | death | 0.008 (0.003 to 0.016) | 0.04 (0.02 to 0.08) | 0.049 (0.022 to 0.088) | 0.09 (0.04 to 0.17) | 4.74 (2.87 to 7.76) | 2.93 (2.73 to 3.13) |
| Colombia | Acute lymphoid leukemia | death | 0.008 (0.003 to 0.015) | 0.03 (0.01 to 0.06) | 0.034 (0.015 to 0.061) | 0.07 (0.03 to 0.12) | 3.37 (1.84 to 5.8) | 2.43 (2.24 to 2.62) |
| Colombia | Chronic myeloid leukemia | death | 0.006 (0.002 to 0.01) | 0.03 (0.01 to 0.05) | 0.016 (0.007 to 0.028) | 0.03 (0.01 to 0.05) | 1.83 (0.99 to 3.15) | -0.46 (-0.87 to -0.06) |
| Colombia | Chronic lymphoid leukemia | death | 0.003 (0.001 to 0.006) | 0.02 (0.01 to 0.03) | 0.012 (0.005 to 0.021) | 0.02 (0.01 to 0.04) | 2.97 (1.8 to 4.85) | 0.43 (0.26 to 0.6) |
| Colombia | Other leukemia | death | 0.021 (0.009 to 0.039) | 0.11 (0.04 to 0.2) | 0.044 (0.02 to 0.08) | 0.08 (0.04 to 0.15) | 1.12 (0.51 to 2.09) | -1.04 (-1.19 to -0.89) |
| Colombia | Leukemia | DALYs | 1.651 (0.646 to 3.134) | 7.02 (2.84 to 13.1) | 4.803 (2.246 to 8.57) | 9.22 (4.31 to 16.49) | 1.91 (1.07 to 3.36) | 0.91 (0.82 to 0.99) |
| Colombia | Acute myeloid leukemia | DALYs | 0.315 (0.118 to 0.613) | 1.32 (0.51 to 2.52) | 1.546 (0.717 to 2.762) | 2.97 (1.38 to 5.3) | 3.91 (2.25 to 6.6) | 2.91 (2.72 to 3.1) |
| Colombia | Acute lymphoid leukemia | DALYs | 0.332 (0.122 to 0.647) | 1.25 (0.48 to 2.4) | 1.335 (0.612 to 2.386) | 2.57 (1.18 to 4.59) | 3.01 (1.58 to 5.57) | 2.79 (2.59 to 2.99) |
| Colombia | Chronic myeloid leukemia | DALYs | 0.19 (0.077 to 0.354) | 0.85 (0.35 to 1.58) | 0.441 (0.205 to 0.787) | 0.85 (0.39 to 1.51) | 1.32 (0.62 to 2.51) | -0.62 (-1.01 to -0.23) |
| Colombia | Chronic lymphoid leukemia | DALYs | 0.074 (0.031 to 0.138) | 0.4 (0.17 to 0.75) | 0.25 (0.115 to 0.453) | 0.48 (0.22 to 0.87) | 2.38 (1.39 to 4.01) | 0.32 (0.15 to 0.49) |
| Colombia | Other leukemia | DALYs | 0.739 (0.296 to 1.398) | 3.2 (1.31 to 5.93) | 1.231 (0.573 to 2.22) | 2.36 (1.09 to 4.25) | 0.67 (0.16 to 1.5) | -1.24 (-1.44 to -1.03) |
| Comoros | Leukemia | death | 0 (0 to 0) | 0.07 (0.02 to 0.16) | 0.001 (0 to 0.001) | 0.12 (0.05 to 0.23) | 2.68 (1.34 to 7.86) | 1.7 (1.53 to 1.86) |
| Comoros | Acute myeloid leukemia | death | 0 (0 to 0) | 0.01 (0 to 0.02) | 0 (0 to 0) | 0.02 (0.01 to 0.03) | 3.77 (1.55 to 13.18) | 2.56 (2.34 to 2.78) |
| Comoros | Acute lymphoid leukemia | death | 0 (0 to 0) | 0 (0 to 0.01) | 0 (0 to 0) | 0.01 (0 to 0.01) | 3.54 (1.43 to 13.26) | 2.31 (1.98 to 2.64) |
| Comoros | Chronic myeloid leukemia | death | 0 (0 to 0) | 0.01 (0 to 0.03) | 0 (0 to 0) | 0.02 (0.01 to 0.04) | 2.13 (0.75 to 8.66) | 0.98 (0.69 to 1.26) |
| Comoros | Chronic lymphoid leukemia | death | 0 (0 to 0) | 0.02 (0.01 to 0.05) | 0 (0 to 0) | 0.04 (0.02 to 0.09) | 4.05 (2.14 to 8.84) | 2.94 (2.86 to 3.03) |
| Comoros | Other leukemia | death | 0 (0 to 0) | 0.03 (0.01 to 0.07) | 0 (0 to 0) | 0.04 (0.01 to 0.07) | 1.79 (0.68 to 6.03) | 0.56 (0.34 to 0.79) |
| Comoros | Leukemia | DALYs | 0.006 (0.001 to 0.013) | 2.11 (0.54 to 4.72) | 0.021 (0.008 to 0.039) | 3.58 (1.43 to 6.82) | 2.64 (1.17 to 9.46) | 1.59 (1.33 to 1.85) |
| Comoros | Acute myeloid leukemia | DALYs | 0.001 (0 to 0.002) | 0.24 (0.05 to 0.64) | 0.003 (0.001 to 0.007) | 0.54 (0.21 to 1.06) | 3.88 (1.46 to 15.86) | 2.61 (2.31 to 2.91) |
| Comoros | Acute lymphoid leukemia | DALYs | 0 (0 to 0.001) | 0.12 (0.02 to 0.31) | 0.002 (0.001 to 0.004) | 0.26 (0.1 to 0.52) | 3.73 (1.38 to 17.06) | 2.45 (2.03 to 2.89) |
| Comoros | Chronic myeloid leukemia | DALYs | 0.001 (0 to 0.003) | 0.47 (0.1 to 1.13) | 0.004 (0.002 to 0.009) | 0.68 (0.26 to 1.38) | 2.17 (0.66 to 9.89) | 0.95 (0.6 to 1.3) |
| Comoros | Chronic lymphoid leukemia | DALYs | 0.001 (0 to 0.002) | 0.41 (0.11 to 0.98) | 0.005 (0.002 to 0.009) | 0.95 (0.34 to 1.85) | 4.2 (2.17 to 10.34) | 3.12 (3.03 to 3.21) |
| Comoros | Other leukemia | DALYs | 0.002 (0.001 to 0.006) | 0.87 (0.22 to 2.03) | 0.007 (0.002 to 0.013) | 1.14 (0.42 to 2.25) | 1.8 (0.58 to 7.29) | 0.5 (0.19 to 0.82) |
| Congo | Leukemia | death | 0.002 (0.001 to 0.003) | 0.14 (0.05 to 0.27) | 0.006 (0.003 to 0.01) | 0.2 (0.09 to 0.35) | 2.51 (1.33 to 5.32) | 1.16 (1.05 to 1.27) |
| Congo | Acute myeloid leukemia | death | 0 (0 to 0) | 0.01 (0 to 0.04) | 0.001 (0 to 0.002) | 0.03 (0.01 to 0.05) | 3.69 (1.7 to 8.27) | 1.9 (1.74 to 2.06) |
| Congo | Acute lymphoid leukemia | death | 0 (0 to 0) | 0 (0 to 0.01) | 0 (0 to 0.001) | 0.01 (0 to 0.01) | 3.15 (1.44 to 7.62) | 1.31 (1.12 to 1.5) |
| Congo | Chronic myeloid leukemia | death | 0 (0 to 0.001) | 0.03 (0.01 to 0.06) | 0.001 (0 to 0.002) | 0.03 (0.01 to 0.05) | 2.05 (0.88 to 5.26) | 0.41 (0.14 to 0.68) |
| Congo | Chronic lymphoid leukemia | death | 0 (0 to 0.001) | 0.02 (0.01 to 0.05) | 0.002 (0.001 to 0.003) | 0.07 (0.03 to 0.13) | 5.88 (3.29 to 12.1) | 4.25 (3.88 to 4.61) |
| Congo | Other leukemia | death | 0.001 (0 to 0.002) | 0.07 (0.02 to 0.15) | 0.002 (0.001 to 0.004) | 0.07 (0.03 to 0.13) | 1.44 (0.55 to 3.63) | -0.55 (-0.74 to -0.35) |
| Congo | Leukemia | DALYs | 0.056 (0.02 to 0.113) | 4.15 (1.51 to 8.16) | 0.203 (0.095 to 0.363) | 5.68 (2.59 to 10.06) | 2.61 (1.31 to 5.9) | 1.07 (0.95 to 1.2) |
| Congo | Acute myeloid leukemia | DALYs | 0.007 (0.002 to 0.017) | 0.48 (0.15 to 1.18) | 0.034 (0.015 to 0.065) | 0.87 (0.38 to 1.57) | 4.01 (1.86 to 9.45) | 2.05 (1.88 to 2.22) |
| Congo | Acute lymphoid leukemia | DALYs | 0.003 (0.001 to 0.007) | 0.16 (0.05 to 0.4) | 0.012 (0.005 to 0.024) | 0.27 (0.12 to 0.54) | 3.47 (1.47 to 8.94) | 1.63 (1.45 to 1.81) |
| Congo | Chronic myeloid leukemia | DALYs | 0.012 (0.003 to 0.03) | 0.84 (0.26 to 2.06) | 0.038 (0.016 to 0.078) | 0.96 (0.41 to 1.88) | 2.25 (0.94 to 5.97) | 0.51 (0.26 to 0.76) |
| Congo | Chronic lymphoid leukemia | DALYs | 0.006 (0.002 to 0.014) | 0.54 (0.17 to 1.19) | 0.044 (0.017 to 0.084) | 1.55 (0.62 to 2.95) | 6.06 (3.32 to 12.66) | 4.16 (3.78 to 4.54) |
| Congo | Other leukemia | DALYs | 0.029 (0.01 to 0.061) | 2.13 (0.74 to 4.36) | 0.076 (0.032 to 0.135) | 2.03 (0.87 to 3.69) | 1.61 (0.61 to 4.2) | -0.43 (-0.62 to -0.24) |
| Cook Islands | Leukemia | death | 0 (0 to 0) | 0.29 (0.14 to 0.49) | 0 (0 to 0) | 0.27 (0.14 to 0.45) | 0.57 (0.17 to 1.18) | -0.37 (-0.45 to -0.29) |
| Cook Islands | Acute myeloid leukemia | death | 0 (0 to 0) | 0.08 (0.04 to 0.14) | 0 (0 to 0) | 0.09 (0.05 to 0.16) | 0.74 (0.2 to 1.59) | 0.19 (0.13 to 0.25) |
| Cook Islands | Acute lymphoid leukemia | death | 0 (0 to 0) | 0.02 (0.01 to 0.03) | 0 (0 to 0) | 0.02 (0.01 to 0.03) | 0.44 (-0.05 to 1.16) | -0.11 (-0.14 to -0.07) |
| Cook Islands | Chronic myeloid leukemia | death | 0 (0 to 0) | 0.04 (0.02 to 0.07) | 0 (0 to 0) | 0.03 (0.01 to 0.06) | 0.37 (-0.12 to 1.26) | -0.72 (-0.83 to -0.61) |
| Cook Islands | Chronic lymphoid leukemia | death | 0 (0 to 0) | 0.01 (0 to 0.01) | 0 (0 to 0) | 0.01 (0 to 0.01) | 0.68 (0.08 to 1.63) | -0.69 (-0.84 to -0.54) |
| Cook Islands | Other leukemia | death | 0 (0 to 0) | 0.15 (0.07 to 0.24) | 0 (0 to 0) | 0.12 (0.06 to 0.21) | 0.54 (0.13 to 1.19) | -0.67 (-0.77 to -0.57) |
| Cook Islands | Leukemia | DALYs | 0.001 (0.001 to 0.002) | 9.1 (4.51 to 14.88) | 0.002 (0.001 to 0.003) | 8.18 (4.16 to 13.52) | 0.3 (-0.07 to 0.87) | -0.4 (-0.49 to -0.3) |
| Cook Islands | Acute myeloid leukemia | DALYs | 0 (0 to 0.001) | 2.93 (1.43 to 5) | 0.001 (0 to 0.001) | 3.19 (1.58 to 5.33) | 0.45 (-0.04 to 1.26) | 0.19 (0.14 to 0.24) |
| Cook Islands | Acute lymphoid leukemia | DALYs | 0 (0 to 0) | 0.71 (0.34 to 1.22) | 0 (0 to 0) | 0.69 (0.32 to 1.24) | 0.21 (-0.26 to 0.92) | -0.06 (-0.14 to 0.01) |
| Cook Islands | Chronic myeloid leukemia | DALYs | 0 (0 to 0) | 1.23 (0.56 to 2.21) | 0 (0 to 0) | 0.96 (0.44 to 1.74) | 0.18 (-0.24 to 0.96) | -0.78 (-0.94 to -0.63) |
| Cook Islands | Chronic lymphoid leukemia | DALYs | 0 (0 to 0) | 0.18 (0.08 to 0.33) | 0 (0 to 0) | 0.16 (0.08 to 0.28) | 0.52 (-0.01 to 1.36) | -0.6 (-0.75 to -0.45) |
| Cook Islands | Other leukemia | DALYs | 0.001 (0 to 0.001) | 4.05 (1.99 to 6.8) | 0.001 (0 to 0.001) | 3.18 (1.54 to 5.61) | 0.23 (-0.14 to 0.82) | -0.84 (-0.97 to -0.71) |
| Costa Rica | Leukemia | death | 0.007 (0.003 to 0.012) | 0.35 (0.15 to 0.62) | 0.022 (0.01 to 0.039) | 0.42 (0.19 to 0.75) | 2.23 (1.39 to 3.51) | 0.46 (0.31 to 0.6) |
| Costa Rica | Acute myeloid leukemia | death | 0.002 (0.001 to 0.003) | 0.1 (0.04 to 0.17) | 0.008 (0.004 to 0.014) | 0.15 (0.07 to 0.28) | 3.15 (1.97 to 4.99) | 1.65 (1.53 to 1.77) |
| Costa Rica | Acute lymphoid leukemia | death | 0.001 (0.001 to 0.003) | 0.07 (0.03 to 0.12) | 0.005 (0.002 to 0.008) | 0.09 (0.04 to 0.16) | 2.23 (1.3 to 3.75) | 1.05 (0.77 to 1.33) |
| Costa Rica | Chronic myeloid leukemia | death | 0.001 (0.001 to 0.002) | 0.07 (0.03 to 0.13) | 0.003 (0.001 to 0.005) | 0.05 (0.02 to 0.09) | 1 (0.42 to 1.93) | -1.65 (-2.02 to -1.28) |
| Costa Rica | Chronic lymphoid leukemia | death | 0 (0 to 0.001) | 0.03 (0.01 to 0.05) | 0.002 (0.001 to 0.004) | 0.04 (0.02 to 0.07) | 3.59 (2.2 to 5.89) | 1.23 (0.91 to 1.55) |
| Costa Rica | Other leukemia | death | 0.002 (0.001 to 0.003) | 0.09 (0.04 to 0.16) | 0.004 (0.002 to 0.008) | 0.08 (0.04 to 0.15) | 1.72 (0.97 to 2.88) | -0.59 (-0.84 to -0.34) |
| Costa Rica | Leukemia | DALYs | 0.224 (0.093 to 0.395) | 10.18 (4.3 to 17.9) | 0.657 (0.304 to 1.16) | 12.62 (5.84 to 22.33) | 1.93 (1.15 to 3.21) | 0.62 (0.46 to 0.77) |
| Costa Rica | Acute myeloid leukemia | DALYs | 0.068 (0.028 to 0.121) | 3.06 (1.28 to 5.4) | 0.252 (0.115 to 0.444) | 4.83 (2.21 to 8.52) | 2.68 (1.62 to 4.48) | 1.62 (1.49 to 1.74) |
| Costa Rica | Acute lymphoid leukemia | DALYs | 0.062 (0.026 to 0.115) | 2.46 (1.02 to 4.51) | 0.187 (0.084 to 0.341) | 3.58 (1.6 to 6.51) | 2.02 (1.13 to 3.58) | 1.3 (1.02 to 1.58) |
| Costa Rica | Chronic myeloid leukemia | DALYs | 0.037 (0.016 to 0.066) | 1.86 (0.77 to 3.28) | 0.067 (0.031 to 0.119) | 1.3 (0.59 to 2.28) | 0.81 (0.27 to 1.66) | -1.66 (-2.03 to -1.3) |
| Costa Rica | Chronic lymphoid leukemia | DALYs | 0.009 (0.004 to 0.016) | 0.53 (0.23 to 0.94) | 0.038 (0.017 to 0.07) | 0.75 (0.33 to 1.38) | 3.13 (1.84 to 5.25) | 1.07 (0.76 to 1.38) |
| Costa Rica | Other leukemia | DALYs | 0.047 (0.02 to 0.084) | 2.27 (0.97 to 4.06) | 0.112 (0.051 to 0.203) | 2.16 (0.98 to 3.93) | 1.39 (0.7 to 2.45) | -0.54 (-0.81 to -0.27) |
| Croatia | Leukemia | death | 0.03 (0.014 to 0.05) | 0.47 (0.22 to 0.79) | 0.045 (0.022 to 0.076) | 0.51 (0.25 to 0.85) | 0.52 (0.18 to 0.99) | 0.6 (0.44 to 0.75) |
| Croatia | Acute myeloid leukemia | death | 0.004 (0.001 to 0.01) | 0.06 (0.02 to 0.15) | 0.016 (0.007 to 0.029) | 0.19 (0.09 to 0.34) | 3.34 (0.25 to 6.61) | 4.66 (4.23 to 5.09) |
| Croatia | Acute lymphoid leukemia | death | 0.001 (0.001 to 0.002) | 0.02 (0.01 to 0.04) | 0.002 (0.001 to 0.004) | 0.03 (0.01 to 0.05) | 0.85 (0.07 to 1.74) | 1.15 (0.83 to 1.47) |
| Croatia | Chronic myeloid leukemia | death | 0.002 (0.001 to 0.004) | 0.04 (0.02 to 0.06) | 0.003 (0.001 to 0.005) | 0.03 (0.02 to 0.06) | 0.26 (-0.12 to 1.15) | -0.74 (-1.34 to -0.13) |
| Croatia | Chronic lymphoid leukemia | death | 0.011 (0.005 to 0.018) | 0.17 (0.08 to 0.28) | 0.016 (0.008 to 0.028) | 0.17 (0.08 to 0.3) | 0.56 (0.13 to 1.43) | 0.92 (0.35 to 1.5) |
| Croatia | Other leukemia | death | 0.012 (0.006 to 0.02) | 0.19 (0.09 to 0.33) | 0.008 (0.003 to 0.013) | 0.08 (0.04 to 0.15) | -0.37 (-0.55 to 0.05) | -3.13 (-3.28 to -2.98) |
| Croatia | Leukemia | DALYs | 0.772 (0.371 to 1.293) | 12.15 (5.79 to 20.5) | 0.936 (0.46 to 1.562) | 11.78 (5.87 to 19.57) | 0.21 (-0.06 to 0.62) | 0.12 (0 to 0.25) |
| Croatia | Acute myeloid leukemia | DALYs | 0.114 (0.046 to 0.271) | 1.84 (0.75 to 4.34) | 0.353 (0.161 to 0.627) | 4.7 (2.21 to 8.27) | 2.11 (0.03 to 4.36) | 3.76 (3.39 to 4.13) |
| Croatia | Acute lymphoid leukemia | DALYs | 0.044 (0.02 to 0.081) | 0.75 (0.33 to 1.39) | 0.072 (0.035 to 0.123) | 1.13 (0.56 to 1.97) | 0.63 (-0.03 to 1.44) | 1.21 (0.97 to 1.46) |
| Croatia | Chronic myeloid leukemia | DALYs | 0.067 (0.031 to 0.117) | 1.06 (0.49 to 1.86) | 0.062 (0.029 to 0.11) | 0.81 (0.38 to 1.44) | -0.07 (-0.35 to 0.62) | -1.43 (-1.99 to -0.86) |
| Croatia | Chronic lymphoid leukemia | DALYs | 0.246 (0.117 to 0.413) | 3.75 (1.76 to 6.29) | 0.308 (0.151 to 0.533) | 3.42 (1.68 to 5.88) | 0.25 (-0.12 to 1.03) | 0.32 (-0.19 to 0.84) |
| Croatia | Other leukemia | DALYs | 0.302 (0.146 to 0.51) | 4.75 (2.29 to 8.11) | 0.14 (0.065 to 0.255) | 1.72 (0.81 to 3.1) | -0.53 (-0.67 to -0.18) | -3.83 (-3.99 to -3.67) |
| Cuba | Leukemia | death | 0.031 (0.014 to 0.053) | 0.29 (0.13 to 0.5) | 0.059 (0.027 to 0.102) | 0.33 (0.15 to 0.57) | 0.92 (0.5 to 1.48) | 0.68 (0.5 to 0.85) |
| Cuba | Acute myeloid leukemia | death | 0.009 (0.004 to 0.015) | 0.08 (0.04 to 0.14) | 0.02 (0.009 to 0.035) | 0.12 (0.06 to 0.2) | 1.32 (0.77 to 2.07) | 1.6 (1.41 to 1.8) |
| Cuba | Acute lymphoid leukemia | death | 0.003 (0.002 to 0.006) | 0.03 (0.01 to 0.05) | 0.006 (0.003 to 0.011) | 0.04 (0.02 to 0.06) | 0.8 (0.39 to 1.39) | 0.79 (0.66 to 0.91) |
| Cuba | Chronic myeloid leukemia | death | 0.008 (0.003 to 0.013) | 0.07 (0.03 to 0.13) | 0.011 (0.005 to 0.02) | 0.06 (0.03 to 0.11) | 0.52 (0.16 to 1.05) | -0.23 (-0.4 to -0.07) |
| Cuba | Chronic lymphoid leukemia | death | 0.004 (0.002 to 0.007) | 0.04 (0.02 to 0.07) | 0.01 (0.005 to 0.017) | 0.05 (0.02 to 0.09) | 1.4 (0.8 to 2.24) | 1.13 (0.91 to 1.35) |
| Cuba | Other leukemia | death | 0.007 (0.003 to 0.012) | 0.07 (0.03 to 0.12) | 0.011 (0.005 to 0.02) | 0.06 (0.03 to 0.11) | 0.63 (0.25 to 1.14) | -0.14 (-0.31 to 0.04) |
| Cuba | Leukemia | DALYs | 0.993 (0.46 to 1.692) | 9.12 (4.22 to 15.48) | 1.604 (0.768 to 2.73) | 9.96 (4.87 to 16.96) | 0.61 (0.25 to 1.11) | 0.35 (0.21 to 0.49) |
| Cuba | Acute myeloid leukemia | DALYs | 0.318 (0.147 to 0.546) | 2.88 (1.32 to 4.95) | 0.621 (0.292 to 1.056) | 4.02 (1.95 to 6.78) | 0.95 (0.46 to 1.62) | 1.26 (1.1 to 1.42) |
| Cuba | Acute lymphoid leukemia | DALYs | 0.136 (0.062 to 0.232) | 1.18 (0.55 to 2) | 0.203 (0.098 to 0.344) | 1.4 (0.69 to 2.39) | 0.49 (0.12 to 1.05) | 0.54 (0.42 to 0.66) |
| Cuba | Chronic myeloid leukemia | DALYs | 0.232 (0.106 to 0.398) | 2.17 (1 to 3.73) | 0.291 (0.138 to 0.5) | 1.73 (0.83 to 2.97) | 0.25 (-0.07 to 0.73) | -0.7 (-0.83 to -0.57) |
| Cuba | Chronic lymphoid leukemia | DALYs | 0.095 (0.042 to 0.164) | 0.91 (0.41 to 1.58) | 0.214 (0.102 to 0.375) | 1.16 (0.55 to 2.05) | 1.26 (0.65 to 2.11) | 0.98 (0.8 to 1.15) |
| Cuba | Other leukemia | DALYs | 0.213 (0.097 to 0.365) | 1.97 (0.9 to 3.37) | 0.277 (0.131 to 0.47) | 1.64 (0.8 to 2.78) | 0.3 (-0.02 to 0.76) | -0.72 (-0.88 to -0.56) |
| Cyprus | Leukemia | death | 0.002 (0.001 to 0.004) | 0.27 (0.11 to 0.52) | 0.007 (0.003 to 0.012) | 0.35 (0.16 to 0.64) | 2.09 (0.91 to 3.64) | 1.12 (0.9 to 1.35) |
| Cyprus | Acute myeloid leukemia | death | 0.001 (0 to 0.001) | 0.08 (0.03 to 0.15) | 0.003 (0.001 to 0.005) | 0.14 (0.06 to 0.25) | 3.09 (1.23 to 5.16) | 2.4 (2.14 to 2.66) |
| Cyprus | Acute lymphoid leukemia | death | 0 (0 to 0) | 0.02 (0.01 to 0.05) | 0 (0 to 0.001) | 0.02 (0.01 to 0.04) | 1.11 (0.45 to 2.2) | -0.31 (-0.59 to -0.02) |
| Cyprus | Chronic myeloid leukemia | death | 0 (0 to 0) | 0.01 (0.01 to 0.03) | 0 (0 to 0) | 0.01 (0 to 0.01) | 0.29 (-0.14 to 1.06) | -2.78 (-3.21 to -2.36) |
| Cyprus | Chronic lymphoid leukemia | death | 0 (0 to 0.001) | 0.05 (0.02 to 0.11) | 0.001 (0.001 to 0.003) | 0.08 (0.04 to 0.14) | 2.87 (1.54 to 4.88) | 1.87 (1.56 to 2.18) |
| Cyprus | Other leukemia | death | 0.001 (0 to 0.002) | 0.11 (0.04 to 0.22) | 0.002 (0.001 to 0.003) | 0.1 (0.04 to 0.19) | 1.34 (0.4 to 3.54) | 0.12 (-0.13 to 0.37) |
| Cyprus | Leukemia | DALYs | 0.051 (0.02 to 0.096) | 6.18 (2.43 to 11.61) | 0.142 (0.066 to 0.254) | 7.58 (3.49 to 13.46) | 1.8 (0.87 to 3.11) | 0.8 (0.61 to 1) |
| Cyprus | Acute myeloid leukemia | DALYs | 0.017 (0.007 to 0.032) | 1.99 (0.8 to 3.79) | 0.059 (0.027 to 0.107) | 3.11 (1.43 to 5.59) | 2.53 (1.1 to 4.25) | 1.81 (1.63 to 1.99) |
| Cyprus | Acute lymphoid leukemia | DALYs | 0.006 (0.002 to 0.012) | 0.7 (0.28 to 1.42) | 0.015 (0.007 to 0.027) | 0.84 (0.38 to 1.55) | 1.53 (0.77 to 2.85) | 0.44 (0.14 to 0.75) |
| Cyprus | Chronic myeloid leukemia | DALYs | 0.003 (0.001 to 0.005) | 0.32 (0.12 to 0.64) | 0.004 (0.002 to 0.006) | 0.2 (0.09 to 0.35) | 0.41 (-0.02 to 1.18) | -2.18 (-2.58 to -1.77) |
| Cyprus | Chronic lymphoid leukemia | DALYs | 0.008 (0.003 to 0.016) | 0.96 (0.38 to 1.95) | 0.03 (0.013 to 0.053) | 1.52 (0.69 to 2.69) | 2.77 (1.56 to 4.73) | 1.86 (1.55 to 2.18) |
| Cyprus | Other leukemia | DALYs | 0.018 (0.007 to 0.037) | 2.21 (0.81 to 4.58) | 0.035 (0.016 to 0.064) | 1.91 (0.85 to 3.47) | 0.98 (0.25 to 2.69) | -0.52 (-0.73 to -0.31) |
| Czechia | Leukemia | death | 0.08 (0.039 to 0.132) | 0.59 (0.28 to 0.98) | 0.124 (0.063 to 0.204) | 0.58 (0.29 to 0.95) | 0.54 (0.23 to 0.95) | -0.18 (-0.27 to -0.08) |
| Czechia | Acute myeloid leukemia | death | 0.023 (0.011 to 0.038) | 0.17 (0.08 to 0.28) | 0.043 (0.021 to 0.072) | 0.21 (0.1 to 0.34) | 0.87 (0.36 to 1.51) | 1.24 (1.02 to 1.46) |
| Czechia | Acute lymphoid leukemia | death | 0.012 (0.004 to 0.022) | 0.09 (0.03 to 0.16) | 0.006 (0.003 to 0.011) | 0.03 (0.02 to 0.06) | -0.49 (-0.65 to 0.55) | -3.49 (-3.95 to -3.01) |
| Czechia | Chronic myeloid leukemia | death | 0.007 (0.003 to 0.011) | 0.05 (0.02 to 0.09) | 0.005 (0.003 to 0.009) | 0.03 (0.01 to 0.04) | -0.19 (-0.38 to 0.08) | -3.15 (-3.72 to -2.58) |
| Czechia | Chronic lymphoid leukemia | death | 0.012 (0.006 to 0.021) | 0.09 (0.04 to 0.15) | 0.034 (0.018 to 0.056) | 0.15 (0.08 to 0.25) | 1.85 (0.99 to 2.81) | 1.83 (1.18 to 2.48) |
| Czechia | Other leukemia | death | 0.027 (0.013 to 0.045) | 0.2 (0.09 to 0.33) | 0.036 (0.018 to 0.06) | 0.16 (0.08 to 0.27) | 0.34 (0.01 to 0.72) | -1.32 (-1.69 to -0.94) |
| Czechia | Leukemia | DALYs | 1.975 (0.956 to 3.259) | 14.88 (7.13 to 24.62) | 2.559 (1.315 to 4.186) | 12.96 (6.64 to 21.19) | 0.3 (0.03 to 0.65) | -0.63 (-0.74 to -0.53) |
| Czechia | Acute myeloid leukemia | DALYs | 0.602 (0.277 to 1.012) | 4.6 (2.11 to 7.79) | 0.947 (0.473 to 1.565) | 5.05 (2.54 to 8.39) | 0.57 (0.17 to 1.11) | 0.78 (0.57 to 0.99) |
| Czechia | Acute lymphoid leukemia | DALYs | 0.308 (0.126 to 0.54) | 2.37 (0.99 to 4.15) | 0.173 (0.085 to 0.312) | 1 (0.48 to 1.83) | -0.44 (-0.62 to 0.58) | -2.83 (-3.22 to -2.44) |
| Czechia | Chronic myeloid leukemia | DALYs | 0.18 (0.086 to 0.304) | 1.4 (0.67 to 2.38) | 0.126 (0.065 to 0.21) | 0.71 (0.36 to 1.2) | -0.3 (-0.47 to -0.06) | -3.4 (-3.95 to -2.84) |
| Czechia | Chronic lymphoid leukemia | DALYs | 0.26 (0.123 to 0.439) | 1.86 (0.88 to 3.14) | 0.661 (0.35 to 1.084) | 3.07 (1.61 to 4.99) | 1.55 (0.76 to 2.42) | 1.51 (0.83 to 2.2) |
| Czechia | Other leukemia | DALYs | 0.625 (0.304 to 1.04) | 4.64 (2.28 to 7.75) | 0.65 (0.332 to 1.08) | 3.14 (1.58 to 5.21) | 0.04 (-0.2 to 0.35) | -2.12 (-2.52 to -1.72) |
| Côte d'Ivoire | Leukemia | DALYs | 0.214 (0.085 to 0.405) | 3.55 (1.42 to 6.64) | 0.844 (0.372 to 1.548) | 5.3 (2.36 to 9.62) | 2.94 (1.63 to 5.29) | 1.43 (1.35 to 1.51) |
| Côte d'Ivoire | Acute myeloid leukemia | DALYs | 0.04 (0.014 to 0.082) | 0.59 (0.22 to 1.2) | 0.174 (0.072 to 0.341) | 0.99 (0.41 to 1.97) | 3.38 (1.69 to 6.75) | 1.77 (1.66 to 1.87) |
| Côte d'Ivoire | Acute lymphoid leukemia | DALYs | 0.017 (0.006 to 0.037) | 0.22 (0.07 to 0.46) | 0.072 (0.026 to 0.143) | 0.34 (0.13 to 0.68) | 3.11 (1.31 to 6.62) | 1.52 (1.39 to 1.65) |
| Côte d'Ivoire | Chronic myeloid leukemia | DALYs | 0.059 (0.022 to 0.124) | 0.85 (0.32 to 1.79) | 0.204 (0.086 to 0.394) | 1.12 (0.47 to 2.17) | 2.46 (1.21 to 4.98) | 0.95 (0.79 to 1.1) |
| Côte d'Ivoire | Chronic lymphoid leukemia | DALYs | 0.021 (0.007 to 0.042) | 0.5 (0.17 to 1.02) | 0.082 (0.033 to 0.151) | 0.78 (0.31 to 1.47) | 3 (1.68 to 5.7) | 1.72 (1.64 to 1.8) |
| Côte d'Ivoire | Other leukemia | DALYs | 0.077 (0.029 to 0.153) | 1.38 (0.53 to 2.68) | 0.312 (0.132 to 0.594) | 2.06 (0.87 to 3.93) | 3.03 (1.64 to 5.7) | 1.45 (1.32 to 1.57) |
| Côte d'Ivoire | Leukemia | death | 0.006 (0.002 to 0.011) | 0.12 (0.05 to 0.22) | 0.023 (0.01 to 0.041) | 0.18 (0.08 to 0.32) | 2.92 (1.74 to 5.05) | 1.47 (1.4 to 1.53) |
| Côte d'Ivoire | Acute myeloid leukemia | death | 0.001 (0 to 0.002) | 0.02 (0.01 to 0.04) | 0.004 (0.002 to 0.009) | 0.03 (0.01 to 0.06) | 3.39 (1.74 to 6.63) | 1.88 (1.77 to 1.99) |
| Côte d'Ivoire | Acute lymphoid leukemia | death | 0 (0 to 0.001) | 0.01 (0 to 0.01) | 0.001 (0.001 to 0.003) | 0.01 (0 to 0.02) | 2.91 (1.3 to 5.97) | 1.24 (1.09 to 1.39) |
| Côte d'Ivoire | Chronic myeloid leukemia | death | 0.001 (0.001 to 0.003) | 0.02 (0.01 to 0.05) | 0.005 (0.002 to 0.009) | 0.03 (0.01 to 0.06) | 2.46 (1.24 to 4.93) | 1 (0.87 to 1.14) |
| Côte d'Ivoire | Chronic lymphoid leukemia | death | 0.001 (0 to 0.002) | 0.02 (0.01 to 0.04) | 0.003 (0.001 to 0.006) | 0.03 (0.01 to 0.07) | 3.11 (1.81 to 5.69) | 1.77 (1.69 to 1.86) |
| Côte d'Ivoire | Other leukemia | death | 0.002 (0.001 to 0.004) | 0.05 (0.02 to 0.1) | 0.009 (0.004 to 0.016) | 0.07 (0.03 to 0.14) | 2.95 (1.71 to 5.35) | 1.42 (1.32 to 1.53) |
| Democratic People's Republic of Korea | Leukemia | death | 0.015 (0.002 to 0.041) | 0.08 (0.01 to 0.23) | 0.022 (0.004 to 0.059) | 0.07 (0.01 to 0.18) | 0.53 (0.06 to 1.26) | -0.56 (-0.58 to -0.54) |
| Democratic People's Republic of Korea | Acute myeloid leukemia | death | 0.001 (0 to 0.004) | 0.01 (0 to 0.02) | 0.002 (0 to 0.006) | 0.01 (0 to 0.02) | 0.91 (0.2 to 2.17) | 0.22 (0.13 to 0.31) |
| Democratic People's Republic of Korea | Acute lymphoid leukemia | death | 0.001 (0 to 0.004) | 0.01 (0 to 0.02) | 0.002 (0 to 0.007) | 0.01 (0 to 0.02) | 1.12 (0.25 to 2.93) | 0.8 (0.71 to 0.89) |
| Democratic People's Republic of Korea | Chronic myeloid leukemia | death | 0.001 (0 to 0.002) | 0 (0 to 0.01) | 0.001 (0 to 0.002) | 0 (0 to 0.01) | 0.14 (-0.27 to 0.9) | -1.84 (-1.96 to -1.71) |
| Democratic People's Republic of Korea | Chronic lymphoid leukemia | death | 0 (0 to 0.002) | 0 (0 to 0.01) | 0.001 (0 to 0.003) | 0 (0 to 0.01) | 1.08 (0.39 to 2.45) | 0.54 (0.4 to 0.69) |
| Democratic People's Republic of Korea | Other leukemia | death | 0.011 (0.002 to 0.032) | 0.06 (0.01 to 0.17) | 0.016 (0.003 to 0.042) | 0.05 (0.01 to 0.13) | 0.42 (0.01 to 1.1) | -0.82 (-0.86 to -0.78) |
| Democratic People's Republic of Korea | Leukemia | DALYs | 0.56 (0.078 to 1.602) | 2.83 (0.4 to 8) | 0.763 (0.127 to 2) | 2.39 (0.4 to 6.32) | 0.36 (-0.09 to 1.08) | -0.63 (-0.66 to -0.61) |
| Democratic People's Republic of Korea | Acute myeloid leukemia | DALYs | 0.044 (0.006 to 0.131) | 0.22 (0.03 to 0.67) | 0.075 (0.012 to 0.213) | 0.23 (0.04 to 0.68) | 0.71 (0.04 to 1.99) | 0.2 (0.09 to 0.31) |
| Democratic People's Republic of Korea | Acute lymphoid leukemia | DALYs | 0.044 (0.006 to 0.15) | 0.22 (0.03 to 0.75) | 0.084 (0.012 to 0.248) | 0.26 (0.04 to 0.78) | 0.91 (0.09 to 2.67) | 0.76 (0.69 to 0.84) |
| Democratic People's Republic of Korea | Chronic myeloid leukemia | DALYs | 0.021 (0.003 to 0.065) | 0.1 (0.01 to 0.33) | 0.021 (0.003 to 0.063) | 0.07 (0.01 to 0.2) | 0.03 (-0.36 to 0.76) | -1.87 (-2.01 to -1.72) |
| Democratic People's Republic of Korea | Chronic lymphoid leukemia | DALYs | 0.017 (0.002 to 0.053) | 0.09 (0.01 to 0.27) | 0.032 (0.005 to 0.091) | 0.1 (0.02 to 0.28) | 0.88 (0.22 to 2.19) | 0.55 (0.39 to 0.72) |
| Democratic People's Republic of Korea | Other leukemia | DALYs | 0.435 (0.06 to 1.288) | 2.2 (0.31 to 6.4) | 0.552 (0.094 to 1.495) | 1.73 (0.29 to 4.68) | 0.27 (-0.14 to 0.93) | -0.9 (-0.93 to -0.87) |
| Democratic Republic of the Congo | Leukemia | death | 0.016 (0.006 to 0.03) | 0.09 (0.04 to 0.18) | 0.032 (0.012 to 0.066) | 0.08 (0.03 to 0.17) | 1.06 (0.44 to 1.85) | -1.09 (-1.58 to -0.6) |
| Democratic Republic of the Congo | Acute myeloid leukemia | death | 0.002 (0.001 to 0.004) | 0.01 (0 to 0.02) | 0.004 (0.001 to 0.009) | 0.01 (0 to 0.02) | 1.36 (0.36 to 2.87) | -0.76 (-1.49 to -0.02) |
| Democratic Republic of the Congo | Acute lymphoid leukemia | death | 0.001 (0 to 0.001) | 0 (0 to 0.01) | 0.002 (0.001 to 0.003) | 0 (0 to 0.01) | 1.43 (0.63 to 2.72) | -0.84 (-1.44 to -0.23) |
| Democratic Republic of the Congo | Chronic myeloid leukemia | death | 0.002 (0.001 to 0.005) | 0.01 (0 to 0.03) | 0.005 (0.002 to 0.01) | 0.01 (0 to 0.02) | 0.83 (0.14 to 2.06) | -1.5 (-2.15 to -0.84) |
| Democratic Republic of the Congo | Chronic lymphoid leukemia | death | 0.002 (0.001 to 0.004) | 0.01 (0 to 0.02) | 0.007 (0.002 to 0.016) | 0.02 (0.01 to 0.05) | 3.22 (1.52 to 5.9) | 2.01 (1.77 to 2.24) |
| Democratic Republic of the Congo | Other leukemia | death | 0.009 (0.003 to 0.018) | 0.06 (0.02 to 0.12) | 0.015 (0.005 to 0.031) | 0.04 (0.01 to 0.08) | 0.63 (0.13 to 1.3) | -2.15 (-2.63 to -1.68) |
| Democratic Republic of the Congo | Leukemia | DALYs | 0.528 (0.197 to 1.027) | 2.62 (1 to 5.03) | 1.099 (0.393 to 2.284) | 2.32 (0.85 to 4.77) | 1.08 (0.45 to 1.91) | -1.07 (-1.56 to -0.57) |
| Democratic Republic of the Congo | Acute myeloid leukemia | DALYs | 0.062 (0.023 to 0.13) | 0.29 (0.11 to 0.6) | 0.153 (0.052 to 0.315) | 0.3 (0.11 to 0.62) | 1.46 (0.42 to 3.02) | -0.67 (-1.4 to 0.07) |
| Democratic Republic of the Congo | Acute lymphoid leukemia | DALYs | 0.026 (0.008 to 0.061) | 0.11 (0.04 to 0.25) | 0.068 (0.022 to 0.154) | 0.11 (0.04 to 0.25) | 1.62 (0.71 to 3.17) | -0.57 (-1.17 to 0.04) |
| Democratic Republic of the Congo | Chronic myeloid leukemia | DALYs | 0.089 (0.03 to 0.195) | 0.42 (0.14 to 0.89) | 0.168 (0.057 to 0.356) | 0.33 (0.11 to 0.69) | 0.88 (0.14 to 2.25) | -1.42 (-2.06 to -0.77) |
| Democratic Republic of the Congo | Chronic lymphoid leukemia | DALYs | 0.046 (0.016 to 0.101) | 0.27 (0.09 to 0.58) | 0.192 (0.061 to 0.416) | 0.51 (0.16 to 1.14) | 3.2 (1.53 to 5.93) | 1.97 (1.74 to 2.2) |
| Democratic Republic of the Congo | Other leukemia | DALYs | 0.305 (0.105 to 0.627) | 1.53 (0.54 to 3.11) | 0.518 (0.177 to 1.097) | 1.07 (0.37 to 2.27) | 0.7 (0.16 to 1.44) | -1.98 (-2.43 to -1.52) |
| Denmark | Leukemia | death | 0.035 (0.016 to 0.062) | 0.43 (0.2 to 0.78) | 0.043 (0.021 to 0.073) | 0.36 (0.18 to 0.62) | 0.24 (0.06 to 0.52) | -0.38 (-0.55 to -0.2) |
| Denmark | Acute myeloid leukemia | death | 0.016 (0.007 to 0.028) | 0.2 (0.09 to 0.36) | 0.021 (0.01 to 0.036) | 0.18 (0.09 to 0.31) | 0.31 (0.11 to 0.66) | 0.16 (-0.06 to 0.39) |
| Denmark | Acute lymphoid leukemia | death | 0.001 (0.001 to 0.002) | 0.02 (0.01 to 0.03) | 0.001 (0.001 to 0.002) | 0.02 (0.01 to 0.03) | 0.22 (-0.04 to 0.62) | -0.55 (-0.66 to -0.44) |
| Denmark | Chronic myeloid leukemia | death | 0.006 (0.003 to 0.01) | 0.07 (0.03 to 0.13) | 0.002 (0.001 to 0.004) | 0.02 (0.01 to 0.04) | -0.58 (-0.66 to -0.44) | -5.13 (-5.65 to -4.61) |
| Denmark | Chronic lymphoid leukemia | death | 0.006 (0.003 to 0.011) | 0.08 (0.03 to 0.14) | 0.01 (0.005 to 0.018) | 0.08 (0.04 to 0.14) | 0.6 (0.27 to 1.13) | 0.24 (-0.04 to 0.52) |
| Denmark | Other leukemia | death | 0.005 (0.002 to 0.01) | 0.07 (0.03 to 0.12) | 0.008 (0.004 to 0.014) | 0.06 (0.03 to 0.11) | 0.46 (0.15 to 0.93) | 0.62 (0.16 to 1.08) |
| Denmark | Leukemia | DALYs | 0.791 (0.355 to 1.409) | 10.9 (4.9 to 19.5) | 0.839 (0.404 to 1.415) | 8.22 (4 to 13.83) | 0.06 (-0.09 to 0.29) | -0.89 (-1 to -0.77) |
| Denmark | Acute myeloid leukemia | DALYs | 0.374 (0.164 to 0.679) | 5.22 (2.28 to 9.47) | 0.423 (0.204 to 0.725) | 4.29 (2.08 to 7.26) | 0.13 (-0.04 to 0.43) | -0.34 (-0.52 to -0.17) |
| Denmark | Acute lymphoid leukemia | DALYs | 0.039 (0.017 to 0.072) | 0.62 (0.26 to 1.14) | 0.05 (0.023 to 0.085) | 0.64 (0.31 to 1.1) | 0.27 (-0.04 to 0.74) | -0.1 (-0.23 to 0.02) |
| Denmark | Chronic myeloid leukemia | DALYs | 0.14 (0.061 to 0.249) | 1.98 (0.86 to 3.55) | 0.053 (0.025 to 0.09) | 0.54 (0.25 to 0.92) | -0.62 (-0.69 to -0.5) | -5.33 (-5.82 to -4.85) |
| Denmark | Chronic lymphoid leukemia | DALYs | 0.126 (0.056 to 0.226) | 1.59 (0.7 to 2.84) | 0.181 (0.085 to 0.317) | 1.54 (0.73 to 2.69) | 0.44 (0.14 to 0.92) | -0.24 (-0.49 to 0) |
| Denmark | Other leukemia | DALYs | 0.112 (0.049 to 0.2) | 1.5 (0.65 to 2.71) | 0.132 (0.061 to 0.228) | 1.21 (0.57 to 2.08) | 0.19 (-0.05 to 0.52) | -0.13 (-0.52 to 0.26) |
| Djibouti | Leukemia | death | 0 (0 to 0) | 0.04 (0.01 to 0.1) | 0.001 (0 to 0.002) | 0.12 (0.05 to 0.23) | 11.57 (6.27 to 33.23) | 4.7 (4.46 to 4.93) |
| Djibouti | Acute myeloid leukemia | death | 0 (0 to 0) | 0 (0 to 0.01) | 0 (0 to 0) | 0.01 (0.01 to 0.03) | 14.26 (6.95 to 44.46) | 5.48 (5.23 to 5.74) |
| Djibouti | Acute lymphoid leukemia | death | 0 (0 to 0) | 0 (0 to 0) | 0 (0 to 0) | 0.01 (0 to 0.01) | 12.28 (5.89 to 38.02) | 5.21 (4.97 to 5.45) |
| Djibouti | Chronic myeloid leukemia | death | 0 (0 to 0) | 0.01 (0 to 0.02) | 0 (0 to 0) | 0.02 (0.01 to 0.04) | 9.92 (4.76 to 29.73) | 4.23 (3.99 to 4.46) |
| Djibouti | Chronic lymphoid leukemia | death | 0 (0 to 0) | 0.01 (0 to 0.03) | 0 (0 to 0) | 0.04 (0.01 to 0.08) | 18.02 (9.73 to 51.98) | 5.77 (5.45 to 6.1) |
| Djibouti | Other leukemia | death | 0 (0 to 0) | 0.01 (0 to 0.04) | 0 (0 to 0) | 0.03 (0.01 to 0.07) | 8.87 (4.46 to 26.04) | 3.7 (3.51 to 3.9) |
| Djibouti | Leukemia | DALYs | 0.002 (0 to 0.006) | 1.04 (0.2 to 2.71) | 0.027 (0.01 to 0.056) | 3.26 (1.25 to 6.53) | 10.52 (5.34 to 30.72) | 4.57 (4.35 to 4.79) |
| Djibouti | Acute myeloid leukemia | DALYs | 0 (0 to 0.001) | 0.13 (0.02 to 0.39) | 0.005 (0.002 to 0.011) | 0.5 (0.18 to 1.1) | 13.39 (6.36 to 41.6) | 5.45 (5.2 to 5.7) |
| Djibouti | Acute lymphoid leukemia | DALYs | 0 (0 to 0.001) | 0.06 (0.01 to 0.16) | 0.002 (0.001 to 0.005) | 0.22 (0.08 to 0.48) | 11.36 (5.08 to 36.33) | 5.22 (4.98 to 5.45) |
| Djibouti | Chronic myeloid leukemia | DALYs | 0.001 (0 to 0.002) | 0.25 (0.04 to 0.71) | 0.007 (0.002 to 0.014) | 0.69 (0.24 to 1.49) | 9.3 (4.39 to 28.59) | 4.12 (3.89 to 4.35) |
| Djibouti | Chronic lymphoid leukemia | DALYs | 0 (0 to 0.001) | 0.2 (0.04 to 0.55) | 0.005 (0.002 to 0.01) | 0.84 (0.31 to 1.73) | 18.18 (9.48 to 52.46) | 5.98 (5.64 to 6.32) |
| Djibouti | Other leukemia | DALYs | 0.001 (0 to 0.003) | 0.4 (0.08 to 1.07) | 0.009 (0.003 to 0.018) | 1.01 (0.36 to 2.11) | 8.02 (3.84 to 24.19) | 3.56 (3.35 to 3.77) |
| Dominica | Leukemia | death | 0 (0 to 0.001) | 0.41 (0.18 to 0.74) | 0 (0 to 0.001) | 0.57 (0.28 to 0.99) | 0.7 (0.32 to 1.36) | 1.11 (0.97 to 1.26) |
| Dominica | Acute myeloid leukemia | death | 0 (0 to 0) | 0.08 (0.03 to 0.15) | 0 (0 to 0) | 0.13 (0.05 to 0.23) | 1.03 (0.44 to 2.13) | 1.74 (1.57 to 1.91) |
| Dominica | Acute lymphoid leukemia | death | 0 (0 to 0) | 0.04 (0.02 to 0.08) | 0 (0 to 0) | 0.07 (0.03 to 0.12) | 1.07 (0.45 to 2.22) | 1.98 (1.82 to 2.14) |
| Dominica | Chronic myeloid leukemia | death | 0 (0 to 0) | 0.03 (0.01 to 0.05) | 0 (0 to 0) | 0.04 (0.02 to 0.08) | 0.91 (0.25 to 2.01) | 1.2 (1.06 to 1.34) |
| Dominica | Chronic lymphoid leukemia | death | 0 (0 to 0) | 0.02 (0.01 to 0.04) | 0 (0 to 0) | 0.04 (0.02 to 0.07) | 1.23 (0.57 to 2.47) | 2.19 (2.05 to 2.33) |
| Dominica | Other leukemia | death | 0 (0 to 0) | 0.24 (0.1 to 0.44) | 0 (0 to 0) | 0.29 (0.14 to 0.51) | 0.47 (0.12 to 1.08) | 0.56 (0.4 to 0.73) |
| Dominica | Leukemia | DALYs | 0.008 (0.004 to 0.015) | 12.58 (5.55 to 22.17) | 0.014 (0.007 to 0.024) | 16.99 (8.35 to 29.42) | 0.67 (0.26 to 1.36) | 0.97 (0.78 to 1.16) |
| Dominica | Acute myeloid leukemia | DALYs | 0.002 (0.001 to 0.003) | 2.81 (1.15 to 5.28) | 0.004 (0.002 to 0.006) | 4.46 (1.91 to 7.99) | 0.96 (0.39 to 1.97) | 1.64 (1.43 to 1.85) |
| Dominica | Acute lymphoid leukemia | DALYs | 0.001 (0 to 0.002) | 1.53 (0.64 to 2.97) | 0.002 (0.001 to 0.003) | 2.66 (1.28 to 4.58) | 0.99 (0.34 to 2.2) | 1.91 (1.66 to 2.15) |
| Dominica | Chronic myeloid leukemia | DALYs | 0.001 (0 to 0.001) | 0.86 (0.39 to 1.5) | 0.001 (0 to 0.002) | 1.24 (0.59 to 2.21) | 0.86 (0.22 to 1.91) | 0.97 (0.77 to 1.17) |
| Dominica | Chronic lymphoid leukemia | DALYs | 0 (0 to 0.001) | 0.49 (0.22 to 0.89) | 0.001 (0 to 0.001) | 0.84 (0.41 to 1.46) | 1.22 (0.53 to 2.54) | 1.98 (1.83 to 2.14) |
| Dominica | Other leukemia | DALYs | 0.005 (0.002 to 0.008) | 6.9 (2.99 to 12.58) | 0.006 (0.003 to 0.011) | 7.78 (3.77 to 13.82) | 0.41 (0.06 to 1.03) | 0.29 (0.09 to 0.48) |
| Dominican Republic | Leukemia | death | 0.005 (0.002 to 0.01) | 0.12 (0.04 to 0.24) | 0.026 (0.011 to 0.05) | 0.27 (0.12 to 0.5) | 4.15 (2.21 to 8.57) | 3.61 (3.33 to 3.89) |
| Dominican Republic | Acute myeloid leukemia | death | 0.001 (0 to 0.001) | 0.01 (0 to 0.03) | 0.006 (0.003 to 0.011) | 0.06 (0.02 to 0.11) | 7.93 (3.75 to 16.85) | 6.13 (5.72 to 6.54) |
| Dominican Republic | Acute lymphoid leukemia | death | 0 (0 to 0.001) | 0.01 (0 to 0.01) | 0.002 (0.001 to 0.004) | 0.02 (0.01 to 0.04) | 5.4 (2.59 to 11.93) | 4.81 (4.49 to 5.13) |
| Dominican Republic | Chronic myeloid leukemia | death | 0 (0 to 0) | 0.01 (0 to 0.01) | 0.001 (0.001 to 0.003) | 0.02 (0.01 to 0.03) | 5.56 (2.92 to 11.62) | 4.68 (4.34 to 5.03) |
| Dominican Republic | Chronic lymphoid leukemia | death | 0 (0 to 0) | 0 (0 to 0.01) | 0.001 (0.001 to 0.003) | 0.02 (0.01 to 0.03) | 7.87 (4.44 to 15.63) | 5.78 (5.29 to 6.27) |
| Dominican Republic | Other leukemia | death | 0.004 (0.001 to 0.008) | 0.09 (0.03 to 0.18) | 0.016 (0.006 to 0.03) | 0.16 (0.07 to 0.31) | 3.14 (1.57 to 6.79) | 2.66 (2.41 to 2.9) |
| Dominican Republic | Leukemia | DALYs | 0.192 (0.063 to 0.387) | 3.86 (1.31 to 7.7) | 0.912 (0.402 to 1.716) | 8.75 (3.86 to 16.44) | 3.76 (1.86 to 8.17) | 3.59 (3.31 to 3.88) |
| Dominican Republic | Acute myeloid leukemia | DALYs | 0.026 (0.008 to 0.054) | 0.5 (0.17 to 1.03) | 0.218 (0.096 to 0.409) | 2.05 (0.9 to 3.85) | 7.47 (3.48 to 16.49) | 6.2 (5.78 to 6.62) |
| Dominican Republic | Acute lymphoid leukemia | DALYs | 0.015 (0.004 to 0.033) | 0.25 (0.08 to 0.56) | 0.085 (0.037 to 0.162) | 0.78 (0.34 to 1.5) | 4.81 (2.26 to 11.65) | 4.8 (4.46 to 5.14) |
| Dominican Republic | Chronic myeloid leukemia | DALYs | 0.008 (0.003 to 0.017) | 0.17 (0.06 to 0.34) | 0.05 (0.021 to 0.097) | 0.48 (0.21 to 0.93) | 5.05 (2.4 to 11.36) | 4.63 (4.28 to 4.98) |
| Dominican Republic | Chronic lymphoid leukemia | DALYs | 0.004 (0.002 to 0.009) | 0.11 (0.04 to 0.22) | 0.037 (0.016 to 0.068) | 0.38 (0.17 to 0.71) | 7.24 (3.9 to 14.83) | 5.69 (5.22 to 6.17) |
| Dominican Republic | Other leukemia | DALYs | 0.138 (0.047 to 0.281) | 2.83 (0.98 to 5.67) | 0.523 (0.22 to 1.003) | 5.05 (2.11 to 9.66) | 2.78 (1.24 to 6.47) | 2.57 (2.32 to 2.82) |
| Ecuador | Leukemia | death | 0.021 (0.01 to 0.035) | 0.33 (0.15 to 0.55) | 0.081 (0.04 to 0.138) | 0.52 (0.26 to 0.89) | 2.97 (1.75 to 4.5) | 1.8 (1.59 to 2) |
| Ecuador | Acute myeloid leukemia | death | 0.004 (0.002 to 0.007) | 0.06 (0.03 to 0.1) | 0.026 (0.012 to 0.045) | 0.16 (0.08 to 0.28) | 6.11 (2.37 to 9.95) | 4.28 (3.97 to 4.59) |
| Ecuador | Acute lymphoid leukemia | death | 0.004 (0.002 to 0.007) | 0.05 (0.02 to 0.1) | 0.018 (0.008 to 0.031) | 0.11 (0.05 to 0.19) | 4.03 (1.58 to 6.95) | 2.91 (2.66 to 3.16) |
| Ecuador | Chronic myeloid leukemia | death | 0.002 (0.001 to 0.003) | 0.03 (0.01 to 0.04) | 0.007 (0.003 to 0.011) | 0.04 (0.02 to 0.07) | 3.2 (2.05 to 4.95) | 2.52 (2.15 to 2.88) |
| Ecuador | Chronic lymphoid leukemia | death | 0 (0 to 0.001) | 0.01 (0 to 0.02) | 0.004 (0.002 to 0.007) | 0.03 (0.01 to 0.05) | 6.86 (4.48 to 10.52) | 4.31 (3.82 to 4.8) |
| Ecuador | Other leukemia | death | 0.011 (0.005 to 0.02) | 0.18 (0.08 to 0.32) | 0.028 (0.013 to 0.049) | 0.18 (0.09 to 0.32) | 1.44 (0.84 to 2.38) | -0.22 (-0.53 to 0.08) |
| Ecuador | Leukemia | DALYs | 0.791 (0.368 to 1.351) | 10.89 (5.18 to 18.27) | 2.776 (1.393 to 4.651) | 16.57 (8.3 to 27.73) | 2.51 (1.38 to 3.9) | 1.49 (1.29 to 1.69) |
| Ecuador | Acute myeloid leukemia | DALYs | 0.137 (0.064 to 0.248) | 1.92 (0.9 to 3.45) | 0.865 (0.428 to 1.523) | 5.19 (2.55 to 9.08) | 5.29 (2.03 to 8.66) | 4 (3.73 to 4.28) |
| Ecuador | Acute lymphoid leukemia | DALYs | 0.154 (0.068 to 0.296) | 1.94 (0.89 to 3.71) | 0.726 (0.337 to 1.275) | 4.16 (1.95 to 7.25) | 3.73 (1.35 to 6.75) | 2.88 (2.64 to 3.12) |
| Ecuador | Chronic myeloid leukemia | DALYs | 0.057 (0.027 to 0.099) | 0.82 (0.39 to 1.41) | 0.21 (0.104 to 0.35) | 1.27 (0.63 to 2.11) | 2.66 (1.56 to 4.33) | 2.08 (1.76 to 2.41) |
| Ecuador | Chronic lymphoid leukemia | DALYs | 0.013 (0.006 to 0.023) | 0.22 (0.1 to 0.39) | 0.088 (0.044 to 0.151) | 0.57 (0.29 to 0.98) | 5.69 (3.61 to 8.77) | 3.68 (3.27 to 4.09) |
| Ecuador | Other leukemia | DALYs | 0.43 (0.197 to 0.731) | 5.98 (2.77 to 10.1) | 0.887 (0.431 to 1.594) | 5.38 (2.66 to 9.59) | 1.06 (0.51 to 1.93) | -0.74 (-1.11 to -0.37) |
| Egypt | Leukemia | death | 0.095 (0.043 to 0.164) | 0.28 (0.12 to 0.5) | 0.3 (0.141 to 0.509) | 0.42 (0.19 to 0.73) | 2.17 (1.14 to 3.69) | 1.43 (1.31 to 1.56) |
| Egypt | Acute myeloid leukemia | death | 0.021 (0.009 to 0.038) | 0.06 (0.02 to 0.1) | 0.078 (0.038 to 0.134) | 0.1 (0.05 to 0.17) | 2.79 (1.56 to 4.93) | 1.79 (1.66 to 1.93) |
| Egypt | Acute lymphoid leukemia | death | 0.009 (0.004 to 0.018) | 0.02 (0.01 to 0.05) | 0.032 (0.014 to 0.061) | 0.04 (0.02 to 0.08) | 2.38 (1.24 to 4.24) | 1.6 (1.52 to 1.68) |
| Egypt | Chronic myeloid leukemia | death | 0.02 (0.008 to 0.035) | 0.05 (0.02 to 0.1) | 0.049 (0.022 to 0.09) | 0.07 (0.03 to 0.12) | 1.51 (0.62 to 2.89) | 0.59 (0.45 to 0.72) |
| Egypt | Chronic lymphoid leukemia | death | 0.003 (0.001 to 0.005) | 0.01 (0 to 0.02) | 0.015 (0.007 to 0.029) | 0.02 (0.01 to 0.04) | 4.34 (2.36 to 7.62) | 3.09 (2.87 to 3.32) |
| Egypt | Other leukemia | death | 0.042 (0.016 to 0.089) | 0.14 (0.05 to 0.3) | 0.126 (0.05 to 0.25) | 0.19 (0.08 to 0.41) | 1.99 (0.91 to 3.99) | 1.4 (1.25 to 1.55) |
| Egypt | Leukemia | DALYs | 3.541 (1.639 to 6.032) | 9.06 (4.18 to 15.32) | 10.675 (5.227 to 18.193) | 13.06 (6.24 to 22.2) | 2.01 (1.06 to 3.48) | 1.29 (1.21 to 1.36) |
| Egypt | Acute myeloid leukemia | DALYs | 0.819 (0.335 to 1.522) | 2.01 (0.84 to 3.72) | 3.043 (1.441 to 5.341) | 3.51 (1.72 to 6.1) | 2.72 (1.53 to 4.82) | 1.78 (1.69 to 1.88) |
| Egypt | Acute lymphoid leukemia | DALYs | 0.426 (0.165 to 0.825) | 0.96 (0.38 to 1.85) | 1.383 (0.63 to 2.617) | 1.51 (0.68 to 2.88) | 2.25 (1.12 to 4.14) | 1.51 (1.45 to 1.57) |
| Egypt | Chronic myeloid leukemia | DALYs | 0.763 (0.307 to 1.42) | 1.91 (0.81 to 3.46) | 1.769 (0.795 to 3.237) | 2.13 (0.98 to 3.88) | 1.32 (0.51 to 2.68) | 0.36 (0.27 to 0.44) |
| Egypt | Chronic lymphoid leukemia | DALYs | 0.093 (0.037 to 0.171) | 0.26 (0.11 to 0.48) | 0.474 (0.202 to 0.925) | 0.63 (0.27 to 1.21) | 4.1 (2.28 to 7.21) | 3 (2.81 to 3.19) |
| Egypt | Other leukemia | DALYs | 1.44 (0.561 to 2.896) | 3.93 (1.51 to 8.11) | 4.006 (1.625 to 7.676) | 5.28 (2.16 to 10.44) | 1.78 (0.77 to 3.85) | 1.2 (1.1 to 1.31) |
| El Salvador | Leukemia | death | 0.008 (0.003 to 0.014) | 0.23 (0.09 to 0.43) | 0.024 (0.011 to 0.044) | 0.4 (0.18 to 0.73) | 2.17 (1.18 to 3.79) | 1.95 (1.66 to 2.24) |
| El Salvador | Acute myeloid leukemia | death | 0 (0 to 0.001) | 0.01 (0 to 0.02) | 0.003 (0.001 to 0.006) | 0.05 (0.02 to 0.1) | 6.95 (2.26 to 12.94) | 6.23 (5.8 to 6.65) |
| El Salvador | Acute lymphoid leukemia | death | 0 (0 to 0.001) | 0.01 (0 to 0.03) | 0.003 (0.001 to 0.005) | 0.04 (0.02 to 0.08) | 6.62 (1.38 to 15.54) | 6.53 (6.1 to 6.96) |
| El Salvador | Chronic myeloid leukemia | death | 0 (0 to 0) | 0.01 (0 to 0.01) | 0.001 (0 to 0.002) | 0.02 (0.01 to 0.03) | 4.35 (2.36 to 7.58) | 4.35 (3.9 to 4.79) |
| El Salvador | Chronic lymphoid leukemia | death | 0 (0 to 0) | 0 (0 to 0.01) | 0.001 (0 to 0.001) | 0.01 (0.01 to 0.02) | 7.34 (4.41 to 12.37) | 5.32 (5.02 to 5.63) |
| El Salvador | Other leukemia | death | 0.007 (0.002 to 0.012) | 0.2 (0.07 to 0.37) | 0.016 (0.007 to 0.03) | 0.27 (0.12 to 0.5) | 1.5 (0.78 to 2.7) | 0.89 (0.47 to 1.31) |
| El Salvador | Leukemia | DALYs | 0.288 (0.108 to 0.54) | 7.77 (2.96 to 14.52) | 0.777 (0.35 to 1.433) | 12.77 (5.75 to 23.53) | 1.7 (0.82 to 3.19) | 1.72 (1.42 to 2.02) |
| El Salvador | Acute myeloid leukemia | DALYs | 0.015 (0.005 to 0.031) | 0.41 (0.16 to 0.82) | 0.105 (0.043 to 0.2) | 1.73 (0.71 to 3.26) | 6.05 (2 to 11.7) | 6.02 (5.63 to 6.41) |
| El Salvador | Acute lymphoid leukemia | DALYs | 0.015 (0.005 to 0.045) | 0.38 (0.12 to 1.13) | 0.105 (0.04 to 0.204) | 1.69 (0.63 to 3.29) | 5.93 (1.17 to 14.89) | 6.57 (6.11 to 7.03) |
| El Salvador | Chronic myeloid leukemia | DALYs | 0.007 (0.003 to 0.014) | 0.2 (0.08 to 0.39) | 0.03 (0.014 to 0.055) | 0.51 (0.23 to 0.92) | 3.34 (1.76 to 6.1) | 3.82 (3.42 to 4.23) |
| El Salvador | Chronic lymphoid leukemia | DALYs | 0.003 (0.001 to 0.005) | 0.08 (0.03 to 0.16) | 0.018 (0.008 to 0.033) | 0.31 (0.14 to 0.57) | 5.94 (3.48 to 9.94) | 5.01 (4.73 to 5.3) |
| El Salvador | Other leukemia | DALYs | 0.248 (0.092 to 0.464) | 6.71 (2.56 to 12.49) | 0.518 (0.232 to 0.961) | 8.53 (3.84 to 15.82) | 1.09 (0.43 to 2.23) | 0.59 (0.15 to 1.03) |
| Equatorial Guinea | Leukemia | death | 0 (0 to 0) | 0.05 (0.01 to 0.12) | 0.001 (0.001 to 0.003) | 0.24 (0.11 to 0.46) | 12.21 (4.56 to 47.45) | 7.26 (6.61 to 7.92) |
| Equatorial Guinea | Acute myeloid leukemia | death | 0 (0 to 0) | 0 (0 to 0.01) | 0 (0 to 0.001) | 0.04 (0.02 to 0.09) | 28.62 (9.91 to 116.47) | 10.27 (9.28 to 11.26) |
| Equatorial Guinea | Acute lymphoid leukemia | death | 0 (0 to 0) | 0 (0 to 0) | 0 (0 to 0) | 0.01 (0 to 0.02) | 17.75 (6.16 to 81.65) | 7.31 (6.74 to 7.88) |
| Equatorial Guinea | Chronic myeloid leukemia | death | 0 (0 to 0) | 0.01 (0 to 0.02) | 0 (0 to 0.001) | 0.03 (0.01 to 0.08) | 10.6 (3.11 to 40.87) | 6.52 (5.96 to 7.09) |
| Equatorial Guinea | Chronic lymphoid leukemia | death | 0 (0 to 0) | 0.01 (0 to 0.02) | 0 (0 to 0.001) | 0.09 (0.03 to 0.18) | 32.46 (13.65 to 118.74) | 11.98 (10.75 to 13.22) |
| Equatorial Guinea | Other leukemia | death | 0 (0 to 0) | 0.03 (0.01 to 0.08) | 0 (0 to 0.001) | 0.07 (0.03 to 0.14) | 5.99 (1.99 to 25.38) | 4.12 (3.69 to 4.54) |
| Equatorial Guinea | Leukemia | DALYs | 0.004 (0.001 to 0.009) | 1.45 (0.29 to 3.7) | 0.05 (0.021 to 0.1) | 6.67 (2.81 to 13.02) | 12.83 (4.58 to 49.47) | 6.84 (6.18 to 7.51) |
| Equatorial Guinea | Acute myeloid leukemia | DALYs | 0 (0 to 0.001) | 0.14 (0.02 to 0.38) | 0.012 (0.004 to 0.026) | 1.36 (0.52 to 2.98) | 32.95 (10.92 to 138.03) | 10.33 (9.31 to 11.36) |
| Equatorial Guinea | Acute lymphoid leukemia | DALYs | 0 (0 to 0.001) | 0.06 (0.01 to 0.17) | 0.004 (0.001 to 0.008) | 0.34 (0.14 to 0.68) | 21.46 (7.21 to 106.77) | 7.62 (7 to 8.24) |
| Equatorial Guinea | Chronic myeloid leukemia | DALYs | 0.001 (0 to 0.002) | 0.27 (0.05 to 0.75) | 0.009 (0.003 to 0.021) | 1.07 (0.36 to 2.48) | 11.52 (3.45 to 44.98) | 6.31 (5.7 to 6.92) |
| Equatorial Guinea | Chronic lymphoid leukemia | DALYs | 0 (0 to 0.001) | 0.14 (0.02 to 0.41) | 0.009 (0.003 to 0.02) | 1.88 (0.69 to 3.98) | 29.91 (12.81 to 108.88) | 11.43 (10.19 to 12.67) |
| Equatorial Guinea | Other leukemia | DALYs | 0.002 (0 to 0.006) | 0.84 (0.15 to 2.27) | 0.016 (0.006 to 0.034) | 2.02 (0.81 to 4.12) | 6.76 (2.1 to 29.1) | 3.95 (3.49 to 4.42) |
| Eritrea | Leukemia | death | 0 (0 to 0.001) | 0.03 (0.01 to 0.08) | 0.003 (0.001 to 0.005) | 0.1 (0.04 to 0.2) | 6.7 (3.8 to 15.31) | 3.96 (3.52 to 4.4) |
| Eritrea | Acute myeloid leukemia | death | 0 (0 to 0) | 0 (0 to 0.01) | 0 (0 to 0.001) | 0.01 (0 to 0.02) | 9.19 (4.75 to 21.22) | 4.56 (4.03 to 5.09) |
| Eritrea | Acute lymphoid leukemia | death | 0 (0 to 0) | 0 (0 to 0) | 0 (0 to 0) | 0 (0 to 0.01) | 7.17 (3.81 to 17.51) | 3.48 (3.06 to 3.9) |
| Eritrea | Chronic myeloid leukemia | death | 0 (0 to 0) | 0.01 (0 to 0.02) | 0.001 (0 to 0.001) | 0.02 (0.01 to 0.03) | 4.93 (2.51 to 11.85) | 2.74 (2.35 to 3.13) |
| Eritrea | Chronic lymphoid leukemia | death | 0 (0 to 0) | 0.01 (0 to 0.03) | 0.001 (0 to 0.002) | 0.05 (0.02 to 0.1) | 11.98 (6.06 to 30.73) | 5.73 (5.18 to 6.28) |
| Eritrea | Other leukemia | death | 0 (0 to 0) | 0.01 (0 to 0.03) | 0.001 (0 to 0.002) | 0.03 (0.01 to 0.05) | 4.54 (2.42 to 10.73) | 2.47 (2.04 to 2.91) |
| Eritrea | Leukemia | DALYs | 0.012 (0.003 to 0.031) | 0.92 (0.23 to 2.23) | 0.089 (0.034 to 0.176) | 2.62 (1.02 to 5.15) | 6.15 (3.34 to 14.02) | 3.63 (3.21 to 4.06) |
| Eritrea | Acute myeloid leukemia | DALYs | 0.001 (0 to 0.003) | 0.07 (0.02 to 0.18) | 0.011 (0.004 to 0.023) | 0.26 (0.1 to 0.55) | 9.03 (4.6 to 21.11) | 4.45 (3.91 to 5) |
| Eritrea | Acute lymphoid leukemia | DALYs | 0.001 (0 to 0.002) | 0.04 (0.01 to 0.1) | 0.006 (0.002 to 0.013) | 0.12 (0.04 to 0.26) | 7.49 (3.76 to 19.42) | 3.59 (3.17 to 4) |
| Eritrea | Chronic myeloid leukemia | DALYs | 0.003 (0.001 to 0.009) | 0.22 (0.05 to 0.58) | 0.02 (0.007 to 0.042) | 0.49 (0.17 to 1.01) | 4.83 (2.44 to 11.98) | 2.61 (2.21 to 3) |
| Eritrea | Chronic lymphoid leukemia | DALYs | 0.002 (0 to 0.005) | 0.21 (0.05 to 0.58) | 0.024 (0.008 to 0.05) | 0.97 (0.35 to 2.01) | 11.5 (5.88 to 29.21) | 5.78 (5.26 to 6.31) |
| Eritrea | Other leukemia | DALYs | 0.005 (0.001 to 0.013) | 0.38 (0.1 to 0.95) | 0.029 (0.01 to 0.059) | 0.78 (0.28 to 1.57) | 4.31 (2.22 to 10.35) | 2.29 (1.86 to 2.73) |
| Estonia | Leukemia | death | 0.011 (0.005 to 0.018) | 0.52 (0.24 to 0.89) | 0.017 (0.008 to 0.028) | 0.63 (0.32 to 1.06) | 0.58 (0.21 to 1.14) | 0.81 (0.57 to 1.05) |
| Estonia | Acute myeloid leukemia | death | 0.002 (0.001 to 0.006) | 0.12 (0.05 to 0.28) | 0.006 (0.003 to 0.01) | 0.24 (0.12 to 0.4) | 1.5 (0.28 to 2.9) | 3.12 (2.6 to 3.66) |
| Estonia | Acute lymphoid leukemia | death | 0.001 (0 to 0.001) | 0.04 (0.02 to 0.07) | 0.001 (0.001 to 0.002) | 0.06 (0.03 to 0.1) | 0.58 (0.04 to 1.36) | 1.22 (1.04 to 1.41) |
| Estonia | Chronic myeloid leukemia | death | 0.001 (0.001 to 0.002) | 0.06 (0.03 to 0.1) | 0.002 (0.001 to 0.003) | 0.06 (0.03 to 0.1) | 0.36 (-0.07 to 1.08) | 0.28 (-0.35 to 0.91) |
| Estonia | Chronic lymphoid leukemia | death | 0.001 (0 to 0.001) | 0.03 (0.01 to 0.05) | 0.003 (0.001 to 0.004) | 0.09 (0.04 to 0.15) | 3.25 (1.78 to 5.94) | 5.9 (3.97 to 7.87) |
| Estonia | Other leukemia | death | 0.006 (0.003 to 0.01) | 0.28 (0.13 to 0.49) | 0.005 (0.003 to 0.009) | 0.19 (0.09 to 0.32) | -0.05 (-0.3 to 0.41) | -2.58 (-4.05 to -1.09) |
| Estonia | Leukemia | DALYs | 0.279 (0.134 to 0.467) | 14.26 (6.82 to 23.95) | 0.35 (0.176 to 0.587) | 15.63 (7.96 to 25.93) | 0.25 (-0.04 to 0.69) | 0.34 (0.11 to 0.58) |
| Estonia | Acute myeloid leukemia | DALYs | 0.072 (0.031 to 0.157) | 3.74 (1.61 to 8.13) | 0.134 (0.066 to 0.228) | 6.38 (3.16 to 10.69) | 0.88 (0.04 to 1.88) | 2.34 (1.93 to 2.75) |
| Estonia | Acute lymphoid leukemia | DALYs | 0.026 (0.012 to 0.047) | 1.41 (0.67 to 2.57) | 0.037 (0.019 to 0.063) | 2 (1.01 to 3.37) | 0.4 (-0.06 to 1.11) | 1.43 (1.28 to 1.59) |
| Estonia | Chronic myeloid leukemia | DALYs | 0.03 (0.014 to 0.054) | 1.55 (0.71 to 2.73) | 0.033 (0.017 to 0.057) | 1.43 (0.72 to 2.45) | 0.08 (-0.27 to 0.63) | -0.36 (-0.96 to 0.23) |
| Estonia | Chronic lymphoid leukemia | DALYs | 0.014 (0.006 to 0.026) | 0.71 (0.31 to 1.29) | 0.05 (0.025 to 0.086) | 1.93 (0.95 to 3.29) | 2.52 (1.28 to 4.78) | 5.33 (3.6 to 7.09) |
| Estonia | Other leukemia | DALYs | 0.136 (0.063 to 0.241) | 6.84 (3.18 to 12.14) | 0.095 (0.048 to 0.16) | 3.88 (1.97 to 6.5) | -0.3 (-0.49 to 0.04) | -3.23 (-4.58 to -1.85) |
| Eswatini | Leukemia | death | 0.001 (0 to 0.002) | 0.3 (0.14 to 0.51) | 0.002 (0.001 to 0.004) | 0.44 (0.21 to 0.76) | 1.77 (0.83 to 3.08) | 1.36 (0.79 to 1.93) |
| Eswatini | Acute myeloid leukemia | death | 0 (0 to 0) | 0.02 (0.01 to 0.03) | 0 (0 to 0) | 0.03 (0.01 to 0.05) | 2.32 (0.84 to 4.72) | 1.65 (1.25 to 2.05) |
| Eswatini | Acute lymphoid leukemia | death | 0 (0 to 0) | 0.01 (0 to 0.01) | 0 (0 to 0) | 0.01 (0.01 to 0.02) | 2.41 (1.01 to 4.87) | 1.71 (1.19 to 2.23) |
| Eswatini | Chronic myeloid leukemia | death | 0 (0 to 0) | 0.01 (0 to 0.01) | 0 (0 to 0) | 0.01 (0 to 0.02) | 1.84 (0.67 to 4.02) | 1.45 (0.73 to 2.19) |
| Eswatini | Chronic lymphoid leukemia | death | 0 (0 to 0.001) | 0.15 (0.06 to 0.27) | 0.001 (0.001 to 0.002) | 0.25 (0.11 to 0.45) | 2.15 (1.01 to 3.75) | 1.92 (1.31 to 2.54) |
| Eswatini | Other leukemia | death | 0 (0 to 0.001) | 0.12 (0.05 to 0.21) | 0.001 (0 to 0.002) | 0.14 (0.07 to 0.24) | 1.25 (0.49 to 2.44) | 0.45 (-0.1 to 1) |
| Eswatini | Leukemia | DALYs | 0.029 (0.013 to 0.049) | 7.81 (3.65 to 13.36) | 0.079 (0.036 to 0.143) | 11.07 (5.24 to 19.66) | 1.74 (0.7 to 3.22) | 1.33 (0.74 to 1.92) |
| Eswatini | Acute myeloid leukemia | DALYs | 0.003 (0.001 to 0.006) | 0.66 (0.29 to 1.22) | 0.011 (0.004 to 0.02) | 1.16 (0.5 to 2.16) | 2.4 (0.81 to 5.05) | 1.78 (1.36 to 2.21) |
| Eswatini | Acute lymphoid leukemia | DALYs | 0.001 (0.001 to 0.003) | 0.26 (0.11 to 0.5) | 0.005 (0.002 to 0.009) | 0.47 (0.2 to 0.92) | 2.51 (0.95 to 5.33) | 1.84 (1.36 to 2.31) |
| Eswatini | Chronic myeloid leukemia | DALYs | 0.001 (0 to 0.002) | 0.24 (0.11 to 0.46) | 0.003 (0.001 to 0.006) | 0.36 (0.15 to 0.74) | 1.81 (0.55 to 4.17) | 1.48 (0.72 to 2.25) |
| Eswatini | Chronic lymphoid leukemia | DALYs | 0.009 (0.004 to 0.017) | 3.16 (1.3 to 5.58) | 0.029 (0.012 to 0.054) | 5.04 (2.17 to 9.47) | 2.09 (0.9 to 3.89) | 1.91 (1.26 to 2.57) |
| Eswatini | Other leukemia | DALYs | 0.014 (0.006 to 0.025) | 3.48 (1.58 to 6.25) | 0.031 (0.015 to 0.057) | 4.05 (1.94 to 7.29) | 1.28 (0.42 to 2.69) | 0.55 (-0.03 to 1.13) |
| Ethiopia | Leukemia | death | 0.029 (0.005 to 0.084) | 0.13 (0.02 to 0.37) | 0.09 (0.032 to 0.193) | 0.2 (0.07 to 0.43) | 2.09 (0.72 to 7.74) | 1.43 (1.06 to 1.8) |
| Ethiopia | Acute myeloid leukemia | death | 0.003 (0 to 0.009) | 0.01 (0 to 0.04) | 0.016 (0.006 to 0.033) | 0.03 (0.01 to 0.07) | 4.5 (1.77 to 14.05) | 3.54 (3.16 to 3.92) |
| Ethiopia | Acute lymphoid leukemia | death | 0.002 (0 to 0.006) | 0.01 (0 to 0.02) | 0.009 (0.003 to 0.018) | 0.01 (0.01 to 0.03) | 3.16 (0.8 to 11.86) | 2.26 (1.95 to 2.58) |
| Ethiopia | Chronic myeloid leukemia | death | 0.017 (0.003 to 0.054) | 0.07 (0.01 to 0.21) | 0.039 (0.014 to 0.084) | 0.08 (0.03 to 0.18) | 1.24 (0.12 to 6.43) | 0.2 (-0.16 to 0.55) |
| Ethiopia | Chronic lymphoid leukemia | death | 0.003 (0 to 0.009) | 0.02 (0 to 0.06) | 0.017 (0.005 to 0.037) | 0.05 (0.01 to 0.11) | 4.53 (2.22 to 12.98) | 3.19 (2.74 to 3.64) |
| Ethiopia | Other leukemia | death | 0.004 (0 to 0.012) | 0.02 (0 to 0.05) | 0.01 (0.003 to 0.023) | 0.02 (0.01 to 0.05) | 1.57 (0.35 to 7.24) | 0.88 (0.65 to 1.11) |
| Ethiopia | Leukemia | DALYs | 1.075 (0.195 to 3.125) | 3.9 (0.7 to 11.2) | 3.054 (1.1 to 6.281) | 5.48 (1.95 to 11.63) | 1.84 (0.51 to 7.68) | 0.98 (0.64 to 1.33) |
| Ethiopia | Acute myeloid leukemia | DALYs | 0.103 (0.018 to 0.315) | 0.38 (0.07 to 1.13) | 0.562 (0.203 to 1.155) | 0.97 (0.35 to 2.03) | 4.48 (1.6 to 14.56) | 3.46 (3.11 to 3.81) |
| Ethiopia | Acute lymphoid leukemia | DALYs | 0.09 (0.015 to 0.279) | 0.28 (0.05 to 0.84) | 0.383 (0.14 to 0.778) | 0.53 (0.2 to 1.07) | 3.26 (0.74 to 12.98) | 2.24 (1.96 to 2.52) |
| Ethiopia | Chronic myeloid leukemia | DALYs | 0.67 (0.104 to 2.076) | 2.34 (0.38 to 7.17) | 1.409 (0.501 to 2.95) | 2.4 (0.84 to 5.15) | 1.1 (0.01 to 6.67) | -0.25 (-0.57 to 0.08) |
| Ethiopia | Chronic lymphoid leukemia | DALYs | 0.078 (0.013 to 0.23) | 0.41 (0.06 to 1.2) | 0.389 (0.128 to 0.824) | 0.98 (0.31 to 2.1) | 3.98 (1.89 to 12.15) | 3.14 (2.72 to 3.55) |
| Ethiopia | Other leukemia | DALYs | 0.135 (0.016 to 0.42) | 0.5 (0.06 to 1.53) | 0.312 (0.097 to 0.719) | 0.59 (0.18 to 1.38) | 1.3 (0.17 to 6.74) | 0.38 (0.16 to 0.6) |
| Fiji | Leukemia | death | 0.002 (0.001 to 0.004) | 0.5 (0.22 to 0.87) | 0.004 (0.002 to 0.007) | 0.54 (0.27 to 0.93) | 0.88 (0.33 to 1.84) | -0.08 (-0.32 to 0.17) |
| Fiji | Acute myeloid leukemia | death | 0.001 (0 to 0.002) | 0.2 (0.09 to 0.37) | 0.002 (0.001 to 0.004) | 0.28 (0.14 to 0.47) | 1.27 (0.59 to 2.54) | 1.05 (0.87 to 1.22) |
| Fiji | Acute lymphoid leukemia | death | 0 (0 to 0) | 0.04 (0.01 to 0.07) | 0 (0 to 0) | 0.03 (0.02 to 0.06) | 0.46 (-0.06 to 1.46) | -0.91 (-1.37 to -0.45) |
| Fiji | Chronic myeloid leukemia | death | 0 (0 to 0.001) | 0.09 (0.04 to 0.17) | 0.001 (0 to 0.001) | 0.07 (0.03 to 0.13) | 0.39 (-0.1 to 1.24) | -1.57 (-1.96 to -1.18) |
| Fiji | Chronic lymphoid leukemia | death | 0 (0 to 0) | 0.01 (0 to 0.01) | 0 (0 to 0) | 0.01 (0 to 0.02) | 1.64 (0.65 to 3.45) | 0.53 (0.2 to 0.87) |
| Fiji | Other leukemia | death | 0.001 (0 to 0.001) | 0.16 (0.06 to 0.3) | 0.001 (0 to 0.002) | 0.15 (0.07 to 0.27) | 0.6 (0.03 to 1.58) | -0.85 (-1.21 to -0.49) |
| Fiji | Leukemia | DALYs | 0.084 (0.037 to 0.144) | 15.3 (6.84 to 26.46) | 0.139 (0.07 to 0.235) | 16.15 (8.1 to 27.33) | 0.66 (0.16 to 1.55) | -0.16 (-0.37 to 0.04) |
| Fiji | Acute myeloid leukemia | DALYs | 0.041 (0.018 to 0.075) | 7.04 (3.21 to 12.68) | 0.083 (0.042 to 0.141) | 9.35 (4.77 to 15.74) | 1.01 (0.35 to 2.11) | 0.91 (0.75 to 1.07) |
| Fiji | Acute lymphoid leukemia | DALYs | 0.007 (0.003 to 0.014) | 1.18 (0.5 to 2.23) | 0.01 (0.005 to 0.018) | 1.09 (0.53 to 1.99) | 0.33 (-0.18 to 1.3) | -0.93 (-1.32 to -0.55) |
| Fiji | Chronic myeloid leukemia | DALYs | 0.014 (0.006 to 0.025) | 2.71 (1.2 to 5.07) | 0.017 (0.007 to 0.031) | 1.93 (0.84 to 3.69) | 0.21 (-0.23 to 0.98) | -1.87 (-2.25 to -1.49) |
| Fiji | Chronic lymphoid leukemia | DALYs | 0.001 (0 to 0.002) | 0.22 (0.09 to 0.39) | 0.002 (0.001 to 0.004) | 0.27 (0.13 to 0.49) | 1.38 (0.45 to 3.08) | 0.42 (0.14 to 0.71) |
| Fiji | Other leukemia | DALYs | 0.02 (0.009 to 0.036) | 4.16 (1.77 to 7.47) | 0.028 (0.013 to 0.05) | 3.5 (1.68 to 6.29) | 0.35 (-0.14 to 1.29) | -1.17 (-1.48 to -0.86) |
| Finland | Leukemia | death | 0.024 (0.01 to 0.041) | 0.33 (0.14 to 0.58) | 0.035 (0.016 to 0.06) | 0.28 (0.13 to 0.49) | 0.48 (0.27 to 0.78) | -0.59 (-0.63 to -0.55) |
| Finland | Acute myeloid leukemia | death | 0.01 (0.004 to 0.017) | 0.14 (0.06 to 0.25) | 0.017 (0.008 to 0.03) | 0.14 (0.07 to 0.25) | 0.71 (0.44 to 1.1) | 0.11 (0.05 to 0.18) |
| Finland | Acute lymphoid leukemia | death | 0.001 (0.001 to 0.003) | 0.02 (0.01 to 0.04) | 0.002 (0.001 to 0.003) | 0.02 (0.01 to 0.04) | 0.39 (0.1 to 0.8) | -0.35 (-0.51 to -0.2) |
| Finland | Chronic myeloid leukemia | death | 0.004 (0.002 to 0.007) | 0.06 (0.02 to 0.1) | 0.002 (0.001 to 0.003) | 0.01 (0.01 to 0.02) | -0.57 (-0.66 to -0.44) | -5.3 (-5.56 to -5.05) |
| Finland | Chronic lymphoid leukemia | death | 0.005 (0.002 to 0.008) | 0.06 (0.03 to 0.11) | 0.007 (0.003 to 0.013) | 0.05 (0.03 to 0.1) | 0.65 (0.31 to 1.14) | -0.76 (-1.21 to -0.31) |
| Finland | Other leukemia | death | 0.004 (0.002 to 0.007) | 0.05 (0.02 to 0.09) | 0.007 (0.003 to 0.012) | 0.05 (0.02 to 0.09) | 0.81 (0.37 to 1.38) | 0.3 (-0.12 to 0.72) |
| Finland | Leukemia | DALYs | 0.565 (0.247 to 0.982) | 8.45 (3.7 to 14.68) | 0.689 (0.323 to 1.164) | 6.8 (3.22 to 11.35) | 0.22 (0.06 to 0.45) | -0.78 (-0.83 to -0.73) |
| Finland | Acute myeloid leukemia | DALYs | 0.245 (0.104 to 0.426) | 3.66 (1.56 to 6.42) | 0.344 (0.159 to 0.599) | 3.5 (1.64 to 5.95) | 0.41 (0.19 to 0.7) | -0.13 (-0.2 to -0.07) |
| Finland | Acute lymphoid leukemia | DALYs | 0.047 (0.021 to 0.084) | 0.77 (0.34 to 1.38) | 0.066 (0.032 to 0.112) | 0.87 (0.43 to 1.49) | 0.4 (0.07 to 0.84) | 0.26 (0.02 to 0.5) |
| Finland | Chronic myeloid leukemia | DALYs | 0.1 (0.044 to 0.177) | 1.52 (0.68 to 2.68) | 0.037 (0.017 to 0.062) | 0.39 (0.19 to 0.66) | -0.63 (-0.71 to -0.53) | -5.3 (-5.53 to -5.06) |
| Finland | Chronic lymphoid leukemia | DALYs | 0.091 (0.041 to 0.162) | 1.3 (0.58 to 2.3) | 0.132 (0.063 to 0.234) | 1.08 (0.51 to 1.88) | 0.45 (0.13 to 0.91) | -0.89 (-1.3 to -0.47) |
| Finland | Other leukemia | DALYs | 0.082 (0.035 to 0.146) | 1.2 (0.51 to 2.13) | 0.11 (0.051 to 0.194) | 0.96 (0.46 to 1.69) | 0.34 (0.05 to 0.71) | -0.32 (-0.68 to 0.04) |
| France | Leukemia | death | 0.304 (0.136 to 0.538) | 0.37 (0.17 to 0.65) | 0.604 (0.282 to 1.039) | 0.41 (0.19 to 0.69) | 0.98 (0.67 to 1.49) | 0.29 (0.18 to 0.4) |
| France | Acute myeloid leukemia | death | 0.07 (0.031 to 0.125) | 0.09 (0.04 to 0.16) | 0.207 (0.093 to 0.36) | 0.15 (0.07 to 0.26) | 1.94 (1.03 to 2.87) | 2.23 (2.06 to 2.4) |
| France | Acute lymphoid leukemia | death | 0.016 (0.007 to 0.03) | 0.02 (0.01 to 0.04) | 0.025 (0.012 to 0.042) | 0.02 (0.01 to 0.04) | 0.54 (0.26 to 1.09) | -0.08 (-0.2 to 0.03) |
| France | Chronic myeloid leukemia | death | 0.04 (0.018 to 0.071) | 0.05 (0.02 to 0.09) | 0.04 (0.018 to 0.069) | 0.03 (0.01 to 0.04) | -0.01 (-0.21 to 0.27) | -3.45 (-3.93 to -2.96) |
| France | Chronic lymphoid leukemia | death | 0.061 (0.027 to 0.108) | 0.07 (0.03 to 0.13) | 0.122 (0.056 to 0.218) | 0.07 (0.03 to 0.13) | 1.02 (0.61 to 1.63) | -0.18 (-0.41 to 0.04) |
| France | Other leukemia | death | 0.117 (0.052 to 0.206) | 0.14 (0.06 to 0.25) | 0.21 (0.094 to 0.364) | 0.13 (0.06 to 0.23) | 0.79 (0.47 to 1.3) | -0.04 (-0.13 to 0.04) |
| France | Leukemia | DALYs | 6.673 (2.986 to 11.805) | 8.83 (3.96 to 15.6) | 10.71 (5.172 to 18.272) | 9.02 (4.41 to 15.2) | 0.61 (0.38 to 0.98) | -0.01 (-0.12 to 0.09) |
| France | Acute myeloid leukemia | DALYs | 1.701 (0.742 to 3.009) | 2.32 (1.01 to 4.11) | 3.994 (1.877 to 6.759) | 3.57 (1.7 to 6.02) | 1.35 (0.75 to 2.03) | 1.76 (1.6 to 1.92) |
| France | Acute lymphoid leukemia | DALYs | 0.507 (0.218 to 0.935) | 0.74 (0.31 to 1.36) | 0.81 (0.393 to 1.38) | 0.92 (0.44 to 1.57) | 0.6 (0.27 to 1.25) | 0.64 (0.57 to 0.72) |
| France | Chronic myeloid leukemia | DALYs | 0.94 (0.418 to 1.661) | 1.28 (0.57 to 2.27) | 0.725 (0.342 to 1.248) | 0.58 (0.28 to 1) | -0.23 (-0.37 to -0.01) | -3.86 (-4.36 to -3.37) |
| France | Chronic lymphoid leukemia | DALYs | 1.113 (0.501 to 1.951) | 1.35 (0.61 to 2.37) | 1.859 (0.896 to 3.338) | 1.29 (0.63 to 2.28) | 0.67 (0.37 to 1.14) | -0.49 (-0.77 to -0.21) |
| France | Other leukemia | DALYs | 2.411 (1.085 to 4.244) | 3.14 (1.41 to 5.53) | 3.322 (1.584 to 5.666) | 2.65 (1.29 to 4.49) | 0.38 (0.16 to 0.74) | -0.55 (-0.62 to -0.48) |
| Gabon | Leukemia | death | 0.001 (0 to 0.002) | 0.14 (0.05 to 0.28) | 0.003 (0.001 to 0.005) | 0.26 (0.12 to 0.45) | 2.5 (1.23 to 5.53) | 1.96 (1.63 to 2.29) |
| Gabon | Acute myeloid leukemia | death | 0 (0 to 0) | 0.02 (0.01 to 0.05) | 0.001 (0 to 0.001) | 0.04 (0.02 to 0.08) | 3.53 (1.34 to 9.32) | 2.52 (2.27 to 2.77) |
| Gabon | Acute lymphoid leukemia | death | 0 (0 to 0) | 0 (0 to 0.01) | 0 (0 to 0) | 0.01 (0 to 0.02) | 3.58 (1.43 to 8.84) | 2.41 (2.2 to 2.63) |
| Gabon | Chronic myeloid leukemia | death | 0 (0 to 0) | 0.03 (0.01 to 0.07) | 0.001 (0 to 0.001) | 0.04 (0.02 to 0.08) | 1.89 (0.61 to 5.09) | 1.05 (0.88 to 1.22) |
| Gabon | Chronic lymphoid leukemia | death | 0 (0 to 0) | 0.02 (0.01 to 0.06) | 0.001 (0 to 0.002) | 0.09 (0.04 to 0.17) | 5.34 (2.84 to 12.03) | 4.67 (3.97 to 5.36) |
| Gabon | Other leukemia | death | 0 (0 to 0.001) | 0.06 (0.02 to 0.13) | 0.001 (0 to 0.002) | 0.08 (0.03 to 0.15) | 1.38 (0.48 to 3.61) | 0.29 (-0.04 to 0.62) |
| Gabon | Leukemia | DALYs | 0.027 (0.009 to 0.055) | 4.19 (1.45 to 8.45) | 0.096 (0.044 to 0.169) | 7.29 (3.33 to 12.78) | 2.55 (1.2 to 5.72) | 1.74 (1.4 to 2.08) |
| Gabon | Acute myeloid leukemia | DALYs | 0.004 (0.001 to 0.011) | 0.61 (0.18 to 1.7) | 0.02 (0.009 to 0.039) | 1.38 (0.61 to 2.65) | 3.86 (1.56 to 10.09) | 2.59 (2.32 to 2.86) |
| Gabon | Acute lymphoid leukemia | DALYs | 0.001 (0 to 0.003) | 0.16 (0.05 to 0.38) | 0.006 (0.002 to 0.011) | 0.37 (0.16 to 0.7) | 4.02 (1.57 to 9.96) | 2.67 (2.45 to 2.89) |
| Gabon | Chronic myeloid leukemia | DALYs | 0.006 (0.002 to 0.016) | 0.94 (0.29 to 2.34) | 0.018 (0.007 to 0.037) | 1.32 (0.53 to 2.58) | 2.01 (0.67 to 5.56) | 1.02 (0.82 to 1.23) |
| Gabon | Chronic lymphoid leukemia | DALYs | 0.003 (0.001 to 0.008) | 0.58 (0.17 to 1.37) | 0.021 (0.008 to 0.04) | 1.93 (0.77 to 3.65) | 5.2 (2.75 to 11.53) | 4.36 (3.64 to 5.08) |
| Gabon | Other leukemia | DALYs | 0.012 (0.004 to 0.025) | 1.9 (0.66 to 3.87) | 0.031 (0.013 to 0.059) | 2.29 (0.95 to 4.39) | 1.51 (0.51 to 3.96) | 0.29 (-0.06 to 0.64) |
| Gambia | Leukemia | death | 0 (0 to 0.001) | 0.07 (0.02 to 0.14) | 0.002 (0.001 to 0.003) | 0.15 (0.07 to 0.27) | 4.71 (2.68 to 9.78) | 2.59 (2.38 to 2.8) |
| Gambia | Acute myeloid leukemia | death | 0 (0 to 0) | 0.01 (0 to 0.01) | 0 (0 to 0) | 0.02 (0.01 to 0.03) | 5.64 (3.05 to 12.68) | 3.22 (3.02 to 3.42) |
| Gambia | Acute lymphoid leukemia | death | 0 (0 to 0) | 0 (0 to 0.01) | 0 (0 to 0) | 0.01 (0 to 0.01) | 5.16 (2.57 to 12.16) | 2.99 (2.77 to 3.2) |
| Gambia | Chronic myeloid leukemia | death | 0 (0 to 0) | 0.01 (0 to 0.02) | 0 (0 to 0.001) | 0.02 (0.01 to 0.05) | 4.24 (2.13 to 9.65) | 2.37 (2.08 to 2.66) |
| Gambia | Chronic lymphoid leukemia | death | 0 (0 to 0) | 0.02 (0.01 to 0.04) | 0 (0 to 0.001) | 0.04 (0.02 to 0.08) | 5.44 (3.1 to 11.08) | 2.96 (2.76 to 3.16) |
| Gambia | Other leukemia | death | 0 (0 to 0) | 0.03 (0.01 to 0.07) | 0.001 (0 to 0.001) | 0.06 (0.03 to 0.12) | 4.28 (2.33 to 9.47) | 2.28 (2.09 to 2.48) |
| Gambia | Leukemia | DALYs | 0.01 (0.003 to 0.02) | 2.08 (0.71 to 4.29) | 0.055 (0.025 to 0.1) | 4.48 (1.99 to 8.01) | 4.61 (2.57 to 9.91) | 2.62 (2.38 to 2.85) |
| Gambia | Acute myeloid leukemia | DALYs | 0.001 (0 to 0.003) | 0.21 (0.07 to 0.46) | 0.008 (0.003 to 0.015) | 0.54 (0.23 to 1.02) | 5.63 (2.99 to 12.57) | 3.23 (3.03 to 3.43) |
| Gambia | Acute lymphoid leukemia | DALYs | 0.001 (0 to 0.002) | 0.12 (0.04 to 0.27) | 0.005 (0.002 to 0.009) | 0.29 (0.12 to 0.56) | 5.11 (2.42 to 12.42) | 2.99 (2.76 to 3.22) |
| Gambia | Chronic myeloid leukemia | DALYs | 0.002 (0.001 to 0.005) | 0.43 (0.14 to 0.9) | 0.012 (0.005 to 0.023) | 0.85 (0.37 to 1.62) | 4.13 (2.03 to 9.6) | 2.35 (2.05 to 2.66) |
| Gambia | Chronic lymphoid leukemia | DALYs | 0.002 (0.001 to 0.003) | 0.42 (0.14 to 0.91) | 0.01 (0.004 to 0.018) | 1.01 (0.42 to 1.9) | 5.46 (3.07 to 11.32) | 3.01 (2.79 to 3.23) |
| Gambia | Other leukemia | DALYs | 0.004 (0.001 to 0.008) | 0.9 (0.31 to 1.87) | 0.021 (0.009 to 0.038) | 1.78 (0.8 to 3.27) | 4.16 (2.22 to 9.63) | 2.33 (2.11 to 2.55) |
| Georgia | Leukemia | death | 0.02 (0.01 to 0.034) | 0.33 (0.16 to 0.56) | 0.021 (0.01 to 0.035) | 0.39 (0.19 to 0.64) | 0.03 (-0.18 to 0.26) | 1.3 (0.82 to 1.78) |
| Georgia | Acute myeloid leukemia | death | 0.004 (0.002 to 0.008) | 0.06 (0.03 to 0.13) | 0.005 (0.002 to 0.008) | 0.09 (0.04 to 0.16) | 0.28 (-0.42 to 0.94) | 2.13 (0.86 to 3.43) |
| Georgia | Acute lymphoid leukemia | death | 0.002 (0.001 to 0.004) | 0.03 (0.02 to 0.06) | 0.002 (0.001 to 0.003) | 0.04 (0.02 to 0.06) | -0.15 (-0.44 to 0.16) | 0.5 (-0.34 to 1.35) |
| Georgia | Chronic myeloid leukemia | death | 0.001 (0.001 to 0.002) | 0.02 (0.01 to 0.04) | 0.001 (0.001 to 0.002) | 0.03 (0.01 to 0.04) | 0.08 (-0.25 to 0.76) | 1.51 (0.39 to 2.64) |
| Georgia | Chronic lymphoid leukemia | death | 0.001 (0 to 0.001) | 0.01 (0.01 to 0.02) | 0.002 (0.001 to 0.003) | 0.03 (0.01 to 0.05) | 1.27 (0.55 to 2.41) | 4.29 (2.85 to 5.74) |
| Georgia | Other leukemia | death | 0.012 (0.006 to 0.021) | 0.21 (0.1 to 0.35) | 0.011 (0.005 to 0.019) | 0.21 (0.1 to 0.35) | -0.1 (-0.32 to 0.33) | 0.81 (0.15 to 1.47) |
| Georgia | Leukemia | DALYs | 0.715 (0.344 to 1.194) | 11.77 (5.66 to 19.69) | 0.617 (0.305 to 1.022) | 12.74 (6.31 to 21.01) | -0.14 (-0.31 to 0.07) | 0.92 (0.42 to 1.43) |
| Georgia | Acute myeloid leukemia | DALYs | 0.139 (0.059 to 0.295) | 2.3 (0.98 to 4.82) | 0.152 (0.07 to 0.256) | 3.19 (1.5 to 5.39) | 0.09 (-0.48 to 0.65) | 1.71 (0.46 to 2.97) |
| Georgia | Acute lymphoid leukemia | DALYs | 0.082 (0.037 to 0.147) | 1.37 (0.63 to 2.48) | 0.061 (0.029 to 0.101) | 1.37 (0.67 to 2.3) | -0.26 (-0.5 to 0.03) | 0.21 (-0.64 to 1.07) |
| Georgia | Chronic myeloid leukemia | DALYs | 0.044 (0.02 to 0.078) | 0.72 (0.33 to 1.27) | 0.042 (0.02 to 0.072) | 0.89 (0.41 to 1.54) | -0.04 (-0.34 to 0.62) | 1.32 (0.17 to 2.49) |
| Georgia | Chronic lymphoid leukemia | DALYs | 0.021 (0.01 to 0.037) | 0.34 (0.16 to 0.59) | 0.041 (0.019 to 0.07) | 0.71 (0.33 to 1.22) | 0.93 (0.28 to 2.07) | 3.82 (2.37 to 5.28) |
| Georgia | Other leukemia | DALYs | 0.43 (0.207 to 0.727) | 7.05 (3.43 to 11.98) | 0.323 (0.16 to 0.551) | 6.59 (3.3 to 11.29) | -0.25 (-0.43 to 0.14) | 0.46 (-0.27 to 1.2) |
| Germany | Leukemia | death | 0.48 (0.214 to 0.828) | 0.39 (0.17 to 0.67) | 0.851 (0.409 to 1.433) | 0.43 (0.21 to 0.72) | 0.77 (0.48 to 1.08) | 0.46 (0.29 to 0.63) |
| Germany | Acute myeloid leukemia | death | 0.136 (0.059 to 0.236) | 0.11 (0.05 to 0.2) | 0.359 (0.164 to 0.613) | 0.2 (0.09 to 0.33) | 1.64 (1.03 to 2.25) | 2.35 (2.21 to 2.48) |
| Germany | Acute lymphoid leukemia | death | 0.026 (0.011 to 0.047) | 0.02 (0.01 to 0.04) | 0.037 (0.018 to 0.063) | 0.02 (0.01 to 0.04) | 0.41 (0.14 to 0.98) | -0.12 (-0.3 to 0.07) |
| Germany | Chronic myeloid leukemia | death | 0.097 (0.044 to 0.17) | 0.08 (0.04 to 0.14) | 0.062 (0.028 to 0.109) | 0.03 (0.01 to 0.05) | -0.36 (-0.5 to -0.19) | -3.96 (-4.41 to -3.51) |
| Germany | Chronic lymphoid leukemia | death | 0.113 (0.05 to 0.196) | 0.09 (0.04 to 0.15) | 0.216 (0.1 to 0.373) | 0.1 (0.05 to 0.17) | 0.92 (0.56 to 1.45) | 0.53 (0.41 to 0.64) |
| Germany | Other leukemia | death | 0.108 (0.048 to 0.191) | 0.08 (0.04 to 0.15) | 0.178 (0.08 to 0.316) | 0.08 (0.04 to 0.15) | 0.64 (0.27 to 1.1) | -0.01 (-0.46 to 0.44) |
| Germany | Leukemia | DALYs | 11.363 (5.123 to 19.567) | 9.87 (4.44 to 17.05) | 16.911 (8.342 to 28.356) | 10.38 (5.14 to 17.34) | 0.49 (0.29 to 0.74) | 0.14 (0.07 to 0.21) |
| Germany | Acute myeloid leukemia | DALYs | 3.58 (1.578 to 6.284) | 3.21 (1.42 to 5.66) | 7.591 (3.597 to 12.747) | 4.9 (2.37 to 8.21) | 1.12 (0.72 to 1.56) | 1.8 (1.7 to 1.91) |
| Germany | Acute lymphoid leukemia | DALYs | 0.919 (0.384 to 1.658) | 0.9 (0.38 to 1.65) | 1.309 (0.663 to 2.21) | 1.11 (0.57 to 1.86) | 0.42 (0.13 to 1.03) | 0.41 (0.2 to 0.62) |
| Germany | Chronic myeloid leukemia | DALYs | 2.486 (1.123 to 4.388) | 2.2 (0.99 to 3.89) | 1.499 (0.706 to 2.637) | 0.89 (0.42 to 1.55) | -0.4 (-0.52 to -0.22) | -3.95 (-4.3 to -3.61) |
| Germany | Chronic lymphoid leukemia | DALYs | 2.238 (1.001 to 3.878) | 1.79 (0.8 to 3.09) | 3.695 (1.726 to 6.421) | 1.92 (0.89 to 3.35) | 0.65 (0.35 to 1.13) | 0.25 (0.02 to 0.48) |
| Germany | Other leukemia | DALYs | 2.14 (0.95 to 3.745) | 1.78 (0.79 to 3.1) | 2.817 (1.304 to 4.931) | 1.55 (0.73 to 2.7) | 0.32 (0.07 to 0.66) | -0.48 (-0.83 to -0.12) |
| Ghana | Leukemia | death | 0.007 (0.002 to 0.015) | 0.09 (0.03 to 0.19) | 0.038 (0.018 to 0.066) | 0.21 (0.1 to 0.37) | 4.54 (2.57 to 10.65) | 2.68 (2.41 to 2.94) |
| Ghana | Acute myeloid leukemia | death | 0.001 (0 to 0.003) | 0.02 (0.01 to 0.04) | 0.011 (0.005 to 0.019) | 0.06 (0.03 to 0.1) | 6.26 (3.42 to 15.36) | 3.6 (3.34 to 3.85) |
| Ghana | Acute lymphoid leukemia | death | 0.001 (0 to 0.002) | 0.01 (0 to 0.02) | 0.004 (0.002 to 0.007) | 0.02 (0.01 to 0.03) | 3.49 (1.45 to 10.15) | 1.51 (1.08 to 1.94) |
| Ghana | Chronic myeloid leukemia | death | 0.001 (0 to 0.003) | 0.02 (0.01 to 0.04) | 0.007 (0.003 to 0.012) | 0.03 (0.02 to 0.06) | 3.61 (1.64 to 9.09) | 1.96 (1.64 to 2.28) |
| Ghana | Chronic lymphoid leukemia | death | 0.001 (0 to 0.002) | 0.01 (0 to 0.03) | 0.006 (0.003 to 0.01) | 0.04 (0.02 to 0.07) | 6.56 (3.49 to 17.51) | 3.73 (3.5 to 3.96) |
| Ghana | Other leukemia | death | 0.002 (0.001 to 0.005) | 0.04 (0.01 to 0.08) | 0.012 (0.006 to 0.021) | 0.07 (0.04 to 0.13) | 3.77 (1.86 to 9.7) | 2.23 (1.97 to 2.49) |
| Ghana | Leukemia | DALYs | 0.262 (0.083 to 0.562) | 2.93 (0.95 to 6.19) | 1.32 (0.616 to 2.313) | 6.19 (2.89 to 10.69) | 4.04 (2.11 to 9.73) | 2.14 (1.82 to 2.45) |
| Ghana | Acute myeloid leukemia | DALYs | 0.059 (0.018 to 0.127) | 0.62 (0.2 to 1.32) | 0.4 (0.186 to 0.707) | 1.76 (0.83 to 3.08) | 5.8 (3.12 to 14.29) | 3.22 (2.94 to 3.5) |
| Ghana | Acute lymphoid leukemia | DALYs | 0.038 (0.01 to 0.087) | 0.34 (0.1 to 0.75) | 0.157 (0.064 to 0.313) | 0.59 (0.25 to 1.17) | 3.18 (1.18 to 9.91) | 1.07 (0.57 to 1.57) |
| Ghana | Chronic myeloid leukemia | DALYs | 0.061 (0.019 to 0.132) | 0.63 (0.2 to 1.37) | 0.261 (0.118 to 0.485) | 1.1 (0.5 to 2.01) | 3.26 (1.37 to 8.86) | 1.42 (1.04 to 1.8) |
| Ghana | Chronic lymphoid leukemia | DALYs | 0.019 (0.005 to 0.043) | 0.3 (0.08 to 0.68) | 0.135 (0.058 to 0.248) | 0.85 (0.37 to 1.57) | 6.07 (3.14 to 16.27) | 3.45 (3.2 to 3.7) |
| Ghana | Other leukemia | DALYs | 0.085 (0.027 to 0.181) | 1.04 (0.33 to 2.26) | 0.366 (0.18 to 0.663) | 1.89 (0.91 to 3.39) | 3.31 (1.49 to 8.82) | 1.64 (1.34 to 1.95) |
| Greece | Leukemia | death | 0.069 (0.03 to 0.121) | 0.46 (0.2 to 0.81) | 0.138 (0.061 to 0.239) | 0.54 (0.25 to 0.93) | 0.99 (0.73 to 1.33) | 0.17 (-0.21 to 0.55) |
| Greece | Acute myeloid leukemia | death | 0.016 (0.007 to 0.028) | 0.11 (0.05 to 0.19) | 0.047 (0.02 to 0.084) | 0.2 (0.09 to 0.36) | 1.93 (1.03 to 2.6) | 2.3 (2.13 to 2.46) |
| Greece | Acute lymphoid leukemia | death | 0.003 (0.001 to 0.006) | 0.02 (0.01 to 0.04) | 0.005 (0.002 to 0.009) | 0.03 (0.01 to 0.05) | 0.6 (0.36 to 0.92) | 0.56 (0.42 to 0.71) |
| Greece | Chronic myeloid leukemia | death | 0.007 (0.003 to 0.013) | 0.05 (0.02 to 0.09) | 0.01 (0.004 to 0.017) | 0.04 (0.02 to 0.07) | 0.35 (0.1 to 0.68) | -1.89 (-2.24 to -1.54) |
| Greece | Chronic lymphoid leukemia | death | 0.009 (0.004 to 0.016) | 0.06 (0.03 to 0.1) | 0.022 (0.01 to 0.04) | 0.08 (0.04 to 0.14) | 1.57 (1.12 to 2.24) | 0.44 (0.04 to 0.83) |
| Greece | Other leukemia | death | 0.034 (0.015 to 0.06) | 0.23 (0.1 to 0.4) | 0.053 (0.023 to 0.094) | 0.19 (0.08 to 0.33) | 0.58 (0.35 to 0.92) | -1.05 (-1.72 to -0.38) |
| Greece | Leukemia | DALYs | 1.574 (0.677 to 2.751) | 10.94 (4.74 to 19.17) | 2.483 (1.145 to 4.233) | 12.17 (5.69 to 20.62) | 0.58 (0.39 to 0.82) | 0.09 (-0.17 to 0.35) |
| Greece | Acute myeloid leukemia | DALYs | 0.399 (0.17 to 0.707) | 2.83 (1.21 to 5.03) | 0.938 (0.423 to 1.653) | 4.95 (2.27 to 8.66) | 1.35 (0.72 to 1.84) | 2.01 (1.86 to 2.15) |
| Greece | Acute lymphoid leukemia | DALYs | 0.114 (0.05 to 0.203) | 0.89 (0.39 to 1.59) | 0.171 (0.081 to 0.296) | 1.14 (0.54 to 1.95) | 0.5 (0.24 to 0.85) | 0.99 (0.79 to 1.18) |
| Greece | Chronic myeloid leukemia | DALYs | 0.165 (0.071 to 0.292) | 1.14 (0.49 to 2.03) | 0.183 (0.083 to 0.319) | 0.87 (0.4 to 1.49) | 0.11 (-0.08 to 0.39) | -1.76 (-2.06 to -1.46) |
| Greece | Chronic lymphoid leukemia | DALYs | 0.177 (0.078 to 0.31) | 1.15 (0.51 to 2.03) | 0.369 (0.165 to 0.654) | 1.55 (0.71 to 2.75) | 1.09 (0.72 to 1.66) | 0.44 (0.09 to 0.79) |
| Greece | Other leukemia | DALYs | 0.721 (0.312 to 1.272) | 4.93 (2.15 to 8.72) | 0.821 (0.368 to 1.419) | 3.67 (1.66 to 6.26) | 0.14 (-0.01 to 0.38) | -1.47 (-1.96 to -0.99) |
| Greenland | Leukemia | death | 0 (0 to 0) | 0.33 (0.15 to 0.57) | 0 (0 to 0) | 0.29 (0.14 to 0.49) | 0.71 (0.29 to 1.27) | -0.78 (-0.93 to -0.63) |
| Greenland | Acute myeloid leukemia | death | 0 (0 to 0) | 0.06 (0.03 to 0.1) | 0 (0 to 0) | 0.07 (0.03 to 0.13) | 1.16 (0.46 to 2.17) | 0.99 (0.83 to 1.15) |
| Greenland | Acute lymphoid leukemia | death | 0 (0 to 0) | 0.02 (0.01 to 0.03) | 0 (0 to 0) | 0.02 (0.01 to 0.04) | 0.33 (-0.1 to 1.22) | -0.11 (-0.33 to 0.11) |
| Greenland | Chronic myeloid leukemia | death | 0 (0 to 0) | 0.03 (0.01 to 0.06) | 0 (0 to 0) | 0.02 (0.01 to 0.04) | 0.1 (-0.27 to 0.66) | -1.77 (-1.99 to -1.54) |
| Greenland | Chronic lymphoid leukemia | death | 0 (0 to 0) | 0.19 (0.08 to 0.35) | 0 (0 to 0) | 0.15 (0.07 to 0.27) | 0.69 (0.26 to 1.31) | -1.52 (-1.82 to -1.22) |
| Greenland | Other leukemia | death | 0 (0 to 0) | 0.03 (0.01 to 0.05) | 0 (0 to 0) | 0.03 (0.01 to 0.05) | 1 (0.36 to 1.94) | 0.45 (0.31 to 0.59) |
| Greenland | Leukemia | DALYs | 0.003 (0.002 to 0.006) | 8.17 (3.73 to 13.9) | 0.005 (0.002 to 0.008) | 7.22 (3.55 to 12.17) | 0.46 (0.09 to 1.03) | -0.69 (-0.8 to -0.58) |
| Greenland | Acute myeloid leukemia | DALYs | 0.001 (0 to 0.001) | 1.65 (0.75 to 2.87) | 0.001 (0.001 to 0.002) | 1.96 (0.92 to 3.37) | 0.75 (0.19 to 1.66) | 0.83 (0.68 to 0.98) |
| Greenland | Acute lymphoid leukemia | DALYs | 0 (0 to 0.001) | 0.7 (0.31 to 1.26) | 0 (0 to 0.001) | 0.69 (0.34 to 1.3) | 0.09 (-0.3 to 0.87) | -0.3 (-0.56 to -0.04) |
| Greenland | Chronic myeloid leukemia | DALYs | 0 (0 to 0.001) | 0.94 (0.41 to 1.69) | 0 (0 to 0.001) | 0.56 (0.25 to 1.01) | -0.14 (-0.44 to 0.36) | -2.13 (-2.33 to -1.94) |
| Greenland | Chronic lymphoid leukemia | DALYs | 0.002 (0.001 to 0.003) | 4.2 (1.75 to 7.49) | 0.002 (0.001 to 0.004) | 3.33 (1.51 to 5.97) | 0.57 (0.16 to 1.2) | -1.36 (-1.6 to -1.12) |
| Greenland | Other leukemia | DALYs | 0 (0 to 0.001) | 0.68 (0.29 to 1.2) | 0 (0 to 0.001) | 0.67 (0.29 to 1.22) | 0.63 (0.06 to 1.48) | 0.27 (0.15 to 0.4) |
| Grenada | Leukemia | death | 0 (0 to 0) | 0.25 (0.1 to 0.48) | 0 (0 to 0.001) | 0.35 (0.16 to 0.58) | 1.22 (0.72 to 2.37) | 1.19 (0.97 to 1.41) |
| Grenada | Acute myeloid leukemia | death | 0 (0 to 0) | 0.03 (0.01 to 0.05) | 0 (0 to 0) | 0.06 (0.03 to 0.11) | 3.13 (1.88 to 5.65) | 3.29 (3.03 to 3.55) |
| Grenada | Acute lymphoid leukemia | death | 0 (0 to 0) | 0.01 (0 to 0.02) | 0 (0 to 0) | 0.02 (0.01 to 0.03) | 1.36 (0.58 to 3.17) | 1.07 (0.79 to 1.35) |
| Grenada | Chronic myeloid leukemia | death | 0 (0 to 0) | 0.04 (0.01 to 0.07) | 0 (0 to 0) | 0.03 (0.02 to 0.05) | 0.41 (0.01 to 1.27) | -0.6 (-1.07 to -0.12) |
| Grenada | Chronic lymphoid leukemia | death | 0 (0 to 0) | 0.04 (0.01 to 0.08) | 0 (0 to 0) | 0.04 (0.02 to 0.07) | 0.7 (0.16 to 1.8) | 0.62 (-0.14 to 1.39) |
| Grenada | Other leukemia | death | 0 (0 to 0) | 0.14 (0.05 to 0.26) | 0 (0 to 0) | 0.19 (0.09 to 0.33) | 1.22 (0.69 to 2.48) | 1.2 (1.08 to 1.31) |
| Grenada | Leukemia | DALYs | 0.005 (0.002 to 0.01) | 8.14 (3.21 to 15.42) | 0.012 (0.006 to 0.02) | 10.49 (4.96 to 17.37) | 1.22 (0.69 to 2.38) | 0.97 (0.74 to 1.2) |
| Grenada | Acute myeloid leukemia | DALYs | 0.001 (0 to 0.001) | 0.98 (0.37 to 1.98) | 0.003 (0.001 to 0.005) | 2.28 (1.07 to 3.89) | 3.06 (1.8 to 5.8) | 3.21 (2.94 to 3.49) |
| Grenada | Acute lymphoid leukemia | DALYs | 0 (0 to 0.001) | 0.48 (0.17 to 0.98) | 0.001 (0 to 0.001) | 0.65 (0.31 to 1.15) | 1.26 (0.47 to 3.13) | 0.94 (0.64 to 1.25) |
| Grenada | Chronic myeloid leukemia | DALYs | 0.001 (0 to 0.002) | 1.24 (0.48 to 2.35) | 0.001 (0.001 to 0.002) | 0.99 (0.49 to 1.68) | 0.4 (0 to 1.31) | -0.76 (-1.22 to -0.29) |
| Grenada | Chronic lymphoid leukemia | DALYs | 0.001 (0 to 0.001) | 0.97 (0.36 to 1.94) | 0.001 (0.001 to 0.002) | 1.07 (0.46 to 1.84) | 0.93 (0.33 to 2.08) | 0.66 (-0.03 to 1.36) |
| Grenada | Other leukemia | DALYs | 0.003 (0.001 to 0.006) | 4.48 (1.7 to 8.45) | 0.006 (0.003 to 0.01) | 5.49 (2.62 to 9.09) | 1.1 (0.58 to 2.28) | 0.74 (0.6 to 0.88) |
| Guam | Leukemia | death | 0 (0 to 0.001) | 0.37 (0.17 to 0.65) | 0.001 (0 to 0.001) | 0.33 (0.16 to 0.55) | 1 (0.59 to 1.61) | -0.56 (-0.74 to -0.38) |
| Guam | Acute myeloid leukemia | death | 0 (0 to 0) | 0.15 (0.07 to 0.26) | 0 (0 to 0) | 0.09 (0.04 to 0.18) | 0.2 (-0.19 to 1.62) | -1.84 (-2.08 to -1.59) |
| Guam | Acute lymphoid leukemia | death | 0 (0 to 0) | 0.02 (0.01 to 0.03) | 0 (0 to 0) | 0.02 (0.01 to 0.03) | 0.61 (0.08 to 1.56) | -0.28 (-0.68 to 0.12) |
| Guam | Chronic myeloid leukemia | death | 0 (0 to 0) | 0.04 (0.02 to 0.07) | 0 (0 to 0) | 0.03 (0.02 to 0.06) | 0.84 (0.28 to 1.66) | -0.81 (-1.2 to -0.42) |
| Guam | Chronic lymphoid leukemia | death | 0 (0 to 0) | 0.01 (0 to 0.02) | 0 (0 to 0) | 0 (0 to 0.01) | 0.29 (-0.12 to 0.89) | -3.43 (-4.39 to -2.47) |
| Guam | Other leukemia | death | 0 (0 to 0) | 0.16 (0.07 to 0.29) | 0 (0 to 0.001) | 0.18 (0.09 to 0.31) | 2.3 (1.36 to 3.65) | 0.56 (0.06 to 1.06) |
| Guam | Leukemia | DALYs | 0.011 (0.005 to 0.019) | 10.41 (4.86 to 17.5) | 0.019 (0.01 to 0.032) | 10.63 (5.41 to 17.71) | 0.7 (0.3 to 1.28) | 0.01 (-0.09 to 0.11) |
| Guam | Acute myeloid leukemia | DALYs | 0.006 (0.003 to 0.01) | 4.92 (2.31 to 8.45) | 0.007 (0.003 to 0.012) | 3.69 (1.78 to 6.68) | 0.12 (-0.25 to 1.27) | -1.19 (-1.43 to -0.96) |
| Guam | Acute lymphoid leukemia | DALYs | 0.001 (0 to 0.001) | 0.62 (0.28 to 1.07) | 0.001 (0.001 to 0.002) | 0.66 (0.34 to 1.16) | 0.42 (-0.06 to 1.23) | -0.01 (-0.39 to 0.38) |
| Guam | Chronic myeloid leukemia | DALYs | 0.001 (0.001 to 0.002) | 1.14 (0.52 to 1.94) | 0.002 (0.001 to 0.003) | 1.11 (0.56 to 1.85) | 0.78 (0.23 to 1.57) | -0.17 (-0.54 to 0.2) |
| Guam | Chronic lymphoid leukemia | DALYs | 0 (0 to 0) | 0.23 (0.1 to 0.41) | 0 (0 to 0.001) | 0.16 (0.08 to 0.28) | 0.46 (0.01 to 1.08) | -1.77 (-2.59 to -0.94) |
| Guam | Other leukemia | DALYs | 0.003 (0.001 to 0.006) | 3.5 (1.57 to 6.32) | 0.009 (0.005 to 0.015) | 5.01 (2.49 to 8.39) | 1.8 (0.95 to 3.07) | 1.44 (1.01 to 1.88) |
| Guatemala | Leukemia | death | 0.005 (0.001 to 0.01) | 0.11 (0.03 to 0.23) | 0.034 (0.014 to 0.066) | 0.27 (0.11 to 0.51) | 6.37 (3.8 to 12.16) | 3.18 (2.8 to 3.56) |
| Guatemala | Acute myeloid leukemia | death | 0 (0 to 0.001) | 0.01 (0 to 0.01) | 0.005 (0.002 to 0.01) | 0.04 (0.02 to 0.08) | 22.11 (10.29 to 45.55) | 8.34 (7.82 to 8.86) |
| Guatemala | Acute lymphoid leukemia | death | 0 (0 to 0.001) | 0 (0 to 0.01) | 0.008 (0.003 to 0.015) | 0.05 (0.02 to 0.11) | 31.66 (8.12 to 75.86) | 10.78 (9.85 to 11.72) |
| Guatemala | Chronic myeloid leukemia | death | 0 (0 to 0) | 0 (0 to 0.01) | 0.001 (0.001 to 0.003) | 0.01 (0 to 0.02) | 6.4 (3.69 to 12.7) | 3.51 (3.2 to 3.82) |
| Guatemala | Chronic lymphoid leukemia | death | 0 (0 to 0) | 0 (0 to 0) | 0.001 (0 to 0.001) | 0.01 (0 to 0.01) | 9.92 (5.8 to 20.45) | 4.5 (4.04 to 4.97) |
| Guatemala | Other leukemia | death | 0.004 (0.001 to 0.009) | 0.09 (0.03 to 0.19) | 0.02 (0.008 to 0.037) | 0.16 (0.07 to 0.3) | 3.94 (2.22 to 7.87) | 1.49 (0.95 to 2.03) |
| Guatemala | Leukemia | DALYs | 0.185 (0.052 to 0.404) | 3.6 (1.05 to 7.74) | 1.328 (0.545 to 2.575) | 9.09 (3.81 to 17.47) | 6.17 (3.55 to 12.13) | 3.15 (2.77 to 3.53) |
| Guatemala | Acute myeloid leukemia | DALYs | 0.009 (0.002 to 0.02) | 0.17 (0.05 to 0.39) | 0.205 (0.078 to 0.405) | 1.39 (0.54 to 2.7) | 22.8 (10.29 to 48.51) | 8.51 (7.96 to 9.05) |
| Guatemala | Acute lymphoid leukemia | DALYs | 0.01 (0.002 to 0.03) | 0.18 (0.04 to 0.52) | 0.331 (0.121 to 0.677) | 2.09 (0.79 to 4.13) | 32.38 (8.34 to 81.51) | 10.89 (9.95 to 11.84) |
| Guatemala | Chronic myeloid leukemia | DALYs | 0.006 (0.002 to 0.014) | 0.14 (0.04 to 0.3) | 0.044 (0.018 to 0.082) | 0.34 (0.14 to 0.62) | 5.88 (3.26 to 12.18) | 3.39 (3.08 to 3.71) |
| Guatemala | Chronic lymphoid leukemia | DALYs | 0.002 (0.001 to 0.005) | 0.05 (0.01 to 0.11) | 0.021 (0.009 to 0.041) | 0.17 (0.07 to 0.33) | 8.95 (4.95 to 19.35) | 4.52 (4.06 to 4.99) |
| Guatemala | Other leukemia | DALYs | 0.158 (0.044 to 0.344) | 3.06 (0.88 to 6.62) | 0.726 (0.295 to 1.402) | 5.11 (2.1 to 9.78) | 3.59 (1.98 to 7.49) | 1.24 (0.69 to 1.8) |
| Guinea | Leukemia | death | 0.003 (0.001 to 0.005) | 0.07 (0.02 to 0.15) | 0.007 (0.003 to 0.013) | 0.11 (0.05 to 0.21) | 1.68 (0.91 to 3.26) | 1.57 (1.55 to 1.6) |
| Guinea | Acute myeloid leukemia | death | 0 (0 to 0.001) | 0.01 (0 to 0.01) | 0.001 (0 to 0.002) | 0.01 (0 to 0.02) | 2.3 (1.13 to 4.85) | 2.04 (2 to 2.07) |
| Guinea | Acute lymphoid leukemia | death | 0 (0 to 0) | 0 (0 to 0.01) | 0 (0 to 0.001) | 0.01 (0 to 0.01) | 2.49 (1.23 to 5.14) | 2.13 (2.1 to 2.16) |
| Guinea | Chronic myeloid leukemia | death | 0.001 (0 to 0.001) | 0.02 (0 to 0.03) | 0.001 (0.001 to 0.003) | 0.02 (0.01 to 0.04) | 1.66 (0.74 to 3.39) | 1.39 (1.33 to 1.46) |
| Guinea | Chronic lymphoid leukemia | death | 0.001 (0 to 0.002) | 0.02 (0.01 to 0.05) | 0.002 (0.001 to 0.004) | 0.03 (0.01 to 0.07) | 1.53 (0.76 to 3.27) | 1.61 (1.57 to 1.66) |
| Guinea | Other leukemia | death | 0.001 (0 to 0.002) | 0.03 (0.01 to 0.06) | 0.002 (0.001 to 0.004) | 0.04 (0.01 to 0.07) | 1.52 (0.72 to 3.17) | 1.43 (1.38 to 1.48) |
| Guinea | Leukemia | DALYs | 0.08 (0.026 to 0.168) | 2.12 (0.7 to 4.44) | 0.228 (0.096 to 0.429) | 3.31 (1.42 to 6.19) | 1.87 (1.02 to 3.62) | 1.64 (1.61 to 1.66) |
| Guinea | Acute myeloid leukemia | DALYs | 0.008 (0.002 to 0.018) | 0.21 (0.06 to 0.45) | 0.03 (0.012 to 0.06) | 0.39 (0.15 to 0.76) | 2.63 (1.4 to 5.53) | 2.2 (2.17 to 2.22) |
| Guinea | Acute lymphoid leukemia | DALYs | 0.006 (0.002 to 0.013) | 0.13 (0.04 to 0.3) | 0.022 (0.008 to 0.045) | 0.24 (0.09 to 0.49) | 2.71 (1.31 to 5.82) | 2.18 (2.14 to 2.21) |
| Guinea | Chronic myeloid leukemia | DALYs | 0.021 (0.007 to 0.044) | 0.53 (0.17 to 1.13) | 0.058 (0.023 to 0.113) | 0.77 (0.3 to 1.49) | 1.78 (0.81 to 3.6) | 1.41 (1.34 to 1.49) |
| Guinea | Chronic lymphoid leukemia | DALYs | 0.018 (0.006 to 0.039) | 0.54 (0.17 to 1.14) | 0.048 (0.018 to 0.093) | 0.84 (0.32 to 1.63) | 1.59 (0.77 to 3.49) | 1.62 (1.6 to 1.65) |
| Guinea | Other leukemia | DALYs | 0.026 (0.008 to 0.059) | 0.71 (0.23 to 1.57) | 0.071 (0.028 to 0.138) | 1.06 (0.41 to 2.05) | 1.7 (0.83 to 3.58) | 1.51 (1.47 to 1.56) |
| Guinea-Bissau | Leukemia | death | 0 (0 to 0.001) | 0.1 (0.02 to 0.22) | 0.001 (0.001 to 0.003) | 0.16 (0.06 to 0.32) | 2.15 (1.1 to 4.82) | 1.85 (1.74 to 1.96) |
| Guinea-Bissau | Acute myeloid leukemia | death | 0 (0 to 0) | 0.01 (0 to 0.03) | 0 (0 to 0) | 0.02 (0.01 to 0.05) | 2.89 (1.42 to 6.66) | 2.56 (2.45 to 2.67) |
| Guinea-Bissau | Acute lymphoid leukemia | death | 0 (0 to 0) | 0 (0 to 0.01) | 0 (0 to 0) | 0.01 (0 to 0.02) | 2.45 (1.14 to 6.3) | 1.75 (1.69 to 1.81) |
| Guinea-Bissau | Chronic myeloid leukemia | death | 0 (0 to 0) | 0.02 (0 to 0.05) | 0 (0 to 0.001) | 0.03 (0.01 to 0.06) | 1.83 (0.79 to 4.72) | 1.23 (1.15 to 1.31) |
| Guinea-Bissau | Chronic lymphoid leukemia | death | 0 (0 to 0) | 0.02 (0 to 0.04) | 0 (0 to 0) | 0.03 (0.01 to 0.07) | 2.12 (0.96 to 5.61) | 2.15 (2.03 to 2.26) |
| Guinea-Bissau | Other leukemia | death | 0 (0 to 0) | 0.04 (0.01 to 0.1) | 0.001 (0 to 0.001) | 0.07 (0.02 to 0.14) | 2.06 (0.97 to 4.81) | 1.78 (1.64 to 1.92) |
| Guinea-Bissau | Leukemia | DALYs | 0.016 (0.004 to 0.037) | 2.96 (0.75 to 6.96) | 0.051 (0.019 to 0.102) | 4.87 (1.85 to 9.68) | 2.25 (1.14 to 5.1) | 1.77 (1.65 to 1.88) |
| Guinea-Bissau | Acute myeloid leukemia | DALYs | 0.002 (0.001 to 0.006) | 0.38 (0.09 to 0.94) | 0.009 (0.003 to 0.018) | 0.77 (0.27 to 1.59) | 2.9 (1.44 to 6.67) | 2.38 (2.27 to 2.48) |
| Guinea-Bissau | Acute lymphoid leukemia | DALYs | 0.001 (0 to 0.003) | 0.18 (0.04 to 0.47) | 0.004 (0.001 to 0.009) | 0.3 (0.09 to 0.65) | 2.59 (1.17 to 6.79) | 1.8 (1.72 to 1.89) |
| Guinea-Bissau | Chronic myeloid leukemia | DALYs | 0.004 (0.001 to 0.011) | 0.75 (0.17 to 1.83) | 0.012 (0.004 to 0.026) | 1.04 (0.36 to 2.12) | 1.91 (0.8 to 5.04) | 1.21 (1.11 to 1.31) |
| Guinea-Bissau | Chronic lymphoid leukemia | DALYs | 0.002 (0 to 0.004) | 0.44 (0.11 to 1.04) | 0.006 (0.002 to 0.012) | 0.79 (0.28 to 1.61) | 2.15 (0.92 to 5.89) | 2.06 (1.95 to 2.17) |
| Guinea-Bissau | Other leukemia | DALYs | 0.006 (0.001 to 0.015) | 1.21 (0.3 to 2.98) | 0.019 (0.007 to 0.042) | 1.98 (0.67 to 4.17) | 2.21 (1.04 to 5.15) | 1.75 (1.59 to 1.91) |
| Guyana | Leukemia | death | 0.001 (0 to 0.002) | 0.18 (0.07 to 0.34) | 0.002 (0.001 to 0.003) | 0.28 (0.13 to 0.5) | 1.43 (0.7 to 2.74) | 1.64 (1.41 to 1.87) |
| Guyana | Acute myeloid leukemia | death | 0 (0 to 0) | 0.01 (0 to 0.02) | 0 (0 to 0) | 0.02 (0.01 to 0.04) | 1.89 (0.81 to 3.9) | 2.29 (2.12 to 2.46) |
| Guyana | Acute lymphoid leukemia | death | 0 (0 to 0) | 0.02 (0.01 to 0.04) | 0 (0 to 0.001) | 0.04 (0.02 to 0.08) | 2.23 (1.12 to 4.52) | 3.08 (2.77 to 3.38) |
| Guyana | Chronic myeloid leukemia | death | 0 (0 to 0) | 0.05 (0.02 to 0.09) | 0 (0 to 0.001) | 0.06 (0.03 to 0.1) | 0.85 (0.24 to 1.94) | 0.8 (0.66 to 0.93) |
| Guyana | Chronic lymphoid leukemia | death | 0 (0 to 0) | 0.01 (0 to 0.01) | 0 (0 to 0) | 0.03 (0.01 to 0.05) | 5.97 (3.44 to 10.04) | 4.47 (3.57 to 5.38) |
| Guyana | Other leukemia | death | 0 (0 to 0.001) | 0.09 (0.04 to 0.18) | 0.001 (0 to 0.002) | 0.13 (0.06 to 0.23) | 1.17 (0.51 to 2.3) | 1.15 (0.89 to 1.41) |
| Guyana | Leukemia | DALYs | 0.029 (0.011 to 0.057) | 5.6 (2.19 to 10.76) | 0.07 (0.033 to 0.125) | 9.38 (4.36 to 16.83) | 1.4 (0.64 to 2.81) | 1.79 (1.52 to 2.07) |
| Guyana | Acute myeloid leukemia | DALYs | 0.002 (0.001 to 0.005) | 0.42 (0.16 to 0.83) | 0.007 (0.003 to 0.012) | 0.87 (0.39 to 1.63) | 1.89 (0.78 to 4.06) | 2.55 (2.35 to 2.76) |
| Guyana | Acute lymphoid leukemia | DALYs | 0.005 (0.002 to 0.009) | 0.72 (0.26 to 1.42) | 0.014 (0.007 to 0.026) | 1.79 (0.85 to 3.21) | 2.14 (1.01 to 4.55) | 3.22 (2.87 to 3.57) |
| Guyana | Chronic myeloid leukemia | DALYs | 0.008 (0.003 to 0.015) | 1.48 (0.59 to 2.81) | 0.014 (0.006 to 0.025) | 1.85 (0.84 to 3.34) | 0.82 (0.17 to 2.01) | 0.92 (0.76 to 1.09) |
| Guyana | Chronic lymphoid leukemia | DALYs | 0.001 (0 to 0.001) | 0.16 (0.06 to 0.32) | 0.005 (0.002 to 0.009) | 0.71 (0.31 to 1.3) | 6.16 (3.4 to 10.45) | 4.64 (3.69 to 5.59) |
| Guyana | Other leukemia | DALYs | 0.014 (0.005 to 0.027) | 2.81 (1.09 to 5.33) | 0.031 (0.014 to 0.055) | 4.17 (1.88 to 7.54) | 1.17 (0.46 to 2.44) | 1.29 (0.98 to 1.6) |
| Haiti | Leukemia | death | 0.005 (0.001 to 0.012) | 0.14 (0.04 to 0.31) | 0.013 (0.004 to 0.029) | 0.16 (0.06 to 0.35) | 1.51 (0.75 to 3.15) | 0.86 (0.72 to 0.99) |
| Haiti | Acute myeloid leukemia | death | 0.001 (0 to 0.001) | 0.01 (0 to 0.04) | 0.002 (0.001 to 0.005) | 0.02 (0.01 to 0.06) | 2.53 (1.19 to 5.24) | 1.96 (1.85 to 2.07) |
| Haiti | Acute lymphoid leukemia | death | 0 (0 to 0.001) | 0.01 (0 to 0.02) | 0.001 (0 to 0.002) | 0.01 (0 to 0.02) | 2.11 (0.95 to 4.55) | 1.43 (1.29 to 1.57) |
| Haiti | Chronic myeloid leukemia | death | 0.001 (0 to 0.002) | 0.02 (0 to 0.05) | 0.002 (0 to 0.004) | 0.02 (0.01 to 0.05) | 1.09 (0.34 to 2.57) | 0.21 (0.07 to 0.35) |
| Haiti | Chronic lymphoid leukemia | death | 0 (0 to 0) | 0.01 (0 to 0.02) | 0.001 (0 to 0.001) | 0.01 (0 to 0.02) | 2.16 (1.09 to 4.4) | 1.75 (1.61 to 1.9) |
| Haiti | Other leukemia | death | 0.003 (0.001 to 0.008) | 0.09 (0.02 to 0.21) | 0.008 (0.002 to 0.017) | 0.1 (0.03 to 0.22) | 1.33 (0.64 to 2.9) | 0.65 (0.51 to 0.79) |
| Haiti | Leukemia | DALYs | 0.201 (0.053 to 0.471) | 4.62 (1.28 to 10.63) | 0.49 (0.161 to 1.077) | 5.14 (1.75 to 11.2) | 1.44 (0.67 to 3.21) | 0.72 (0.58 to 0.85) |
| Haiti | Acute myeloid leukemia | DALYs | 0.024 (0.006 to 0.06) | 0.53 (0.14 to 1.33) | 0.083 (0.024 to 0.194) | 0.83 (0.25 to 1.95) | 2.52 (1.14 to 5.17) | 1.89 (1.79 to 2) |
| Haiti | Acute lymphoid leukemia | DALYs | 0.013 (0.003 to 0.043) | 0.28 (0.07 to 0.87) | 0.042 (0.013 to 0.105) | 0.4 (0.12 to 0.94) | 2.18 (0.87 to 5.07) | 1.5 (1.36 to 1.63) |
| Haiti | Chronic myeloid leukemia | DALYs | 0.029 (0.007 to 0.077) | 0.68 (0.17 to 1.71) | 0.06 (0.017 to 0.146) | 0.63 (0.18 to 1.49) | 1.04 (0.3 to 2.6) | 0.07 (-0.07 to 0.21) |
| Haiti | Chronic lymphoid leukemia | DALYs | 0.005 (0.002 to 0.013) | 0.15 (0.04 to 0.36) | 0.017 (0.006 to 0.037) | 0.23 (0.07 to 0.5) | 2.14 (1.09 to 4.47) | 1.71 (1.55 to 1.88) |
| Haiti | Other leukemia | DALYs | 0.129 (0.027 to 0.315) | 2.98 (0.67 to 7.05) | 0.288 (0.079 to 0.657) | 3.06 (0.89 to 6.78) | 1.23 (0.52 to 2.92) | 0.45 (0.31 to 0.59) |
| Honduras | Leukemia | death | 0.005 (0.002 to 0.011) | 0.22 (0.07 to 0.46) | 0.029 (0.012 to 0.056) | 0.46 (0.2 to 0.89) | 4.49 (2.71 to 8.19) | 2.66 (2.33 to 2.98) |
| Honduras | Acute myeloid leukemia | death | 0.001 (0 to 0.002) | 0.04 (0.01 to 0.08) | 0.006 (0.003 to 0.013) | 0.1 (0.04 to 0.19) | 5.53 (3.3 to 10.31) | 3.04 (2.75 to 3.33) |
| Honduras | Acute lymphoid leukemia | death | 0.001 (0 to 0.002) | 0.04 (0.01 to 0.08) | 0.006 (0.002 to 0.013) | 0.09 (0.03 to 0.19) | 5.27 (2.81 to 11.31) | 3.41 (3.03 to 3.79) |
| Honduras | Chronic myeloid leukemia | death | 0.001 (0 to 0.002) | 0.03 (0.01 to 0.06) | 0.004 (0.001 to 0.007) | 0.06 (0.02 to 0.11) | 4.17 (2.34 to 8.19) | 2.34 (1.88 to 2.8) |
| Honduras | Chronic lymphoid leukemia | death | 0 (0 to 0) | 0.01 (0 to 0.02) | 0.002 (0.001 to 0.004) | 0.04 (0.01 to 0.07) | 9.39 (5.6 to 16.4) | 4.83 (4.36 to 5.29) |
| Honduras | Other leukemia | death | 0.002 (0.001 to 0.005) | 0.1 (0.03 to 0.23) | 0.011 (0.004 to 0.022) | 0.18 (0.07 to 0.36) | 3.47 (1.78 to 6.75) | 1.95 (1.66 to 2.24) |
| Honduras | Leukemia | DALYs | 0.195 (0.056 to 0.426) | 7.04 (2.17 to 14.78) | 0.899 (0.371 to 1.761) | 12.67 (5.28 to 24.82) | 3.61 (1.99 to 7.35) | 2.12 (1.81 to 2.44) |
| Honduras | Acute myeloid leukemia | DALYs | 0.035 (0.01 to 0.075) | 1.29 (0.41 to 2.73) | 0.209 (0.083 to 0.415) | 2.85 (1.15 to 5.63) | 4.99 (2.81 to 10.27) | 2.84 (2.54 to 3.15) |
| Honduras | Acute lymphoid leukemia | DALYs | 0.04 (0.01 to 0.099) | 1.34 (0.38 to 3.07) | 0.211 (0.077 to 0.475) | 2.81 (1.02 to 6.1) | 4.24 (2.09 to 10) | 2.78 (2.37 to 3.19) |
| Honduras | Chronic myeloid leukemia | DALYs | 0.026 (0.008 to 0.057) | 0.96 (0.3 to 2.08) | 0.108 (0.043 to 0.212) | 1.57 (0.61 to 3.05) | 3.2 (1.6 to 7.08) | 1.64 (1.19 to 2.09) |
| Honduras | Chronic lymphoid leukemia | DALYs | 0.005 (0.002 to 0.01) | 0.22 (0.08 to 0.47) | 0.045 (0.019 to 0.083) | 0.74 (0.32 to 1.36) | 8.16 (4.83 to 14.88) | 4.45 (4.06 to 4.85) |
| Honduras | Other leukemia | DALYs | 0.089 (0.024 to 0.203) | 3.23 (0.95 to 7.12) | 0.326 (0.118 to 0.699) | 4.68 (1.74 to 9.6) | 2.66 (1.26 to 5.8) | 1.34 (1.08 to 1.6) |
| Hungary | Leukemia | death | 0.087 (0.045 to 0.139) | 0.61 (0.31 to 0.98) | 0.11 (0.058 to 0.179) | 0.58 (0.3 to 0.93) | 0.26 (0.02 to 0.54) | -0.36 (-0.68 to -0.03) |
| Hungary | Acute myeloid leukemia | death | 0.024 (0.012 to 0.04) | 0.17 (0.09 to 0.28) | 0.045 (0.023 to 0.074) | 0.24 (0.12 to 0.4) | 0.83 (0.38 to 1.29) | 1.25 (0.88 to 1.62) |
| Hungary | Acute lymphoid leukemia | death | 0.006 (0.003 to 0.01) | 0.05 (0.02 to 0.08) | 0.006 (0.003 to 0.01) | 0.04 (0.02 to 0.06) | -0.09 (-0.32 to 0.46) | -1.34 (-1.59 to -1.08) |
| Hungary | Chronic myeloid leukemia | death | 0.016 (0.008 to 0.025) | 0.11 (0.06 to 0.18) | 0.007 (0.003 to 0.011) | 0.04 (0.02 to 0.06) | -0.58 (-0.67 to -0.47) | -4.74 (-5.04 to -4.44) |
| Hungary | Chronic lymphoid leukemia | death | 0.024 (0.012 to 0.038) | 0.16 (0.09 to 0.26) | 0.033 (0.017 to 0.054) | 0.16 (0.08 to 0.26) | 0.39 (0.11 to 0.72) | -0.06 (-0.37 to 0.26) |
| Hungary | Other leukemia | death | 0.017 (0.009 to 0.028) | 0.12 (0.06 to 0.2) | 0.02 (0.01 to 0.033) | 0.1 (0.05 to 0.17) | 0.17 (-0.08 to 0.46) | -0.82 (-1.44 to -0.19) |
| Hungary | Leukemia | DALYs | 2.243 (1.165 to 3.552) | 16.27 (8.44 to 25.97) | 2.467 (1.297 to 3.982) | 14.52 (7.59 to 23.5) | 0.1 (-0.12 to 0.36) | -0.61 (-0.92 to -0.3) |
| Hungary | Acute myeloid leukemia | DALYs | 0.673 (0.337 to 1.084) | 4.97 (2.52 to 8) | 1.059 (0.548 to 1.759) | 6.51 (3.39 to 10.82) | 0.57 (0.18 to 1.01) | 0.91 (0.55 to 1.27) |
| Hungary | Acute lymphoid leukemia | DALYs | 0.201 (0.102 to 0.326) | 1.59 (0.81 to 2.59) | 0.177 (0.091 to 0.307) | 1.27 (0.65 to 2.19) | -0.12 (-0.34 to 0.42) | -1.15 (-1.41 to -0.89) |
| Hungary | Chronic myeloid leukemia | DALYs | 0.43 (0.222 to 0.698) | 3.19 (1.64 to 5.21) | 0.151 (0.078 to 0.245) | 0.93 (0.49 to 1.49) | -0.65 (-0.73 to -0.55) | -5.16 (-5.49 to -4.84) |
| Hungary | Chronic lymphoid leukemia | DALYs | 0.515 (0.276 to 0.823) | 3.46 (1.85 to 5.54) | 0.658 (0.341 to 1.076) | 3.42 (1.79 to 5.6) | 0.28 (0 to 0.62) | -0.14 (-0.45 to 0.17) |
| Hungary | Other leukemia | DALYs | 0.424 (0.219 to 0.677) | 3.04 (1.56 to 4.88) | 0.422 (0.215 to 0.696) | 2.4 (1.24 to 3.98) | 0 (-0.23 to 0.25) | -1.14 (-1.72 to -0.55) |
| Iceland | Leukemia | death | 0.001 (0 to 0.002) | 0.34 (0.16 to 0.6) | 0.002 (0.001 to 0.003) | 0.31 (0.15 to 0.53) | 0.74 (0.45 to 1.11) | -0.36 (-0.47 to -0.25) |
| Iceland | Acute myeloid leukemia | death | 0 (0 to 0.001) | 0.13 (0.06 to 0.23) | 0.001 (0 to 0.001) | 0.15 (0.07 to 0.26) | 1.19 (0.76 to 1.87) | 0.59 (0.49 to 0.7) |
| Iceland | Acute lymphoid leukemia | death | 0 (0 to 0) | 0.01 (0.01 to 0.03) | 0 (0 to 0) | 0.02 (0.01 to 0.03) | 0.93 (0.52 to 1.49) | -0.5 (-0.94 to -0.05) |
| Iceland | Chronic myeloid leukemia | death | 0 (0 to 0) | 0.03 (0.01 to 0.04) | 0 (0 to 0) | 0.01 (0.01 to 0.02) | 0 (-0.29 to 0.35) | -2.94 (-3.41 to -2.48) |
| Iceland | Chronic lymphoid leukemia | death | 0 (0 to 0.001) | 0.12 (0.05 to 0.22) | 0 (0 to 0.001) | 0.06 (0.03 to 0.11) | 0.03 (-0.24 to 0.37) | -1.98 (-2.39 to -1.56) |
| Iceland | Other leukemia | death | 0 (0 to 0) | 0.05 (0.02 to 0.08) | 0 (0 to 0.001) | 0.06 (0.03 to 0.11) | 1.73 (0.83 to 2.53) | 0.69 (0.08 to 1.31) |
| Iceland | Leukemia | DALYs | 0.024 (0.011 to 0.042) | 8.8 (4.03 to 15.11) | 0.038 (0.019 to 0.064) | 7.73 (3.82 to 12.98) | 0.56 (0.32 to 0.89) | -0.46 (-0.54 to -0.37) |
| Iceland | Acute myeloid leukemia | DALYs | 0.01 (0.004 to 0.017) | 3.64 (1.63 to 6.31) | 0.019 (0.009 to 0.032) | 4.02 (1.98 to 6.76) | 0.92 (0.57 to 1.47) | 0.42 (0.34 to 0.51) |
| Iceland | Acute lymphoid leukemia | DALYs | 0.002 (0.001 to 0.003) | 0.64 (0.29 to 1.13) | 0.003 (0.002 to 0.005) | 0.74 (0.37 to 1.25) | 0.83 (0.42 to 1.45) | -0.22 (-0.58 to 0.14) |
| Iceland | Chronic myeloid leukemia | DALYs | 0.002 (0.001 to 0.003) | 0.73 (0.33 to 1.28) | 0.002 (0.001 to 0.003) | 0.4 (0.19 to 0.67) | -0.03 (-0.29 to 0.27) | -2.65 (-2.98 to -2.31) |
| Iceland | Chronic lymphoid leukemia | DALYs | 0.007 (0.003 to 0.013) | 2.62 (1.16 to 4.61) | 0.007 (0.003 to 0.013) | 1.28 (0.62 to 2.26) | -0.04 (-0.3 to 0.3) | -2.09 (-2.49 to -1.69) |
| Iceland | Other leukemia | DALYs | 0.003 (0.001 to 0.006) | 1.17 (0.53 to 1.99) | 0.007 (0.003 to 0.012) | 1.29 (0.61 to 2.25) | 1.07 (0.43 to 1.66) | 0.11 (-0.43 to 0.66) |
| India | Leukemia | death | 0.222 (0.07 to 0.494) | 0.05 (0.01 to 0.1) | 1.218 (0.556 to 2.215) | 0.1 (0.05 to 0.19) | 4.49 (2.78 to 8.49) | 2.64 (2.45 to 2.82) |
| India | Acute myeloid leukemia | death | 0.046 (0.014 to 0.104) | 0.01 (0 to 0.02) | 0.333 (0.155 to 0.603) | 0.03 (0.01 to 0.05) | 6.29 (3.76 to 12.28) | 3.87 (3.69 to 4.04) |
| India | Acute lymphoid leukemia | death | 0.016 (0.005 to 0.038) | 0 (0 to 0.01) | 0.077 (0.035 to 0.139) | 0.01 (0 to 0.01) | 3.73 (2.11 to 7.24) | 2.52 (2.3 to 2.75) |
| India | Chronic myeloid leukemia | death | 0.076 (0.023 to 0.176) | 0.01 (0 to 0.03) | 0.314 (0.143 to 0.582) | 0.03 (0.01 to 0.05) | 3.13 (1.8 to 6.32) | 1.75 (1.57 to 1.92) |
| India | Chronic lymphoid leukemia | death | 0.038 (0.01 to 0.088) | 0.01 (0 to 0.02) | 0.308 (0.124 to 0.584) | 0.03 (0.01 to 0.06) | 7.17 (4.52 to 13.95) | 3.49 (3.25 to 3.73) |
| India | Other leukemia | death | 0.046 (0.013 to 0.111) | 0.01 (0 to 0.02) | 0.187 (0.084 to 0.351) | 0.02 (0.01 to 0.03) | 3.04 (1.69 to 7.3) | 1.38 (1.17 to 1.6) |
| India | Leukemia | DALYs | 7.819 (2.408 to 17.817) | 1.33 (0.42 to 2.98) | 38.293 (17.789 to 69.717) | 2.99 (1.38 to 5.42) | 3.9 (2.37 to 7.46) | 2.62 (2.47 to 2.78) |
| India | Acute myeloid leukemia | DALYs | 1.665 (0.522 to 3.894) | 0.27 (0.09 to 0.62) | 11.119 (5.153 to 20.131) | 0.85 (0.4 to 1.53) | 5.68 (3.32 to 11.37) | 3.86 (3.7 to 4.02) |
| India | Acute lymphoid leukemia | DALYs | 0.716 (0.206 to 1.713) | 0.1 (0.03 to 0.24) | 3.207 (1.468 to 5.832) | 0.23 (0.11 to 0.42) | 3.48 (1.89 to 7.22) | 2.57 (2.36 to 2.79) |
| India | Chronic myeloid leukemia | DALYs | 2.888 (0.881 to 6.715) | 0.46 (0.14 to 1.07) | 11.007 (4.973 to 20.259) | 0.83 (0.38 to 1.53) | 2.81 (1.58 to 5.9) | 1.76 (1.6 to 1.92) |
| India | Chronic lymphoid leukemia | DALYs | 0.976 (0.283 to 2.276) | 0.22 (0.06 to 0.5) | 7.282 (3.036 to 13.781) | 0.63 (0.26 to 1.2) | 6.46 (4.06 to 12.48) | 3.67 (3.48 to 3.86) |
| India | Other leukemia | DALYs | 1.573 (0.429 to 3.857) | 0.28 (0.08 to 0.67) | 5.678 (2.55 to 10.56) | 0.45 (0.2 to 0.84) | 2.61 (1.38 to 6.64) | 1.41 (1.22 to 1.59) |
| Indonesia | Leukemia | death | 0.092 (0.022 to 0.224) | 0.08 (0.02 to 0.19) | 0.465 (0.197 to 0.89) | 0.2 (0.08 to 0.38) | 4.05 (2.39 to 9.84) | 3.54 (3.42 to 3.65) |
| Indonesia | Acute myeloid leukemia | death | 0.013 (0.003 to 0.037) | 0.01 (0 to 0.03) | 0.096 (0.036 to 0.218) | 0.04 (0.01 to 0.09) | 6.48 (3.64 to 15.47) | 4.83 (4.73 to 4.93) |
| Indonesia | Acute lymphoid leukemia | death | 0.006 (0.001 to 0.021) | 0 (0 to 0.01) | 0.038 (0.016 to 0.078) | 0.02 (0.01 to 0.03) | 4.89 (2.45 to 12.77) | 4.39 (4.22 to 4.56) |
| Indonesia | Chronic myeloid leukemia | death | 0.006 (0.001 to 0.018) | 0.01 (0 to 0.01) | 0.03 (0.011 to 0.063) | 0.01 (0 to 0.03) | 3.68 (1.97 to 9.45) | 3.24 (3.16 to 3.33) |
| Indonesia | Chronic lymphoid leukemia | death | 0.002 (0 to 0.005) | 0 (0 to 0.01) | 0.018 (0.008 to 0.037) | 0.01 (0 to 0.02) | 8.41 (5.16 to 20.16) | 5.47 (5.35 to 5.58) |
| Indonesia | Other leukemia | death | 0.064 (0.014 to 0.165) | 0.05 (0.01 to 0.14) | 0.284 (0.119 to 0.542) | 0.12 (0.05 to 0.24) | 3.4 (1.9 to 8.47) | 3.07 (2.95 to 3.19) |
| Indonesia | Leukemia | DALYs | 3.67 (0.872 to 8.943) | 2.61 (0.62 to 6.36) | 16.486 (7.041 to 30.901) | 6.23 (2.65 to 11.79) | 3.49 (1.95 to 8.66) | 3.38 (3.19 to 3.57) |
| Indonesia | Acute myeloid leukemia | DALYs | 0.516 (0.121 to 1.515) | 0.36 (0.08 to 1.05) | 3.577 (1.372 to 8.24) | 1.31 (0.5 to 3.01) | 5.93 (3.23 to 14.2) | 4.8 (4.64 to 4.96) |
| Indonesia | Acute lymphoid leukemia | DALYs | 0.286 (0.062 to 0.954) | 0.18 (0.04 to 0.61) | 1.469 (0.623 to 3.075) | 0.54 (0.23 to 1.14) | 4.13 (1.89 to 11.19) | 4.13 (3.89 to 4.37) |
| Indonesia | Chronic myeloid leukemia | DALYs | 0.252 (0.054 to 0.719) | 0.18 (0.04 to 0.52) | 1.031 (0.39 to 2.233) | 0.39 (0.15 to 0.84) | 3.09 (1.53 to 8.3) | 2.96 (2.81 to 3.12) |
| Indonesia | Chronic lymphoid leukemia | DALYs | 0.059 (0.014 to 0.157) | 0.05 (0.01 to 0.13) | 0.506 (0.22 to 1.026) | 0.21 (0.09 to 0.43) | 7.64 (4.63 to 18.05) | 5.39 (5.23 to 5.54) |
| Indonesia | Other leukemia | DALYs | 2.557 (0.576 to 6.485) | 1.83 (0.41 to 4.65) | 9.902 (4.216 to 18.524) | 3.78 (1.59 to 7.17) | 2.87 (1.51 to 7.53) | 2.88 (2.67 to 3.08) |
| Iran (Islamic Republic of) | Leukemia | death | 0.109 (0.048 to 0.196) | 0.38 (0.17 to 0.69) | 0.36 (0.185 to 0.601) | 0.48 (0.25 to 0.8) | 2.31 (1.66 to 3.64) | 0.9 (0.72 to 1.07) |
| Iran (Islamic Republic of) | Acute myeloid leukemia | death | 0.029 (0.013 to 0.052) | 0.09 (0.04 to 0.15) | 0.11 (0.057 to 0.181) | 0.13 (0.07 to 0.22) | 2.8 (1.99 to 4.31) | 1.53 (1.38 to 1.69) |
| Iran (Islamic Republic of) | Acute lymphoid leukemia | death | 0.013 (0.006 to 0.023) | 0.04 (0.02 to 0.07) | 0.041 (0.021 to 0.068) | 0.05 (0.03 to 0.08) | 2.18 (1.35 to 3.57) | 1 (0.83 to 1.17) |
| Iran (Islamic Republic of) | Chronic myeloid leukemia | death | 0.016 (0.007 to 0.03) | 0.05 (0.02 to 0.1) | 0.038 (0.019 to 0.064) | 0.05 (0.02 to 0.08) | 1.34 (0.84 to 2.22) | -0.32 (-0.42 to -0.23) |
| Iran (Islamic Republic of) | Chronic lymphoid leukemia | death | 0.004 (0.002 to 0.008) | 0.02 (0.01 to 0.03) | 0.018 (0.009 to 0.03) | 0.02 (0.01 to 0.04) | 2.95 (2.2 to 4.35) | 1.09 (0.76 to 1.42) |
| Iran (Islamic Republic of) | Other leukemia | death | 0.046 (0.019 to 0.087) | 0.19 (0.08 to 0.35) | 0.153 (0.072 to 0.266) | 0.22 (0.11 to 0.39) | 2.31 (1.53 to 4.59) | 0.82 (0.47 to 1.17) |
| Iran (Islamic Republic of) | Leukemia | DALYs | 3.86 (1.676 to 6.941) | 11.17 (4.91 to 20.19) | 11.306 (5.642 to 19.053) | 13.44 (6.72 to 22.5) | 1.93 (1.33 to 3.24) | 0.72 (0.6 to 0.84) |
| Iran (Islamic Republic of) | Acute myeloid leukemia | DALYs | 1.149 (0.494 to 2.099) | 3.06 (1.36 to 5.48) | 4.069 (2.063 to 6.743) | 4.52 (2.3 to 7.41) | 2.54 (1.75 to 4.06) | 1.43 (1.3 to 1.56) |
| Iran (Islamic Republic of) | Acute lymphoid leukemia | DALYs | 0.53 (0.222 to 0.999) | 1.34 (0.58 to 2.43) | 1.623 (0.817 to 2.694) | 1.83 (0.93 to 3.04) | 2.06 (1.1 to 3.62) | 1.12 (1.02 to 1.22) |
| Iran (Islamic Republic of) | Chronic myeloid leukemia | DALYs | 0.587 (0.248 to 1.098) | 1.69 (0.73 to 3.13) | 1.236 (0.607 to 2.099) | 1.43 (0.71 to 2.43) | 1.11 (0.61 to 2.07) | -0.57 (-0.68 to -0.46) |
| Iran (Islamic Republic of) | Chronic lymphoid leukemia | DALYs | 0.148 (0.061 to 0.266) | 0.44 (0.19 to 0.79) | 0.525 (0.25 to 0.89) | 0.65 (0.31 to 1.11) | 2.56 (1.82 to 3.9) | 0.99 (0.72 to 1.26) |
| Iran (Islamic Republic of) | Other leukemia | DALYs | 1.447 (0.59 to 2.682) | 4.64 (1.85 to 8.74) | 3.854 (1.79 to 6.731) | 5.01 (2.31 to 8.67) | 1.66 (1.05 to 3.72) | 0.4 (0.1 to 0.71) |
| Iraq | Leukemia | death | 0.058 (0.026 to 0.1) | 0.67 (0.31 to 1.17) | 0.172 (0.085 to 0.293) | 0.69 (0.33 to 1.16) | 1.99 (1.14 to 3.53) | 0.21 (-0.04 to 0.46) |
| Iraq | Acute myeloid leukemia | death | 0.006 (0.003 to 0.013) | 0.07 (0.03 to 0.14) | 0.025 (0.012 to 0.047) | 0.09 (0.04 to 0.17) | 2.89 (1.59 to 5.19) | 0.93 (0.66 to 1.21) |
| Iraq | Acute lymphoid leukemia | death | 0.003 (0.001 to 0.006) | 0.03 (0.01 to 0.06) | 0.009 (0.004 to 0.018) | 0.03 (0.01 to 0.06) | 2.44 (1.24 to 4.78) | 0.49 (0.31 to 0.68) |
| Iraq | Chronic myeloid leukemia | death | 0.007 (0.003 to 0.013) | 0.08 (0.03 to 0.14) | 0.014 (0.006 to 0.024) | 0.05 (0.02 to 0.09) | 0.94 (0.25 to 2.21) | -1.27 (-1.64 to -0.9) |
| Iraq | Chronic lymphoid leukemia | death | 0.001 (0 to 0.002) | 0.01 (0.01 to 0.02) | 0.004 (0.002 to 0.007) | 0.02 (0.01 to 0.03) | 3.14 (1.76 to 5.56) | 1.28 (0.88 to 1.68) |
| Iraq | Other leukemia | death | 0.041 (0.019 to 0.071) | 0.48 (0.22 to 0.85) | 0.12 (0.06 to 0.207) | 0.5 (0.25 to 0.84) | 1.97 (1.07 to 3.78) | 0.23 (0.01 to 0.46) |
| Iraq | Leukemia | DALYs | 2.016 (0.908 to 3.483) | 20.34 (9.42 to 34.76) | 5.957 (2.954 to 10.279) | 19.56 (9.75 to 33.69) | 1.96 (1.06 to 3.63) | -0.05 (-0.23 to 0.13) |
| Iraq | Acute myeloid leukemia | DALYs | 0.238 (0.099 to 0.488) | 2.31 (0.97 to 4.76) | 0.954 (0.428 to 1.802) | 2.88 (1.32 to 5.48) | 3.01 (1.62 to 5.53) | 0.91 (0.67 to 1.15) |
| Iraq | Acute lymphoid leukemia | DALYs | 0.114 (0.043 to 0.277) | 0.99 (0.38 to 2.34) | 0.377 (0.163 to 0.766) | 1.07 (0.48 to 2.12) | 2.3 (1.08 to 4.86) | 0.25 (0.12 to 0.37) |
| Iraq | Chronic myeloid leukemia | DALYs | 0.255 (0.106 to 0.487) | 2.51 (1.05 to 4.69) | 0.478 (0.211 to 0.868) | 1.53 (0.7 to 2.7) | 0.87 (0.19 to 2.14) | -1.58 (-1.85 to -1.3) |
| Iraq | Chronic lymphoid leukemia | DALYs | 0.03 (0.013 to 0.056) | 0.34 (0.15 to 0.64) | 0.124 (0.057 to 0.223) | 0.47 (0.22 to 0.83) | 3.11 (1.73 to 5.71) | 1.04 (0.72 to 1.36) |
| Iraq | Other leukemia | DALYs | 1.378 (0.643 to 2.419) | 14.19 (6.7 to 24.51) | 4.024 (1.985 to 7.225) | 13.61 (6.81 to 23.8) | 1.92 (0.98 to 3.88) | -0.06 (-0.23 to 0.1) |
| Ireland | Leukemia | death | 0.016 (0.007 to 0.028) | 0.39 (0.18 to 0.7) | 0.027 (0.013 to 0.045) | 0.36 (0.17 to 0.61) | 0.69 (0.44 to 1.05) | -0.56 (-0.78 to -0.33) |
| Ireland | Acute myeloid leukemia | death | 0.005 (0.002 to 0.009) | 0.12 (0.05 to 0.21) | 0.013 (0.006 to 0.022) | 0.17 (0.08 to 0.29) | 1.65 (1.04 to 2.33) | 1.43 (1.28 to 1.59) |
| Ireland | Acute lymphoid leukemia | death | 0.001 (0.001 to 0.002) | 0.03 (0.01 to 0.06) | 0.002 (0.001 to 0.003) | 0.02 (0.01 to 0.04) | 0.2 (-0.07 to 0.9) | -1.68 (-1.92 to -1.43) |
| Ireland | Chronic myeloid leukemia | death | 0.002 (0.001 to 0.004) | 0.06 (0.03 to 0.11) | 0.001 (0.001 to 0.002) | 0.02 (0.01 to 0.03) | -0.48 (-0.58 to -0.32) | -5.32 (-5.84 to -4.81) |
| Ireland | Chronic lymphoid leukemia | death | 0.004 (0.002 to 0.006) | 0.09 (0.04 to 0.16) | 0.007 (0.003 to 0.012) | 0.09 (0.04 to 0.15) | 0.91 (0.54 to 1.46) | -0.13 (-0.34 to 0.09) |
| Ireland | Other leukemia | death | 0.004 (0.002 to 0.007) | 0.09 (0.04 to 0.17) | 0.004 (0.002 to 0.008) | 0.06 (0.03 to 0.1) | 0.17 (-0.07 to 0.55) | -2.31 (-3.07 to -1.54) |
| Ireland | Leukemia | DALYs | 0.369 (0.165 to 0.647) | 9.34 (4.17 to 16.38) | 0.589 (0.288 to 0.977) | 8.51 (4.17 to 14.15) | 0.6 (0.37 to 0.91) | -0.6 (-0.84 to -0.36) |
| Ireland | Acute myeloid leukemia | DALYs | 0.12 (0.054 to 0.21) | 3.08 (1.36 to 5.39) | 0.284 (0.139 to 0.476) | 4.17 (2.04 to 6.96) | 1.37 (0.88 to 1.97) | 1.08 (0.91 to 1.26) |
| Ireland | Acute lymphoid leukemia | DALYs | 0.042 (0.018 to 0.075) | 1.12 (0.49 to 2.01) | 0.058 (0.027 to 0.101) | 0.97 (0.46 to 1.71) | 0.39 (0.06 to 1.2) | -0.79 (-0.99 to -0.59) |
| Ireland | Chronic myeloid leukemia | DALYs | 0.059 (0.026 to 0.104) | 1.55 (0.68 to 2.73) | 0.035 (0.016 to 0.061) | 0.52 (0.25 to 0.9) | -0.41 (-0.53 to -0.23) | -4.68 (-5.17 to -4.18) |
| Ireland | Chronic lymphoid leukemia | DALYs | 0.072 (0.032 to 0.126) | 1.72 (0.78 to 3.03) | 0.132 (0.062 to 0.226) | 1.74 (0.83 to 2.99) | 0.83 (0.48 to 1.33) | -0.17 (-0.41 to 0.07) |
| Ireland | Other leukemia | DALYs | 0.076 (0.035 to 0.135) | 1.87 (0.85 to 3.32) | 0.08 (0.038 to 0.14) | 1.11 (0.52 to 1.92) | 0.05 (-0.14 to 0.36) | -2.54 (-3.3 to -1.78) |
| Israel | Leukemia | death | 0.024 (0.011 to 0.042) | 0.51 (0.22 to 0.89) | 0.064 (0.03 to 0.109) | 0.54 (0.25 to 0.91) | 1.64 (1.23 to 2.14) | -0.19 (-0.41 to 0.04) |
| Israel | Acute myeloid leukemia | death | 0.009 (0.004 to 0.016) | 0.18 (0.08 to 0.33) | 0.028 (0.013 to 0.049) | 0.25 (0.11 to 0.42) | 2.2 (1.62 to 2.9) | 0.93 (0.74 to 1.12) |
| Israel | Acute lymphoid leukemia | death | 0.002 (0.001 to 0.003) | 0.04 (0.02 to 0.06) | 0.003 (0.001 to 0.005) | 0.03 (0.01 to 0.05) | 0.89 (0.53 to 1.66) | -1.35 (-1.75 to -0.95) |
| Israel | Chronic myeloid leukemia | death | 0.003 (0.001 to 0.005) | 0.06 (0.02 to 0.1) | 0.003 (0.001 to 0.005) | 0.02 (0.01 to 0.04) | 0.06 (-0.16 to 0.38) | -4.25 (-4.69 to -3.8) |
| Israel | Chronic lymphoid leukemia | death | 0.007 (0.003 to 0.012) | 0.15 (0.07 to 0.27) | 0.019 (0.008 to 0.033) | 0.15 (0.07 to 0.27) | 1.71 (1.18 to 2.45) | -0.46 (-0.77 to -0.14) |
| Israel | Other leukemia | death | 0.004 (0.002 to 0.007) | 0.09 (0.04 to 0.15) | 0.011 (0.005 to 0.019) | 0.09 (0.04 to 0.16) | 1.62 (1.03 to 2.34) | -0.26 (-0.54 to 0.02) |
| Israel | Leukemia | DALYs | 0.571 (0.249 to 0.99) | 12.04 (5.25 to 20.87) | 1.306 (0.614 to 2.203) | 11.94 (5.64 to 20.07) | 1.29 (0.99 to 1.68) | -0.44 (-0.68 to -0.19) |
| Israel | Acute myeloid leukemia | DALYs | 0.224 (0.095 to 0.396) | 4.74 (2.04 to 8.38) | 0.623 (0.285 to 1.065) | 5.81 (2.68 to 9.88) | 1.78 (1.32 to 2.39) | 0.55 (0.34 to 0.75) |
| Israel | Acute lymphoid leukemia | DALYs | 0.057 (0.024 to 0.102) | 1.23 (0.52 to 2.2) | 0.113 (0.053 to 0.194) | 1.15 (0.54 to 1.98) | 0.98 (0.59 to 1.82) | -0.85 (-1.25 to -0.44) |
| Israel | Chronic myeloid leukemia | DALYs | 0.064 (0.027 to 0.113) | 1.37 (0.58 to 2.42) | 0.058 (0.027 to 0.099) | 0.53 (0.25 to 0.9) | -0.1 (-0.27 to 0.16) | -4.48 (-4.94 to -4.02) |
| Israel | Chronic lymphoid leukemia | DALYs | 0.135 (0.061 to 0.238) | 2.81 (1.28 to 4.95) | 0.316 (0.146 to 0.547) | 2.7 (1.26 to 4.69) | 1.34 (0.89 to 1.95) | -0.69 (-1.02 to -0.36) |
| Israel | Other leukemia | DALYs | 0.09 (0.039 to 0.157) | 1.89 (0.81 to 3.31) | 0.195 (0.091 to 0.338) | 1.75 (0.82 to 2.99) | 1.17 (0.72 to 1.76) | -0.67 (-0.96 to -0.37) |
| Italy | Leukemia | death | 0.326 (0.142 to 0.578) | 0.38 (0.16 to 0.67) | 0.554 (0.256 to 0.944) | 0.37 (0.17 to 0.63) | 0.7 (0.48 to 0.96) | -0.16 (-0.28 to -0.05) |
| Italy | Acute myeloid leukemia | death | 0.073 (0.031 to 0.14) | 0.09 (0.04 to 0.16) | 0.215 (0.097 to 0.374) | 0.15 (0.07 to 0.27) | 1.93 (0.9 to 2.61) | 2.24 (1.92 to 2.57) |
| Italy | Acute lymphoid leukemia | death | 0.02 (0.008 to 0.035) | 0.02 (0.01 to 0.04) | 0.032 (0.015 to 0.057) | 0.03 (0.01 to 0.05) | 0.65 (0.42 to 1.03) | 0.38 (0.25 to 0.52) |
| Italy | Chronic myeloid leukemia | death | 0.05 (0.023 to 0.09) | 0.06 (0.03 to 0.11) | 0.035 (0.016 to 0.061) | 0.02 (0.01 to 0.04) | -0.3 (-0.4 to -0.13) | -4.22 (-4.55 to -3.9) |
| Italy | Chronic lymphoid leukemia | death | 0.064 (0.029 to 0.115) | 0.07 (0.03 to 0.13) | 0.125 (0.057 to 0.22) | 0.07 (0.03 to 0.13) | 0.95 (0.66 to 1.41) | -0.11 (-0.34 to 0.13) |
| Italy | Other leukemia | death | 0.119 (0.052 to 0.213) | 0.14 (0.06 to 0.24) | 0.146 (0.068 to 0.252) | 0.09 (0.04 to 0.16) | 0.23 (0.06 to 0.52) | -1.59 (-1.67 to -1.5) |
| Italy | Leukemia | DALYs | 7.779 (3.437 to 13.896) | 9.61 (4.25 to 17.24) | 10.43 (4.819 to 17.853) | 8.71 (4 to 15) | 0.34 (0.21 to 0.54) | -0.52 (-0.63 to -0.4) |
| Italy | Acute myeloid leukemia | DALYs | 1.826 (0.783 to 3.369) | 2.3 (0.97 to 4.22) | 4.253 (1.918 to 7.372) | 3.71 (1.66 to 6.4) | 1.33 (0.59 to 1.85) | 1.73 (1.43 to 2.03) |
| Italy | Acute lymphoid leukemia | DALYs | 0.689 (0.292 to 1.26) | 0.96 (0.41 to 1.77) | 1.075 (0.479 to 1.888) | 1.26 (0.57 to 2.28) | 0.56 (0.32 to 0.95) | 0.79 (0.54 to 1.04) |
| Italy | Chronic myeloid leukemia | DALYs | 1.249 (0.565 to 2.252) | 1.58 (0.71 to 2.87) | 0.702 (0.319 to 1.216) | 0.55 (0.25 to 0.94) | -0.44 (-0.51 to -0.3) | -4.59 (-4.96 to -4.22) |
| Italy | Chronic lymphoid leukemia | DALYs | 1.287 (0.585 to 2.306) | 1.43 (0.65 to 2.56) | 2.018 (0.919 to 3.525) | 1.36 (0.62 to 2.37) | 0.57 (0.36 to 0.96) | -0.44 (-0.7 to -0.18) |
| Italy | Other leukemia | DALYs | 2.729 (1.198 to 4.951) | 3.34 (1.44 to 6.1) | 2.382 (1.101 to 4.135) | 1.83 (0.84 to 3.17) | -0.13 (-0.23 to 0.08) | -2.37 (-2.49 to -2.24) |
| Jamaica | Leukemia | death | 0.003 (0.001 to 0.005) | 0.15 (0.07 to 0.27) | 0.013 (0.006 to 0.022) | 0.42 (0.21 to 0.73) | 3.65 (2.24 to 5.59) | 3.49 (2.81 to 4.18) |
| Jamaica | Acute myeloid leukemia | death | 0 (0 to 0.001) | 0.02 (0.01 to 0.04) | 0.003 (0.001 to 0.005) | 0.09 (0.04 to 0.15) | 5.88 (3.26 to 9.45) | 5.44 (4.82 to 6.05) |
| Jamaica | Acute lymphoid leukemia | death | 0 (0 to 0) | 0.01 (0 to 0.01) | 0.001 (0 to 0.001) | 0.02 (0.01 to 0.04) | 3.62 (1.91 to 6.84) | 3.53 (2.89 to 4.19) |
| Jamaica | Chronic myeloid leukemia | death | 0 (0 to 0) | 0.01 (0.01 to 0.02) | 0.001 (0.001 to 0.002) | 0.04 (0.02 to 0.07) | 4.7 (2.51 to 7.89) | 4.81 (4.13 to 5.49) |
| Jamaica | Chronic lymphoid leukemia | death | 0 (0 to 0) | 0.01 (0 to 0.01) | 0.001 (0 to 0.002) | 0.03 (0.02 to 0.06) | 9.82 (5.68 to 16.65) | 8.37 (7.42 to 9.33) |
| Jamaica | Other leukemia | death | 0.002 (0.001 to 0.003) | 0.11 (0.05 to 0.19) | 0.007 (0.004 to 0.012) | 0.24 (0.12 to 0.41) | 2.8 (1.64 to 4.41) | 2.4 (1.61 to 3.2) |
| Jamaica | Leukemia | DALYs | 0.086 (0.037 to 0.149) | 4.8 (2.08 to 8.3) | 0.424 (0.208 to 0.732) | 14.02 (6.88 to 24.28) | 3.93 (2.38 to 6.06) | 3.64 (2.88 to 4.42) |
| Jamaica | Acute myeloid leukemia | DALYs | 0.012 (0.005 to 0.023) | 0.68 (0.28 to 1.25) | 0.089 (0.043 to 0.156) | 2.94 (1.41 to 5.14) | 6.17 (3.44 to 10.04) | 5.65 (5.01 to 6.3) |
| Jamaica | Acute lymphoid leukemia | DALYs | 0.005 (0.002 to 0.01) | 0.28 (0.12 to 0.5) | 0.025 (0.011 to 0.045) | 0.82 (0.35 to 1.46) | 3.69 (1.84 to 7.32) | 3.79 (3.11 to 4.48) |
| Jamaica | Chronic myeloid leukemia | DALYs | 0.006 (0.003 to 0.012) | 0.36 (0.15 to 0.65) | 0.041 (0.02 to 0.07) | 1.33 (0.65 to 2.3) | 5.34 (2.88 to 8.92) | 5.11 (4.37 to 5.86) |
| Jamaica | Chronic lymphoid leukemia | DALYs | 0.002 (0.001 to 0.004) | 0.12 (0.05 to 0.23) | 0.023 (0.011 to 0.041) | 0.79 (0.38 to 1.39) | 9.99 (5.62 to 16.9) | 8.36 (7.41 to 9.32) |
| Jamaica | Other leukemia | DALYs | 0.06 (0.026 to 0.103) | 3.37 (1.46 to 5.79) | 0.245 (0.119 to 0.424) | 8.14 (3.95 to 14.05) | 3.12 (1.83 to 4.92) | 2.64 (1.75 to 3.54) |
| Japan | Leukemia | death | 0.203 (0.059 to 0.439) | 0.12 (0.04 to 0.27) | 0.336 (0.105 to 0.71) | 0.1 (0.03 to 0.21) | 0.66 (0.43 to 0.98) | -0.83 (-0.87 to -0.78) |
| Japan | Acute myeloid leukemia | death | 0.08 (0.023 to 0.174) | 0.05 (0.01 to 0.11) | 0.176 (0.056 to 0.375) | 0.05 (0.02 to 0.11) | 1.19 (0.85 to 1.63) | 0.41 (0.25 to 0.56) |
| Japan | Acute lymphoid leukemia | death | 0.021 (0.006 to 0.046) | 0.01 (0 to 0.03) | 0.025 (0.008 to 0.054) | 0.01 (0 to 0.02) | 0.23 (0.06 to 0.5) | -1.11 (-1.2 to -1.02) |
| Japan | Chronic myeloid leukemia | death | 0.031 (0.009 to 0.066) | 0.02 (0.01 to 0.04) | 0.017 (0.005 to 0.038) | 0 (0 to 0.01) | -0.44 (-0.53 to -0.29) | -5.6 (-5.91 to -5.28) |
| Japan | Chronic lymphoid leukemia | death | 0.006 (0.002 to 0.013) | 0 (0 to 0.01) | 0.011 (0.003 to 0.025) | 0 (0 to 0.01) | 1.01 (0.65 to 1.58) | -0.86 (-0.96 to -0.77) |
| Japan | Other leukemia | death | 0.065 (0.019 to 0.143) | 0.04 (0.01 to 0.09) | 0.106 (0.031 to 0.23) | 0.03 (0.01 to 0.06) | 0.62 (0.36 to 0.99) | -1.26 (-1.31 to -1.21) |
| Japan | Leukemia | DALYs | 6.005 (1.756 to 12.984) | 3.74 (1.08 to 8.16) | 6.699 (2.174 to 13.992) | 2.68 (0.86 to 5.58) | 0.12 (0.01 to 0.31) | -1.32 (-1.36 to -1.27) |
| Japan | Acute myeloid leukemia | DALYs | 2.382 (0.694 to 5.137) | 1.49 (0.43 to 3.23) | 3.479 (1.131 to 7.249) | 1.39 (0.45 to 2.91) | 0.46 (0.3 to 0.74) | -0.22 (-0.32 to -0.12) |
| Japan | Acute lymphoid leukemia | DALYs | 0.795 (0.223 to 1.766) | 0.53 (0.15 to 1.19) | 0.852 (0.27 to 1.809) | 0.46 (0.14 to 0.98) | 0.07 (-0.06 to 0.33) | -0.73 (-0.83 to -0.64) |
| Japan | Chronic myeloid leukemia | DALYs | 0.954 (0.277 to 2.092) | 0.6 (0.17 to 1.33) | 0.34 (0.104 to 0.736) | 0.13 (0.04 to 0.29) | -0.64 (-0.69 to -0.57) | -6.09 (-6.46 to -5.72) |
| Japan | Chronic lymphoid leukemia | DALYs | 0.129 (0.038 to 0.284) | 0.08 (0.02 to 0.17) | 0.197 (0.061 to 0.43) | 0.06 (0.02 to 0.13) | 0.53 (0.31 to 0.92) | -0.88 (-0.99 to -0.78) |
| Japan | Other leukemia | DALYs | 1.746 (0.507 to 3.836) | 1.05 (0.3 to 2.31) | 1.83 (0.574 to 3.852) | 0.63 (0.2 to 1.33) | 0.05 (-0.07 to 0.24) | -1.95 (-2.01 to -1.88) |
| Jordan | Leukemia | death | 0.011 (0.005 to 0.019) | 0.77 (0.36 to 1.3) | 0.053 (0.029 to 0.084) | 0.83 (0.45 to 1.31) | 3.78 (2.58 to 6.04) | 0.29 (0.11 to 0.48) |
| Jordan | Acute myeloid leukemia | death | 0 (0 to 0.001) | 0.02 (0.01 to 0.04) | 0.003 (0.001 to 0.004) | 0.04 (0.02 to 0.06) | 7.31 (3.61 to 12.18) | 2.35 (2.23 to 2.47) |
| Jordan | Acute lymphoid leukemia | death | 0 (0 to 0) | 0.01 (0 to 0.01) | 0.001 (0 to 0.001) | 0.01 (0 to 0.01) | 6.11 (2.56 to 10.9) | 1.59 (1.53 to 1.65) |
| Jordan | Chronic myeloid leukemia | death | 0 (0 to 0) | 0.01 (0.01 to 0.03) | 0.001 (0 to 0.001) | 0.01 (0.01 to 0.02) | 2.5 (1.12 to 4.74) | -1.03 (-1.21 to -0.84) |
| Jordan | Chronic lymphoid leukemia | death | 0 (0 to 0) | 0 (0 to 0.01) | 0 (0 to 0.001) | 0.01 (0 to 0.01) | 5.41 (2.87 to 9.2) | 1.05 (0.7 to 1.39) |
| Jordan | Other leukemia | death | 0.01 (0.005 to 0.017) | 0.73 (0.34 to 1.22) | 0.049 (0.027 to 0.076) | 0.77 (0.41 to 1.2) | 3.66 (2.47 to 5.98) | 0.22 (0.03 to 0.42) |
| Jordan | Leukemia | DALYs | 0.387 (0.18 to 0.656) | 21.25 (9.98 to 35.52) | 1.675 (0.916 to 2.611) | 20.69 (11.26 to 32.13) | 3.33 (2.21 to 5.34) | -0.17 (-0.39 to 0.05) |
| Jordan | Acute myeloid leukemia | DALYs | 0.013 (0.006 to 0.027) | 0.65 (0.29 to 1.27) | 0.103 (0.054 to 0.163) | 1.11 (0.58 to 1.75) | 6.72 (3.25 to 11.59) | 2.04 (1.96 to 2.12) |
| Jordan | Acute lymphoid leukemia | DALYs | 0.005 (0.002 to 0.011) | 0.21 (0.09 to 0.44) | 0.034 (0.017 to 0.055) | 0.33 (0.17 to 0.54) | 6 (2.35 to 11.28) | 1.63 (1.57 to 1.69) |
| Jordan | Chronic myeloid leukemia | DALYs | 0.009 (0.004 to 0.017) | 0.44 (0.2 to 0.83) | 0.028 (0.015 to 0.045) | 0.31 (0.17 to 0.51) | 2.18 (0.91 to 4.63) | -1.57 (-1.83 to -1.31) |
| Jordan | Chronic lymphoid leukemia | DALYs | 0.002 (0.001 to 0.004) | 0.12 (0.05 to 0.23) | 0.012 (0.006 to 0.021) | 0.15 (0.08 to 0.26) | 5.21 (2.82 to 8.84) | 0.76 (0.41 to 1.12) |
| Jordan | Other leukemia | DALYs | 0.358 (0.167 to 0.608) | 19.84 (9.33 to 33.03) | 1.498 (0.817 to 2.34) | 18.79 (10.25 to 29.38) | 3.18 (2.08 to 5.22) | -0.27 (-0.5 to -0.04) |
| Kazakhstan | Leukemia | death | 0.043 (0.02 to 0.072) | 0.32 (0.15 to 0.53) | 0.062 (0.031 to 0.1) | 0.35 (0.17 to 0.57) | 0.46 (0.23 to 0.8) | 0.75 (0.55 to 0.95) |
| Kazakhstan | Acute myeloid leukemia | death | 0.013 (0.006 to 0.023) | 0.09 (0.04 to 0.16) | 0.023 (0.011 to 0.038) | 0.12 (0.06 to 0.2) | 0.75 (0.38 to 1.29) | 1.67 (1.35 to 1.98) |
| Kazakhstan | Acute lymphoid leukemia | death | 0.007 (0.003 to 0.011) | 0.05 (0.02 to 0.08) | 0.01 (0.005 to 0.016) | 0.05 (0.03 to 0.09) | 0.5 (0.14 to 0.99) | 1.05 (0.81 to 1.28) |
| Kazakhstan | Chronic myeloid leukemia | death | 0.007 (0.003 to 0.012) | 0.05 (0.02 to 0.09) | 0.007 (0.004 to 0.012) | 0.04 (0.02 to 0.06) | 0.03 (-0.21 to 0.4) | -0.86 (-1.11 to -0.61) |
| Kazakhstan | Chronic lymphoid leukemia | death | 0.006 (0.003 to 0.009) | 0.04 (0.02 to 0.07) | 0.01 (0.005 to 0.016) | 0.06 (0.03 to 0.1) | 0.77 (0.29 to 1.47) | 1.27 (1.08 to 1.46) |
| Kazakhstan | Other leukemia | death | 0.01 (0.005 to 0.018) | 0.08 (0.04 to 0.14) | 0.013 (0.006 to 0.021) | 0.08 (0.04 to 0.13) | 0.21 (-0.04 to 0.55) | 0.12 (-0.08 to 0.33) |
| Kazakhstan | Leukemia | DALYs | 1.444 (0.672 to 2.487) | 10.02 (4.71 to 17.2) | 1.989 (0.983 to 3.189) | 10.49 (5.17 to 16.96) | 0.38 (0.15 to 0.75) | 0.46 (0.27 to 0.65) |
| Kazakhstan | Acute myeloid leukemia | DALYs | 0.485 (0.222 to 0.867) | 3.26 (1.5 to 5.81) | 0.8 (0.393 to 1.325) | 4.1 (2 to 6.79) | 0.65 (0.29 to 1.16) | 1.4 (1.12 to 1.68) |
| Kazakhstan | Acute lymphoid leukemia | DALYs | 0.265 (0.119 to 0.468) | 1.75 (0.78 to 3.07) | 0.373 (0.181 to 0.628) | 1.92 (0.93 to 3.23) | 0.41 (0.06 to 0.94) | 0.78 (0.57 to 0.98) |
| Kazakhstan | Chronic myeloid leukemia | DALYs | 0.24 (0.112 to 0.421) | 1.67 (0.79 to 2.92) | 0.23 (0.112 to 0.38) | 1.19 (0.58 to 1.97) | -0.04 (-0.27 to 0.31) | -1.25 (-1.58 to -0.92) |
| Kazakhstan | Chronic lymphoid leukemia | DALYs | 0.161 (0.073 to 0.275) | 1.2 (0.55 to 2.03) | 0.264 (0.129 to 0.436) | 1.45 (0.7 to 2.42) | 0.64 (0.2 to 1.34) | 0.88 (0.7 to 1.07) |
| Kazakhstan | Other leukemia | DALYs | 0.294 (0.135 to 0.498) | 2.15 (0.99 to 3.67) | 0.322 (0.149 to 0.526) | 1.82 (0.84 to 2.99) | 0.1 (-0.14 to 0.48) | -0.39 (-0.65 to -0.14) |
| Kenya | Leukemia | death | 0.005 (0.002 to 0.011) | 0.06 (0.02 to 0.11) | 0.032 (0.015 to 0.058) | 0.12 (0.06 to 0.23) | 4.98 (3.37 to 8.77) | 3.21 (3.06 to 3.36) |
| Kenya | Acute myeloid leukemia | death | 0.001 (0 to 0.002) | 0.01 (0 to 0.02) | 0.007 (0.004 to 0.013) | 0.02 (0.01 to 0.04) | 6.36 (4.15 to 10.89) | 3.83 (3.63 to 4.02) |
| Kenya | Acute lymphoid leukemia | death | 0 (0 to 0.001) | 0 (0 to 0.01) | 0.003 (0.002 to 0.006) | 0.01 (0 to 0.02) | 5.62 (3.65 to 10.35) | 3.63 (3.45 to 3.81) |
| Kenya | Chronic myeloid leukemia | death | 0.001 (0 to 0.002) | 0.01 (0 to 0.01) | 0.004 (0.002 to 0.008) | 0.01 (0.01 to 0.02) | 4.34 (2.84 to 7.59) | 2.61 (2.48 to 2.75) |
| Kenya | Chronic lymphoid leukemia | death | 0.001 (0 to 0.003) | 0.02 (0.01 to 0.04) | 0.008 (0.003 to 0.016) | 0.04 (0.02 to 0.08) | 5.22 (3.53 to 9.32) | 3.57 (3.38 to 3.77) |
| Kenya | Other leukemia | death | 0.002 (0.001 to 0.004) | 0.02 (0.01 to 0.04) | 0.009 (0.004 to 0.017) | 0.03 (0.02 to 0.06) | 4.17 (2.59 to 8.06) | 2.61 (2.46 to 2.76) |
| Kenya | Leukemia | DALYs | 0.198 (0.073 to 0.404) | 1.71 (0.63 to 3.47) | 1.18 (0.557 to 2.121) | 3.74 (1.76 to 6.74) | 4.95 (3.31 to 8.71) | 3.11 (2.97 to 3.25) |
| Kenya | Acute myeloid leukemia | DALYs | 0.041 (0.015 to 0.086) | 0.33 (0.12 to 0.68) | 0.301 (0.144 to 0.55) | 0.86 (0.41 to 1.55) | 6.28 (4.15 to 10.84) | 3.77 (3.6 to 3.94) |
| Kenya | Acute lymphoid leukemia | DALYs | 0.024 (0.008 to 0.052) | 0.16 (0.06 to 0.33) | 0.153 (0.071 to 0.292) | 0.38 (0.18 to 0.71) | 5.42 (3.46 to 10) | 3.54 (3.36 to 3.72) |
| Kenya | Chronic myeloid leukemia | DALYs | 0.033 (0.012 to 0.067) | 0.26 (0.1 to 0.53) | 0.172 (0.079 to 0.313) | 0.49 (0.23 to 0.89) | 4.2 (2.69 to 7.38) | 2.47 (2.31 to 2.63) |
| Kenya | Chronic lymphoid leukemia | DALYs | 0.032 (0.011 to 0.068) | 0.39 (0.13 to 0.83) | 0.204 (0.09 to 0.385) | 0.92 (0.39 to 1.75) | 5.31 (3.59 to 9.36) | 3.45 (3.28 to 3.63) |
| Kenya | Other leukemia | DALYs | 0.068 (0.022 to 0.146) | 0.58 (0.19 to 1.25) | 0.349 (0.16 to 0.647) | 1.09 (0.5 to 2.01) | 4.16 (2.53 to 8.11) | 2.6 (2.44 to 2.77) |
| Kiribati | Leukemia | death | 0 (0 to 0) | 0.31 (0.13 to 0.58) | 0 (0 to 0) | 0.3 (0.12 to 0.56) | 0.75 (0.28 to 1.44) | -0.47 (-0.68 to -0.26) |
| Kiribati | Acute myeloid leukemia | death | 0 (0 to 0) | 0.04 (0.01 to 0.07) | 0 (0 to 0) | 0.04 (0.01 to 0.07) | 1 (0.31 to 2.02) | -0.18 (-0.42 to 0.07) |
| Kiribati | Acute lymphoid leukemia | death | 0 (0 to 0) | 0.01 (0.01 to 0.03) | 0 (0 to 0) | 0.01 (0.01 to 0.03) | 0.91 (0.27 to 1.97) | -0.38 (-0.61 to -0.15) |
| Kiribati | Chronic myeloid leukemia | death | 0 (0 to 0) | 0.02 (0.01 to 0.04) | 0 (0 to 0) | 0.02 (0.01 to 0.04) | 0.86 (0.23 to 1.9) | -0.3 (-0.66 to 0.07) |
| Kiribati | Chronic lymphoid leukemia | death | 0 (0 to 0) | 0.01 (0 to 0.01) | 0 (0 to 0) | 0.01 (0 to 0.02) | 1.02 (0.33 to 2.21) | -0.05 (-0.25 to 0.15) |
| Kiribati | Other leukemia | death | 0 (0 to 0) | 0.23 (0.09 to 0.44) | 0 (0 to 0) | 0.22 (0.08 to 0.42) | 0.68 (0.23 to 1.36) | -0.55 (-0.74 to -0.36) |
| Kiribati | Leukemia | DALYs | 0.006 (0.003 to 0.01) | 11.07 (4.7 to 19.64) | 0.01 (0.005 to 0.018) | 10.49 (4.53 to 18.5) | 0.75 (0.25 to 1.49) | -0.52 (-0.75 to -0.3) |
| Kiribati | Acute myeloid leukemia | DALYs | 0.001 (0 to 0.001) | 1.39 (0.57 to 2.59) | 0.002 (0.001 to 0.003) | 1.5 (0.58 to 2.88) | 0.99 (0.26 to 2.08) | -0.17 (-0.43 to 0.09) |
| Kiribati | Acute lymphoid leukemia | DALYs | 0 (0 to 0.001) | 0.59 (0.22 to 1.11) | 0.001 (0 to 0.001) | 0.62 (0.23 to 1.21) | 0.99 (0.26 to 2.15) | -0.16 (-0.4 to 0.09) |
| Kiribati | Chronic myeloid leukemia | DALYs | 0 (0 to 0.001) | 0.78 (0.33 to 1.48) | 0.001 (0 to 0.001) | 0.78 (0.31 to 1.47) | 0.86 (0.19 to 2.01) | -0.35 (-0.72 to 0.02) |
| Kiribati | Chronic lymphoid leukemia | DALYs | 0 (0 to 0) | 0.19 (0.07 to 0.38) | 0 (0 to 0) | 0.2 (0.07 to 0.4) | 1.03 (0.37 to 2.15) | -0.16 (-0.36 to 0.04) |
| Kiribati | Other leukemia | DALYs | 0.004 (0.002 to 0.008) | 8.12 (3.38 to 14.82) | 0.007 (0.003 to 0.013) | 7.39 (3.08 to 13.52) | 0.67 (0.18 to 1.4) | -0.64 (-0.85 to -0.44) |
| Kuwait | Leukemia | death | 0.004 (0.002 to 0.006) | 0.53 (0.27 to 0.85) | 0.012 (0.007 to 0.019) | 0.44 (0.24 to 0.69) | 1.99 (1.44 to 2.79) | -0.23 (-0.63 to 0.18) |
| Kuwait | Acute myeloid leukemia | death | 0.001 (0 to 0.001) | 0.05 (0.03 to 0.09) | 0.003 (0.002 to 0.005) | 0.1 (0.05 to 0.16) | 5.11 (3.48 to 6.97) | 3.12 (2.47 to 3.77) |
| Kuwait | Acute lymphoid leukemia | death | 0 (0 to 0) | 0.02 (0.01 to 0.04) | 0.001 (0.001 to 0.002) | 0.03 (0.02 to 0.04) | 2.85 (1.75 to 4.38) | 1.22 (0.69 to 1.75) |
| Kuwait | Chronic myeloid leukemia | death | 0 (0 to 0) | 0.02 (0.01 to 0.04) | 0.001 (0 to 0.001) | 0.02 (0.01 to 0.03) | 2.33 (1.38 to 3.65) | 0.65 (0.13 to 1.18) |
| Kuwait | Chronic lymphoid leukemia | death | 0 (0 to 0) | 0.02 (0.01 to 0.04) | 0.001 (0 to 0.001) | 0.04 (0.02 to 0.06) | 5.5 (3.46 to 8.36) | 2.69 (2.21 to 3.17) |
| Kuwait | Other leukemia | death | 0.003 (0.002 to 0.005) | 0.41 (0.21 to 0.67) | 0.007 (0.004 to 0.01) | 0.26 (0.14 to 0.41) | 1.21 (0.76 to 1.95) | -1.43 (-1.86 to -0.99) |
| Kuwait | Leukemia | DALYs | 0.156 (0.083 to 0.244) | 14.47 (7.66 to 22.85) | 0.403 (0.23 to 0.617) | 11 (6.2 to 16.98) | 1.58 (1.11 to 2.28) | -0.67 (-1.07 to -0.27) |
| Kuwait | Acute myeloid leukemia | DALYs | 0.021 (0.011 to 0.034) | 1.74 (0.89 to 2.82) | 0.115 (0.064 to 0.182) | 2.8 (1.57 to 4.48) | 4.4 (2.92 to 6.2) | 2.69 (2.09 to 3.3) |
| Kuwait | Acute lymphoid leukemia | DALYs | 0.013 (0.007 to 0.021) | 0.88 (0.44 to 1.37) | 0.048 (0.027 to 0.076) | 1.02 (0.57 to 1.62) | 2.63 (1.55 to 4.19) | 1.08 (0.6 to 1.56) |
| Kuwait | Chronic myeloid leukemia | DALYs | 0.007 (0.004 to 0.012) | 0.62 (0.32 to 1.02) | 0.021 (0.012 to 0.033) | 0.55 (0.3 to 0.87) | 1.89 (1.04 to 3.12) | 0.22 (-0.3 to 0.75) |
| Kuwait | Chronic lymphoid leukemia | DALYs | 0.003 (0.002 to 0.006) | 0.51 (0.24 to 0.86) | 0.021 (0.011 to 0.034) | 0.84 (0.43 to 1.42) | 5.08 (3.24 to 7.69) | 2.61 (2.14 to 3.07) |
| Kuwait | Other leukemia | DALYs | 0.111 (0.058 to 0.176) | 10.72 (5.67 to 17.03) | 0.198 (0.11 to 0.312) | 5.79 (3.16 to 9.04) | 0.79 (0.42 to 1.41) | -2.17 (-2.64 to -1.7) |
| Kyrgyzstan | Leukemia | death | 0.007 (0.003 to 0.013) | 0.21 (0.09 to 0.38) | 0.01 (0.004 to 0.016) | 0.19 (0.09 to 0.34) | 0.35 (0.13 to 0.69) | -0.49 (-0.71 to -0.28) |
| Kyrgyzstan | Acute myeloid leukemia | death | 0.001 (0.001 to 0.003) | 0.04 (0.02 to 0.08) | 0.003 (0.002 to 0.006) | 0.07 (0.03 to 0.11) | 1.45 (0.55 to 2.31) | 2.11 (1.5 to 2.72) |
| Kyrgyzstan | Acute lymphoid leukemia | death | 0.001 (0 to 0.001) | 0.02 (0.01 to 0.04) | 0.001 (0.001 to 0.002) | 0.02 (0.01 to 0.04) | 0.76 (0.34 to 1.35) | 0.67 (0.22 to 1.11) |
| Kyrgyzstan | Chronic myeloid leukemia | death | 0.001 (0 to 0.001) | 0.02 (0.01 to 0.04) | 0.001 (0 to 0.001) | 0.01 (0.01 to 0.03) | 0.07 (-0.17 to 0.43) | -1.25 (-1.7 to -0.8) |
| Kyrgyzstan | Chronic lymphoid leukemia | death | 0.001 (0 to 0.001) | 0.02 (0.01 to 0.04) | 0.001 (0 to 0.001) | 0.01 (0.01 to 0.03) | -0.1 (-0.32 to 0.34) | -2.54 (-2.85 to -2.22) |
| Kyrgyzstan | Other leukemia | death | 0.004 (0.002 to 0.006) | 0.11 (0.05 to 0.19) | 0.003 (0.002 to 0.006) | 0.08 (0.03 to 0.13) | -0.03 (-0.2 to 0.26) | -1.57 (-1.73 to -1.41) |
| Kyrgyzstan | Leukemia | DALYs | 0.263 (0.111 to 0.473) | 7.49 (3.29 to 13.34) | 0.328 (0.152 to 0.561) | 5.85 (2.73 to 9.99) | 0.24 (0.02 to 0.6) | -1.15 (-1.42 to -0.87) |
| Kyrgyzstan | Acute myeloid leukemia | DALYs | 0.055 (0.023 to 0.106) | 1.54 (0.65 to 2.98) | 0.129 (0.058 to 0.225) | 2.21 (1 to 3.82) | 1.35 (0.48 to 2.22) | 1.73 (1.09 to 2.38) |
| Kyrgyzstan | Acute lymphoid leukemia | DALYs | 0.029 (0.012 to 0.054) | 0.81 (0.33 to 1.46) | 0.05 (0.023 to 0.088) | 0.83 (0.39 to 1.47) | 0.7 (0.27 to 1.38) | 0.3 (-0.19 to 0.78) |
| Kyrgyzstan | Chronic myeloid leukemia | DALYs | 0.025 (0.011 to 0.044) | 0.73 (0.33 to 1.31) | 0.024 (0.011 to 0.043) | 0.44 (0.21 to 0.76) | -0.02 (-0.26 to 0.32) | -1.87 (-2.31 to -1.43) |
| Kyrgyzstan | Chronic lymphoid leukemia | DALYs | 0.023 (0.01 to 0.04) | 0.7 (0.3 to 1.23) | 0.019 (0.008 to 0.033) | 0.38 (0.17 to 0.67) | -0.18 (-0.38 to 0.29) | -3.18 (-3.53 to -2.83) |
| Kyrgyzstan | Other leukemia | DALYs | 0.132 (0.056 to 0.237) | 3.72 (1.64 to 6.64) | 0.107 (0.048 to 0.186) | 2 (0.91 to 3.44) | -0.19 (-0.35 to 0.13) | -2.77 (-2.97 to -2.57) |
| Lao People's Democratic Republic | Leukemia | death | 0.002 (0 to 0.006) | 0.1 (0.02 to 0.27) | 0.01 (0.004 to 0.018) | 0.19 (0.08 to 0.36) | 2.9 (1.27 to 9.37) | 2.18 (2.13 to 2.23) |
| Lao People's Democratic Republic | Acute myeloid leukemia | death | 0 (0 to 0.001) | 0.01 (0 to 0.03) | 0.002 (0.001 to 0.003) | 0.03 (0.01 to 0.07) | 6.29 (2.96 to 20.68) | 4.45 (4.35 to 4.56) |
| Lao People's Democratic Republic | Acute lymphoid leukemia | death | 0 (0 to 0.001) | 0.01 (0 to 0.02) | 0.001 (0 to 0.002) | 0.01 (0 to 0.03) | 4.51 (1.63 to 17.59) | 3.55 (3.44 to 3.65) |
| Lao People's Democratic Republic | Chronic myeloid leukemia | death | 0 (0 to 0.001) | 0.01 (0 to 0.03) | 0.001 (0 to 0.001) | 0.01 (0.01 to 0.03) | 2.22 (0.67 to 9.05) | 1.42 (1.31 to 1.54) |
| Lao People's Democratic Republic | Chronic lymphoid leukemia | death | 0 (0 to 0) | 0 (0 to 0.01) | 0 (0 to 0.001) | 0.01 (0 to 0.01) | 4.8 (2.2 to 15.22) | 3.66 (3.55 to 3.78) |
| Lao People's Democratic Republic | Other leukemia | death | 0.002 (0 to 0.005) | 0.08 (0.01 to 0.21) | 0.006 (0.002 to 0.012) | 0.13 (0.05 to 0.25) | 2.41 (0.95 to 8.74) | 1.72 (1.69 to 1.75) |
| Lao People's Democratic Republic | Leukemia | DALYs | 0.091 (0.015 to 0.249) | 3.4 (0.6 to 9.09) | 0.348 (0.134 to 0.671) | 5.96 (2.35 to 11.47) | 2.83 (1.17 to 9.6) | 2.08 (2.03 to 2.14) |
| Lao People's Democratic Republic | Acute myeloid leukemia | DALYs | 0.008 (0.001 to 0.026) | 0.3 (0.05 to 0.95) | 0.06 (0.02 to 0.134) | 0.99 (0.34 to 2.21) | 6.42 (2.88 to 21.89) | 4.51 (4.39 to 4.63) |
| Lao People's Democratic Republic | Acute lymphoid leukemia | DALYs | 0.006 (0.001 to 0.027) | 0.2 (0.03 to 0.9) | 0.031 (0.011 to 0.075) | 0.48 (0.17 to 1.14) | 4.44 (1.44 to 18.86) | 3.49 (3.37 to 3.62) |
| Lao People's Democratic Republic | Chronic myeloid leukemia | DALYs | 0.008 (0.001 to 0.024) | 0.3 (0.05 to 0.89) | 0.025 (0.009 to 0.053) | 0.44 (0.15 to 0.92) | 2.12 (0.6 to 9.19) | 1.27 (1.18 to 1.36) |
| Lao People's Democratic Republic | Chronic lymphoid leukemia | DALYs | 0.001 (0 to 0.004) | 0.06 (0.01 to 0.18) | 0.008 (0.003 to 0.016) | 0.16 (0.06 to 0.33) | 4.64 (2.06 to 15.03) | 3.53 (3.42 to 3.64) |
| Lao People's Democratic Republic | Other leukemia | DALYs | 0.068 (0.011 to 0.197) | 2.53 (0.43 to 7.12) | 0.224 (0.082 to 0.446) | 3.88 (1.47 to 7.68) | 2.32 (0.81 to 9.16) | 1.58 (1.54 to 1.62) |
| Latvia | Leukemia | death | 0.02 (0.009 to 0.033) | 0.56 (0.26 to 0.95) | 0.021 (0.01 to 0.035) | 0.54 (0.27 to 0.91) | 0.07 (-0.15 to 0.36) | -0.15 (-0.4 to 0.1) |
| Latvia | Acute myeloid leukemia | death | 0.005 (0.002 to 0.008) | 0.14 (0.06 to 0.24) | 0.006 (0.003 to 0.01) | 0.16 (0.08 to 0.27) | 0.16 (-0.17 to 0.59) | 0.64 (0.24 to 1.05) |
| Latvia | Acute lymphoid leukemia | death | 0.001 (0.001 to 0.002) | 0.04 (0.02 to 0.07) | 0.001 (0.001 to 0.002) | 0.04 (0.02 to 0.07) | -0.12 (-0.4 to 0.28) | -0.28 (-0.61 to 0.05) |
| Latvia | Chronic myeloid leukemia | death | 0.003 (0.001 to 0.005) | 0.08 (0.04 to 0.14) | 0.002 (0.001 to 0.004) | 0.06 (0.03 to 0.1) | -0.22 (-0.43 to 0.08) | -1.42 (-1.76 to -1.09) |
| Latvia | Chronic lymphoid leukemia | death | 0.006 (0.003 to 0.01) | 0.16 (0.07 to 0.28) | 0.008 (0.004 to 0.013) | 0.18 (0.09 to 0.31) | 0.32 (-0.02 to 0.8) | 0.34 (0.07 to 0.61) |
| Latvia | Other leukemia | death | 0.005 (0.002 to 0.008) | 0.14 (0.06 to 0.24) | 0.004 (0.002 to 0.007) | 0.11 (0.06 to 0.19) | -0.12 (-0.34 to 0.2) | -1.02 (-1.34 to -0.7) |
| Latvia | Leukemia | DALYs | 0.532 (0.25 to 0.885) | 15.67 (7.31 to 26.2) | 0.47 (0.238 to 0.781) | 14.12 (7.16 to 23.18) | -0.12 (-0.29 to 0.16) | -0.52 (-0.81 to -0.22) |
| Latvia | Acute myeloid leukemia | DALYs | 0.147 (0.067 to 0.248) | 4.38 (2 to 7.37) | 0.145 (0.069 to 0.241) | 4.68 (2.26 to 7.86) | -0.02 (-0.29 to 0.34) | 0.33 (-0.1 to 0.75) |
| Latvia | Acute lymphoid leukemia | DALYs | 0.046 (0.02 to 0.082) | 1.47 (0.64 to 2.64) | 0.036 (0.017 to 0.062) | 1.36 (0.64 to 2.34) | -0.23 (-0.46 to 0.12) | -0.32 (-0.7 to 0.06) |
| Latvia | Chronic myeloid leukemia | DALYs | 0.072 (0.033 to 0.125) | 2.08 (0.97 to 3.65) | 0.047 (0.023 to 0.081) | 1.38 (0.69 to 2.36) | -0.35 (-0.51 to -0.08) | -1.77 (-2.16 to -1.37) |
| Latvia | Chronic lymphoid leukemia | DALYs | 0.13 (0.059 to 0.222) | 3.58 (1.63 to 6.11) | 0.149 (0.074 to 0.258) | 3.76 (1.89 to 6.44) | 0.15 (-0.15 to 0.57) | 0.09 (-0.21 to 0.4) |
| Latvia | Other leukemia | DALYs | 0.137 (0.063 to 0.235) | 4.15 (1.93 to 7.16) | 0.094 (0.047 to 0.161) | 2.94 (1.45 to 4.96) | -0.31 (-0.49 to -0.05) | -1.64 (-2 to -1.27) |
| Lebanon | Leukemia | death | 0.014 (0.006 to 0.025) | 0.64 (0.29 to 1.12) | 0.036 (0.018 to 0.06) | 0.68 (0.35 to 1.14) | 1.46 (0.85 to 2.64) | 0.23 (0.07 to 0.39) |
| Lebanon | Acute myeloid leukemia | death | 0.003 (0.001 to 0.006) | 0.13 (0.06 to 0.24) | 0.009 (0.004 to 0.016) | 0.17 (0.08 to 0.3) | 1.83 (1.01 to 3.22) | 0.86 (0.67 to 1.05) |
| Lebanon | Acute lymphoid leukemia | death | 0.001 (0 to 0.002) | 0.05 (0.02 to 0.09) | 0.003 (0.001 to 0.005) | 0.05 (0.03 to 0.09) | 1.43 (0.71 to 2.72) | 0.36 (0.14 to 0.59) |
| Lebanon | Chronic myeloid leukemia | death | 0.003 (0.001 to 0.006) | 0.14 (0.06 to 0.25) | 0.006 (0.003 to 0.011) | 0.11 (0.05 to 0.2) | 0.87 (0.28 to 1.85) | -0.71 (-0.8 to -0.61) |
| Lebanon | Chronic lymphoid leukemia | death | 0.001 (0 to 0.001) | 0.03 (0.01 to 0.06) | 0.003 (0.001 to 0.005) | 0.05 (0.02 to 0.09) | 2.86 (1.66 to 4.74) | 1.94 (1.7 to 2.18) |
| Lebanon | Other leukemia | death | 0.006 (0.002 to 0.012) | 0.29 (0.11 to 0.57) | 0.015 (0.007 to 0.029) | 0.29 (0.13 to 0.55) | 1.44 (0.7 to 3.65) | 0.07 (-0.09 to 0.24) |
| Lebanon | Leukemia | DALYs | 0.434 (0.188 to 0.752) | 17.43 (7.7 to 30.31) | 0.969 (0.508 to 1.613) | 18.3 (9.6 to 30.4) | 1.23 (0.66 to 2.37) | 0.21 (-0.01 to 0.44) |
| Lebanon | Acute myeloid leukemia | DALYs | 0.106 (0.045 to 0.192) | 4.13 (1.74 to 7.49) | 0.285 (0.134 to 0.483) | 5.33 (2.51 to 9.04) | 1.68 (0.85 to 2.99) | 0.91 (0.69 to 1.14) |
| Lebanon | Acute lymphoid leukemia | DALYs | 0.042 (0.016 to 0.078) | 1.57 (0.61 to 2.92) | 0.104 (0.05 to 0.174) | 1.95 (0.94 to 3.27) | 1.48 (0.71 to 2.94) | 0.76 (0.42 to 1.1) |
| Lebanon | Chronic myeloid leukemia | DALYs | 0.1 (0.044 to 0.186) | 3.96 (1.75 to 7.31) | 0.169 (0.079 to 0.302) | 3.18 (1.48 to 5.7) | 0.7 (0.17 to 1.7) | -0.81 (-0.96 to -0.66) |
| Lebanon | Chronic lymphoid leukemia | DALYs | 0.018 (0.007 to 0.033) | 0.72 (0.28 to 1.36) | 0.064 (0.028 to 0.117) | 1.22 (0.54 to 2.23) | 2.62 (1.47 to 4.39) | 2.04 (1.75 to 2.33) |
| Lebanon | Other leukemia | DALYs | 0.168 (0.064 to 0.335) | 7.05 (2.71 to 13.98) | 0.348 (0.151 to 0.682) | 6.61 (2.88 to 12.93) | 1.07 (0.44 to 3.05) | -0.11 (-0.34 to 0.11) |
| Lesotho | Leukemia | death | 0.001 (0 to 0.002) | 0.11 (0.04 to 0.22) | 0.004 (0.002 to 0.008) | 0.35 (0.15 to 0.64) | 2.98 (1.39 to 5.95) | 4.77 (4.37 to 5.17) |
| Lesotho | Acute myeloid leukemia | death | 0 (0 to 0) | 0.01 (0 to 0.01) | 0 (0 to 0.001) | 0.02 (0.01 to 0.03) | 3.16 (1.17 to 7.25) | 3.92 (3.74 to 4.1) |
| Lesotho | Acute lymphoid leukemia | death | 0 (0 to 0) | 0 (0 to 0.01) | 0 (0 to 0) | 0.01 (0 to 0.01) | 3.26 (1.29 to 7.71) | 4.18 (3.98 to 4.37) |
| Lesotho | Chronic myeloid leukemia | death | 0 (0 to 0) | 0 (0 to 0.01) | 0 (0 to 0) | 0.01 (0 to 0.02) | 4 (1.79 to 8.73) | 5.46 (5.01 to 5.91) |
| Lesotho | Chronic lymphoid leukemia | death | 0 (0 to 0.001) | 0.06 (0.02 to 0.11) | 0.002 (0.001 to 0.004) | 0.21 (0.08 to 0.39) | 3.76 (1.64 to 7.42) | 5.85 (5.32 to 6.38) |
| Lesotho | Other leukemia | death | 0 (0 to 0.001) | 0.05 (0.02 to 0.1) | 0.002 (0.001 to 0.003) | 0.11 (0.05 to 0.21) | 2.09 (0.73 to 4.77) | 3.3 (2.99 to 3.62) |
| Lesotho | Leukemia | DALYs | 0.033 (0.012 to 0.065) | 3.03 (1.1 to 5.91) | 0.14 (0.06 to 0.256) | 9.34 (4.01 to 17.06) | 3.19 (1.44 to 6.5) | 4.88 (4.46 to 5.31) |
| Lesotho | Acute myeloid leukemia | DALYs | 0.003 (0.001 to 0.006) | 0.21 (0.07 to 0.46) | 0.012 (0.005 to 0.023) | 0.64 (0.26 to 1.22) | 3.48 (1.29 to 8.23) | 4.18 (3.99 to 4.37) |
| Lesotho | Acute lymphoid leukemia | DALYs | 0.001 (0 to 0.003) | 0.09 (0.03 to 0.22) | 0.005 (0.002 to 0.011) | 0.29 (0.12 to 0.55) | 3.43 (1.29 to 8.71) | 4.29 (4.12 to 4.45) |
| Lesotho | Chronic myeloid leukemia | DALYs | 0.001 (0 to 0.003) | 0.1 (0.04 to 0.22) | 0.006 (0.003 to 0.012) | 0.38 (0.16 to 0.73) | 4.26 (1.72 to 9.81) | 5.72 (5.23 to 6.21) |
| Lesotho | Chronic lymphoid leukemia | DALYs | 0.012 (0.004 to 0.025) | 1.22 (0.41 to 2.48) | 0.062 (0.025 to 0.117) | 4.71 (1.89 to 8.94) | 4.05 (1.78 to 8.28) | 6.11 (5.53 to 6.7) |
| Lesotho | Other leukemia | DALYs | 0.016 (0.005 to 0.034) | 1.4 (0.47 to 2.92) | 0.054 (0.023 to 0.105) | 3.32 (1.45 to 6.34) | 2.38 (0.81 to 5.62) | 3.63 (3.29 to 3.97) |
| Liberia | Leukemia | death | 0.002 (0.001 to 0.003) | 0.14 (0.05 to 0.26) | 0.005 (0.002 to 0.009) | 0.2 (0.09 to 0.37) | 2.06 (1.04 to 3.9) | 2.29 (1.65 to 2.93) |
| Liberia | Acute myeloid leukemia | death | 0 (0 to 0) | 0.02 (0.01 to 0.04) | 0.001 (0 to 0.002) | 0.03 (0.01 to 0.06) | 2.71 (1.21 to 5.51) | 3.2 (2.35 to 4.06) |
| Liberia | Acute lymphoid leukemia | death | 0 (0 to 0) | 0.01 (0 to 0.01) | 0 (0 to 0.001) | 0.01 (0 to 0.02) | 3.12 (1.57 to 6.65) | 2.5 (1.75 to 3.26) |
| Liberia | Chronic myeloid leukemia | death | 0 (0 to 0.001) | 0.03 (0.01 to 0.05) | 0.001 (0 to 0.002) | 0.03 (0.01 to 0.06) | 1.93 (0.87 to 4.01) | 1.51 (0.85 to 2.17) |
| Liberia | Chronic lymphoid leukemia | death | 0 (0 to 0.001) | 0.03 (0.01 to 0.05) | 0.001 (0 to 0.001) | 0.04 (0.02 to 0.08) | 1.38 (0.57 to 3.03) | 2.34 (1.73 to 2.95) |
| Liberia | Other leukemia | death | 0.001 (0 to 0.001) | 0.06 (0.02 to 0.11) | 0.002 (0.001 to 0.004) | 0.08 (0.03 to 0.16) | 2.05 (1.02 to 4.21) | 2.26 (1.69 to 2.84) |
| Liberia | Leukemia | DALYs | 0.053 (0.021 to 0.098) | 4.25 (1.71 to 7.89) | 0.183 (0.085 to 0.332) | 6.16 (2.89 to 11.15) | 2.47 (1.29 to 4.53) | 2.32 (1.67 to 2.98) |
| Liberia | Acute myeloid leukemia | DALYs | 0.008 (0.003 to 0.017) | 0.64 (0.25 to 1.27) | 0.035 (0.013 to 0.066) | 1.06 (0.41 to 2.02) | 3.12 (1.49 to 6.15) | 3.08 (2.23 to 3.94) |
| Liberia | Acute lymphoid leukemia | DALYs | 0.004 (0.001 to 0.008) | 0.28 (0.09 to 0.59) | 0.017 (0.007 to 0.034) | 0.44 (0.17 to 0.85) | 3.66 (1.83 to 7.9) | 2.72 (1.95 to 3.48) |
| Liberia | Chronic myeloid leukemia | DALYs | 0.013 (0.005 to 0.025) | 0.99 (0.37 to 1.99) | 0.041 (0.019 to 0.074) | 1.18 (0.53 to 2.16) | 2.21 (1.02 to 4.48) | 1.56 (0.89 to 2.23) |
| Liberia | Chronic lymphoid leukemia | DALYs | 0.008 (0.003 to 0.015) | 0.68 (0.25 to 1.34) | 0.019 (0.008 to 0.037) | 0.96 (0.42 to 1.82) | 1.47 (0.58 to 3.21) | 2.24 (1.64 to 2.86) |
| Liberia | Other leukemia | DALYs | 0.02 (0.008 to 0.04) | 1.66 (0.63 to 3.25) | 0.071 (0.032 to 0.133) | 2.51 (1.09 to 4.65) | 2.52 (1.28 to 4.96) | 2.4 (1.81 to 2.99) |
| Libya | Leukemia | death | 0.011 (0.005 to 0.019) | 0.53 (0.24 to 0.93) | 0.033 (0.017 to 0.057) | 0.61 (0.31 to 1.05) | 2.09 (1.21 to 3.65) | 0.72 (0.55 to 0.89) |
| Libya | Acute myeloid leukemia | death | 0.003 (0.001 to 0.005) | 0.13 (0.06 to 0.23) | 0.01 (0.005 to 0.018) | 0.17 (0.08 to 0.3) | 2.49 (1.08 to 4.55) | 0.97 (0.79 to 1.16) |
| Libya | Acute lymphoid leukemia | death | 0.001 (0 to 0.002) | 0.04 (0.02 to 0.07) | 0.003 (0.001 to 0.006) | 0.05 (0.02 to 0.09) | 2.4 (1.35 to 4.48) | 1.08 (0.95 to 1.21) |
| Libya | Chronic myeloid leukemia | death | 0.002 (0.001 to 0.003) | 0.09 (0.04 to 0.15) | 0.004 (0.002 to 0.008) | 0.07 (0.03 to 0.13) | 1.28 (0.42 to 2.6) | -0.6 (-0.81 to -0.4) |
| Libya | Chronic lymphoid leukemia | death | 0 (0 to 0.001) | 0.02 (0.01 to 0.03) | 0.002 (0.001 to 0.003) | 0.03 (0.01 to 0.06) | 3.6 (2.09 to 6.16) | 2.16 (1.93 to 2.4) |
| Libya | Other leukemia | death | 0.005 (0.002 to 0.009) | 0.25 (0.1 to 0.49) | 0.014 (0.006 to 0.026) | 0.28 (0.13 to 0.52) | 1.99 (0.98 to 4) | 0.76 (0.57 to 0.96) |
| Libya | Leukemia | DALYs | 0.356 (0.16 to 0.625) | 15 (6.77 to 26.37) | 1.072 (0.55 to 1.902) | 17.02 (8.91 to 29.82) | 2.01 (1.11 to 3.63) | 0.63 (0.44 to 0.82) |
| Libya | Acute myeloid leukemia | DALYs | 0.102 (0.047 to 0.179) | 4.14 (1.88 to 7.22) | 0.355 (0.166 to 0.625) | 5.29 (2.48 to 9.4) | 2.48 (1.05 to 4.61) | 1.01 (0.82 to 1.21) |
| Libya | Acute lymphoid leukemia | DALYs | 0.04 (0.016 to 0.072) | 1.42 (0.59 to 2.51) | 0.129 (0.06 to 0.229) | 1.84 (0.85 to 3.29) | 2.23 (1.17 to 4.33) | 1.03 (0.87 to 1.2) |
| Libya | Chronic myeloid leukemia | DALYs | 0.065 (0.028 to 0.116) | 2.65 (1.17 to 4.71) | 0.144 (0.064 to 0.266) | 2.17 (0.94 to 3.96) | 1.22 (0.39 to 2.56) | -0.73 (-0.96 to -0.49) |
| Libya | Chronic lymphoid leukemia | DALYs | 0.01 (0.004 to 0.019) | 0.48 (0.18 to 0.88) | 0.047 (0.02 to 0.086) | 0.83 (0.35 to 1.5) | 3.58 (2.04 to 6.33) | 2.16 (1.91 to 2.41) |
| Libya | Other leukemia | DALYs | 0.139 (0.056 to 0.292) | 6.31 (2.6 to 12.87) | 0.397 (0.175 to 0.764) | 6.89 (3.09 to 13.24) | 1.85 (0.81 to 3.93) | 0.59 (0.37 to 0.8) |
| Lithuania | Leukemia | death | 0.024 (0.011 to 0.041) | 0.55 (0.24 to 0.93) | 0.031 (0.015 to 0.052) | 0.54 (0.26 to 0.91) | 0.27 (0.02 to 0.63) | 0.03 (-0.17 to 0.22) |
| Lithuania | Acute myeloid leukemia | death | 0.007 (0.003 to 0.013) | 0.15 (0.06 to 0.29) | 0.012 (0.005 to 0.022) | 0.22 (0.1 to 0.4) | 0.81 (-0.2 to 1.73) | 1.97 (1.63 to 2.31) |
| Lithuania | Acute lymphoid leukemia | death | 0.002 (0.001 to 0.004) | 0.05 (0.02 to 0.08) | 0.002 (0.001 to 0.003) | 0.03 (0.02 to 0.06) | -0.23 (-0.42 to 0.07) | -1.55 (-1.73 to -1.37) |
| Lithuania | Chronic myeloid leukemia | death | 0.004 (0.002 to 0.007) | 0.09 (0.04 to 0.15) | 0.002 (0.001 to 0.004) | 0.04 (0.02 to 0.08) | -0.39 (-0.57 to 0.09) | -2.82 (-3.41 to -2.23) |
| Lithuania | Chronic lymphoid leukemia | death | 0.004 (0.002 to 0.008) | 0.1 (0.04 to 0.17) | 0.01 (0.005 to 0.017) | 0.16 (0.07 to 0.27) | 1.23 (0.59 to 2.97) | 2.24 (1.46 to 3.04) |
| Lithuania | Other leukemia | death | 0.007 (0.003 to 0.013) | 0.17 (0.08 to 0.3) | 0.005 (0.002 to 0.009) | 0.09 (0.04 to 0.15) | -0.28 (-0.48 to 0.16) | -2.79 (-3.54 to -2.04) |
| Lithuania | Leukemia | DALYs | 0.658 (0.295 to 1.121) | 15.05 (6.77 to 25.74) | 0.658 (0.319 to 1.097) | 13.21 (6.38 to 22.12) | 0 (-0.2 to 0.28) | -0.43 (-0.66 to -0.19) |
| Lithuania | Acute myeloid leukemia | DALYs | 0.198 (0.085 to 0.38) | 4.58 (1.96 to 8.78) | 0.277 (0.128 to 0.492) | 5.92 (2.72 to 10.39) | 0.4 (-0.33 to 1.09) | 1.37 (1.02 to 1.72) |
| Lithuania | Acute lymphoid leukemia | DALYs | 0.07 (0.031 to 0.121) | 1.65 (0.74 to 2.89) | 0.044 (0.021 to 0.079) | 1.09 (0.52 to 1.96) | -0.36 (-0.53 to -0.11) | -1.66 (-1.87 to -1.46) |
| Lithuania | Chronic myeloid leukemia | DALYs | 0.109 (0.049 to 0.194) | 2.51 (1.13 to 4.48) | 0.053 (0.024 to 0.094) | 1.13 (0.53 to 2.03) | -0.52 (-0.66 to -0.12) | -3.15 (-3.78 to -2.52) |
| Lithuania | Chronic lymphoid leukemia | DALYs | 0.103 (0.046 to 0.184) | 2.29 (1.01 to 4.04) | 0.184 (0.089 to 0.324) | 3.23 (1.59 to 5.7) | 0.78 (0.27 to 2.25) | 1.72 (0.93 to 2.52) |
| Lithuania | Other leukemia | DALYs | 0.178 (0.082 to 0.31) | 4.01 (1.83 to 7.02) | 0.099 (0.047 to 0.174) | 1.84 (0.87 to 3.23) | -0.44 (-0.6 to -0.07) | -3.26 (-3.98 to -2.53) |
| Luxembourg | Leukemia | death | 0.003 (0.001 to 0.005) | 0.51 (0.24 to 0.88) | 0.004 (0.002 to 0.007) | 0.41 (0.2 to 0.7) | 0.55 (0.32 to 0.88) | -0.73 (-0.84 to -0.62) |
| Luxembourg | Acute myeloid leukemia | death | 0.001 (0 to 0.001) | 0.12 (0.05 to 0.21) | 0.001 (0.001 to 0.003) | 0.15 (0.07 to 0.26) | 1.35 (0.72 to 1.96) | 0.96 (0.77 to 1.15) |
| Luxembourg | Acute lymphoid leukemia | death | 0 (0 to 0) | 0.02 (0.01 to 0.03) | 0 (0 to 0) | 0.01 (0.01 to 0.02) | 0.34 (0.07 to 0.88) | -1.29 (-1.39 to -1.2) |
| Luxembourg | Chronic myeloid leukemia | death | 0 (0 to 0) | 0.04 (0.02 to 0.06) | 0 (0 to 0) | 0.01 (0.01 to 0.02) | -0.34 (-0.45 to -0.16) | -4.49 (-4.8 to -4.18) |
| Luxembourg | Chronic lymphoid leukemia | death | 0 (0 to 0.001) | 0.07 (0.03 to 0.12) | 0.001 (0 to 0.001) | 0.06 (0.03 to 0.1) | 0.62 (0.3 to 1.07) | -0.86 (-1.14 to -0.59) |
| Luxembourg | Other leukemia | death | 0.001 (0.001 to 0.002) | 0.27 (0.13 to 0.46) | 0.002 (0.001 to 0.003) | 0.18 (0.09 to 0.3) | 0.32 (0.1 to 0.62) | -1.3 (-1.39 to -1.21) |
| Luxembourg | Leukemia | DALYs | 0.064 (0.03 to 0.111) | 12.32 (5.72 to 21.46) | 0.088 (0.042 to 0.147) | 9.58 (4.56 to 15.97) | 0.38 (0.18 to 0.66) | -0.99 (-1.08 to -0.9) |
| Luxembourg | Acute myeloid leukemia | DALYs | 0.016 (0.007 to 0.028) | 3.12 (1.44 to 5.51) | 0.034 (0.016 to 0.057) | 3.77 (1.79 to 6.38) | 1.12 (0.59 to 1.63) | 0.63 (0.48 to 0.79) |
| Luxembourg | Acute lymphoid leukemia | DALYs | 0.003 (0.001 to 0.005) | 0.63 (0.28 to 1.14) | 0.005 (0.002 to 0.008) | 0.58 (0.27 to 1.02) | 0.52 (0.18 to 1.21) | -0.69 (-0.83 to -0.55) |
| Luxembourg | Chronic myeloid leukemia | DALYs | 0.005 (0.002 to 0.009) | 0.97 (0.46 to 1.67) | 0.003 (0.002 to 0.005) | 0.35 (0.17 to 0.59) | -0.34 (-0.46 to -0.17) | -4.32 (-4.62 to -4.02) |
| Luxembourg | Chronic lymphoid leukemia | DALYs | 0.008 (0.004 to 0.013) | 1.38 (0.65 to 2.41) | 0.011 (0.005 to 0.019) | 1.1 (0.51 to 1.9) | 0.46 (0.18 to 0.87) | -1 (-1.3 to -0.7) |
| Luxembourg | Other leukemia | DALYs | 0.032 (0.015 to 0.056) | 6.22 (2.94 to 10.84) | 0.035 (0.017 to 0.059) | 3.78 (1.84 to 6.33) | 0.1 (-0.08 to 0.34) | -1.76 (-1.82 to -1.7) |
| Madagascar | Leukemia | death | 0.002 (0.001 to 0.005) | 0.04 (0.01 to 0.09) | 0.009 (0.004 to 0.018) | 0.08 (0.03 to 0.15) | 2.94 (1.64 to 5.58) | 2.65 (2.38 to 2.93) |
| Madagascar | Acute myeloid leukemia | death | 0 (0 to 0.001) | 0 (0 to 0.01) | 0.001 (0 to 0.002) | 0.01 (0 to 0.02) | 3.96 (2.05 to 8.26) | 3.25 (2.87 to 3.63) |
| Madagascar | Acute lymphoid leukemia | death | 0 (0 to 0) | 0 (0 to 0) | 0.001 (0 to 0.001) | 0 (0 to 0.01) | 3.61 (1.93 to 7.84) | 2.96 (2.57 to 3.34) |
| Madagascar | Chronic myeloid leukemia | death | 0.001 (0 to 0.001) | 0.01 (0 to 0.02) | 0.002 (0.001 to 0.004) | 0.01 (0 to 0.02) | 2.34 (1.1 to 5.32) | 1.85 (1.44 to 2.26) |
| Madagascar | Chronic lymphoid leukemia | death | 0 (0 to 0.001) | 0.01 (0 to 0.03) | 0.003 (0.001 to 0.006) | 0.03 (0.01 to 0.06) | 4.6 (2.53 to 9.55) | 4.01 (3.78 to 4.23) |
| Madagascar | Other leukemia | death | 0.001 (0 to 0.002) | 0.02 (0 to 0.04) | 0.003 (0.001 to 0.006) | 0.02 (0.01 to 0.05) | 2.11 (1.05 to 4.39) | 1.63 (1.42 to 1.85) |
| Madagascar | Leukemia | DALYs | 0.087 (0.026 to 0.192) | 1.26 (0.4 to 2.76) | 0.333 (0.13 to 0.648) | 2.18 (0.89 to 4.19) | 2.83 (1.56 to 5.54) | 2.44 (2.11 to 2.77) |
| Madagascar | Acute myeloid leukemia | DALYs | 0.009 (0.003 to 0.024) | 0.13 (0.04 to 0.32) | 0.047 (0.018 to 0.095) | 0.27 (0.1 to 0.54) | 4 (2.1 to 8.49) | 3.18 (2.78 to 3.58) |
| Madagascar | Acute lymphoid leukemia | DALYs | 0.006 (0.002 to 0.014) | 0.07 (0.02 to 0.17) | 0.027 (0.01 to 0.056) | 0.13 (0.05 to 0.27) | 3.55 (1.82 to 8.03) | 2.93 (2.48 to 3.38) |
| Madagascar | Chronic myeloid leukemia | DALYs | 0.024 (0.007 to 0.058) | 0.32 (0.09 to 0.76) | 0.077 (0.028 to 0.157) | 0.44 (0.16 to 0.9) | 2.22 (0.99 to 5.18) | 1.65 (1.19 to 2.12) |
| Madagascar | Chronic lymphoid leukemia | DALYs | 0.012 (0.004 to 0.027) | 0.23 (0.07 to 0.53) | 0.071 (0.025 to 0.147) | 0.64 (0.22 to 1.32) | 5.1 (2.78 to 10.32) | 4.18 (3.94 to 4.42) |
| Madagascar | Other leukemia | DALYs | 0.036 (0.011 to 0.082) | 0.51 (0.16 to 1.15) | 0.112 (0.04 to 0.229) | 0.7 (0.26 to 1.42) | 2.08 (1.03 to 4.4) | 1.49 (1.24 to 1.75) |
| Malawi | Leukemia | death | 0.002 (0 to 0.005) | 0.05 (0.01 to 0.11) | 0.008 (0.003 to 0.016) | 0.1 (0.04 to 0.19) | 2.88 (1.48 to 7.6) | 3.22 (2.94 to 3.5) |
| Malawi | Acute myeloid leukemia | death | 0 (0 to 0.001) | 0.01 (0 to 0.02) | 0.002 (0.001 to 0.004) | 0.02 (0.01 to 0.04) | 3.4 (1.6 to 8.24) | 3.37 (3.16 to 3.59) |
| Malawi | Acute lymphoid leukemia | death | 0 (0 to 0) | 0 (0 to 0.01) | 0.001 (0 to 0.001) | 0.01 (0 to 0.01) | 3.03 (1.42 to 7.83) | 2.81 (2.58 to 3.04) |
| Malawi | Chronic myeloid leukemia | death | 0 (0 to 0.001) | 0 (0 to 0.01) | 0.001 (0 to 0.002) | 0.01 (0 to 0.02) | 1.92 (0.67 to 5.78) | 1.71 (1.44 to 1.98) |
| Malawi | Chronic lymphoid leukemia | death | 0.001 (0 to 0.002) | 0.02 (0 to 0.04) | 0.003 (0.001 to 0.006) | 0.05 (0.02 to 0.1) | 4.12 (2.1 to 11.36) | 4.47 (4.08 to 4.86) |
| Malawi | Other leukemia | death | 0.001 (0 to 0.001) | 0.01 (0 to 0.03) | 0.002 (0.001 to 0.003) | 0.02 (0.01 to 0.04) | 1.62 (0.56 to 4.99) | 1.51 (1.22 to 1.8) |
| Malawi | Leukemia | DALYs | 0.077 (0.019 to 0.185) | 1.42 (0.34 to 3.42) | 0.277 (0.109 to 0.557) | 2.8 (1.11 to 5.55) | 2.59 (1.25 to 6.78) | 2.73 (2.48 to 2.97) |
| Malawi | Acute myeloid leukemia | DALYs | 0.018 (0.004 to 0.043) | 0.3 (0.07 to 0.72) | 0.077 (0.029 to 0.154) | 0.66 (0.26 to 1.28) | 3.29 (1.5 to 8.02) | 3.1 (2.9 to 3.29) |
| Malawi | Acute lymphoid leukemia | DALYs | 0.007 (0.002 to 0.017) | 0.11 (0.03 to 0.26) | 0.03 (0.01 to 0.062) | 0.22 (0.08 to 0.46) | 3.05 (1.31 to 7.89) | 2.68 (2.45 to 2.9) |
| Malawi | Chronic myeloid leukemia | DALYs | 0.01 (0.003 to 0.025) | 0.17 (0.04 to 0.42) | 0.029 (0.011 to 0.062) | 0.25 (0.1 to 0.54) | 1.79 (0.55 to 5.36) | 1.34 (1.08 to 1.6) |
| Malawi | Chronic lymphoid leukemia | DALYs | 0.017 (0.003 to 0.042) | 0.41 (0.08 to 1.04) | 0.08 (0.03 to 0.159) | 1.08 (0.41 to 2.16) | 3.81 (1.92 to 10.75) | 4.23 (3.87 to 4.59) |
| Malawi | Other leukemia | DALYs | 0.025 (0.006 to 0.062) | 0.43 (0.1 to 1.05) | 0.062 (0.023 to 0.132) | 0.57 (0.22 to 1.19) | 1.46 (0.38 to 4.67) | 1.1 (0.83 to 1.37) |
| Malaysia | Leukemia | death | 0.021 (0.008 to 0.039) | 0.19 (0.07 to 0.36) | 0.088 (0.043 to 0.153) | 0.32 (0.15 to 0.56) | 3.28 (1.95 to 5.66) | 1.78 (1.55 to 2.01) |
| Malaysia | Acute myeloid leukemia | death | 0.007 (0.003 to 0.014) | 0.06 (0.02 to 0.12) | 0.036 (0.018 to 0.065) | 0.13 (0.06 to 0.22) | 4.24 (2.5 to 7.6) | 2.53 (2.31 to 2.75) |
| Malaysia | Acute lymphoid leukemia | death | 0.003 (0.001 to 0.006) | 0.02 (0.01 to 0.05) | 0.013 (0.006 to 0.023) | 0.04 (0.02 to 0.07) | 3.08 (1.7 to 5.8) | 1.8 (1.67 to 1.93) |
| Malaysia | Chronic myeloid leukemia | death | 0.003 (0.001 to 0.006) | 0.03 (0.01 to 0.05) | 0.01 (0.005 to 0.018) | 0.04 (0.02 to 0.06) | 2.45 (1.21 to 4.71) | 0.93 (0.72 to 1.14) |
| Malaysia | Chronic lymphoid leukemia | death | 0.002 (0.001 to 0.004) | 0.02 (0.01 to 0.05) | 0.013 (0.005 to 0.025) | 0.05 (0.02 to 0.11) | 5.78 (3.41 to 10.56) | 3.08 (2.64 to 3.53) |
| Malaysia | Other leukemia | death | 0.006 (0.002 to 0.011) | 0.05 (0.02 to 0.1) | 0.016 (0.007 to 0.032) | 0.06 (0.03 to 0.12) | 1.84 (0.76 to 3.98) | 0.2 (-0.06 to 0.46) |
| Malaysia | Leukemia | DALYs | 0.79 (0.323 to 1.492) | 6.11 (2.46 to 11.41) | 2.997 (1.461 to 5.184) | 9.61 (4.67 to 16.59) | 2.79 (1.6 to 4.98) | 1.5 (1.33 to 1.68) |
| Malaysia | Acute myeloid leukemia | DALYs | 0.267 (0.107 to 0.519) | 2.07 (0.83 to 4.02) | 1.267 (0.611 to 2.271) | 4.02 (1.94 to 7.2) | 3.75 (2.15 to 6.78) | 2.36 (2.18 to 2.54) |
| Malaysia | Acute lymphoid leukemia | DALYs | 0.146 (0.053 to 0.298) | 0.97 (0.36 to 1.98) | 0.562 (0.264 to 0.987) | 1.67 (0.79 to 2.96) | 2.85 (1.52 to 5.76) | 1.73 (1.62 to 1.84) |
| Malaysia | Chronic myeloid leukemia | DALYs | 0.111 (0.044 to 0.213) | 0.87 (0.35 to 1.66) | 0.346 (0.163 to 0.609) | 1.11 (0.52 to 1.95) | 2.11 (1 to 4.18) | 0.75 (0.57 to 0.92) |
| Malaysia | Chronic lymphoid leukemia | DALYs | 0.047 (0.016 to 0.097) | 0.5 (0.17 to 1.06) | 0.286 (0.124 to 0.557) | 1.08 (0.46 to 2.11) | 5.07 (2.94 to 9.14) | 2.7 (2.35 to 3.05) |
| Malaysia | Other leukemia | DALYs | 0.22 (0.081 to 0.441) | 1.71 (0.64 to 3.35) | 0.537 (0.244 to 1.042) | 1.74 (0.79 to 3.43) | 1.45 (0.48 to 3.45) | -0.18 (-0.4 to 0.04) |
| Maldives | Leukemia | death | 0 (0 to 0) | 0.08 (0.02 to 0.23) | 0 (0 to 0.001) | 0.12 (0.05 to 0.23) | 3.92 (1.72 to 12.71) | 1.05 (0.91 to 1.2) |
| Maldives | Acute myeloid leukemia | death | 0 (0 to 0) | 0.02 (0 to 0.05) | 0 (0 to 0) | 0.05 (0.02 to 0.09) | 9.19 (4.58 to 27.4) | 3.46 (3.37 to 3.55) |
| Maldives | Acute lymphoid leukemia | death | 0 (0 to 0) | 0 (0 to 0.01) | 0 (0 to 0) | 0.01 (0 to 0.01) | 5.67 (2.25 to 21.82) | 1.96 (1.85 to 2.06) |
| Maldives | Chronic myeloid leukemia | death | 0 (0 to 0) | 0.02 (0 to 0.05) | 0 (0 to 0) | 0.02 (0.01 to 0.03) | 1.9 (0.43 to 9.35) | -0.64 (-0.85 to -0.44) |
| Maldives | Chronic lymphoid leukemia | death | 0 (0 to 0) | 0 (0 to 0.01) | 0 (0 to 0) | 0.01 (0 to 0.02) | 7.2 (3.59 to 21.73) | 2.99 (2.81 to 3.16) |
| Maldives | Other leukemia | death | 0 (0 to 0) | 0.04 (0.01 to 0.13) | 0 (0 to 0) | 0.05 (0.02 to 0.09) | 2.33 (0.72 to 10.32) | -0.1 (-0.22 to 0.02) |
| Maldives | Leukemia | DALYs | 0.003 (0.001 to 0.009) | 2.63 (0.48 to 7.06) | 0.015 (0.007 to 0.029) | 3.41 (1.46 to 6.51) | 3.63 (1.43 to 12.55) | 0.74 (0.6 to 0.88) |
| Maldives | Acute myeloid leukemia | DALYs | 0.001 (0 to 0.002) | 0.53 (0.1 to 1.41) | 0.007 (0.003 to 0.014) | 1.41 (0.6 to 2.77) | 9.46 (4.6 to 28.44) | 3.51 (3.41 to 3.61) |
| Maldives | Acute lymphoid leukemia | DALYs | 0 (0 to 0.001) | 0.13 (0.02 to 0.4) | 0.001 (0.001 to 0.003) | 0.23 (0.1 to 0.46) | 5.71 (2.08 to 22.89) | 2.03 (1.88 to 2.19) |
| Maldives | Chronic myeloid leukemia | DALYs | 0.001 (0 to 0.002) | 0.61 (0.1 to 1.81) | 0.002 (0.001 to 0.004) | 0.46 (0.17 to 0.94) | 1.6 (0.24 to 8.79) | -1.21 (-1.37 to -1.06) |
| Maldives | Chronic lymphoid leukemia | DALYs | 0 (0 to 0) | 0.08 (0.01 to 0.21) | 0.001 (0 to 0.001) | 0.18 (0.07 to 0.36) | 6.49 (3.15 to 20.99) | 2.76 (2.58 to 2.93) |
| Maldives | Other leukemia | DALYs | 0.002 (0 to 0.005) | 1.29 (0.21 to 3.74) | 0.004 (0.002 to 0.009) | 1.13 (0.43 to 2.23) | 1.78 (0.36 to 9.34) | -0.73 (-0.83 to -0.62) |
| Mali | Leukemia | death | 0.002 (0.001 to 0.005) | 0.05 (0.01 to 0.12) | 0.01 (0.004 to 0.02) | 0.1 (0.04 to 0.2) | 3.25 (1.91 to 7.37) | 2.44 (2.33 to 2.54) |
| Mali | Acute myeloid leukemia | death | 0 (0 to 0) | 0 (0 to 0.01) | 0.001 (0 to 0.002) | 0.01 (0 to 0.02) | 4.68 (2.35 to 11.06) | 3.4 (3.31 to 3.49) |
| Mali | Acute lymphoid leukemia | death | 0 (0 to 0) | 0 (0 to 0.01) | 0.001 (0 to 0.001) | 0 (0 to 0.01) | 4.48 (2.19 to 11.43) | 3.18 (3.08 to 3.28) |
| Mali | Chronic myeloid leukemia | death | 0 (0 to 0.001) | 0.01 (0 to 0.02) | 0.002 (0.001 to 0.004) | 0.02 (0.01 to 0.04) | 3.19 (1.45 to 8.07) | 2.32 (2.23 to 2.41) |
| Mali | Chronic lymphoid leukemia | death | 0.001 (0 to 0.002) | 0.02 (0 to 0.04) | 0.003 (0.001 to 0.005) | 0.03 (0.01 to 0.06) | 3.07 (1.64 to 7.36) | 2.43 (2.29 to 2.57) |
| Mali | Other leukemia | death | 0.001 (0 to 0.002) | 0.02 (0.01 to 0.05) | 0.004 (0.002 to 0.007) | 0.04 (0.02 to 0.08) | 2.95 (1.59 to 6.8) | 2.19 (2.09 to 2.29) |
| Mali | Leukemia | DALYs | 0.076 (0.021 to 0.177) | 1.55 (0.43 to 3.54) | 0.338 (0.141 to 0.681) | 3.09 (1.28 to 6.11) | 3.43 (1.9 to 7.98) | 2.5 (2.41 to 2.6) |
| Mali | Acute myeloid leukemia | DALYs | 0.008 (0.002 to 0.018) | 0.14 (0.04 to 0.34) | 0.046 (0.017 to 0.095) | 0.37 (0.14 to 0.75) | 4.98 (2.56 to 11.79) | 3.49 (3.4 to 3.58) |
| Mali | Acute lymphoid leukemia | DALYs | 0.005 (0.001 to 0.012) | 0.08 (0.02 to 0.2) | 0.027 (0.01 to 0.057) | 0.19 (0.07 to 0.39) | 4.75 (2.25 to 12.35) | 3.26 (3.15 to 3.37) |
| Mali | Chronic myeloid leukemia | DALYs | 0.017 (0.004 to 0.04) | 0.32 (0.08 to 0.75) | 0.072 (0.026 to 0.155) | 0.59 (0.22 to 1.26) | 3.33 (1.42 to 8.79) | 2.35 (2.27 to 2.44) |
| Mali | Chronic lymphoid leukemia | DALYs | 0.017 (0.005 to 0.039) | 0.39 (0.11 to 0.88) | 0.071 (0.028 to 0.145) | 0.79 (0.31 to 1.59) | 3.1 (1.61 to 7.86) | 2.44 (2.31 to 2.57) |
| Mali | Other leukemia | DALYs | 0.03 (0.008 to 0.07) | 0.62 (0.17 to 1.45) | 0.123 (0.049 to 0.249) | 1.15 (0.47 to 2.28) | 3.09 (1.56 to 7.38) | 2.25 (2.16 to 2.34) |
| Malta | Leukemia | death | 0.001 (0 to 0.002) | 0.28 (0.11 to 0.5) | 0.002 (0.001 to 0.004) | 0.26 (0.12 to 0.46) | 1.06 (0.68 to 1.68) | -0.42 (-0.71 to -0.13) |
| Malta | Acute myeloid leukemia | death | 0 (0 to 0.001) | 0.11 (0.05 to 0.21) | 0.001 (0.001 to 0.002) | 0.15 (0.07 to 0.27) | 1.77 (1.2 to 2.62) | 0.92 (0.57 to 1.27) |
| Malta | Acute lymphoid leukemia | death | 0 (0 to 0) | 0.03 (0.01 to 0.05) | 0 (0 to 0) | 0.02 (0.01 to 0.04) | 0.45 (0.11 to 1.16) | -1.26 (-1.58 to -0.95) |
| Malta | Chronic myeloid leukemia | death | 0 (0 to 0) | 0.05 (0.02 to 0.09) | 0 (0 to 0) | 0.02 (0.01 to 0.04) | -0.13 (-0.35 to 0.19) | -4.33 (-4.81 to -3.85) |
| Malta | Chronic lymphoid leukemia | death | 0 (0 to 0) | 0.04 (0.02 to 0.08) | 0 (0 to 0.001) | 0.04 (0.02 to 0.07) | 1.32 (0.71 to 2.19) | -0.5 (-0.89 to -0.11) |
| Malta | Other leukemia | death | 0 (0 to 0) | 0.04 (0.02 to 0.08) | 0 (0 to 0.001) | 0.03 (0.01 to 0.06) | 0.69 (0.27 to 1.36) | -1.15 (-1.34 to -0.96) |
| Malta | Leukemia | DALYs | 0.028 (0.011 to 0.05) | 6.49 (2.69 to 11.76) | 0.051 (0.023 to 0.089) | 6.58 (3.03 to 11.55) | 0.84 (0.52 to 1.38) | -0.21 (-0.5 to 0.09) |
| Malta | Acute myeloid leukemia | DALYs | 0.012 (0.005 to 0.022) | 2.79 (1.16 to 5.07) | 0.029 (0.013 to 0.052) | 3.8 (1.74 to 6.69) | 1.43 (0.94 to 2.18) | 1.02 (0.68 to 1.38) |
| Malta | Acute lymphoid leukemia | DALYs | 0.004 (0.001 to 0.007) | 0.9 (0.35 to 1.69) | 0.006 (0.003 to 0.01) | 0.93 (0.42 to 1.65) | 0.54 (0.18 to 1.29) | -0.29 (-0.62 to 0.04) |
| Malta | Chronic myeloid leukemia | DALYs | 0.005 (0.002 to 0.009) | 1.12 (0.47 to 2.09) | 0.004 (0.002 to 0.007) | 0.49 (0.22 to 0.87) | -0.19 (-0.39 to 0.1) | -3.97 (-4.43 to -3.51) |
| Malta | Chronic lymphoid leukemia | DALYs | 0.003 (0.001 to 0.006) | 0.76 (0.32 to 1.41) | 0.007 (0.003 to 0.012) | 0.7 (0.31 to 1.28) | 1.05 (0.48 to 1.8) | -0.6 (-1 to -0.2) |
| Malta | Other leukemia | DALYs | 0.004 (0.002 to 0.007) | 0.91 (0.38 to 1.69) | 0.005 (0.002 to 0.01) | 0.64 (0.29 to 1.16) | 0.39 (0.08 to 0.92) | -1.23 (-1.42 to -1.04) |
| Marshall Islands | Leukemia | death | 0 (0 to 0) | 0.28 (0.09 to 0.57) | 0 (0 to 0) | 0.35 (0.13 to 0.67) | 1.63 (0.86 to 3.44) | 0.45 (0.23 to 0.67) |
| Marshall Islands | Acute myeloid leukemia | death | 0 (0 to 0) | 0.05 (0.02 to 0.12) | 0 (0 to 0) | 0.08 (0.03 to 0.15) | 1.92 (0.96 to 4.01) | 0.89 (0.7 to 1.09) |
| Marshall Islands | Acute lymphoid leukemia | death | 0 (0 to 0) | 0.02 (0.01 to 0.04) | 0 (0 to 0) | 0.02 (0.01 to 0.05) | 1.66 (0.72 to 3.79) | 0.7 (0.47 to 0.94) |
| Marshall Islands | Chronic myeloid leukemia | death | 0 (0 to 0) | 0.04 (0.01 to 0.09) | 0 (0 to 0) | 0.05 (0.02 to 0.1) | 1.44 (0.59 to 3.26) | -0.25 (-0.62 to 0.12) |
| Marshall Islands | Chronic lymphoid leukemia | death | 0 (0 to 0) | 0 (0 to 0.01) | 0 (0 to 0) | 0.01 (0 to 0.01) | 1.89 (0.87 to 4.08) | 0.51 (0.37 to 0.65) |
| Marshall Islands | Other leukemia | death | 0 (0 to 0) | 0.16 (0.05 to 0.35) | 0 (0 to 0) | 0.2 (0.07 to 0.39) | 1.55 (0.71 to 3.39) | 0.45 (0.25 to 0.65) |
| Marshall Islands | Leukemia | DALYs | 0.002 (0.001 to 0.004) | 9.01 (2.93 to 17.95) | 0.006 (0.002 to 0.011) | 11.85 (4.82 to 22.71) | 1.65 (0.86 to 3.57) | 0.6 (0.34 to 0.85) |
| Marshall Islands | Acute myeloid leukemia | DALYs | 0.001 (0 to 0.001) | 1.97 (0.6 to 4.33) | 0.002 (0.001 to 0.003) | 2.91 (1.16 to 5.83) | 1.88 (0.89 to 4.14) | 0.98 (0.76 to 1.19) |
| Marshall Islands | Acute lymphoid leukemia | DALYs | 0 (0 to 0) | 0.72 (0.22 to 1.51) | 0.001 (0 to 0.001) | 0.99 (0.36 to 2.03) | 1.53 (0.6 to 3.76) | 0.89 (0.7 to 1.08) |
| Marshall Islands | Chronic myeloid leukemia | DALYs | 0 (0 to 0.001) | 1.41 (0.43 to 3.12) | 0.001 (0 to 0.002) | 1.63 (0.6 to 3.49) | 1.45 (0.57 to 3.39) | -0.08 (-0.49 to 0.33) |
| Marshall Islands | Chronic lymphoid leukemia | DALYs | 0 (0 to 0) | 0.12 (0.03 to 0.25) | 0 (0 to 0) | 0.16 (0.06 to 0.34) | 1.97 (0.96 to 4.2) | 0.8 (0.66 to 0.94) |
| Marshall Islands | Other leukemia | DALYs | 0.001 (0 to 0.002) | 4.8 (1.45 to 9.99) | 0.003 (0.001 to 0.006) | 6.15 (2.31 to 12.08) | 1.61 (0.7 to 3.5) | 0.58 (0.34 to 0.82) |
| Mauritania | Leukemia | death | 0.002 (0.001 to 0.004) | 0.18 (0.07 to 0.34) | 0.005 (0.003 to 0.009) | 0.24 (0.12 to 0.42) | 1.68 (0.77 to 3.33) | 1.1 (1.01 to 1.18) |
| Mauritania | Acute myeloid leukemia | death | 0 (0 to 0.001) | 0.03 (0.01 to 0.06) | 0.001 (0 to 0.002) | 0.04 (0.02 to 0.08) | 2.12 (0.91 to 4.08) | 1.84 (1.72 to 1.96) |
| Mauritania | Acute lymphoid leukemia | death | 0 (0 to 0) | 0.01 (0 to 0.02) | 0 (0 to 0.001) | 0.01 (0 to 0.02) | 1.97 (0.56 to 4.73) | 1.21 (1.13 to 1.28) |
| Mauritania | Chronic myeloid leukemia | death | 0 (0 to 0.001) | 0.03 (0.01 to 0.07) | 0.001 (0 to 0.002) | 0.04 (0.02 to 0.07) | 1.26 (0.43 to 3.17) | 0.53 (0.42 to 0.63) |
| Mauritania | Chronic lymphoid leukemia | death | 0 (0 to 0.001) | 0.04 (0.01 to 0.08) | 0.001 (0 to 0.002) | 0.05 (0.03 to 0.1) | 1.75 (0.84 to 3.46) | 1.03 (0.97 to 1.1) |
| Mauritania | Other leukemia | death | 0.001 (0 to 0.001) | 0.07 (0.03 to 0.14) | 0.002 (0.001 to 0.004) | 0.09 (0.04 to 0.17) | 1.64 (0.64 to 3.49) | 1.06 (0.95 to 1.16) |
| Mauritania | Leukemia | DALYs | 0.062 (0.025 to 0.116) | 5.26 (2.18 to 9.81) | 0.162 (0.079 to 0.293) | 6.48 (3.18 to 11.43) | 1.62 (0.64 to 3.32) | 0.9 (0.81 to 0.99) |
| Mauritania | Acute myeloid leukemia | DALYs | 0.011 (0.004 to 0.023) | 0.86 (0.32 to 1.78) | 0.035 (0.014 to 0.069) | 1.31 (0.53 to 2.61) | 2.15 (0.89 to 4.32) | 1.69 (1.58 to 1.8) |
| Mauritania | Acute lymphoid leukemia | DALYs | 0.004 (0.001 to 0.009) | 0.3 (0.1 to 0.65) | 0.013 (0.005 to 0.026) | 0.42 (0.17 to 0.83) | 2.09 (0.52 to 5.47) | 1.29 (1.22 to 1.37) |
| Mauritania | Chronic myeloid leukemia | DALYs | 0.015 (0.006 to 0.03) | 1.21 (0.47 to 2.41) | 0.032 (0.013 to 0.064) | 1.19 (0.5 to 2.33) | 1.13 (0.26 to 3.2) | 0.2 (0.08 to 0.32) |
| Mauritania | Chronic lymphoid leukemia | DALYs | 0.009 (0.003 to 0.019) | 0.98 (0.35 to 1.92) | 0.025 (0.011 to 0.048) | 1.22 (0.55 to 2.3) | 1.69 (0.78 to 3.46) | 0.84 (0.77 to 0.91) |
| Mauritania | Other leukemia | DALYs | 0.022 (0.009 to 0.043) | 1.91 (0.78 to 3.78) | 0.056 (0.025 to 0.105) | 2.34 (1.06 to 4.43) | 1.57 (0.49 to 3.64) | 0.85 (0.75 to 0.96) |
| Mauritius | Leukemia | death | 0.001 (0 to 0.002) | 0.13 (0.06 to 0.24) | 0.003 (0.002 to 0.006) | 0.21 (0.1 to 0.37) | 2.23 (1.48 to 3.4) | 1.54 (1.43 to 1.65) |
| Mauritius | Acute myeloid leukemia | death | 0 (0 to 0) | 0.02 (0.01 to 0.03) | 0.001 (0 to 0.001) | 0.04 (0.02 to 0.08) | 4.3 (2.72 to 6.73) | 3.64 (3.13 to 4.16) |
| Mauritius | Acute lymphoid leukemia | death | 0 (0 to 0) | 0.01 (0 to 0.01) | 0 (0 to 0) | 0.01 (0.01 to 0.02) | 2.77 (1.72 to 4.62) | 3.39 (2.82 to 3.97) |
| Mauritius | Chronic myeloid leukemia | death | 0 (0 to 0) | 0.01 (0 to 0.01) | 0 (0 to 0.001) | 0.02 (0.01 to 0.03) | 4.54 (2.97 to 6.86) | 3.71 (3.07 to 4.34) |
| Mauritius | Chronic lymphoid leukemia | death | 0 (0 to 0) | 0 (0 to 0.01) | 0 (0 to 0) | 0 (0 to 0.01) | 3.14 (1.91 to 5.08) | 1.47 (1.12 to 1.81) |
| Mauritius | Other leukemia | death | 0.001 (0 to 0.001) | 0.1 (0.04 to 0.18) | 0.002 (0.001 to 0.004) | 0.13 (0.06 to 0.23) | 1.68 (1.03 to 2.65) | 0.74 (0.52 to 0.95) |
| Mauritius | Leukemia | DALYs | 0.04 (0.017 to 0.073) | 4.2 (1.8 to 7.63) | 0.109 (0.052 to 0.193) | 6.67 (3.16 to 11.84) | 1.74 (1.06 to 2.84) | 1.49 (1.38 to 1.6) |
| Mauritius | Acute myeloid leukemia | DALYs | 0.005 (0.002 to 0.01) | 0.54 (0.23 to 1) | 0.025 (0.011 to 0.044) | 1.55 (0.73 to 2.76) | 3.62 (2.26 to 5.97) | 3.66 (3.14 to 4.19) |
| Mauritius | Acute lymphoid leukemia | DALYs | 0.002 (0.001 to 0.004) | 0.22 (0.09 to 0.4) | 0.008 (0.004 to 0.014) | 0.54 (0.26 to 0.94) | 2.38 (1.35 to 4.27) | 3.67 (3.11 to 4.24) |
| Mauritius | Chronic myeloid leukemia | DALYs | 0.002 (0.001 to 0.003) | 0.21 (0.09 to 0.38) | 0.008 (0.004 to 0.015) | 0.47 (0.22 to 0.84) | 3.42 (2.2 to 5.38) | 3.12 (2.54 to 3.71) |
| Mauritius | Chronic lymphoid leukemia | DALYs | 0.001 (0 to 0.001) | 0.07 (0.03 to 0.13) | 0.002 (0.001 to 0.004) | 0.13 (0.06 to 0.22) | 3.07 (1.88 to 4.92) | 1.81 (1.51 to 2.1) |
| Mauritius | Other leukemia | DALYs | 0.03 (0.013 to 0.054) | 3.17 (1.36 to 5.72) | 0.066 (0.03 to 0.116) | 3.99 (1.84 to 7.08) | 1.22 (0.66 to 2.1) | 0.58 (0.38 to 0.79) |
| Mexico | Leukemia | death | 0.143 (0.063 to 0.242) | 0.28 (0.12 to 0.47) | 0.436 (0.212 to 0.722) | 0.36 (0.17 to 0.59) | 2.05 (1.51 to 2.77) | 0.89 (0.81 to 0.97) |
| Mexico | Acute myeloid leukemia | death | 0.035 (0.015 to 0.059) | 0.07 (0.03 to 0.11) | 0.137 (0.066 to 0.229) | 0.11 (0.05 to 0.19) | 2.95 (2.19 to 3.97) | 1.8 (1.71 to 1.9) |
| Mexico | Acute lymphoid leukemia | death | 0.031 (0.013 to 0.053) | 0.05 (0.02 to 0.09) | 0.132 (0.063 to 0.225) | 0.1 (0.05 to 0.18) | 3.31 (1.88 to 4.59) | 2.57 (2.35 to 2.8) |
| Mexico | Chronic myeloid leukemia | death | 0.016 (0.008 to 0.028) | 0.03 (0.02 to 0.06) | 0.035 (0.017 to 0.058) | 0.03 (0.01 to 0.05) | 1.11 (0.73 to 1.74) | -1.14 (-1.49 to -0.79) |
| Mexico | Chronic lymphoid leukemia | death | 0.007 (0.003 to 0.011) | 0.02 (0.01 to 0.03) | 0.027 (0.013 to 0.046) | 0.02 (0.01 to 0.04) | 3.14 (2.38 to 4.42) | 1.5 (1.34 to 1.67) |
| Mexico | Other leukemia | death | 0.055 (0.025 to 0.093) | 0.11 (0.05 to 0.19) | 0.105 (0.05 to 0.176) | 0.09 (0.04 to 0.15) | 0.92 (0.56 to 1.49) | -0.74 (-0.87 to -0.62) |
| Mexico | Leukemia | DALYs | 5.603 (2.505 to 9.54) | 9.28 (4.17 to 15.63) | 15.276 (7.479 to 25.325) | 11.96 (5.83 to 19.82) | 1.73 (1.22 to 2.4) | 0.87 (0.79 to 0.95) |
| Mexico | Acute myeloid leukemia | DALYs | 1.387 (0.616 to 2.375) | 2.28 (1.02 to 3.88) | 4.803 (2.293 to 8.059) | 3.75 (1.8 to 6.3) | 2.46 (1.76 to 3.42) | 1.66 (1.56 to 1.76) |
| Mexico | Acute lymphoid leukemia | DALYs | 1.387 (0.604 to 2.426) | 2.07 (0.9 to 3.57) | 5.464 (2.658 to 9.307) | 4.19 (2.03 to 7.14) | 2.94 (1.66 to 4.19) | 2.65 (2.44 to 2.86) |
| Mexico | Chronic myeloid leukemia | DALYs | 0.605 (0.282 to 1.023) | 1.06 (0.5 to 1.79) | 1.107 (0.548 to 1.846) | 0.87 (0.43 to 1.45) | 0.83 (0.48 to 1.43) | -1.41 (-1.77 to -1.04) |
| Mexico | Chronic lymphoid leukemia | DALYs | 0.169 (0.079 to 0.285) | 0.37 (0.17 to 0.62) | 0.616 (0.3 to 1.037) | 0.52 (0.26 to 0.88) | 2.64 (1.94 to 3.87) | 1.31 (1.15 to 1.46) |
| Mexico | Other leukemia | DALYs | 2.055 (0.929 to 3.54) | 3.51 (1.59 to 5.98) | 3.285 (1.594 to 5.518) | 2.62 (1.27 to 4.41) | 0.6 (0.27 to 1.14) | -1.08 (-1.25 to -0.91) |
| Micronesia (Federated States of) | Leukemia | death | 0 (0 to 0) | 0.47 (0.19 to 0.86) | 0 (0 to 0.001) | 0.48 (0.21 to 0.9) | 0.53 (-0.05 to 1.25) | -0.14 (-0.32 to 0.04) |
| Micronesia (Federated States of) | Acute myeloid leukemia | death | 0 (0 to 0) | 0.09 (0.04 to 0.18) | 0 (0 to 0) | 0.11 (0.04 to 0.2) | 0.68 (-0.07 to 1.58) | 0.19 (-0.05 to 0.43) |
| Micronesia (Federated States of) | Acute lymphoid leukemia | death | 0 (0 to 0) | 0.03 (0.01 to 0.06) | 0 (0 to 0) | 0.03 (0.01 to 0.06) | 0.68 (-0.03 to 1.63) | 0.13 (-0.11 to 0.37) |
| Micronesia (Federated States of) | Chronic myeloid leukemia | death | 0 (0 to 0) | 0.09 (0.03 to 0.17) | 0 (0 to 0) | 0.07 (0.03 to 0.14) | 0.24 (-0.25 to 0.97) | -1.19 (-1.43 to -0.95) |
| Micronesia (Federated States of) | Chronic lymphoid leukemia | death | 0 (0 to 0) | 0.01 (0 to 0.01) | 0 (0 to 0) | 0.01 (0 to 0.02) | 0.93 (0.2 to 2.17) | 0.35 (0.2 to 0.51) |
| Micronesia (Federated States of) | Other leukemia | death | 0 (0 to 0) | 0.25 (0.09 to 0.47) | 0 (0 to 0) | 0.26 (0.1 to 0.51) | 0.53 (-0.08 to 1.31) | 0 (-0.13 to 0.14) |
| Micronesia (Federated States of) | Leukemia | DALYs | 0.01 (0.004 to 0.019) | 16.25 (7.02 to 29.79) | 0.015 (0.007 to 0.029) | 16.54 (7.13 to 31.23) | 0.48 (-0.16 to 1.21) | -0.2 (-0.4 to 0) |
| Micronesia (Federated States of) | Acute myeloid leukemia | DALYs | 0.002 (0.001 to 0.005) | 3.62 (1.51 to 7.09) | 0.004 (0.001 to 0.008) | 4.16 (1.55 to 7.93) | 0.62 (-0.17 to 1.56) | 0.19 (-0.06 to 0.44) |
| Micronesia (Federated States of) | Acute lymphoid leukemia | DALYs | 0.001 (0 to 0.002) | 1.19 (0.44 to 2.56) | 0.001 (0.001 to 0.003) | 1.35 (0.53 to 2.73) | 0.64 (-0.09 to 1.65) | 0.2 (-0.08 to 0.49) |
| Micronesia (Federated States of) | Chronic myeloid leukemia | DALYs | 0.002 (0.001 to 0.004) | 3 (1.16 to 6.12) | 0.002 (0.001 to 0.004) | 2.37 (0.94 to 4.88) | 0.18 (-0.33 to 0.91) | -1.29 (-1.56 to -1.03) |
| Micronesia (Federated States of) | Chronic lymphoid leukemia | DALYs | 0 (0 to 0) | 0.21 (0.09 to 0.4) | 0 (0 to 0) | 0.26 (0.11 to 0.5) | 0.96 (0.21 to 2.18) | 0.43 (0.25 to 0.61) |
| Micronesia (Federated States of) | Other leukemia | DALYs | 0.005 (0.002 to 0.01) | 8.23 (2.98 to 15.77) | 0.008 (0.003 to 0.015) | 8.4 (3.1 to 16.16) | 0.49 (-0.16 to 1.29) | -0.1 (-0.25 to 0.05) |
| Monaco | Leukemia | death | 0.001 (0 to 0.001) | 0.75 (0.33 to 1.34) | 0.001 (0 to 0.002) | 0.94 (0.45 to 1.63) | 0.73 (0.2 to 1.44) | 1.04 (0.82 to 1.26) |
| Monaco | Acute myeloid leukemia | death | 0 (0 to 0) | 0.22 (0.09 to 0.41) | 0 (0 to 0.001) | 0.34 (0.15 to 0.63) | 1.14 (0.37 to 2.25) | 1.69 (1.47 to 1.91) |
| Monaco | Acute lymphoid leukemia | death | 0 (0 to 0) | 0.03 (0.01 to 0.05) | 0 (0 to 0) | 0.03 (0.01 to 0.06) | 0.49 (-0.33 to 1.26) | 0.64 (0.44 to 0.83) |
| Monaco | Chronic myeloid leukemia | death | 0 (0 to 0) | 0.05 (0.02 to 0.1) | 0 (0 to 0) | 0.03 (0.01 to 0.07) | 0.01 (-0.32 to 0.55) | -1.11 (-1.23 to -1) |
| Monaco | Chronic lymphoid leukemia | death | 0 (0 to 0) | 0.07 (0.03 to 0.14) | 0 (0 to 0) | 0.09 (0.03 to 0.18) | 0.75 (0.1 to 1.74) | 1 (0.7 to 1.31) |
| Monaco | Other leukemia | death | 0 (0 to 0) | 0.38 (0.15 to 0.7) | 0 (0 to 0.001) | 0.45 (0.2 to 0.77) | 0.61 (0.11 to 1.31) | 0.85 (0.63 to 1.08) |
| Monaco | Leukemia | DALYs | 0.011 (0.005 to 0.019) | 18.79 (8.43 to 33.08) | 0.017 (0.008 to 0.029) | 23.34 (11.61 to 39.68) | 0.62 (0.13 to 1.31) | 0.94 (0.73 to 1.14) |
| Monaco | Acute myeloid leukemia | DALYs | 0.003 (0.001 to 0.006) | 5.85 (2.46 to 11.04) | 0.006 (0.003 to 0.011) | 8.57 (4.08 to 15.69) | 0.96 (0.28 to 1.94) | 1.44 (1.25 to 1.64) |
| Monaco | Acute lymphoid leukemia | DALYs | 0 (0 to 0.001) | 1.11 (0.35 to 2.2) | 0.001 (0 to 0.001) | 1.51 (0.27 to 3.13) | 0.58 (-0.29 to 1.42) | 1.09 (0.85 to 1.33) |
| Monaco | Chronic myeloid leukemia | DALYs | 0.001 (0 to 0.001) | 1.36 (0.54 to 2.81) | 0.001 (0 to 0.001) | 1.09 (0.46 to 2.18) | 0.06 (-0.3 to 0.6) | -0.76 (-0.88 to -0.64) |
| Monaco | Chronic lymphoid leukemia | DALYs | 0.001 (0 to 0.002) | 1.53 (0.6 to 2.86) | 0.002 (0.001 to 0.003) | 1.86 (0.62 to 3.5) | 0.68 (0.04 to 1.63) | 0.94 (0.64 to 1.24) |
| Monaco | Other leukemia | DALYs | 0.005 (0.002 to 0.01) | 8.95 (3.7 to 16.62) | 0.008 (0.004 to 0.014) | 10.31 (4.71 to 17.91) | 0.5 (0.02 to 1.11) | 0.75 (0.55 to 0.96) |
| Mongolia | Leukemia | death | 0.002 (0.001 to 0.003) | 0.14 (0.06 to 0.27) | 0.005 (0.002 to 0.01) | 0.2 (0.09 to 0.37) | 2.21 (1.3 to 3.8) | 1.01 (0.85 to 1.17) |
| Mongolia | Acute myeloid leukemia | death | 0.001 (0 to 0.002) | 0.07 (0.03 to 0.13) | 0.003 (0.001 to 0.006) | 0.11 (0.05 to 0.21) | 2.61 (1.51 to 4.6) | 1.42 (1.31 to 1.52) |
| Mongolia | Acute lymphoid leukemia | death | 0 (0 to 0) | 0.01 (0 to 0.02) | 0 (0 to 0.001) | 0.01 (0.01 to 0.03) | 2.12 (0.93 to 4.2) | 0.82 (0.66 to 0.98) |
| Mongolia | Chronic myeloid leukemia | death | 0 (0 to 0) | 0.01 (0 to 0.02) | 0 (0 to 0.001) | 0.02 (0.01 to 0.03) | 2.3 (1.03 to 4.43) | 0.77 (0.62 to 0.92) |
| Mongolia | Chronic lymphoid leukemia | death | 0 (0 to 0) | 0.01 (0 to 0.02) | 0 (0 to 0.001) | 0.02 (0.01 to 0.04) | 2.59 (1.16 to 5.25) | 1.59 (1.23 to 1.95) |
| Mongolia | Other leukemia | death | 0 (0 to 0.001) | 0.04 (0.01 to 0.08) | 0.001 (0 to 0.002) | 0.04 (0.02 to 0.08) | 1.21 (0.54 to 2.57) | 0.12 (-0.14 to 0.37) |
| Mongolia | Leukemia | DALYs | 0.06 (0.023 to 0.115) | 4.51 (1.81 to 8.45) | 0.201 (0.093 to 0.36) | 6.38 (2.93 to 11.3) | 2.34 (1.34 to 4.19) | 0.99 (0.83 to 1.15) |
| Mongolia | Acute myeloid leukemia | DALYs | 0.034 (0.013 to 0.065) | 2.45 (0.96 to 4.61) | 0.126 (0.056 to 0.233) | 3.84 (1.72 to 7.19) | 2.71 (1.54 to 4.86) | 1.46 (1.34 to 1.57) |
| Mongolia | Acute lymphoid leukemia | DALYs | 0.006 (0.002 to 0.014) | 0.4 (0.14 to 0.87) | 0.019 (0.008 to 0.04) | 0.56 (0.24 to 1.13) | 2.12 (0.92 to 4.5) | 0.85 (0.69 to 1) |
| Mongolia | Chronic myeloid leukemia | DALYs | 0.005 (0.002 to 0.01) | 0.38 (0.15 to 0.76) | 0.016 (0.007 to 0.035) | 0.51 (0.22 to 1.05) | 2.44 (1.09 to 4.76) | 0.67 (0.5 to 0.84) |
| Mongolia | Chronic lymphoid leukemia | DALYs | 0.004 (0.001 to 0.008) | 0.31 (0.11 to 0.64) | 0.014 (0.005 to 0.027) | 0.47 (0.18 to 0.94) | 2.63 (1.13 to 5.26) | 1.29 (0.93 to 1.65) |
| Mongolia | Other leukemia | DALYs | 0.012 (0.004 to 0.025) | 0.98 (0.37 to 1.93) | 0.026 (0.011 to 0.052) | 1 (0.44 to 1.93) | 1.26 (0.49 to 2.86) | -0.32 (-0.6 to -0.04) |
| Montenegro | Leukemia | death | 0.003 (0.001 to 0.004) | 0.41 (0.2 to 0.68) | 0.005 (0.002 to 0.008) | 0.5 (0.26 to 0.84) | 0.88 (0.44 to 1.39) | 0.92 (0.85 to 0.98) |
| Montenegro | Acute myeloid leukemia | death | 0.001 (0 to 0.002) | 0.14 (0.07 to 0.24) | 0.002 (0.001 to 0.003) | 0.17 (0.09 to 0.28) | 0.78 (0.26 to 1.47) | 0.8 (0.74 to 0.86) |
| Montenegro | Acute lymphoid leukemia | death | 0 (0 to 0.001) | 0.05 (0.02 to 0.08) | 0 (0 to 0.001) | 0.05 (0.02 to 0.08) | 0.31 (-0.12 to 0.84) | -0.11 (-0.16 to -0.05) |
| Montenegro | Chronic myeloid leukemia | death | 0 (0 to 0.001) | 0.07 (0.03 to 0.13) | 0.001 (0 to 0.001) | 0.06 (0.03 to 0.1) | 0.26 (-0.19 to 0.86) | -0.57 (-0.89 to -0.25) |
| Montenegro | Chronic lymphoid leukemia | death | 0 (0 to 0.001) | 0.08 (0.04 to 0.14) | 0.002 (0.001 to 0.003) | 0.18 (0.09 to 0.3) | 2.61 (1.48 to 4.26) | 3.29 (3.05 to 3.54) |
| Montenegro | Other leukemia | death | 0 (0 to 0.001) | 0.07 (0.03 to 0.12) | 0 (0 to 0.001) | 0.05 (0.02 to 0.08) | 0.09 (-0.3 to 0.61) | -1.39 (-1.56 to -1.22) |
| Montenegro | Leukemia | DALYs | 0.073 (0.037 to 0.12) | 11.41 (5.66 to 18.63) | 0.122 (0.063 to 0.202) | 13.47 (6.97 to 22.2) | 0.67 (0.29 to 1.13) | 0.75 (0.66 to 0.83) |
| Montenegro | Acute myeloid leukemia | DALYs | 0.028 (0.014 to 0.047) | 4.29 (2.09 to 7.26) | 0.044 (0.023 to 0.072) | 5.07 (2.6 to 8.36) | 0.59 (0.17 to 1.15) | 0.73 (0.67 to 0.78) |
| Montenegro | Acute lymphoid leukemia | DALYs | 0.011 (0.005 to 0.019) | 1.72 (0.82 to 2.96) | 0.014 (0.007 to 0.024) | 1.76 (0.85 to 3.11) | 0.23 (-0.17 to 0.74) | 0.14 (0.05 to 0.23) |
| Montenegro | Chronic myeloid leukemia | DALYs | 0.013 (0.006 to 0.024) | 2.05 (0.92 to 3.66) | 0.014 (0.007 to 0.025) | 1.6 (0.79 to 2.7) | 0.1 (-0.28 to 0.64) | -0.85 (-1.23 to -0.47) |
| Montenegro | Chronic lymphoid leukemia | DALYs | 0.011 (0.006 to 0.02) | 1.82 (0.89 to 3.16) | 0.04 (0.02 to 0.067) | 3.99 (2.02 to 6.69) | 2.53 (1.42 to 4.23) | 3.27 (2.99 to 3.55) |
| Montenegro | Other leukemia | DALYs | 0.01 (0.005 to 0.017) | 1.53 (0.74 to 2.73) | 0.01 (0.005 to 0.018) | 1.04 (0.49 to 1.91) | 0 (-0.33 to 0.49) | -1.48 (-1.6 to -1.36) |
| Morocco | Leukemia | death | 0.019 (0.008 to 0.034) | 0.13 (0.05 to 0.23) | 0.069 (0.034 to 0.12) | 0.22 (0.11 to 0.38) | 2.62 (1.61 to 4.52) | 1.83 (1.74 to 1.92) |
| Morocco | Acute myeloid leukemia | death | 0.005 (0.002 to 0.009) | 0.03 (0.01 to 0.05) | 0.02 (0.009 to 0.037) | 0.06 (0.03 to 0.11) | 3.48 (2.05 to 5.83) | 2.53 (2.44 to 2.62) |
| Morocco | Acute lymphoid leukemia | death | 0.002 (0.001 to 0.003) | 0.01 (0 to 0.02) | 0.006 (0.003 to 0.011) | 0.02 (0.01 to 0.03) | 2.67 (1.44 to 5.02) | 2.01 (1.95 to 2.07) |
| Morocco | Chronic myeloid leukemia | death | 0.003 (0.001 to 0.007) | 0.02 (0.01 to 0.04) | 0.009 (0.004 to 0.017) | 0.03 (0.01 to 0.05) | 1.57 (0.73 to 3.04) | 0.57 (0.44 to 0.7) |
| Morocco | Chronic lymphoid leukemia | death | 0.001 (0 to 0.002) | 0.01 (0 to 0.01) | 0.004 (0.002 to 0.008) | 0.01 (0.01 to 0.02) | 4.23 (2.45 to 7.34) | 2.77 (2.5 to 3.03) |
| Morocco | Other leukemia | death | 0.009 (0.003 to 0.017) | 0.06 (0.02 to 0.12) | 0.03 (0.013 to 0.056) | 0.1 (0.04 to 0.18) | 2.43 (1.41 to 4.87) | 1.73 (1.64 to 1.81) |
| Morocco | Leukemia | DALYs | 0.627 (0.267 to 1.13) | 3.78 (1.62 to 6.73) | 2.071 (1.029 to 3.68) | 5.95 (2.96 to 10.56) | 2.3 (1.35 to 4.18) | 1.61 (1.55 to 1.67) |
| Morocco | Acute myeloid leukemia | DALYs | 0.157 (0.063 to 0.303) | 0.92 (0.37 to 1.75) | 0.664 (0.289 to 1.22) | 1.84 (0.8 to 3.36) | 3.24 (1.86 to 5.66) | 2.47 (2.41 to 2.53) |
| Morocco | Acute lymphoid leukemia | DALYs | 0.062 (0.023 to 0.125) | 0.33 (0.13 to 0.66) | 0.199 (0.09 to 0.382) | 0.55 (0.25 to 1.04) | 2.2 (1.1 to 4.55) | 1.69 (1.62 to 1.77) |
| Morocco | Chronic myeloid leukemia | DALYs | 0.119 (0.045 to 0.236) | 0.7 (0.27 to 1.38) | 0.268 (0.118 to 0.5) | 0.76 (0.34 to 1.42) | 1.25 (0.49 to 2.58) | 0.19 (0.13 to 0.25) |
| Morocco | Chronic lymphoid leukemia | DALYs | 0.023 (0.008 to 0.045) | 0.15 (0.05 to 0.29) | 0.111 (0.045 to 0.218) | 0.33 (0.13 to 0.63) | 3.93 (2.26 to 7) | 2.65 (2.48 to 2.83) |
| Morocco | Other leukemia | DALYs | 0.267 (0.097 to 0.522) | 1.68 (0.61 to 3.28) | 0.828 (0.362 to 1.6) | 2.46 (1.08 to 4.68) | 2.11 (1.11 to 4.56) | 1.45 (1.37 to 1.53) |
| Mozambique | Leukemia | death | 0.003 (0.001 to 0.007) | 0.04 (0.01 to 0.11) | 0.015 (0.006 to 0.031) | 0.13 (0.05 to 0.25) | 4.88 (2.49 to 13.71) | 4.59 (4.34 to 4.84) |
| Mozambique | Acute myeloid leukemia | death | 0 (0 to 0.001) | 0 (0 to 0.01) | 0.002 (0.001 to 0.004) | 0.01 (0 to 0.03) | 6.95 (3.39 to 20.58) | 5.38 (5.17 to 5.59) |
| Mozambique | Acute lymphoid leukemia | death | 0 (0 to 0) | 0 (0 to 0) | 0.001 (0 to 0.002) | 0.01 (0 to 0.01) | 6.54 (3.22 to 21.02) | 4.89 (4.68 to 5.1) |
| Mozambique | Chronic myeloid leukemia | death | 0.001 (0 to 0.002) | 0.01 (0 to 0.02) | 0.003 (0.001 to 0.007) | 0.02 (0.01 to 0.05) | 5.13 (2.46 to 14.48) | 4.92 (4.57 to 5.28) |
| Mozambique | Chronic lymphoid leukemia | death | 0.001 (0 to 0.002) | 0.01 (0 to 0.03) | 0.004 (0.001 to 0.009) | 0.05 (0.01 to 0.11) | 6.8 (3.37 to 20.14) | 5.89 (5.59 to 6.18) |
| Mozambique | Other leukemia | death | 0.001 (0 to 0.003) | 0.02 (0 to 0.05) | 0.005 (0.002 to 0.01) | 0.04 (0.01 to 0.08) | 3.28 (1.46 to 10.41) | 3.08 (2.91 to 3.25) |
| Mozambique | Leukemia | DALYs | 0.089 (0.019 to 0.235) | 1.19 (0.24 to 3.13) | 0.558 (0.221 to 1.089) | 3.62 (1.41 to 7.12) | 5.26 (2.64 to 14.79) | 4.69 (4.43 to 4.94) |
| Mozambique | Acute myeloid leukemia | DALYs | 0.009 (0.002 to 0.026) | 0.11 (0.02 to 0.31) | 0.078 (0.028 to 0.164) | 0.44 (0.16 to 0.91) | 7.79 (3.78 to 23.33) | 5.62 (5.41 to 5.82) |
| Mozambique | Acute lymphoid leukemia | DALYs | 0.005 (0.001 to 0.016) | 0.06 (0.01 to 0.19) | 0.046 (0.017 to 0.099) | 0.22 (0.08 to 0.48) | 7.57 (3.69 to 24.53) | 5.19 (4.98 to 5.4) |
| Mozambique | Chronic myeloid leukemia | DALYs | 0.022 (0.004 to 0.059) | 0.26 (0.05 to 0.71) | 0.14 (0.049 to 0.287) | 0.81 (0.29 to 1.66) | 5.48 (2.47 to 15.73) | 5.04 (4.68 to 5.4) |
| Mozambique | Chronic lymphoid leukemia | DALYs | 0.013 (0.002 to 0.036) | 0.22 (0.04 to 0.63) | 0.101 (0.036 to 0.21) | 0.96 (0.32 to 2.02) | 7.04 (3.38 to 20.76) | 6.13 (5.81 to 6.46) |
| Mozambique | Other leukemia | DALYs | 0.041 (0.008 to 0.107) | 0.53 (0.11 to 1.42) | 0.193 (0.077 to 0.38) | 1.19 (0.46 to 2.37) | 3.75 (1.68 to 11.64) | 3.36 (3.18 to 3.53) |
| Myanmar | Leukemia | death | 0.022 (0.004 to 0.069) | 0.08 (0.01 to 0.25) | 0.087 (0.031 to 0.168) | 0.18 (0.06 to 0.35) | 2.93 (1.16 to 10.81) | 3.05 (2.88 to 3.22) |
| Myanmar | Acute myeloid leukemia | death | 0.002 (0 to 0.007) | 0.01 (0 to 0.02) | 0.016 (0.005 to 0.034) | 0.03 (0.01 to 0.07) | 6.63 (2.74 to 25.73) | 5.68 (5.38 to 5.97) |
| Myanmar | Acute lymphoid leukemia | death | 0.001 (0 to 0.005) | 0 (0 to 0.02) | 0.007 (0.002 to 0.015) | 0.01 (0 to 0.03) | 4.37 (1.58 to 18.2) | 4.55 (4.32 to 4.78) |
| Myanmar | Chronic myeloid leukemia | death | 0.002 (0 to 0.006) | 0.01 (0 to 0.02) | 0.006 (0.002 to 0.012) | 0.01 (0 to 0.02) | 1.79 (0.42 to 8.29) | 1.74 (1.54 to 1.93) |
| Myanmar | Chronic lymphoid leukemia | death | 0 (0 to 0.001) | 0 (0 to 0.01) | 0.003 (0.001 to 0.006) | 0.01 (0 to 0.01) | 5.54 (2.47 to 19.11) | 4.71 (4.47 to 4.95) |
| Myanmar | Other leukemia | death | 0.016 (0.003 to 0.053) | 0.06 (0.01 to 0.19) | 0.056 (0.021 to 0.112) | 0.12 (0.04 to 0.23) | 2.42 (0.82 to 9.44) | 2.54 (2.39 to 2.69) |
| Myanmar | Leukemia | DALYs | 0.859 (0.133 to 2.745) | 2.81 (0.45 to 8.65) | 2.978 (1.071 to 5.8) | 5.59 (2.01 to 10.87) | 2.47 (0.84 to 9.95) | 2.78 (2.61 to 2.95) |
| Myanmar | Acute myeloid leukemia | DALYs | 0.079 (0.012 to 0.256) | 0.26 (0.04 to 0.84) | 0.557 (0.177 to 1.237) | 1.03 (0.33 to 2.27) | 6.05 (2.32 to 23.67) | 5.59 (5.3 to 5.88) |
| Myanmar | Acute lymphoid leukemia | DALYs | 0.055 (0.006 to 0.224) | 0.16 (0.02 to 0.65) | 0.26 (0.086 to 0.611) | 0.47 (0.16 to 1.11) | 3.73 (1.1 to 17.76) | 4.28 (4.04 to 4.51) |
| Myanmar | Chronic myeloid leukemia | DALYs | 0.075 (0.01 to 0.239) | 0.25 (0.03 to 0.76) | 0.185 (0.061 to 0.396) | 0.35 (0.12 to 0.74) | 1.48 (0.22 to 7.76) | 1.44 (1.28 to 1.6) |
| Myanmar | Chronic lymphoid leukemia | DALYs | 0.014 (0.002 to 0.042) | 0.05 (0.01 to 0.16) | 0.082 (0.029 to 0.177) | 0.16 (0.06 to 0.36) | 5 (2.18 to 17.61) | 4.48 (4.27 to 4.7) |
| Myanmar | Other leukemia | DALYs | 0.636 (0.091 to 2.153) | 2.08 (0.32 to 6.74) | 1.894 (0.696 to 3.774) | 3.58 (1.31 to 7.13) | 1.98 (0.53 to 8.51) | 2.2 (2.03 to 2.37) |
| Namibia | Leukemia | death | 0.001 (0 to 0.001) | 0.11 (0.05 to 0.2) | 0.003 (0.001 to 0.005) | 0.18 (0.09 to 0.31) | 2.17 (1.12 to 4.08) | 1.69 (1.52 to 1.87) |
| Namibia | Acute myeloid leukemia | death | 0 (0 to 0) | 0.01 (0 to 0.01) | 0 (0 to 0) | 0.01 (0.01 to 0.03) | 2.79 (1.09 to 5.82) | 2.02 (1.88 to 2.15) |
| Namibia | Acute lymphoid leukemia | death | 0 (0 to 0) | 0 (0 to 0.01) | 0 (0 to 0) | 0 (0 to 0.01) | 2.66 (0.96 to 5.87) | 1.88 (1.64 to 2.12) |
| Namibia | Chronic myeloid leukemia | death | 0 (0 to 0) | 0 (0 to 0.01) | 0 (0 to 0) | 0 (0 to 0.01) | 1.8 (0.6 to 4.18) | 1.05 (0.78 to 1.33) |
| Namibia | Chronic lymphoid leukemia | death | 0 (0 to 0.001) | 0.05 (0.02 to 0.11) | 0.001 (0.001 to 0.003) | 0.11 (0.04 to 0.21) | 2.89 (1.47 to 5.38) | 2.58 (2.43 to 2.74) |
| Namibia | Other leukemia | death | 0 (0 to 0.001) | 0.04 (0.02 to 0.08) | 0.001 (0 to 0.001) | 0.05 (0.02 to 0.09) | 1.28 (0.52 to 2.95) | 0.3 (0.05 to 0.54) |
| Namibia | Leukemia | DALYs | 0.026 (0.011 to 0.047) | 3.09 (1.3 to 5.67) | 0.077 (0.037 to 0.143) | 4.76 (2.32 to 8.55) | 2.02 (0.96 to 3.97) | 1.4 (1.18 to 1.62) |
| Namibia | Acute myeloid leukemia | DALYs | 0.003 (0.001 to 0.006) | 0.29 (0.11 to 0.58) | 0.01 (0.004 to 0.02) | 0.52 (0.22 to 1.01) | 2.82 (0.97 to 6.21) | 1.96 (1.78 to 2.13) |
| Namibia | Acute lymphoid leukemia | DALYs | 0.001 (0 to 0.002) | 0.1 (0.04 to 0.2) | 0.004 (0.001 to 0.008) | 0.18 (0.07 to 0.38) | 2.76 (0.86 to 6.76) | 1.96 (1.72 to 2.21) |
| Namibia | Chronic myeloid leukemia | DALYs | 0.001 (0 to 0.002) | 0.1 (0.04 to 0.2) | 0.002 (0.001 to 0.005) | 0.14 (0.06 to 0.27) | 1.71 (0.47 to 4.47) | 0.79 (0.46 to 1.11) |
| Namibia | Chronic lymphoid leukemia | DALYs | 0.009 (0.004 to 0.018) | 1.26 (0.48 to 2.51) | 0.035 (0.015 to 0.066) | 2.4 (1.01 to 4.64) | 2.72 (1.33 to 5.17) | 2.36 (2.19 to 2.54) |
| Namibia | Other leukemia | DALYs | 0.012 (0.005 to 0.021) | 1.34 (0.57 to 2.47) | 0.026 (0.012 to 0.047) | 1.51 (0.71 to 2.68) | 1.24 (0.39 to 3) | 0.11 (-0.19 to 0.41) |
| Nauru | Leukemia | death | 0 (0 to 0) | 0.55 (0.23 to 0.98) | 0 (0 to 0) | 0.51 (0.23 to 0.92) | 0.05 (-0.24 to 0.43) | -0.77 (-0.97 to -0.57) |
| Nauru | Acute myeloid leukemia | death | 0 (0 to 0) | 0.13 (0.05 to 0.25) | 0 (0 to 0) | 0.12 (0.05 to 0.22) | 0.08 (-0.31 to 0.57) | -0.83 (-1.21 to -0.45) |
| Nauru | Acute lymphoid leukemia | death | 0 (0 to 0) | 0.03 (0.01 to 0.08) | 0 (0 to 0) | 0.03 (0.01 to 0.07) | 0.21 (-0.15 to 0.77) | -0.48 (-0.72 to -0.24) |
| Nauru | Chronic myeloid leukemia | death | 0 (0 to 0) | 0.1 (0.04 to 0.21) | 0 (0 to 0) | 0.08 (0.03 to 0.15) | -0.1 (-0.4 to 0.32) | -1.34 (-1.52 to -1.17) |
| Nauru | Chronic lymphoid leukemia | death | 0 (0 to 0) | 0.01 (0 to 0.02) | 0 (0 to 0) | 0.01 (0 to 0.02) | 0.21 (-0.2 to 0.82) | -0.52 (-0.88 to -0.15) |
| Nauru | Other leukemia | death | 0 (0 to 0) | 0.28 (0.11 to 0.52) | 0 (0 to 0) | 0.27 (0.11 to 0.51) | 0.07 (-0.24 to 0.45) | -0.61 (-0.79 to -0.43) |
| Nauru | Leukemia | DALYs | 0.001 (0 to 0.002) | 18.26 (7.72 to 32.62) | 0.001 (0.001 to 0.002) | 16.89 (7.44 to 30.84) | 0.09 (-0.23 to 0.49) | -0.71 (-0.88 to -0.53) |
| Nauru | Acute myeloid leukemia | DALYs | 0 (0 to 0.001) | 4.76 (2.01 to 9.01) | 0 (0 to 0.001) | 4.48 (1.99 to 8.21) | 0.11 (-0.28 to 0.64) | -0.73 (-1.09 to -0.38) |
| Nauru | Acute lymphoid leukemia | DALYs | 0 (0 to 0) | 1.26 (0.44 to 3.09) | 0 (0 to 0) | 1.32 (0.48 to 2.78) | 0.24 (-0.16 to 0.87) | -0.36 (-0.58 to -0.15) |
| Nauru | Chronic myeloid leukemia | DALYs | 0 (0 to 0) | 3.4 (1.14 to 6.9) | 0 (0 to 0) | 2.63 (0.89 to 5.26) | -0.09 (-0.4 to 0.38) | -1.31 (-1.52 to -1.11) |
| Nauru | Chronic lymphoid leukemia | DALYs | 0 (0 to 0) | 0.3 (0.13 to 0.61) | 0 (0 to 0) | 0.32 (0.13 to 0.6) | 0.28 (-0.16 to 0.87) | -0.32 (-0.65 to 0) |
| Nauru | Other leukemia | DALYs | 0.001 (0 to 0.001) | 8.53 (3.44 to 15.73) | 0.001 (0 to 0.001) | 8.14 (3.24 to 15.31) | 0.11 (-0.22 to 0.53) | -0.54 (-0.7 to -0.38) |
| Nepal | Leukemia | death | 0.004 (0.001 to 0.009) | 0.03 (0.01 to 0.09) | 0.025 (0.01 to 0.051) | 0.11 (0.04 to 0.22) | 6.02 (3.42 to 14.95) | 4.53 (4.29 to 4.77) |
| Nepal | Acute myeloid leukemia | death | 0.001 (0 to 0.002) | 0.01 (0 to 0.02) | 0.006 (0.002 to 0.012) | 0.03 (0.01 to 0.05) | 8.71 (4.62 to 22.51) | 5.68 (5.5 to 5.85) |
| Nepal | Acute lymphoid leukemia | death | 0 (0 to 0.001) | 0 (0 to 0) | 0.002 (0.001 to 0.003) | 0.01 (0 to 0.01) | 6.19 (2.99 to 17.74) | 4.97 (4.75 to 5.18) |
| Nepal | Chronic myeloid leukemia | death | 0.001 (0 to 0.004) | 0.01 (0 to 0.03) | 0.007 (0.002 to 0.014) | 0.03 (0.01 to 0.06) | 3.91 (1.93 to 11.11) | 3.36 (3 to 3.72) |
| Nepal | Chronic lymphoid leukemia | death | 0.001 (0 to 0.002) | 0.01 (0 to 0.02) | 0.007 (0.002 to 0.014) | 0.03 (0.01 to 0.07) | 9.95 (5.65 to 25.15) | 5.68 (5.5 to 5.87) |
| Nepal | Other leukemia | death | 0.001 (0 to 0.002) | 0.01 (0 to 0.02) | 0.004 (0.001 to 0.009) | 0.02 (0.01 to 0.04) | 4.51 (2.36 to 13.49) | 3.54 (3.36 to 3.72) |
| Nepal | Leukemia | DALYs | 0.132 (0.026 to 0.352) | 1.06 (0.22 to 2.73) | 0.822 (0.316 to 1.597) | 3.21 (1.22 to 6.24) | 5.22 (2.78 to 13.75) | 4.36 (4.05 to 4.67) |
| Nepal | Acute myeloid leukemia | DALYs | 0.024 (0.005 to 0.067) | 0.19 (0.04 to 0.51) | 0.225 (0.083 to 0.443) | 0.84 (0.31 to 1.63) | 8.23 (4.16 to 21.92) | 5.8 (5.58 to 6.03) |
| Nepal | Acute lymphoid leukemia | DALYs | 0.01 (0.002 to 0.029) | 0.07 (0.01 to 0.2) | 0.069 (0.026 to 0.144) | 0.24 (0.09 to 0.5) | 5.73 (2.57 to 17.36) | 4.91 (4.67 to 5.15) |
| Nepal | Chronic myeloid leukemia | DALYs | 0.055 (0.01 to 0.149) | 0.42 (0.08 to 1.11) | 0.241 (0.089 to 0.495) | 0.92 (0.33 to 1.9) | 3.39 (1.54 to 10.3) | 3.18 (2.75 to 3.6) |
| Nepal | Chronic lymphoid leukemia | DALYs | 0.015 (0.003 to 0.043) | 0.16 (0.03 to 0.44) | 0.155 (0.05 to 0.343) | 0.69 (0.22 to 1.52) | 9.01 (4.81 to 23.17) | 5.61 (5.35 to 5.86) |
| Nepal | Other leukemia | DALYs | 0.027 (0.005 to 0.079) | 0.22 (0.04 to 0.64) | 0.132 (0.045 to 0.269) | 0.52 (0.18 to 1.09) | 3.85 (1.77 to 12.36) | 3.39 (3.15 to 3.64) |
| Netherlands | Leukemia | death | 0.063 (0.027 to 0.113) | 0.32 (0.13 to 0.57) | 0.141 (0.065 to 0.245) | 0.4 (0.19 to 0.7) | 1.23 (0.92 to 1.66) | 0.94 (0.82 to 1.06) |
| Netherlands | Acute myeloid leukemia | death | 0.021 (0.009 to 0.038) | 0.11 (0.04 to 0.2) | 0.055 (0.025 to 0.096) | 0.17 (0.08 to 0.29) | 1.61 (1.2 to 2.21) | 1.83 (1.63 to 2.04) |
| Netherlands | Acute lymphoid leukemia | death | 0.004 (0.002 to 0.007) | 0.02 (0.01 to 0.04) | 0.007 (0.003 to 0.011) | 0.02 (0.01 to 0.04) | 0.65 (0.35 to 1.19) | -0.01 (-0.11 to 0.09) |
| Netherlands | Chronic myeloid leukemia | death | 0.008 (0.004 to 0.015) | 0.04 (0.02 to 0.08) | 0.004 (0.002 to 0.007) | 0.01 (0.01 to 0.02) | -0.5 (-0.59 to -0.35) | -5.12 (-5.6 to -4.64) |
| Netherlands | Chronic lymphoid leukemia | death | 0.014 (0.006 to 0.026) | 0.07 (0.03 to 0.13) | 0.008 (0.003 to 0.018) | 0.02 (0.01 to 0.05) | -0.41 (-0.56 to 0.22) | -4.47 (-5.68 to -3.25) |
| Netherlands | Other leukemia | death | 0.015 (0.007 to 0.028) | 0.08 (0.03 to 0.14) | 0.066 (0.027 to 0.118) | 0.18 (0.08 to 0.32) | 3.29 (2.35 to 4.37) | 3.28 (2.97 to 3.6) |
| Netherlands | Leukemia | DALYs | 1.451 (0.609 to 2.627) | 7.63 (3.2 to 13.86) | 2.713 (1.282 to 4.688) | 8.95 (4.22 to 15.32) | 0.87 (0.64 to 1.26) | 0.51 (0.37 to 0.65) |
| Netherlands | Acute myeloid leukemia | DALYs | 0.541 (0.221 to 0.989) | 2.9 (1.19 to 5.31) | 1.164 (0.543 to 1.998) | 3.97 (1.85 to 6.88) | 1.15 (0.83 to 1.63) | 1.27 (1.1 to 1.44) |
| Netherlands | Acute lymphoid leukemia | DALYs | 0.14 (0.057 to 0.258) | 0.78 (0.32 to 1.44) | 0.222 (0.1 to 0.395) | 0.92 (0.42 to 1.64) | 0.59 (0.24 to 1.15) | 0.34 (0.19 to 0.48) |
| Netherlands | Chronic myeloid leukemia | DALYs | 0.2 (0.086 to 0.368) | 1.07 (0.45 to 1.96) | 0.102 (0.046 to 0.18) | 0.35 (0.16 to 0.62) | -0.49 (-0.58 to -0.34) | -4.7 (-5.16 to -4.24) |
| Netherlands | Chronic lymphoid leukemia | DALYs | 0.265 (0.118 to 0.479) | 1.32 (0.58 to 2.37) | 0.158 (0.068 to 0.332) | 0.46 (0.2 to 0.98) | -0.41 (-0.55 to 0.31) | -4.31 (-5.5 to -3.11) |
| Netherlands | Other leukemia | DALYs | 0.305 (0.13 to 0.547) | 1.56 (0.66 to 2.82) | 1.068 (0.471 to 1.861) | 3.24 (1.42 to 5.57) | 2.5 (1.78 to 3.36) | 2.66 (2.39 to 2.92) |
| New Zealand | Leukemia | death | 0.016 (0.007 to 0.027) | 0.41 (0.19 to 0.71) | 0.036 (0.018 to 0.06) | 0.46 (0.23 to 0.77) | 1.23 (0.83 to 1.76) | 0.3 (0.18 to 0.41) |
| New Zealand | Acute myeloid leukemia | death | 0.007 (0.003 to 0.012) | 0.18 (0.08 to 0.3) | 0.018 (0.009 to 0.03) | 0.24 (0.12 to 0.4) | 1.64 (1.13 to 2.28) | 0.98 (0.76 to 1.2) |
| New Zealand | Acute lymphoid leukemia | death | 0.001 (0.001 to 0.002) | 0.03 (0.01 to 0.05) | 0.002 (0.001 to 0.003) | 0.02 (0.01 to 0.04) | 0.36 (0.04 to 1.21) | -0.85 (-1.01 to -0.69) |
| New Zealand | Chronic myeloid leukemia | death | 0.002 (0.001 to 0.004) | 0.06 (0.03 to 0.11) | 0.002 (0.001 to 0.003) | 0.02 (0.01 to 0.04) | -0.32 (-0.45 to -0.13) | -4.51 (-4.86 to -4.15) |
| New Zealand | Chronic lymphoid leukemia | death | 0.003 (0.002 to 0.006) | 0.08 (0.04 to 0.14) | 0.009 (0.004 to 0.016) | 0.11 (0.05 to 0.18) | 1.75 (1.15 to 2.6) | 0.75 (0.52 to 0.97) |
| New Zealand | Other leukemia | death | 0.002 (0.001 to 0.004) | 0.06 (0.03 to 0.1) | 0.005 (0.003 to 0.009) | 0.06 (0.03 to 0.11) | 1.41 (0.78 to 2.1) | 0.88 (0.71 to 1.05) |
| New Zealand | Leukemia | DALYs | 0.401 (0.189 to 0.681) | 10.53 (4.95 to 17.83) | 0.769 (0.383 to 1.253) | 11.2 (5.63 to 18.14) | 0.92 (0.59 to 1.36) | 0.08 (-0.03 to 0.18) |
| New Zealand | Acute myeloid leukemia | DALYs | 0.176 (0.082 to 0.298) | 4.64 (2.16 to 7.85) | 0.408 (0.201 to 0.663) | 6.11 (3.04 to 9.93) | 1.31 (0.88 to 1.87) | 0.81 (0.6 to 1.02) |
| New Zealand | Acute lymphoid leukemia | DALYs | 0.043 (0.02 to 0.074) | 1.16 (0.55 to 2) | 0.053 (0.027 to 0.088) | 0.98 (0.5 to 1.61) | 0.25 (-0.04 to 1.04) | -0.65 (-0.81 to -0.5) |
| New Zealand | Chronic myeloid leukemia | DALYs | 0.067 (0.032 to 0.114) | 1.79 (0.87 to 3.07) | 0.041 (0.02 to 0.067) | 0.65 (0.32 to 1.07) | -0.39 (-0.51 to -0.21) | -4.41 (-4.76 to -4.06) |
| New Zealand | Chronic lymphoid leukemia | DALYs | 0.067 (0.031 to 0.115) | 1.69 (0.77 to 2.92) | 0.168 (0.083 to 0.282) | 2.11 (1.05 to 3.52) | 1.51 (0.98 to 2.31) | 0.62 (0.39 to 0.85) |
| New Zealand | Other leukemia | DALYs | 0.048 (0.023 to 0.083) | 1.24 (0.58 to 2.13) | 0.099 (0.048 to 0.165) | 1.35 (0.67 to 2.23) | 1.04 (0.53 to 1.58) | 0.57 (0.43 to 0.7) |
| Nicaragua | Leukemia | death | 0.003 (0.001 to 0.006) | 0.15 (0.05 to 0.29) | 0.014 (0.007 to 0.025) | 0.3 (0.14 to 0.52) | 3.86 (2.57 to 6.74) | 2.35 (2.05 to 2.65) |
| Nicaragua | Acute myeloid leukemia | death | 0 (0 to 0.001) | 0.03 (0.01 to 0.05) | 0.003 (0.001 to 0.005) | 0.06 (0.03 to 0.1) | 4.93 (3.06 to 8.57) | 2.92 (2.74 to 3.11) |
| Nicaragua | Acute lymphoid leukemia | death | 0.001 (0 to 0.001) | 0.03 (0.01 to 0.06) | 0.004 (0.002 to 0.007) | 0.08 (0.03 to 0.14) | 5.86 (2.87 to 11.42) | 4.42 (4.09 to 4.75) |
| Nicaragua | Chronic myeloid leukemia | death | 0 (0 to 0.001) | 0.02 (0.01 to 0.03) | 0.002 (0.001 to 0.003) | 0.03 (0.02 to 0.06) | 3.59 (2.28 to 6.41) | 2.47 (2.02 to 2.92) |
| Nicaragua | Chronic lymphoid leukemia | death | 0 (0 to 0.001) | 0.02 (0.01 to 0.03) | 0.001 (0.001 to 0.002) | 0.03 (0.01 to 0.05) | 3.19 (2 to 5.5) | 0.75 (0.05 to 1.45) |
| Nicaragua | Other leukemia | death | 0.001 (0 to 0.002) | 0.06 (0.02 to 0.13) | 0.004 (0.002 to 0.008) | 0.1 (0.04 to 0.18) | 2.65 (1.6 to 5.09) | 1.33 (0.99 to 1.67) |
| Nicaragua | Leukemia | DALYs | 0.113 (0.039 to 0.228) | 5.02 (1.79 to 9.8) | 0.461 (0.217 to 0.815) | 8.53 (4.02 to 14.9) | 3.08 (1.87 to 5.85) | 1.93 (1.76 to 2.11) |
| Nicaragua | Acute myeloid leukemia | DALYs | 0.019 (0.006 to 0.038) | 0.85 (0.3 to 1.7) | 0.097 (0.045 to 0.173) | 1.78 (0.83 to 3.13) | 4.22 (2.52 to 7.84) | 2.62 (2.47 to 2.78) |
| Nicaragua | Acute lymphoid leukemia | DALYs | 0.027 (0.009 to 0.058) | 1.06 (0.36 to 2.21) | 0.158 (0.063 to 0.289) | 2.69 (1.09 to 4.92) | 4.82 (2.17 to 9.9) | 4.02 (3.74 to 4.31) |
| Nicaragua | Chronic myeloid leukemia | DALYs | 0.013 (0.005 to 0.026) | 0.6 (0.22 to 1.15) | 0.047 (0.022 to 0.085) | 0.91 (0.42 to 1.62) | 2.59 (1.51 to 5.08) | 1.73 (1.37 to 2.1) |
| Nicaragua | Chronic lymphoid leukemia | DALYs | 0.009 (0.003 to 0.017) | 0.45 (0.16 to 0.88) | 0.029 (0.014 to 0.051) | 0.61 (0.29 to 1.07) | 2.33 (1.29 to 4.39) | 0.12 (-0.44 to 0.68) |
| Nicaragua | Other leukemia | DALYs | 0.046 (0.015 to 0.093) | 2.06 (0.72 to 4.08) | 0.13 (0.06 to 0.241) | 2.53 (1.17 to 4.65) | 1.85 (0.94 to 4.06) | 0.6 (0.38 to 0.81) |
| Niger | Leukemia | death | 0.002 (0.001 to 0.004) | 0.06 (0.02 to 0.13) | 0.009 (0.003 to 0.018) | 0.1 (0.04 to 0.21) | 3.6 (2.24 to 6.26) | 1.91 (1.81 to 2.01) |
| Niger | Acute myeloid leukemia | death | 0 (0 to 0.001) | 0.01 (0 to 0.02) | 0.002 (0 to 0.003) | 0.02 (0 to 0.04) | 3.89 (2.18 to 7.32) | 2.29 (2.19 to 2.39) |
| Niger | Acute lymphoid leukemia | death | 0 (0 to 0) | 0 (0 to 0) | 0 (0 to 0.001) | 0 (0 to 0.01) | 3.46 (1.61 to 7.16) | 1.61 (1.52 to 1.7) |
| Niger | Chronic myeloid leukemia | death | 0 (0 to 0.001) | 0.01 (0 to 0.02) | 0.001 (0 to 0.002) | 0.01 (0 to 0.02) | 2.68 (1.33 to 5.65) | 1.07 (0.97 to 1.16) |
| Niger | Chronic lymphoid leukemia | death | 0 (0 to 0.001) | 0.01 (0 to 0.03) | 0.002 (0.001 to 0.003) | 0.02 (0.01 to 0.05) | 4.22 (2.31 to 8.6) | 2.21 (2.11 to 2.32) |
| Niger | Other leukemia | death | 0.001 (0 to 0.002) | 0.03 (0.01 to 0.07) | 0.004 (0.001 to 0.009) | 0.05 (0.02 to 0.11) | 3.56 (2.12 to 6.45) | 1.85 (1.73 to 1.97) |
| Niger | Leukemia | DALYs | 0.067 (0.021 to 0.147) | 1.76 (0.55 to 3.79) | 0.299 (0.109 to 0.627) | 2.9 (1.06 to 6) | 3.49 (2.14 to 6.23) | 1.78 (1.68 to 1.89) |
| Niger | Acute myeloid leukemia | DALYs | 0.013 (0.004 to 0.03) | 0.3 (0.08 to 0.71) | 0.059 (0.019 to 0.131) | 0.52 (0.16 to 1.15) | 3.64 (2 to 7.11) | 2.04 (1.94 to 2.14) |
| Niger | Acute lymphoid leukemia | DALYs | 0.003 (0.001 to 0.008) | 0.07 (0.02 to 0.16) | 0.015 (0.004 to 0.037) | 0.11 (0.03 to 0.25) | 3.59 (1.56 to 7.78) | 1.68 (1.58 to 1.78) |
| Niger | Chronic myeloid leukemia | DALYs | 0.011 (0.003 to 0.025) | 0.24 (0.07 to 0.56) | 0.039 (0.013 to 0.087) | 0.33 (0.11 to 0.74) | 2.63 (1.27 to 5.7) | 0.97 (0.86 to 1.08) |
| Niger | Chronic lymphoid leukemia | DALYs | 0.008 (0.002 to 0.018) | 0.28 (0.08 to 0.62) | 0.042 (0.015 to 0.09) | 0.51 (0.18 to 1.06) | 4.14 (2.18 to 8.6) | 2.15 (2.04 to 2.25) |
| Niger | Other leukemia | DALYs | 0.032 (0.009 to 0.072) | 0.88 (0.26 to 1.97) | 0.144 (0.047 to 0.312) | 1.43 (0.48 to 3.11) | 3.53 (1.98 to 6.53) | 1.78 (1.65 to 1.91) |
| Nigeria | Leukemia | death | 0.036 (0.012 to 0.079) | 0.08 (0.03 to 0.17) | 0.143 (0.062 to 0.263) | 0.15 (0.07 to 0.28) | 2.96 (1.65 to 5.99) | 2.44 (2.35 to 2.52) |
| Nigeria | Acute myeloid leukemia | death | 0.003 (0.001 to 0.008) | 0.01 (0 to 0.01) | 0.017 (0.007 to 0.033) | 0.01 (0.01 to 0.03) | 4.03 (2.2 to 7.85) | 3.2 (3.01 to 3.4) |
| Nigeria | Acute lymphoid leukemia | death | 0.002 (0.001 to 0.004) | 0 (0 to 0.01) | 0.009 (0.004 to 0.017) | 0.01 (0 to 0.01) | 3.97 (2.08 to 8.79) | 2.74 (2.65 to 2.83) |
| Nigeria | Chronic myeloid leukemia | death | 0.005 (0.002 to 0.012) | 0.01 (0 to 0.02) | 0.025 (0.01 to 0.047) | 0.02 (0.01 to 0.04) | 3.51 (1.83 to 7.54) | 2.77 (2.67 to 2.87) |
| Nigeria | Chronic lymphoid leukemia | death | 0.008 (0.003 to 0.018) | 0.02 (0.01 to 0.04) | 0.036 (0.014 to 0.068) | 0.05 (0.02 to 0.09) | 3.32 (1.96 to 6.5) | 2.94 (2.86 to 3.03) |
| Nigeria | Other leukemia | death | 0.017 (0.005 to 0.038) | 0.04 (0.01 to 0.08) | 0.057 (0.025 to 0.108) | 0.06 (0.03 to 0.12) | 2.3 (1.19 to 5.1) | 1.86 (1.78 to 1.94) |
| Nigeria | Leukemia | DALYs | 1.138 (0.387 to 2.479) | 2.18 (0.74 to 4.76) | 4.78 (2.093 to 8.77) | 4.21 (1.83 to 7.72) | 3.2 (1.74 to 6.57) | 2.41 (2.32 to 2.5) |
| Nigeria | Acute myeloid leukemia | DALYs | 0.125 (0.039 to 0.295) | 0.21 (0.07 to 0.49) | 0.671 (0.265 to 1.368) | 0.5 (0.2 to 0.99) | 4.38 (2.38 to 8.99) | 3.15 (2.98 to 3.33) |
| Nigeria | Acute lymphoid leukemia | DALYs | 0.076 (0.023 to 0.174) | 0.12 (0.04 to 0.27) | 0.401 (0.169 to 0.784) | 0.26 (0.11 to 0.5) | 4.27 (2.18 to 9.76) | 2.76 (2.66 to 2.86) |
| Nigeria | Chronic myeloid leukemia | DALYs | 0.206 (0.066 to 0.46) | 0.36 (0.11 to 0.79) | 0.947 (0.392 to 1.849) | 0.72 (0.3 to 1.4) | 3.59 (1.75 to 8) | 2.55 (2.46 to 2.64) |
| Nigeria | Chronic lymphoid leukemia | DALYs | 0.195 (0.063 to 0.417) | 0.44 (0.14 to 0.94) | 0.897 (0.364 to 1.695) | 1.03 (0.42 to 1.94) | 3.59 (2.04 to 7.11) | 3.12 (3.02 to 3.21) |
| Nigeria | Other leukemia | DALYs | 0.536 (0.172 to 1.173) | 1.04 (0.33 to 2.3) | 1.864 (0.809 to 3.535) | 1.7 (0.73 to 3.22) | 2.48 (1.22 to 5.49) | 1.78 (1.7 to 1.86) |
| Niue | Leukemia | death | 0 (0 to 0) | 0.39 (0.18 to 0.72) | 0 (0 to 0) | 0.4 (0.2 to 0.7) | -0.02 (-0.31 to 0.4) | -0.16 (-0.27 to -0.05) |
| Niue | Acute myeloid leukemia | death | 0 (0 to 0) | 0.1 (0.04 to 0.18) | 0 (0 to 0) | 0.12 (0.06 to 0.21) | 0.1 (-0.26 to 0.69) | 0.31 (0.19 to 0.42) |
| Niue | Acute lymphoid leukemia | death | 0 (0 to 0) | 0.03 (0.01 to 0.05) | 0 (0 to 0) | 0.03 (0.01 to 0.05) | 0 (-0.35 to 0.6) | -0.14 (-0.31 to 0.03) |
| Niue | Chronic myeloid leukemia | death | 0 (0 to 0) | 0.06 (0.03 to 0.12) | 0 (0 to 0) | 0.05 (0.02 to 0.09) | -0.21 (-0.47 to 0.23) | -1.32 (-1.51 to -1.13) |
| Niue | Chronic lymphoid leukemia | death | 0 (0 to 0) | 0.01 (0 to 0.01) | 0 (0 to 0) | 0.01 (0 to 0.02) | 0.16 (-0.21 to 0.87) | 0.28 (0.18 to 0.39) |
| Niue | Other leukemia | death | 0 (0 to 0) | 0.2 (0.09 to 0.37) | 0 (0 to 0) | 0.2 (0.09 to 0.36) | -0.04 (-0.32 to 0.39) | -0.12 (-0.21 to -0.02) |
| Niue | Leukemia | DALYs | 0 (0 to 0) | 12.58 (5.77 to 22.03) | 0 (0 to 0) | 12.41 (6.13 to 21.93) | -0.06 (-0.37 to 0.42) | -0.35 (-0.48 to -0.22) |
| Niue | Acute myeloid leukemia | DALYs | 0 (0 to 0) | 3.74 (1.69 to 6.76) | 0 (0 to 0) | 4.2 (2.01 to 7.66) | 0.04 (-0.34 to 0.67) | 0.18 (0.06 to 0.31) |
| Niue | Acute lymphoid leukemia | DALYs | 0 (0 to 0) | 1.04 (0.45 to 1.92) | 0 (0 to 0) | 1.08 (0.49 to 1.95) | -0.06 (-0.43 to 0.57) | -0.25 (-0.42 to -0.08) |
| Niue | Chronic myeloid leukemia | DALYs | 0 (0 to 0) | 1.91 (0.82 to 3.58) | 0 (0 to 0) | 1.44 (0.65 to 2.77) | -0.25 (-0.52 to 0.17) | -1.57 (-1.78 to -1.36) |
| Niue | Chronic lymphoid leukemia | DALYs | 0 (0 to 0) | 0.22 (0.09 to 0.41) | 0 (0 to 0) | 0.26 (0.12 to 0.46) | 0.19 (-0.21 to 0.89) | 0.28 (0.17 to 0.4) |
| Niue | Other leukemia | DALYs | 0 (0 to 0) | 5.67 (2.52 to 10.18) | 0 (0 to 0) | 5.43 (2.51 to 9.71) | -0.08 (-0.38 to 0.39) | -0.4 (-0.52 to -0.28) |
| North Macedonia | Leukemia | death | 0.006 (0.003 to 0.011) | 0.33 (0.15 to 0.56) | 0.014 (0.007 to 0.023) | 0.44 (0.22 to 0.75) | 1.21 (0.68 to 1.99) | 1.1 (0.93 to 1.27) |
| North Macedonia | Acute myeloid leukemia | death | 0.001 (0.001 to 0.002) | 0.07 (0.03 to 0.12) | 0.002 (0.001 to 0.004) | 0.08 (0.04 to 0.13) | 0.82 (0.29 to 1.56) | 0.38 (0.33 to 0.44) |
| North Macedonia | Acute lymphoid leukemia | death | 0 (0 to 0.001) | 0.02 (0.01 to 0.04) | 0.001 (0 to 0.001) | 0.02 (0.01 to 0.04) | 0.69 (0.17 to 1.6) | 0.29 (0.15 to 0.44) |
| North Macedonia | Chronic myeloid leukemia | death | 0.001 (0 to 0.001) | 0.03 (0.01 to 0.05) | 0.001 (0 to 0.001) | 0.02 (0.01 to 0.04) | 0.25 (-0.19 to 1.01) | -1.39 (-1.67 to -1.1) |
| North Macedonia | Chronic lymphoid leukemia | death | 0.001 (0 to 0.002) | 0.05 (0.02 to 0.09) | 0.005 (0.002 to 0.008) | 0.15 (0.08 to 0.26) | 4.36 (2.76 to 6.71) | 4.08 (3.54 to 4.64) |
| North Macedonia | Other leukemia | death | 0.003 (0.001 to 0.005) | 0.16 (0.07 to 0.28) | 0.005 (0.002 to 0.009) | 0.17 (0.08 to 0.29) | 0.69 (0.2 to 1.41) | 0.19 (0.07 to 0.32) |
| North Macedonia | Leukemia | DALYs | 0.19 (0.088 to 0.328) | 9.4 (4.33 to 16.16) | 0.377 (0.189 to 0.653) | 12.16 (6.1 to 21.12) | 0.98 (0.48 to 1.71) | 0.88 (0.71 to 1.05) |
| North Macedonia | Acute myeloid leukemia | DALYs | 0.042 (0.018 to 0.075) | 2.03 (0.89 to 3.65) | 0.071 (0.033 to 0.125) | 2.36 (1.09 to 4.2) | 0.71 (0.22 to 1.42) | 0.44 (0.37 to 0.51) |
| North Macedonia | Acute lymphoid leukemia | DALYs | 0.014 (0.004 to 0.027) | 0.66 (0.21 to 1.28) | 0.022 (0.008 to 0.041) | 0.8 (0.3 to 1.49) | 0.64 (0.14 to 1.52) | 0.52 (0.37 to 0.68) |
| North Macedonia | Chronic myeloid leukemia | DALYs | 0.017 (0.007 to 0.032) | 0.82 (0.34 to 1.6) | 0.019 (0.008 to 0.036) | 0.62 (0.27 to 1.21) | 0.15 (-0.26 to 0.84) | -1.54 (-1.86 to -1.22) |
| North Macedonia | Chronic lymphoid leukemia | DALYs | 0.022 (0.01 to 0.039) | 1.14 (0.53 to 2.04) | 0.114 (0.057 to 0.194) | 3.42 (1.71 to 5.8) | 4.18 (2.57 to 6.45) | 3.96 (3.4 to 4.52) |
| North Macedonia | Other leukemia | DALYs | 0.096 (0.044 to 0.168) | 4.75 (2.17 to 8.41) | 0.15 (0.068 to 0.271) | 4.95 (2.22 to 8.97) | 0.56 (0.11 to 1.23) | 0.16 (0.03 to 0.29) |
| Northern Mariana Islands | Leukemia | death | 0 (0 to 0) | 0.77 (0.39 to 1.24) | 0 (0 to 0) | 0.46 (0.23 to 0.73) | 0.11 (-0.22 to 0.74) | -2.34 (-2.58 to -2.1) |
| Northern Mariana Islands | Acute myeloid leukemia | death | 0 (0 to 0) | 0.27 (0.13 to 0.45) | 0 (0 to 0) | 0.12 (0.06 to 0.22) | -0.26 (-0.56 to 0.87) | -3.64 (-4.02 to -3.26) |
| Northern Mariana Islands | Acute lymphoid leukemia | death | 0 (0 to 0) | 0.06 (0.02 to 0.11) | 0 (0 to 0) | 0.03 (0.01 to 0.06) | -0.34 (-0.66 to 1.01) | -3.18 (-3.9 to -2.44) |
| Northern Mariana Islands | Chronic myeloid leukemia | death | 0 (0 to 0) | 0.14 (0.06 to 0.25) | 0 (0 to 0) | 0.05 (0.02 to 0.08) | -0.23 (-0.5 to 0.24) | -4.41 (-4.96 to -3.85) |
| Northern Mariana Islands | Chronic lymphoid leukemia | death | 0 (0 to 0) | 0.01 (0 to 0.02) | 0 (0 to 0) | 0.01 (0 to 0.01) | 0.82 (0.2 to 1.8) | -2.73 (-3.21 to -2.23) |
| Northern Mariana Islands | Other leukemia | death | 0 (0 to 0) | 0.29 (0.14 to 0.49) | 0 (0 to 0) | 0.26 (0.13 to 0.43) | 0.99 (0.32 to 2.02) | -0.64 (-0.83 to -0.45) |
| Northern Mariana Islands | Leukemia | DALYs | 0.009 (0.004 to 0.015) | 23.74 (11.73 to 37.73) | 0.007 (0.004 to 0.012) | 13.67 (6.92 to 21.78) | -0.18 (-0.44 to 0.32) | -2.46 (-2.71 to -2.2) |
| Northern Mariana Islands | Acute myeloid leukemia | DALYs | 0.004 (0.002 to 0.007) | 9.38 (4.35 to 15.53) | 0.002 (0.001 to 0.004) | 4.3 (2.08 to 7.68) | -0.43 (-0.67 to 0.4) | -3.51 (-3.86 to -3.16) |
| Northern Mariana Islands | Acute lymphoid leukemia | DALYs | 0.001 (0 to 0.002) | 2.37 (0.81 to 4.69) | 0.001 (0 to 0.001) | 1.04 (0.47 to 2.04) | -0.52 (-0.76 to 0.59) | -3.5 (-4.18 to -2.8) |
| Northern Mariana Islands | Chronic myeloid leukemia | DALYs | 0.001 (0.001 to 0.002) | 4.12 (1.78 to 7.3) | 0.001 (0 to 0.001) | 1.36 (0.6 to 2.37) | -0.38 (-0.61 to 0.05) | -4.34 (-4.9 to -3.78) |
| Northern Mariana Islands | Chronic lymphoid leukemia | DALYs | 0 (0 to 0) | 0.26 (0.13 to 0.46) | 0 (0 to 0) | 0.19 (0.09 to 0.34) | 0.58 (0.02 to 1.48) | -1.97 (-2.4 to -1.54) |
| Northern Mariana Islands | Other leukemia | DALYs | 0.002 (0.001 to 0.004) | 7.61 (3.57 to 12.82) | 0.004 (0.002 to 0.006) | 6.78 (3.42 to 11.2) | 0.46 (-0.09 to 1.4) | -0.57 (-0.76 to -0.38) |
| Norway | Leukemia | death | 0.013 (0.006 to 0.024) | 0.2 (0.09 to 0.36) | 0.02 (0.009 to 0.034) | 0.2 (0.09 to 0.35) | 0.47 (0.29 to 0.77) | 0.22 (0.17 to 0.28) |
| Norway | Acute myeloid leukemia | death | 0.005 (0.002 to 0.009) | 0.08 (0.04 to 0.15) | 0.009 (0.004 to 0.016) | 0.1 (0.05 to 0.18) | 0.77 (0.54 to 1.16) | 0.97 (0.86 to 1.08) |
| Norway | Acute lymphoid leukemia | death | 0.001 (0 to 0.001) | 0.01 (0 to 0.02) | 0.001 (0 to 0.001) | 0.01 (0 to 0.02) | 0.36 (0.16 to 0.68) | -0.21 (-0.3 to -0.12) |
| Norway | Chronic myeloid leukemia | death | 0.001 (0.001 to 0.002) | 0.02 (0.01 to 0.04) | 0.001 (0 to 0.001) | 0.01 (0 to 0.01) | -0.52 (-0.58 to -0.4) | -4.18 (-4.35 to -4.02) |
| Norway | Chronic lymphoid leukemia | death | 0.004 (0.002 to 0.008) | 0.06 (0.03 to 0.11) | 0.006 (0.003 to 0.01) | 0.06 (0.02 to 0.1) | 0.37 (0.15 to 0.68) | -0.16 (-0.37 to 0.06) |
| Norway | Other leukemia | death | 0.002 (0.001 to 0.003) | 0.03 (0.01 to 0.05) | 0.003 (0.001 to 0.005) | 0.03 (0.01 to 0.05) | 0.55 (0.28 to 0.89) | 0.62 (0.45 to 0.78) |
| Norway | Leukemia | DALYs | 0.315 (0.145 to 0.558) | 5.43 (2.49 to 9.67) | 0.418 (0.195 to 0.719) | 4.97 (2.34 to 8.52) | 0.33 (0.18 to 0.6) | -0.14 (-0.2 to -0.08) |
| Norway | Acute myeloid leukemia | DALYs | 0.136 (0.062 to 0.24) | 2.44 (1.12 to 4.3) | 0.214 (0.099 to 0.366) | 2.64 (1.22 to 4.51) | 0.57 (0.38 to 0.91) | 0.49 (0.41 to 0.58) |
| Norway | Acute lymphoid leukemia | DALYs | 0.02 (0.009 to 0.037) | 0.41 (0.19 to 0.75) | 0.027 (0.013 to 0.047) | 0.4 (0.19 to 0.68) | 0.35 (0.14 to 0.7) | -0.09 (-0.2 to 0.01) |
| Norway | Chronic myeloid leukemia | DALYs | 0.034 (0.016 to 0.061) | 0.63 (0.29 to 1.12) | 0.016 (0.007 to 0.027) | 0.2 (0.09 to 0.34) | -0.54 (-0.6 to -0.43) | -4.3 (-4.45 to -4.15) |
| Norway | Chronic lymphoid leukemia | DALYs | 0.084 (0.035 to 0.152) | 1.27 (0.54 to 2.31) | 0.107 (0.048 to 0.189) | 1.11 (0.5 to 1.96) | 0.28 (0.08 to 0.6) | -0.37 (-0.65 to -0.09) |
| Norway | Other leukemia | DALYs | 0.041 (0.019 to 0.073) | 0.67 (0.31 to 1.2) | 0.055 (0.026 to 0.095) | 0.62 (0.3 to 1.07) | 0.34 (0.12 to 0.65) | 0.11 (-0.02 to 0.23) |
| Oman | Leukemia | death | 0.002 (0.001 to 0.003) | 0.24 (0.1 to 0.45) | 0.009 (0.005 to 0.015) | 0.49 (0.26 to 0.79) | 4.03 (2.3 to 7.85) | 2.83 (2.4 to 3.27) |
| Oman | Acute myeloid leukemia | death | 0 (0 to 0.001) | 0.05 (0.02 to 0.09) | 0.003 (0.002 to 0.005) | 0.13 (0.07 to 0.21) | 6.56 (3.77 to 12.36) | 3.62 (3.11 to 4.12) |
| Oman | Acute lymphoid leukemia | death | 0 (0 to 0) | 0.02 (0.01 to 0.03) | 0.001 (0.001 to 0.002) | 0.04 (0.02 to 0.06) | 5.58 (2.87 to 11.89) | 2.99 (2.45 to 3.53) |
| Oman | Chronic myeloid leukemia | death | 0 (0 to 0.001) | 0.04 (0.02 to 0.08) | 0.001 (0.001 to 0.002) | 0.06 (0.03 to 0.1) | 1.96 (0.73 to 4.72) | 1.27 (0.78 to 1.75) |
| Oman | Chronic lymphoid leukemia | death | 0 (0 to 0) | 0.01 (0 to 0.02) | 0 (0 to 0.001) | 0.03 (0.01 to 0.06) | 6.2 (3.43 to 12.73) | 4.53 (3.9 to 5.16) |
| Oman | Other leukemia | death | 0.001 (0 to 0.002) | 0.12 (0.04 to 0.24) | 0.003 (0.002 to 0.006) | 0.23 (0.12 to 0.42) | 3.06 (1.59 to 8.04) | 2.71 (2.33 to 3.09) |
| Oman | Leukemia | DALYs | 0.066 (0.03 to 0.121) | 6.62 (2.92 to 12.2) | 0.344 (0.181 to 0.566) | 11.82 (6.31 to 18.93) | 4.18 (2.32 to 8.3) | 2.28 (1.75 to 2.81) |
| Oman | Acute myeloid leukemia | DALYs | 0.017 (0.007 to 0.033) | 1.52 (0.64 to 2.94) | 0.136 (0.072 to 0.225) | 3.76 (2.02 to 6.09) | 6.89 (3.86 to 12.99) | 3.32 (2.77 to 3.88) |
| Oman | Acute lymphoid leukemia | DALYs | 0.008 (0.003 to 0.015) | 0.57 (0.2 to 1.16) | 0.054 (0.025 to 0.097) | 1.25 (0.58 to 2.21) | 6.14 (3.08 to 13.32) | 2.85 (2.25 to 3.45) |
| Oman | Chronic myeloid leukemia | DALYs | 0.013 (0.005 to 0.025) | 1.24 (0.49 to 2.39) | 0.038 (0.019 to 0.067) | 1.32 (0.63 to 2.27) | 1.9 (0.63 to 4.96) | 0.47 (-0.15 to 1.09) |
| Oman | Chronic lymphoid leukemia | DALYs | 0.002 (0.001 to 0.004) | 0.25 (0.08 to 0.53) | 0.015 (0.007 to 0.027) | 0.72 (0.31 to 1.32) | 6.21 (3.36 to 12.76) | 3.98 (3.22 to 4.74) |
| Oman | Other leukemia | DALYs | 0.027 (0.01 to 0.053) | 3.04 (1.11 to 5.93) | 0.102 (0.051 to 0.183) | 4.76 (2.42 to 8.44) | 2.83 (1.44 to 7.77) | 1.92 (1.43 to 2.41) |
| Pakistan | Leukemia | death | 0.042 (0.009 to 0.102) | 0.07 (0.01 to 0.16) | 0.197 (0.079 to 0.379) | 0.16 (0.07 to 0.3) | 3.73 (2.15 to 9.31) | 3.16 (2.83 to 3.5) |
| Pakistan | Acute myeloid leukemia | death | 0.008 (0.002 to 0.02) | 0.01 (0 to 0.03) | 0.046 (0.019 to 0.087) | 0.03 (0.01 to 0.06) | 4.88 (2.77 to 12.19) | 3.88 (3.62 to 4.13) |
| Pakistan | Acute lymphoid leukemia | death | 0.003 (0.001 to 0.007) | 0 (0 to 0.01) | 0.016 (0.006 to 0.031) | 0.01 (0 to 0.02) | 4.37 (2.58 to 10.66) | 3.27 (2.99 to 3.55) |
| Pakistan | Chronic myeloid leukemia | death | 0.009 (0.002 to 0.023) | 0.01 (0 to 0.04) | 0.037 (0.015 to 0.072) | 0.03 (0.01 to 0.05) | 2.91 (1.44 to 7.7) | 2.23 (1.86 to 2.6) |
| Pakistan | Chronic lymphoid leukemia | death | 0.013 (0.003 to 0.033) | 0.02 (0.01 to 0.06) | 0.069 (0.024 to 0.136) | 0.07 (0.02 to 0.13) | 4.19 (2.34 to 10.99) | 3.75 (3.36 to 4.14) |
| Pakistan | Other leukemia | death | 0.008 (0.002 to 0.022) | 0.01 (0 to 0.04) | 0.03 (0.012 to 0.06) | 0.02 (0.01 to 0.05) | 2.6 (1.21 to 7.84) | 2.15 (1.85 to 2.45) |
| Pakistan | Leukemia | DALYs | 1.404 (0.302 to 3.45) | 2.02 (0.43 to 4.94) | 7.167 (2.745 to 13.954) | 4.76 (1.9 to 9.14) | 4.1 (2.4 to 9.91) | 3.16 (2.83 to 3.49) |
| Pakistan | Acute myeloid leukemia | DALYs | 0.303 (0.062 to 0.759) | 0.4 (0.08 to 0.99) | 1.919 (0.741 to 3.705) | 1.15 (0.46 to 2.15) | 5.34 (3.19 to 13.09) | 3.96 (3.69 to 4.22) |
| Pakistan | Acute lymphoid leukemia | DALYs | 0.135 (0.028 to 0.336) | 0.16 (0.03 to 0.4) | 0.764 (0.282 to 1.55) | 0.4 (0.15 to 0.79) | 4.64 (2.76 to 11.51) | 3.33 (3.04 to 3.63) |
| Pakistan | Chronic myeloid leukemia | DALYs | 0.339 (0.073 to 0.817) | 0.48 (0.1 to 1.17) | 1.428 (0.574 to 2.832) | 0.9 (0.36 to 1.77) | 3.21 (1.73 to 8.04) | 2.26 (1.88 to 2.64) |
| Pakistan | Chronic lymphoid leukemia | DALYs | 0.347 (0.073 to 0.865) | 0.58 (0.12 to 1.45) | 1.943 (0.689 to 3.779) | 1.59 (0.56 to 3.15) | 4.6 (2.55 to 11.76) | 3.72 (3.35 to 4.1) |
| Pakistan | Other leukemia | DALYs | 0.28 (0.054 to 0.739) | 0.4 (0.08 to 1.05) | 1.113 (0.419 to 2.248) | 0.72 (0.28 to 1.45) | 2.98 (1.46 to 8.58) | 2.22 (1.9 to 2.54) |
| Palau | Leukemia | death | 0 (0 to 0) | 0.22 (0.1 to 0.38) | 0 (0 to 0) | 0.23 (0.11 to 0.38) | 1.1 (0.45 to 2.02) | 0.07 (-0.01 to 0.14) |
| Palau | Acute myeloid leukemia | death | 0 (0 to 0) | 0.06 (0.03 to 0.1) | 0 (0 to 0) | 0.06 (0.03 to 0.11) | 1.28 (0.53 to 2.49) | 0.32 (0.22 to 0.42) |
| Palau | Acute lymphoid leukemia | death | 0 (0 to 0) | 0.02 (0.01 to 0.04) | 0 (0 to 0) | 0.03 (0.01 to 0.04) | 1.21 (0.47 to 2.31) | 0.39 (0.28 to 0.5) |
| Palau | Chronic myeloid leukemia | death | 0 (0 to 0) | 0.02 (0.01 to 0.03) | 0 (0 to 0) | 0.02 (0.01 to 0.03) | 1.05 (0.33 to 2.22) | -0.14 (-0.23 to -0.05) |
| Palau | Chronic lymphoid leukemia | death | 0 (0 to 0) | 0.01 (0 to 0.01) | 0 (0 to 0) | 0.01 (0 to 0.02) | 1.47 (0.58 to 2.85) | 0.39 (0.3 to 0.48) |
| Palau | Other leukemia | death | 0 (0 to 0) | 0.11 (0.05 to 0.2) | 0 (0 to 0) | 0.11 (0.05 to 0.19) | 0.94 (0.33 to 1.89) | -0.13 (-0.19 to -0.06) |
| Palau | Leukemia | DALYs | 0.001 (0 to 0.001) | 6.43 (2.97 to 10.94) | 0.002 (0.001 to 0.003) | 6.83 (3.41 to 11.04) | 1 (0.36 to 1.91) | 0.11 (0.04 to 0.19) |
| Palau | Acute myeloid leukemia | DALYs | 0 (0 to 0) | 2.01 (0.96 to 3.52) | 0.001 (0 to 0.001) | 2.32 (1.17 to 3.87) | 1.11 (0.4 to 2.24) | 0.36 (0.26 to 0.45) |
| Palau | Acute lymphoid leukemia | DALYs | 0 (0 to 0) | 0.79 (0.36 to 1.41) | 0 (0 to 0) | 0.95 (0.47 to 1.58) | 1.04 (0.33 to 2.09) | 0.54 (0.42 to 0.66) |
| Palau | Chronic myeloid leukemia | DALYs | 0 (0 to 0) | 0.61 (0.28 to 1.07) | 0 (0 to 0) | 0.61 (0.29 to 0.99) | 0.93 (0.24 to 2.03) | -0.11 (-0.19 to -0.02) |
| Palau | Chronic lymphoid leukemia | DALYs | 0 (0 to 0) | 0.22 (0.1 to 0.42) | 0 (0 to 0) | 0.26 (0.12 to 0.45) | 1.37 (0.52 to 2.65) | 0.38 (0.3 to 0.47) |
| Palau | Other leukemia | DALYs | 0 (0 to 0.001) | 2.8 (1.25 to 4.91) | 0.001 (0 to 0.001) | 2.69 (1.29 to 4.47) | 0.88 (0.26 to 1.87) | -0.19 (-0.24 to -0.15) |
| Palestine | Leukemia | death | 0.005 (0.002 to 0.009) | 0.51 (0.2 to 0.98) | 0.014 (0.007 to 0.024) | 0.57 (0.26 to 0.99) | 1.99 (1.16 to 3.56) | 0.04 (-0.23 to 0.31) |
| Palestine | Acute myeloid leukemia | death | 0 (0 to 0) | 0.02 (0.01 to 0.04) | 0.001 (0.001 to 0.002) | 0.04 (0.02 to 0.06) | 4.93 (2.65 to 9.31) | 1.85 (1.54 to 2.15) |
| Palestine | Acute lymphoid leukemia | death | 0 (0 to 0) | 0.01 (0 to 0.02) | 0 (0 to 0.001) | 0.01 (0.01 to 0.02) | 4.89 (2.57 to 9.87) | 1.93 (1.64 to 2.22) |
| Palestine | Chronic myeloid leukemia | death | 0.001 (0 to 0.001) | 0.05 (0.01 to 0.12) | 0.002 (0.001 to 0.003) | 0.06 (0.02 to 0.11) | 1.95 (0.94 to 4.85) | -0.13 (-0.47 to 0.22) |
| Palestine | Chronic lymphoid leukemia | death | 0 (0 to 0) | 0.01 (0 to 0.03) | 0.001 (0 to 0.001) | 0.03 (0.01 to 0.05) | 4.01 (2.12 to 8.44) | 1.93 (1.41 to 2.46) |
| Palestine | Other leukemia | death | 0.004 (0.001 to 0.007) | 0.42 (0.16 to 0.83) | 0.01 (0.005 to 0.017) | 0.43 (0.2 to 0.76) | 1.72 (0.92 to 3.36) | -0.17 (-0.41 to 0.07) |
| Palestine | Leukemia | DALYs | 0.146 (0.057 to 0.273) | 14.31 (5.63 to 26.52) | 0.454 (0.216 to 0.768) | 14.87 (7.17 to 25.12) | 2.11 (1.21 to 3.71) | -0.19 (-0.46 to 0.08) |
| Palestine | Acute myeloid leukemia | DALYs | 0.008 (0.003 to 0.016) | 0.67 (0.25 to 1.37) | 0.048 (0.022 to 0.083) | 1.27 (0.59 to 2.19) | 5.23 (2.76 to 10.06) | 1.86 (1.54 to 2.17) |
| Palestine | Acute lymphoid leukemia | DALYs | 0.003 (0.001 to 0.007) | 0.27 (0.1 to 0.57) | 0.021 (0.009 to 0.037) | 0.53 (0.24 to 0.92) | 5.33 (2.79 to 11.06) | 2.09 (1.76 to 2.41) |
| Palestine | Chronic myeloid leukemia | DALYs | 0.017 (0.005 to 0.037) | 1.65 (0.44 to 3.59) | 0.051 (0.021 to 0.094) | 1.61 (0.64 to 3.04) | 1.99 (0.93 to 5.32) | -0.45 (-0.81 to -0.1) |
| Palestine | Chronic lymphoid leukemia | DALYs | 0.004 (0.001 to 0.008) | 0.37 (0.13 to 0.8) | 0.018 (0.007 to 0.033) | 0.65 (0.27 to 1.23) | 3.99 (2.1 to 8.39) | 1.57 (1.06 to 2.09) |
| Palestine | Other leukemia | DALYs | 0.114 (0.045 to 0.22) | 11.35 (4.44 to 21.64) | 0.317 (0.154 to 0.541) | 10.81 (5.19 to 18.42) | 1.77 (0.92 to 3.52) | -0.47 (-0.71 to -0.23) |
| Panama | Leukemia | death | 0.002 (0.001 to 0.005) | 0.14 (0.04 to 0.31) | 0.012 (0.006 to 0.022) | 0.29 (0.13 to 0.53) | 4.14 (2.36 to 9.34) | 2.55 (2.44 to 2.65) |
| Panama | Acute myeloid leukemia | death | 0.001 (0 to 0.001) | 0.04 (0.01 to 0.08) | 0.004 (0.002 to 0.007) | 0.1 (0.04 to 0.17) | 5.3 (2.96 to 11.86) | 3.21 (3.09 to 3.33) |
| Panama | Acute lymphoid leukemia | death | 0 (0 to 0.001) | 0.03 (0.01 to 0.05) | 0.002 (0.001 to 0.005) | 0.06 (0.03 to 0.11) | 4.12 (2.19 to 9.69) | 3.01 (2.84 to 3.18) |
| Panama | Chronic myeloid leukemia | death | 0 (0 to 0.001) | 0.02 (0.01 to 0.04) | 0.001 (0.001 to 0.002) | 0.03 (0.01 to 0.05) | 2.64 (1.29 to 6.42) | 1.25 (1.07 to 1.43) |
| Panama | Chronic lymphoid leukemia | death | 0 (0 to 0) | 0.01 (0 to 0.03) | 0.001 (0 to 0.002) | 0.02 (0.01 to 0.04) | 4.56 (2.45 to 10.62) | 1.88 (1.43 to 2.34) |
| Panama | Other leukemia | death | 0.001 (0 to 0.002) | 0.05 (0.01 to 0.1) | 0.004 (0.002 to 0.007) | 0.09 (0.04 to 0.17) | 3.73 (2.04 to 8.53) | 2.29 (2.11 to 2.47) |
| Panama | Leukemia | DALYs | 0.085 (0.022 to 0.18) | 4.51 (1.23 to 9.47) | 0.387 (0.178 to 0.692) | 9.22 (4.25 to 16.48) | 3.56 (1.93 to 8.28) | 2.61 (2.5 to 2.71) |
| Panama | Acute myeloid leukemia | DALYs | 0.023 (0.006 to 0.048) | 1.22 (0.34 to 2.55) | 0.127 (0.056 to 0.225) | 3.03 (1.34 to 5.37) | 4.61 (2.47 to 10.92) | 3.19 (3.07 to 3.31) |
| Panama | Acute lymphoid leukemia | DALYs | 0.021 (0.005 to 0.047) | 1.01 (0.27 to 2.22) | 0.098 (0.043 to 0.185) | 2.31 (1.02 to 4.37) | 3.62 (1.84 to 9.11) | 3.04 (2.87 to 3.2) |
| Panama | Chronic myeloid leukemia | DALYs | 0.012 (0.003 to 0.025) | 0.62 (0.17 to 1.29) | 0.038 (0.017 to 0.068) | 0.9 (0.41 to 1.62) | 2.27 (1.03 to 6.02) | 1.31 (1.13 to 1.51) |
| Panama | Chronic lymphoid leukemia | DALYs | 0.004 (0.001 to 0.008) | 0.25 (0.07 to 0.54) | 0.019 (0.009 to 0.036) | 0.47 (0.21 to 0.87) | 4.13 (2.2 to 9.67) | 1.91 (1.44 to 2.39) |
| Panama | Other leukemia | DALYs | 0.026 (0.007 to 0.055) | 1.42 (0.4 to 2.97) | 0.106 (0.047 to 0.193) | 2.52 (1.12 to 4.61) | 3.1 (1.55 to 7.35) | 2.31 (2.11 to 2.52) |
| Papua New Guinea | Leukemia | death | 0.003 (0.001 to 0.006) | 0.12 (0.03 to 0.28) | 0.009 (0.003 to 0.019) | 0.15 (0.04 to 0.32) | 2.09 (1.23 to 3.6) | 0.22 (0.03 to 0.41) |
| Papua New Guinea | Acute myeloid leukemia | death | 0.001 (0 to 0.001) | 0.02 (0.01 to 0.06) | 0.002 (0.001 to 0.005) | 0.03 (0.01 to 0.07) | 2.34 (1.31 to 4.34) | 0.21 (0 to 0.43) |
| Papua New Guinea | Acute lymphoid leukemia | death | 0 (0 to 0) | 0.01 (0 to 0.02) | 0.001 (0 to 0.001) | 0.01 (0 to 0.02) | 2.42 (1.29 to 4.6) | 0.56 (0.33 to 0.79) |
| Papua New Guinea | Chronic myeloid leukemia | death | 0 (0 to 0.001) | 0.02 (0 to 0.04) | 0.001 (0 to 0.003) | 0.02 (0.01 to 0.04) | 1.55 (0.69 to 3.22) | -0.57 (-0.82 to -0.32) |
| Papua New Guinea | Chronic lymphoid leukemia | death | 0 (0 to 0) | 0 (0 to 0) | 0 (0 to 0) | 0 (0 to 0) | 2.15 (1.07 to 4.14) | 0.13 (-0.04 to 0.31) |
| Papua New Guinea | Other leukemia | death | 0.002 (0 to 0.004) | 0.07 (0.02 to 0.18) | 0.005 (0.001 to 0.011) | 0.09 (0.02 to 0.2) | 2.11 (1.24 to 3.59) | 0.37 (0.2 to 0.55) |
| Papua New Guinea | Leukemia | DALYs | 0.112 (0.031 to 0.244) | 4.11 (1.16 to 8.92) | 0.351 (0.113 to 0.77) | 4.78 (1.51 to 10.38) | 2.12 (1.22 to 3.76) | 0.2 (-0.01 to 0.41) |
| Papua New Guinea | Acute myeloid leukemia | DALYs | 0.027 (0.007 to 0.063) | 0.91 (0.24 to 2.17) | 0.091 (0.029 to 0.204) | 1.13 (0.35 to 2.49) | 2.39 (1.3 to 4.5) | 0.25 (0.03 to 0.47) |
| Papua New Guinea | Acute lymphoid leukemia | DALYs | 0.008 (0.002 to 0.021) | 0.26 (0.07 to 0.63) | 0.027 (0.008 to 0.067) | 0.33 (0.1 to 0.77) | 2.37 (1.21 to 4.7) | 0.49 (0.26 to 0.72) |
| Papua New Guinea | Chronic myeloid leukemia | DALYs | 0.017 (0.004 to 0.039) | 0.63 (0.16 to 1.43) | 0.043 (0.013 to 0.098) | 0.6 (0.18 to 1.36) | 1.58 (0.68 to 3.42) | -0.56 (-0.83 to -0.3) |
| Papua New Guinea | Chronic lymphoid leukemia | DALYs | 0.001 (0 to 0.002) | 0.04 (0.01 to 0.1) | 0.003 (0.001 to 0.008) | 0.05 (0.01 to 0.12) | 2.29 (1.17 to 4.28) | 0.28 (0.1 to 0.46) |
| Papua New Guinea | Other leukemia | DALYs | 0.06 (0.015 to 0.138) | 2.26 (0.56 to 5.24) | 0.186 (0.056 to 0.422) | 2.66 (0.8 to 6.08) | 2.12 (1.23 to 3.7) | 0.33 (0.14 to 0.53) |
| Paraguay | Leukemia | death | 0.005 (0.002 to 0.009) | 0.21 (0.09 to 0.39) | 0.02 (0.009 to 0.035) | 0.34 (0.16 to 0.6) | 2.9 (1.68 to 4.81) | 1.78 (1.57 to 1.98) |
| Paraguay | Acute myeloid leukemia | death | 0.001 (0 to 0.002) | 0.03 (0.01 to 0.07) | 0.006 (0.003 to 0.011) | 0.1 (0.05 to 0.18) | 5.84 (2.99 to 9.97) | 4.45 (4.11 to 4.78) |
| Paraguay | Acute lymphoid leukemia | death | 0 (0 to 0.001) | 0.02 (0.01 to 0.03) | 0.003 (0.001 to 0.005) | 0.04 (0.02 to 0.08) | 4.51 (1.93 to 8.3) | 3.64 (3.33 to 3.95) |
| Paraguay | Chronic myeloid leukemia | death | 0 (0 to 0.001) | 0.02 (0.01 to 0.03) | 0.002 (0.001 to 0.003) | 0.03 (0.01 to 0.05) | 3.27 (1.79 to 5.63) | 2.44 (2.01 to 2.86) |
| Paraguay | Chronic lymphoid leukemia | death | 0 (0 to 0) | 0.01 (0 to 0.02) | 0.002 (0.001 to 0.004) | 0.04 (0.02 to 0.07) | 6.8 (4.42 to 10.8) | 4.54 (4.15 to 4.92) |
| Paraguay | Other leukemia | death | 0.003 (0.001 to 0.006) | 0.13 (0.05 to 0.24) | 0.007 (0.003 to 0.014) | 0.13 (0.06 to 0.24) | 1.4 (0.55 to 3.08) | -0.39 (-0.7 to -0.08) |
| Paraguay | Leukemia | DALYs | 0.172 (0.069 to 0.324) | 6.24 (2.57 to 11.69) | 0.624 (0.301 to 1.096) | 9.87 (4.73 to 17.46) | 2.64 (1.42 to 4.72) | 1.58 (1.38 to 1.79) |
| Paraguay | Acute myeloid leukemia | DALYs | 0.032 (0.012 to 0.061) | 1.13 (0.46 to 2.16) | 0.203 (0.097 to 0.36) | 3.16 (1.52 to 5.64) | 5.39 (2.81 to 9.59) | 4.22 (3.9 to 4.55) |
| Paraguay | Acute lymphoid leukemia | DALYs | 0.021 (0.008 to 0.043) | 0.69 (0.27 to 1.38) | 0.114 (0.047 to 0.212) | 1.66 (0.68 to 3.12) | 4.32 (1.86 to 8.55) | 3.57 (3.28 to 3.86) |
| Paraguay | Chronic myeloid leukemia | DALYs | 0.014 (0.006 to 0.025) | 0.51 (0.22 to 0.96) | 0.054 (0.025 to 0.096) | 0.87 (0.41 to 1.55) | 2.96 (1.46 to 5.29) | 2.12 (1.7 to 2.55) |
| Paraguay | Chronic lymphoid leukemia | DALYs | 0.006 (0.003 to 0.011) | 0.26 (0.11 to 0.47) | 0.043 (0.021 to 0.078) | 0.79 (0.37 to 1.42) | 6.44 (4.03 to 10.42) | 4.33 (3.95 to 4.72) |
| Paraguay | Other leukemia | DALYs | 0.099 (0.039 to 0.192) | 3.64 (1.48 to 6.93) | 0.21 (0.095 to 0.406) | 3.39 (1.53 to 6.48) | 1.12 (0.3 to 2.88) | -0.81 (-1.14 to -0.48) |
| Peru | Leukemia | death | 0.027 (0.01 to 0.052) | 0.2 (0.08 to 0.39) | 0.111 (0.049 to 0.204) | 0.34 (0.15 to 0.62) | 3.06 (1.49 to 5.75) | 1.97 (1.74 to 2.19) |
| Peru | Acute myeloid leukemia | death | 0.005 (0.002 to 0.01) | 0.04 (0.01 to 0.07) | 0.028 (0.012 to 0.054) | 0.09 (0.04 to 0.16) | 4.92 (2.44 to 9.41) | 3.5 (3.2 to 3.8) |
| Peru | Acute lymphoid leukemia | death | 0.004 (0.001 to 0.008) | 0.03 (0.01 to 0.05) | 0.017 (0.007 to 0.031) | 0.05 (0.02 to 0.09) | 3.5 (1.48 to 7.08) | 2.65 (2.45 to 2.86) |
| Peru | Chronic myeloid leukemia | death | 0.002 (0.001 to 0.003) | 0.01 (0 to 0.03) | 0.006 (0.003 to 0.011) | 0.02 (0.01 to 0.03) | 2.74 (1.3 to 5.8) | 1.92 (1.58 to 2.26) |
| Peru | Chronic lymphoid leukemia | death | 0.001 (0 to 0.002) | 0.01 (0 to 0.02) | 0.006 (0.003 to 0.011) | 0.02 (0.01 to 0.04) | 4.95 (2.66 to 9.3) | 3.26 (2.96 to 3.56) |
| Peru | Other leukemia | death | 0.016 (0.006 to 0.031) | 0.12 (0.05 to 0.24) | 0.053 (0.023 to 0.103) | 0.16 (0.07 to 0.31) | 2.31 (1 to 4.77) | 1.07 (0.8 to 1.35) |
| Peru | Leukemia | DALYs | 0.973 (0.357 to 1.906) | 6.36 (2.41 to 12.25) | 3.521 (1.573 to 6.468) | 10.38 (4.65 to 19.04) | 2.62 (1.16 to 5.31) | 1.94 (1.71 to 2.16) |
| Peru | Acute myeloid leukemia | DALYs | 0.165 (0.062 to 0.336) | 1.12 (0.43 to 2.25) | 0.903 (0.397 to 1.676) | 2.67 (1.18 to 4.97) | 4.49 (2.14 to 8.95) | 3.55 (3.25 to 3.86) |
| Peru | Acute lymphoid leukemia | DALYs | 0.148 (0.049 to 0.322) | 0.89 (0.31 to 1.85) | 0.633 (0.277 to 1.179) | 1.82 (0.8 to 3.39) | 3.27 (1.24 to 7.1) | 2.85 (2.64 to 3.06) |
| Peru | Chronic myeloid leukemia | DALYs | 0.059 (0.022 to 0.117) | 0.39 (0.15 to 0.76) | 0.193 (0.086 to 0.349) | 0.57 (0.25 to 1.03) | 2.29 (0.97 to 5.09) | 1.77 (1.43 to 2.12) |
| Peru | Chronic lymphoid leukemia | DALYs | 0.031 (0.011 to 0.06) | 0.22 (0.08 to 0.43) | 0.159 (0.07 to 0.294) | 0.48 (0.21 to 0.9) | 4.19 (2.06 to 8.44) | 3.07 (2.78 to 3.37) |
| Peru | Other leukemia | DALYs | 0.571 (0.207 to 1.136) | 3.74 (1.42 to 7.37) | 1.633 (0.694 to 3.156) | 4.83 (2.05 to 9.35) | 1.86 (0.67 to 4.23) | 0.94 (0.66 to 1.22) |
| Philippines | Leukemia | death | 0.047 (0.016 to 0.096) | 0.14 (0.05 to 0.29) | 0.151 (0.068 to 0.281) | 0.18 (0.08 to 0.33) | 2.2 (1.34 to 4.3) | 0.22 (-0.09 to 0.52) |
| Philippines | Acute myeloid leukemia | death | 0.01 (0.004 to 0.021) | 0.03 (0.01 to 0.06) | 0.042 (0.019 to 0.078) | 0.05 (0.02 to 0.09) | 3.04 (1.94 to 5.63) | 0.91 (0.53 to 1.29) |
| Philippines | Acute lymphoid leukemia | death | 0.004 (0.001 to 0.007) | 0.01 (0 to 0.02) | 0.013 (0.006 to 0.024) | 0.01 (0.01 to 0.03) | 2.5 (1.61 to 4.51) | 0.7 (0.4 to 1.01) |
| Philippines | Chronic myeloid leukemia | death | 0.004 (0.002 to 0.009) | 0.01 (0 to 0.03) | 0.012 (0.005 to 0.022) | 0.01 (0.01 to 0.03) | 1.72 (0.94 to 3.51) | -0.25 (-0.56 to 0.06) |
| Philippines | Chronic lymphoid leukemia | death | 0.001 (0 to 0.002) | 0 (0 to 0.01) | 0.004 (0.002 to 0.007) | 0.01 (0 to 0.01) | 2.94 (1.79 to 5.75) | 0.44 (0 to 0.89) |
| Philippines | Other leukemia | death | 0.028 (0.009 to 0.059) | 0.08 (0.03 to 0.18) | 0.08 (0.035 to 0.152) | 0.1 (0.04 to 0.18) | 1.9 (1.09 to 3.95) | -0.08 (-0.35 to 0.19) |
| Philippines | Leukemia | DALYs | 1.728 (0.596 to 3.53) | 4.21 (1.45 to 8.59) | 5.458 (2.469 to 10.184) | 5.62 (2.54 to 10.52) | 2.16 (1.35 to 4.13) | 0.51 (0.25 to 0.77) |
| Philippines | Acute myeloid leukemia | DALYs | 0.383 (0.138 to 0.764) | 0.93 (0.33 to 1.86) | 1.543 (0.683 to 2.869) | 1.58 (0.7 to 2.91) | 3.03 (1.91 to 5.53) | 1.22 (0.89 to 1.55) |
| Philippines | Acute lymphoid leukemia | DALYs | 0.151 (0.052 to 0.316) | 0.33 (0.11 to 0.67) | 0.524 (0.232 to 0.99) | 0.5 (0.22 to 0.94) | 2.47 (1.54 to 4.48) | 1.03 (0.77 to 1.29) |
| Philippines | Chronic myeloid leukemia | DALYs | 0.163 (0.059 to 0.327) | 0.39 (0.14 to 0.79) | 0.428 (0.194 to 0.783) | 0.44 (0.2 to 0.81) | 1.63 (0.9 to 3.16) | -0.01 (-0.28 to 0.26) |
| Philippines | Chronic lymphoid leukemia | DALYs | 0.03 (0.011 to 0.06) | 0.09 (0.03 to 0.18) | 0.117 (0.054 to 0.208) | 0.14 (0.06 to 0.24) | 2.9 (1.82 to 5.42) | 0.65 (0.23 to 1.08) |
| Philippines | Other leukemia | DALYs | 1.001 (0.338 to 2.146) | 2.48 (0.84 to 5.21) | 2.846 (1.246 to 5.425) | 2.97 (1.3 to 5.63) | 1.84 (1.08 to 3.72) | 0.19 (-0.03 to 0.42) |
| Poland | Leukemia | death | 0.203 (0.098 to 0.334) | 0.47 (0.22 to 0.77) | 0.4 (0.206 to 0.669) | 0.57 (0.29 to 0.96) | 0.97 (0.61 to 1.44) | 0.61 (0.47 to 0.75) |
| Poland | Acute myeloid leukemia | death | 0.051 (0.023 to 0.108) | 0.12 (0.05 to 0.25) | 0.148 (0.067 to 0.255) | 0.22 (0.1 to 0.38) | 1.9 (0.02 to 3.27) | 2.38 (2.18 to 2.58) |
| Poland | Acute lymphoid leukemia | death | 0.017 (0.008 to 0.028) | 0.04 (0.02 to 0.07) | 0.021 (0.011 to 0.036) | 0.03 (0.02 to 0.06) | 0.28 (-0.11 to 0.7) | -0.59 (-0.7 to -0.48) |
| Poland | Chronic myeloid leukemia | death | 0.034 (0.015 to 0.058) | 0.08 (0.04 to 0.13) | 0.029 (0.014 to 0.053) | 0.04 (0.02 to 0.08) | -0.16 (-0.38 to 0.95) | -2.86 (-3.3 to -2.42) |
| Poland | Chronic lymphoid leukemia | death | 0.018 (0.008 to 0.032) | 0.04 (0.02 to 0.07) | 0.134 (0.067 to 0.23) | 0.18 (0.09 to 0.32) | 6.44 (3.69 to 13.61) | 6 (4.94 to 7.09) |
| Poland | Other leukemia | death | 0.083 (0.039 to 0.142) | 0.19 (0.09 to 0.33) | 0.068 (0.034 to 0.124) | 0.09 (0.05 to 0.17) | -0.18 (-0.38 to 0.71) | -3.38 (-3.69 to -3.06) |
| Poland | Leukemia | DALYs | 5.36 (2.607 to 8.913) | 12.36 (5.99 to 20.6) | 8.947 (4.663 to 14.621) | 13.87 (7.2 to 22.7) | 0.67 (0.37 to 1.1) | 0.31 (0.19 to 0.43) |
| Poland | Acute myeloid leukemia | DALYs | 1.517 (0.679 to 3.079) | 3.52 (1.58 to 7.13) | 3.581 (1.691 to 6.095) | 5.77 (2.79 to 9.74) | 1.36 (-0.06 to 2.44) | 1.82 (1.65 to 1.99) |
| Poland | Acute lymphoid leukemia | DALYs | 0.556 (0.268 to 0.945) | 1.32 (0.63 to 2.27) | 0.673 (0.346 to 1.112) | 1.26 (0.64 to 2.11) | 0.21 (-0.1 to 0.61) | -0.38 (-0.5 to -0.26) |
| Poland | Chronic myeloid leukemia | DALYs | 0.938 (0.424 to 1.618) | 2.17 (0.98 to 3.73) | 0.607 (0.292 to 1.175) | 0.94 (0.45 to 1.84) | -0.35 (-0.53 to 0.53) | -3.56 (-4 to -3.12) |
| Poland | Chronic lymphoid leukemia | DALYs | 0.405 (0.181 to 0.715) | 0.92 (0.41 to 1.63) | 2.769 (1.407 to 4.736) | 3.95 (2.01 to 6.77) | 5.84 (3.16 to 12.96) | 5.9 (4.81 to 7) |
| Poland | Other leukemia | DALYs | 1.944 (0.909 to 3.305) | 4.43 (2.06 to 7.54) | 1.317 (0.655 to 2.399) | 1.94 (0.96 to 3.56) | -0.32 (-0.5 to 0.48) | -3.68 (-3.99 to -3.37) |
| Portugal | Leukemia | death | 0.038 (0.015 to 0.07) | 0.29 (0.11 to 0.53) | 0.082 (0.037 to 0.143) | 0.34 (0.15 to 0.59) | 1.14 (0.79 to 1.68) | 0.17 (-0.08 to 0.42) |
| Portugal | Acute myeloid leukemia | death | 0.01 (0.004 to 0.019) | 0.08 (0.03 to 0.14) | 0.032 (0.014 to 0.056) | 0.14 (0.07 to 0.25) | 2.19 (1.31 to 3.25) | 1.77 (1.34 to 2.2) |
| Portugal | Acute lymphoid leukemia | death | 0.003 (0.001 to 0.005) | 0.02 (0.01 to 0.04) | 0.004 (0.002 to 0.007) | 0.02 (0.01 to 0.04) | 0.55 (0.22 to 1.25) | -0.69 (-1.07 to -0.32) |
| Portugal | Chronic myeloid leukemia | death | 0.006 (0.002 to 0.011) | 0.04 (0.02 to 0.08) | 0.005 (0.002 to 0.009) | 0.02 (0.01 to 0.04) | -0.14 (-0.31 to 0.14) | -3.93 (-4.42 to -3.45) |
| Portugal | Chronic lymphoid leukemia | death | 0.006 (0.002 to 0.011) | 0.04 (0.02 to 0.08) | 0.02 (0.009 to 0.035) | 0.07 (0.03 to 0.13) | 2.24 (1.59 to 3.3) | 1.39 (0.99 to 1.8) |
| Portugal | Other leukemia | death | 0.014 (0.005 to 0.025) | 0.1 (0.04 to 0.19) | 0.021 (0.009 to 0.037) | 0.08 (0.04 to 0.14) | 0.55 (0.25 to 1.03) | -0.91 (-1.19 to -0.63) |
| Portugal | Leukemia | DALYs | 0.998 (0.398 to 1.833) | 7.87 (3.1 to 14.58) | 1.63 (0.74 to 2.835) | 8.3 (3.8 to 14.44) | 0.63 (0.39 to 1.08) | -0.26 (-0.52 to 0) |
| Portugal | Acute myeloid leukemia | DALYs | 0.286 (0.111 to 0.537) | 2.31 (0.9 to 4.35) | 0.694 (0.314 to 1.218) | 3.77 (1.7 to 6.63) | 1.43 (0.88 to 2.21) | 1.18 (0.74 to 1.62) |
| Portugal | Acute lymphoid leukemia | DALYs | 0.096 (0.036 to 0.186) | 0.83 (0.3 to 1.64) | 0.146 (0.065 to 0.259) | 0.97 (0.43 to 1.74) | 0.52 (0.15 to 1.28) | -0.21 (-0.56 to 0.15) |
| Portugal | Chronic myeloid leukemia | DALYs | 0.147 (0.059 to 0.274) | 1.15 (0.46 to 2.15) | 0.101 (0.045 to 0.18) | 0.51 (0.22 to 0.92) | -0.31 (-0.45 to -0.09) | -4.17 (-4.65 to -3.69) |
| Portugal | Chronic lymphoid leukemia | DALYs | 0.125 (0.05 to 0.228) | 0.88 (0.35 to 1.61) | 0.331 (0.148 to 0.589) | 1.37 (0.62 to 2.42) | 1.66 (1.13 to 2.47) | 1.23 (0.83 to 1.63) |
| Portugal | Other leukemia | DALYs | 0.345 (0.136 to 0.637) | 2.7 (1.06 to 5.03) | 0.358 (0.158 to 0.627) | 1.69 (0.76 to 3.04) | 0.04 (-0.15 to 0.34) | -1.75 (-2 to -1.49) |
| Puerto Rico | Leukemia | death | 0.016 (0.008 to 0.026) | 0.45 (0.21 to 0.73) | 0.031 (0.016 to 0.051) | 0.46 (0.24 to 0.76) | 0.94 (0.51 to 1.54) | 0.03 (-0.07 to 0.13) |
| Puerto Rico | Acute myeloid leukemia | death | 0.004 (0.002 to 0.007) | 0.12 (0.06 to 0.21) | 0.009 (0.005 to 0.015) | 0.15 (0.08 to 0.26) | 1.07 (0.55 to 1.86) | 0.77 (0.61 to 0.92) |
| Puerto Rico | Acute lymphoid leukemia | death | 0.001 (0.001 to 0.002) | 0.03 (0.02 to 0.06) | 0.002 (0.001 to 0.003) | 0.04 (0.02 to 0.06) | 0.53 (0.13 to 1.1) | 0.2 (0.02 to 0.39) |
| Puerto Rico | Chronic myeloid leukemia | death | 0.002 (0.001 to 0.004) | 0.06 (0.03 to 0.11) | 0.002 (0.001 to 0.004) | 0.03 (0.01 to 0.05) | -0.1 (-0.33 to 0.25) | -3.6 (-4.05 to -3.14) |
| Puerto Rico | Chronic lymphoid leukemia | death | 0.002 (0.001 to 0.003) | 0.04 (0.02 to 0.07) | 0.003 (0.001 to 0.005) | 0.04 (0.02 to 0.06) | 0.84 (0.34 to 1.6) | -1.15 (-1.4 to -0.91) |
| Puerto Rico | Other leukemia | death | 0.007 (0.003 to 0.011) | 0.18 (0.08 to 0.31) | 0.015 (0.008 to 0.026) | 0.21 (0.1 to 0.35) | 1.32 (0.76 to 2.08) | 0.57 (0.22 to 0.91) |
| Puerto Rico | Leukemia | DALYs | 0.459 (0.225 to 0.741) | 12.75 (6.26 to 20.58) | 0.698 (0.364 to 1.157) | 12.87 (6.8 to 21.18) | 0.52 (0.17 to 1.03) | -0.09 (-0.21 to 0.02) |
| Puerto Rico | Acute myeloid leukemia | DALYs | 0.148 (0.069 to 0.245) | 4.12 (1.91 to 6.78) | 0.245 (0.124 to 0.409) | 4.92 (2.48 to 8.19) | 0.66 (0.21 to 1.3) | 0.57 (0.44 to 0.71) |
| Puerto Rico | Acute lymphoid leukemia | DALYs | 0.05 (0.024 to 0.082) | 1.38 (0.66 to 2.27) | 0.066 (0.034 to 0.111) | 1.52 (0.78 to 2.55) | 0.32 (-0.04 to 0.83) | 0.34 (0.16 to 0.51) |
| Puerto Rico | Chronic myeloid leukemia | DALYs | 0.066 (0.032 to 0.108) | 1.84 (0.89 to 3.01) | 0.046 (0.023 to 0.079) | 0.85 (0.43 to 1.46) | -0.31 (-0.49 to -0.03) | -3.69 (-4.14 to -3.24) |
| Puerto Rico | Chronic lymphoid leukemia | DALYs | 0.032 (0.015 to 0.053) | 0.88 (0.43 to 1.48) | 0.051 (0.026 to 0.086) | 0.74 (0.37 to 1.24) | 0.62 (0.17 to 1.29) | -1.04 (-1.27 to -0.81) |
| Puerto Rico | Other leukemia | DALYs | 0.164 (0.08 to 0.272) | 4.53 (2.2 to 7.54) | 0.291 (0.148 to 0.479) | 4.84 (2.51 to 7.96) | 0.77 (0.32 to 1.38) | 0.29 (-0.09 to 0.66) |
| Qatar | Leukemia | death | 0.001 (0.001 to 0.002) | 0.78 (0.39 to 1.27) | 0.008 (0.004 to 0.013) | 1.12 (0.58 to 1.91) | 6.57 (4.16 to 10.17) | 1.6 (1.39 to 1.82) |
| Qatar | Acute myeloid leukemia | death | 0 (0 to 0.001) | 0.18 (0.09 to 0.31) | 0.003 (0.002 to 0.006) | 0.29 (0.14 to 0.5) | 9.07 (5.5 to 14.38) | 1.93 (1.8 to 2.06) |
| Qatar | Acute lymphoid leukemia | death | 0 (0 to 0) | 0.02 (0.01 to 0.03) | 0 (0 to 0.001) | 0.03 (0.01 to 0.05) | 7.28 (3.86 to 13.71) | 1.26 (0.94 to 1.58) |
| Qatar | Chronic myeloid leukemia | death | 0 (0 to 0) | 0.11 (0.05 to 0.18) | 0.001 (0.001 to 0.002) | 0.13 (0.06 to 0.25) | 4.33 (2.12 to 8.12) | 0.87 (0.58 to 1.17) |
| Qatar | Chronic lymphoid leukemia | death | 0 (0 to 0) | 0.35 (0.16 to 0.6) | 0.002 (0.001 to 0.003) | 0.55 (0.28 to 0.93) | 6.7 (4.29 to 10.79) | 2.09 (1.8 to 2.38) |
| Qatar | Other leukemia | death | 0 (0 to 0) | 0.12 (0.05 to 0.21) | 0.001 (0 to 0.002) | 0.11 (0.05 to 0.2) | 4.11 (2.01 to 9.48) | -0.06 (-0.3 to 0.18) |
| Qatar | Leukemia | DALYs | 0.04 (0.02 to 0.066) | 18.22 (9.5 to 28.96) | 0.304 (0.157 to 0.523) | 20.52 (10.95 to 32.9) | 6.6 (4.12 to 10.15) | 0.57 (0.38 to 0.76) |
| Qatar | Acute myeloid leukemia | DALYs | 0.014 (0.006 to 0.027) | 4.94 (2.4 to 8.29) | 0.14 (0.067 to 0.274) | 6.5 (3.46 to 10.68) | 8.92 (5.36 to 14.36) | 1.06 (0.97 to 1.15) |
| Qatar | Acute lymphoid leukemia | DALYs | 0.003 (0.001 to 0.006) | 0.67 (0.3 to 1.28) | 0.025 (0.011 to 0.049) | 0.79 (0.37 to 1.46) | 7.7 (4.04 to 14.89) | 0.3 (0.11 to 0.49) |
| Qatar | Chronic myeloid leukemia | DALYs | 0.009 (0.004 to 0.015) | 2.98 (1.48 to 4.91) | 0.048 (0.023 to 0.088) | 2.46 (1.2 to 4.43) | 4.35 (2.09 to 8.35) | -0.86 (-1.03 to -0.69) |
| Qatar | Chronic lymphoid leukemia | DALYs | 0.007 (0.003 to 0.011) | 6.48 (3.05 to 10.9) | 0.055 (0.029 to 0.091) | 8.54 (4.44 to 13.81) | 7.03 (4.37 to 11.37) | 1.4 (1.07 to 1.73) |
| Qatar | Other leukemia | DALYs | 0.007 (0.003 to 0.012) | 3.15 (1.44 to 5.42) | 0.036 (0.016 to 0.063) | 2.23 (1.06 to 3.83) | 3.98 (1.89 to 9.42) | -1.28 (-1.57 to -0.98) |
| Republic of Korea | Leukemia | death | 0.041 (0.011 to 0.09) | 0.12 (0.03 to 0.25) | 0.094 (0.035 to 0.184) | 0.11 (0.04 to 0.22) | 1.28 (0.46 to 2.64) | -0.49 (-0.63 to -0.35) |
| Republic of Korea | Acute myeloid leukemia | death | 0.008 (0.002 to 0.02) | 0.02 (0.01 to 0.05) | 0.042 (0.015 to 0.083) | 0.05 (0.02 to 0.1) | 3.98 (1.64 to 7.88) | 3.36 (3.04 to 3.68) |
| Republic of Korea | Acute lymphoid leukemia | death | 0.003 (0.001 to 0.008) | 0.01 (0 to 0.02) | 0.009 (0.003 to 0.018) | 0.01 (0 to 0.02) | 1.82 (0.2 to 4.26) | 1.44 (1.14 to 1.75) |
| Republic of Korea | Chronic myeloid leukemia | death | 0.005 (0.001 to 0.013) | 0.01 (0 to 0.03) | 0.007 (0.002 to 0.013) | 0.01 (0 to 0.02) | 0.29 (-0.41 to 1.33) | -2.17 (-2.3 to -2.03) |
| Republic of Korea | Chronic lymphoid leukemia | death | 0.001 (0 to 0.002) | 0 (0 to 0.01) | 0.003 (0.001 to 0.006) | 0 (0 to 0.01) | 3.53 (0.92 to 7.8) | 1.24 (0.8 to 1.69) |
| Republic of Korea | Other leukemia | death | 0.024 (0.006 to 0.053) | 0.07 (0.02 to 0.16) | 0.033 (0.012 to 0.068) | 0.04 (0.01 to 0.08) | 0.4 (0.01 to 1.68) | -3.05 (-3.48 to -2.63) |
| Republic of Korea | Leukemia | DALYs | 1.594 (0.406 to 3.579) | 3.85 (1.01 to 8.47) | 2.503 (0.938 to 4.893) | 3.13 (1.16 to 6.16) | 0.57 (0.09 to 1.54) | -1.11 (-1.3 to -0.92) |
| Republic of Korea | Acute myeloid leukemia | DALYs | 0.347 (0.082 to 0.814) | 0.8 (0.19 to 1.88) | 1.162 (0.424 to 2.291) | 1.45 (0.52 to 2.86) | 2.35 (0.87 to 5.02) | 2.5 (2.22 to 2.78) |
| Republic of Korea | Acute lymphoid leukemia | DALYs | 0.146 (0.034 to 0.377) | 0.31 (0.07 to 0.8) | 0.375 (0.133 to 0.75) | 0.51 (0.18 to 1.04) | 1.56 (0.13 to 4.06) | 1.82 (1.45 to 2.19) |
| Republic of Korea | Chronic myeloid leukemia | DALYs | 0.213 (0.051 to 0.529) | 0.49 (0.12 to 1.22) | 0.191 (0.068 to 0.377) | 0.24 (0.08 to 0.47) | -0.1 (-0.56 to 0.7) | -2.91 (-3.1 to -2.73) |
| Republic of Korea | Chronic lymphoid leukemia | DALYs | 0.019 (0.005 to 0.048) | 0.06 (0.01 to 0.14) | 0.074 (0.026 to 0.151) | 0.09 (0.03 to 0.18) | 2.84 (0.66 to 6.41) | 1.56 (1.15 to 1.97) |
| Republic of Korea | Other leukemia | DALYs | 0.869 (0.228 to 2.025) | 2.19 (0.59 to 4.97) | 0.702 (0.256 to 1.468) | 0.85 (0.3 to 1.78) | -0.19 (-0.43 to 0.72) | -4.47 (-4.96 to -3.99) |
| Republic of Moldova | Leukemia | death | 0.014 (0.006 to 0.024) | 0.3 (0.14 to 0.52) | 0.014 (0.007 to 0.023) | 0.25 (0.12 to 0.41) | 0.03 (-0.14 to 0.3) | -0.1 (-0.54 to 0.34) |
| Republic of Moldova | Acute myeloid leukemia | death | 0.003 (0.001 to 0.006) | 0.07 (0.03 to 0.13) | 0.004 (0.002 to 0.007) | 0.08 (0.04 to 0.13) | 0.36 (0.02 to 0.82) | 1.08 (0.52 to 1.65) |
| Republic of Moldova | Acute lymphoid leukemia | death | 0.003 (0.001 to 0.005) | 0.06 (0.02 to 0.11) | 0.002 (0.001 to 0.003) | 0.03 (0.02 to 0.06) | -0.31 (-0.48 to 0.15) | -1.51 (-1.8 to -1.23) |
| Republic of Moldova | Chronic myeloid leukemia | death | 0.001 (0 to 0.002) | 0.02 (0.01 to 0.03) | 0.001 (0 to 0.001) | 0.01 (0.01 to 0.02) | -0.29 (-0.49 to 0.1) | -2.06 (-2.65 to -1.47) |
| Republic of Moldova | Chronic lymphoid leukemia | death | 0.001 (0.001 to 0.002) | 0.03 (0.01 to 0.05) | 0.002 (0.001 to 0.003) | 0.03 (0.01 to 0.05) | 0.35 (0.04 to 0.87) | 0.49 (-0.01 to 0.99) |
| Republic of Moldova | Other leukemia | death | 0.005 (0.002 to 0.01) | 0.12 (0.05 to 0.21) | 0.005 (0.003 to 0.009) | 0.1 (0.05 to 0.16) | -0.01 (-0.2 to 0.28) | -0.17 (-0.64 to 0.3) |
| Republic of Moldova | Leukemia | DALYs | 0.442 (0.201 to 0.772) | 9.49 (4.3 to 16.69) | 0.395 (0.196 to 0.647) | 7.4 (3.64 to 12.23) | -0.1 (-0.27 to 0.17) | -0.37 (-0.8 to 0.07) |
| Republic of Moldova | Acute myeloid leukemia | DALYs | 0.108 (0.048 to 0.193) | 2.31 (1.03 to 4.1) | 0.132 (0.064 to 0.218) | 2.49 (1.2 to 4.19) | 0.22 (-0.1 to 0.66) | 0.87 (0.31 to 1.42) |
| Republic of Moldova | Acute lymphoid leukemia | DALYs | 0.092 (0.036 to 0.166) | 1.98 (0.77 to 3.6) | 0.058 (0.028 to 0.095) | 1.12 (0.53 to 1.89) | -0.37 (-0.53 to 0.05) | -1.6 (-1.89 to -1.31) |
| Republic of Moldova | Chronic myeloid leukemia | DALYs | 0.028 (0.012 to 0.053) | 0.59 (0.25 to 1.11) | 0.018 (0.009 to 0.03) | 0.33 (0.16 to 0.56) | -0.36 (-0.53 to 0.01) | -2.2 (-2.78 to -1.6) |
| Republic of Moldova | Chronic lymphoid leukemia | DALYs | 0.031 (0.014 to 0.054) | 0.68 (0.31 to 1.19) | 0.036 (0.018 to 0.06) | 0.63 (0.31 to 1.04) | 0.18 (-0.1 to 0.63) | 0.24 (-0.26 to 0.74) |
| Republic of Moldova | Other leukemia | DALYs | 0.183 (0.081 to 0.325) | 3.94 (1.73 to 7.07) | 0.151 (0.075 to 0.249) | 2.83 (1.39 to 4.69) | -0.17 (-0.34 to 0.1) | -0.52 (-1 to -0.05) |
| Romania | Leukemia | death | 0.072 (0.036 to 0.117) | 0.26 (0.13 to 0.43) | 0.143 (0.074 to 0.23) | 0.41 (0.21 to 0.66) | 0.99 (0.59 to 1.55) | 1.76 (1.63 to 1.89) |
| Romania | Acute myeloid leukemia | death | 0.02 (0.01 to 0.033) | 0.07 (0.03 to 0.12) | 0.049 (0.025 to 0.078) | 0.14 (0.07 to 0.23) | 1.48 (0.85 to 2.3) | 2.95 (2.79 to 3.11) |
| Romania | Acute lymphoid leukemia | death | 0.007 (0.004 to 0.012) | 0.03 (0.01 to 0.05) | 0.013 (0.007 to 0.021) | 0.04 (0.02 to 0.07) | 0.74 (0.29 to 1.3) | 1.59 (1.41 to 1.78) |
| Romania | Chronic myeloid leukemia | death | 0.006 (0.003 to 0.009) | 0.02 (0.01 to 0.03) | 0.006 (0.003 to 0.01) | 0.02 (0.01 to 0.03) | 0.08 (-0.18 to 0.46) | -0.83 (-1.19 to -0.46) |
| Romania | Chronic lymphoid leukemia | death | 0.011 (0.005 to 0.018) | 0.04 (0.02 to 0.06) | 0.03 (0.016 to 0.05) | 0.08 (0.04 to 0.13) | 1.73 (1.06 to 2.71) | 2.79 (2.52 to 3.07) |
| Romania | Other leukemia | death | 0.028 (0.014 to 0.046) | 0.1 (0.05 to 0.17) | 0.045 (0.023 to 0.072) | 0.13 (0.07 to 0.2) | 0.6 (0.26 to 1.08) | 0.73 (0.58 to 0.88) |
| Romania | Leukemia | DALYs | 2.202 (1.094 to 3.672) | 8.03 (3.95 to 13.47) | 3.527 (1.843 to 5.692) | 11.46 (6.04 to 18.5) | 0.6 (0.27 to 1.07) | 1.35 (1.23 to 1.46) |
| Romania | Acute myeloid leukemia | DALYs | 0.634 (0.305 to 1.064) | 2.33 (1.1 to 3.95) | 1.273 (0.652 to 2.045) | 4.31 (2.23 to 6.93) | 1.01 (0.5 to 1.7) | 2.5 (2.35 to 2.65) |
| Romania | Acute lymphoid leukemia | DALYs | 0.256 (0.123 to 0.439) | 0.97 (0.46 to 1.68) | 0.377 (0.203 to 0.618) | 1.4 (0.75 to 2.3) | 0.48 (0.1 to 1.04) | 1.49 (1.33 to 1.64) |
| Romania | Chronic myeloid leukemia | DALYs | 0.176 (0.087 to 0.298) | 0.65 (0.32 to 1.1) | 0.151 (0.081 to 0.241) | 0.5 (0.27 to 0.8) | -0.14 (-0.36 to 0.17) | -1.26 (-1.6 to -0.92) |
| Romania | Chronic lymphoid leukemia | DALYs | 0.29 (0.144 to 0.478) | 1.01 (0.5 to 1.66) | 0.658 (0.353 to 1.075) | 1.86 (0.99 to 3.03) | 1.27 (0.69 to 2.12) | 2.4 (2.15 to 2.66) |
| Romania | Other leukemia | DALYs | 0.846 (0.434 to 1.417) | 3.08 (1.56 to 5.19) | 1.068 (0.564 to 1.731) | 3.41 (1.78 to 5.49) | 0.26 (-0.01 to 0.67) | 0.25 (0.1 to 0.41) |
| Russian Federation | Leukemia | death | 0.567 (0.269 to 0.952) | 0.31 (0.15 to 0.53) | 0.868 (0.434 to 1.415) | 0.38 (0.19 to 0.62) | 0.53 (0.3 to 0.88) | 0.5 (0.27 to 0.74) |
| Russian Federation | Acute myeloid leukemia | death | 0.141 (0.063 to 0.241) | 0.08 (0.03 to 0.13) | 0.174 (0.085 to 0.296) | 0.08 (0.04 to 0.13) | 0.24 (-0.02 to 0.74) | -0.39 (-0.62 to -0.16) |
| Russian Federation | Acute lymphoid leukemia | death | 0.098 (0.046 to 0.168) | 0.05 (0.03 to 0.09) | 0.142 (0.071 to 0.232) | 0.07 (0.03 to 0.11) | 0.45 (0.21 to 0.81) | 0.81 (0.59 to 1.02) |
| Russian Federation | Chronic myeloid leukemia | death | 0.067 (0.031 to 0.117) | 0.04 (0.02 to 0.06) | 0.104 (0.051 to 0.171) | 0.05 (0.02 to 0.07) | 0.55 (0.16 to 0.99) | 0.51 (-0.03 to 1.05) |
| Russian Federation | Chronic lymphoid leukemia | death | 0.117 (0.055 to 0.199) | 0.06 (0.03 to 0.11) | 0.264 (0.131 to 0.43) | 0.11 (0.06 to 0.18) | 1.25 (0.81 to 1.89) | 2.09 (1.81 to 2.37) |
| Russian Federation | Other leukemia | death | 0.145 (0.068 to 0.248) | 0.08 (0.04 to 0.14) | 0.184 (0.09 to 0.303) | 0.08 (0.04 to 0.13) | 0.27 (0.03 to 0.56) | -0.49 (-0.85 to -0.12) |
| Russian Federation | Leukemia | DALYs | 16.715 (7.945 to 28.224) | 9.29 (4.37 to 15.67) | 22.569 (11.444 to 36.375) | 10.46 (5.33 to 16.88) | 0.35 (0.14 to 0.66) | 0.12 (-0.15 to 0.38) |
| Russian Federation | Acute myeloid leukemia | DALYs | 4.535 (2.011 to 7.84) | 2.55 (1.12 to 4.41) | 5.102 (2.518 to 8.591) | 2.48 (1.22 to 4.18) | 0.12 (-0.11 to 0.61) | -0.6 (-0.84 to -0.37) |
| Russian Federation | Acute lymphoid leukemia | DALYs | 3.233 (1.521 to 5.574) | 1.84 (0.85 to 3.17) | 4.397 (2.237 to 7.201) | 2.21 (1.12 to 3.65) | 0.36 (0.13 to 0.73) | 0.76 (0.53 to 0.99) |
| Russian Federation | Chronic myeloid leukemia | DALYs | 1.919 (0.888 to 3.384) | 1.06 (0.49 to 1.88) | 2.622 (1.297 to 4.296) | 1.2 (0.6 to 1.97) | 0.37 (0.03 to 0.78) | 0.03 (-0.58 to 0.64) |
| Russian Federation | Chronic lymphoid leukemia | DALYs | 2.955 (1.391 to 5.048) | 1.59 (0.74 to 2.73) | 6.065 (3.097 to 9.854) | 2.57 (1.3 to 4.16) | 1.05 (0.66 to 1.68) | 1.8 (1.5 to 2.1) |
| Russian Federation | Other leukemia | DALYs | 4.072 (1.899 to 6.919) | 2.26 (1.06 to 3.84) | 4.383 (2.184 to 7.201) | 2 (1.01 to 3.27) | 0.08 (-0.14 to 0.35) | -1.1 (-1.55 to -0.66) |
| Rwanda | Leukemia | death | 0.002 (0 to 0.005) | 0.06 (0.01 to 0.15) | 0.007 (0.002 to 0.014) | 0.11 (0.04 to 0.21) | 2.36 (1.02 to 6.4) | 1.82 (1.38 to 2.25) |
| Rwanda | Acute myeloid leukemia | death | 0 (0 to 0) | 0 (0 to 0.01) | 0.001 (0 to 0.002) | 0.01 (0 to 0.02) | 4.67 (1.9 to 12.37) | 3.97 (3.51 to 4.43) |
| Rwanda | Acute lymphoid leukemia | death | 0 (0 to 0) | 0 (0 to 0.01) | 0 (0 to 0.001) | 0 (0 to 0.01) | 3.39 (0.91 to 11.01) | 2.49 (2.02 to 2.96) |
| Rwanda | Chronic myeloid leukemia | death | 0.001 (0 to 0.002) | 0.02 (0 to 0.05) | 0.001 (0.001 to 0.003) | 0.02 (0.01 to 0.04) | 1.35 (0.25 to 5.34) | -0.08 (-0.63 to 0.48) |
| Rwanda | Chronic lymphoid leukemia | death | 0 (0 to 0.001) | 0.02 (0 to 0.05) | 0.002 (0.001 to 0.005) | 0.05 (0.02 to 0.09) | 4.36 (2.25 to 10.16) | 3.77 (3.38 to 4.16) |
| Rwanda | Other leukemia | death | 0.001 (0 to 0.002) | 0.02 (0 to 0.06) | 0.002 (0.001 to 0.004) | 0.03 (0.01 to 0.05) | 1.44 (0.3 to 5.16) | 0.4 (0.05 to 0.75) |
| Rwanda | Leukemia | DALYs | 0.074 (0.017 to 0.181) | 1.92 (0.45 to 4.72) | 0.23 (0.081 to 0.465) | 2.95 (1.05 to 5.84) | 2.12 (0.77 to 6.36) | 1.32 (0.87 to 1.78) |
| Rwanda | Acute myeloid leukemia | DALYs | 0.006 (0.001 to 0.017) | 0.14 (0.03 to 0.39) | 0.034 (0.011 to 0.072) | 0.37 (0.13 to 0.79) | 4.69 (1.75 to 12.85) | 3.94 (3.48 to 4.4) |
| Rwanda | Acute lymphoid leukemia | DALYs | 0.004 (0.001 to 0.012) | 0.08 (0.02 to 0.25) | 0.017 (0.005 to 0.04) | 0.17 (0.05 to 0.37) | 3.52 (0.87 to 12.23) | 2.7 (2.26 to 3.15) |
| Rwanda | Chronic myeloid leukemia | DALYs | 0.025 (0.005 to 0.068) | 0.62 (0.12 to 1.63) | 0.057 (0.019 to 0.122) | 0.66 (0.22 to 1.39) | 1.27 (0.15 to 5.76) | -0.36 (-0.92 to 0.21) |
| Rwanda | Chronic lymphoid leukemia | DALYs | 0.011 (0.003 to 0.028) | 0.39 (0.09 to 0.96) | 0.058 (0.02 to 0.118) | 0.97 (0.34 to 1.96) | 4.13 (2.08 to 9.85) | 3.45 (3.05 to 3.85) |
| Rwanda | Other leukemia | DALYs | 0.028 (0.006 to 0.073) | 0.69 (0.16 to 1.78) | 0.065 (0.021 to 0.141) | 0.78 (0.26 to 1.68) | 1.34 (0.2 to 5.37) | 0.08 (-0.29 to 0.46) |
| Saint Kitts and Nevis | Leukemia | death | 0 (0 to 0) | 0.4 (0.17 to 0.71) | 0 (0 to 0) | 0.34 (0.16 to 0.57) | 0.54 (0.13 to 1.18) | -0.55 (-0.7 to -0.4) |
| Saint Kitts and Nevis | Acute myeloid leukemia | death | 0 (0 to 0) | 0.01 (0.01 to 0.02) | 0 (0 to 0) | 0.02 (0.01 to 0.03) | 2.11 (1.04 to 3.69) | 1.33 (1.21 to 1.44) |
| Saint Kitts and Nevis | Acute lymphoid leukemia | death | 0 (0 to 0) | 0.02 (0.01 to 0.03) | 0 (0 to 0) | 0.02 (0.01 to 0.03) | 1.3 (0.39 to 2.55) | 0.84 (0.73 to 0.96) |
| Saint Kitts and Nevis | Chronic myeloid leukemia | death | 0 (0 to 0) | 0.06 (0.03 to 0.11) | 0 (0 to 0) | 0.05 (0.02 to 0.08) | 0.43 (0.02 to 1.06) | -0.99 (-1.16 to -0.82) |
| Saint Kitts and Nevis | Chronic lymphoid leukemia | death | 0 (0 to 0) | 0.02 (0.01 to 0.04) | 0 (0 to 0) | 0.03 (0.02 to 0.06) | 1.74 (0.95 to 3.18) | 0.99 (0.79 to 1.19) |
| Saint Kitts and Nevis | Other leukemia | death | 0 (0 to 0) | 0.29 (0.12 to 0.52) | 0 (0 to 0) | 0.22 (0.1 to 0.38) | 0.36 (0 to 0.9) | -0.85 (-1 to -0.69) |
| Saint Kitts and Nevis | Leukemia | DALYs | 0.004 (0.002 to 0.008) | 12.67 (5.52 to 22.07) | 0.007 (0.003 to 0.011) | 9.44 (4.36 to 16.28) | 0.5 (-0.01 to 1.22) | -1.09 (-1.27 to -0.9) |
| Saint Kitts and Nevis | Acute myeloid leukemia | DALYs | 0 (0 to 0) | 0.47 (0.21 to 0.83) | 0 (0 to 0.001) | 0.68 (0.29 to 1.24) | 2.03 (0.8 to 3.87) | 1.27 (1.13 to 1.4) |
| Saint Kitts and Nevis | Acute lymphoid leukemia | DALYs | 0 (0 to 0) | 0.59 (0.24 to 1.12) | 0 (0 to 0.001) | 0.67 (0.26 to 1.3) | 1.18 (0.15 to 2.55) | 0.62 (0.46 to 0.78) |
| Saint Kitts and Nevis | Chronic myeloid leukemia | DALYs | 0.001 (0 to 0.001) | 2.04 (0.91 to 3.6) | 0.001 (0 to 0.002) | 1.36 (0.63 to 2.33) | 0.38 (-0.1 to 1.09) | -1.52 (-1.74 to -1.3) |
| Saint Kitts and Nevis | Chronic lymphoid leukemia | DALYs | 0 (0 to 0) | 0.54 (0.23 to 0.99) | 0.001 (0 to 0.001) | 0.85 (0.39 to 1.5) | 2.15 (1.2 to 3.78) | 0.9 (0.68 to 1.12) |
| Saint Kitts and Nevis | Other leukemia | DALYs | 0.003 (0.001 to 0.006) | 9.03 (3.82 to 16.07) | 0.004 (0.002 to 0.007) | 5.89 (2.67 to 10.16) | 0.3 (-0.13 to 0.91) | -1.53 (-1.72 to -1.34) |
| Saint Lucia | Leukemia | death | 0 (0 to 0) | 0.28 (0.11 to 0.51) | 0.001 (0 to 0.001) | 0.33 (0.16 to 0.57) | 1.76 (1.16 to 2.88) | 0.18 (0.01 to 0.34) |
| Saint Lucia | Acute myeloid leukemia | death | 0 (0 to 0) | 0.04 (0.02 to 0.08) | 0 (0 to 0) | 0.08 (0.04 to 0.14) | 3.38 (2.11 to 5.51) | 2.27 (2.17 to 2.37) |
| Saint Lucia | Acute lymphoid leukemia | death | 0 (0 to 0) | 0.01 (0.01 to 0.03) | 0 (0 to 0) | 0.02 (0.01 to 0.04) | 2.08 (1.26 to 3.71) | 1.25 (1.14 to 1.37) |
| Saint Lucia | Chronic myeloid leukemia | death | 0 (0 to 0) | 0.05 (0.02 to 0.1) | 0 (0 to 0) | 0.04 (0.02 to 0.07) | 0.88 (0.43 to 1.67) | -1.47 (-1.75 to -1.18) |
| Saint Lucia | Chronic lymphoid leukemia | death | 0 (0 to 0) | 0.03 (0.01 to 0.06) | 0 (0 to 0) | 0.05 (0.02 to 0.09) | 2.66 (1.72 to 4.23) | 0.78 (0.52 to 1.03) |
| Saint Lucia | Other leukemia | death | 0 (0 to 0) | 0.14 (0.06 to 0.26) | 0 (0 to 0.001) | 0.14 (0.07 to 0.25) | 1.37 (0.83 to 2.36) | -0.44 (-0.63 to -0.25) |
| Saint Lucia | Leukemia | DALYs | 0.008 (0.003 to 0.015) | 8.7 (3.56 to 15.56) | 0.022 (0.011 to 0.037) | 10.44 (5.2 to 17.51) | 1.62 (1 to 2.72) | 0.38 (0.25 to 0.51) |
| Saint Lucia | Acute myeloid leukemia | DALYs | 0.001 (0.001 to 0.003) | 1.45 (0.59 to 2.72) | 0.006 (0.003 to 0.01) | 2.87 (1.39 to 4.96) | 3.16 (1.94 to 5.24) | 2.45 (2.32 to 2.57) |
| Saint Lucia | Acute lymphoid leukemia | DALYs | 0.001 (0 to 0.001) | 0.57 (0.22 to 1.07) | 0.002 (0.001 to 0.003) | 0.89 (0.44 to 1.49) | 1.85 (1.05 to 3.45) | 1.45 (1.33 to 1.57) |
| Saint Lucia | Chronic myeloid leukemia | DALYs | 0.002 (0.001 to 0.003) | 1.62 (0.65 to 2.96) | 0.003 (0.001 to 0.005) | 1.28 (0.62 to 2.16) | 0.76 (0.31 to 1.53) | -1.36 (-1.62 to -1.1) |
| Saint Lucia | Chronic lymphoid leukemia | DALYs | 0.001 (0 to 0.001) | 0.77 (0.31 to 1.4) | 0.002 (0.001 to 0.004) | 1.14 (0.55 to 1.96) | 2.59 (1.65 to 4.16) | 0.83 (0.58 to 1.07) |
| Saint Lucia | Other leukemia | DALYs | 0.004 (0.002 to 0.007) | 4.29 (1.73 to 7.7) | 0.009 (0.005 to 0.015) | 4.26 (2.13 to 7.23) | 1.2 (0.67 to 2.12) | -0.34 (-0.49 to -0.19) |
| Saint Vincent and the Grenadines | Leukemia | death | 0 (0 to 0) | 0.23 (0.08 to 0.43) | 0 (0 to 0.001) | 0.37 (0.18 to 0.63) | 1.91 (1.23 to 3.44) | 1.72 (1.63 to 1.82) |
| Saint Vincent and the Grenadines | Acute myeloid leukemia | death | 0 (0 to 0) | 0.02 (0.01 to 0.03) | 0 (0 to 0) | 0.07 (0.03 to 0.12) | 6.14 (3.67 to 11.18) | 4.91 (4.43 to 5.39) |
| Saint Vincent and the Grenadines | Acute lymphoid leukemia | death | 0 (0 to 0) | 0.01 (0 to 0.01) | 0 (0 to 0) | 0.02 (0.01 to 0.04) | 5.21 (2.56 to 11.49) | 4.8 (3.83 to 5.78) |
| Saint Vincent and the Grenadines | Chronic myeloid leukemia | death | 0 (0 to 0) | 0.08 (0.03 to 0.17) | 0 (0 to 0) | 0.04 (0.02 to 0.07) | -0.14 (-0.38 to 0.44) | -2.59 (-3.64 to -1.53) |
| Saint Vincent and the Grenadines | Chronic lymphoid leukemia | death | 0 (0 to 0) | 0.02 (0.01 to 0.04) | 0 (0 to 0) | 0.06 (0.03 to 0.1) | 4.05 (2.6 to 7.43) | 3.39 (3.09 to 3.68) |
| Saint Vincent and the Grenadines | Other leukemia | death | 0 (0 to 0) | 0.1 (0.04 to 0.19) | 0 (0 to 0) | 0.18 (0.09 to 0.31) | 2.27 (1.44 to 4.1) | 2 (1.77 to 2.22) |
| Saint Vincent and the Grenadines | Leukemia | DALYs | 0.005 (0.002 to 0.01) | 6.9 (2.57 to 13.09) | 0.015 (0.007 to 0.026) | 11.67 (5.68 to 19.68) | 1.81 (1.12 to 3.38) | 1.69 (1.6 to 1.78) |
| Saint Vincent and the Grenadines | Acute myeloid leukemia | DALYs | 0 (0 to 0.001) | 0.58 (0.21 to 1.15) | 0.003 (0.001 to 0.006) | 2.47 (1.16 to 4.32) | 5.61 (3.32 to 10.48) | 4.86 (4.34 to 5.38) |
| Saint Vincent and the Grenadines | Acute lymphoid leukemia | DALYs | 0 (0 to 0) | 0.25 (0.09 to 0.52) | 0.001 (0.001 to 0.002) | 0.96 (0.42 to 1.69) | 4.37 (2.08 to 10.34) | 4.59 (3.64 to 5.54) |
| Saint Vincent and the Grenadines | Chronic myeloid leukemia | DALYs | 0.002 (0.001 to 0.004) | 2.57 (0.88 to 5.09) | 0.002 (0.001 to 0.003) | 1.28 (0.61 to 2.21) | -0.16 (-0.4 to 0.45) | -2.59 (-3.57 to -1.6) |
| Saint Vincent and the Grenadines | Chronic lymphoid leukemia | DALYs | 0 (0 to 0.001) | 0.5 (0.18 to 0.98) | 0.002 (0.001 to 0.003) | 1.35 (0.65 to 2.33) | 4.05 (2.56 to 7.26) | 3.26 (2.93 to 3.6) |
| Saint Vincent and the Grenadines | Other leukemia | DALYs | 0.002 (0.001 to 0.004) | 3 (1.18 to 5.62) | 0.007 (0.004 to 0.012) | 5.61 (2.76 to 9.4) | 2.1 (1.25 to 3.96) | 1.86 (1.58 to 2.14) |
| Samoa | Leukemia | death | 0 (0 to 0.001) | 0.47 (0.22 to 0.8) | 0.001 (0 to 0.001) | 0.44 (0.19 to 0.78) | 0.52 (0.06 to 1.18) | -0.42 (-0.5 to -0.34) |
| Samoa | Acute myeloid leukemia | death | 0 (0 to 0) | 0.1 (0.05 to 0.18) | 0 (0 to 0) | 0.1 (0.05 to 0.18) | 0.57 (0.01 to 1.39) | -0.35 (-0.44 to -0.25) |
| Samoa | Acute lymphoid leukemia | death | 0 (0 to 0) | 0.03 (0.01 to 0.05) | 0 (0 to 0) | 0.03 (0.01 to 0.05) | 0.57 (-0.03 to 1.46) | -0.27 (-0.38 to -0.15) |
| Samoa | Chronic myeloid leukemia | death | 0 (0 to 0) | 0.09 (0.04 to 0.16) | 0 (0 to 0) | 0.07 (0.03 to 0.14) | 0.34 (-0.15 to 1.05) | -1.04 (-1.25 to -0.84) |
| Samoa | Chronic lymphoid leukemia | death | 0 (0 to 0) | 0.01 (0 to 0.02) | 0 (0 to 0) | 0.01 (0 to 0.02) | 0.59 (0.01 to 1.49) | -0.49 (-0.61 to -0.37) |
| Samoa | Other leukemia | death | 0 (0 to 0) | 0.24 (0.11 to 0.41) | 0 (0 to 0.001) | 0.23 (0.1 to 0.42) | 0.57 (0.1 to 1.25) | -0.25 (-0.29 to -0.21) |
| Samoa | Leukemia | DALYs | 0.016 (0.007 to 0.027) | 14.79 (7.08 to 25) | 0.024 (0.011 to 0.041) | 13.89 (6.4 to 24.18) | 0.5 (-0.02 to 1.24) | -0.42 (-0.52 to -0.32) |
| Samoa | Acute myeloid leukemia | DALYs | 0.004 (0.002 to 0.007) | 3.66 (1.7 to 6.56) | 0.006 (0.003 to 0.012) | 3.57 (1.64 to 6.59) | 0.54 (-0.06 to 1.44) | -0.33 (-0.43 to -0.22) |
| Samoa | Acute lymphoid leukemia | DALYs | 0.001 (0.001 to 0.003) | 1.12 (0.5 to 2.05) | 0.002 (0.001 to 0.004) | 1.12 (0.46 to 2.15) | 0.54 (-0.12 to 1.57) | -0.2 (-0.35 to -0.04) |
| Samoa | Chronic myeloid leukemia | DALYs | 0.003 (0.001 to 0.005) | 2.72 (1.23 to 5.02) | 0.004 (0.002 to 0.007) | 2.24 (0.94 to 4.21) | 0.33 (-0.17 to 1.11) | -1.02 (-1.26 to -0.79) |
| Samoa | Chronic lymphoid leukemia | DALYs | 0 (0 to 0) | 0.26 (0.12 to 0.48) | 0 (0 to 0.001) | 0.26 (0.11 to 0.49) | 0.63 (0.02 to 1.55) | -0.33 (-0.45 to -0.21) |
| Samoa | Other leukemia | DALYs | 0.007 (0.003 to 0.012) | 7.03 (3.33 to 12.03) | 0.011 (0.005 to 0.02) | 6.7 (2.98 to 11.98) | 0.53 (0 to 1.33) | -0.28 (-0.33 to -0.23) |
| San Marino | Leukemia | death | 0 (0 to 0) | 0.63 (0.29 to 1.08) | 0 (0 to 0.001) | 0.69 (0.3 to 1.32) | 1.25 (0.43 to 2.42) | 0.69 (0.56 to 0.82) |
| San Marino | Acute myeloid leukemia | death | 0 (0 to 0) | 0.14 (0.06 to 0.24) | 0 (0 to 0) | 0.18 (0.07 to 0.35) | 1.54 (0.53 to 3.19) | 1.36 (1.15 to 1.56) |
| San Marino | Acute lymphoid leukemia | death | 0 (0 to 0) | 0.11 (0.05 to 0.21) | 0 (0 to 0) | 0.14 (0.05 to 0.28) | 1.27 (0.37 to 2.8) | 1.09 (0.91 to 1.27) |
| San Marino | Chronic myeloid leukemia | death | 0 (0 to 0) | 0.04 (0.02 to 0.08) | 0 (0 to 0) | 0.03 (0.01 to 0.05) | 0.35 (-0.2 to 1.2) | -1.32 (-1.45 to -1.19) |
| San Marino | Chronic lymphoid leukemia | death | 0 (0 to 0) | 0.01 (0 to 0.01) | 0 (0 to 0) | 0.01 (0 to 0.01) | 1.18 (0.3 to 2.68) | 0.91 (0.65 to 1.17) |
| San Marino | Other leukemia | death | 0 (0 to 0) | 0.33 (0.14 to 0.58) | 0 (0 to 0) | 0.34 (0.14 to 0.66) | 1.24 (0.41 to 2.41) | 0.43 (0.33 to 0.54) |
| San Marino | Leukemia | DALYs | 0.005 (0.002 to 0.008) | 14.66 (6.72 to 25.04) | 0.009 (0.004 to 0.018) | 17.07 (7.57 to 32.84) | 1.03 (0.27 to 2.2) | 0.82 (0.71 to 0.94) |
| San Marino | Acute myeloid leukemia | DALYs | 0.001 (0 to 0.002) | 3.14 (1.39 to 5.47) | 0.002 (0.001 to 0.004) | 4.13 (1.69 to 8.06) | 1.29 (0.36 to 2.88) | 1.32 (1.13 to 1.51) |
| San Marino | Acute lymphoid leukemia | DALYs | 0.001 (0 to 0.002) | 3.54 (1.47 to 6.63) | 0.002 (0.001 to 0.005) | 5.16 (2.1 to 10) | 1.3 (0.43 to 2.88) | 1.64 (1.46 to 1.83) |
| San Marino | Chronic myeloid leukemia | DALYs | 0 (0 to 0.001) | 1.05 (0.47 to 1.82) | 0 (0 to 0.001) | 0.74 (0.31 to 1.41) | 0.3 (-0.23 to 1.12) | -0.99 (-1.11 to -0.87) |
| San Marino | Chronic lymphoid leukemia | DALYs | 0 (0 to 0) | 0.15 (0.06 to 0.26) | 0 (0 to 0) | 0.18 (0.08 to 0.36) | 1.14 (0.24 to 2.61) | 1.2 (0.97 to 1.43) |
| San Marino | Other leukemia | DALYs | 0.002 (0.001 to 0.004) | 6.78 (2.95 to 12.06) | 0.004 (0.002 to 0.008) | 6.86 (2.85 to 13.28) | 0.88 (0.16 to 1.99) | 0.25 (0.16 to 0.34) |
| Sao Tome and Principe | Leukemia | death | 0 (0 to 0) | 0.11 (0.04 to 0.21) | 0 (0 to 0) | 0.2 (0.1 to 0.37) | 2.47 (1.38 to 4.77) | 2.26 (2.12 to 2.39) |
| Sao Tome and Principe | Acute myeloid leukemia | death | 0 (0 to 0) | 0.02 (0.01 to 0.04) | 0 (0 to 0) | 0.04 (0.02 to 0.07) | 3 (1.53 to 6.45) | 2.71 (2.62 to 2.79) |
| Sao Tome and Principe | Acute lymphoid leukemia | death | 0 (0 to 0) | 0.01 (0 to 0.01) | 0 (0 to 0) | 0.01 (0 to 0.02) | 3.22 (1.57 to 7.82) | 2.33 (2.18 to 2.49) |
| Sao Tome and Principe | Chronic myeloid leukemia | death | 0 (0 to 0) | 0.02 (0.01 to 0.04) | 0 (0 to 0) | 0.03 (0.02 to 0.06) | 2.7 (1.35 to 5.79) | 1.79 (1.5 to 2.07) |
| Sao Tome and Principe | Chronic lymphoid leukemia | death | 0 (0 to 0) | 0.02 (0.01 to 0.05) | 0 (0 to 0) | 0.04 (0.02 to 0.08) | 1.97 (0.94 to 4.26) | 2.42 (2.3 to 2.53) |
| Sao Tome and Principe | Other leukemia | death | 0 (0 to 0) | 0.04 (0.01 to 0.09) | 0 (0 to 0) | 0.08 (0.03 to 0.14) | 2.25 (1.09 to 4.95) | 2.16 (2.03 to 2.3) |
| Sao Tome and Principe | Leukemia | DALYs | 0.002 (0.001 to 0.005) | 3.24 (1.17 to 6.68) | 0.009 (0.004 to 0.016) | 6.23 (3 to 11.37) | 2.82 (1.53 to 5.8) | 2.2 (2 to 2.39) |
| Sao Tome and Principe | Acute myeloid leukemia | DALYs | 0 (0 to 0.001) | 0.62 (0.18 to 1.35) | 0.002 (0.001 to 0.004) | 1.32 (0.55 to 2.52) | 3.47 (1.77 to 7.67) | 2.69 (2.57 to 2.82) |
| Sao Tome and Principe | Acute lymphoid leukemia | DALYs | 0 (0 to 0) | 0.24 (0.06 to 0.58) | 0.001 (0 to 0.002) | 0.48 (0.18 to 0.97) | 3.5 (1.6 to 9.05) | 2.32 (2.1 to 2.55) |
| Sao Tome and Principe | Chronic myeloid leukemia | DALYs | 0 (0 to 0.001) | 0.69 (0.24 to 1.44) | 0.002 (0.001 to 0.004) | 1.19 (0.51 to 2.3) | 2.84 (1.33 to 6.16) | 1.57 (1.22 to 1.92) |
| Sao Tome and Principe | Chronic lymphoid leukemia | DALYs | 0 (0 to 0.001) | 0.52 (0.17 to 1.08) | 0.001 (0 to 0.002) | 1.05 (0.46 to 1.92) | 2.06 (0.92 to 4.44) | 2.3 (2.16 to 2.45) |
| Sao Tome and Principe | Other leukemia | DALYs | 0.001 (0 to 0.002) | 1.16 (0.4 to 2.4) | 0.003 (0.001 to 0.005) | 2.19 (0.94 to 4.1) | 2.63 (1.28 to 5.69) | 2.22 (2.03 to 2.41) |
| Saudi Arabia | Leukemia | death | 0.021 (0.01 to 0.038) | 0.3 (0.14 to 0.55) | 0.104 (0.055 to 0.171) | 0.45 (0.24 to 0.73) | 4 (2.29 to 7.51) | 0.95 (0.51 to 1.39) |
| Saudi Arabia | Acute myeloid leukemia | death | 0.005 (0.002 to 0.01) | 0.07 (0.03 to 0.14) | 0.033 (0.017 to 0.055) | 0.13 (0.06 to 0.21) | 5.13 (2.77 to 9.8) | 1.18 (0.84 to 1.52) |
| Saudi Arabia | Acute lymphoid leukemia | death | 0.003 (0.001 to 0.005) | 0.03 (0.01 to 0.05) | 0.013 (0.006 to 0.022) | 0.04 (0.02 to 0.07) | 4.02 (2 to 8.56) | 0.58 (0.22 to 0.95) |
| Saudi Arabia | Chronic myeloid leukemia | death | 0.005 (0.002 to 0.01) | 0.07 (0.03 to 0.14) | 0.024 (0.013 to 0.039) | 0.09 (0.05 to 0.14) | 3.61 (1.69 to 7.37) | 0.42 (0.17 to 0.66) |
| Saudi Arabia | Chronic lymphoid leukemia | death | 0.002 (0.001 to 0.004) | 0.04 (0.02 to 0.08) | 0.013 (0.007 to 0.021) | 0.1 (0.05 to 0.16) | 5.66 (2.98 to 12.01) | 2.34 (1.98 to 2.7) |
| Saudi Arabia | Other leukemia | death | 0.006 (0.002 to 0.016) | 0.09 (0.03 to 0.24) | 0.021 (0.008 to 0.062) | 0.1 (0.04 to 0.27) | 2.7 (0.98 to 8.39) | 0.41 (-0.6 to 1.44) |
| Saudi Arabia | Leukemia | DALYs | 0.756 (0.339 to 1.358) | 8.51 (3.91 to 15.31) | 4.143 (2.208 to 6.848) | 12.96 (6.79 to 20.96) | 4.48 (2.49 to 8.4) | 1.07 (0.73 to 1.41) |
| Saudi Arabia | Acute myeloid leukemia | DALYs | 0.2 (0.082 to 0.38) | 2.21 (0.87 to 4.19) | 1.331 (0.686 to 2.249) | 3.9 (1.94 to 6.47) | 5.67 (3.08 to 10.79) | 1.3 (0.99 to 1.6) |
| Saudi Arabia | Acute lymphoid leukemia | DALYs | 0.117 (0.045 to 0.229) | 1.02 (0.42 to 1.94) | 0.623 (0.289 to 1.065) | 1.54 (0.75 to 2.62) | 4.32 (2.16 to 9.3) | 0.86 (0.61 to 1.11) |
| Saudi Arabia | Chronic myeloid leukemia | DALYs | 0.195 (0.089 to 0.36) | 2.14 (0.97 to 3.97) | 1.003 (0.53 to 1.616) | 2.79 (1.5 to 4.47) | 4.14 (1.99 to 8.43) | 0.65 (0.52 to 0.77) |
| Saudi Arabia | Chronic lymphoid leukemia | DALYs | 0.046 (0.02 to 0.088) | 0.81 (0.34 to 1.53) | 0.374 (0.199 to 0.604) | 2.03 (1.07 to 3.25) | 7.09 (3.75 to 15.22) | 2.63 (2.33 to 2.94) |
| Saudi Arabia | Other leukemia | DALYs | 0.198 (0.057 to 0.553) | 2.33 (0.68 to 6.52) | 0.812 (0.318 to 2.449) | 2.7 (1.1 to 7.77) | 3.09 (1.21 to 9.37) | 0.54 (-0.4 to 1.48) |
| Senegal | Leukemia | death | 0.004 (0.001 to 0.007) | 0.1 (0.04 to 0.21) | 0.015 (0.006 to 0.027) | 0.18 (0.08 to 0.33) | 2.95 (1.71 to 5.19) | 2.14 (1.95 to 2.33) |
| Senegal | Acute myeloid leukemia | death | 0.001 (0 to 0.001) | 0.02 (0.01 to 0.03) | 0.003 (0.001 to 0.005) | 0.03 (0.01 to 0.06) | 3.51 (1.77 to 6.62) | 2.79 (2.56 to 3.03) |
| Senegal | Acute lymphoid leukemia | death | 0 (0 to 0.001) | 0.01 (0 to 0.01) | 0.001 (0 to 0.002) | 0.01 (0 to 0.02) | 2.83 (1.28 to 5.6) | 1.76 (1.56 to 1.96) |
| Senegal | Chronic myeloid leukemia | death | 0.001 (0 to 0.002) | 0.02 (0.01 to 0.04) | 0.002 (0.001 to 0.005) | 0.03 (0.01 to 0.05) | 2.03 (0.9 to 3.98) | 1.03 (0.84 to 1.21) |
| Senegal | Chronic lymphoid leukemia | death | 0.001 (0 to 0.001) | 0.02 (0.01 to 0.04) | 0.003 (0.001 to 0.005) | 0.04 (0.02 to 0.07) | 3.29 (1.79 to 6.57) | 2.45 (2.24 to 2.65) |
| Senegal | Other leukemia | death | 0.001 (0.001 to 0.003) | 0.04 (0.02 to 0.09) | 0.006 (0.002 to 0.011) | 0.08 (0.03 to 0.14) | 3.08 (1.7 to 5.69) | 2.25 (2.06 to 2.43) |
| Senegal | Leukemia | DALYs | 0.127 (0.049 to 0.251) | 3.12 (1.2 to 6.19) | 0.484 (0.206 to 0.886) | 5.16 (2.25 to 9.47) | 2.83 (1.56 to 5.02) | 1.94 (1.73 to 2.15) |
| Senegal | Acute myeloid leukemia | DALYs | 0.023 (0.008 to 0.048) | 0.51 (0.18 to 1.07) | 0.097 (0.038 to 0.189) | 0.98 (0.38 to 1.9) | 3.26 (1.62 to 6.1) | 2.46 (2.23 to 2.69) |
| Senegal | Acute lymphoid leukemia | DALYs | 0.011 (0.003 to 0.024) | 0.22 (0.07 to 0.47) | 0.042 (0.015 to 0.087) | 0.35 (0.13 to 0.72) | 2.9 (1.22 to 6.15) | 1.81 (1.6 to 2.02) |
| Senegal | Chronic myeloid leukemia | DALYs | 0.032 (0.012 to 0.068) | 0.73 (0.27 to 1.54) | 0.093 (0.037 to 0.186) | 0.91 (0.36 to 1.79) | 1.94 (0.77 to 3.87) | 0.79 (0.58 to 0.99) |
| Senegal | Chronic lymphoid leukemia | DALYs | 0.015 (0.005 to 0.032) | 0.47 (0.15 to 0.98) | 0.065 (0.026 to 0.124) | 0.86 (0.35 to 1.63) | 3.21 (1.66 to 6.76) | 2.3 (2.09 to 2.52) |
| Senegal | Other leukemia | DALYs | 0.046 (0.016 to 0.094) | 1.19 (0.44 to 2.44) | 0.187 (0.077 to 0.353) | 2.06 (0.85 to 3.81) | 3.09 (1.6 to 5.73) | 2.18 (1.97 to 2.39) |
| Serbia | Leukemia | death | 0.047 (0.022 to 0.079) | 0.42 (0.19 to 0.71) | 0.082 (0.042 to 0.135) | 0.53 (0.27 to 0.87) | 0.75 (0.32 to 1.41) | 1.01 (0.86 to 1.16) |
| Serbia | Acute myeloid leukemia | death | 0.008 (0.004 to 0.014) | 0.07 (0.03 to 0.12) | 0.017 (0.008 to 0.028) | 0.12 (0.06 to 0.2) | 1.03 (0.44 to 1.97) | 2.31 (2.01 to 2.61) |
| Serbia | Acute lymphoid leukemia | death | 0.003 (0.001 to 0.005) | 0.02 (0.01 to 0.05) | 0.005 (0.002 to 0.009) | 0.04 (0.02 to 0.06) | 0.83 (0.21 to 1.71) | 1.79 (1.55 to 2.03) |
| Serbia | Chronic myeloid leukemia | death | 0.002 (0.001 to 0.004) | 0.02 (0.01 to 0.03) | 0.004 (0.002 to 0.006) | 0.03 (0.01 to 0.04) | 0.81 (0.16 to 1.86) | 1.58 (1.14 to 2.03) |
| Serbia | Chronic lymphoid leukemia | death | 0.002 (0.001 to 0.004) | 0.02 (0.01 to 0.04) | 0.008 (0.004 to 0.013) | 0.05 (0.02 to 0.08) | 2.07 (1.07 to 3.63) | 3.34 (2.96 to 3.72) |
| Serbia | Other leukemia | death | 0.031 (0.015 to 0.053) | 0.28 (0.13 to 0.48) | 0.049 (0.024 to 0.082) | 0.3 (0.15 to 0.51) | 0.56 (0.16 to 1.18) | 0.24 (0.13 to 0.34) |
| Serbia | Leukemia | DALYs | 1.326 (0.62 to 2.238) | 11.59 (5.29 to 19.64) | 1.935 (0.987 to 3.214) | 13.56 (6.84 to 22.82) | 0.46 (0.09 to 1) | 0.67 (0.54 to 0.79) |
| Serbia | Acute myeloid leukemia | DALYs | 0.264 (0.123 to 0.451) | 2.3 (1.05 to 3.98) | 0.461 (0.232 to 0.782) | 3.58 (1.77 to 6.2) | 0.75 (0.24 to 1.54) | 2.07 (1.81 to 2.33) |
| Serbia | Acute lymphoid leukemia | DALYs | 0.095 (0.041 to 0.179) | 0.87 (0.37 to 1.67) | 0.153 (0.072 to 0.271) | 1.24 (0.57 to 2.21) | 0.61 (0.06 to 1.42) | 1.61 (1.43 to 1.79) |
| Serbia | Chronic myeloid leukemia | DALYs | 0.065 (0.029 to 0.115) | 0.57 (0.25 to 1.01) | 0.097 (0.049 to 0.164) | 0.71 (0.35 to 1.18) | 0.5 (-0.02 to 1.4) | 1.21 (0.78 to 1.65) |
| Serbia | Chronic lymphoid leukemia | DALYs | 0.069 (0.03 to 0.121) | 0.6 (0.26 to 1.07) | 0.182 (0.092 to 0.307) | 1.22 (0.6 to 2.07) | 1.65 (0.79 to 3.01) | 3.03 (2.7 to 3.36) |
| Serbia | Other leukemia | DALYs | 0.835 (0.384 to 1.405) | 7.25 (3.32 to 12.38) | 1.042 (0.525 to 1.769) | 6.81 (3.42 to 11.45) | 0.25 (-0.08 to 0.75) | -0.36 (-0.5 to -0.22) |
| Seychelles | Leukemia | death | 0 (0 to 0) | 0.34 (0.14 to 0.62) | 0 (0 to 0.001) | 0.38 (0.19 to 0.68) | 1.23 (0.8 to 2.02) | 0.22 (0.12 to 0.32) |
| Seychelles | Acute myeloid leukemia | death | 0 (0 to 0) | 0.03 (0.01 to 0.06) | 0 (0 to 0) | 0.05 (0.02 to 0.08) | 2.21 (1.25 to 4.09) | 1.28 (1.14 to 1.42) |
| Seychelles | Acute lymphoid leukemia | death | 0 (0 to 0) | 0.02 (0.01 to 0.03) | 0 (0 to 0) | 0.03 (0.01 to 0.05) | 2.13 (1.22 to 4.17) | 1.39 (1.22 to 1.56) |
| Seychelles | Chronic myeloid leukemia | death | 0 (0 to 0) | 0.07 (0.03 to 0.13) | 0 (0 to 0) | 0.06 (0.03 to 0.1) | 0.65 (0.17 to 1.55) | -0.74 (-0.8 to -0.67) |
| Seychelles | Chronic lymphoid leukemia | death | 0 (0 to 0) | 0.17 (0.06 to 0.32) | 0 (0 to 0) | 0.19 (0.08 to 0.35) | 1.26 (0.77 to 2.1) | 0.29 (0.17 to 0.4) |
| Seychelles | Other leukemia | death | 0 (0 to 0) | 0.06 (0.02 to 0.12) | 0 (0 to 0) | 0.06 (0.03 to 0.12) | 1.07 (0.48 to 2.47) | -0.05 (-0.16 to 0.06) |
| Seychelles | Leukemia | DALYs | 0.007 (0.003 to 0.012) | 11.38 (4.8 to 20.39) | 0.015 (0.007 to 0.025) | 12.37 (6.21 to 20.97) | 1.17 (0.73 to 1.96) | 0.1 (0.01 to 0.19) |
| Seychelles | Acute myeloid leukemia | DALYs | 0.001 (0 to 0.001) | 1.11 (0.47 to 2.08) | 0.002 (0.001 to 0.004) | 1.74 (0.84 to 3.04) | 2.03 (1.08 to 3.85) | 1.18 (1.04 to 1.31) |
| Seychelles | Acute lymphoid leukemia | DALYs | 0 (0 to 0.001) | 0.71 (0.26 to 1.43) | 0.001 (0.001 to 0.003) | 1.23 (0.57 to 2.25) | 2.02 (1.13 to 3.99) | 1.48 (1.31 to 1.66) |
| Seychelles | Chronic myeloid leukemia | DALYs | 0.002 (0.001 to 0.003) | 2.83 (1.14 to 5.37) | 0.003 (0.001 to 0.005) | 2.29 (1.06 to 4.12) | 0.54 (0.07 to 1.41) | -0.77 (-0.84 to -0.71) |
| Seychelles | Chronic lymphoid leukemia | DALYs | 0.003 (0.001 to 0.005) | 4.61 (1.76 to 8.46) | 0.006 (0.003 to 0.011) | 4.94 (2.15 to 8.99) | 1.31 (0.77 to 2.13) | 0.09 (-0.03 to 0.22) |
| Seychelles | Other leukemia | DALYs | 0.001 (0.001 to 0.003) | 2.12 (0.83 to 4.12) | 0.003 (0.001 to 0.005) | 2.17 (1.04 to 3.87) | 0.97 (0.39 to 2.3) | -0.18 (-0.28 to -0.08) |
| Sierra Leone | Leukemia | death | 0.001 (0 to 0.003) | 0.06 (0.02 to 0.14) | 0.005 (0.002 to 0.01) | 0.12 (0.04 to 0.25) | 3.14 (1.67 to 7.3) | 2.79 (2.64 to 2.94) |
| Sierra Leone | Acute myeloid leukemia | death | 0 (0 to 0) | 0.01 (0 to 0.02) | 0.001 (0 to 0.002) | 0.02 (0.01 to 0.04) | 3.76 (1.89 to 9.46) | 3.27 (2.96 to 3.58) |
| Sierra Leone | Acute lymphoid leukemia | death | 0 (0 to 0) | 0 (0 to 0.01) | 0 (0 to 0.001) | 0.01 (0 to 0.01) | 3.62 (1.79 to 9.02) | 2.54 (2.25 to 2.84) |
| Sierra Leone | Chronic myeloid leukemia | death | 0 (0 to 0.001) | 0.01 (0 to 0.03) | 0.001 (0 to 0.002) | 0.02 (0.01 to 0.04) | 2.97 (1.47 to 7.54) | 2.34 (2.18 to 2.51) |
| Sierra Leone | Chronic lymphoid leukemia | death | 0 (0 to 0.001) | 0.01 (0 to 0.03) | 0.001 (0 to 0.002) | 0.03 (0.01 to 0.06) | 2.95 (1.43 to 7.75) | 3.06 (2.94 to 3.17) |
| Sierra Leone | Other leukemia | death | 0 (0 to 0.001) | 0.02 (0.01 to 0.06) | 0.002 (0.001 to 0.004) | 0.05 (0.02 to 0.1) | 3.05 (1.55 to 7.28) | 2.71 (2.59 to 2.83) |
| Sierra Leone | Leukemia | DALYs | 0.038 (0.01 to 0.09) | 1.73 (0.46 to 4.03) | 0.172 (0.063 to 0.34) | 3.62 (1.33 to 7.11) | 3.52 (1.91 to 8.06) | 2.87 (2.7 to 3.04) |
| Sierra Leone | Acute myeloid leukemia | DALYs | 0.006 (0.001 to 0.015) | 0.25 (0.06 to 0.62) | 0.029 (0.01 to 0.063) | 0.58 (0.19 to 1.2) | 3.97 (2.06 to 9.9) | 3.14 (2.82 to 3.47) |
| Sierra Leone | Acute lymphoid leukemia | DALYs | 0.003 (0.001 to 0.007) | 0.1 (0.02 to 0.27) | 0.014 (0.004 to 0.032) | 0.22 (0.06 to 0.49) | 4.07 (1.98 to 9.97) | 2.76 (2.5 to 3.03) |
| Sierra Leone | Chronic myeloid leukemia | DALYs | 0.009 (0.002 to 0.023) | 0.39 (0.1 to 0.96) | 0.04 (0.015 to 0.081) | 0.74 (0.28 to 1.48) | 3.32 (1.66 to 8.33) | 2.5 (2.34 to 2.67) |
| Sierra Leone | Chronic lymphoid leukemia | DALYs | 0.006 (0.001 to 0.013) | 0.29 (0.07 to 0.68) | 0.023 (0.008 to 0.046) | 0.65 (0.22 to 1.3) | 3.18 (1.53 to 8.4) | 3.13 (3.01 to 3.26) |
| Sierra Leone | Other leukemia | DALYs | 0.015 (0.003 to 0.036) | 0.69 (0.16 to 1.66) | 0.066 (0.023 to 0.137) | 1.44 (0.49 to 2.94) | 3.51 (1.83 to 8.23) | 2.87 (2.73 to 3) |
| Singapore | Leukemia | death | 0.003 (0.001 to 0.006) | 0.11 (0.03 to 0.25) | 0.01 (0.005 to 0.018) | 0.13 (0.06 to 0.24) | 2.65 (1.69 to 5.82) | 0.42 (0.18 to 0.66) |
| Singapore | Acute myeloid leukemia | death | 0.001 (0 to 0.003) | 0.06 (0.02 to 0.13) | 0.007 (0.003 to 0.012) | 0.09 (0.04 to 0.16) | 3.86 (2.56 to 8.07) | 1.44 (1.11 to 1.77) |
| Singapore | Acute lymphoid leukemia | death | 0 (0 to 0.001) | 0.01 (0 to 0.03) | 0.001 (0 to 0.002) | 0.01 (0.01 to 0.02) | 1.79 (0.89 to 4.66) | 0.01 (-0.16 to 0.17) |
| Singapore | Chronic myeloid leukemia | death | 0 (0 to 0.001) | 0.02 (0 to 0.04) | 0.001 (0 to 0.001) | 0.01 (0 to 0.01) | 0.36 (-0.02 to 1.61) | -3.47 (-3.91 to -3.03) |
| Singapore | Chronic lymphoid leukemia | death | 0 (0 to 0) | 0 (0 to 0.01) | 0.001 (0 to 0.001) | 0.01 (0 to 0.01) | 4.64 (2.97 to 9.62) | 1.33 (0.92 to 1.74) |
| Singapore | Other leukemia | death | 0 (0 to 0.001) | 0.02 (0.01 to 0.05) | 0.001 (0.001 to 0.002) | 0.02 (0.01 to 0.03) | 1.43 (0.77 to 3.56) | -1.15 (-1.28 to -1.02) |
| Singapore | Leukemia | DALYs | 0.099 (0.027 to 0.218) | 3.39 (0.94 to 7.44) | 0.29 (0.138 to 0.504) | 3.72 (1.75 to 6.52) | 1.95 (1.15 to 4.67) | 0.13 (-0.07 to 0.33) |
| Singapore | Acute myeloid leukemia | DALYs | 0.048 (0.013 to 0.106) | 1.7 (0.47 to 3.68) | 0.185 (0.087 to 0.321) | 2.35 (1.09 to 4.1) | 2.82 (1.78 to 6.34) | 1.01 (0.71 to 1.3) |
| Singapore | Acute lymphoid leukemia | DALYs | 0.016 (0.004 to 0.037) | 0.48 (0.13 to 1.09) | 0.047 (0.022 to 0.082) | 0.63 (0.29 to 1.11) | 1.84 (0.86 to 5.01) | 0.62 (0.46 to 0.78) |
| Singapore | Chronic myeloid leukemia | DALYs | 0.017 (0.005 to 0.037) | 0.55 (0.15 to 1.2) | 0.019 (0.009 to 0.034) | 0.25 (0.11 to 0.43) | 0.18 (-0.18 to 1.36) | -3.53 (-3.94 to -3.12) |
| Singapore | Chronic lymphoid leukemia | DALYs | 0.002 (0.001 to 0.005) | 0.1 (0.03 to 0.22) | 0.011 (0.005 to 0.021) | 0.15 (0.07 to 0.27) | 3.96 (2.43 to 8.36) | 1.15 (0.75 to 1.55) |
| Singapore | Other leukemia | DALYs | 0.015 (0.004 to 0.033) | 0.56 (0.15 to 1.24) | 0.027 (0.013 to 0.049) | 0.35 (0.16 to 0.63) | 0.85 (0.32 to 2.52) | -1.68 (-1.85 to -1.5) |
| Slovakia | Leukemia | death | 0.027 (0.013 to 0.045) | 0.45 (0.22 to 0.74) | 0.043 (0.022 to 0.073) | 0.47 (0.24 to 0.8) | 0.6 (0.15 to 1.16) | 0.22 (0.11 to 0.33) |
| Slovakia | Acute myeloid leukemia | death | 0.011 (0.006 to 0.02) | 0.19 (0.09 to 0.33) | 0.019 (0.009 to 0.034) | 0.21 (0.11 to 0.37) | 0.69 (0.2 to 1.38) | 0.34 (0.16 to 0.52) |
| Slovakia | Acute lymphoid leukemia | death | 0.002 (0.001 to 0.004) | 0.04 (0.02 to 0.07) | 0.003 (0.001 to 0.005) | 0.04 (0.02 to 0.06) | 0.36 (-0.14 to 1.07) | -0.3 (-0.42 to -0.18) |
| Slovakia | Chronic myeloid leukemia | death | 0.003 (0.002 to 0.006) | 0.06 (0.03 to 0.1) | 0.002 (0.001 to 0.003) | 0.02 (0.01 to 0.04) | -0.41 (-0.61 to -0.1) | -3.78 (-4.07 to -3.48) |
| Slovakia | Chronic lymphoid leukemia | death | 0.006 (0.003 to 0.011) | 0.11 (0.05 to 0.18) | 0.013 (0.007 to 0.022) | 0.14 (0.07 to 0.23) | 1 (0.39 to 1.91) | 0.93 (0.78 to 1.08) |
| Slovakia | Other leukemia | death | 0.003 (0.002 to 0.007) | 0.06 (0.03 to 0.11) | 0.006 (0.003 to 0.011) | 0.07 (0.03 to 0.12) | 0.72 (-0.13 to 1.82) | 1 (0.7 to 1.29) |
| Slovakia | Leukemia | DALYs | 0.7 (0.346 to 1.162) | 11.82 (5.81 to 19.76) | 1.022 (0.52 to 1.715) | 11.72 (5.94 to 19.86) | 0.46 (0.05 to 1) | -0.02 (-0.14 to 0.11) |
| Slovakia | Acute myeloid leukemia | DALYs | 0.313 (0.151 to 0.535) | 5.3 (2.53 to 9.05) | 0.488 (0.245 to 0.841) | 5.69 (2.86 to 9.77) | 0.56 (0.11 to 1.23) | 0.19 (0 to 0.37) |
| Slovakia | Acute lymphoid leukemia | DALYs | 0.07 (0.03 to 0.136) | 1.21 (0.52 to 2.37) | 0.095 (0.045 to 0.17) | 1.24 (0.58 to 2.24) | 0.36 (-0.12 to 1.08) | -0.08 (-0.2 to 0.04) |
| Slovakia | Chronic myeloid leukemia | DALYs | 0.089 (0.04 to 0.156) | 1.5 (0.68 to 2.66) | 0.045 (0.021 to 0.08) | 0.51 (0.24 to 0.9) | -0.49 (-0.68 to -0.2) | -4.25 (-4.58 to -3.91) |
| Slovakia | Chronic lymphoid leukemia | DALYs | 0.144 (0.07 to 0.247) | 2.38 (1.17 to 4.05) | 0.271 (0.137 to 0.464) | 2.87 (1.44 to 4.9) | 0.88 (0.27 to 1.75) | 0.7 (0.53 to 0.87) |
| Slovakia | Other leukemia | DALYs | 0.085 (0.038 to 0.163) | 1.44 (0.65 to 2.76) | 0.123 (0.057 to 0.219) | 1.41 (0.65 to 2.52) | 0.46 (-0.23 to 1.4) | 0.55 (0.28 to 0.82) |
| Slovenia | Leukemia | death | 0.011 (0.006 to 0.02) | 0.47 (0.23 to 0.83) | 0.021 (0.011 to 0.036) | 0.48 (0.25 to 0.82) | 0.87 (0.27 to 1.67) | 0.18 (0.06 to 0.3) |
| Slovenia | Acute myeloid leukemia | death | 0.003 (0.001 to 0.005) | 0.11 (0.05 to 0.2) | 0.01 (0.004 to 0.017) | 0.23 (0.11 to 0.41) | 2.68 (1.09 to 4.59) | 3.3 (2.88 to 3.71) |
| Slovenia | Acute lymphoid leukemia | death | 0.001 (0 to 0.001) | 0.03 (0.02 to 0.06) | 0.001 (0 to 0.002) | 0.02 (0.01 to 0.05) | 0.11 (-0.38 to 1.44) | -2.36 (-2.87 to -1.85) |
| Slovenia | Chronic myeloid leukemia | death | 0.002 (0.001 to 0.003) | 0.08 (0.04 to 0.14) | 0.002 (0.001 to 0.003) | 0.04 (0.02 to 0.07) | -0.11 (-0.44 to 0.45) | -2.88 (-3.2 to -2.57) |
| Slovenia | Chronic lymphoid leukemia | death | 0.004 (0.002 to 0.007) | 0.16 (0.08 to 0.28) | 0.007 (0.003 to 0.012) | 0.14 (0.07 to 0.24) | 0.72 (0.13 to 1.65) | -0.91 (-1.12 to -0.7) |
| Slovenia | Other leukemia | death | 0.002 (0.001 to 0.004) | 0.1 (0.05 to 0.17) | 0.003 (0.001 to 0.005) | 0.05 (0.03 to 0.1) | 0.1 (-0.29 to 0.72) | -1.47 (-1.76 to -1.18) |
| Slovenia | Leukemia | DALYs | 0.281 (0.136 to 0.498) | 11.72 (5.63 to 20.82) | 0.431 (0.225 to 0.732) | 11.18 (5.78 to 18.96) | 0.53 (0.03 to 1.24) | -0.14 (-0.26 to -0.01) |
| Slovenia | Acute myeloid leukemia | DALYs | 0.073 (0.034 to 0.134) | 3.08 (1.43 to 5.69) | 0.204 (0.097 to 0.355) | 5.51 (2.65 to 9.5) | 1.77 (0.69 to 3.24) | 2.54 (2.17 to 2.92) |
| Slovenia | Acute lymphoid leukemia | DALYs | 0.026 (0.012 to 0.049) | 1.13 (0.52 to 2.16) | 0.027 (0.012 to 0.064) | 0.87 (0.37 to 1.98) | 0.06 (-0.42 to 1.45) | -1.89 (-2.38 to -1.4) |
| Slovenia | Chronic myeloid leukemia | DALYs | 0.051 (0.024 to 0.092) | 2.12 (0.98 to 3.83) | 0.038 (0.018 to 0.073) | 1.04 (0.49 to 1.95) | -0.25 (-0.52 to 0.21) | -3.09 (-3.4 to -2.77) |
| Slovenia | Chronic lymphoid leukemia | DALYs | 0.078 (0.038 to 0.136) | 3.2 (1.56 to 5.58) | 0.118 (0.059 to 0.208) | 2.68 (1.35 to 4.77) | 0.51 (-0.03 to 1.31) | -1.03 (-1.24 to -0.81) |
| Slovenia | Other leukemia | DALYs | 0.052 (0.025 to 0.096) | 2.18 (1.04 to 3.96) | 0.044 (0.021 to 0.081) | 1.09 (0.51 to 2.02) | -0.16 (-0.46 to 0.34) | -1.95 (-2.24 to -1.67) |
| Solomon Islands | Leukemia | death | 0.001 (0 to 0.001) | 0.3 (0.09 to 0.68) | 0.002 (0.001 to 0.004) | 0.4 (0.13 to 0.84) | 2.12 (1.13 to 3.93) | 0.68 (0.36 to 1.01) |
| Solomon Islands | Acute myeloid leukemia | death | 0 (0 to 0) | 0.04 (0.01 to 0.11) | 0 (0 to 0.001) | 0.06 (0.02 to 0.14) | 2.54 (1.26 to 5.16) | 0.9 (0.55 to 1.26) |
| Solomon Islands | Acute lymphoid leukemia | death | 0 (0 to 0) | 0.02 (0 to 0.05) | 0 (0 to 0) | 0.03 (0.01 to 0.06) | 2.37 (1.2 to 4.61) | 0.88 (0.49 to 1.27) |
| Solomon Islands | Chronic myeloid leukemia | death | 0 (0 to 0) | 0.06 (0.01 to 0.14) | 0 (0 to 0.001) | 0.07 (0.02 to 0.16) | 1.69 (0.64 to 3.48) | 0.16 (-0.18 to 0.5) |
| Solomon Islands | Chronic lymphoid leukemia | death | 0 (0 to 0) | 0 (0 to 0.01) | 0 (0 to 0) | 0.01 (0 to 0.01) | 2.43 (1.11 to 5.17) | 0.83 (0.51 to 1.15) |
| Solomon Islands | Other leukemia | death | 0 (0 to 0.001) | 0.18 (0.04 to 0.41) | 0.001 (0 to 0.002) | 0.24 (0.07 to 0.53) | 2.12 (1.1 to 4.01) | 0.76 (0.45 to 1.07) |
| Solomon Islands | Leukemia | DALYs | 0.023 (0.006 to 0.055) | 11.17 (3.07 to 25.79) | 0.075 (0.023 to 0.168) | 14.85 (4.72 to 32.54) | 2.2 (1.16 to 4.13) | 0.75 (0.41 to 1.09) |
| Solomon Islands | Acute myeloid leukemia | DALYs | 0.004 (0.001 to 0.01) | 1.76 (0.49 to 4.33) | 0.014 (0.005 to 0.033) | 2.62 (0.89 to 5.98) | 2.61 (1.27 to 5.32) | 0.98 (0.62 to 1.33) |
| Solomon Islands | Acute lymphoid leukemia | DALYs | 0.002 (0 to 0.006) | 0.72 (0.17 to 2.32) | 0.006 (0.002 to 0.016) | 1.05 (0.3 to 2.92) | 2.47 (1.25 to 4.86) | 1.01 (0.62 to 1.4) |
| Solomon Islands | Chronic myeloid leukemia | DALYs | 0.005 (0.001 to 0.012) | 2.23 (0.52 to 5.58) | 0.013 (0.003 to 0.031) | 2.55 (0.62 to 6.15) | 1.75 (0.65 to 3.67) | 0.22 (-0.13 to 0.57) |
| Solomon Islands | Chronic lymphoid leukemia | DALYs | 0 (0 to 0.001) | 0.13 (0.04 to 0.32) | 0.001 (0 to 0.002) | 0.2 (0.07 to 0.44) | 2.6 (1.21 to 5.46) | 0.97 (0.65 to 1.3) |
| Solomon Islands | Other leukemia | DALYs | 0.013 (0.003 to 0.032) | 6.33 (1.39 to 15.08) | 0.041 (0.01 to 0.099) | 8.43 (2.21 to 19.36) | 2.2 (1.12 to 4.33) | 0.83 (0.49 to 1.16) |
| Somalia | Leukemia | death | 0.001 (0 to 0.003) | 0.03 (0.01 to 0.1) | 0.003 (0.001 to 0.009) | 0.04 (0.01 to 0.11) | 2.17 (1.09 to 4.07) | 1.05 (0.92 to 1.18) |
| Somalia | Acute myeloid leukemia | death | 0 (0 to 0) | 0 (0 to 0.01) | 0 (0 to 0.001) | 0 (0 to 0.01) | 2.92 (1.06 to 6.19) | 1.66 (1.47 to 1.86) |
| Somalia | Acute lymphoid leukemia | death | 0 (0 to 0) | 0 (0 to 0) | 0 (0 to 0.001) | 0 (0 to 0.01) | 2.75 (1.43 to 5.62) | 1.18 (1.06 to 1.31) |
| Somalia | Chronic myeloid leukemia | death | 0 (0 to 0.001) | 0.01 (0 to 0.02) | 0.001 (0 to 0.002) | 0.01 (0 to 0.02) | 1.8 (0.66 to 4.12) | 0.61 (0.44 to 0.78) |
| Somalia | Chronic lymphoid leukemia | death | 0 (0 to 0.001) | 0.01 (0 to 0.02) | 0.001 (0 to 0.002) | 0.01 (0 to 0.04) | 3.58 (1.71 to 7.53) | 2.65 (2.42 to 2.88) |
| Somalia | Other leukemia | death | 0.001 (0 to 0.001) | 0.02 (0 to 0.05) | 0.001 (0 to 0.004) | 0.02 (0 to 0.04) | 1.72 (0.81 to 3.37) | 0.15 (0.09 to 0.21) |
| Somalia | Leukemia | DALYs | 0.04 (0.007 to 0.109) | 1.07 (0.19 to 2.95) | 0.126 (0.024 to 0.341) | 1.26 (0.23 to 3.4) | 2.17 (1.09 to 4.23) | 0.97 (0.84 to 1.1) |
| Somalia | Acute myeloid leukemia | DALYs | 0.003 (0.001 to 0.01) | 0.08 (0.01 to 0.24) | 0.013 (0.002 to 0.04) | 0.11 (0.02 to 0.34) | 3.08 (1.17 to 6.52) | 1.79 (1.6 to 1.99) |
| Somalia | Acute lymphoid leukemia | DALYs | 0.002 (0 to 0.007) | 0.05 (0.01 to 0.16) | 0.009 (0.002 to 0.028) | 0.07 (0.01 to 0.21) | 3.01 (1.52 to 6.08) | 1.34 (1.21 to 1.48) |
| Somalia | Chronic myeloid leukemia | DALYs | 0.011 (0.002 to 0.03) | 0.25 (0.04 to 0.7) | 0.03 (0.006 to 0.08) | 0.26 (0.05 to 0.7) | 1.84 (0.67 to 4.26) | 0.63 (0.46 to 0.8) |
| Somalia | Chronic lymphoid leukemia | DALYs | 0.004 (0.001 to 0.013) | 0.17 (0.03 to 0.54) | 0.02 (0.003 to 0.058) | 0.3 (0.05 to 0.9) | 3.51 (1.58 to 7.53) | 2.67 (2.43 to 2.91) |
| Somalia | Other leukemia | DALYs | 0.019 (0.003 to 0.053) | 0.51 (0.08 to 1.44) | 0.054 (0.009 to 0.149) | 0.51 (0.09 to 1.4) | 1.8 (0.86 to 3.48) | 0.22 (0.14 to 0.29) |
| South Africa | Leukemia | death | 0.067 (0.032 to 0.115) | 0.3 (0.14 to 0.51) | 0.162 (0.085 to 0.258) | 0.37 (0.19 to 0.59) | 1.41 (1.06 to 1.91) | 0.91 (0.66 to 1.17) |
| South Africa | Acute myeloid leukemia | death | 0.004 (0.002 to 0.007) | 0.01 (0.01 to 0.02) | 0.011 (0.006 to 0.017) | 0.02 (0.01 to 0.03) | 1.87 (0.8 to 2.93) | 1.43 (1.31 to 1.55) |
| South Africa | Acute lymphoid leukemia | death | 0.002 (0.001 to 0.004) | 0.01 (0 to 0.01) | 0.005 (0.002 to 0.008) | 0.01 (0 to 0.01) | 1.41 (0.2 to 2.7) | 1.05 (0.83 to 1.26) |
| South Africa | Chronic myeloid leukemia | death | 0.001 (0.001 to 0.002) | 0.01 (0 to 0.01) | 0.002 (0.001 to 0.003) | 0 (0 to 0.01) | 0.45 (0.06 to 1.09) | -0.86 (-1.15 to -0.56) |
| South Africa | Chronic lymphoid leukemia | death | 0.034 (0.015 to 0.06) | 0.17 (0.07 to 0.3) | 0.09 (0.041 to 0.149) | 0.22 (0.1 to 0.37) | 1.61 (1.29 to 2.11) | 1.1 (0.81 to 1.38) |
| South Africa | Other leukemia | death | 0.026 (0.013 to 0.044) | 0.1 (0.05 to 0.18) | 0.055 (0.028 to 0.09) | 0.12 (0.06 to 0.19) | 1.11 (0.63 to 1.81) | 0.57 (0.29 to 0.84) |
| South Africa | Leukemia | DALYs | 2.266 (1.085 to 3.844) | 8.66 (4.19 to 14.55) | 4.67 (2.449 to 7.51) | 9.36 (4.88 to 14.9) | 1.06 (0.68 to 1.57) | 0.43 (0.19 to 0.68) |
| South Africa | Acute myeloid leukemia | DALYs | 0.168 (0.079 to 0.318) | 0.55 (0.26 to 1.01) | 0.459 (0.238 to 0.76) | 0.79 (0.41 to 1.3) | 1.73 (0.63 to 2.92) | 1.26 (1.12 to 1.4) |
| South Africa | Acute lymphoid leukemia | DALYs | 0.098 (0.041 to 0.221) | 0.28 (0.12 to 0.61) | 0.218 (0.099 to 0.39) | 0.36 (0.17 to 0.64) | 1.23 (0.02 to 2.71) | 0.76 (0.5 to 1.01) |
| South Africa | Chronic myeloid leukemia | DALYs | 0.056 (0.026 to 0.103) | 0.19 (0.09 to 0.35) | 0.072 (0.036 to 0.123) | 0.13 (0.07 to 0.22) | 0.27 (-0.09 to 0.94) | -1.36 (-1.67 to -1.04) |
| South Africa | Chronic lymphoid leukemia | DALYs | 0.911 (0.396 to 1.58) | 4.09 (1.76 to 7.05) | 2.135 (0.981 to 3.49) | 4.68 (2.15 to 7.69) | 1.34 (1.01 to 1.81) | 0.81 (0.54 to 1.08) |
| South Africa | Other leukemia | DALYs | 1.033 (0.498 to 1.784) | 3.55 (1.72 to 6.03) | 1.786 (0.97 to 2.931) | 3.4 (1.81 to 5.54) | 0.73 (0.28 to 1.45) | -0.18 (-0.55 to 0.19) |
| South Sudan | Leukemia | death | 0.002 (0.001 to 0.004) | 0.08 (0.02 to 0.17) | 0.005 (0.002 to 0.01) | 0.13 (0.05 to 0.25) | 1.66 (0.77 to 3.62) | 2.11 (1.97 to 2.24) |
| South Sudan | Acute myeloid leukemia | death | 0 (0 to 0.001) | 0.01 (0 to 0.03) | 0.001 (0 to 0.002) | 0.02 (0.01 to 0.04) | 2.33 (1.06 to 5.5) | 2.77 (2.6 to 2.94) |
| South Sudan | Acute lymphoid leukemia | death | 0 (0 to 0) | 0 (0 to 0.01) | 0 (0 to 0.001) | 0.01 (0 to 0.01) | 2.13 (0.9 to 4.89) | 2.4 (2.26 to 2.55) |
| South Sudan | Chronic myeloid leukemia | death | 0 (0 to 0.001) | 0.02 (0 to 0.04) | 0.001 (0 to 0.002) | 0.02 (0.01 to 0.05) | 1.45 (0.5 to 3.71) | 1.67 (1.46 to 1.87) |
| South Sudan | Chronic lymphoid leukemia | death | 0 (0 to 0.001) | 0.02 (0.01 to 0.05) | 0.001 (0 to 0.003) | 0.04 (0.01 to 0.08) | 2.21 (1.01 to 4.87) | 3.02 (2.81 to 3.23) |
| South Sudan | Other leukemia | death | 0.001 (0 to 0.002) | 0.03 (0.01 to 0.06) | 0.002 (0.001 to 0.004) | 0.04 (0.02 to 0.08) | 1.2 (0.41 to 2.86) | 1.33 (1.28 to 1.39) |
| South Sudan | Leukemia | DALYs | 0.07 (0.022 to 0.15) | 2.26 (0.71 to 4.84) | 0.185 (0.075 to 0.367) | 3.67 (1.51 to 7.09) | 1.65 (0.7 to 3.76) | 1.94 (1.79 to 2.08) |
| South Sudan | Acute myeloid leukemia | DALYs | 0.011 (0.003 to 0.032) | 0.32 (0.08 to 0.95) | 0.035 (0.012 to 0.085) | 0.63 (0.22 to 1.52) | 2.35 (1.01 to 5.75) | 2.7 (2.52 to 2.88) |
| South Sudan | Acute lymphoid leukemia | DALYs | 0.004 (0.001 to 0.01) | 0.12 (0.03 to 0.28) | 0.014 (0.005 to 0.031) | 0.23 (0.09 to 0.48) | 2.18 (0.87 to 5.2) | 2.48 (2.31 to 2.64) |
| South Sudan | Chronic myeloid leukemia | DALYs | 0.017 (0.005 to 0.043) | 0.51 (0.14 to 1.29) | 0.041 (0.013 to 0.09) | 0.74 (0.24 to 1.6) | 1.43 (0.44 to 3.83) | 1.5 (1.29 to 1.72) |
| South Sudan | Chronic lymphoid leukemia | DALYs | 0.009 (0.003 to 0.024) | 0.41 (0.12 to 1.06) | 0.032 (0.011 to 0.065) | 0.86 (0.3 to 1.76) | 2.43 (1.1 to 5.23) | 3.15 (2.93 to 3.37) |
| South Sudan | Other leukemia | DALYs | 0.028 (0.009 to 0.059) | 0.9 (0.29 to 1.87) | 0.062 (0.023 to 0.127) | 1.22 (0.47 to 2.46) | 1.19 (0.36 to 2.9) | 1.13 (1.07 to 1.19) |
| Spain | Leukemia | death | 0.191 (0.087 to 0.332) | 0.36 (0.17 to 0.63) | 0.382 (0.183 to 0.642) | 0.38 (0.18 to 0.63) | 1 (0.74 to 1.36) | -0.01 (-0.09 to 0.07) |
| Spain | Acute myeloid leukemia | death | 0.037 (0.016 to 0.07) | 0.07 (0.03 to 0.13) | 0.136 (0.064 to 0.229) | 0.15 (0.07 to 0.24) | 2.69 (1.32 to 3.77) | 2.57 (2.26 to 2.87) |
| Spain | Acute lymphoid leukemia | death | 0.012 (0.005 to 0.021) | 0.02 (0.01 to 0.04) | 0.02 (0.009 to 0.034) | 0.02 (0.01 to 0.04) | 0.67 (0.34 to 1.29) | -0.53 (-0.78 to -0.29) |
| Spain | Chronic myeloid leukemia | death | 0.024 (0.011 to 0.042) | 0.05 (0.02 to 0.08) | 0.017 (0.008 to 0.03) | 0.02 (0.01 to 0.03) | -0.3 (-0.42 to -0.1) | -5.15 (-5.72 to -4.58) |
| Spain | Chronic lymphoid leukemia | death | 0.029 (0.013 to 0.05) | 0.05 (0.02 to 0.09) | 0.079 (0.038 to 0.139) | 0.07 (0.03 to 0.12) | 1.74 (1.24 to 2.5) | 0.41 (0.07 to 0.74) |
| Spain | Other leukemia | death | 0.09 (0.041 to 0.157) | 0.17 (0.08 to 0.3) | 0.13 (0.061 to 0.225) | 0.12 (0.06 to 0.21) | 0.45 (0.22 to 0.8) | -0.97 (-1.16 to -0.78) |
| Spain | Leukemia | DALYs | 4.77 (2.173 to 8.283) | 9.67 (4.4 to 16.85) | 7.351 (3.573 to 12.152) | 8.94 (4.4 to 14.76) | 0.54 (0.35 to 0.82) | -0.53 (-0.63 to -0.43) |
| Spain | Acute myeloid leukemia | DALYs | 0.98 (0.426 to 1.772) | 2.02 (0.89 to 3.62) | 2.808 (1.349 to 4.636) | 3.58 (1.75 to 5.92) | 1.87 (0.91 to 2.67) | 1.9 (1.6 to 2.21) |
| Spain | Acute lymphoid leukemia | DALYs | 0.408 (0.179 to 0.739) | 0.9 (0.39 to 1.65) | 0.695 (0.338 to 1.176) | 1.1 (0.54 to 1.88) | 0.71 (0.32 to 1.49) | 0.01 (-0.31 to 0.34) |
| Spain | Chronic myeloid leukemia | DALYs | 0.635 (0.286 to 1.103) | 1.32 (0.6 to 2.3) | 0.375 (0.181 to 0.64) | 0.45 (0.22 to 0.76) | -0.41 (-0.52 to -0.26) | -5.31 (-5.88 to -4.74) |
| Spain | Chronic lymphoid leukemia | DALYs | 0.584 (0.274 to 1.01) | 1.06 (0.5 to 1.82) | 1.27 (0.617 to 2.194) | 1.28 (0.63 to 2.19) | 1.18 (0.79 to 1.79) | 0.08 (-0.31 to 0.47) |
| Spain | Other leukemia | DALYs | 2.165 (0.989 to 3.773) | 4.37 (2 to 7.67) | 2.203 (1.04 to 3.749) | 2.54 (1.21 to 4.29) | 0.02 (-0.13 to 0.27) | -1.85 (-2.04 to -1.66) |
| Sri Lanka | Leukemia | death | 0.014 (0.005 to 0.027) | 0.11 (0.04 to 0.22) | 0.043 (0.019 to 0.078) | 0.18 (0.08 to 0.32) | 2.13 (1.05 to 4.19) | 1.61 (1.5 to 1.72) |
| Sri Lanka | Acute myeloid leukemia | death | 0.003 (0.001 to 0.006) | 0.02 (0.01 to 0.05) | 0.013 (0.005 to 0.024) | 0.05 (0.02 to 0.09) | 3.12 (1.54 to 6.29) | 2.64 (2.55 to 2.73) |
| Sri Lanka | Acute lymphoid leukemia | death | 0.002 (0.001 to 0.003) | 0.01 (0 to 0.03) | 0.005 (0.002 to 0.009) | 0.02 (0.01 to 0.04) | 1.89 (0.84 to 3.99) | 1.61 (1.48 to 1.74) |
| Sri Lanka | Chronic myeloid leukemia | death | 0.001 (0 to 0.002) | 0.01 (0 to 0.01) | 0.002 (0.001 to 0.004) | 0.01 (0 to 0.02) | 1.33 (0.4 to 3.07) | 0.44 (0.2 to 0.68) |
| Sri Lanka | Chronic lymphoid leukemia | death | 0 (0 to 0) | 0 (0 to 0) | 0.001 (0 to 0.001) | 0 (0 to 0.01) | 3.21 (1.68 to 6.58) | 2.03 (1.77 to 2.29) |
| Sri Lanka | Other leukemia | death | 0.008 (0.003 to 0.016) | 0.07 (0.02 to 0.14) | 0.023 (0.01 to 0.042) | 0.1 (0.04 to 0.18) | 1.86 (0.83 to 3.92) | 1.27 (1.15 to 1.39) |
| Sri Lanka | Leukemia | DALYs | 0.509 (0.19 to 1.01) | 3.59 (1.34 to 7.07) | 1.269 (0.554 to 2.335) | 5.11 (2.22 to 9.47) | 1.49 (0.66 to 3.16) | 1.07 (0.92 to 1.22) |
| Sri Lanka | Acute myeloid leukemia | DALYs | 0.121 (0.043 to 0.25) | 0.83 (0.29 to 1.71) | 0.411 (0.172 to 0.766) | 1.63 (0.68 to 3.04) | 2.41 (1.11 to 5.2) | 2.27 (2.17 to 2.38) |
| Sri Lanka | Acute lymphoid leukemia | DALYs | 0.069 (0.024 to 0.148) | 0.44 (0.16 to 0.94) | 0.162 (0.068 to 0.308) | 0.68 (0.29 to 1.29) | 1.35 (0.45 to 3.21) | 1.19 (0.99 to 1.38) |
| Sri Lanka | Chronic myeloid leukemia | DALYs | 0.036 (0.013 to 0.074) | 0.25 (0.09 to 0.5) | 0.069 (0.03 to 0.131) | 0.28 (0.12 to 0.52) | 0.9 (0.13 to 2.42) | -0.09 (-0.39 to 0.21) |
| Sri Lanka | Chronic lymphoid leukemia | DALYs | 0.005 (0.002 to 0.01) | 0.04 (0.01 to 0.08) | 0.017 (0.007 to 0.031) | 0.07 (0.03 to 0.12) | 2.49 (1.22 to 5.13) | 1.53 (1.23 to 1.83) |
| Sri Lanka | Other leukemia | DALYs | 0.278 (0.102 to 0.566) | 2.03 (0.75 to 4.08) | 0.61 (0.255 to 1.154) | 2.45 (1.02 to 4.65) | 1.19 (0.4 to 2.8) | 0.54 (0.39 to 0.69) |
| Sudan | Leukemia | death | 0.023 (0.008 to 0.047) | 0.22 (0.07 to 0.44) | 0.099 (0.045 to 0.191) | 0.46 (0.21 to 0.87) | 3.41 (1.9 to 6.98) | 3.01 (2.8 to 3.21) |
| Sudan | Acute myeloid leukemia | death | 0.004 (0.001 to 0.009) | 0.04 (0.01 to 0.09) | 0.026 (0.01 to 0.051) | 0.11 (0.04 to 0.21) | 5.11 (2.78 to 10.43) | 3.91 (3.73 to 4.1) |
| Sudan | Acute lymphoid leukemia | death | 0.002 (0.001 to 0.005) | 0.02 (0.01 to 0.04) | 0.012 (0.004 to 0.025) | 0.05 (0.02 to 0.09) | 4.71 (2.34 to 9.85) | 3.68 (3.45 to 3.9) |
| Sudan | Chronic myeloid leukemia | death | 0.004 (0.001 to 0.009) | 0.03 (0.01 to 0.09) | 0.012 (0.004 to 0.027) | 0.05 (0.02 to 0.11) | 2.15 (0.86 to 4.92) | 1.6 (1.46 to 1.73) |
| Sudan | Chronic lymphoid leukemia | death | 0.001 (0 to 0.001) | 0.01 (0 to 0.01) | 0.004 (0.001 to 0.008) | 0.02 (0.01 to 0.04) | 5.25 (2.91 to 10.95) | 4.29 (4 to 4.58) |
| Sudan | Other leukemia | death | 0.012 (0.003 to 0.025) | 0.12 (0.04 to 0.24) | 0.046 (0.019 to 0.088) | 0.24 (0.1 to 0.44) | 2.9 (1.56 to 6.49) | 2.83 (2.61 to 3.05) |
| Sudan | Leukemia | DALYs | 0.79 (0.246 to 1.697) | 6.78 (2.24 to 14.2) | 3.558 (1.546 to 7.077) | 13.7 (6.04 to 26.55) | 3.51 (1.79 to 7.42) | 2.8 (2.61 to 2.98) |
| Sudan | Acute myeloid leukemia | DALYs | 0.154 (0.046 to 0.347) | 1.3 (0.39 to 2.89) | 1.006 (0.37 to 2.06) | 3.63 (1.38 to 7.23) | 5.54 (2.83 to 11.74) | 3.94 (3.76 to 4.11) |
| Sudan | Acute lymphoid leukemia | DALYs | 0.083 (0.023 to 0.208) | 0.65 (0.19 to 1.52) | 0.498 (0.182 to 1.095) | 1.64 (0.61 to 3.49) | 5.01 (2.37 to 11.19) | 3.64 (3.43 to 3.86) |
| Sudan | Chronic myeloid leukemia | DALYs | 0.143 (0.034 to 0.361) | 1.18 (0.28 to 2.86) | 0.449 (0.13 to 1.014) | 1.66 (0.5 to 3.69) | 2.14 (0.75 to 5.32) | 1.34 (1.23 to 1.44) |
| Sudan | Chronic lymphoid leukemia | DALYs | 0.018 (0.005 to 0.044) | 0.17 (0.05 to 0.41) | 0.115 (0.039 to 0.247) | 0.5 (0.17 to 1.06) | 5.22 (2.76 to 10.58) | 3.99 (3.74 to 4.23) |
| Sudan | Other leukemia | DALYs | 0.391 (0.1 to 0.851) | 3.48 (0.94 to 7.44) | 1.491 (0.604 to 2.891) | 6.27 (2.56 to 11.94) | 2.81 (1.36 to 6.75) | 2.45 (2.26 to 2.64) |
| Suriname | Leukemia | death | 0.001 (0 to 0.001) | 0.19 (0.08 to 0.35) | 0.002 (0.001 to 0.003) | 0.28 (0.13 to 0.49) | 2.23 (1.47 to 3.56) | 1.43 (1.22 to 1.64) |
| Suriname | Acute myeloid leukemia | death | 0 (0 to 0) | 0.03 (0.01 to 0.05) | 0 (0 to 0.001) | 0.05 (0.03 to 0.09) | 3.11 (1.97 to 5.12) | 2.6 (2.42 to 2.78) |
| Suriname | Acute lymphoid leukemia | death | 0 (0 to 0) | 0.01 (0 to 0.02) | 0 (0 to 0) | 0.01 (0.01 to 0.03) | 2.27 (1.35 to 3.99) | 1.84 (1.66 to 2.03) |
| Suriname | Chronic myeloid leukemia | death | 0 (0 to 0) | 0.02 (0.01 to 0.05) | 0 (0 to 0) | 0.03 (0.01 to 0.05) | 1.48 (0.81 to 2.58) | 0.23 (-0.04 to 0.49) |
| Suriname | Chronic lymphoid leukemia | death | 0 (0 to 0) | 0.01 (0 to 0.02) | 0 (0 to 0) | 0.02 (0.01 to 0.04) | 4.6 (2.99 to 7.49) | 3.4 (3.05 to 3.76) |
| Suriname | Other leukemia | death | 0 (0 to 0.001) | 0.12 (0.05 to 0.22) | 0.001 (0 to 0.002) | 0.16 (0.07 to 0.29) | 1.99 (1.28 to 3.18) | 1.1 (0.88 to 1.32) |
| Suriname | Leukemia | DALYs | 0.018 (0.007 to 0.033) | 5.83 (2.41 to 10.82) | 0.055 (0.027 to 0.095) | 8.93 (4.33 to 15.57) | 2.05 (1.29 to 3.42) | 1.41 (1.19 to 1.62) |
| Suriname | Acute myeloid leukemia | DALYs | 0.003 (0.001 to 0.006) | 0.95 (0.4 to 1.81) | 0.012 (0.006 to 0.021) | 1.94 (0.93 to 3.36) | 2.88 (1.79 to 4.95) | 2.65 (2.44 to 2.85) |
| Suriname | Acute lymphoid leukemia | DALYs | 0.001 (0 to 0.002) | 0.32 (0.13 to 0.63) | 0.003 (0.002 to 0.006) | 0.57 (0.26 to 1.05) | 2.07 (1.13 to 3.92) | 1.91 (1.71 to 2.12) |
| Suriname | Chronic myeloid leukemia | DALYs | 0.002 (0.001 to 0.004) | 0.73 (0.3 to 1.39) | 0.005 (0.002 to 0.009) | 0.85 (0.39 to 1.5) | 1.35 (0.68 to 2.44) | 0.17 (-0.11 to 0.45) |
| Suriname | Chronic lymphoid leukemia | DALYs | 0.001 (0 to 0.001) | 0.22 (0.09 to 0.42) | 0.003 (0.002 to 0.006) | 0.54 (0.25 to 0.95) | 4.52 (2.91 to 7.38) | 3.35 (3.02 to 3.68) |
| Suriname | Other leukemia | DALYs | 0.011 (0.004 to 0.021) | 3.6 (1.49 to 6.84) | 0.031 (0.015 to 0.055) | 5.03 (2.37 to 8.99) | 1.82 (1.09 to 3.12) | 1.04 (0.83 to 1.25) |
| Sweden | Leukemia | death | 0.044 (0.018 to 0.079) | 0.3 (0.12 to 0.53) | 0.075 (0.035 to 0.129) | 0.34 (0.16 to 0.58) | 0.7 (0.49 to 1.04) | 0.4 (0.32 to 0.49) |
| Sweden | Acute myeloid leukemia | death | 0.016 (0.007 to 0.03) | 0.11 (0.05 to 0.2) | 0.034 (0.016 to 0.059) | 0.16 (0.08 to 0.28) | 1.07 (0.72 to 1.5) | 1.24 (1.04 to 1.44) |
| Sweden | Acute lymphoid leukemia | death | 0.002 (0.001 to 0.004) | 0.02 (0.01 to 0.03) | 0.002 (0.001 to 0.004) | 0.01 (0.01 to 0.02) | -0.06 (-0.25 to 0.76) | -1.92 (-2.19 to -1.65) |
| Sweden | Chronic myeloid leukemia | death | 0.005 (0.002 to 0.01) | 0.04 (0.02 to 0.07) | 0.003 (0.001 to 0.004) | 0.01 (0.01 to 0.02) | -0.53 (-0.61 to -0.41) | -5.05 (-5.81 to -4.28) |
| Sweden | Chronic lymphoid leukemia | death | 0.012 (0.005 to 0.022) | 0.07 (0.03 to 0.14) | 0.023 (0.011 to 0.042) | 0.09 (0.04 to 0.16) | 0.94 (0.64 to 1.43) | 0.8 (0.57 to 1.04) |
| Sweden | Other leukemia | death | 0.008 (0.003 to 0.014) | 0.05 (0.02 to 0.09) | 0.013 (0.006 to 0.023) | 0.06 (0.03 to 0.1) | 0.63 (0.33 to 1.05) | 0.49 (0.41 to 0.58) |
| Sweden | Leukemia | DALYs | 0.965 (0.406 to 1.715) | 7.42 (3.17 to 13.27) | 1.39 (0.659 to 2.34) | 7.61 (3.63 to 12.78) | 0.44 (0.28 to 0.72) | 0 (-0.09 to 0.09) |
| Sweden | Acute myeloid leukemia | DALYs | 0.373 (0.157 to 0.667) | 2.94 (1.23 to 5.28) | 0.669 (0.308 to 1.142) | 3.87 (1.82 to 6.57) | 0.79 (0.5 to 1.19) | 0.98 (0.78 to 1.18) |
| Sweden | Acute lymphoid leukemia | DALYs | 0.08 (0.032 to 0.149) | 0.75 (0.29 to 1.42) | 0.078 (0.037 to 0.136) | 0.6 (0.28 to 1.02) | -0.03 (-0.25 to 0.79) | -1.51 (-1.8 to -1.22) |
| Sweden | Chronic myeloid leukemia | DALYs | 0.134 (0.057 to 0.238) | 1.11 (0.47 to 1.96) | 0.062 (0.029 to 0.104) | 0.37 (0.18 to 0.62) | -0.54 (-0.61 to -0.42) | -4.83 (-5.53 to -4.11) |
| Sweden | Chronic lymphoid leukemia | DALYs | 0.22 (0.094 to 0.393) | 1.45 (0.62 to 2.59) | 0.367 (0.171 to 0.631) | 1.65 (0.77 to 2.81) | 0.67 (0.41 to 1.06) | 0.45 (0.17 to 0.74) |
| Sweden | Other leukemia | DALYs | 0.158 (0.067 to 0.286) | 1.17 (0.49 to 2.13) | 0.214 (0.1 to 0.367) | 1.12 (0.52 to 1.9) | 0.36 (0.13 to 0.69) | 0.03 (-0.05 to 0.11) |
| Switzerland | Leukemia | death | 0.04 (0.018 to 0.07) | 0.38 (0.17 to 0.68) | 0.047 (0.022 to 0.081) | 0.26 (0.13 to 0.45) | 0.19 (0.04 to 0.43) | -0.97 (-1.38 to -0.55) |
| Switzerland | Acute myeloid leukemia | death | 0.013 (0.006 to 0.023) | 0.13 (0.06 to 0.23) | 0.021 (0.009 to 0.036) | 0.12 (0.05 to 0.21) | 0.59 (0.22 to 0.96) | 0.21 (-0.14 to 0.55) |
| Switzerland | Acute lymphoid leukemia | death | 0.002 (0.001 to 0.004) | 0.03 (0.01 to 0.05) | 0.002 (0.001 to 0.004) | 0.01 (0.01 to 0.03) | -0.07 (-0.29 to 0.3) | -1.97 (-2.38 to -1.55) |
| Switzerland | Chronic myeloid leukemia | death | 0.006 (0.003 to 0.011) | 0.06 (0.03 to 0.11) | 0.003 (0.001 to 0.005) | 0.02 (0.01 to 0.03) | -0.54 (-0.63 to -0.39) | -5 (-5.43 to -4.56) |
| Switzerland | Chronic lymphoid leukemia | death | 0.011 (0.005 to 0.02) | 0.1 (0.05 to 0.18) | 0.011 (0.005 to 0.02) | 0.06 (0.03 to 0.1) | 0 (-0.21 to 0.37) | -2 (-2.31 to -1.69) |
| Switzerland | Other leukemia | death | 0.007 (0.003 to 0.012) | 0.06 (0.03 to 0.11) | 0.01 (0.005 to 0.018) | 0.05 (0.03 to 0.09) | 0.55 (0.21 to 1.01) | 0.45 (-0.38 to 1.28) |
| Switzerland | Leukemia | DALYs | 0.939 (0.429 to 1.649) | 9.99 (4.6 to 17.67) | 0.932 (0.447 to 1.588) | 6.11 (2.93 to 10.36) | -0.01 (-0.12 to 0.18) | -1.5 (-1.92 to -1.08) |
| Switzerland | Acute myeloid leukemia | DALYs | 0.338 (0.153 to 0.608) | 3.72 (1.7 to 6.74) | 0.435 (0.193 to 0.749) | 2.98 (1.32 to 5.1) | 0.29 (0.02 to 0.59) | -0.46 (-0.84 to -0.08) |
| Switzerland | Acute lymphoid leukemia | DALYs | 0.092 (0.04 to 0.169) | 1.1 (0.48 to 2.04) | 0.078 (0.035 to 0.138) | 0.65 (0.29 to 1.15) | -0.16 (-0.38 to 0.26) | -2.02 (-2.37 to -1.67) |
| Switzerland | Chronic myeloid leukemia | DALYs | 0.165 (0.076 to 0.294) | 1.8 (0.83 to 3.22) | 0.069 (0.032 to 0.122) | 0.45 (0.21 to 0.8) | -0.58 (-0.67 to -0.44) | -5.15 (-5.58 to -4.72) |
| Switzerland | Chronic lymphoid leukemia | DALYs | 0.205 (0.091 to 0.362) | 1.94 (0.87 to 3.42) | 0.181 (0.083 to 0.321) | 1 (0.46 to 1.76) | -0.11 (-0.29 to 0.26) | -2.24 (-2.55 to -1.92) |
| Switzerland | Other leukemia | DALYs | 0.139 (0.062 to 0.245) | 1.44 (0.65 to 2.53) | 0.169 (0.081 to 0.296) | 1.03 (0.49 to 1.82) | 0.22 (-0.02 to 0.56) | -0.37 (-1.18 to 0.45) |
| Syrian Arab Republic | Leukemia | death | 0.086 (0.036 to 0.154) | 1.53 (0.65 to 2.75) | 0.18 (0.089 to 0.308) | 1.51 (0.75 to 2.61) | 1.09 (0.47 to 2.42) | -0.5 (-0.74 to -0.25) |
| Syrian Arab Republic | Acute myeloid leukemia | death | 0.011 (0.004 to 0.022) | 0.18 (0.07 to 0.35) | 0.029 (0.013 to 0.053) | 0.21 (0.09 to 0.4) | 1.66 (0.63 to 3.52) | 0.28 (0.02 to 0.55) |
| Syrian Arab Republic | Acute lymphoid leukemia | death | 0.004 (0.002 to 0.01) | 0.07 (0.03 to 0.14) | 0.01 (0.005 to 0.019) | 0.08 (0.04 to 0.14) | 1.31 (0.28 to 2.99) | -0.05 (-0.29 to 0.2) |
| Syrian Arab Republic | Chronic myeloid leukemia | death | 0.011 (0.004 to 0.021) | 0.19 (0.07 to 0.35) | 0.015 (0.006 to 0.028) | 0.12 (0.05 to 0.22) | 0.34 (-0.16 to 1.2) | -2.39 (-2.74 to -2.05) |
| Syrian Arab Republic | Chronic lymphoid leukemia | death | 0.002 (0.001 to 0.004) | 0.04 (0.01 to 0.07) | 0.006 (0.003 to 0.011) | 0.05 (0.02 to 0.09) | 2.27 (1.11 to 4.51) | 0.75 (0.38 to 1.12) |
| Syrian Arab Republic | Other leukemia | death | 0.058 (0.024 to 0.105) | 1.05 (0.44 to 1.92) | 0.12 (0.057 to 0.204) | 1.05 (0.51 to 1.8) | 1.07 (0.4 to 2.57) | -0.45 (-0.68 to -0.22) |
| Syrian Arab Republic | Leukemia | DALYs | 2.879 (1.17 to 5.291) | 43.68 (18.24 to 78.57) | 5.279 (2.585 to 9.09) | 39.33 (19.3 to 68.2) | 0.83 (0.24 to 2.04) | -0.84 (-1.08 to -0.61) |
| Syrian Arab Republic | Acute myeloid leukemia | DALYs | 0.383 (0.147 to 0.783) | 5.67 (2.24 to 11.37) | 0.942 (0.416 to 1.78) | 6.62 (2.97 to 12.52) | 1.46 (0.48 to 3.32) | 0.18 (-0.08 to 0.44) |
| Syrian Arab Republic | Acute lymphoid leukemia | DALYs | 0.172 (0.062 to 0.398) | 2.32 (0.89 to 5.11) | 0.355 (0.165 to 0.686) | 2.5 (1.18 to 4.85) | 1.06 (0.08 to 2.76) | -0.16 (-0.39 to 0.07) |
| Syrian Arab Republic | Chronic myeloid leukemia | DALYs | 0.391 (0.152 to 0.744) | 5.88 (2.3 to 11.1) | 0.459 (0.194 to 0.865) | 3.3 (1.42 to 6.2) | 0.17 (-0.3 to 0.98) | -2.79 (-3.15 to -2.43) |
| Syrian Arab Republic | Chronic lymphoid leukemia | DALYs | 0.056 (0.022 to 0.109) | 0.95 (0.37 to 1.83) | 0.174 (0.078 to 0.311) | 1.3 (0.59 to 2.31) | 2.08 (0.97 to 4.16) | 0.52 (0.17 to 0.87) |
| Syrian Arab Republic | Other leukemia | DALYs | 1.876 (0.764 to 3.447) | 28.86 (11.97 to 52.79) | 3.35 (1.609 to 5.715) | 25.59 (12.31 to 43.7) | 0.79 (0.18 to 2.2) | -0.88 (-1.1 to -0.66) |
| Taiwan (Province of China) | Leukemia | death | 0.021 (0.008 to 0.041) | 0.13 (0.05 to 0.25) | 0.069 (0.028 to 0.131) | 0.18 (0.08 to 0.35) | 2.3 (1.53 to 3.69) | 1.41 (1.32 to 1.5) |
| Taiwan (Province of China) | Acute myeloid leukemia | death | 0.003 (0.001 to 0.006) | 0.02 (0.01 to 0.03) | 0.028 (0.01 to 0.057) | 0.07 (0.03 to 0.15) | 8.26 (2.56 to 14.34) | 7.63 (6.76 to 8.51) |
| Taiwan (Province of China) | Acute lymphoid leukemia | death | 0.001 (0 to 0.003) | 0.01 (0 to 0.01) | 0.005 (0.002 to 0.01) | 0.01 (0.01 to 0.03) | 2.74 (1.29 to 5.33) | 3.46 (3.19 to 3.73) |
| Taiwan (Province of China) | Chronic myeloid leukemia | death | 0.002 (0.001 to 0.004) | 0.01 (0 to 0.02) | 0.004 (0.002 to 0.009) | 0.01 (0 to 0.02) | 1.1 (0.46 to 2.53) | 0.48 (0.03 to 0.92) |
| Taiwan (Province of China) | Chronic lymphoid leukemia | death | 0.001 (0 to 0.002) | 0.01 (0 to 0.02) | 0.004 (0.001 to 0.009) | 0.01 (0 to 0.02) | 2.37 (1.06 to 5.88) | 0.15 (-0.5 to 0.8) |
| Taiwan (Province of China) | Other leukemia | death | 0.013 (0.005 to 0.027) | 0.08 (0.03 to 0.16) | 0.028 (0.011 to 0.061) | 0.07 (0.03 to 0.16) | 1.09 (0.51 to 2.48) | -1.33 (-1.78 to -0.87) |
| Taiwan (Province of China) | Leukemia | DALYs | 0.719 (0.257 to 1.427) | 3.8 (1.39 to 7.48) | 1.851 (0.771 to 3.487) | 5.29 (2.23 to 9.95) | 1.58 (0.93 to 2.92) | 1.2 (1.12 to 1.28) |
| Taiwan (Province of China) | Acute myeloid leukemia | DALYs | 0.111 (0.038 to 0.228) | 0.57 (0.2 to 1.18) | 0.772 (0.284 to 1.534) | 2.2 (0.82 to 4.38) | 5.96 (1.98 to 11.17) | 6.75 (6.03 to 7.47) |
| Taiwan (Province of China) | Acute lymphoid leukemia | DALYs | 0.055 (0.019 to 0.112) | 0.26 (0.09 to 0.53) | 0.2 (0.084 to 0.382) | 0.64 (0.27 to 1.22) | 2.67 (1.2 to 5.47) | 4.1 (3.78 to 4.42) |
| Taiwan (Province of China) | Chronic myeloid leukemia | DALYs | 0.066 (0.024 to 0.131) | 0.36 (0.13 to 0.71) | 0.107 (0.042 to 0.223) | 0.3 (0.12 to 0.63) | 0.62 (0.1 to 1.81) | -0.1 (-0.47 to 0.27) |
| Taiwan (Province of China) | Chronic lymphoid leukemia | DALYs | 0.03 (0.01 to 0.061) | 0.18 (0.06 to 0.37) | 0.085 (0.033 to 0.183) | 0.22 (0.08 to 0.47) | 1.87 (0.75 to 5.04) | 0.06 (-0.61 to 0.73) |
| Taiwan (Province of China) | Other leukemia | DALYs | 0.457 (0.164 to 0.91) | 2.43 (0.89 to 4.81) | 0.687 (0.265 to 1.471) | 1.93 (0.74 to 4.13) | 0.5 (0.04 to 1.63) | -1.81 (-2.25 to -1.36) |
| Tajikistan | Leukemia | death | 0.004 (0.001 to 0.009) | 0.13 (0.04 to 0.26) | 0.008 (0.003 to 0.016) | 0.15 (0.06 to 0.28) | 1.02 (0.49 to 2.13) | 0.46 (-0.07 to 0.99) |
| Tajikistan | Acute myeloid leukemia | death | 0.001 (0 to 0.002) | 0.03 (0.01 to 0.06) | 0.002 (0.001 to 0.005) | 0.04 (0.01 to 0.07) | 1.68 (0.85 to 3.18) | 1.24 (0.58 to 1.9) |
| Tajikistan | Acute lymphoid leukemia | death | 0.001 (0 to 0.001) | 0.01 (0 to 0.03) | 0.001 (0 to 0.003) | 0.02 (0.01 to 0.04) | 1.43 (0.73 to 2.88) | 0.74 (0.29 to 1.2) |
| Tajikistan | Chronic myeloid leukemia | death | 0 (0 to 0.001) | 0.01 (0 to 0.03) | 0.001 (0 to 0.001) | 0.01 (0 to 0.02) | 0.3 (-0.15 to 1.16) | -1.23 (-1.94 to -0.51) |
| Tajikistan | Chronic lymphoid leukemia | death | 0 (0 to 0) | 0.01 (0 to 0.02) | 0.001 (0 to 0.001) | 0.01 (0.01 to 0.03) | 1.69 (0.78 to 3.48) | 1.94 (1.35 to 2.53) |
| Tajikistan | Other leukemia | death | 0.002 (0.001 to 0.005) | 0.06 (0.02 to 0.14) | 0.004 (0.001 to 0.007) | 0.07 (0.03 to 0.13) | 0.68 (0.16 to 1.87) | 0.07 (-0.39 to 0.53) |
| Tajikistan | Leukemia | DALYs | 0.165 (0.053 to 0.365) | 4.63 (1.56 to 9.88) | 0.311 (0.117 to 0.642) | 4.35 (1.71 to 8.59) | 0.88 (0.35 to 2.11) | -0.38 (-0.92 to 0.16) |
| Tajikistan | Acute myeloid leukemia | DALYs | 0.038 (0.011 to 0.085) | 1.05 (0.32 to 2.32) | 0.098 (0.036 to 0.204) | 1.26 (0.48 to 2.57) | 1.59 (0.75 to 3.19) | 0.81 (0.18 to 1.45) |
| Tajikistan | Acute lymphoid leukemia | DALYs | 0.023 (0.007 to 0.056) | 0.61 (0.19 to 1.4) | 0.053 (0.019 to 0.117) | 0.66 (0.24 to 1.42) | 1.3 (0.61 to 2.86) | 0.2 (-0.24 to 0.63) |
| Tajikistan | Chronic myeloid leukemia | DALYs | 0.015 (0.004 to 0.033) | 0.44 (0.13 to 0.97) | 0.018 (0.006 to 0.039) | 0.26 (0.09 to 0.54) | 0.19 (-0.23 to 1.04) | -2.16 (-2.89 to -1.43) |
| Tajikistan | Chronic lymphoid leukemia | DALYs | 0.008 (0.002 to 0.017) | 0.24 (0.08 to 0.52) | 0.018 (0.007 to 0.038) | 0.33 (0.12 to 0.69) | 1.37 (0.53 to 3.07) | 0.91 (0.29 to 1.53) |
| Tajikistan | Other leukemia | DALYs | 0.082 (0.024 to 0.183) | 2.29 (0.72 to 4.98) | 0.124 (0.046 to 0.257) | 1.85 (0.72 to 3.69) | 0.52 (0 to 1.85) | -1.06 (-1.55 to -0.57) |
| Thailand | Leukemia | death | 0.047 (0.014 to 0.101) | 0.11 (0.03 to 0.25) | 0.24 (0.098 to 0.468) | 0.24 (0.1 to 0.48) | 4.15 (2.16 to 8.98) | 2.52 (2.18 to 2.87) |
| Thailand | Acute myeloid leukemia | death | 0.003 (0.001 to 0.007) | 0.01 (0 to 0.02) | 0.037 (0.014 to 0.075) | 0.04 (0.01 to 0.08) | 12.19 (4.44 to 29.84) | 6.87 (6.44 to 7.3) |
| Thailand | Acute lymphoid leukemia | death | 0.001 (0 to 0.003) | 0 (0 to 0.01) | 0.006 (0.003 to 0.012) | 0.01 (0 to 0.01) | 5.1 (2.06 to 12.59) | 3.85 (3.53 to 4.18) |
| Thailand | Chronic myeloid leukemia | death | 0.003 (0.001 to 0.006) | 0.01 (0 to 0.01) | 0.019 (0.008 to 0.035) | 0.02 (0.01 to 0.04) | 6.11 (3.19 to 13.63) | 4.25 (3.91 to 4.6) |
| Thailand | Chronic lymphoid leukemia | death | 0.001 (0 to 0.003) | 0 (0 to 0.01) | 0.013 (0.005 to 0.025) | 0.01 (0 to 0.03) | 10.04 (5.55 to 22.92) | 4.2 (3.87 to 4.54) |
| Thailand | Other leukemia | death | 0.039 (0.011 to 0.086) | 0.1 (0.03 to 0.22) | 0.165 (0.065 to 0.327) | 0.17 (0.07 to 0.33) | 3.24 (1.59 to 7.25) | 1.72 (1.32 to 2.12) |
| Thailand | Leukemia | DALYs | 1.744 (0.527 to 3.78) | 3.65 (1.1 to 7.86) | 7.13 (3.018 to 13.726) | 7.54 (3.17 to 14.62) | 3.09 (1.49 to 7.13) | 2.17 (1.74 to 2.6) |
| Thailand | Acute myeloid leukemia | DALYs | 0.108 (0.031 to 0.253) | 0.22 (0.06 to 0.52) | 1.15 (0.454 to 2.286) | 1.22 (0.48 to 2.37) | 9.63 (3.57 to 24.67) | 6.51 (6.11 to 6.91) |
| Thailand | Acute lymphoid leukemia | DALYs | 0.045 (0.012 to 0.113) | 0.08 (0.02 to 0.21) | 0.229 (0.097 to 0.44) | 0.26 (0.11 to 0.51) | 4.12 (1.45 to 10.58) | 3.71 (3.32 to 4.11) |
| Thailand | Chronic myeloid leukemia | DALYs | 0.098 (0.029 to 0.223) | 0.2 (0.06 to 0.45) | 0.592 (0.255 to 1.123) | 0.63 (0.27 to 1.21) | 5.01 (2.51 to 11.41) | 4.07 (3.71 to 4.44) |
| Thailand | Chronic lymphoid leukemia | DALYs | 0.029 (0.008 to 0.065) | 0.08 (0.02 to 0.18) | 0.26 (0.105 to 0.502) | 0.26 (0.1 to 0.49) | 8.11 (4.41 to 18.69) | 3.92 (3.6 to 4.25) |
| Thailand | Other leukemia | DALYs | 1.464 (0.437 to 3.186) | 3.07 (0.91 to 6.69) | 4.899 (2.008 to 9.483) | 5.17 (2.14 to 10.04) | 2.35 (1.02 to 5.47) | 1.33 (0.83 to 1.83) |
| Timor-Leste | Leukemia | death | 0 (0 to 0.001) | 0.06 (0.01 to 0.16) | 0.001 (0 to 0.002) | 0.08 (0.02 to 0.2) | 2.31 (1.13 to 5.73) | 1.61 (1.03 to 2.2) |
| Timor-Leste | Acute myeloid leukemia | death | 0 (0 to 0) | 0.01 (0 to 0.02) | 0 (0 to 0) | 0.01 (0 to 0.04) | 3.89 (1.83 to 10.09) | 3.39 (2.75 to 4.03) |
| Timor-Leste | Acute lymphoid leukemia | death | 0 (0 to 0) | 0 (0 to 0.01) | 0 (0 to 0) | 0.01 (0 to 0.02) | 3.04 (0.84 to 9.57) | 2.95 (2.35 to 3.55) |
| Timor-Leste | Chronic myeloid leukemia | death | 0 (0 to 0) | 0 (0 to 0.01) | 0 (0 to 0) | 0 (0 to 0.01) | 1.68 (0.54 to 4.62) | 0.91 (0.23 to 1.59) |
| Timor-Leste | Chronic lymphoid leukemia | death | 0 (0 to 0) | 0 (0 to 0) | 0 (0 to 0) | 0 (0 to 0.01) | 5.75 (3.06 to 13.65) | 3.74 (3.12 to 4.36) |
| Timor-Leste | Other leukemia | death | 0 (0 to 0) | 0.04 (0.01 to 0.12) | 0 (0 to 0.001) | 0.06 (0.02 to 0.13) | 1.99 (0.92 to 5.07) | 1.15 (0.58 to 1.71) |
| Timor-Leste | Leukemia | DALYs | 0.009 (0.002 to 0.025) | 1.85 (0.34 to 4.96) | 0.025 (0.007 to 0.057) | 2.57 (0.74 to 5.99) | 1.79 (0.58 to 5.12) | 1.53 (0.88 to 2.18) |
| Timor-Leste | Acute myeloid leukemia | DALYs | 0.001 (0 to 0.003) | 0.21 (0.03 to 0.66) | 0.004 (0.001 to 0.011) | 0.45 (0.12 to 1.17) | 3.34 (1.25 to 9.66) | 3.47 (2.78 to 4.17) |
| Timor-Leste | Acute lymphoid leukemia | DALYs | 0.001 (0 to 0.002) | 0.11 (0.02 to 0.4) | 0.002 (0.001 to 0.005) | 0.21 (0.06 to 0.54) | 2.47 (0.38 to 8.56) | 2.8 (2.15 to 3.46) |
| Timor-Leste | Chronic myeloid leukemia | DALYs | 0.001 (0 to 0.002) | 0.13 (0.02 to 0.38) | 0.001 (0 to 0.003) | 0.14 (0.04 to 0.36) | 1.22 (0.14 to 4.01) | 0.69 (-0.03 to 1.42) |
| Timor-Leste | Chronic lymphoid leukemia | DALYs | 0 (0 to 0) | 0.03 (0.01 to 0.09) | 0.001 (0 to 0.002) | 0.07 (0.02 to 0.18) | 4.6 (2.29 to 11.45) | 3.56 (2.9 to 4.23) |
| Timor-Leste | Other leukemia | DALYs | 0.006 (0.001 to 0.018) | 1.38 (0.24 to 3.81) | 0.016 (0.004 to 0.039) | 1.69 (0.47 to 4.03) | 1.49 (0.42 to 4.66) | 1.02 (0.39 to 1.65) |
| Togo | Leukemia | death | 0.001 (0 to 0.002) | 0.08 (0.03 to 0.17) | 0.006 (0.003 to 0.012) | 0.16 (0.07 to 0.29) | 4.63 (2.72 to 8.93) | 2.37 (2.3 to 2.44) |
| Togo | Acute myeloid leukemia | death | 0 (0 to 0) | 0.01 (0 to 0.02) | 0.001 (0 to 0.002) | 0.03 (0.01 to 0.05) | 5.73 (3.12 to 11.88) | 3.07 (3 to 3.13) |
| Togo | Acute lymphoid leukemia | death | 0 (0 to 0) | 0 (0 to 0.01) | 0 (0 to 0.001) | 0.01 (0 to 0.01) | 4.87 (2.28 to 10.67) | 2.39 (2.27 to 2.5) |
| Togo | Chronic myeloid leukemia | death | 0 (0 to 0.001) | 0.02 (0.01 to 0.04) | 0.001 (0 to 0.002) | 0.02 (0.01 to 0.05) | 3.5 (1.74 to 7.48) | 1.48 (1.42 to 1.54) |
| Togo | Chronic lymphoid leukemia | death | 0 (0 to 0) | 0.02 (0.01 to 0.04) | 0.001 (0 to 0.002) | 0.04 (0.01 to 0.07) | 4.84 (2.71 to 10) | 2.56 (2.46 to 2.66) |
| Togo | Other leukemia | death | 0 (0 to 0.001) | 0.03 (0.01 to 0.07) | 0.002 (0.001 to 0.005) | 0.06 (0.02 to 0.12) | 4.77 (2.79 to 9.33) | 2.39 (2.25 to 2.54) |
| Togo | Leukemia | DALYs | 0.04 (0.013 to 0.085) | 2.36 (0.8 to 4.87) | 0.224 (0.093 to 0.416) | 4.53 (1.9 to 8.43) | 4.55 (2.6 to 8.96) | 2.28 (2.22 to 2.33) |
| Togo | Acute myeloid leukemia | DALYs | 0.007 (0.002 to 0.016) | 0.36 (0.11 to 0.8) | 0.044 (0.017 to 0.084) | 0.84 (0.32 to 1.61) | 5.39 (2.85 to 11.61) | 2.87 (2.8 to 2.94) |
| Togo | Acute lymphoid leukemia | DALYs | 0.003 (0.001 to 0.008) | 0.14 (0.04 to 0.33) | 0.02 (0.007 to 0.041) | 0.3 (0.1 to 0.61) | 4.91 (2.21 to 11.32) | 2.58 (2.47 to 2.69) |
| Togo | Chronic myeloid leukemia | DALYs | 0.011 (0.003 to 0.025) | 0.58 (0.18 to 1.25) | 0.051 (0.02 to 0.096) | 0.88 (0.35 to 1.66) | 3.5 (1.7 to 7.65) | 1.45 (1.37 to 1.54) |
| Togo | Chronic lymphoid leukemia | DALYs | 0.005 (0.002 to 0.011) | 0.41 (0.12 to 0.88) | 0.029 (0.012 to 0.057) | 0.82 (0.33 to 1.61) | 4.77 (2.56 to 9.84) | 2.44 (2.34 to 2.54) |
| Togo | Other leukemia | DALYs | 0.014 (0.004 to 0.03) | 0.86 (0.29 to 1.88) | 0.08 (0.031 to 0.154) | 1.7 (0.67 to 3.24) | 4.83 (2.76 to 9.59) | 2.37 (2.23 to 2.51) |
| Tokelau | Leukemia | death | 0 (0 to 0) | 0.32 (0.11 to 0.62) | 0 (0 to 0) | 0.35 (0.16 to 0.64) | 0.11 (-0.24 to 0.77) | 0.17 (0.08 to 0.26) |
| Tokelau | Acute myeloid leukemia | death | 0 (0 to 0) | 0.07 (0.02 to 0.15) | 0 (0 to 0) | 0.09 (0.04 to 0.17) | 0.33 (-0.16 to 1.38) | 0.79 (0.68 to 0.89) |
| Tokelau | Acute lymphoid leukemia | death | 0 (0 to 0) | 0.02 (0.01 to 0.04) | 0 (0 to 0) | 0.03 (0.01 to 0.05) | 0.36 (-0.16 to 1.38) | 0.76 (0.64 to 0.89) |
| Tokelau | Chronic myeloid leukemia | death | 0 (0 to 0) | 0.06 (0.02 to 0.12) | 0 (0 to 0) | 0.04 (0.02 to 0.09) | -0.23 (-0.53 to 0.33) | -1.03 (-1.18 to -0.88) |
| Tokelau | Chronic lymphoid leukemia | death | 0 (0 to 0) | 0.01 (0 to 0.01) | 0 (0 to 0) | 0.01 (0 to 0.01) | 0.34 (-0.18 to 1.33) | 0.59 (0.52 to 0.65) |
| Tokelau | Other leukemia | death | 0 (0 to 0) | 0.17 (0.06 to 0.35) | 0 (0 to 0) | 0.18 (0.08 to 0.35) | 0.1 (-0.26 to 0.79) | 0.15 (0.07 to 0.22) |
| Tokelau | Leukemia | DALYs | 0 (0 to 0) | 10.67 (3.81 to 20.62) | 0 (0 to 0) | 11.56 (5.26 to 21.33) | 0.11 (-0.27 to 0.84) | 0.11 (0 to 0.21) |
| Tokelau | Acute myeloid leukemia | DALYs | 0 (0 to 0) | 2.6 (0.89 to 5.51) | 0 (0 to 0) | 3.39 (1.51 to 6.4) | 0.34 (-0.18 to 1.41) | 0.81 (0.7 to 0.92) |
| Tokelau | Acute lymphoid leukemia | DALYs | 0 (0 to 0) | 0.8 (0.26 to 1.86) | 0 (0 to 0) | 1.06 (0.44 to 2.06) | 0.33 (-0.21 to 1.47) | 0.82 (0.68 to 0.96) |
| Tokelau | Chronic myeloid leukemia | DALYs | 0 (0 to 0) | 1.9 (0.64 to 4.07) | 0 (0 to 0) | 1.45 (0.58 to 2.8) | -0.23 (-0.54 to 0.36) | -1.16 (-1.33 to -1) |
| Tokelau | Chronic lymphoid leukemia | DALYs | 0 (0 to 0) | 0.17 (0.06 to 0.37) | 0 (0 to 0) | 0.22 (0.09 to 0.44) | 0.39 (-0.15 to 1.42) | 0.64 (0.58 to 0.71) |
| Tokelau | Other leukemia | DALYs | 0 (0 to 0) | 5.2 (1.81 to 10.52) | 0 (0 to 0) | 5.46 (2.25 to 10.27) | 0.08 (-0.3 to 0.83) | -0.02 (-0.12 to 0.07) |
| Tonga | Leukemia | death | 0 (0 to 0) | 0.22 (0.1 to 0.38) | 0 (0 to 0) | 0.26 (0.13 to 0.44) | 0.6 (0.2 to 1.23) | 0.16 (-0.16 to 0.49) |
| Tonga | Acute myeloid leukemia | death | 0 (0 to 0) | 0.06 (0.03 to 0.1) | 0 (0 to 0) | 0.07 (0.04 to 0.13) | 0.77 (0.22 to 1.64) | 0.54 (0.19 to 0.88) |
| Tonga | Acute lymphoid leukemia | death | 0 (0 to 0) | 0.01 (0.01 to 0.03) | 0 (0 to 0) | 0.02 (0.01 to 0.03) | 0.73 (0.18 to 1.61) | 0.58 (0.2 to 0.96) |
| Tonga | Chronic myeloid leukemia | death | 0 (0 to 0) | 0.03 (0.01 to 0.05) | 0 (0 to 0) | 0.03 (0.01 to 0.05) | 0.19 (-0.2 to 0.83) | -0.91 (-1.27 to -0.55) |
| Tonga | Chronic lymphoid leukemia | death | 0 (0 to 0) | 0 (0 to 0.01) | 0 (0 to 0) | 0 (0 to 0.01) | 0.55 (0.02 to 1.36) | -0.05 (-0.42 to 0.32) |
| Tonga | Other leukemia | death | 0 (0 to 0) | 0.12 (0.05 to 0.21) | 0 (0 to 0) | 0.14 (0.07 to 0.24) | 0.59 (0.17 to 1.3) | 0.15 (-0.15 to 0.46) |
| Tonga | Leukemia | DALYs | 0.005 (0.002 to 0.008) | 7.16 (3.51 to 11.9) | 0.007 (0.004 to 0.012) | 8.32 (4.33 to 13.84) | 0.52 (0.1 to 1.19) | 0.13 (-0.19 to 0.45) |
| Tonga | Acute myeloid leukemia | DALYs | 0.001 (0.001 to 0.002) | 2.04 (0.96 to 3.62) | 0.002 (0.001 to 0.004) | 2.72 (1.4 to 4.54) | 0.73 (0.18 to 1.64) | 0.57 (0.23 to 0.91) |
| Tonga | Acute lymphoid leukemia | DALYs | 0 (0 to 0.001) | 0.57 (0.27 to 1.02) | 0.001 (0 to 0.001) | 0.76 (0.37 to 1.32) | 0.7 (0.14 to 1.62) | 0.63 (0.26 to 1) |
| Tonga | Chronic myeloid leukemia | DALYs | 0.001 (0 to 0.001) | 0.93 (0.42 to 1.65) | 0.001 (0 to 0.001) | 0.81 (0.4 to 1.45) | 0.14 (-0.23 to 0.81) | -1.03 (-1.38 to -0.67) |
| Tonga | Chronic lymphoid leukemia | DALYs | 0 (0 to 0) | 0.11 (0.05 to 0.21) | 0 (0 to 0) | 0.13 (0.06 to 0.23) | 0.53 (0.01 to 1.34) | 0.03 (-0.31 to 0.38) |
| Tonga | Other leukemia | DALYs | 0.002 (0.001 to 0.004) | 3.52 (1.6 to 6.07) | 0.003 (0.002 to 0.006) | 3.91 (1.96 to 6.7) | 0.46 (0.03 to 1.15) | 0.04 (-0.26 to 0.34) |
| Trinidad and Tobago | Leukemia | death | 0.003 (0.001 to 0.005) | 0.33 (0.15 to 0.55) | 0.006 (0.003 to 0.01) | 0.32 (0.16 to 0.55) | 0.93 (0.42 to 1.59) | 0.07 (-0.05 to 0.19) |
| Trinidad and Tobago | Acute myeloid leukemia | death | 0.001 (0 to 0.001) | 0.08 (0.04 to 0.14) | 0.002 (0.001 to 0.004) | 0.12 (0.06 to 0.21) | 1.61 (0.89 to 2.68) | 1.66 (1.46 to 1.86) |
| Trinidad and Tobago | Acute lymphoid leukemia | death | 0 (0 to 0) | 0.03 (0.01 to 0.05) | 0 (0 to 0.001) | 0.03 (0.01 to 0.05) | 0.71 (0.22 to 1.43) | 0.33 (0.12 to 0.55) |
| Trinidad and Tobago | Chronic myeloid leukemia | death | 0 (0 to 0.001) | 0.05 (0.03 to 0.09) | 0.001 (0 to 0.001) | 0.03 (0.02 to 0.06) | 0.28 (-0.08 to 0.78) | -1.99 (-2.25 to -1.73) |
| Trinidad and Tobago | Chronic lymphoid leukemia | death | 0 (0 to 0) | 0.02 (0.01 to 0.03) | 0 (0 to 0.001) | 0.02 (0.01 to 0.04) | 1.9 (1.02 to 3.13) | 1.04 (0.61 to 1.47) |
| Trinidad and Tobago | Other leukemia | death | 0.001 (0.001 to 0.002) | 0.14 (0.07 to 0.24) | 0.002 (0.001 to 0.004) | 0.12 (0.06 to 0.2) | 0.7 (0.23 to 1.3) | -0.67 (-0.82 to -0.51) |
| Trinidad and Tobago | Leukemia | DALYs | 0.106 (0.05 to 0.176) | 10.52 (4.92 to 17.4) | 0.179 (0.088 to 0.305) | 10.53 (5.18 to 17.93) | 0.69 (0.21 to 1.35) | 0.07 (-0.03 to 0.17) |
| Trinidad and Tobago | Acute myeloid leukemia | DALYs | 0.031 (0.015 to 0.052) | 2.98 (1.41 to 5) | 0.071 (0.034 to 0.122) | 4.21 (2.08 to 7.24) | 1.28 (0.61 to 2.29) | 1.62 (1.43 to 1.8) |
| Trinidad and Tobago | Acute lymphoid leukemia | DALYs | 0.013 (0.006 to 0.023) | 1.17 (0.54 to 2.03) | 0.02 (0.009 to 0.035) | 1.26 (0.59 to 2.23) | 0.48 (0.03 to 1.16) | 0.31 (0.08 to 0.53) |
| Trinidad and Tobago | Chronic myeloid leukemia | DALYs | 0.016 (0.008 to 0.027) | 1.64 (0.77 to 2.77) | 0.019 (0.009 to 0.032) | 1.06 (0.51 to 1.8) | 0.16 (-0.19 to 0.64) | -2 (-2.24 to -1.76) |
| Trinidad and Tobago | Chronic lymphoid leukemia | DALYs | 0.003 (0.002 to 0.006) | 0.4 (0.19 to 0.68) | 0.01 (0.005 to 0.016) | 0.52 (0.25 to 0.88) | 1.79 (0.9 to 2.99) | 1.13 (0.76 to 1.51) |
| Trinidad and Tobago | Other leukemia | DALYs | 0.042 (0.02 to 0.071) | 4.33 (2.02 to 7.25) | 0.061 (0.03 to 0.103) | 3.49 (1.7 to 5.94) | 0.43 (0 to 1.01) | -0.81 (-0.96 to -0.66) |
| Tunisia | Leukemia | death | 0.011 (0.005 to 0.019) | 0.21 (0.09 to 0.38) | 0.034 (0.016 to 0.06) | 0.28 (0.13 to 0.49) | 2.16 (1.09 to 3.77) | 0.97 (0.9 to 1.05) |
| Tunisia | Acute myeloid leukemia | death | 0.003 (0.001 to 0.005) | 0.05 (0.02 to 0.09) | 0.01 (0.005 to 0.019) | 0.08 (0.04 to 0.15) | 2.62 (1.33 to 4.61) | 1.65 (1.59 to 1.7) |
| Tunisia | Acute lymphoid leukemia | death | 0.001 (0 to 0.002) | 0.02 (0.01 to 0.04) | 0.003 (0.002 to 0.006) | 0.03 (0.01 to 0.05) | 2.04 (1.01 to 3.83) | 1.24 (1.19 to 1.29) |
| Tunisia | Chronic myeloid leukemia | death | 0.002 (0.001 to 0.003) | 0.03 (0.01 to 0.05) | 0.004 (0.002 to 0.007) | 0.03 (0.01 to 0.06) | 1.48 (0.6 to 2.89) | 0.18 (0.1 to 0.25) |
| Tunisia | Chronic lymphoid leukemia | death | 0.001 (0 to 0.001) | 0.01 (0.01 to 0.03) | 0.003 (0.001 to 0.006) | 0.03 (0.01 to 0.05) | 3.41 (1.76 to 6.2) | 1.93 (1.81 to 2.05) |
| Tunisia | Other leukemia | death | 0.004 (0.002 to 0.008) | 0.1 (0.04 to 0.18) | 0.013 (0.006 to 0.024) | 0.11 (0.05 to 0.21) | 1.92 (0.86 to 4.01) | 0.55 (0.46 to 0.64) |
| Tunisia | Leukemia | DALYs | 0.341 (0.151 to 0.615) | 5.8 (2.55 to 10.41) | 0.969 (0.453 to 1.699) | 7.49 (3.53 to 13.1) | 1.84 (0.85 to 3.33) | 0.89 (0.83 to 0.94) |
| Tunisia | Acute myeloid leukemia | DALYs | 0.106 (0.047 to 0.192) | 1.71 (0.76 to 3.05) | 0.351 (0.164 to 0.63) | 2.65 (1.24 to 4.75) | 2.31 (1.09 to 4.2) | 1.57 (1.53 to 1.62) |
| Tunisia | Acute lymphoid leukemia | DALYs | 0.045 (0.019 to 0.086) | 0.68 (0.3 to 1.31) | 0.128 (0.061 to 0.229) | 0.99 (0.47 to 1.77) | 1.83 (0.84 to 3.64) | 1.29 (1.26 to 1.33) |
| Tunisia | Chronic myeloid leukemia | DALYs | 0.051 (0.022 to 0.094) | 0.86 (0.38 to 1.58) | 0.113 (0.052 to 0.205) | 0.86 (0.4 to 1.56) | 1.21 (0.42 to 2.51) | -0.04 (-0.11 to 0.02) |
| Tunisia | Chronic lymphoid leukemia | DALYs | 0.02 (0.008 to 0.037) | 0.35 (0.15 to 0.65) | 0.08 (0.037 to 0.143) | 0.62 (0.29 to 1.12) | 3.04 (1.53 to 5.54) | 1.85 (1.74 to 1.95) |
| Tunisia | Other leukemia | DALYs | 0.119 (0.049 to 0.221) | 2.2 (0.92 to 4.09) | 0.298 (0.131 to 0.565) | 2.37 (1.04 to 4.47) | 1.5 (0.56 to 3.38) | 0.27 (0.19 to 0.34) |
| Turkey | Leukemia | death | 0.227 (0.104 to 0.39) | 0.59 (0.27 to 1.02) | 0.485 (0.24 to 0.815) | 0.55 (0.27 to 0.94) | 1.13 (0.57 to 2.13) | -0.43 (-0.62 to -0.23) |
| Turkey | Acute myeloid leukemia | death | 0.077 (0.035 to 0.137) | 0.19 (0.09 to 0.34) | 0.211 (0.105 to 0.349) | 0.23 (0.12 to 0.39) | 1.73 (0.92 to 3.03) | 0.54 (0.4 to 0.69) |
| Turkey | Acute lymphoid leukemia | death | 0.025 (0.01 to 0.049) | 0.05 (0.02 to 0.11) | 0.047 (0.021 to 0.081) | 0.05 (0.02 to 0.09) | 0.93 (0.26 to 2.11) | -0.51 (-0.77 to -0.24) |
| Turkey | Chronic myeloid leukemia | death | 0.035 (0.015 to 0.063) | 0.09 (0.04 to 0.17) | 0.035 (0.017 to 0.064) | 0.04 (0.02 to 0.08) | 0.02 (-0.31 to 0.58) | -3.4 (-3.62 to -3.17) |
| Turkey | Chronic lymphoid leukemia | death | 0.022 (0.009 to 0.039) | 0.06 (0.03 to 0.11) | 0.076 (0.036 to 0.139) | 0.09 (0.04 to 0.16) | 2.52 (1.36 to 4.28) | 1.37 (1.2 to 1.54) |
| Turkey | Other leukemia | death | 0.069 (0.028 to 0.125) | 0.19 (0.08 to 0.35) | 0.116 (0.054 to 0.219) | 0.14 (0.06 to 0.26) | 0.67 (0.13 to 2.25) | -1.31 (-1.54 to -1.07) |
| Turkey | Leukemia | DALYs | 7.559 (3.335 to 13.216) | 17.44 (7.93 to 30.06) | 13.433 (6.626 to 22.395) | 14.67 (7.25 to 24.48) | 0.78 (0.26 to 1.64) | -0.93 (-1.13 to -0.73) |
| Turkey | Acute myeloid leukemia | DALYs | 2.76 (1.233 to 4.936) | 6.17 (2.78 to 10.92) | 6.393 (3.215 to 10.544) | 6.87 (3.45 to 11.36) | 1.32 (0.62 to 2.47) | 0.12 (-0.01 to 0.26) |
| Turkey | Acute lymphoid leukemia | DALYs | 1.061 (0.404 to 2.172) | 2.12 (0.83 to 4.28) | 1.832 (0.77 to 3.189) | 1.95 (0.83 to 3.39) | 0.73 (0.11 to 1.83) | -0.72 (-1.01 to -0.43) |
| Turkey | Chronic myeloid leukemia | DALYs | 1.065 (0.454 to 1.913) | 2.58 (1.1 to 4.63) | 0.821 (0.394 to 1.522) | 0.92 (0.44 to 1.66) | -0.23 (-0.49 to 0.23) | -4.34 (-4.61 to -4.07) |
| Turkey | Chronic lymphoid leukemia | DALYs | 0.577 (0.248 to 1.05) | 1.51 (0.65 to 2.74) | 1.679 (0.779 to 3.07) | 1.9 (0.88 to 3.47) | 1.91 (0.92 to 3.51) | 0.72 (0.57 to 0.88) |
| Turkey | Other leukemia | DALYs | 2.095 (0.79 to 3.859) | 5.06 (1.96 to 9.09) | 2.708 (1.282 to 5.128) | 3.03 (1.43 to 5.76) | 0.29 (-0.16 to 1.65) | -2.07 (-2.31 to -1.83) |
| Turkmenistan | Leukemia | death | 0.004 (0.002 to 0.008) | 0.19 (0.09 to 0.34) | 0.011 (0.006 to 0.019) | 0.27 (0.13 to 0.45) | 1.57 (0.95 to 2.67) | 1 (0.73 to 1.26) |
| Turkmenistan | Acute myeloid leukemia | death | 0.001 (0 to 0.002) | 0.04 (0.02 to 0.07) | 0.003 (0.002 to 0.006) | 0.08 (0.04 to 0.14) | 2.61 (1.56 to 4.53) | 2.57 (2.2 to 2.95) |
| Turkmenistan | Acute lymphoid leukemia | death | 0.001 (0 to 0.001) | 0.02 (0.01 to 0.04) | 0.002 (0.001 to 0.003) | 0.03 (0.02 to 0.06) | 1.83 (0.99 to 3.36) | 1.75 (1.48 to 2.02) |
| Turkmenistan | Chronic myeloid leukemia | death | 0 (0 to 0.001) | 0.02 (0.01 to 0.04) | 0.001 (0 to 0.002) | 0.02 (0.01 to 0.04) | 1.21 (0.49 to 2.41) | 0.2 (-0.07 to 0.48) |
| Turkmenistan | Chronic lymphoid leukemia | death | 0 (0 to 0.001) | 0.02 (0.01 to 0.03) | 0.001 (0.001 to 0.002) | 0.03 (0.01 to 0.05) | 2.41 (1.27 to 4.37) | 1.94 (1.75 to 2.13) |
| Turkmenistan | Other leukemia | death | 0.002 (0.001 to 0.004) | 0.09 (0.04 to 0.16) | 0.004 (0.002 to 0.007) | 0.1 (0.05 to 0.17) | 0.97 (0.47 to 1.87) | -0.06 (-0.33 to 0.2) |
| Turkmenistan | Leukemia | DALYs | 0.171 (0.076 to 0.308) | 6.68 (3.04 to 11.73) | 0.415 (0.213 to 0.721) | 8.68 (4.47 to 14.97) | 1.42 (0.79 to 2.6) | 0.86 (0.53 to 1.2) |
| Turkmenistan | Acute myeloid leukemia | DALYs | 0.039 (0.017 to 0.073) | 1.49 (0.66 to 2.75) | 0.135 (0.065 to 0.244) | 2.72 (1.32 to 4.96) | 2.45 (1.41 to 4.48) | 2.48 (2.06 to 2.91) |
| Turkmenistan | Acute lymphoid leukemia | DALYs | 0.025 (0.01 to 0.047) | 0.9 (0.39 to 1.66) | 0.068 (0.033 to 0.122) | 1.35 (0.66 to 2.42) | 1.68 (0.85 to 3.31) | 1.65 (1.3 to 2.01) |
| Turkmenistan | Chronic myeloid leukemia | DALYs | 0.016 (0.007 to 0.029) | 0.66 (0.29 to 1.18) | 0.034 (0.016 to 0.059) | 0.71 (0.34 to 1.22) | 1.11 (0.4 to 2.35) | 0.06 (-0.26 to 0.37) |
| Turkmenistan | Chronic lymphoid leukemia | DALYs | 0.011 (0.005 to 0.019) | 0.49 (0.22 to 0.88) | 0.034 (0.016 to 0.06) | 0.8 (0.37 to 1.41) | 2.19 (1.09 to 4.09) | 1.68 (1.44 to 1.91) |
[truncated: 35,163 more chars]
