# Supplementary material for: Global burden and trends of leukemia attributable to high body mass index risk in adults over the past 30 years
Source: Front Oncol. 2024 Jun 19;14:1404135. doi: 10.3389/fonc.2024.1404135 (PMC11219942; doi:10.3389/fonc.2024.1404135)
Supplement: Supplementary file 2 [file Table_2.docx]

| **location** | **Causes** | **Measure** | **Cases in 1990 No.×103** | **ASR in 1990 per 100000** | **Cases in 2019 No.×103** | **ASR in 2019 per 100000** | **Change in absolute number (%)** | **EAPC from 1990 and 2019** |
| --- | --- | --- | --- | --- | --- | --- | --- | --- |
| Global | Acute myeloid leukemia | death | 2.41 (1.06 to 4.25) | 0.06 (0.03 to 0.11) | 7.01 (3.44 to 11.72) | 0.09 (0.04 to 0.15) | 1.9 (1.47 to 2.4) | 1.34 (1.25 to 1.43) |
| High SDI | Acute myeloid leukemia | death | 1.3 (0.58 to 2.26) | 0.13 (0.06 to 0.22) | 3.38 (1.65 to 5.6) | 0.18 (0.09 to 0.3) | 1.6 (1.19 to 2.04) | 1.44 (1.32 to 1.56) |
| High-middle SDI | Acute myeloid leukemia | death | 0.75 (0.34 to 1.3) | 0.07 (0.03 to 0.12) | 1.84 (0.88 to 3.05) | 0.09 (0.04 to 0.15) | 1.45 (1 to 1.87) | 1.07 (0.98 to 1.15) |
| Middle SDI | Acute myeloid leukemia | death | 0.26 (0.1 to 0.48) | 0.02 (0.01 to 0.04) | 1.19 (0.59 to 2.02) | 0.05 (0.02 to 0.08) | 3.67 (2.7 to 5.2) | 2.83 (2.77 to 2.89) |
| Low-middle SDI | Acute myeloid leukemia | death | 0.08 (0.03 to 0.17) | 0.01 (0 to 0.02) | 0.47 (0.22 to 0.83) | 0.03 (0.02 to 0.06) | 4.97 (3.39 to 8.22) | 3.77 (3.71 to 3.84) |
| Low SDI | Acute myeloid leukemia | death | 0.03 (0.01 to 0.06) | 0.01 (0 to 0.02) | 0.13 (0.06 to 0.25) | 0.02 (0.01 to 0.04) | 4.03 (2.71 to 6.92) | 3.05 (2.86 to 3.24) |
| Andean Latin America | Acute myeloid leukemia | death | 0.01 (0 to 0.02) | 0.04 (0.02 to 0.08) | 0.07 (0.03 to 0.11) | 0.11 (0.05 to 0.2) | 5.52 (2.74 to 8.74) | 3.8 (3.6 to 3.99) |
| Australasia | Acute myeloid leukemia | death | 0.05 (0.02 to 0.08) | 0.19 (0.09 to 0.33) | 0.13 (0.06 to 0.2) | 0.26 (0.13 to 0.42) | 1.81 (1.34 to 2.41) | 0.94 (0.88 to 0.99) |
| Caribbean | Acute myeloid leukemia | death | 0.02 (0.01 to 0.03) | 0.06 (0.03 to 0.1) | 0.05 (0.02 to 0.08) | 0.09 (0.05 to 0.15) | 1.71 (1.23 to 2.42) | 1.67 (1.52 to 1.83) |
| Central Asia | Acute myeloid leukemia | death | 0.03 (0.01 to 0.06) | 0.06 (0.03 to 0.12) | 0.08 (0.04 to 0.13) | 0.09 (0.05 to 0.15) | 1.34 (0.84 to 2.01) | 1.65 (1.31 to 2) |
| Central Europe | Acute myeloid leukemia | death | 0.16 (0.08 to 0.29) | 0.11 (0.05 to 0.2) | 0.38 (0.19 to 0.63) | 0.19 (0.09 to 0.31) | 1.33 (0.38 to 1.95) | 2.1 (1.93 to 2.27) |
| Central Latin America | Acute myeloid leukemia | death | 0.06 (0.02 to 0.1) | 0.05 (0.02 to 0.1) | 0.25 (0.12 to 0.42) | 0.1 (0.05 to 0.17) | 3.43 (2.6 to 4.6) | 2.15 (2.06 to 2.24) |
| Central Sub-Saharan Africa | Acute myeloid leukemia | death | 0 (0 to 0) | 0.01 (0 to 0.02) | 0.01 (0 to 0.02) | 0.01 (0 to 0.02) | 2.44 (1.31 to 4.47) | 0.78 (0.25 to 1.31) |
| East Asia | Acute myeloid leukemia | death | 0.06 (0.01 to 0.15) | 0.01 (0 to 0.02) | 0.36 (0.13 to 0.71) | 0.02 (0.01 to 0.04) | 5.07 (2.71 to 12.03) | 4.32 (4.14 to 4.5) |
| Eastern Europe | Acute myeloid leukemia | death | 0.27 (0.13 to 0.46) | 0.1 (0.05 to 0.17) | 0.32 (0.15 to 0.52) | 0.1 (0.05 to 0.16) | 0.15 (-0.04 to 0.4) | -0.41 (-0.59 to -0.22) |
| Eastern Sub-Saharan Africa | Acute myeloid leukemia | death | 0.01 (0 to 0.02) | 0.01 (0 to 0.02) | 0.04 (0.02 to 0.07) | 0.02 (0.01 to 0.04) | 4.81 (2.66 to 9.89) | 3.61 (3.35 to 3.87) |
| High-income Asia Pacific | Acute myeloid leukemia | death | 0.09 (0.03 to 0.2) | 0.04 (0.01 to 0.1) | 0.23 (0.08 to 0.47) | 0.05 (0.02 to 0.11) | 1.49 (1.06 to 2.12) | 0.79 (0.63 to 0.95) |
| High-income North America | Acute myeloid leukemia | death | 0.62 (0.29 to 1.04) | 0.18 (0.08 to 0.3) | 1.71 (0.9 to 2.72) | 0.28 (0.15 to 0.44) | 1.74 (1.34 to 2.27) | 1.6 (1.39 to 1.81) |
| North Africa and Middle East | Acute myeloid leukemia | death | 0.18 (0.08 to 0.32) | 0.09 (0.04 to 0.16) | 0.65 (0.35 to 1.05) | 0.13 (0.07 to 0.22) | 2.54 (1.82 to 3.8) | 1.26 (1.22 to 1.3) |
| Oceania | Acute myeloid leukemia | death | 0 (0 to 0) | 0.06 (0.02 to 0.11) | 0.01 (0 to 0.01) | 0.06 (0.03 to 0.12) | 1.42 (0.85 to 2.24) | -0.02 (-0.2 to 0.15) |
| South Asia | Acute myeloid leukemia | death | 0.06 (0.02 to 0.13) | 0.01 (0 to 0.02) | 0.41 (0.19 to 0.74) | 0.03 (0.01 to 0.05) | 6.18 (3.81 to 12.26) | 3.91 (3.75 to 4.07) |
| Southeast Asia | Acute myeloid leukemia | death | 0.04 (0.01 to 0.1) | 0.01 (0 to 0.03) | 0.28 (0.12 to 0.53) | 0.04 (0.02 to 0.08) | 5.43 (3.46 to 10.04) | 3.93 (3.87 to 4) |
| Southern Latin America | Acute myeloid leukemia | death | 0.03 (0.01 to 0.06) | 0.07 (0.03 to 0.12) | 0.1 (0.05 to 0.17) | 0.12 (0.06 to 0.21) | 2.19 (1.68 to 3.28) | 1.99 (1.67 to 2.3) |
| Southern Sub-Saharan Africa | Acute myeloid leukemia | death | 0 (0 to 0.01) | 0.01 (0.01 to 0.02) | 0.01 (0.01 to 0.02) | 0.02 (0.01 to 0.03) | 1.87 (0.98 to 2.83) | 1.43 (1.33 to 1.53) |
| Tropical Latin America | Acute myeloid leukemia | death | 0.07 (0.03 to 0.13) | 0.07 (0.03 to 0.13) | 0.31 (0.16 to 0.51) | 0.13 (0.06 to 0.21) | 3.28 (2.58 to 4.62) | 2.29 (2.15 to 2.44) |
| Western Europe | Acute myeloid leukemia | death | 0.62 (0.28 to 1.09) | 0.11 (0.05 to 0.2) | 1.58 (0.75 to 2.71) | 0.18 (0.08 to 0.3) | 1.53 (0.96 to 1.97) | 1.8 (1.66 to 1.94) |
| Western Sub-Saharan Africa | Acute myeloid leukemia | death | 0.01 (0 to 0.02) | 0.01 (0 to 0.02) | 0.06 (0.03 to 0.1) | 0.02 (0.01 to 0.04) | 4.18 (2.83 to 7.03) | 3.08 (3.02 to 3.14) |
| Global | Acute lymphoid leukemia | death | 0.79 (0.33 to 1.46) | 0.02 (0.01 to 0.03) | 2.01 (0.94 to 3.42) | 0.02 (0.01 to 0.04) | 1.53 (1.13 to 1.98) | 1.08 (1.02 to 1.15) |
| High SDI | Acute lymphoid leukemia | death | 0.25 (0.11 to 0.45) | 0.03 (0.01 to 0.05) | 0.43 (0.22 to 0.69) | 0.03 (0.01 to 0.04) | 0.69 (0.49 to 1.04) | 0.17 (0.11 to 0.23) |
| High-middle SDI | Acute lymphoid leukemia | death | 0.33 (0.15 to 0.58) | 0.03 (0.01 to 0.05) | 0.63 (0.3 to 1.07) | 0.03 (0.02 to 0.06) | 0.93 (0.63 to 1.23) | 0.47 (0.38 to 0.56) |
| Middle SDI | Acute lymphoid leukemia | death | 0.16 (0.06 to 0.34) | 0.01 (0 to 0.03) | 0.7 (0.32 to 1.26) | 0.03 (0.01 to 0.05) | 3.33 (1.96 to 4.99) | 3.04 (2.93 to 3.14) |
| Low-middle SDI | Acute lymphoid leukemia | death | 0.04 (0.01 to 0.09) | 0 (0 to 0.01) | 0.19 (0.08 to 0.35) | 0.01 (0.01 to 0.02) | 3.98 (2.49 to 6.93) | 3.43 (3.34 to 3.53) |
| Low SDI | Acute lymphoid leukemia | death | 0.01 (0 to 0.03) | 0 (0 to 0.01) | 0.05 (0.02 to 0.1) | 0.01 (0 to 0.01) | 3.33 (1.98 to 6.51) | 2.37 (2.19 to 2.55) |
| Andean Latin America | Acute lymphoid leukemia | death | 0.01 (0 to 0.02) | 0.03 (0.01 to 0.07) | 0.04 (0.02 to 0.07) | 0.07 (0.03 to 0.13) | 3.88 (1.77 to 6.56) | 2.79 (2.65 to 2.92) |
| Australasia | Acute lymphoid leukemia | death | 0.01 (0 to 0.01) | 0.03 (0.02 to 0.06) | 0.01 (0.01 to 0.02) | 0.03 (0.02 to 0.05) | 0.63 (0.34 to 1.25) | -0.93 (-1.17 to -0.69) |
| Caribbean | Acute lymphoid leukemia | death | 0.01 (0 to 0.01) | 0.02 (0.01 to 0.04) | 0.01 (0.01 to 0.02) | 0.03 (0.01 to 0.05) | 1.17 (0.75 to 1.71) | 0.97 (0.86 to 1.08) |
| Central Asia | Acute lymphoid leukemia | death | 0.02 (0.01 to 0.03) | 0.04 (0.02 to 0.06) | 0.04 (0.02 to 0.06) | 0.04 (0.02 to 0.07) | 0.84 (0.52 to 1.33) | 0.57 (0.43 to 0.72) |
| Central Europe | Acute lymphoid leukemia | death | 0.06 (0.03 to 0.09) | 0.04 (0.02 to 0.07) | 0.07 (0.03 to 0.11) | 0.04 (0.02 to 0.06) | 0.17 (-0.01 to 0.42) | -0.31 (-0.41 to -0.21) |
| Central Latin America | Acute lymphoid leukemia | death | 0.05 (0.02 to 0.09) | 0.04 (0.02 to 0.08) | 0.21 (0.1 to 0.36) | 0.08 (0.04 to 0.14) | 3.39 (2.04 to 4.78) | 2.54 (2.38 to 2.71) |
| Central Sub-Saharan Africa | Acute lymphoid leukemia | death | 0 (0 to 0) | 0 (0 to 0.01) | 0 (0 to 0.01) | 0 (0 to 0.01) | 2.26 (1.3 to 4.01) | 0.32 (-0.14 to 0.79) |
| East Asia | Acute lymphoid leukemia | death | 0.08 (0.01 to 0.21) | 0.01 (0 to 0.02) | 0.44 (0.15 to 0.87) | 0.02 (0.01 to 0.04) | 4.76 (1.91 to 12.81) | 4.77 (4.4 to 5.14) |
| Eastern Europe | Acute lymphoid leukemia | death | 0.16 (0.07 to 0.27) | 0.06 (0.03 to 0.1) | 0.19 (0.1 to 0.31) | 0.06 (0.03 to 0.1) | 0.22 (0.04 to 0.47) | 0.15 (0.01 to 0.29) |
| Eastern Sub-Saharan Africa | Acute lymphoid leukemia | death | 0 (0 to 0.01) | 0 (0 to 0.01) | 0.02 (0.01 to 0.04) | 0.01 (0 to 0.02) | 3.77 (1.6 to 9.16) | 2.63 (2.4 to 2.87) |
| High-income Asia Pacific | Acute lymphoid leukemia | death | 0.02 (0.01 to 0.05) | 0.01 (0 to 0.03) | 0.04 (0.01 to 0.07) | 0.01 (0 to 0.02) | 0.46 (0.15 to 0.9) | -0.66 (-0.77 to -0.55) |
| High-income North America | Acute lymphoid leukemia | death | 0.11 (0.05 to 0.19) | 0.03 (0.02 to 0.06) | 0.22 (0.12 to 0.34) | 0.04 (0.02 to 0.06) | 0.9 (0.7 to 1.25) | 0.63 (0.47 to 0.79) |
| North Africa and Middle East | Acute lymphoid leukemia | death | 0.07 (0.03 to 0.13) | 0.03 (0.01 to 0.06) | 0.2 (0.1 to 0.34) | 0.04 (0.02 to 0.06) | 1.99 (1.26 to 3.15) | 0.74 (0.63 to 0.85) |
| Oceania | Acute lymphoid leukemia | death | 0 (0 to 0) | 0.01 (0.01 to 0.03) | 0 (0 to 0) | 0.01 (0.01 to 0.03) | 1.31 (0.76 to 2.08) | -0.49 (-0.8 to -0.19) |
| South Asia | Acute lymphoid leukemia | death | 0.02 (0.01 to 0.05) | 0 (0 to 0.01) | 0.1 (0.05 to 0.18) | 0.01 (0 to 0.01) | 3.88 (2.33 to 7.83) | 2.73 (2.53 to 2.94) |
| Southeast Asia | Acute lymphoid leukemia | death | 0.02 (0.01 to 0.05) | 0.01 (0 to 0.02) | 0.1 (0.04 to 0.18) | 0.01 (0.01 to 0.03) | 3.84 (2.24 to 7.43) | 3.2 (3.14 to 3.26) |
| Southern Latin America | Acute lymphoid leukemia | death | 0.01 (0 to 0.02) | 0.03 (0.01 to 0.05) | 0.04 (0.02 to 0.06) | 0.05 (0.02 to 0.08) | 1.78 (1.24 to 2.79) | 1.65 (1.39 to 1.92) |
| Southern Sub-Saharan Africa | Acute lymphoid leukemia | death | 0 (0 to 0) | 0.01 (0 to 0.01) | 0.01 (0 to 0.01) | 0.01 (0 to 0.01) | 1.48 (0.37 to 2.68) | 1.1 (0.91 to 1.3) |
| Tropical Latin America | Acute lymphoid leukemia | death | 0.02 (0.01 to 0.04) | 0.02 (0.01 to 0.03) | 0.08 (0.04 to 0.13) | 0.03 (0.02 to 0.05) | 2.69 (1.87 to 4.13) | 2.07 (1.9 to 2.23) |
| Western Europe | Acute lymphoid leukemia | death | 0.12 (0.05 to 0.21) | 0.02 (0.01 to 0.04) | 0.18 (0.08 to 0.3) | 0.02 (0.01 to 0.04) | 0.48 (0.28 to 0.94) | -0.21 (-0.34 to -0.08) |
| Western Sub-Saharan Africa | Acute lymphoid leukemia | death | 0 (0 to 0.01) | 0 (0 to 0.01) | 0.02 (0.01 to 0.04) | 0.01 (0 to 0.01) | 3.74 (2.29 to 6.83) | 2.29 (2.21 to 2.37) |
| Global | Chronic myeloid leukemia | death | 1.28 (0.56 to 2.25) | 0.03 (0.01 to 0.06) | 1.83 (0.92 to 3.06) | 0.02 (0.01 to 0.04) | 0.43 (0.27 to 0.7) | -1.74 (-1.91 to -1.56) |
| High SDI | Chronic myeloid leukemia | death | 0.57 (0.26 to 0.99) | 0.06 (0.03 to 0.1) | 0.44 (0.22 to 0.72) | 0.02 (0.01 to 0.04) | -0.23 (-0.32 to -0.1) | -3.76 (-4.12 to -3.41) |
| High-middle SDI | Chronic myeloid leukemia | death | 0.4 (0.18 to 0.68) | 0.04 (0.02 to 0.06) | 0.46 (0.23 to 0.75) | 0.02 (0.01 to 0.04) | 0.15 (0.01 to 0.34) | -2.2 (-2.47 to -1.93) |
| Middle SDI | Chronic myeloid leukemia | death | 0.17 (0.07 to 0.32) | 0.01 (0.01 to 0.03) | 0.47 (0.24 to 0.78) | 0.02 (0.01 to 0.03) | 1.72 (1.21 to 2.61) | 0.57 (0.47 to 0.67) |
| Low-middle SDI | Chronic myeloid leukemia | death | 0.08 (0.03 to 0.19) | 0.01 (0 to 0.03) | 0.31 (0.14 to 0.56) | 0.02 (0.01 to 0.04) | 2.66 (1.65 to 5.07) | 1.72 (1.63 to 1.81) |
| Low SDI | Chronic myeloid leukemia | death | 0.05 (0.01 to 0.13) | 0.02 (0.01 to 0.05) | 0.16 (0.07 to 0.3) | 0.03 (0.01 to 0.05) | 1.96 (1.01 to 4.58) | 1.04 (0.87 to 1.22) |
| Andean Latin America | Chronic myeloid leukemia | death | 0 (0 to 0.01) | 0.02 (0.01 to 0.04) | 0.02 (0.01 to 0.03) | 0.03 (0.02 to 0.05) | 3.02 (1.93 to 4.89) | 2.09 (1.88 to 2.3) |
| Australasia | Chronic myeloid leukemia | death | 0.02 (0.01 to 0.03) | 0.08 (0.04 to 0.13) | 0.02 (0.01 to 0.03) | 0.03 (0.02 to 0.05) | -0.16 (-0.31 to 0.08) | -4 (-4.35 to -3.65) |
| Caribbean | Chronic myeloid leukemia | death | 0.01 (0.01 to 0.02) | 0.05 (0.02 to 0.08) | 0.02 (0.01 to 0.04) | 0.04 (0.02 to 0.07) | 0.62 (0.33 to 1.02) | -0.52 (-0.66 to -0.38) |
| Central Asia | Chronic myeloid leukemia | death | 0.01 (0.01 to 0.02) | 0.03 (0.01 to 0.05) | 0.02 (0.01 to 0.03) | 0.03 (0.01 to 0.04) | 0.46 (0.18 to 0.87) | -0.39 (-0.51 to -0.26) |
| Central Europe | Chronic myeloid leukemia | death | 0.08 (0.04 to 0.13) | 0.06 (0.03 to 0.09) | 0.07 (0.03 to 0.11) | 0.03 (0.02 to 0.05) | -0.19 (-0.34 to 0.17) | -2.5 (-2.81 to -2.18) |
| Central Latin America | Chronic myeloid leukemia | death | 0.03 (0.01 to 0.05) | 0.03 (0.01 to 0.06) | 0.07 (0.04 to 0.12) | 0.03 (0.01 to 0.05) | 1.31 (0.9 to 1.94) | -0.86 (-1.14 to -0.59) |
| Central Sub-Saharan Africa | Chronic myeloid leukemia | death | 0 (0 to 0.01) | 0.01 (0 to 0.03) | 0.01 (0 to 0.02) | 0.01 (0.01 to 0.03) | 1.65 (0.8 to 3.34) | 0.01 (-0.49 to 0.51) |
| East Asia | Chronic myeloid leukemia | death | 0.03 (0.01 to 0.07) | 0 (0 to 0.01) | 0.06 (0.02 to 0.11) | 0 (0 to 0.01) | 1.21 (0.44 to 3.52) | 0.01 (-0.08 to 0.1) |
| Eastern Europe | Chronic myeloid leukemia | death | 0.13 (0.06 to 0.23) | 0.05 (0.02 to 0.08) | 0.16 (0.08 to 0.26) | 0.05 (0.02 to 0.08) | 0.24 (0.01 to 0.52) | -0.25 (-0.64 to 0.14) |
| Eastern Sub-Saharan Africa | Chronic myeloid leukemia | death | 0.02 (0.01 to 0.07) | 0.03 (0.01 to 0.07) | 0.07 (0.03 to 0.13) | 0.03 (0.01 to 0.07) | 1.75 (0.48 to 6.09) | 0.76 (0.45 to 1.07) |
| High-income Asia Pacific | Chronic myeloid leukemia | death | 0.04 (0.01 to 0.08) | 0.02 (0.01 to 0.04) | 0.02 (0.01 to 0.05) | 0.01 (0 to 0.01) | -0.32 (-0.43 to -0.13) | -4.71 (-4.99 to -4.42) |
| High-income North America | Chronic myeloid leukemia | death | 0.25 (0.12 to 0.41) | 0.07 (0.04 to 0.12) | 0.19 (0.1 to 0.3) | 0.03 (0.02 to 0.05) | -0.24 (-0.33 to -0.1) | -3.8 (-4.18 to -3.42) |
| North Africa and Middle East | Chronic myeloid leukemia | death | 0.13 (0.06 to 0.22) | 0.07 (0.03 to 0.12) | 0.27 (0.13 to 0.44) | 0.06 (0.03 to 0.09) | 1.09 (0.68 to 1.76) | -0.78 (-0.88 to -0.69) |
| Oceania | Chronic myeloid leukemia | death | 0 (0 to 0) | 0.04 (0.01 to 0.07) | 0 (0 to 0) | 0.03 (0.01 to 0.06) | 0.92 (0.46 to 1.57) | -1.15 (-1.45 to -0.84) |
| South Asia | Chronic myeloid leukemia | death | 0.09 (0.03 to 0.21) | 0.01 (0 to 0.03) | 0.38 (0.17 to 0.69) | 0.02 (0.01 to 0.04) | 3.09 (1.78 to 6.35) | 1.81 (1.66 to 1.96) |
| Southeast Asia | Chronic myeloid leukemia | death | 0.02 (0.01 to 0.05) | 0.01 (0 to 0.02) | 0.09 (0.04 to 0.16) | 0.01 (0.01 to 0.03) | 3.2 (1.88 to 6.26) | 2.48 (2.41 to 2.55) |
| Southern Latin America | Chronic myeloid leukemia | death | 0.02 (0.01 to 0.04) | 0.05 (0.02 to 0.09) | 0.03 (0.01 to 0.04) | 0.03 (0.01 to 0.05) | 0.12 (-0.08 to 0.49) | -2.42 (-2.92 to -1.92) |
| Southern Sub-Saharan Africa | Chronic myeloid leukemia | death | 0 (0 to 0) | 0 (0 to 0.01) | 0 (0 to 0) | 0 (0 to 0.01) | 0.74 (0.33 to 1.33) | -0.2 (-0.48 to 0.08) |
| Tropical Latin America | Chronic myeloid leukemia | death | 0.04 (0.02 to 0.07) | 0.04 (0.02 to 0.07) | 0.06 (0.03 to 0.1) | 0.03 (0.01 to 0.04) | 0.61 (0.35 to 1.1) | -1.78 (-2.21 to -1.34) |
| Western Europe | Chronic myeloid leukemia | death | 0.33 (0.15 to 0.57) | 0.06 (0.03 to 0.1) | 0.22 (0.11 to 0.39) | 0.02 (0.01 to 0.04) | -0.31 (-0.4 to -0.19) | -4.14 (-4.49 to -3.79) |
| Western Sub-Saharan Africa | Chronic myeloid leukemia | death | 0.01 (0 to 0.03) | 0.01 (0 to 0.03) | 0.06 (0.03 to 0.11) | 0.02 (0.01 to 0.04) | 3.08 (1.94 to 5.3) | 2.11 (2.06 to 2.16) |
| Global | Chronic lymphoid leukemia | death | 1.39 (0.62 to 2.44) | 0.04 (0.02 to 0.07) | 3.62 (1.76 to 6.15) | 0.05 (0.02 to 0.08) | 1.6 (1.34 to 2.01) | 0.39 (0.25 to 0.54) |
| High SDI | Chronic lymphoid leukemia | death | 0.75 (0.34 to 1.3) | 0.07 (0.03 to 0.12) | 1.44 (0.72 to 2.4) | 0.07 (0.03 to 0.11) | 0.91 (0.7 to 1.31) | -0.39 (-0.6 to -0.18) |
| High-middle SDI | Chronic lymphoid leukemia | death | 0.44 (0.21 to 0.76) | 0.04 (0.02 to 0.07) | 1.13 (0.56 to 1.87) | 0.06 (0.03 to 0.09) | 1.55 (1.24 to 2.04) | 0.89 (0.67 to 1.1) |
| Middle SDI | Chronic lymphoid leukemia | death | 0.12 (0.05 to 0.22) | 0.01 (0 to 0.02) | 0.57 (0.26 to 0.98) | 0.02 (0.01 to 0.04) | 3.83 (3.08 to 5.05) | 2.37 (2.3 to 2.45) |
| Low-middle SDI | Chronic lymphoid leukemia | death | 0.05 (0.01 to 0.12) | 0.01 (0 to 0.02) | 0.34 (0.14 to 0.63) | 0.03 (0.01 to 0.05) | 5.37 (3.67 to 9.2) | 3.47 (3.36 to 3.58) |
| Low SDI | Chronic lymphoid leukemia | death | 0.03 (0.01 to 0.06) | 0.01 (0 to 0.03) | 0.14 (0.05 to 0.28) | 0.03 (0.01 to 0.06) | 4.24 (2.9 to 7.52) | 3.19 (3.09 to 3.3) |
| Andean Latin America | Chronic lymphoid leukemia | death | 0 (0 to 0) | 0.01 (0 to 0.02) | 0.01 (0.01 to 0.02) | 0.02 (0.01 to 0.04) | 5.89 (3.8 to 9.44) | 3.7 (3.5 to 3.91) |
| Australasia | Chronic lymphoid leukemia | death | 0.02 (0.01 to 0.03) | 0.08 (0.04 to 0.14) | 0.05 (0.02 to 0.08) | 0.09 (0.04 to 0.15) | 1.5 (1.07 to 2.15) | -0.05 (-0.21 to 0.11) |
| Caribbean | Chronic lymphoid leukemia | death | 0.01 (0 to 0.01) | 0.03 (0.01 to 0.05) | 0.02 (0.01 to 0.03) | 0.03 (0.02 to 0.06) | 1.64 (1.14 to 2.31) | 1.09 (0.9 to 1.29) |
| Central Asia | Chronic lymphoid leukemia | death | 0.01 (0.01 to 0.02) | 0.03 (0.01 to 0.05) | 0.03 (0.01 to 0.05) | 0.04 (0.02 to 0.07) | 1.22 (0.72 to 2.01) | 1.53 (1.39 to 1.66) |
| Central Europe | Chronic lymphoid leukemia | death | 0.1 (0.05 to 0.16) | 0.07 (0.03 to 0.11) | 0.3 (0.16 to 0.5) | 0.13 (0.07 to 0.22) | 2.13 (1.58 to 3.12) | 2.88 (2.46 to 3.3) |
| Central Latin America | Chronic lymphoid leukemia | death | 0.01 (0.01 to 0.02) | 0.02 (0.01 to 0.03) | 0.05 (0.03 to 0.09) | 0.02 (0.01 to 0.04) | 3.21 (2.38 to 4.51) | 1.19 (1.06 to 1.32) |
| Central Sub-Saharan Africa | Chronic lymphoid leukemia | death | 0 (0 to 0.01) | 0.01 (0 to 0.02) | 0.01 (0 to 0.03) | 0.03 (0.01 to 0.06) | 4.84 (3.01 to 8.05) | 3.36 (3.24 to 3.49) |
| East Asia | Chronic lymphoid leukemia | death | 0.03 (0.01 to 0.08) | 0 (0 to 0.01) | 0.22 (0.08 to 0.43) | 0.01 (0 to 0.02) | 6.44 (3.78 to 14.61) | 5.03 (4.77 to 5.28) |
| Eastern Europe | Chronic lymphoid leukemia | death | 0.2 (0.09 to 0.33) | 0.07 (0.03 to 0.12) | 0.37 (0.19 to 0.59) | 0.1 (0.05 to 0.17) | 0.85 (0.53 to 1.29) | 1.42 (1.22 to 1.62) |
| Eastern Sub-Saharan Africa | Chronic lymphoid leukemia | death | 0.01 (0 to 0.03) | 0.02 (0 to 0.04) | 0.06 (0.02 to 0.12) | 0.04 (0.02 to 0.09) | 4.79 (3.1 to 9.24) | 3.85 (3.62 to 4.08) |
| High-income Asia Pacific | Chronic lymphoid leukemia | death | 0.01 (0 to 0.01) | 0 (0 to 0.01) | 0.02 (0 to 0.03) | 0 (0 to 0.01) | 1.33 (0.96 to 1.98) | -0.38 (-0.49 to -0.26) |
| High-income North America | Chronic lymphoid leukemia | death | 0.36 (0.16 to 0.62) | 0.1 (0.05 to 0.17) | 0.71 (0.36 to 1.14) | 0.1 (0.05 to 0.17) | 0.95 (0.69 to 1.43) | -0.2 (-0.46 to 0.06) |
| North Africa and Middle East | Chronic lymphoid leukemia | death | 0.04 (0.02 to 0.08) | 0.03 (0.01 to 0.05) | 0.18 (0.09 to 0.29) | 0.05 (0.02 to 0.07) | 3.08 (2.22 to 4.52) | 1.86 (1.77 to 1.94) |
| Oceania | Chronic lymphoid leukemia | death | 0 (0 to 0) | 0 (0 to 0.01) | 0 (0 to 0) | 0 (0 to 0.01) | 1.54 (0.94 to 2.43) | -0.25 (-0.44 to -0.07) |
| South Asia | Chronic lymphoid leukemia | death | 0.05 (0.01 to 0.13) | 0.01 (0 to 0.03) | 0.4 (0.16 to 0.76) | 0.03 (0.01 to 0.06) | 6.46 (4.15 to 12.73) | 3.44 (3.23 to 3.66) |
| Southeast Asia | Chronic lymphoid leukemia | death | 0.01 (0 to 0.02) | 0 (0 to 0.01) | 0.06 (0.03 to 0.12) | 0.01 (0 to 0.02) | 6.77 (4.48 to 12.61) | 4.31 (4.19 to 4.42) |
| Southern Latin America | Chronic lymphoid leukemia | death | 0.01 (0.01 to 0.02) | 0.03 (0.01 to 0.05) | 0.03 (0.02 to 0.06) | 0.04 (0.02 to 0.07) | 1.54 (1.05 to 2.41) | 0.51 (0.09 to 0.94) |
| Southern Sub-Saharan Africa | Chronic lymphoid leukemia | death | 0.04 (0.02 to 0.07) | 0.15 (0.06 to 0.26) | 0.11 (0.05 to 0.17) | 0.21 (0.09 to 0.34) | 1.69 (1.38 to 2.21) | 1.38 (1.13 to 1.62) |
| Tropical Latin America | Chronic lymphoid leukemia | death | 0.02 (0.01 to 0.03) | 0.02 (0.01 to 0.04) | 0.08 (0.04 to 0.14) | 0.04 (0.02 to 0.06) | 3.95 (3.1 to 5.37) | 2.16 (1.99 to 2.33) |
| Western Europe | Chronic lymphoid leukemia | death | 0.44 (0.2 to 0.78) | 0.07 (0.03 to 0.13) | 0.84 (0.4 to 1.47) | 0.08 (0.04 to 0.14) | 0.89 (0.67 to 1.28) | 0.02 (-0.2 to 0.24) |
| Western Sub-Saharan Africa | Chronic lymphoid leukemia | death | 0.02 (0.01 to 0.03) | 0.02 (0.01 to 0.04) | 0.07 (0.03 to 0.13) | 0.04 (0.02 to 0.08) | 3.31 (2.27 to 5.5) | 2.66 (2.61 to 2.7) |
| Global | Other leukemia | death | 3.47 (1.37 to 6.59) | 0.09 (0.04 to 0.17) | 7.27 (3.48 to 12.66) | 0.09 (0.04 to 0.16) | 1.09 (0.76 to 1.62) | -0.03 (-0.1 to 0.04) |
| High SDI | Other leukemia | death | 1.11 (0.48 to 1.95) | 0.11 (0.05 to 0.19) | 1.98 (0.98 to 3.36) | 0.1 (0.05 to 0.17) | 0.78 (0.58 to 1.09) | -0.3 (-0.34 to -0.26) |
| High-middle SDI | Other leukemia | death | 1.19 (0.49 to 2.18) | 0.11 (0.05 to 0.21) | 2.01 (0.94 to 3.52) | 0.1 (0.05 to 0.18) | 0.69 (0.46 to 1.06) | -0.57 (-0.69 to -0.44) |
| Middle SDI | Other leukemia | death | 0.89 (0.29 to 1.91) | 0.08 (0.03 to 0.17) | 2.35 (1.08 to 4.19) | 0.1 (0.04 to 0.17) | 1.65 (0.97 to 3.16) | 0.65 (0.56 to 0.75) |
| Low-middle SDI | Other leukemia | death | 0.21 (0.06 to 0.47) | 0.03 (0.01 to 0.07) | 0.68 (0.31 to 1.27) | 0.05 (0.02 to 0.09) | 2.31 (1.42 to 4.61) | 1.54 (1.46 to 1.61) |
| Low SDI | Other leukemia | death | 0.07 (0.02 to 0.17) | 0.03 (0.01 to 0.06) | 0.24 (0.1 to 0.45) | 0.04 (0.02 to 0.08) | 2.18 (1.48 to 3.88) | 1.41 (1.27 to 1.55) |
| Andean Latin America | Other leukemia | death | 0.03 (0.01 to 0.06) | 0.15 (0.06 to 0.27) | 0.1 (0.05 to 0.19) | 0.18 (0.09 to 0.33) | 2.06 (1.26 to 3.46) | 0.68 (0.47 to 0.89) |
| Australasia | Other leukemia | death | 0.01 (0 to 0.02) | 0.05 (0.02 to 0.08) | 0.04 (0.02 to 0.06) | 0.07 (0.03 to 0.11) | 2.4 (1.59 to 3.34) | 1.98 (1.62 to 2.34) |
| Caribbean | Other leukemia | death | 0.03 (0.01 to 0.05) | 0.1 (0.04 to 0.18) | 0.07 (0.03 to 0.12) | 0.13 (0.06 to 0.22) | 1.47 (0.99 to 2.2) | 1.05 (0.94 to 1.16) |
| Central Asia | Other leukemia | death | 0.06 (0.03 to 0.1) | 0.11 (0.05 to 0.19) | 0.08 (0.04 to 0.14) | 0.11 (0.06 to 0.19) | 0.45 (0.22 to 0.85) | -0.1 (-0.23 to 0.03) |
| Central Europe | Other leukemia | death | 0.23 (0.11 to 0.38) | 0.16 (0.08 to 0.26) | 0.27 (0.14 to 0.44) | 0.12 (0.06 to 0.2) | 0.18 (-0.01 to 0.55) | -1.16 (-1.3 to -1.01) |
| Central Latin America | Other leukemia | death | 0.1 (0.04 to 0.18) | 0.1 (0.04 to 0.18) | 0.23 (0.11 to 0.39) | 0.09 (0.05 to 0.16) | 1.28 (0.83 to 1.94) | -0.43 (-0.5 to -0.36) |
| Central Sub-Saharan Africa | Other leukemia | death | 0.01 (0 to 0.02) | 0.05 (0.02 to 0.1) | 0.02 (0.01 to 0.05) | 0.04 (0.01 to 0.08) | 1.05 (0.5 to 1.94) | -1.26 (-1.62 to -0.91) |
| East Asia | Other leukemia | death | 0.67 (0.13 to 1.71) | 0.07 (0.01 to 0.17) | 1.55 (0.55 to 3.09) | 0.08 (0.03 to 0.15) | 1.3 (0.55 to 3.87) | 0.34 (0.11 to 0.57) |
| Eastern Europe | Other leukemia | death | 0.26 (0.12 to 0.45) | 0.1 (0.04 to 0.16) | 0.29 (0.14 to 0.47) | 0.09 (0.04 to 0.14) | 0.1 (-0.05 to 0.32) | -0.93 (-1.2 to -0.66) |
| Eastern Sub-Saharan Africa | Other leukemia | death | 0.02 (0 to 0.04) | 0.02 (0.01 to 0.05) | 0.06 (0.02 to 0.1) | 0.03 (0.01 to 0.06) | 2.36 (1.28 to 5.31) | 1.63 (1.52 to 1.74) |
| High-income Asia Pacific | Other leukemia | death | 0.09 (0.03 to 0.19) | 0.04 (0.01 to 0.1) | 0.14 (0.04 to 0.3) | 0.03 (0.01 to 0.06) | 0.57 (0.32 to 1) | -1.8 (-1.94 to -1.67) |
| High-income North America | Other leukemia | death | 0.55 (0.26 to 0.93) | 0.16 (0.07 to 0.26) | 1.01 (0.53 to 1.62) | 0.16 (0.08 to 0.25) | 0.82 (0.61 to 1.16) | -0.07 (-0.22 to 0.08) |
| North Africa and Middle East | Other leukemia | death | 0.35 (0.15 to 0.61) | 0.2 (0.09 to 0.36) | 0.95 (0.49 to 1.61) | 0.22 (0.12 to 0.38) | 1.74 (1.19 to 3.43) | 0.48 (0.38 to 0.58) |
| Oceania | Other leukemia | death | 0 (0 to 0.01) | 0.11 (0.04 to 0.22) | 0.01 (0 to 0.02) | 0.12 (0.04 to 0.24) | 1.54 (1.02 to 2.31) | 0.01 (-0.14 to 0.17) |
| South Asia | Other leukemia | death | 0.06 (0.02 to 0.14) | 0.01 (0 to 0.02) | 0.23 (0.1 to 0.44) | 0.02 (0.01 to 0.03) | 3.01 (1.66 to 7.4) | 1.5 (1.3 to 1.69) |
| Southeast Asia | Other leukemia | death | 0.17 (0.05 to 0.41) | 0.06 (0.02 to 0.14) | 0.67 (0.3 to 1.26) | 0.11 (0.05 to 0.2) | 2.88 (1.7 to 6.16) | 2.12 (2 to 2.24) |
| Southern Latin America | Other leukemia | death | 0.05 (0.02 to 0.1) | 0.12 (0.05 to 0.22) | 0.13 (0.06 to 0.22) | 0.15 (0.07 to 0.26) | 1.32 (0.95 to 2.06) | 0.62 (0.45 to 0.79) |
| Southern Sub-Saharan Africa | Other leukemia | death | 0.03 (0.01 to 0.05) | 0.09 (0.04 to 0.15) | 0.06 (0.03 to 0.1) | 0.1 (0.05 to 0.17) | 1.12 (0.7 to 1.79) | 0.68 (0.43 to 0.93) |
| Tropical Latin America | Other leukemia | death | 0.08 (0.03 to 0.14) | 0.08 (0.03 to 0.15) | 0.24 (0.12 to 0.39) | 0.1 (0.05 to 0.17) | 2.06 (1.56 to 3.02) | 0.94 (0.85 to 1.04) |
| Western Europe | Other leukemia | death | 0.63 (0.28 to 1.1) | 0.11 (0.05 to 0.19) | 0.99 (0.47 to 1.73) | 0.1 (0.05 to 0.17) | 0.58 (0.4 to 0.87) | -0.32 (-0.43 to -0.2) |
| Western Sub-Saharan Africa | Other leukemia | death | 0.04 (0.01 to 0.07) | 0.04 (0.01 to 0.08) | 0.13 (0.06 to 0.24) | 0.07 (0.03 to 0.12) | 2.75 (1.81 to 5.01) | 1.99 (1.93 to 2.06) |
| Global | Acute myeloid leukemia | DALYs | 71.16 (31.15 to 126.41) | 1.64 (0.72 to 2.9) | 186.48 (93.01 to 307.99) | 2.26 (1.13 to 3.74) | 1.62 (1.29 to 2.12) | 1.17 (1.11 to 1.23) |
| High SDI | Acute myeloid leukemia | DALYs | 33.78 (15.32 to 58.65) | 3.42 (1.56 to 5.95) | 74.23 (37.63 to 120.93) | 4.63 (2.35 to 7.51) | 1.2 (0.92 to 1.59) | 1.13 (1.01 to 1.26) |
| High-middle SDI | Acute myeloid leukemia | DALYs | 23.68 (10.66 to 40.78) | 2.07 (0.93 to 3.57) | 49.64 (24.25 to 81.82) | 2.59 (1.26 to 4.28) | 1.1 (0.8 to 1.47) | 0.68 (0.64 to 0.73) |
| Middle SDI | Acute myeloid leukemia | DALYs | 9.75 (3.92 to 18.48) | 0.71 (0.29 to 1.35) | 41.01 (20.56 to 69.31) | 1.54 (0.77 to 2.61) | 3.2 (2.3 to 4.65) | 2.76 (2.72 to 2.81) |
| Low-middle SDI | Acute myeloid leukemia | DALYs | 2.91 (0.96 to 6.26) | 0.37 (0.12 to 0.79) | 16.41 (7.91 to 29.47) | 1.02 (0.49 to 1.83) | 4.63 (3.06 to 7.89) | 3.77 (3.71 to 3.82) |
| Low SDI | Acute myeloid leukemia | DALYs | 0.98 (0.29 to 2.22) | 0.31 (0.09 to 0.69) | 5.06 (2.18 to 9.56) | 0.7 (0.31 to 1.29) | 4.15 (2.75 to 7.21) | 3.07 (2.89 to 3.25) |
| Andean Latin America | Acute myeloid leukemia | DALYs | 0.36 (0.15 to 0.68) | 1.37 (0.58 to 2.59) | 2.12 (1.04 to 3.67) | 3.49 (1.71 to 6.05) | 4.94 (2.43 to 7.97) | 3.7 (3.51 to 3.9) |
| Australasia | Acute myeloid leukemia | DALYs | 1.14 (0.53 to 1.91) | 4.94 (2.31 to 8.34) | 2.71 (1.4 to 4.32) | 6.2 (3.24 to 9.79) | 1.38 (1.04 to 1.88) | 0.68 (0.62 to 0.74) |
| Caribbean | Acute myeloid leukemia | DALYs | 0.62 (0.29 to 1.05) | 2.05 (0.95 to 3.49) | 1.47 (0.75 to 2.47) | 2.89 (1.47 to 4.85) | 1.39 (0.96 to 2.04) | 1.41 (1.28 to 1.54) |
| Central Asia | Acute myeloid leukemia | DALYs | 1.28 (0.57 to 2.35) | 2.3 (1.04 to 4.18) | 2.95 (1.47 to 4.87) | 3.19 (1.59 to 5.27) | 1.3 (0.83 to 2.02) | 1.4 (1.1 to 1.71) |
| Central Europe | Acute myeloid leukemia | DALYs | 4.82 (2.31 to 8.2) | 3.34 (1.6 to 5.7) | 9.38 (4.82 to 15.51) | 5.08 (2.61 to 8.43) | 0.94 (0.23 to 1.48) | 1.71 (1.56 to 1.86) |
| Central Latin America | Acute myeloid leukemia | DALYs | 2.19 (0.94 to 3.9) | 1.88 (0.8 to 3.29) | 8.45 (4.13 to 14.47) | 3.34 (1.63 to 5.71) | 2.85 (2.11 to 3.94) | 2.01 (1.93 to 2.09) |
| Central Sub-Saharan Africa | Acute myeloid leukemia | DALYs | 0.08 (0.03 to 0.19) | 0.27 (0.1 to 0.58) | 0.31 (0.12 to 0.59) | 0.39 (0.16 to 0.73) | 2.64 (1.46 to 4.9) | 0.96 (0.43 to 1.49) |
| East Asia | Acute myeloid leukemia | DALYs | 2.25 (0.43 to 5.72) | 0.2 (0.04 to 0.51) | 11.56 (4.32 to 22.59) | 0.59 (0.22 to 1.15) | 4.14 (2.08 to 10.35) | 4.2 (4.03 to 4.37) |
| Eastern Europe | Acute myeloid leukemia | DALYs | 8.88 (4.11 to 14.97) | 3.29 (1.52 to 5.56) | 9.11 (4.51 to 14.92) | 3.03 (1.49 to 4.97) | 0.03 (-0.14 to 0.27) | -0.77 (-1 to -0.54) |
| Eastern Sub-Saharan Africa | Acute myeloid leukemia | DALYs | 0.26 (0.07 to 0.62) | 0.25 (0.07 to 0.57) | 1.54 (0.66 to 2.91) | 0.63 (0.27 to 1.17) | 4.89 (2.67 to 10.07) | 3.54 (3.3 to 3.78) |
| High-income Asia Pacific | Acute myeloid leukemia | DALYs | 2.78 (0.79 to 6.12) | 1.37 (0.39 to 3.03) | 4.83 (1.68 to 9.73) | 1.45 (0.51 to 2.91) | 0.74 (0.49 to 1.2) | 0.27 (0.16 to 0.39) |
| High-income North America | Acute myeloid leukemia | DALYs | 16.18 (7.69 to 26.68) | 4.92 (2.35 to 8.1) | 37.96 (20.47 to 59.68) | 6.84 (3.68 to 10.66) | 1.35 (1.08 to 1.76) | 1.17 (0.95 to 1.39) |
| North Africa and Middle East | Acute myeloid leukemia | DALYs | 6.77 (3.04 to 11.93) | 2.99 (1.35 to 5.23) | 22.73 (11.94 to 36.87) | 4.14 (2.18 to 6.71) | 2.36 (1.64 to 3.56) | 1.07 (1.02 to 1.11) |
| Oceania | Acute myeloid leukemia | DALYs | 0.1 (0.04 to 0.19) | 2.18 (0.89 to 4.06) | 0.23 (0.11 to 0.43) | 2.21 (1.01 to 4.12) | 1.31 (0.75 to 2.14) | -0.21 (-0.41 to -0.02) |
| South Asia | Acute myeloid leukemia | DALYs | 2.1 (0.62 to 5.03) | 0.27 (0.08 to 0.64) | 14.11 (6.47 to 25.64) | 0.85 (0.39 to 1.54) | 5.71 (3.53 to 11.48) | 3.96 (3.81 to 4.1) |
| Southeast Asia | Acute myeloid leukemia | DALYs | 1.64 (0.52 to 3.76) | 0.47 (0.15 to 1.08) | 9.67 (4.25 to 18.59) | 1.38 (0.6 to 2.63) | 4.91 (3.1 to 9.18) | 3.88 (3.8 to 3.95) |
| Southern Latin America | Acute myeloid leukemia | DALYs | 0.96 (0.39 to 1.78) | 2.03 (0.82 to 3.75) | 2.79 (1.33 to 4.77) | 3.6 (1.71 to 6.15) | 1.89 (1.42 to 2.91) | 1.84 (1.53 to 2.14) |
| Southern Sub-Saharan Africa | Acute myeloid leukemia | DALYs | 0.22 (0.1 to 0.4) | 0.53 (0.25 to 0.96) | 0.6 (0.3 to 0.99) | 0.77 (0.4 to 1.29) | 1.77 (0.81 to 2.77) | 1.32 (1.19 to 1.44) |
| Tropical Latin America | Acute myeloid leukemia | DALYs | 2.65 (1.07 to 4.85) | 2.25 (0.92 to 4.07) | 9.52 (4.76 to 15.45) | 3.82 (1.91 to 6.21) | 2.6 (1.99 to 3.84) | 2.02 (1.91 to 2.13) |
| Western Europe | Acute myeloid leukemia | DALYs | 15.46 (6.83 to 27.29) | 3 (1.33 to 5.31) | 32.21 (15.3 to 54.59) | 4.28 (2.03 to 7.23) | 1.08 (0.68 to 1.44) | 1.33 (1.18 to 1.48) |
| Western Sub-Saharan Africa | Acute myeloid leukemia | DALYs | 0.42 (0.15 to 0.88) | 0.36 (0.13 to 0.73) | 2.24 (1.04 to 3.96) | 0.8 (0.37 to 1.41) | 4.28 (2.87 to 7.29) | 2.88 (2.83 to 2.94) |
| Global | Acute lymphoid leukemia | DALYs | 29.53 (12.22 to 56.07) | 0.63 (0.26 to 1.18) | 74.35 (35.02 to 128.29) | 0.9 (0.43 to 1.56) | 1.52 (1.04 to 2.06) | 1.34 (1.28 to 1.4) |
| High SDI | Acute lymphoid leukemia | DALYs | 8.66 (3.89 to 15.51) | 0.92 (0.41 to 1.65) | 14.22 (7.35 to 23.15) | 1.09 (0.57 to 1.76) | 0.64 (0.42 to 1.01) | 0.49 (0.39 to 0.59) |
| High-middle SDI | Acute lymphoid leukemia | DALYs | 11.73 (5.15 to 21.04) | 1.01 (0.44 to 1.8) | 22.43 (10.73 to 38.08) | 1.24 (0.59 to 2.12) | 0.91 (0.58 to 1.25) | 0.7 (0.58 to 0.82) |
| Middle SDI | Acute lymphoid leukemia | DALYs | 6.95 (2.5 to 14.58) | 0.47 (0.17 to 0.97) | 27.64 (12.79 to 48.99) | 1.05 (0.48 to 1.85) | 2.98 (1.67 to 4.64) | 3.09 (3 to 3.18) |
| Low-middle SDI | Acute lymphoid leukemia | DALYs | 1.65 (0.49 to 3.77) | 0.19 (0.06 to 0.42) | 7.66 (3.39 to 14.02) | 0.45 (0.2 to 0.83) | 3.65 (2.21 to 6.7) | 3.37 (3.29 to 3.45) |
| Low SDI | Acute lymphoid leukemia | DALYs | 0.52 (0.14 to 1.3) | 0.15 (0.04 to 0.36) | 2.37 (0.97 to 4.55) | 0.29 (0.12 to 0.54) | 3.51 (2 to 7.02) | 2.46 (2.3 to 2.62) |
| Andean Latin America | Acute lymphoid leukemia | DALYs | 0.36 (0.14 to 0.73) | 1.24 (0.49 to 2.47) | 1.65 (0.78 to 2.89) | 2.61 (1.24 to 4.6) | 3.57 (1.5 to 6.31) | 2.82 (2.68 to 2.96) |
| Australasia | Acute lymphoid leukemia | DALYs | 0.27 (0.13 to 0.48) | 1.23 (0.57 to 2.16) | 0.42 (0.22 to 0.67) | 1.14 (0.59 to 1.82) | 0.54 (0.25 to 1.08) | -0.73 (-0.97 to -0.49) |
| Caribbean | Acute lymphoid leukemia | DALYs | 0.25 (0.12 to 0.45) | 0.79 (0.36 to 1.39) | 0.49 (0.24 to 0.86) | 0.98 (0.48 to 1.71) | 0.94 (0.56 to 1.45) | 0.8 (0.72 to 0.89) |
| Central Asia | Acute lymphoid leukemia | DALYs | 0.79 (0.34 to 1.42) | 1.37 (0.6 to 2.42) | 1.45 (0.71 to 2.47) | 1.53 (0.76 to 2.6) | 0.83 (0.49 to 1.4) | 0.37 (0.22 to 0.51) |
| Central Europe | Acute lymphoid leukemia | DALYs | 1.78 (0.86 to 3.01) | 1.27 (0.6 to 2.15) | 2.02 (1.06 to 3.3) | 1.24 (0.64 to 2.05) | 0.13 (-0.06 to 0.41) | -0.05 (-0.14 to 0.04) |
| Central Latin America | Acute lymphoid leukemia | DALYs | 2.13 (0.88 to 3.85) | 1.65 (0.68 to 2.95) | 8.52 (4.12 to 14.58) | 3.29 (1.59 to 5.65) | 3 (1.74 to 4.38) | 2.63 (2.48 to 2.78) |
| Central Sub-Saharan Africa | Acute lymphoid leukemia | DALYs | 0.03 (0.01 to 0.08) | 0.1 (0.03 to 0.23) | 0.12 (0.04 to 0.26) | 0.13 (0.05 to 0.28) | 2.54 (1.46 to 4.56) | 0.66 (0.21 to 1.12) |
| East Asia | Acute lymphoid leukemia | DALYs | 3.08 (0.54 to 8.79) | 0.26 (0.05 to 0.74) | 16.74 (5.82 to 33.26) | 0.88 (0.31 to 1.76) | 4.43 (1.68 to 12.37) | 5.08 (4.69 to 5.48) |
| Eastern Europe | Acute lymphoid leukemia | DALYs | 5.33 (2.46 to 9.03) | 2.01 (0.92 to 3.43) | 5.99 (3.01 to 9.67) | 2.08 (1.05 to 3.4) | 0.12 (-0.05 to 0.39) | -0.03 (-0.2 to 0.15) |
| Eastern Sub-Saharan Africa | Acute lymphoid leukemia | DALYs | 0.18 (0.04 to 0.46) | 0.15 (0.04 to 0.37) | 0.88 (0.36 to 1.69) | 0.3 (0.13 to 0.58) | 3.93 (1.57 to 9.69) | 2.66 (2.44 to 2.88) |
| High-income Asia Pacific | Acute lymphoid leukemia | DALYs | 0.96 (0.26 to 2.16) | 0.48 (0.13 to 1.1) | 1.28 (0.43 to 2.63) | 0.49 (0.16 to 1.01) | 0.33 (0.04 to 0.81) | -0.22 (-0.32 to -0.12) |
| High-income North America | Acute lymphoid leukemia | DALYs | 3.86 (1.85 to 6.48) | 1.22 (0.58 to 2.04) | 6.7 (3.7 to 10.37) | 1.47 (0.81 to 2.27) | 0.74 (0.55 to 1.08) | 0.66 (0.44 to 0.88) |
| North Africa and Middle East | Acute lymphoid leukemia | DALYs | 2.89 (1.17 to 5.68) | 1.15 (0.48 to 2.26) | 8.31 (4.16 to 13.9) | 1.42 (0.72 to 2.37) | 1.88 (1.11 to 3.12) | 0.63 (0.52 to 0.75) |
| Oceania | Acute lymphoid leukemia | DALYs | 0.03 (0.01 to 0.05) | 0.5 (0.19 to 1) | 0.06 (0.02 to 0.12) | 0.49 (0.2 to 1.01) | 1.23 (0.69 to 2.03) | -0.49 (-0.79 to -0.18) |
| South Asia | Acute lymphoid leukemia | DALYs | 0.92 (0.25 to 2.23) | 0.1 (0.03 to 0.25) | 4.31 (1.91 to 7.98) | 0.24 (0.11 to 0.44) | 3.69 (2.13 to 7.76) | 2.82 (2.62 to 3.02) |
| Southeast Asia | Acute lymphoid leukemia | DALYs | 0.88 (0.24 to 2.35) | 0.23 (0.06 to 0.6) | 3.83 (1.72 to 7.13) | 0.54 (0.24 to 1) | 3.37 (1.8 to 6.8) | 3.13 (3.05 to 3.21) |
| Southern Latin America | Acute lymphoid leukemia | DALYs | 0.48 (0.18 to 0.9) | 1.01 (0.38 to 1.89) | 1.26 (0.59 to 2.11) | 1.68 (0.78 to 2.83) | 1.61 (1.08 to 2.67) | 1.67 (1.42 to 1.92) |
| Southern Sub-Saharan Africa | Acute lymphoid leukemia | DALYs | 0.12 (0.05 to 0.25) | 0.26 (0.11 to 0.52) | 0.28 (0.13 to 0.5) | 0.35 (0.17 to 0.6) | 1.33 (0.19 to 2.64) | 0.88 (0.65 to 1.1) |
| Tropical Latin America | Acute lymphoid leukemia | DALYs | 0.88 (0.35 to 1.69) | 0.68 (0.27 to 1.28) | 3.01 (1.56 to 4.9) | 1.22 (0.63 to 1.98) | 2.4 (1.58 to 4) | 2.23 (2.07 to 2.38) |
| Western Europe | Acute lymphoid leukemia | DALYs | 4.1 (1.77 to 7.44) | 0.88 (0.38 to 1.61) | 6.06 (2.94 to 10.26) | 1.03 (0.5 to 1.74) | 0.48 (0.24 to 0.94) | 0.24 (0.06 to 0.42) |
| Western Sub-Saharan Africa | Acute lymphoid leukemia | DALYs | 0.2 (0.07 to 0.43) | 0.15 (0.05 to 0.32) | 0.99 (0.45 to 1.83) | 0.3 (0.14 to 0.54) | 3.9 (2.32 to 7.13) | 2.25 (2.16 to 2.35) |
| Global | Chronic myeloid leukemia | DALYs | 38.32 (16.58 to 69.27) | 0.88 (0.38 to 1.58) | 54.5 (27.49 to 90.64) | 0.66 (0.33 to 1.1) | 0.42 (0.23 to 0.74) | -1.52 (-1.7 to -1.34) |
| High SDI | Chronic myeloid leukemia | DALYs | 15.22 (7 to 26.47) | 1.55 (0.71 to 2.7) | 10.38 (5.39 to 16.71) | 0.66 (0.35 to 1.06) | -0.32 (-0.41 to -0.18) | -3.8 (-4.16 to -3.43) |
| High-middle SDI | Chronic myeloid leukemia | DALYs | 11.59 (5.32 to 19.96) | 1.03 (0.47 to 1.78) | 11.77 (5.99 to 19.08) | 0.61 (0.31 to 0.98) | 0.02 (-0.1 to 0.21) | -2.46 (-2.73 to -2.19) |
| Middle SDI | Chronic myeloid leukemia | DALYs | 6.27 (2.62 to 11.73) | 0.47 (0.2 to 0.88) | 15.37 (8.01 to 25.48) | 0.58 (0.3 to 0.96) | 1.45 (0.96 to 2.34) | 0.47 (0.38 to 0.57) |
| Low-middle SDI | Chronic myeloid leukemia | DALYs | 3.12 (0.96 to 7.25) | 0.4 (0.12 to 0.91) | 10.85 (4.92 to 20.07) | 0.68 (0.31 to 1.25) | 2.47 (1.5 to 4.93) | 1.72 (1.64 to 1.81) |
| Low SDI | Chronic myeloid leukemia | DALYs | 2.08 (0.54 to 5.23) | 0.64 (0.17 to 1.59) | 6.08 (2.64 to 11.38) | 0.84 (0.37 to 1.57) | 1.93 (0.99 to 4.78) | 0.9 (0.73 to 1.07) |
| Andean Latin America | Chronic myeloid leukemia | DALYs | 0.16 (0.07 to 0.29) | 0.61 (0.26 to 1.1) | 0.55 (0.27 to 0.94) | 0.91 (0.45 to 1.56) | 2.5 (1.53 to 4.23) | 1.79 (1.57 to 2.01) |
| Australasia | Chronic myeloid leukemia | DALYs | 0.44 (0.2 to 0.74) | 1.91 (0.9 to 3.24) | 0.32 (0.16 to 0.52) | 0.73 (0.38 to 1.18) | -0.27 (-0.39 to -0.08) | -3.99 (-4.31 to -3.67) |
| Caribbean | Chronic myeloid leukemia | DALYs | 0.4 (0.18 to 0.68) | 1.38 (0.62 to 2.38) | 0.57 (0.28 to 0.96) | 1.11 (0.54 to 1.89) | 0.43 (0.17 to 0.81) | -0.76 (-0.88 to -0.64) |
| Central Asia | Chronic myeloid leukemia | DALYs | 0.5 (0.23 to 0.9) | 0.93 (0.43 to 1.67) | 0.71 (0.35 to 1.16) | 0.79 (0.39 to 1.29) | 0.4 (0.12 to 0.83) | -0.84 (-0.99 to -0.7) |
| Central Europe | Chronic myeloid leukemia | DALYs | 2.28 (1.13 to 3.79) | 1.57 (0.78 to 2.61) | 1.48 (0.77 to 2.49) | 0.78 (0.41 to 1.31) | -0.35 (-0.48 to -0.05) | -2.96 (-3.28 to -2.65) |
| Central Latin America | Chronic myeloid leukemia | DALYs | 1.11 (0.5 to 1.96) | 1.02 (0.46 to 1.78) | 2.17 (1.07 to 3.7) | 0.87 (0.43 to 1.47) | 0.96 (0.58 to 1.56) | -1.15 (-1.42 to -0.87) |
| Central Sub-Saharan Africa | Chronic myeloid leukemia | DALYs | 0.13 (0.04 to 0.29) | 0.41 (0.14 to 0.91) | 0.35 (0.13 to 0.7) | 0.45 (0.17 to 0.89) | 1.75 (0.82 to 3.62) | 0.14 (-0.34 to 0.62) |
| East Asia | Chronic myeloid leukemia | DALYs | 0.94 (0.2 to 2.43) | 0.09 (0.02 to 0.22) | 1.85 (0.68 to 3.55) | 0.09 (0.03 to 0.18) | 0.96 (0.26 to 3.22) | -0.02 (-0.14 to 0.1) |
| Eastern Europe | Chronic myeloid leukemia | DALYs | 3.85 (1.84 to 6.67) | 1.4 (0.67 to 2.43) | 4.21 (2.14 to 6.74) | 1.34 (0.68 to 2.15) | 0.09 (-0.11 to 0.35) | -0.72 (-1.15 to -0.28) |
| Eastern Sub-Saharan Africa | Chronic myeloid leukemia | DALYs | 0.96 (0.21 to 2.65) | 0.91 (0.21 to 2.46) | 2.58 (1.11 to 5.02) | 1.07 (0.45 to 2.1) | 1.67 (0.41 to 6.18) | 0.41 (0.12 to 0.7) |
| High-income Asia Pacific | Chronic myeloid leukemia | DALYs | 1.19 (0.34 to 2.61) | 0.59 (0.17 to 1.31) | 0.56 (0.19 to 1.14) | 0.17 (0.06 to 0.34) | -0.53 (-0.62 to -0.37) | -5.06 (-5.4 to -4.72) |
| High-income North America | Chronic myeloid leukemia | DALYs | 6.89 (3.38 to 11.24) | 2.13 (1.05 to 3.46) | 4.19 (2.23 to 6.56) | 0.77 (0.42 to 1.21) | -0.39 (-0.46 to -0.29) | -4.37 (-4.77 to -3.96) |
| North Africa and Middle East | Chronic myeloid leukemia | DALYs | 4.45 (1.96 to 8.12) | 2.04 (0.92 to 3.63) | 8.97 (4.41 to 15.2) | 1.65 (0.82 to 2.79) | 1.02 (0.59 to 1.72) | -0.94 (-1.06 to -0.83) |
| Oceania | Chronic myeloid leukemia | DALYs | 0.05 (0.02 to 0.1) | 1.16 (0.46 to 2.28) | 0.09 (0.04 to 0.19) | 0.94 (0.37 to 1.87) | 0.89 (0.4 to 1.52) | -1.2 (-1.51 to -0.89) |
| South Asia | Chronic myeloid leukemia | DALYs | 3.5 (1 to 8.27) | 0.45 (0.13 to 1.05) | 13.4 (6.04 to 24.58) | 0.81 (0.37 to 1.47) | 2.83 (1.61 to 6.04) | 1.84 (1.71 to 1.97) |
| Southeast Asia | Chronic myeloid leukemia | DALYs | 0.81 (0.25 to 1.83) | 0.23 (0.07 to 0.53) | 3 (1.38 to 5.5) | 0.43 (0.2 to 0.79) | 2.72 (1.53 to 5.51) | 2.25 (2.16 to 2.34) |
| Southern Latin America | Chronic myeloid leukemia | DALYs | 0.65 (0.27 to 1.18) | 1.38 (0.57 to 2.51) | 0.61 (0.29 to 1.02) | 0.77 (0.37 to 1.3) | -0.06 (-0.22 to 0.25) | -2.79 (-3.29 to -2.29) |
| Southern Sub-Saharan Africa | Chronic myeloid leukemia | DALYs | 0.07 (0.03 to 0.13) | 0.18 (0.08 to 0.32) | 0.11 (0.06 to 0.19) | 0.15 (0.08 to 0.25) | 0.6 (0.19 to 1.21) | -0.51 (-0.8 to -0.22) |
| Tropical Latin America | Chronic myeloid leukemia | DALYs | 1.32 (0.55 to 2.39) | 1.16 (0.49 to 2.09) | 1.63 (0.84 to 2.66) | 0.66 (0.34 to 1.08) | 0.23 (0.02 to 0.64) | -2.48 (-2.94 to -2.01) |
| Western Europe | Chronic myeloid leukemia | DALYs | 8.07 (3.64 to 14.21) | 1.57 (0.71 to 2.77) | 4.82 (2.35 to 8.22) | 0.6 (0.29 to 1.02) | -0.4 (-0.48 to -0.28) | -4.29 (-4.65 to -3.93) |
| Western Sub-Saharan Africa | Chronic myeloid leukemia | DALYs | 0.56 (0.2 to 1.15) | 0.48 (0.17 to 0.98) | 2.32 (1.02 to 4.24) | 0.81 (0.36 to 1.49) | 3.11 (1.91 to 5.44) | 1.9 (1.83 to 1.96) |
| Global | Chronic lymphoid leukemia | DALYs | 31.17 (13.93 to 54.87) | 0.8 (0.36 to 1.41) | 76.95 (37.84 to 129.81) | 0.94 (0.46 to 1.59) | 1.47 (1.22 to 1.87) | 0.42 (0.3 to 0.54) |
| High SDI | Chronic lymphoid leukemia | DALYs | 15.13 (7.1 to 25.79) | 1.44 (0.68 to 2.46) | 25.88 (13.34 to 42.43) | 1.36 (0.7 to 2.23) | 0.71 (0.51 to 1.08) | -0.51 (-0.74 to -0.27) |
| High-middle SDI | Chronic lymphoid leukemia | DALYs | 10.62 (5.03 to 18.1) | 0.98 (0.46 to 1.67) | 24.22 (12.16 to 39.46) | 1.19 (0.6 to 1.94) | 1.28 (1 to 1.73) | 0.64 (0.46 to 0.82) |
| Middle SDI | Chronic lymphoid leukemia | DALYs | 3.3 (1.28 to 6.33) | 0.29 (0.12 to 0.56) | 14.7 (6.83 to 25.2) | 0.57 (0.27 to 0.99) | 3.45 (2.65 to 4.7) | 2.44 (2.36 to 2.52) |
| Low-middle SDI | Chronic lymphoid leukemia | DALYs | 1.4 (0.4 to 3.13) | 0.22 (0.06 to 0.5) | 8.45 (3.44 to 15.68) | 0.6 (0.24 to 1.11) | 5.05 (3.46 to 8.73) | 3.55 (3.45 to 3.64) |
| Low SDI | Chronic lymphoid leukemia | DALYs | 0.71 (0.19 to 1.64) | 0.29 (0.08 to 0.68) | 3.67 (1.37 to 7.16) | 0.69 (0.26 to 1.35) | 4.19 (2.86 to 7.51) | 3.22 (3.12 to 3.33) |
| Andean Latin America | Chronic lymphoid leukemia | DALYs | 0.05 (0.02 to 0.1) | 0.23 (0.1 to 0.42) | 0.31 (0.15 to 0.54) | 0.54 (0.26 to 0.93) | 4.92 (3.03 to 8.06) | 3.32 (3.13 to 3.52) |
| Australasia | Chronic lymphoid leukemia | DALYs | 0.39 (0.18 to 0.66) | 1.63 (0.78 to 2.8) | 0.86 (0.43 to 1.44) | 1.71 (0.86 to 2.86) | 1.24 (0.84 to 1.79) | -0.16 (-0.36 to 0.05) |
| Caribbean | Chronic lymphoid leukemia | DALYs | 0.16 (0.07 to 0.27) | 0.6 (0.27 to 1.02) | 0.4 (0.2 to 0.67) | 0.77 (0.38 to 1.3) | 1.54 (1.04 to 2.22) | 1.07 (0.92 to 1.22) |
| Central Asia | Chronic lymphoid leukemia | DALYs | 0.38 (0.17 to 0.66) | 0.75 (0.34 to 1.3) | 0.78 (0.38 to 1.3) | 0.98 (0.49 to 1.66) | 1.07 (0.59 to 1.89) | 0.93 (0.83 to 1.03) |
| Central Europe | Chronic lymphoid leukemia | DALYs | 2.2 (1.1 to 3.61) | 1.47 (0.73 to 2.41) | 6.18 (3.28 to 10.14) | 2.89 (1.54 to 4.74) | 1.81 (1.31 to 2.72) | 2.74 (2.32 to 3.16) |
| Central Latin America | Chronic lymphoid leukemia | DALYs | 0.32 (0.14 to 0.57) | 0.36 (0.16 to 0.64) | 1.2 (0.59 to 2.04) | 0.5 (0.25 to 0.86) | 2.69 (1.93 to 3.88) | 1.02 (0.9 to 1.13) |
| Central Sub-Saharan Africa | Chronic lymphoid leukemia | DALYs | 0.06 (0.02 to 0.14) | 0.26 (0.08 to 0.57) | 0.37 (0.13 to 0.73) | 0.65 (0.23 to 1.31) | 4.85 (3 to 8.18) | 3.33 (3.2 to 3.45) |
| East Asia | Chronic lymphoid leukemia | DALYs | 1.06 (0.2 to 2.75) | 0.1 (0.02 to 0.25) | 7.06 (2.59 to 13.58) | 0.35 (0.13 to 0.68) | 5.68 (3.19 to 13.74) | 5.12 (4.84 to 5.39) |
| Eastern Europe | Chronic lymphoid leukemia | DALYs | 5.07 (2.42 to 8.67) | 1.77 (0.83 to 3.03) | 8.52 (4.44 to 13.68) | 2.47 (1.28 to 3.97) | 0.68 (0.39 to 1.12) | 1.1 (0.9 to 1.31) |
| Eastern Sub-Saharan Africa | Chronic lymphoid leukemia | DALYs | 0.26 (0.07 to 0.63) | 0.35 (0.09 to 0.85) | 1.48 (0.57 to 2.8) | 0.93 (0.35 to 1.77) | 4.67 (3.02 to 9.26) | 3.8 (3.57 to 4.03) |
| High-income Asia Pacific | Chronic lymphoid leukemia | DALYs | 0.15 (0.04 to 0.33) | 0.08 (0.02 to 0.17) | 0.29 (0.1 to 0.6) | 0.07 (0.02 to 0.15) | 0.91 (0.63 to 1.44) | -0.19 (-0.3 to -0.07) |
| High-income North America | Chronic lymphoid leukemia | DALYs | 7.49 (3.61 to 12.6) | 2.14 (1.04 to 3.58) | 13.09 (6.91 to 21) | 2.05 (1.09 to 3.3) | 0.75 (0.52 to 1.18) | -0.58 (-0.86 to -0.3) |
| North Africa and Middle East | Chronic lymphoid leukemia | DALYs | 1.23 (0.57 to 2.13) | 0.65 (0.3 to 1.13) | 4.63 (2.42 to 7.32) | 1.03 (0.53 to 1.64) | 2.75 (1.95 to 4.06) | 1.47 (1.39 to 1.56) |
| Oceania | Chronic lymphoid leukemia | DALYs | 0 (0 to 0.01) | 0.09 (0.04 to 0.18) | 0.01 (0 to 0.02) | 0.1 (0.04 to 0.19) | 1.57 (0.95 to 2.47) | -0.14 (-0.34 to 0.06) |
| South Asia | Chronic lymphoid leukemia | DALYs | 1.39 (0.37 to 3.29) | 0.24 (0.06 to 0.57) | 9.78 (3.95 to 18.53) | 0.68 (0.27 to 1.29) | 6.05 (3.88 to 11.9) | 3.62 (3.47 to 3.78) |
| Southeast Asia | Chronic lymphoid leukemia | DALYs | 0.22 (0.07 to 0.51) | 0.08 (0.02 to 0.19) | 1.54 (0.69 to 2.88) | 0.25 (0.11 to 0.47) | 5.99 (3.93 to 11.08) | 4.08 (3.97 to 4.2) |
| Southern Latin America | Chronic lymphoid leukemia | DALYs | 0.27 (0.11 to 0.5) | 0.58 (0.24 to 1.07) | 0.61 (0.28 to 1.05) | 0.72 (0.34 to 1.24) | 1.25 (0.81 to 2.04) | 0.3 (-0.12 to 0.72) |
| Southern Sub-Saharan Africa | Chronic lymphoid leukemia | DALYs | 1.04 (0.45 to 1.82) | 3.55 (1.51 to 6.11) | 2.56 (1.17 to 4.22) | 4.43 (2.03 to 7.35) | 1.46 (1.13 to 1.95) | 1.16 (0.93 to 1.39) |
| Tropical Latin America | Chronic lymphoid leukemia | DALYs | 0.4 (0.17 to 0.71) | 0.43 (0.19 to 0.78) | 1.67 (0.85 to 2.73) | 0.69 (0.35 to 1.14) | 3.21 (2.54 to 4.4) | 1.78 (1.63 to 1.94) |
| Western Europe | Chronic lymphoid leukemia | DALYs | 8.61 (3.93 to 14.91) | 1.49 (0.68 to 2.58) | 13.83 (6.73 to 23.55) | 1.48 (0.72 to 2.51) | 0.61 (0.42 to 0.97) | -0.24 (-0.52 to 0.03) |
| Western Sub-Saharan Africa | Chronic lymphoid leukemia | DALYs | 0.41 (0.14 to 0.83) | 0.46 (0.16 to 0.93) | 1.79 (0.75 to 3.28) | 0.95 (0.4 to 1.73) | 3.41 (2.3 to 5.66) | 2.67 (2.62 to 2.72) |
| Global | Other leukemia | DALYs | 103.73 (39.51 to 204.34) | 2.38 (0.92 to 4.62) | 191.82 (91.8 to 335.4) | 2.33 (1.12 to 4.08) | 0.85 (0.51 to 1.45) | -0.23 (-0.32 to -0.14) |
| High SDI | Other leukemia | DALYs | 25.62 (11.35 to 45.14) | 2.55 (1.13 to 4.51) | 36.96 (18.72 to 61.04) | 2.17 (1.11 to 3.59) | 0.44 (0.28 to 0.71) | -0.67 (-0.71 to -0.63) |
| High-middle SDI | Other leukemia | DALYs | 35.09 (14.13 to 67.4) | 3.11 (1.26 to 5.93) | 51.14 (23.84 to 89.54) | 2.67 (1.24 to 4.7) | 0.46 (0.21 to 0.87) | -0.84 (-0.99 to -0.7) |
| Middle SDI | Other leukemia | DALYs | 32.99 (10.56 to 72.05) | 2.46 (0.8 to 5.26) | 73.56 (33.87 to 130.22) | 2.8 (1.29 to 4.96) | 1.23 (0.63 to 2.64) | 0.33 (0.2 to 0.46) |
| Low-middle SDI | Other leukemia | DALYs | 7.44 (2.08 to 17.48) | 0.96 (0.28 to 2.21) | 21.85 (9.81 to 41.16) | 1.41 (0.63 to 2.64) | 1.94 (1.1 to 4.19) | 1.3 (1.22 to 1.37) |
| Low SDI | Other leukemia | DALYs | 2.52 (0.72 to 5.76) | 0.85 (0.25 to 1.9) | 8.15 (3.36 to 15.72) | 1.22 (0.51 to 2.31) | 2.23 (1.5 to 4.11) | 1.4 (1.26 to 1.54) |
| Andean Latin America | Other leukemia | DALYs | 1.24 (0.5 to 2.32) | 4.67 (1.92 to 8.65) | 3.23 (1.53 to 5.85) | 5.36 (2.55 to 9.67) | 1.61 (0.86 to 2.87) | 0.35 (0.12 to 0.58) |
| Australasia | Other leukemia | DALYs | 0.23 (0.11 to 0.39) | 0.98 (0.46 to 1.66) | 0.63 (0.32 to 1.03) | 1.33 (0.69 to 2.17) | 1.76 (1.2 to 2.46) | 1.64 (1.31 to 1.96) |
| Caribbean | Other leukemia | DALYs | 0.85 (0.36 to 1.51) | 2.94 (1.23 to 5.18) | 1.91 (0.93 to 3.31) | 3.77 (1.82 to 6.53) | 1.25 (0.78 to 1.99) | 0.98 (0.87 to 1.08) |
| Central Asia | Other leukemia | DALYs | 2.06 (0.93 to 3.69) | 3.71 (1.68 to 6.56) | 2.79 (1.38 to 4.7) | 3.22 (1.61 to 5.44) | 0.36 (0.12 to 0.78) | -0.81 (-0.96 to -0.65) |
| Central Europe | Other leukemia | DALYs | 5.76 (2.86 to 9.54) | 3.94 (1.94 to 6.53) | 5.62 (2.97 to 9.22) | 2.85 (1.51 to 4.68) | -0.02 (-0.19 to 0.28) | -1.46 (-1.61 to -1.32) |
| Central Latin America | Other leukemia | DALYs | 3.68 (1.55 to 6.53) | 3.26 (1.38 to 5.77) | 6.97 (3.35 to 12.12) | 2.79 (1.34 to 4.84) | 0.9 (0.5 to 1.54) | -0.69 (-0.8 to -0.59) |
| Central Sub-Saharan Africa | Other leukemia | DALYs | 0.41 (0.13 to 0.86) | 1.39 (0.46 to 2.88) | 0.87 (0.32 to 1.77) | 1.18 (0.43 to 2.4) | 1.15 (0.54 to 2.16) | -1.07 (-1.4 to -0.74) |
| East Asia | Other leukemia | DALYs | 26.47 (5.07 to 68.01) | 2.32 (0.45 to 5.93) | 49.71 (17.65 to 98.72) | 2.53 (0.9 to 5.03) | 0.88 (0.25 to 3.13) | 0.05 (-0.21 to 0.31) |
| Eastern Europe | Other leukemia | DALYs | 7.46 (3.59 to 12.76) | 2.71 (1.29 to 4.66) | 7.06 (3.57 to 11.27) | 2.22 (1.13 to 3.57) | -0.05 (-0.19 to 0.14) | -1.5 (-1.84 to -1.15) |
| Eastern Sub-Saharan Africa | Other leukemia | DALYs | 0.6 (0.17 to 1.43) | 0.59 (0.16 to 1.39) | 2 (0.84 to 3.75) | 0.88 (0.37 to 1.63) | 2.36 (1.21 to 5.42) | 1.52 (1.41 to 1.62) |
| High-income Asia Pacific | Other leukemia | DALYs | 2.63 (0.75 to 5.7) | 1.3 (0.37 to 2.83) | 2.56 (0.85 to 5.32) | 0.68 (0.23 to 1.39) | -0.03 (-0.17 to 0.28) | -2.79 (-2.98 to -2.6) |
| High-income North America | Other leukemia | DALYs | 12.73 (6.13 to 21.19) | 3.78 (1.83 to 6.26) | 19.16 (10.16 to 29.95) | 3.31 (1.78 to 5.17) | 0.51 (0.34 to 0.78) | -0.58 (-0.69 to -0.46) |
| North Africa and Middle East | Other leukemia | DALYs | 11.17 (4.78 to 20.01) | 5.38 (2.37 to 9.48) | 27.82 (14.2 to 47.8) | 5.63 (2.89 to 9.6) | 1.49 (0.97 to 3.09) | 0.15 (0.08 to 0.23) |
| Oceania | Other leukemia | DALYs | 0.13 (0.05 to 0.27) | 3.22 (1.14 to 6.43) | 0.33 (0.12 to 0.69) | 3.44 (1.29 to 6.95) | 1.5 (0.95 to 2.32) | -0.02 (-0.2 to 0.16) |
| South Asia | Other leukemia | DALYs | 2 (0.51 to 5.05) | 0.28 (0.07 to 0.69) | 7.36 (3.28 to 14.02) | 0.46 (0.21 to 0.88) | 2.68 (1.46 to 6.85) | 1.58 (1.41 to 1.75) |
| Southeast Asia | Other leukemia | DALYs | 6.56 (1.83 to 15.6) | 1.91 (0.53 to 4.49) | 22.14 (9.95 to 41.17) | 3.22 (1.45 to 6.04) | 2.37 (1.32 to 5.33) | 1.83 (1.65 to 2.01) |
| Southern Latin America | Other leukemia | DALYs | 1.49 (0.6 to 2.74) | 3.18 (1.28 to 5.83) | 2.94 (1.39 to 4.92) | 3.69 (1.77 to 6.17) | 0.97 (0.65 to 1.62) | 0.32 (0.18 to 0.47) |
| Southern Sub-Saharan Africa | Other leukemia | DALYs | 1.15 (0.54 to 1.97) | 2.99 (1.43 to 5.08) | 2.03 (1.1 to 3.35) | 2.99 (1.6 to 4.89) | 0.77 (0.37 to 1.45) | 0.01 (-0.33 to 0.34) |
| Tropical Latin America | Other leukemia | DALYs | 2.53 (1.04 to 4.61) | 2.29 (0.94 to 4.14) | 6.14 (3.15 to 10.11) | 2.51 (1.28 to 4.12) | 1.43 (1.02 to 2.25) | 0.51 (0.42 to 0.59) |
| Western Europe | Other leukemia | DALYs | 13.48 (6.11 to 23.59) | 2.51 (1.13 to 4.41) | 16.11 (7.79 to 27.54) | 1.9 (0.93 to 3.22) | 0.2 (0.07 to 0.4) | -0.97 (-1.07 to -0.86) |
| Western Sub-Saharan Africa | Other leukemia | DALYs | 1.13 (0.4 to 2.39) | 1.09 (0.38 to 2.28) | 4.43 (2.06 to 7.82) | 1.84 (0.85 to 3.25) | 2.91 (1.91 to 5.26) | 1.91 (1.84 to 1.98) |
