# Supplementary figures and images for: Global burden and trends of leukemia attributable to high body mass index risk in adults over the past 30 years
Source: Front Oncol. 2024 Jun 19;14:1404135. doi: 10.3389/fonc.2024.1404135 (PMC11219942; doi:10.3389/fonc.2024.1404135)

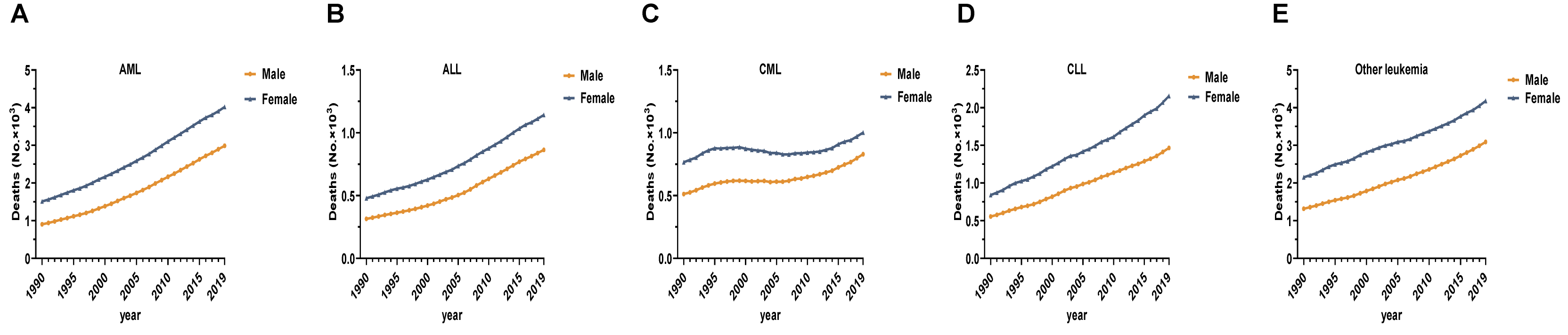

Supplement: Supplementary Figure S1 — The number of deaths cases in different subtypes of leukemia attributable to high BMI risk by different sex. A AML B ALL C CML D CLL E other leukemia. AML acute myeloid leukemia, ALL acute lymphoblastic leukemia, CML chronic myeloid leukemia, CLL chronic lymphocytic leukemia. [file Image_1.tif]

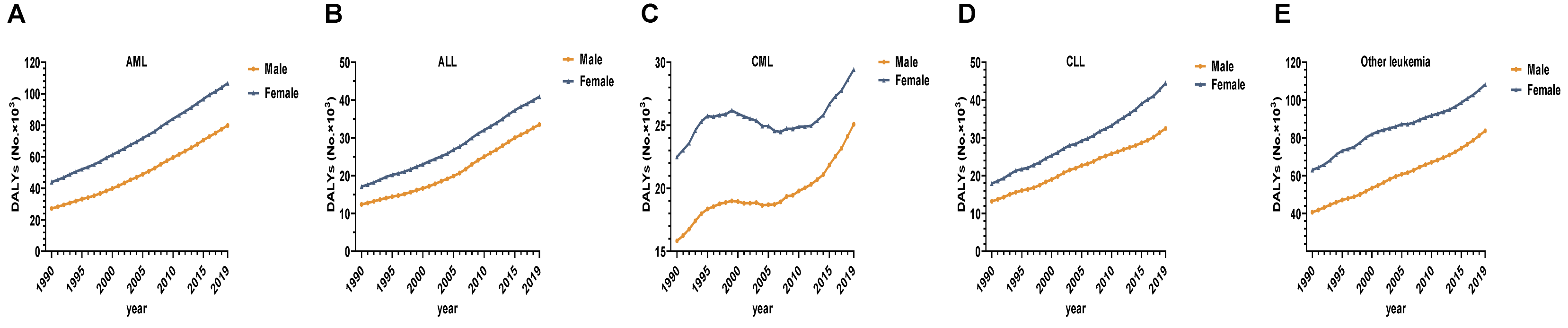

Supplement: Supplementary Figure S2 — The number of DALYs cases in different subtypes of leukemia attributable to high BMI risk by different sex. A AML B ALL C CML D CLL E other leukemia. AML acute myeloid leukemia, ALL acute lymphoblastic leukemia, CML chronic myeloid leukemia, CLL chronic lymphocytic leukemia. [file Image_2.tif]

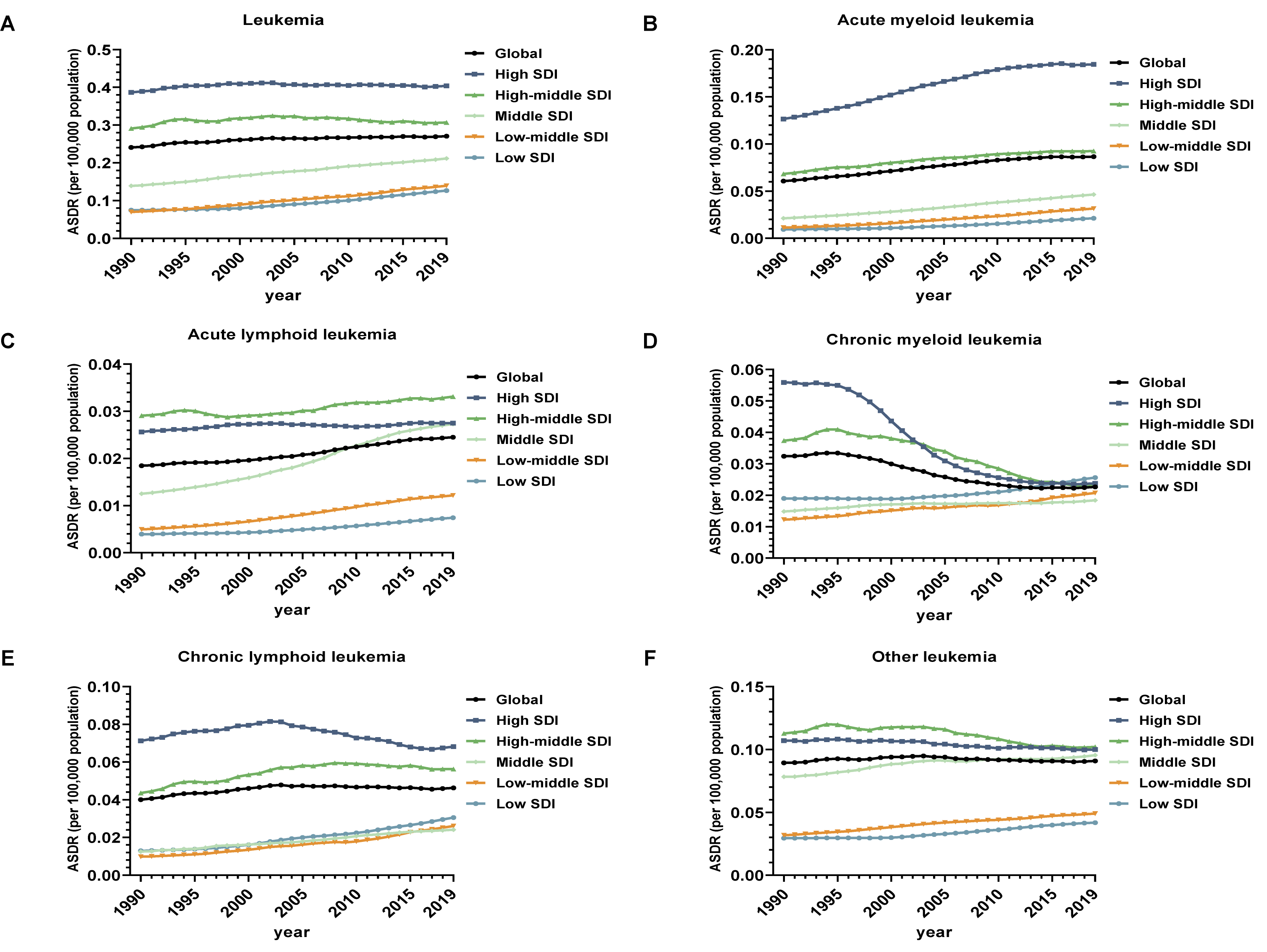

Supplement: Supplementary Figure S3 — The ASDRs of different subtypes of leukemia attributable to high BMI risk in global and five SDI regions from 1990 to 2019. A all leukemia B AML C ALL D CML E CLL F other leukemia. AML acute myeloid leukemia, ALL acute lymphoblastic leukemia, CML chronic myeloid leukemia, CLL chronic lymphocytic leukemia. [file Image_3.tif]

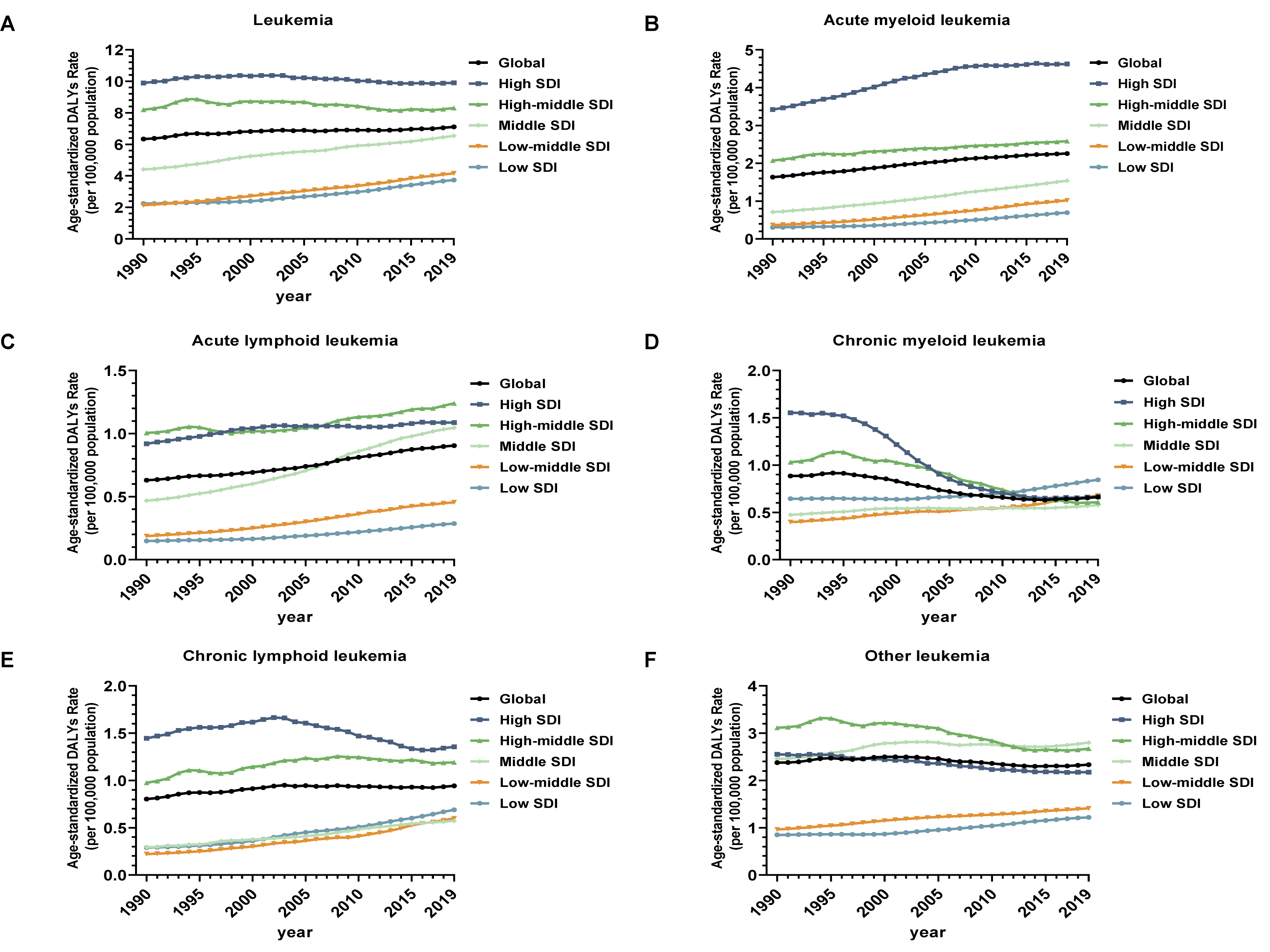

Supplement: Supplementary Figure S4 — The age-standardized DALYs rates of different subtypes of leukemia attributable to high BMI risk in global and five SDI regions from 1990 to 2019. A all leukemia B AML C ALL D CML E CLL F other leukemia. AML acute myeloid leukemia, ALL acute lymphoblastic leukemia, CML chronic myeloid leukemia, CLL chronic lymphocytic leukemia. [file Image_4.tif]

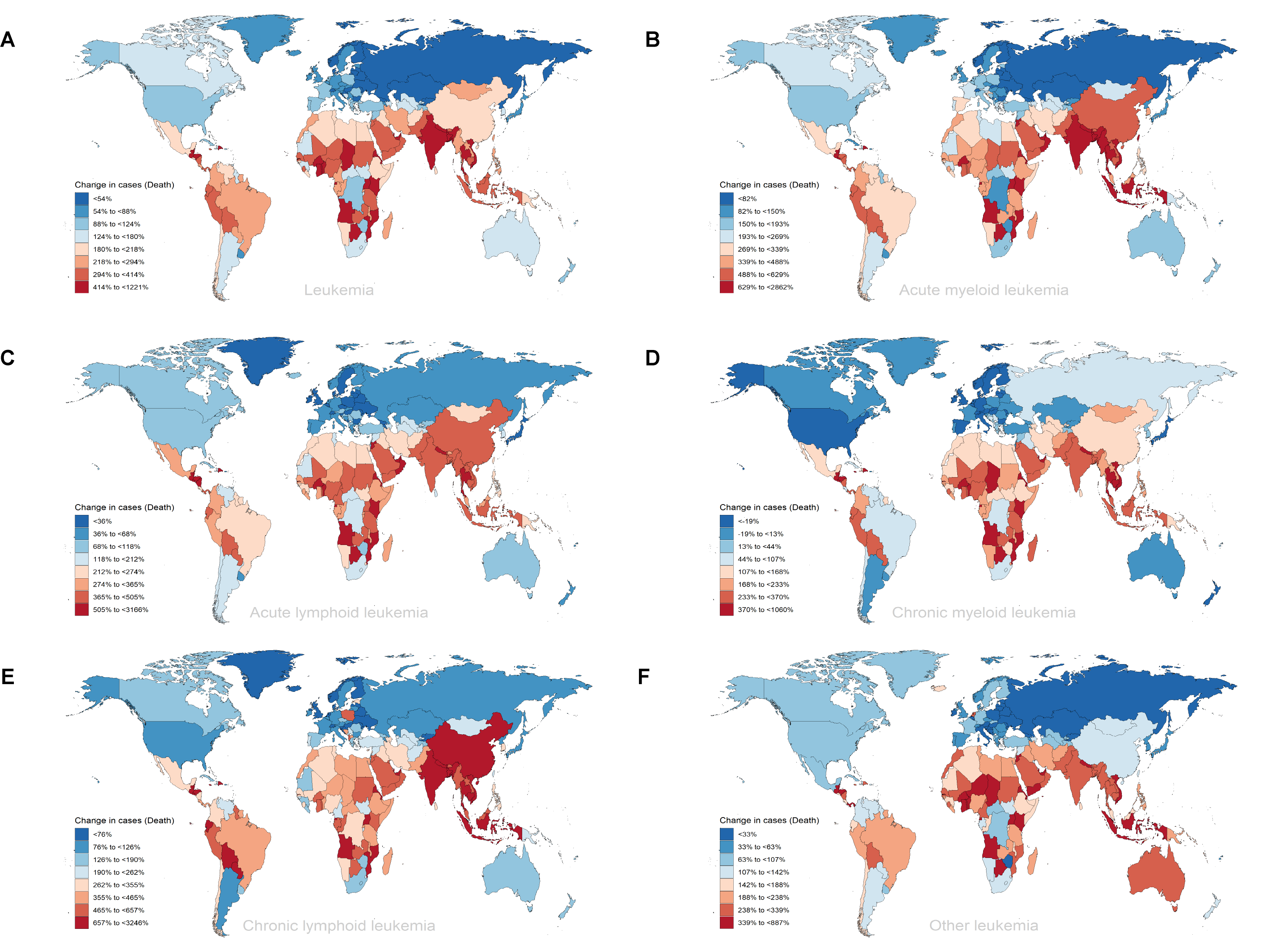

Supplement: Supplementary Figure S5 — The percentage change of the number of deaths from different subtypes of leukemia attributable to high BMI risk in 1990 and 2019. A leukemia B AML C ALL D CML E CLL F other leukemia. AML acute myeloid leukemia, ALL acute lymphoblastic leukemia, CML chronic myeloid leukemia, CLL chronic lymphocytic leukemia. [file Image_5.tif]

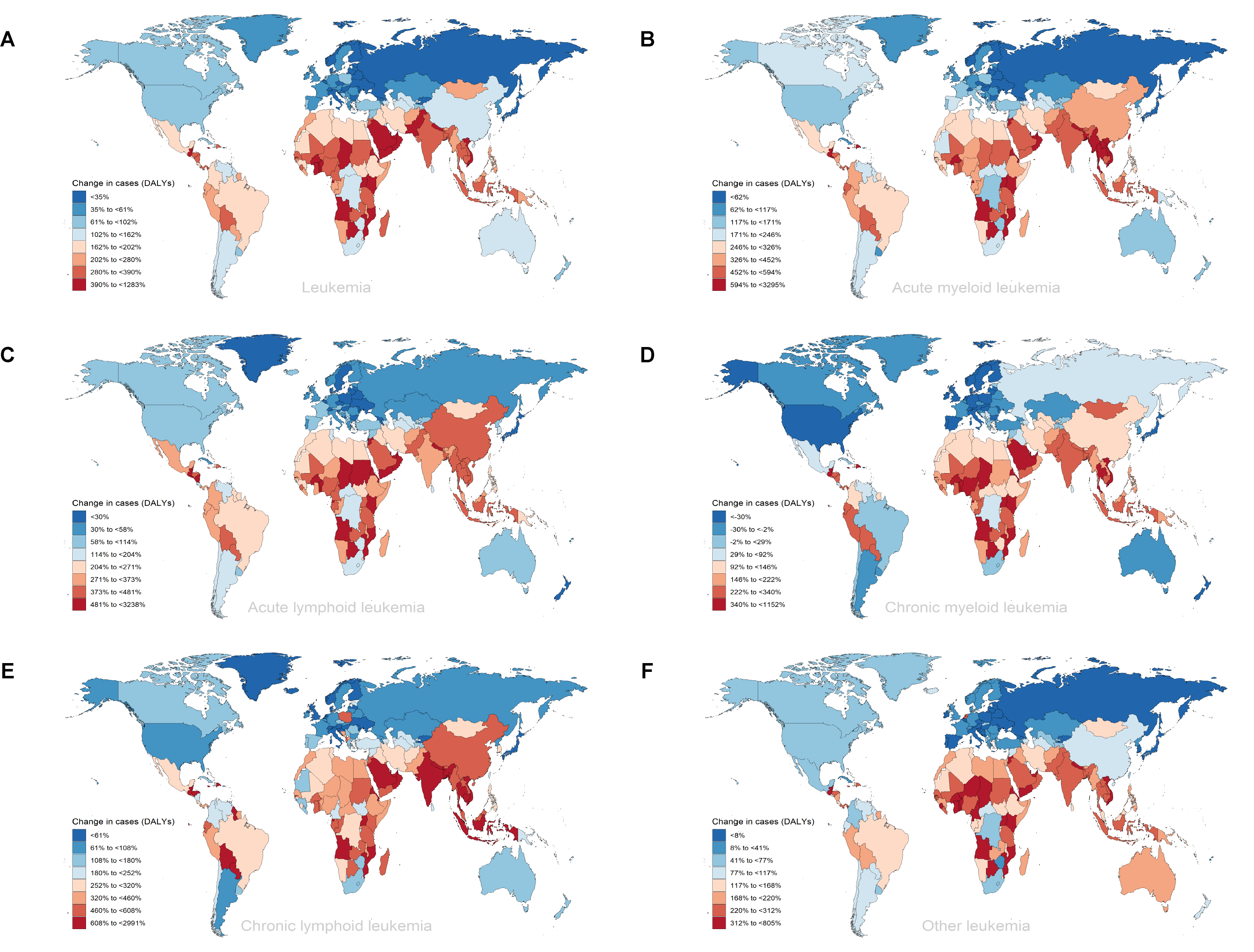

Supplement: Supplementary Figure S6 — The percentage change of the number of DALYs from different subtypes of leukemia attributable to high BMI risk in 1990 and 2019. A leukemia B AML C ALL D CML E CLL F other leukemia. AML acute myeloid leukemia, ALL acute lymphoblastic leukemia, CML chronic myeloid leukemia, CLL chronic lymphocytic leukemia. [file Image_6.tif]

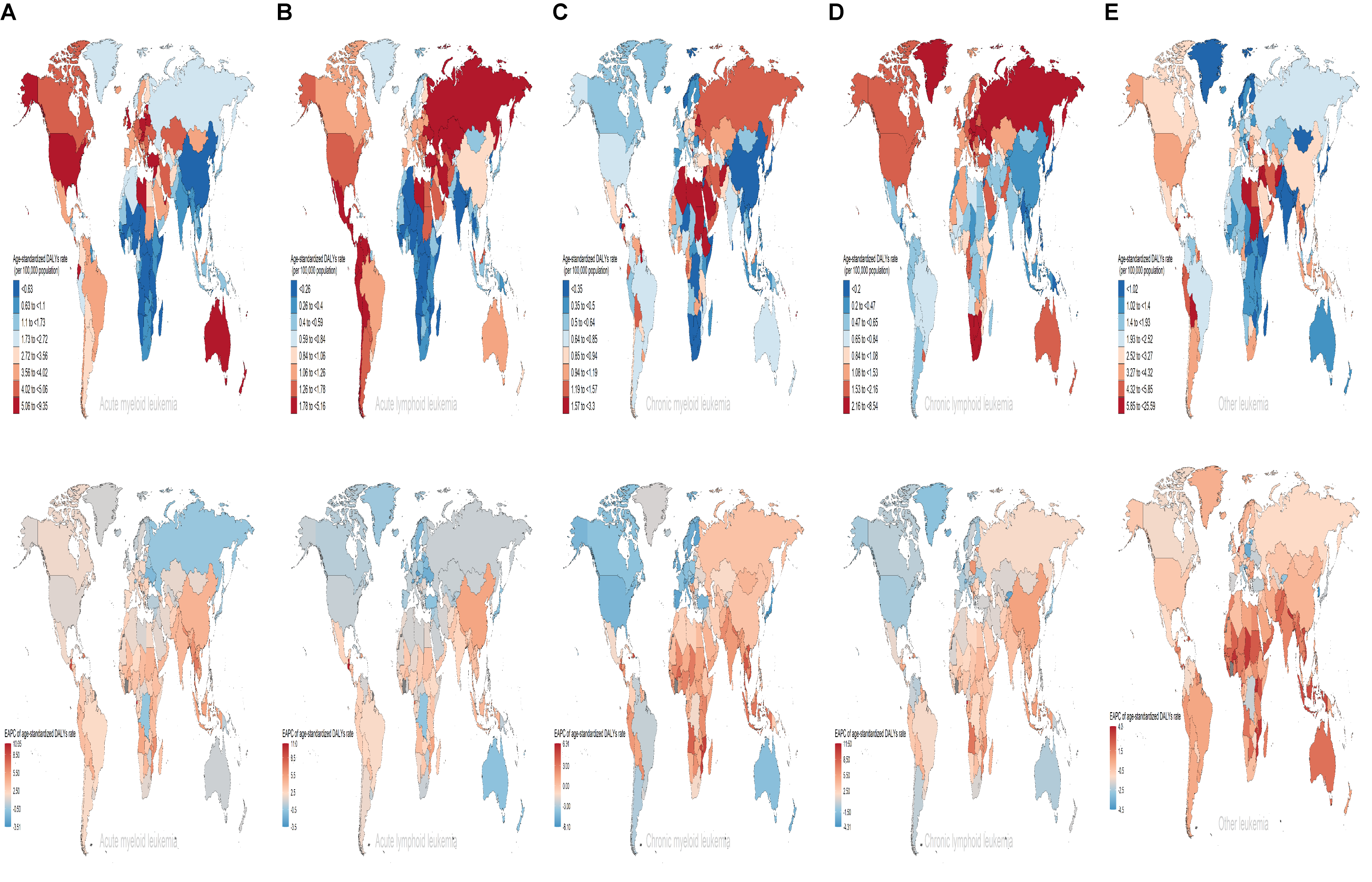

Supplement: Supplementary Figure S7 — The age-standardized DALYs rates of different subtypes of leukemia attributable to high BMI risk in 2019 and the corresponding EAPC from 1990 to 2019. A AML B ALL C CML D CLL E other leukemia. AML acute myeloid leukemia, ALL acute lymphoblastic leukemia, CML chronic myeloid leukemia, CLL chronic lymphocytic leukemia. [file Image_7.tif]

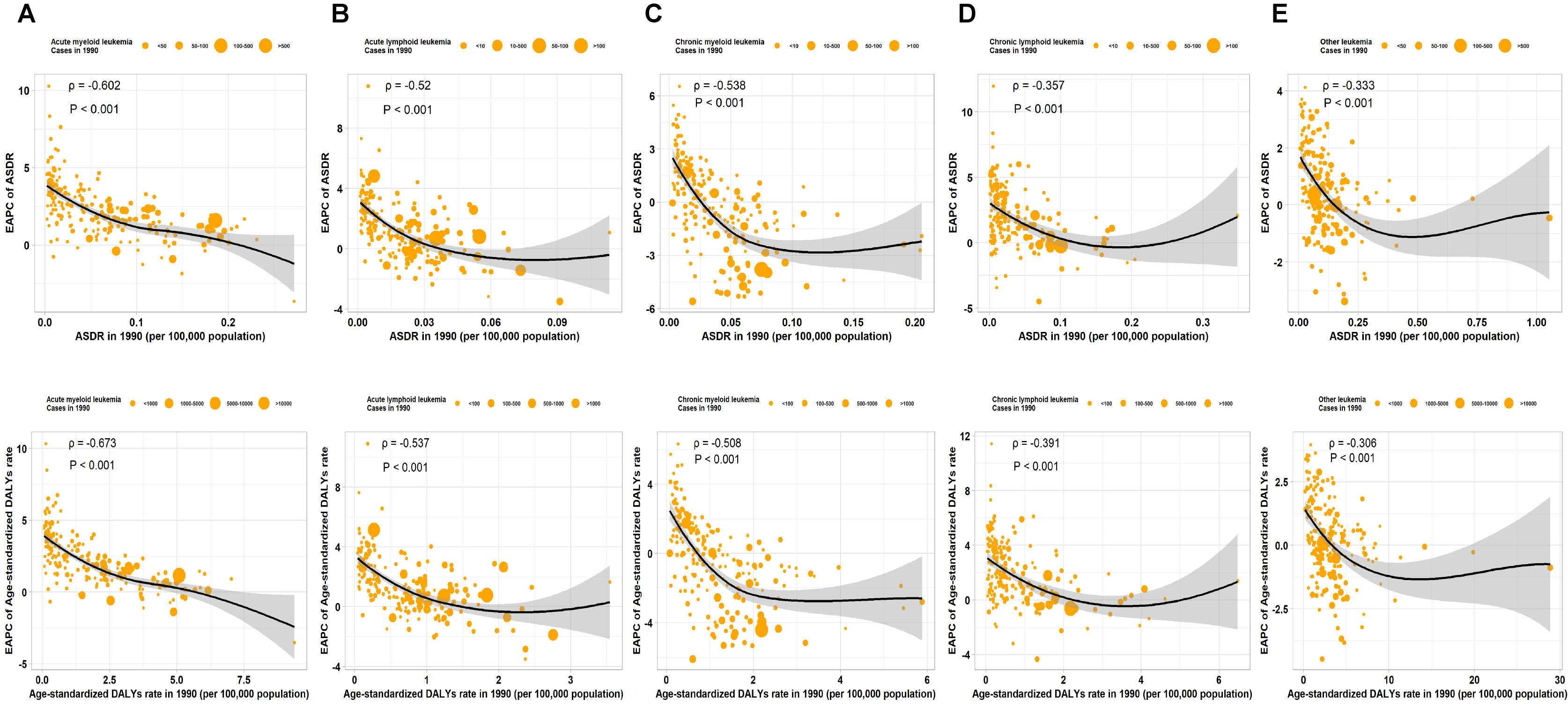

Supplement: Supplementary Figure S8 — The correlation between EAPC of ASR and ASR of 1990 for different subtypes of leukemia attributable to high BMI risk in 204 countries or territories. A AML B ALL C CML D CLL E other leukemia. AML acute myeloid leukemia, ALL acute lymphoblastic leukemia, CML chronic myeloid leukemia, CLL chronic lymphocytic leukemia. [file Image_8.tif]

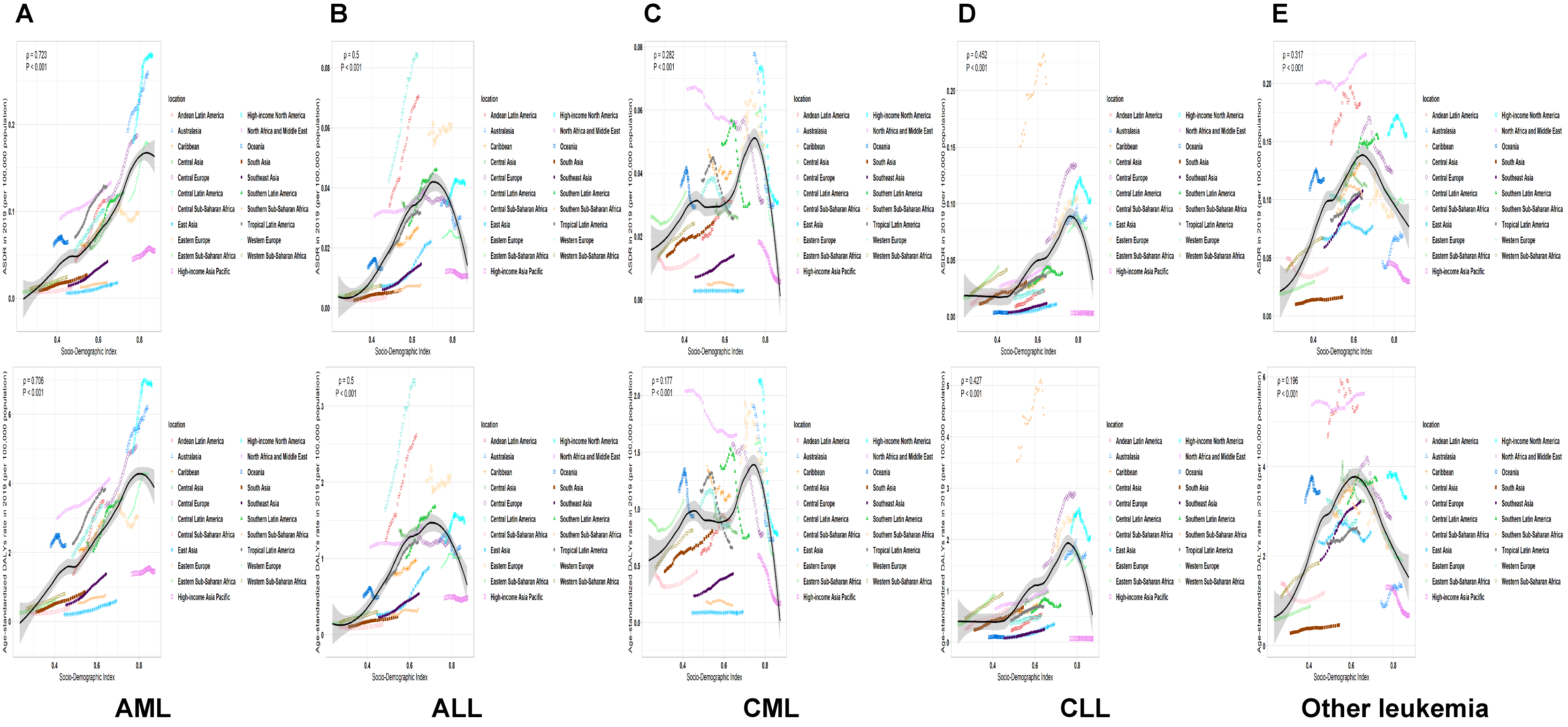

Supplement: Supplementary Figure S9 — The correlation between ASR of 2019 and SDI from 1990 to 2019 in different subtypes of leukemia attributable to high BMI risk in 21 regions. A AML B ALL C CML D CLL E other leukemia. AML acute myeloid leukemia, ALL acute lymphoblastic leukemia, CML chronic myeloid leukemia, CLL chronic lymphocytic leukemia. [file Image_9.tif]

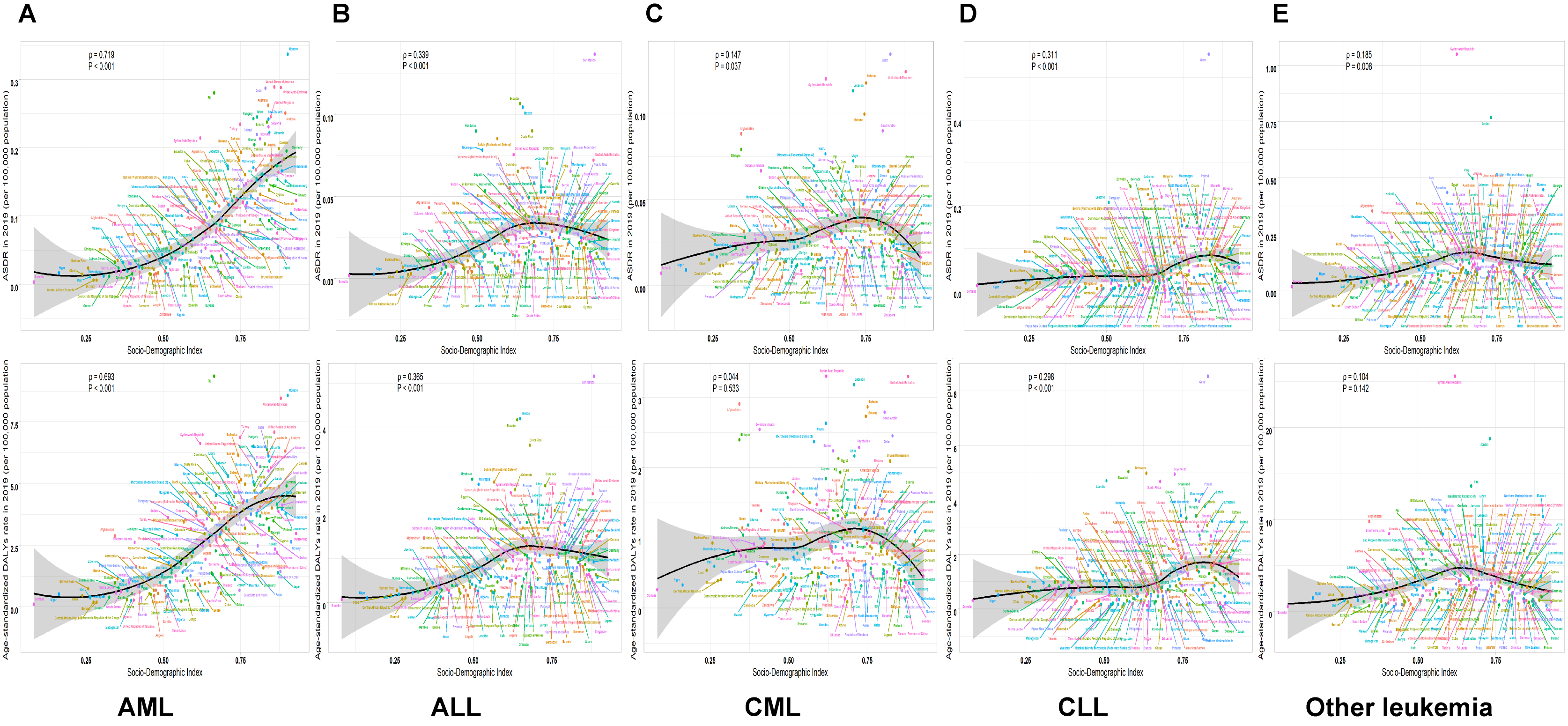

Supplement: Supplementary Figure S10 — The correlation between ASR of 2019 and SDI from 1990 to 2019 in different subtypes of leukemia attributable to high BMI risk in 204 countries and territories. A AML B ALL C CML D CLL E other leukemia. AML acute myeloid leukemia, ALL acute lymphoblastic leukemia, CML chronic myeloid leukemia, CLL chronic lymphocytic leukemia. [file Image_10.tif]
